# Mutations of RAS genes identified in acute myeloid leukemia affect glycerophospholipid metabolism pathway

**DOI:** 10.3389/fonc.2023.1280192

**Published:** 2023-11-14

**Authors:** Tianqi Liang, Yanxiang Kong, Hongman Xue, Wenqing Wang, Chunmou Li, Chun Chen

**Affiliations:** ^1^ Pediatric Hematology Laboratory, Division of Hematology/Oncology, Department of Pediatrics, The Seventh Affiliated Hospital of Sun Yat-Sen University, Shenzhen, Guangdong, China; ^2^ Department of Reproductive Medicine, The Seventh Affiliated Hospital, Sun Yat-sen University, Shenzhen, Guangdong, China

**Keywords:** acute myeloid leukemia, NRAS Q61K, KRAS G12V, glycerophospholipid metabolism, DGKzeta, PLA2G4A

## Abstract

**Background:**

Acute myeloid leukemia (AML) is a malignant disease originating from myeloid hematopoietic stem cells. Recent studies have shown that certain gene mutations promote tumor cell survival and affect the prognosis of patients by affecting metabolic mechanisms in tumor cells. *RAS* gene mutations are prevalent in AML, and the *RAS* signaling pathway is closely related to many metabolic pathways. However, the effects of different *RAS* gene mutations on AML cell metabolism are unclear.

**Objectives:**

The main purpose of this study was to explore the effect of *RAS* gene mutation on the metabolic pathway of tumor cells.

**Methods:**

In this study, we first used a retrovirus carrying a mutant gene to prepare Ba/F3 cell lines with *RAS* gene mutations, and then compared full-transcriptome data of Ba/F3 cells before and after *RAS* gene mutation and found that differentially expressed genes after NRAS^Q61K^ and KRAS^G12V^ mutation.

**Results:**

We found a total of 1899 differentially expressed genes after NRAS^Q61K^ and KRAS^G12V^ mutation. 1089 of these genes were involved in metabolic processes, of which 167 genes were enriched in metabolism-related pathways. In metabolism-related pathways, differential genes were associated with the lipid metabolism pathway. Moreover, by comparing groups, we found that the expression of the *DGKzeta* and *PLA2G4A* genes in the glycerophospholipid metabolism pathway was significantly upregulated.

**Conclusion:**

In conclusion, our study revealed that *RAS* gene mutation is closely related to the glycerophospholipid metabolism pathway in Ba/F3 cells, which may contribute to new precision therapy strategies and the development and application of new therapeutic drugs for AML.

## 1 Introduction

Acute myeloid leukemia (AML) is a heterogeneous hematologic malignancy originating from hematopoietic stem cells and is characterized by clonal proliferation and abnormal differentiation of myeloid cells in bone marrow and peripheral blood (1). AML is the most common leukemia in adults and the second most common acute leukemia in children, with high mortality and low overall survival in both adults and children (2). AML is accompanied by many kinds of cytogenetic abnormalities, and different cytogenetic abnormalities can significantly affect prognosis in AML (3). During AML pathogenesis, metabolic mechanisms are altered to meet the high demands of metabolic models established by cloning malignant tumor cells (4). By using different sources of nutrients for energy and biomass supply, AML cells exhibit metabolic plasticity and rapidly outcompete normal hematopoietic cells, leading to their high involvement in disease progression and resistance to treatment (5). The *RAS* oncogene has been identified as a key factor in the regulation of cell proliferation induced by retroviruses (6). The RAS protein encoded by this gene is a specialized guanine nucleotide-binding and hydrolyzing molecule that belongs to the small G-protein superfamily (7). Mutant Ras proteins differentially activate the RAF/MEK/ERK kinase cascade and other noncanonical downstream signaling molecules, which are closely related to tumorigenesis (8). In addition, studies have shown that the RAS protein family can significantly affect the metabolism of tumor cells and exert a significant impact on the metabolism of various organic compounds in tumor cells (9). Statistical analyses revealed a high incidence of *RAS* gene mutations in AML, especially in children (10). However, whether *RAS* gene mutations affect the metabolism of AML cells remains unclear.

In our study, by comparing changes in the transcriptome before and after *RAS* gene mutation, we identified key pathways and genes related to cell metabolism that are affected by *RAS* gene mutation, which may lead to the identification of new targets and strategies for the treatment of AML.

## 2 Materials and methods

### 2.1 Cell culture

Ba/F3 is an IL-3 dependent mouse pre B-cell line. Because it can survive independently of IL-3 after the introduction of a driving mutant gene, it has been used as a common tool to study the role of secondary mutant genes (11). In the published literature, Ba/F3 cell line was also used as a model cell to study AML (12–14). In our study, Ba/F3 cells were maintained in RPMI (Gibco, Thermo Fisher Scientific, USA) supplemented with 10% fetal bovine serum (FBS, FBS-S500), 100 U/ml penicillin, 100 µg/ml streptomycin (Gibco, Thermo Fisher Scientific, USA) and 1 ng/ml IL-3 (PeproTech, USA) at 37°C in a humidified atmosphere containing 5% CO_2_.

### 2.2 Generation of RAS gene-mutated Ba/F3 cells

Following the experimental method described by Chen et al (15), we used a retrovirus carrying a mutant gene to prepare Ba/F3 cell lines with *RAS* gene mutations. Retroviruses carrying the pMSCV-IRES-GFP plasmid vector harboring full-length KRAS-G12V and NRAS-Q61K were used, along with the pVSV-G plasmid, to transfect GP2-293 cells (16, 17). Recombinant retroviruses were isolated by centrifugation at 20000×g for 2 h, and these viruses were used to infect Ba/F3 cells in the presence of 5 μg/ml polybrene (Sigma–ldrich, USA) under centrifugation at 1800×g for 2 h at room temperature. Infected Ba/F3 cells were cultured in RPMI with 10% FBS in the presence of IL-3 for 24 h and then seeded in semisolid medium containing RPMI, 10% FBS, and 1% methylcellulose but not IL-3. Single colonies were selected after 8-10 days in culture and expanded in IL-3-free liquid medium. Ba/F3 cells successfully producing RAS gene mutations can grow independently of IL3.

### 2.3 Transcriptome analysis

After cultivation and further amplification, stable Ba/F3 parental, KRAS^G12V^ and NRAS^Q61K^ cell lines were obtained. The cells were collected in lyophilization tubes and frozen in liquid nitrogen for 10 minutes. Raw data and normalized gene expression data are deposited in the sequence read archive database under accession numbers PRJNA1006527. The isolation of RNA and next-generation sequencing were performed by Beijing Genomics Institute (Beijing, China). Gene Ontology (GO), Kyoto Encyclopedia of Genes and Genomes (KEGG) pathway, Venn diagram and heatmap analyses were performed with OmicShare tools, a free online platform used for data analysis (https://www.omicshare.com/tools/).

### 2.4 Statistical analysis

Data visualization and statistical analysis were carried out using GraphPad Prism 8.0 software (GraphPad Software Inc., CA, USA). Differences between experimental groups were analyzed for significance by unpaired Student’s t test. A *P* value <0.05 was considered significant.

## 3 Results

### 3.1 Mutations in the RAS gene significantly affect metabolic pathways in Ba/F3 cells

**Figure 1:**
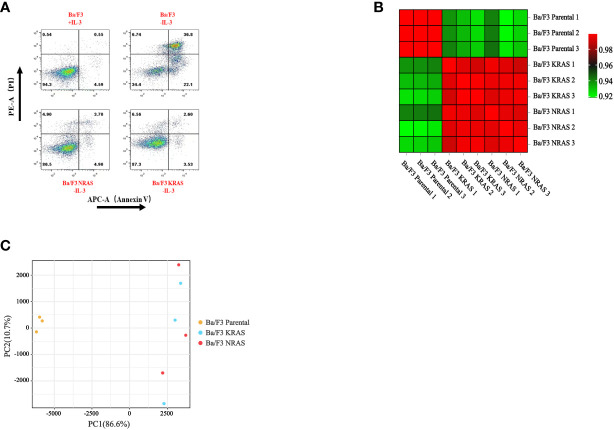
Changes in cellular characteristics after RAS gene mutation. **(A)** After the removal of IL-3, apoptosis occurred in wild-type Ba/F3 cells, and Ba/F3 cells with RAS gene mutations continued to grow. The heatmap **(B)** and principal component analysis **(C)** were used to analyze the intergroup variability before and after RAS gene mutation.

**Figure 2:**
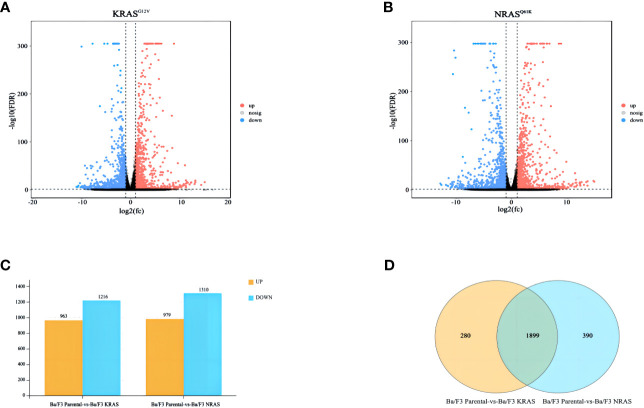
Differentially expressed genes after RAS gene mutation. **(A)** Differentially expressed genes after KRAS^G12V^ mutation. **(B)** Differentially expressed genes after NRAS^Q61K^ mutation. **(C)** Bar charts showing the number of significantly different genes between the two groups (FDR < 0.05, multiple differences greater than or equal to 2). **(D)** Venn diagram showing common differentially expressed genes.

We performed transcriptome analysis in 3 strains of cell lines, including the Ba/F3 parental strain, Ba/F3 KRAS^G12V^ strain and Ba/F3 NRAS^Q61K^ strain. Flow cytometry showed that KRAS^G12V^ and NRAS^Q61K^ mutant cell lines were successfully prepared. (**Figure 1A**). Heatmap analysis and principal component analysis revealed large intergroup variability and small intragroup variability before and after *RAS* gene mutation (**Figures 1B, C**). These data indicate an ideal cell line model for our transcriptome analysis. To explore the effect of *RAS* gene mutation, we analyzed whole-genome and full-transcriptome sequencing data of the Ba/F3 parental, KRAS^G12V^ and NRAS^Q61K^ cell lines. A volcano map shows that many genes were differentially expressed before and after the induction of the KRAS^G12V^ and NRAS^Q61K^ mutants. After KRAS^G12V^ induction, 963 genes were upregulated, and 1216 genes were downregulated. In addition, after NRAS^Q61K^ induction, 979 genes were upregulated, and 1310 genes were downregulated (**Figures 2A–C**; **Supplementary Tables S1**–**S4**). According to a Venn analysis, there were 1899 common differentially expressed genes (**Figure 2D**; **Supplementary Table S5**).

### 3.2 Genes after KRAS^G12V^ and NRAS^Q61K^ induction mainly affect the metabolism-related pathways of Ba/F3 cells

**Figure 3:**
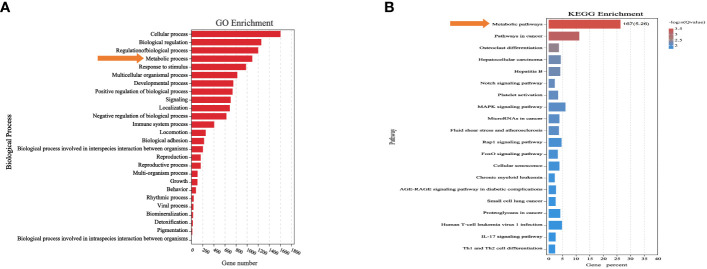
Differentially expressed genes were associated with metabolic processes and pathways. **(A)** Biological process significantly affected metabolic process. **(B)** Differentially expressed genes after RAS mutation were found to mainly affect metabolic pathways.

We analyzed the differentially expressed genes after induction of KRAS^G12V^ and NRAS^Q61K^ via Gene Ontology (GO) and Kyoto Encyclopedia of Genes and Genomes (KEGG) enrichment analysis. Finally, we found that a total of 1089 genes were involved in metabolic processes (**Supplementary Table S6**), of which 167 genes were enriched in metabolism-related pathways (*P*<0.05) (**Figures 3A, B**) (**Supplementary Table S7**).

### 3.3 Metabolism-related genes mainly affected glycerophospholipid metabolism after KRAS^G12V^ and NRAS^Q61K^ induction

**Figure 4:**
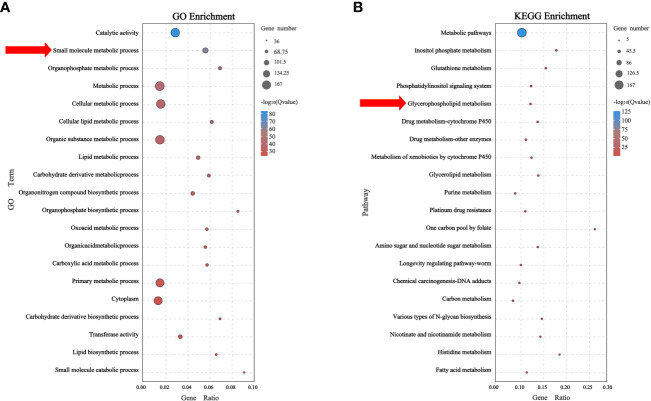
Metabolic-related genes mainly affect glycerophospholipid metabolism **(A)** Biological process of related genes affected small molecule metabolic process. **(B)** Related genes were found to mainly affect glycerophospholipid metabolism by KEGG enrichment analysis.

A total of 167 genes related to metabolic pathways were identified in Ba/F3 cells with *RAS* gene mutations. The GO analysis showed that these genes mainly affected the small molecule metabolic process in cells. To identify a specific metabolic pathway, we carried out a KEGG analysis. The results showed that these genes were enriched in multiple metabolic pathways, of which 12 genes were enriched in the glycerophospholipid metabolism pathway (*P*<0.05) (**Figures 4A, B**; **Supplementary Table S8**).

### 3.4 *DGKzeta* and *PLA2G4A* were key genes in the glycerophospholipid metabolism of Ba/F3 cells with RAS mutations

**Figure 5:**
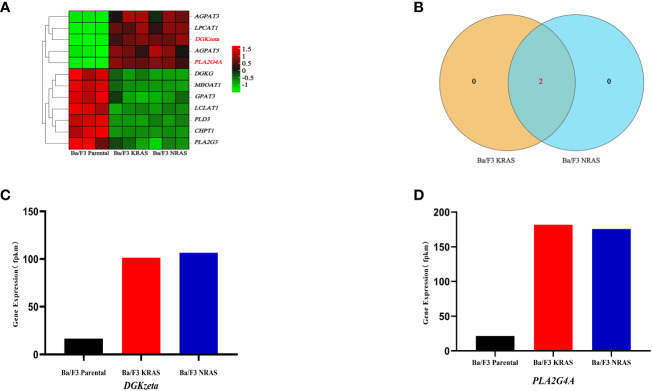
DGK*zeta* and *PLA2G4A* were candidate genes in glycerophospholipid metabolism. **(A)** The heatmap shows the expression of genes in different strains of cell lines. **(B)** The shared key genes were found by Venn analysis (FPKM>100). **(C, D)** Expression of key genes related to glycerophospholipid metabolism was detected by RNA-seq.

There are 12 genes involved in the regulation of glycerophospholipid metabolism, and the heatmap shows the differences in their expression among the Ba/F3 parental group, KRAS^G12V^ group and NRAS^Q61K^ group (**Figure 5A**). Through Venn analysis, 2 of the 12 genes involved in glycerophospholipid metabolism were found to be significantly upregulated and coexpressed in the KRAS^G12V^ and NRAS^Q61K^ mutant cell lines (FPKM>100) (**Figure 5B**). Gene expression analysis showed that the *DGK* ***zeta*** and *PLA2G4A* genes were increased significantly in both the KRAS^G12V^ and NRAS^Q61K^ mutant cell lines, and a significant difference was found between the Ba/F3 parental groups (*P*<0.05) (**Figures 5C, D**).

## 4 Discussion

Studies have shown that the original metabolic patterns in tumor cells change to meet the increased bioenergetic and biosynthetic demand during tumorigenesis and progression and to mitigate oxidative stress during the proliferation and survival of tumor cells (18). Studies on the metabolic mechanisms of tumor cells are helpful to explore the occurrence, progression, diagnosis and treatment of tumors. It has been proven that the fatty acids produced by lipid decomposition enter the tricarboxylic acid (TCA) cycle and oxidative phosphorylation (OXPHOS) metabolic pathway after oxidation by mitochondrial β-oxidation, thus producing ATP and NADPH to provide energy (5). More importantly, some special lipids produced by lipid metabolism can be used as essential lipid signaling molecules to regulate the biological processes of tumor cells. Meanwhile, the two upregulated genes *DGKzeta* and *PLA2G4A* found in our study are involved in lipid signal regulation, suggesting that RAS gene mutations in AML may have biological effects by affecting lipid signals.

Diacylglycerol (DAG) is a key secondary lipid messenger in signal transduction downstream of many receptors and plays an important role in driving adaptive and innate immune cell activation, proliferation, migration and effector functions (19). Diacylglycerol kinases (DGKs) can regulate the DAG signaling pathway by phosphorylating DAG and converting it into phosphatidic acid (PA) (20). DGK has 10 different isoforms, which are composed of five different classes of DGKs, each of which regulates different cellular functions according to its different structure and location in different cells. Studies have confirmed that DGKα is highly expressed in several refractory cancer cells, such as melanoma, hepatocellular carcinoma and glioblastoma. It can slow tumor cell apoptosis and promote cell proliferation (21). As an isoform of DGKα, DGKzeta is highly expressed in lymphoid tissues (22), which affects tumor cell apoptosis and cell cycle arrest. In human AML HL-60 cells, knockout of DGKzeta can induce apoptosis and G2/M phase arrest through the MAPK/survivin/caspase pathway (23). Our study found that the expression of DGKzeta was significantly upregulated after RAS gene mutation, indicating that DGKzeta may be the key factor affecting the regulation of AML cell proliferation after RAS gene mutation. DGKzeta has a negative regulatory effect on T cells (19), which can suppress the development of natural regulatory T cells and predominantly mediates Ras and Akt signaling downstream of the TCR (24). Interestingly, DGKzeta expression was also significantly upregulated after RAS gene mutation in our study, and whether it affects the immune escape of tumor cells needs to be further studied.

Phospholipase A2 enzymes (PLA2s) are the key enzymes of phospholipase metabolism. According to their location in the body, substrate specificity and differences in physiologic function, PLA2s can be divided into six subfamilies. Its function is to hydrolyze the sn-2 acyl bond of glycerol phospholipids (GPLs), release lysophospholipids (LPLs) and generate free fatty acids (25). These fatty acids are important energy sources for AML cells. PLA2G4A (cPLA2-IVA) belongs to a kind of cPLA2. In tumor cells, its activation is mainly regulated by the MAPK signaling pathway, and it is a key enzyme in AA metabolism (26). Overexpression of PLA2 can increase the release of AA and enhance the protumoral effects mediated by eicosanoids in promoting tumor survival, proliferation, antiapoptosis, transformation and metastasis (27). Studies have shown that cPLA2 plays a carcinogenic role in most cancers except colon cancer (28). Downregulation or deletion of cPLA2 can significantly inhibit the formation of small intestinal tumors induced by Apc(Min) and lung tumors induced by urethane (29, 30). Moreover, the inactivation of cPLA2 inhibits the occurrence of liver cancer (31) and the formation of prostate tumors (32). Using weighted gene coexpression network analysis to analyze the RNA sequencing data and clinicopathological characteristics of large samples of AML patients, it was found that the high expression of PLA2G4A was related to adverse overall survival (33). It was also found that PLA2G4A can be used as an independent prognostic marker in some specific types of AML. For example, in non-M3/nucleophosmin (NPM1) wild-type AML, patients with high expression of PLA2G4A had a significantly shorter overall survival rate. Moreover, some proteins with well-characterized oncogenic properties in AML, such as RUVBL2, CAP1, STAT3 and MYCBP, can physically interact with PLA2G4A (34). It has also been found that the high expression of PLA2G4A in FLT3-mutated AML is not only an indicator of poor prognosis but also related to drug resistance to tyrosine kinase inhibitors and changes in the tumor microenvironment of AML (35). Our study found that the expression of PLA2G4A was significantly upregulated after RAS gene mutation, which may be a potential therapeutic target for the treatment of AML with RAS gene mutation.

In conclusion, our study revealed that RAS gene mutations may affect cell metabolism. This effect may be achieved by altering the glycerophospholipid metabolism pathway. Among these candidate genes, *DGKzeta* and *PLA2G4A* were identified as key to cell metabolism. These results may provide a new strategy and therapeutic target for AML therapy with RAS gene mutations.

## Data availability statement

The datasets presented in this study can be found in online repositories. The names of the repository/repositories and accession number(s) can be found below: https://www.ncbi.nlm.nih.gov/, PRJNA1006527.

## Author contributions

TL: Methodology, Writing – original draft. YK: Writing – original draft. HX: Data curation, Writing – review & editing. WW: Data curation, Writing – review & editing. CL: Data curation, Writing – review & editing. CC: Project administration, Writing – original draft.
