# Supplementary material for: Mutations of RAS genes identified in acute myeloid leukemia affect glycerophospholipid metabolism pathway
Source: Front Oncol. 2023 Nov 14;13:1280192. doi: 10.3389/fonc.2023.1280192 (PMC10682766; doi:10.3389/fonc.2023.1280192)
Supplement: Supplementary file 1 [file DataSheet_1.pdf]

The up-regulated genes of KRAS（G12V）cell line

ENSEMBL	BaF3	BaF3 KRAS（G12V）	log2fc	FDR	Pvalue
ENSMUSG00000028214	0.001	36.852	15.1694553	7.27664E-17	7.2084E-18
ENSMUSG00000092586	0.001	22.263	14.44236039	5.13769E-11	7.73062E-12
ENSMUSG00000042351	0.001	9.675666667	13.24014535	3.89887E-20	3.19602E-21
ENSMUSG00000030638	0.001	9.623	13.23227101	1.00125E-16	1.0016E-17
ENSMUSG00000022583	0.001	8.66	13.08015131	7.72536E-13	1.00304E-13
ENSMUSG00000057596	0.001	5.448333333	12.41159926	2.39732E-16	2.44645E-17
ENSMUSG00000061815	0.001	5.180333333	12.33882922	1.08503E-15	1.15173E-16
ENSMUSG00000023993	0.001	4.263333333	12.05776614	5.44005E-12	7.5318E-13
ENSMUSG00000044912	0.001	3.329666667	11.70116204	1.28072E-15	1.36836E-16
ENSMUSG00000022053	0.001	2.951	11.52698821	2.65431E-17	2.55566E-18
ENSMUSG00000032690	0.001	2.854666667	11.47910658	5.45973E-15	6.1064E-16
ENSMUSG00000028217	0.001	2.57	11.32755264	8.14968E-14	9.83397E-15
ENSMUSG00000073001	0.001	2.560333333	11.32211593	4.17205E-06	1.01265E-06
ENSMUSG00000000182	0.011666667	29.52666667	11.30541036	1.5465E-20	1.24623E-21
ENSMUSG00000034923	0.001	2.525	11.30206767	8.14415E-09	1.48682E-09
ENSMUSG00000028536	0.001	2.495	11.2848241	6.31413E-09	1.13562E-09
ENSMUSG00000026582	0.042	93.18966667	11.11556495	5.43956E-52	1.54552E-53
ENSMUSG00000040328	0.001	1.896	10.88874325	8.44488E-11	1.2924E-11
ENSMUSG00000108929	0.001	1.874	10.87190524	9.8041E-13	1.28179E-13
ENSMUSG00000114635	0.001	1.736333333	10.76182822	1.44664E-09	2.4511E-10
ENSMUSG00000022659	0.001	1.716333333	10.74511405	2.17152E-10	3.43039E-11
ENSMUSG00000029553	0.001	1.480666667	10.53203118	3.42268E-09	6.02981E-10
ENSMUSG00000074344	0.001	1.310333333	10.35571815	7.25759E-06	1.80898E-06
ENSMUSG00000029372	0.001	1.283333333	10.32568023	1.80326E-06	4.20271E-07
ENSMUSG00000017897	0.001	1.125	10.13570929	3.09618E-09	5.42666E-10
ENSMUSG00000032062	0.001	1.082333333	10.07992917	1.08191E-06	2.4577E-07
ENSMUSG00000032258	0.001	1.059333333	10.04894091	8.87623E-11	1.36088E-11
ENSMUSG00000069892	0.006666667	5.865333333	9.781031791	6.55764E-19	5.79004E-20
ENSMUSG00000006522	0.001	0.851666667	9.734145075	1.04872E-06	2.3772E-07
ENSMUSG00000074491	0.001	0.817	9.674192268	4.91357E-05	1.36569E-05

ENSMUSG00000030742	0.099666667	80.15566667	9.651477722	2.16625E-56	5.77868E-58
ENSMUSG00000031750	0.001	0.798333333	9.64084744	1.40407E-06	3.23927E-07
ENSMUSG00000040759	0.001	0.761666667	9.573015949	0.000351937	0.000110336
ENSMUSG00000002076	0.001	0.744333333	9.539805035	9.99006E-07	2.25895E-07
ENSMUSG00000022215	0.001	0.728333333	9.508455064	0.000204355	6.17821E-05
ENSMUSG00000026829	0.001	0.725	9.501837185	3.38949E-05	9.21536E-06
ENSMUSG00000017724	0.001	0.687666667	9.425565605	4.41399E-07	9.6007E-08
ENSMUSG00000073413	0.001	0.643666667	9.330169948	0.011859877	0.004864239
ENSMUSG00000026572	0.001	0.636	9.312882955	5.42148E-07	1.19389E-07
ENSMUSG00000029586	0.001	0.614333333	9.262877855	4.71819E-06	1.15308E-06
ENSMUSG00000069893	0.001	0.606333333	9.243967327	1.43458E-06	3.31562E-07
ENSMUSG00000037833	0.001	0.6	9.22881869	8.60992E-07	1.93551E-07
ENSMUSG00000001020	0.161	96.13233333	9.221817253	1.17304E-15	1.24842E-16
ENSMUSG00000023333	0.001	0.559333333	9.1275645	4.75146E-06	1.16187E-06
ENSMUSG00000022938	0.001	0.526333333	9.039832955	0.004052816	0.001518926
ENSMUSG00000030410	0.008333333	4.106666667	8.944858446	4.17251E-08	8.11604E-09
ENSMUSG00000024034	0.001	0.488	8.930737338	1.85463E-06	4.32801E-07
ENSMUSG00000006389	0.9	419.774	8.865472095	0	0
ENSMUSG00000057135	0.001	0.433333333	8.759333407	0.002598097	0.000940513
ENSMUSG00000033730	0.005	2.112666667	8.722921349	9.53789E-12	1.34504E-12
ENSMUSG00000062980	0.009333333	3.845666667	8.686625582	2.45924E-15	2.68389E-16
ENSMUSG00000030465	0.001	0.405666667	8.664150952	6.15044E-09	1.1049E-09
ENSMUSG00000072115	0.001	0.404333333	8.659401334	0.022021503	0.009565853
ENSMUSG00000022623	0.045666667	16.82866667	8.525563674	4.33021E-88	6.9488E-90
ENSMUSG00000005611	0.638333333	219.0453333	8.422703777	2.2242E-155	1.6842E-157
ENSMUSG00000044303	0.001	0.319	8.317412614	0.033188624	0.015002318
ENSMUSG00000059336	0.036666667	11.23366667	8.259144067	9.91278E-37	4.37966E-38
ENSMUSG00000022622	0.001	0.296333333	8.211077108	0.014109358	0.005878981
ENSMUSG00000006344	0.005	1.464	8.193771743	6.49884E-08	1.29164E-08
ENSMUSG00000070469	0.001	0.269	8.071462363	6.37082E-08	1.26487E-08
ENSMUSG00000030769	0.001	0.265666667	8.053473413	0.001441274	0.000498721
ENSMUSG00000066510	0.001	0.264	8.044394119	0.006919439	0.002709132

ENSMUSG00000035441	0.001	0.249666667	7.963859408	8.68404E-06	2.18504E-06
ENSMUSG00000030350	0.014666667	3.635333333	7.953402821	2.99646E-12	4.07161E-13
ENSMUSG00000047343	0.001	0.235	7.876516947	0.041805649	0.019312787
ENSMUSG00000005131	0.001	0.229	7.839203788	0.011832545	0.004852206
ENSMUSG00000078616	0.001	0.207333333	7.695808269	0.011511112	0.0047092
ENSMUSG00000000320	0.001	0.206666667	7.691161905	0.000731483	0.000240457
ENSMUSG00000089694	0.001	0.197666667	7.626925794	0.031893128	0.014352461
ENSMUSG00000002365	0.042666667	8.407	7.622337858	5.01198E-40	2.00201E-41
ENSMUSG00000078921	0.028666667	5.58	7.604747153	4.07942E-21	3.19099E-22
ENSMUSG00000075065	0.001	0.191	7.577428828	7.64497E-05	2.18701E-05
ENSMUSG00000022219	0.017666667	3.299	7.544855109	9.96236E-09	1.8333E-09
ENSMUSG00000027204	0.008333333	1.553666667	7.542567608	3.63965E-22	2.69023E-23
ENSMUSG00000021699	0.001	0.186	7.539158811	0.002063936	0.000735103
ENSMUSG00000078920	0.180333333	32.48033333	7.492756535	4.72396E-82	8.20417E-84
ENSMUSG00000020447	0.001	0.160333333	7.324930583	0.000290522	8.98106E-05
ENSMUSG00000031824	0.001	0.157	7.294620749	0.000361725	0.000113556
ENSMUSG00000056656	0.001	0.15	7.22881869	0.006977306	0.002732758
ENSMUSG00000009772	0.007	0.957	7.095020192	1.711E-07	3.54444E-08
ENSMUSG00000001025	0.029333333	3.775666667	7.008046569	3.57199E-06	8.5956E-07
ENSMUSG00000032021	0.001	0.127333333	6.992466327	0.035179731	0.016002562
ENSMUSG00000064280	0.001	0.127	6.988684687	0.021393275	0.009272082
ENSMUSG00000069601	0.003666667	0.464666667	6.985583227	0.00090098	0.000301495
ENSMUSG00000022836	0.046	5.767666667	6.970210117	5.27282E-67	1.15017E-68
ENSMUSG00000070683	0.001	0.123	6.942514505	0.03519616	0.01601248
ENSMUSG00000030787	0.014333333	1.727666667	6.913306924	7.99055E-10	1.32556E-10
ENSMUSG00000018500	0.022666667	2.700333333	6.896421451	8.07711E-10	1.34048E-10
ENSMUSG00000106447	0.001	0.112666667	6.815916936	0.022773382	0.009933593
ENSMUSG00000026805	0.001	0.107333333	6.745954377	0.019046607	0.008158484
ENSMUSG00000040562	0.439	46.70166667	6.733109287	5.97124E-91	8.87701E-93
ENSMUSG00000057074	0.001	0.106	6.727920455	0.044073148	0.020482762
ENSMUSG00000030737	0.031333333	3.118666667	6.637087295	6.60922E-14	7.92004E-15
ENSMUSG00000031026	0.025333333	2.507666667	6.629164862	2.83168E-37	1.23535E-38

ENSMUSG00000020689	1.675666667	161.8493333	6.593772424	9.1622E-166	6.4285E-168
ENSMUSG00000035638	0.589	56.44666667	6.582476944	3.71027E-58	9.56241E-60
ENSMUSG00000029298	0.007333333	0.692333333	6.560853882	4.76379E-07	1.0388E-07
ENSMUSG00000029861	0.001	0.092	6.523561956	0.011223283	0.004581314
ENSMUSG00000022055	0.001	0.09	6.491853096	0.026191085	0.011544455
ENSMUSG00000061531	0.001	0.089666667	6.486499862	0.045879989	0.021424473
ENSMUSG00000074874	0.190666667	16.756	6.457481333	6.55951E-32	3.31279E-33
ENSMUSG00000020140	0.001	0.086	6.426264755	0.021393275	0.009272082
ENSMUSG00000028197	0.001	0.086	6.426264755	0.00064453	0.000209858
ENSMUSG00000030107	0.128333333	10.939	6.413441103	9.4985E-38	4.06466E-39
ENSMUSG00000029765	0.001666667	0.141	6.402585758	6.68441E-06	1.66007E-06
ENSMUSG00000044258	0.423333333	33.98466667	6.326946071	1.05979E-90	1.59024E-92
ENSMUSG00000020838	0.092666667	7.337	6.306995998	1.28035E-08	2.3837E-09
ENSMUSG00000027803	0.001	0.077666667	6.279223644	0.016439423	0.006943501
ENSMUSG00000059810	0.035666667	2.769666667	6.278992155	7.0108E-09	1.27164E-09
ENSMUSG00000022102	2.738666667	212.645	6.278829442	0	0
ENSMUSG00000042807	0.001	0.077333333	6.273018494	5.46825E-05	1.52936E-05
ENSMUSG00000031639	0.005	0.385666667	6.269282553	2.60205E-05	6.95206E-06
ENSMUSG00000031530	1.062333333	79.002	6.216580753	0	0
ENSMUSG00000073802	0.014333333	1.05	6.194871359	1.7057E-05	4.43873E-06
ENSMUSG00000054675	0.418	29.38833333	6.135596791	1.06118E-37	4.54842E-39
ENSMUSG00000030054	0.376333333	25.66333333	6.091553679	2.0065E-49	6.11918E-51
ENSMUSG00000026832	0.034666667	2.281	6.039973514	1.08877E-31	5.52895E-33
ENSMUSG00000042106	1.489666667	92.59133333	5.957815713	4.7805E-193	2.69E-195
ENSMUSG00000037820	1.271666667	72.43033333	5.831801553	0	0
ENSMUSG00000090958	0.005333333	0.3	5.813781191	0.000536232	0.000172846
ENSMUSG00000074577	0.229333333	12.833	5.806268598	1.21808E-93	1.74314E-95
ENSMUSG00000033207	0.549666667	30.321	5.785616532	5.1758E-232	2.2292E-234
ENSMUSG00000032261	0.001	0.054333333	5.763765654	0.04894244	0.023030849
ENSMUSG00000026715	0.01	0.536	5.744161096	0.005136387	0.001957144
ENSMUSG00000038963	2.192	117.465	5.743839345	0	0
ENSMUSG00000005824	1.592	82.704	5.699044866	1.6413E-271	5.5868E-274

ENSMUSG00000048148	0.02	1.035	5.693486957	1.50601E-20	1.21255E-21
ENSMUSG00000027636	1.603	80.796	5.65543754	0	0
ENSMUSG00000006445	0.010333333	0.515333333	5.640128294	3.85352E-06	9.31592E-07
ENSMUSG00000094151	0.011333333	0.516	5.508726915	0.000539571	0.00017396
ENSMUSG00000017737	0.084333333	3.740333333	5.470920058	1.22191E-10	1.89123E-11
ENSMUSG00000021280	0.399666667	17.21633333	5.428836853	3.9613E-78	7.29243E-80
ENSMUSG00000039109	6.287333333	268.7406667	5.417622589	0	0
ENSMUSG00000005125	12.084	512.5503333	5.406523692	0	0
ENSMUSG00000021675	0.352666667	14.74733333	5.386005073	4.66419E-89	7.25792E-91
ENSMUSG00000026579	0.48	19.38466667	5.335737711	1.8631E-181	1.1519E-183
ENSMUSG00000042345	0.612	22.81566667	5.220349346	7.9369E-153	6.3959E-155
ENSMUSG00000062937	15.85266667	582.3593333	5.199112165	0	0
ENSMUSG00000027784	0.002	0.071	5.14974712	0.00417221	0.001566281
ENSMUSG00000027239	0.058	2.042	5.137786156	2.35425E-05	6.25075E-06
ENSMUSG00000118672	0.001	0.035	5.129283017	0.004386291	0.001651829
ENSMUSG00000061751	0.148	5.169333333	5.126309153	3.80102E-28	2.17842E-29
ENSMUSG00000028184	0.004666667	0.160666667	5.105534414	0.008071505	0.003206173
ENSMUSG00000069833	0.042333333	1.423	5.07099776	1.87388E-40	7.43304E-42
ENSMUSG00000027765	5.311	177.172	5.060023281	0	0
ENSMUSG00000031028	0.003333333	0.109666667	5.040015679	0.011238334	0.004588238
ENSMUSG00000000244	2.242	72.866	5.022387612	1.122E-283	3.5855E-286
ENSMUSG00000007379	0.203666667	6.479666667	4.991637814	6.23723E-74	1.23055E-75
ENSMUSG00000033007	0.008	0.247333333	4.950312876	0.003878667	0.001449616
ENSMUSG00000057378	0.002666667	0.082	4.942514505	0.000209732	6.35388E-05
ENSMUSG00000029373	0.152333333	4.604333333	4.917688712	4.98181E-07	1.08877E-07
ENSMUSG00000032036	0.020333333	0.611666667	4.91082701	1.06804E-05	2.71627E-06
ENSMUSG00000026581	6.330333333	190.021	4.907733586	2.37683E-95	3.3023E-97
ENSMUSG00000002602	0.076	2.28	4.906890596	7.05237E-19	6.24645E-20
ENSMUSG00000026580	0.393666667	11.68666667	4.891745126	5.4017E-112	6.2666E-114
ENSMUSG00000022438	0.402666667	11.90266667	4.885554971	4.42204E-80	7.86413E-82
ENSMUSG00000057137	0.217333333	6.257666667	4.847643442	4.84763E-35	2.25291E-36
ENSMUSG00000024679	0.326666667	9.167333333	4.810610979	7.97307E-34	3.78299E-35

ENSMUSG00000024402	0.029	0.807333333	4.799039654	0.000480715	0.000153749
ENSMUSG00000020599	0.015	0.412666667	4.781942503	0.000412081	0.000130537
ENSMUSG00000031698	0.366666667	9.891	4.753575365	3.39677E-71	6.93749E-73
ENSMUSG00000021071	0.011	0.296333333	4.751645489	0.000808019	0.00026803
ENSMUSG00000029096	0.042666667	1.130333333	4.727495069	8.4349E-05	2.42646E-05
ENSMUSG00000090066	0.016666667	0.438	4.715893371	5.59485E-13	7.19854E-14
ENSMUSG00000079563	0.352333333	9.252	4.71475239	1.99269E-45	6.76918E-47
ENSMUSG00000002847	0.01	0.262333333	4.71332923	0.020457039	0.008826584
ENSMUSG00000026873	0.005333333	0.138	4.693486957	0.01010355	0.004090552
ENSMUSG00000024737	0.008666667	0.22	4.665882496	0.030332767	0.013580735
ENSMUSG00000000308	0.013333333	0.330666667	4.632268215	0.026774794	0.011818481
ENSMUSG00000049988	0.010666667	0.263	4.62388149	0.017775502	0.007563098
ENSMUSG00000029561	0.276333333	6.759666667	4.6124706	1.53015E-41	5.84634E-43
ENSMUSG00000000386	0.084	2.001333333	4.574428338	4.25375E-14	5.02944E-15
ENSMUSG00000047250	7.197666667	168.6626667	4.550467569	0	0
ENSMUSG00000021190	2.613666667	60.536	4.533646286	3.3425E-191	1.904E-193
ENSMUSG00000049744	0.346	7.928	4.51811302	4.44665E-14	5.26678E-15
ENSMUSG00000050675	0.415333333	9.350666667	4.49272766	5.43669E-68	1.15192E-69
ENSMUSG00000024409	0.025333333	0.566333333	4.482542624	0.012471564	0.005134178
ENSMUSG00000079110	0.310333333	6.827666667	4.459502054	4.8415E-26	3.0169E-27
ENSMUSG00000024401	0.315666667	6.932	4.456797824	1.09273E-30	5.69329E-32
ENSMUSG00000020990	0.059333333	1.297666667	4.450933198	3.74143E-07	8.08845E-08
ENSMUSG00000034457	0.013333333	0.290666667	4.44625623	0.003090347	0.001132233
ENSMUSG00000007035	0.207	4.421333333	4.416778833	5.28781E-08	1.04177E-08
ENSMUSG00000041762	0.081666667	1.719666667	4.396237792	1.63894E-19	1.39131E-20
ENSMUSG00000027070	0.001	0.021	4.392317423	0.022396052	0.009755637
ENSMUSG00000007659	4.664	96.63766667	4.372945927	0	0
ENSMUSG00000052334	0.377	7.776333333	4.366453633	9.88131E-35	4.61974E-36
ENSMUSG00000074899	0.064666667	1.272	4.297932614	1.46236E-40	5.78037E-42
ENSMUSG00000063450	0.005666667	0.111333333	4.296241451	0.019240483	0.008253559
ENSMUSG00000078776	0.002666667	0.052	4.285402219	0.027171099	0.01201795
ENSMUSG00000028435	0.011333333	0.220333333	4.28104362	0.025106183	0.011034861

ENSMUSG00000024885	0.084	1.630333333	4.278633826	1.45842E-07	3.00094E-08
ENSMUSG00000035547	0.183333333	3.539666667	4.271072484	4.51239E-20	3.71461E-21
ENSMUSG00000032014	0.522333333	9.526	4.18882787	4.38464E-35	2.02556E-36
ENSMUSG00000021453	0.284666667	5.143333333	4.175358182	1.00488E-14	1.14065E-15
ENSMUSG00000022212	0.01	0.179	4.161887682	0.041882067	0.01935193
ENSMUSG00000027315	0.098333333	1.743666667	4.14829991	5.10292E-12	7.05086E-13
ENSMUSG00000046916	1.453	25.55333333	4.136404889	2.8641E-122	3.084E-124
ENSMUSG00000021200	0.009333333	0.163333333	4.129283017	0.034808176	0.015811786
ENSMUSG00000037580	5.249333333	91.66866667	4.126222573	0	0
ENSMUSG00000035183	4.162666667	72.584	4.124071622	8.8031E-263	3.1189E-265
ENSMUSG00000039103	0.676333333	11.70033333	4.112671362	3.45579E-51	1.00589E-52
ENSMUSG00000020872	0.017	0.294	4.112209504	0.031096914	0.013959587
ENSMUSG00000039131	0.026	0.449333333	4.111202562	0.010264253	0.004160606
ENSMUSG00000046768	0.189333333	3.256333333	4.104248054	2.12779E-11	3.09228E-12
ENSMUSG00000022041	0.017	0.289333333	4.08912589	0.001473353	0.000510838
ENSMUSG00000024427	0.065333333	1.109333333	4.085729874	3.03469E-14	3.55435E-15
ENSMUSG00000026255	0.011	0.186	4.079727192	0.04698551	0.022028844
ENSMUSG00000024042	0.932	15.75233333	4.07909178	2.161E-177	1.3811E-179
ENSMUSG00000001211	5.18	86.70166667	4.065035724	1.04564E-48	3.29055E-50
ENSMUSG00000035168	0.011333333	0.189666667	4.064822001	6.56633E-05	1.85974E-05
ENSMUSG00000013089	2.207	36.74833333	4.057520285	2.4785E-220	1.1536E-222
ENSMUSG00000029862	0.014666667	0.243666667	4.054295977	0.002444011	0.000880829
ENSMUSG00000003420	0.065	1.057333333	4.023846742	6.34454E-05	1.79363E-05
ENSMUSG00000046402	0.007666667	0.124333333	4.019469864	0.04684029	0.021950996
ENSMUSG00000036718	2.024666667	31.89	3.977347785	9.6718E-199	5.3079E-201
ENSMUSG00000003206	1.219	18.97733333	3.96050725	5.37251E-58	1.39585E-59
ENSMUSG00000032661	0.289	4.493666667	3.958751714	1.0833E-32	5.32056E-34
ENSMUSG00000031799	10.301	159.332	3.951179741	0	0
ENSMUSG00000118623	0.118	1.824666667	3.950774169	1.2444E-11	1.77094E-12
ENSMUSG00000020787	2.514666667	38.53233333	3.937630471	4.2112E-213	2.0478E-215
ENSMUSG00000027947	0.006	0.091	3.922832139	0.046846053	0.021956952
ENSMUSG00000030137	8.467333333	124.8253333	3.881859264	4.0094E-255	1.5041E-257

ENSMUSG00000002699	10.59533333	153.9276667	3.860751681	0	0
ENSMUSG00000020407	0.328666667	4.744	3.851406959	4.85497E-14	5.7774E-15
ENSMUSG00000094430	0.090666667	1.297333333	3.838833154	0.002781659	0.001013339
ENSMUSG00000028332	1.246	17.454	3.808181729	1.99129E-51	5.74079E-53
ENSMUSG00000030257	0.768666667	10.69833333	3.798884143	3.0826E-146	2.741E-148
ENSMUSG00000059852	0.029	0.398333333	3.779851407	0.000994165	0.000334957
ENSMUSG00000020029	18.036	245.3766667	3.766046747	0	0
ENSMUSG00000029344	8.888666667	119.114	3.744232154	1.2965E-221	5.7643E-224
ENSMUSG00000031789	0.081666667	1.055	3.691351845	0.017876643	0.007613873
ENSMUSG00000044197	0.834333333	10.702	3.681112739	1.83914E-65	4.13949E-67
ENSMUSG00000035969	0.103666667	1.324	3.674879137	1.22251E-05	3.138E-06
ENSMUSG00000037868	0.154666667	1.936666667	3.646341453	1.02772E-11	1.45216E-12
ENSMUSG00000027351	5.288666667	65.64166667	3.633635917	0	0
ENSMUSG00000025867	0.012	0.148	3.624490865	0.007206789	0.00283165
ENSMUSG00000074604	6.811333333	80.348	3.560252971	1.2637E-105	1.5538E-107
ENSMUSG00000079481	0.081333333	0.947333333	3.541953502	0.000109677	3.20536E-05
ENSMUSG00000054568	0.023666667	0.273333333	3.52973298	0.029915553	0.013358609
ENSMUSG00000021624	0.068666667	0.783666667	3.512558296	0.000285935	8.83329E-05
ENSMUSG00000034664	34.154	387.0796667	3.502504068	0	0
ENSMUSG00000037348	2.281333333	24.64333333	3.433248248	3.2242E-104	3.9869E-106
ENSMUSG00000033722	0.181333333	1.93	3.411884792	1.3144E-18	1.18154E-19
ENSMUSG00000026959	0.010666667	0.112333333	3.396604781	0.042142566	0.019485997
ENSMUSG00000034041	10.57533333	111.3546667	3.396386977	9.9064E-305	2.8216E-307
ENSMUSG00000033191	6.222	65.344	3.392604471	3.1578E-280	1.031E-282
ENSMUSG00000089665	0.16	1.679666667	3.392031145	0.013468654	0.005582077
ENSMUSG00000057191	3.087	32.266	3.385737351	9.96957E-76	1.8838E-77
ENSMUSG00000030745	0.330666667	3.443666667	3.380495977	5.22506E-21	4.09801E-22
ENSMUSG00000019960	7.791	81.00833333	3.378189906	6.5161E-150	5.5225E-152
ENSMUSG00000028602	0.031	0.317333333	3.355658952	0.002951453	0.001078065
ENSMUSG00000034459	0.030666667	0.305	3.314065977	0.01179609	0.004835618
ENSMUSG00000075122	0.072333333	0.717	3.309240577	0.000278228	8.5836E-05
ENSMUSG00000032698	12.127	120.2003333	3.309146294	7.549E-180	4.7722E-182

ENSMUSG00000028456	0.017	0.168	3.304854582	0.001242009	0.000424759
ENSMUSG00000000290	8.062666667	78.144	3.276806122	1.2333E-294	3.684E-297
ENSMUSG00000049577	22.72633333	220.257	3.276751027	0	0
ENSMUSG00000061462	0.025	0.241333333	3.271027197	0.012714752	0.005239591
ENSMUSG00000068227	0.308333333	2.927666667	3.247188533	3.18493E-11	4.69497E-12
ENSMUSG00000015890	0.025	0.232333333	3.216196155	0.028665825	0.012730852
ENSMUSG00000032322	2.893	26.47733333	3.1941196	2.63743E-66	5.84466E-68
ENSMUSG00000074342	2.298666667	20.96866667	3.189365949	9.93332E-17	9.92987E-18
ENSMUSG00000055485	1.477666667	13.463	3.187607159	5.3424E-103	6.7175E-105
ENSMUSG00000028862	1.388333333	12.63	3.185428739	8.26014E-85	1.38864E-86
ENSMUSG00000022957	0.429666667	3.903333333	3.183416907	1.92258E-06	4.49305E-07
ENSMUSG00000047880	0.046666667	0.423333333	3.181329765	0.001829704	0.000645195
ENSMUSG00000030589	0.201	1.821333333	3.179727576	4.46745E-08	8.75069E-09
ENSMUSG00000035783	5.176333333	46.049	3.153167398	6.5894E-146	5.9051E-148
ENSMUSG00000024617	0.019666667	0.172333333	3.131377421	0.039322259	0.018056279
ENSMUSG00000040447	16.93733333	146.0246667	3.107933436	0	0
ENSMUSG00000031511	12.967	111.7106667	3.106850301	0	0
ENSMUSG00000048376	25.21	214.9043333	3.09162655	0	0
ENSMUSG00000070348	0.111	0.943333333	3.087207971	9.44749E-10	1.57644E-10
ENSMUSG00000056220	21.49933333	181.7146667	3.079311038	0	0
ENSMUSG00000032402	1.881	15.81333333	3.071569755	9.1968E-124	9.7111E-126
ENSMUSG00000031078	0.048666667	0.407666667	3.06638413	0.022413546	0.009765735
ENSMUSG00000039384	1.726333333	14.41033333	3.061320745	1.74606E-63	4.08769E-65
ENSMUSG00000032596	1.720333333	14.27733333	3.052966508	2.19262E-95	3.03113E-97
ENSMUSG00000024399	3.726666667	30.88666667	3.051026494	4.62495E-43	1.68677E-44
ENSMUSG00000003153	23.241	191.8243333	3.04504169	1.6524E-275	5.5098E-278
ENSMUSG00000007030	0.094333333	0.765333333	3.020248684	1.81951E-08	3.43426E-09
ENSMUSG00000048612	0.309333333	2.496333333	3.012576379	5.11432E-21	4.00761E-22
ENSMUSG00000036585	0.032666667	0.258333333	2.983342656	0.015354423	0.006445765
ENSMUSG00000055538	0.066	0.519	2.975196609	4.59803E-05	1.27352E-05
ENSMUSG00000042745	6.226	48.925	2.974194369	3.11358E-87	5.06132E-89
ENSMUSG00000045659	1.098666667	8.589	2.966736429	4.50773E-66	1.0052E-67

ENSMUSG00000051615	4.336333333	33.46833333	2.948249143	1.6796E-236	7.1173E-239
ENSMUSG00000072595	0.872666667	6.727666667	2.94660363	6.89321E-15	7.75756E-16
ENSMUSG00000042096	0.104333333	0.804	2.945995345	0.001054188	0.000356278
ENSMUSG00000022453	0.197333333	1.518666667	2.944098666	1.11575E-05	2.84692E-06
ENSMUSG00000032020	7.074666667	54.43566667	2.943818146	3.4254E-221	1.5705E-223
ENSMUSG00000020101	0.097	0.730666667	2.91315674	0.009252529	0.003720294
ENSMUSG00000040264	0.282666667	2.123666667	2.909383668	1.27261E-11	1.81321E-12
ENSMUSG00000038037	0.132	0.987333333	2.902999305	1.8248E-07	3.79792E-08
ENSMUSG00000030041	0.723	5.371666667	2.89330223	2.76354E-12	3.7436E-13
ENSMUSG00000054385	0.738666667	5.408	2.872099771	5.41272E-28	3.10963E-29
ENSMUSG00000061411	2.659	19.442	2.870220954	3.3481E-127	3.326E-129
ENSMUSG00000045092	1.052333333	7.676333333	2.8668256	5.35065E-43	1.95887E-44
ENSMUSG00000044786	1.709333333	12.42833333	2.862127174	8.8285E-28	5.10881E-29
ENSMUSG00000050195	0.024333333	0.176666667	2.860023991	0.031129799	0.013976512
ENSMUSG00000052353	0.019666667	0.142333333	2.85544921	0.004188666	0.001573041
ENSMUSG00000045942	0.145666667	1.05	2.849646644	0.042718733	0.019790985
ENSMUSG00000021876	0.393	2.812	2.838995377	1.82121E-09	3.119E-10
ENSMUSG00000046223	2.371333333	16.859	2.829748586	2.10699E-45	7.18673E-47
ENSMUSG00000051590	0.183333333	1.301	2.827079939	7.16339E-15	8.07157E-16
ENSMUSG00000045827	0.480333333	3.406333333	2.826111785	1.29859E-22	9.41807E-24
ENSMUSG00000031342	0.174	1.230333333	2.821890026	5.5625E-10	9.08857E-11
ENSMUSG00000043017	5.315	37.527	2.819787365	2.5578E-152	2.0789E-154
ENSMUSG00000066026	11.704	82.55333333	2.818324795	1.5676E-113	1.8078E-115
ENSMUSG00000037754	0.485	3.404333333	2.811315652	3.08569E-49	9.49608E-51
ENSMUSG00000034165	39.44733333	276.468	2.809112823	0	0
ENSMUSG00000026177	0.053666667	0.375333333	2.806074234	0.020025544	0.008626495
ENSMUSG00000068877	0.240333333	1.641666667	2.77205256	0.018346406	0.007838166
ENSMUSG00000049493	0.055333333	0.376666667	2.767067626	0.022995868	0.010035432
ENSMUSG00000057729	0.673333333	4.569333333	2.762590899	5.4823E-06	1.34896E-06
ENSMUSG00000034066	0.048	0.325333333	2.760812336	0.004296419	0.001615597
ENSMUSG00000062861	2.729666667	18.356	2.74945502	2.1128E-87	3.41982E-89
ENSMUSG00000042594	9.049666667	60.543	2.742023606	2.8415E-205	1.4607E-207

ENSMUSG00000040498	0.055333333	0.37	2.74130453	0.021148261	0.009152738
ENSMUSG00000001473	8.156	53.50033333	2.7136142	3.03191E-74	5.96062E-76
ENSMUSG00000026749	4.105666667	26.78066667	2.705503468	1.24412E-76	2.31624E-78
ENSMUSG00000029860	43.78166667	285.4896667	2.705039747	4.7742E-176	3.1176E-178
ENSMUSG00000025511	7.306333333	47.633	2.704741935	1.46834E-59	3.67212E-61
ENSMUSG00000040283	5.063333333	32.48233333	2.681495902	3.048E-204	1.6092E-206
ENSMUSG00000006494	2.376333333	15.22866667	2.679980508	3.29062E-65	7.45218E-67
ENSMUSG00000037003	0.290666667	1.855	2.673981647	4.28015E-14	5.06362E-15
ENSMUSG00000066684	0.530333333	3.375	2.669916167	0.000820122	0.0002725
ENSMUSG00000032643	6.48	41.20233333	2.668660323	2.16946E-90	3.28547E-92
ENSMUSG00000039431	0.754666667	4.791	2.666415356	4.34867E-22	3.22941E-23
ENSMUSG00000078137	0.083333333	0.527333333	2.6617496	1.29338E-05	3.33161E-06
ENSMUSG00000037169	1.082	6.778666667	2.64730103	3.38253E-19	2.9349E-20
ENSMUSG00000110206	4.137666667	25.77733333	2.639213694	6.92612E-51	2.02563E-52
ENSMUSG00000035314	0.033333333	0.207333333	2.63691458	0.018011053	0.007678627
ENSMUSG00000042842	0.732666667	4.554666667	2.636116589	8.67664E-18	8.15526E-19
ENSMUSG00000074364	10.401	64.55566667	2.633821498	2.9734E-158	2.2309E-160
ENSMUSG00000030539	4.032	24.97966667	2.631186682	1.73275E-83	2.96114E-85
ENSMUSG00000022014	1.067666667	6.567666667	2.620919609	4.56937E-12	6.30094E-13
ENSMUSG00000098112	37.33433333	228.742	2.615146413	3.6098E-114	4.1126E-116
ENSMUSG00000040479	16.57866667	101.473	2.613696017	6.0671E-250	2.3181E-252
ENSMUSG00000002104	2.024333333	12.34366667	2.608252235	2.46154E-32	1.22778E-33
ENSMUSG00000079492	0.292333333	1.782333333	2.608080929	8.42731E-05	2.42369E-05
ENSMUSG00000050721	7.780666667	47.41266667	2.607306859	1.788E-168	1.2421E-170
ENSMUSG00000023830	0.021	0.127666667	2.603920658	0.008465648	0.003380378
ENSMUSG00000034663	11.84833333	71.436	2.591967166	8.94648E-42	3.3996E-43
ENSMUSG00000022148	37.89933333	227.4126667	2.585068237	7.6081E-93	1.1099E-94
ENSMUSG00000032366	23.66766667	141.7893333	2.582759618	2.5038E-163	1.809E-165
ENSMUSG00000074305	6.839	40.88766667	2.579808441	2.5974E-208	1.2992E-210
ENSMUSG00000040533	0.305333333	1.824333333	2.578912353	2.63758E-06	6.26092E-07
ENSMUSG00000030032	0.163333333	0.964666667	2.562211268	6.2404E-10	1.02482E-10
ENSMUSG00000027646	0.042333333	0.25	2.562062099	0.029263426	0.013036912

ENSMUSG00000033998	0.076666667	0.451666667	2.558587085	0.023611167	0.010321992
ENSMUSG00000032595	0.043	0.253	2.55672882	0.011980391	0.004920271
ENSMUSG00000032413	3.474666667	20.154	2.536119713	2.47019E-88	3.92965E-90
ENSMUSG00000079625	0.196	1.125333333	2.521426844	0.000852091	0.000283892
ENSMUSG00000022456	0.662333333	3.794666667	2.518343789	6.46445E-27	3.87553E-28
ENSMUSG00000005686	2.170333333	12.37733333	2.51171198	1.72967E-68	3.59272E-70
ENSMUSG00000036687	0.056	0.318333333	2.5070395	0.020838264	0.009008441
ENSMUSG00000053560	14.055	79.77033333	2.504768853	4.19246E-19	3.66094E-20
ENSMUSG00000062593	9.445	53.35933333	2.49811794	0.000154528	4.60523E-05
ENSMUSG00000031827	52.00166667	293.7093333	2.497759344	6.2342E-239	2.5552E-241
ENSMUSG00000053310	4.764333333	26.78633333	2.491150851	4.94908E-39	2.0422E-40
ENSMUSG00000038517	3.573333333	19.84466667	2.473408924	9.46344E-78	1.74872E-79
ENSMUSG00000031497	1.335	7.358333333	2.46253929	3.02498E-23	2.12873E-24
ENSMUSG00000096472	5.559	30.36466667	2.449496245	1.60295E-75	3.05112E-77
ENSMUSG00000037966	6.500666667	35.31133333	2.441471713	5.4735E-59	1.37645E-60
ENSMUSG00000071656	0.412333333	2.234666667	2.438176649	4.98212E-11	7.47924E-12
ENSMUSG00000024300	1.008333333	5.395333333	2.419739453	1.06304E-15	1.12618E-16
ENSMUSG00000097075	0.343333333	1.837	2.41966979	0.000807295	0.000267678
ENSMUSG00000022037	34.267	182.9436667	2.416507674	1.7677E-199	9.5786E-202
ENSMUSG00000069804	0.071333333	0.379666667	2.412085045	0.011561978	0.004734696
ENSMUSG00000075415	27.47833333	146.1966667	2.411544011	2.3789E-290	7.4366E-293
ENSMUSG00000021250	1.297666667	6.898333333	2.410327999	5.57794E-16	5.7775E-17
ENSMUSG00000030555	0.287333333	1.523666667	2.406750045	6.35671E-07	1.40912E-07
ENSMUSG00000059900	3.718333333	19.6	2.398125641	1.22654E-33	5.85365E-35
ENSMUSG00000039699	0.527	2.748666667	2.382857094	3.99637E-05	1.09661E-05
ENSMUSG00000040483	0.319333333	1.664	2.381520373	0.009232224	0.003711489
ENSMUSG00000039005	1.574666667	8.185666667	2.378053455	9.94148E-50	2.98348E-51
ENSMUSG00000059895	46.43733333	240.7433333	2.374138815	4.5913E-123	4.88E-125
ENSMUSG00000025492	22.428	116.2196667	2.373481342	5.74853E-44	2.03664E-45
ENSMUSG00000038172	3.781666667	19.556	2.370517202	1.9818E-118	2.189E-120
ENSMUSG00000046806	3.287	16.89666667	2.361895302	4.16793E-75	8.02027E-77
ENSMUSG00000021823	29.02333333	148.146	2.351734546	2.0346E-117	2.2614E-119

ENSMUSG00000033306	2.084	10.52466667	2.336347359	2.18752E-99	2.8569E-101
ENSMUSG00000050914	1.419666667	7.148666667	2.332121954	2.54876E-07	5.38789E-08
ENSMUSG00000035390	0.07	0.349333333	2.319177484	0.031400716	0.014104691
ENSMUSG00000042228	17.62533333	87.88333333	2.317939049	3.662E-266	1.272E-268
ENSMUSG00000054150	8.426	41.75366667	2.308983075	1.4224E-137	1.3142E-139
ENSMUSG00000028465	103.3193333	508.172	2.298206646	2.0257E-244	8.021E-247
ENSMUSG00000026657	3.013333333	14.752	2.291478384	5.97893E-81	1.04668E-82
ENSMUSG00000055322	5.494666667	26.82566667	2.287510157	4.6533E-155	3.5881E-157
ENSMUSG00000060600	13.20166667	64.30066667	2.284113619	2.35017E-66	5.19176E-68
ENSMUSG00000030659	0.204333333	0.987666667	2.273099647	0.001765612	0.000621122
ENSMUSG00000000184	26.86733333	129.5756667	2.269869775	5.75146E-31	2.96862E-32
ENSMUSG00000027296	0.112666667	0.541	2.263567848	0.023167871	0.010115322
ENSMUSG00000047798	1.535333333	7.344	2.258014148	1.33833E-18	1.20492E-19
ENSMUSG00000037706	3.151333333	15.07166667	2.257804694	1.1671E-27	6.80236E-29
ENSMUSG00000026180	2.351666667	11.22566667	2.255045639	6.36571E-06	1.5765E-06
ENSMUSG00000024397	0.393666667	1.878	2.254150599	0.00837724	0.003341003
ENSMUSG00000030263	11.379	54.22366667	2.252548896	9.8753E-152	8.0951E-154
ENSMUSG00000031990	0.221333333	1.043333333	2.23690751	0.000531669	0.000171079
ENSMUSG00000027078	2.588666667	12.14033333	2.229526922	1.86642E-22	1.3614E-23
ENSMUSG00000037824	7.076	33.12366667	2.22685643	2.26593E-54	6.17052E-56
ENSMUSG00000044551	1.522666667	7.126333333	2.226559817	6.06993E-22	4.54559E-23
ENSMUSG00000046688	1.622666667	7.581666667	2.224148361	2.65162E-22	1.94631E-23
ENSMUSG00000059430	23.752	110.724	2.220847064	6.9401E-54	1.91401E-55
ENSMUSG00000040253	0.451666667	2.102333333	2.218661082	9.97763E-12	1.40844E-12
ENSMUSG00000025701	0.245666667	1.143	2.21805138	2.87591E-05	7.7377E-06
ENSMUSG00000061859	2.525666667	11.71133333	2.213169183	2.22524E-37	9.64607E-39
ENSMUSG00000052142	11.63733333	53.56866667	2.202628882	8.5705E-172	5.8347E-174
ENSMUSG00000037336	0.603666667	2.773333333	2.199796982	1.48286E-09	2.5135E-10
ENSMUSG00000047419	0.359	1.645333333	2.196324145	4.74551E-26	2.95049E-27
ENSMUSG00000037946	2.102333333	9.623666667	2.194595241	3.35259E-42	1.25067E-43
ENSMUSG00000016552	0.079333333	0.362333333	2.191318462	0.000647563	0.000210981
ENSMUSG00000054404	0.894333333	4.083333333	2.19086279	4.61151E-09	8.20749E-10

ENSMUSG00000024887	1.663333333	7.590666667	2.190149284	9.94148E-47	3.26663E-48
ENSMUSG00000023328	0.422333333	1.927	2.189902548	0.00017175	5.14476E-05
ENSMUSG00000039968	11.66666667	52.742	2.176559859	3.6616E-140	3.3322E-142
ENSMUSG00000030144	2.137333333	9.633333333	2.172223162	1.0254E-09	1.71815E-10
ENSMUSG00000074151	1.098333333	4.918	2.16275577	2.1654E-29	1.18988E-30
ENSMUSG00000031557	21.45966667	95.42833333	2.152790009	1.70424E-65	3.82403E-67
ENSMUSG00000018909	4.955	22.02733333	2.152337888	4.0187E-119	4.411E-121
ENSMUSG00000034758	1.428666667	6.310333333	2.143046866	4.65937E-18	4.32436E-19
ENSMUSG00000005609	39.47933333	174.1703333	2.141329376	7.445E-237	3.1032E-239
ENSMUSG00000091780	4.383666667	19.33566667	2.141054502	4.90868E-30	2.62228E-31
ENSMUSG00000038205	0.986333333	4.337	2.136550247	3.65725E-17	3.5696E-18
ENSMUSG00000028300	0.566	2.486	2.134952338	1.15944E-12	1.53035E-13
ENSMUSG00000060216	36.29	159.3263333	2.134340772	3.1638E-161	2.3517E-163
ENSMUSG00000000562	0.724333333	3.179666667	2.134149858	3.55261E-09	6.27106E-10
ENSMUSG00000030788	11.19466667	49.133	2.13388076	2.3184E-133	2.1743E-135
ENSMUSG00000034738	1.818	7.966	2.13150328	1.14993E-17	1.08402E-18
ENSMUSG00000018166	8.932	39.01633333	2.127023047	2.85268E-14	3.33324E-15
ENSMUSG00000044367	0.82	3.576	2.124650922	1.88263E-16	1.91468E-17
ENSMUSG00000027397	9.072	39.54166667	2.123881136	3.07704E-67	6.62648E-69
ENSMUSG00000040061	16.13166667	70.22266667	2.122041282	5.04455E-99	6.6583E-101
ENSMUSG00000059456	9.863666667	42.89433333	2.120591118	6.2132E-109	7.3376E-111
ENSMUSG00000028970	2.770333333	12.046	2.120422684	1.57179E-44	5.45949E-46
ENSMUSG00000001227	2.271666667	9.876	2.12017568	1.01625E-32	4.98416E-34
ENSMUSG00000031749	2.864	12.42833333	2.117529443	4.04004E-38	1.69797E-39
ENSMUSG00000094347	44.856	194.2876667	2.114821442	1.5963E-221	7.208E-224
ENSMUSG00000036931	0.881666667	3.805333333	2.109717612	7.02635E-12	9.84518E-13
ENSMUSG00000026566	27.36533333	118.013	2.108524457	2.4153E-172	1.6275E-174
ENSMUSG00000045671	6.152666667	26.35933333	2.099030144	8.39433E-90	1.29458E-91
ENSMUSG00000049916	3.54	15.16066667	2.09851193	1.58369E-18	1.43131E-19
ENSMUSG00000000823	5.337333333	22.79166667	2.094315407	2.9236E-86	4.81343E-88
ENSMUSG00000043811	1.160666667	4.902333333	2.078514881	4.1782E-13	5.32035E-14
ENSMUSG00000029442	0.776333333	3.272333333	2.075571576	1.47846E-15	1.58784E-16

ENSMUSG00000039031	6.893333333	28.73266667	2.05941821	6.8526E-120	7.4739E-122
ENSMUSG00000050014	1.523666667	6.350333333	2.059285004	1.23256E-23	8.53673E-25
ENSMUSG00000047045	14.70066667	61.24966667	2.058820411	6.6564E-180	4.1617E-182
ENSMUSG00000034107	0.109	0.453666667	2.057304526	0.015583368	0.006553783
ENSMUSG00000043881	0.139666667	0.578333333	2.049913514	6.59209E-05	1.86795E-05
ENSMUSG00000039987	3.489666667	14.27533333	2.032363292	2.45712E-79	4.438E-81
ENSMUSG00000044229	0.463	1.868	2.012410356	1.15198E-08	2.1311E-09
ENSMUSG00000025877	7.047	28.20966667	2.0011085	0.047673864	0.022381381
ENSMUSG00000024236	10.35566667	41.42166667	1.999965171	9.52263E-60	2.36164E-61
ENSMUSG00000061143	2.085333333	8.334666667	1.99884649	1.26369E-76	2.36147E-78
ENSMUSG00000014606	46.905	186.4056667	1.990632093	5.515E-190	3.2182E-192
ENSMUSG00000024381	3.835333333	15.22833333	1.98933418	4.12674E-37	1.80607E-38
ENSMUSG00000041012	0.485333333	1.926	1.988559848	0.004369142	0.001644461
ENSMUSG00000035697	22.167	87.66966667	1.983664228	2.5941E-155	1.9823E-157
ENSMUSG00000031778	0.291	1.149	1.98128774	1.92078E-05	5.03628E-06
ENSMUSG00000000791	1.850333333	7.297333333	1.979584163	1.23169E-24	8.17134E-26
ENSMUSG00000074785	0.456333333	1.799333333	1.97930253	4.21279E-15	4.66495E-16
ENSMUSG00000036834	0.094	0.370333333	1.978091749	0.01351178	0.005600889
ENSMUSG00000036553	0.075	0.294	1.970853654	0.019045272	0.008156589
ENSMUSG00000027111	1.477333333	5.790666667	1.970734071	5.1122E-20	4.22968E-21
ENSMUSG00000052681	68.61566667	268.904	1.970481293	1.36889E-75	2.5961E-77
ENSMUSG00000018199	2.558333333	10.01966667	1.969558359	2.3816E-94	3.37511E-96
ENSMUSG00000049225	15.88633333	61.596	1.955050486	9.20913E-08	1.85654E-08
ENSMUSG00000045838	0.918333333	3.554666667	1.952624462	1.49344E-19	1.26364E-20
ENSMUSG00000074918	9.844333333	38.01766667	1.949304575	3.6856E-12	5.04898E-13
ENSMUSG00000066721	0.950333333	3.667333333	1.948225864	1.47718E-12	1.96512E-13
ENSMUSG00000053835	0.652333333	2.511	1.944580773	1.13706E-07	2.3152E-08
ENSMUSG00000020527	2.63	10.06633333	1.936403572	2.33864E-38	9.79645E-40
ENSMUSG00000047953	0.213333333	0.813333333	1.930737338	0.002956689	0.001080182
ENSMUSG00000070690	0.622666667	2.371333333	1.929166519	2.06143E-08	3.90232E-09
ENSMUSG00000049709	0.701666667	2.671333333	1.928702276	2.05547E-12	2.763E-13
ENSMUSG00000048200	0.519666667	1.966333333	1.919849481	0.000130198	3.8458E-05

ENSMUSG00000032373	3.794666667	14.358	1.919809736	2.66044E-47	8.68639E-49
ENSMUSG00000022094	4.172666667	15.76633333	1.917805601	5.19794E-75	1.00384E-76
ENSMUSG00000013707	3.123666667	11.787	1.91588416	2.06372E-14	2.38557E-15
ENSMUSG00000042622	0.769666667	2.882666667	1.90509835	2.23909E-07	4.70529E-08
ENSMUSG00000041220	7.843666667	29.29533333	1.901070734	3.21245E-93	4.64181E-95
ENSMUSG00000039013	2.113333333	7.889333333	1.900383055	4.18054E-11	6.23523E-12
ENSMUSG00000053113	1.703333333	6.351666667	1.898774412	3.6435E-17	3.55364E-18
ENSMUSG00000055202	0.192	0.714	1.894817763	0.000653198	0.000212998
ENSMUSG00000008318	3.009333333	11.132	1.887196991	1.38238E-07	2.83677E-08
ENSMUSG00000037679	9.409666667	34.77933333	1.886014757	3.00421E-68	6.32355E-70
ENSMUSG00000001435	0.256	0.942333333	1.880093667	0.001875704	0.000663109
ENSMUSG00000086322	0.18	0.661333333	1.877380713	0.034073386	0.015442499
ENSMUSG00000102752	0.337666667	1.240333333	1.877056216	2.13493E-06	5.0218E-07
ENSMUSG00000074802	3.829	14.04066667	1.874571873	1.81453E-26	1.10733E-27
ENSMUSG00000039063	0.719666667	2.630333333	1.869844896	1.48187E-07	3.05021E-08
ENSMUSG00000027314	0.098333333	0.359	1.86823139	0.015368093	0.00645257
ENSMUSG00000021322	0.245333333	0.889333333	1.857980995	0.000466252	0.000148928
ENSMUSG00000078853	0.548666667	1.983666667	1.854167782	4.41792E-05	1.22057E-05
ENSMUSG00000027368	1.28	4.623666667	1.852893583	5.47841E-07	1.20681E-07
ENSMUSG00000028771	26.558	95.82466667	1.851250569	8.919E-104	1.1091E-105
ENSMUSG00000020604	0.348	1.251333333	1.846306938	0.000167378	5.00798E-05
ENSMUSG00000105867	0.475333333	1.706333333	1.843888025	5.096E-05	1.41959E-05
ENSMUSG00000056515	17.62933333	63.11533333	1.840012619	5.268E-175	3.5132E-177
ENSMUSG00000060579	0.727666667	2.579666667	1.825835029	0.001865152	0.000658731
ENSMUSG00000020134	8.26	29.193	1.821408789	1.96611E-91	2.89555E-93
ENSMUSG00000041920	13.942	49.16866667	1.818301701	2.69133E-99	3.5336E-101
ENSMUSG00000079184	5.480333333	19.321	1.817834215	3.04513E-61	7.42509E-63
ENSMUSG00000031328	77.68866667	273.8553333	1.81763792	7.3744E-183	4.5081E-185
ENSMUSG00000046168	0.163333333	0.575333333	1.81657881	0.02884763	0.01282963
ENSMUSG00000019920	21.24333333	74.77133333	1.8154751	1.0845E-151	8.9654E-154
ENSMUSG00000006587	0.203666667	0.715666667	1.813077906	0.02525626	0.011106087
ENSMUSG00000044244	0.095333333	0.334666667	1.811672217	0.036456649	0.016625647

ENSMUSG00000031355	19.49333333	68.366	1.810298207	8.0325E-126	8.3143E-128
ENSMUSG00000038473	0.409666667	1.426666667	1.800125881	0.000788652	0.000261058
ENSMUSG00000043157	1.073	3.713	1.790935239	3.60087E-07	7.76458E-08
ENSMUSG00000036622	7.480333333	25.86766667	1.78997546	6.28495E-68	1.34038E-69
ENSMUSG00000022791	15.899	54.938	1.788868363	2.19554E-55	5.93307E-57
ENSMUSG00000047996	2.006	6.916333333	1.785685796	4.65139E-20	3.83873E-21
ENSMUSG00000018169	1.523666667	5.218666667	1.776133937	3.68196E-11	5.47114E-12
ENSMUSG00000026727	50.13	171.5113333	1.774557772	1.2675E-120	1.3736E-122
ENSMUSG00000040350	0.341	1.163666667	1.770834212	0.001669788	0.000585093
ENSMUSG00000112023	4.159333333	14.175	1.768924521	2.69493E-09	4.69155E-10
ENSMUSG00000038563	8.664	29.486	1.766924975	5.93406E-89	9.27519E-91
ENSMUSG00000006641	3.02	10.25566667	1.763800822	6.35986E-17	6.27812E-18
ENSMUSG00000027195	25.71633333	86.564	1.75108221	8.9657E-135	8.3459E-137
ENSMUSG00000030672	1.494666667	5.029	1.750447776	8.69123E-05	2.50201E-05
ENSMUSG00000092216	0.367	1.234	1.749490426	0.03176463	0.014288015
ENSMUSG00000030281	4.13	13.87933333	1.748724586	1.0586E-14	1.20458E-15
ENSMUSG00000015745	10.80566667	36.22066667	1.745025017	3.8947E-31	2.00214E-32
ENSMUSG00000002997	31.75866667	106.193	1.74146642	1.2946E-107	1.5648E-109
ENSMUSG00000026784	3.908	13.03666667	1.738072663	5.54552E-16	5.73622E-17
ENSMUSG00000028063	10.85966667	36.16033333	1.735428156	5.9444E-49	1.8624E-50
ENSMUSG00000038712	15.221	50.419	1.727904359	1.0459E-56	2.76824E-58
ENSMUSG00000075706	58.95533333	195.2346667	1.72751501	1.3843E-102	1.7503E-104
ENSMUSG00000020788	49.502	163.4476667	1.723270062	1.6917E-216	8.1091E-219
ENSMUSG00000074923	1.055	3.459333333	1.713251036	2.30841E-12	3.11103E-13
ENSMUSG00000022020	2.682666667	8.787	1.71170289	4.89029E-31	2.52073E-32
ENSMUSG00000029265	37.265	121.974	1.710680491	1.69594E-61	4.11173E-63
ENSMUSG00000025986	5.198	17.00466667	1.709902187	3.26525E-100	4.2418E-102
ENSMUSG00000030782	0.138666667	0.452666667	1.706828046	0.049924298	0.023580084
ENSMUSG00000022106	1.924	6.275666667	1.705659926	1.76467E-08	3.32707E-09
ENSMUSG00000030701	15.25	49.38766667	1.695341567	2.25433E-49	6.90629E-51
ENSMUSG00000026094	24.447	79.16766667	1.695253894	4.62819E-65	1.05456E-66
ENSMUSG00000029580	919.7383333	2976.486	1.694314732	1.1456E-127	1.1301E-129

ENSMUSG00000028885	1.419666667	4.579	1.689480336	1.47033E-11	2.10719E-12
ENSMUSG00000028339	1.121666667	3.616666667	1.689016633	6.68016E-19	5.9075E-20
ENSMUSG00000050592	4.089666667	13.12533333	1.682298898	2.04093E-43	7.34423E-45
ENSMUSG00000036461	48.46066667	155.364	1.680766093	4.5253E-197	2.5149E-199
ENSMUSG00000024912	0.648	2.075333333	1.679277358	0.006509665	0.002532416
ENSMUSG00000030123	10.47133333	33.44633333	1.675402904	1.4626E-122	1.5648E-124
ENSMUSG00000025477	5.818	18.547	1.672590646	2.47135E-27	1.46101E-28
ENSMUSG00000050912	54.791	174.134	1.66818708	2.3654E-223	1.0352E-225
ENSMUSG00000009687	79.99533333	253.991	1.666789632	2.4014E-102	3.0695E-104
ENSMUSG00000071113	0.202333333	0.639666667	1.66058629	0.048180041	0.022642444
ENSMUSG00000034595	23.21733333	73.39533333	1.660486057	4.0131E-103	5.0181E-105
ENSMUSG00000039959	1.813	5.729333333	1.659988351	1.31862E-27	7.71292E-29
ENSMUSG00000042590	14.07333333	44.44866667	1.659176065	5.84193E-48	1.86682E-49
ENSMUSG00000020255	25.08233333	79.12533333	1.657468108	4.0067E-201	2.1432E-203
ENSMUSG00000020902	1.902	5.994	1.656001838	1.8461E-27	1.08752E-28
ENSMUSG00000037960	19.723	62.04566667	1.653451443	1.33807E-27	7.83598E-29
ENSMUSG00000041836	13.299	41.829	1.65318574	5.40774E-94	7.70119E-96
ENSMUSG00000031555	6.923666667	21.74966667	1.651385116	1.29184E-78	2.35124E-80
ENSMUSG00000032966	56.83533333	178.5223333	1.651244561	7.4851E-126	7.6957E-128
ENSMUSG00000030096	7.984333333	25.079	1.651235966	7.04822E-57	1.8557E-58
ENSMUSG00000015702	2.017666667	6.336666667	1.651036276	9.207E-12	1.2971E-12
ENSMUSG00000049775	583.109	1824.687	1.645811515	1.06564E-89	1.65083E-91
ENSMUSG00000022255	63.24166667	197.7653333	1.644842261	3.7381E-149	3.22E-151
ENSMUSG00000044017	0.063	0.196333333	1.6398814	0.03158015	0.014196259
ENSMUSG00000059248	27.425	84.84933333	1.629411704	6.74849E-62	1.61739E-63
ENSMUSG00000031955	0.512666667	1.578333333	1.622308922	1.14668E-06	2.61437E-07
ENSMUSG00000030579	3.544666667	10.90233333	1.620915065	0.000747555	0.000246311
ENSMUSG00000092232	0.649333333	1.996	1.620080544	0.011435876	0.00467402
ENSMUSG00000032289	1.398333333	4.296333333	1.61939762	4.14919E-32	2.08684E-33
ENSMUSG00000064125	0.152	0.464333333	1.611089528	0.003104977	0.00113824
ENSMUSG00000027939	0.092666667	0.283	1.610679671	0.018161638	0.007747872
ENSMUSG00000035158	3.580666667	10.925	1.609333154	5.3664E-40	2.15103E-41

ENSMUSG00000026988	3.609	11.00266667	1.608182177	1.22158E-13	1.49781E-14
ENSMUSG00000052485	0.376666667	1.146666667	1.606085792	0.039641024	0.018216421
ENSMUSG00000001918	15.48766667	47.12566667	1.60539322	1.36478E-55	3.65963E-57
ENSMUSG00000044469	1.591	4.834333333	1.603383116	4.4193E-13	5.63964E-14
ENSMUSG00000034187	30.95066667	93.89633333	1.601098337	1.02464E-83	1.74391E-85
ENSMUSG00000054342	8.337666667	25.282	1.600394996	3.1517E-32	1.57859E-33
ENSMUSG00000037628	5.006333333	15.177	1.600060388	3.23441E-14	3.79726E-15
ENSMUSG00000025921	3.490333333	10.55466667	1.596444289	1.61515E-18	1.46088E-19
ENSMUSG00000027639	14.391	43.44333333	1.593967958	1.4327E-80	2.528E-82
ENSMUSG00000015714	52.81033333	159.0036667	1.590167882	4.16793E-75	7.99575E-77
ENSMUSG00000037286	22.88433333	68.84066667	1.588900805	2.3525E-114	2.6638E-116
ENSMUSG00000032556	3.492	10.503	1.588676004	1.98504E-09	3.41022E-10
ENSMUSG00000039191	24.63333333	73.97	1.586328412	5.0978E-139	4.6746E-141
ENSMUSG00000044447	4.457666667	13.29333333	1.576342264	2.16825E-38	9.06763E-40
ENSMUSG00000050957	4.062666667	12.10966667	1.575660249	3.09805E-09	5.43393E-10
ENSMUSG00000021608	9.669666667	28.79366667	1.574213454	4.62819E-65	1.0523E-66
ENSMUSG00000025494	3.759666667	11.17533333	1.571641201	5.9603E-23	4.25647E-24
ENSMUSG00000036634	1.681666667	4.995666667	1.570785448	1.88062E-11	2.72262E-12
ENSMUSG00000025006	9.193666667	27.24733333	1.567402777	7.88966E-51	2.31291E-52
ENSMUSG00000003348	10.06566667	29.81666667	1.566676254	1.05055E-63	2.45214E-65
ENSMUSG00000031785	80.31666667	237.7823333	1.56587023	5.00578E-36	2.24991E-37
ENSMUSG00000027935	3.009333333	8.897	1.563875035	1.90397E-10	2.98789E-11
ENSMUSG00000040433	13.75266667	40.591	1.561448496	2.71062E-96	3.69074E-98
ENSMUSG00000032177	5.270333333	15.52133333	1.558286379	1.62907E-51	4.6852E-53
ENSMUSG00000026519	6.161	18.12566667	1.556797618	1.49743E-30	7.85385E-32
ENSMUSG00000041406	16.69833333	48.72866667	1.545066633	7.72796E-91	1.15423E-92
ENSMUSG00000032913	4.3	12.54166667	1.544320516	9.28584E-36	4.21878E-37
ENSMUSG00000030748	2.841333333	8.285666667	1.544049689	7.98495E-22	6.01298E-23
ENSMUSG00000033446	1.229666667	3.573666667	1.539137785	8.6996E-19	7.77797E-20
ENSMUSG00000023088	13.30833333	38.57866667	1.535473376	6.289E-114	7.2087E-116
ENSMUSG00000054021	29.861	86.39433333	1.532674208	2.68135E-61	6.51943E-63
ENSMUSG00000030921	5.798666667	16.69966667	1.526026193	6.56752E-31	3.39896E-32

ENSMUSG00000037446	7.556333333	21.68966667	1.521249631	6.06231E-43	2.22362E-44
ENSMUSG00000027712	25.64533333	73.55566667	1.520140166	4.00861E-32	2.01336E-33
ENSMUSG00000036381	15.27933333	43.78266667	1.51877823	8.91104E-44	3.16947E-45
ENSMUSG00000054951	0.462	1.322666667	1.517484768	0.01135056	0.004635633
ENSMUSG00000029263	4.394	12.577	1.517181061	5.73893E-47	1.87776E-48
ENSMUSG00000021948	21.44	61.34766667	1.516703567	4.43763E-87	7.24448E-89
ENSMUSG00000024371	0.641666667	1.831666667	1.513261035	0.000408798	0.000129412
ENSMUSG00000029406	1.754333333	5.006	1.512735397	5.62054E-24	3.84204E-25
ENSMUSG00000059182	30.185	86.04266667	1.511220437	2.5492E-107	3.099E-109
ENSMUSG00000028480	10.69133333	30.46466667	1.51069517	5.98033E-32	3.01197E-33
ENSMUSG00000058186	1.26	3.589333333	1.510292176	5.29911E-15	5.91571E-16
ENSMUSG00000024968	8.360333333	23.80933333	1.509894855	7.35246E-39	3.05437E-40
ENSMUSG00000063851	0.487666667	1.387666667	1.508693789	0.017775502	0.007563386
ENSMUSG00000031938	5.594	15.91466667	1.508404781	1.03491E-26	6.24755E-28
ENSMUSG00000079484	0.539666667	1.534666667	1.507784848	0.013379975	0.005543464
ENSMUSG00000075054	4.207	11.936	1.504455717	1.08055E-15	1.14623E-16
ENSMUSG00000028088	0.200666667	0.567666667	1.500243043	0.010669985	0.004336937
ENSMUSG00000037922	9.809666667	27.71166667	1.498217462	1.02739E-53	2.8477E-55
ENSMUSG00000022089	13.96166667	39.44	1.498188379	6.71745E-52	1.91327E-53
ENSMUSG00000067212	16.18166667	45.66466667	1.496718093	8.2173E-52	2.34617E-53
ENSMUSG00000041396	4.901	13.81533333	1.49512232	5.85473E-19	5.14907E-20
ENSMUSG00000074793	0.448	1.262666667	1.494903193	0.000572573	0.000185077
ENSMUSG00000046879	9.337666667	26.24966667	1.491165109	1.91937E-43	6.89348E-45
ENSMUSG00000030707	96.33533333	270.042	1.487046865	1.2011E-140	1.0847E-142
ENSMUSG00000064179	3.986333333	11.156	1.484685582	1.28192E-08	2.3884E-09
ENSMUSG00000027634	31.48333333	88.09133333	1.484411794	2.303E-126	2.3518E-128
ENSMUSG00000020178	8.370666667	23.379	1.481798789	1.31924E-31	6.70845E-33
ENSMUSG00000006262	11.118	31.01666667	1.480146362	5.26832E-42	1.98729E-43
ENSMUSG00000025036	1.175666667	3.279666667	1.480070117	2.37614E-07	5.00648E-08
ENSMUSG00000045216	8.578333333	23.906	1.478603474	1.20512E-34	5.64258E-36
ENSMUSG00000044562	0.267	0.742333333	1.47522741	0.003299953	0.001215676
ENSMUSG00000042155	3.959666667	10.99	1.472740495	4.27253E-36	1.91737E-37

ENSMUSG00000031390	0.165	0.457	1.469728141	0.037001641	0.016901082
ENSMUSG00000037752	0.441	1.217666667	1.465268692	3.41117E-05	9.27971E-06
ENSMUSG00000030557	21.46666667	59.267	1.465130845	4.49877E-67	9.75072E-69
ENSMUSG00000054931	0.341333333	0.940666667	1.462502273	0.003007608	0.00109983
ENSMUSG00000074652	0.378	1.041666667	1.462435549	1.18054E-05	3.02207E-06
ENSMUSG00000006362	27.74733333	76.37333333	1.460719864	3.58208E-90	5.44964E-92
ENSMUSG00000022022	2.627333333	7.218666667	1.458133138	1.93604E-09	3.322E-10
ENSMUSG00000005968	2.520666667	6.909333333	1.454741166	1.55599E-09	2.64501E-10
ENSMUSG00000050965	29.07033333	79.62933333	1.453752365	4.96079E-43	1.8127E-44
ENSMUSG00000048897	5.544333333	15.175	1.452610614	5.30861E-38	2.2385E-39
ENSMUSG00000039621	6.094666667	16.67733333	1.4522694	2.10699E-45	7.17228E-47
ENSMUSG00000018293	560.1133333	1531.515	1.45116882	2.20875E-67	4.74126E-69
ENSMUSG00000028656	180.5756667	492.4243333	1.447298558	2.66598E-98	3.5744E-100
ENSMUSG00000030844	7.267666667	19.76866667	1.443651413	2.46236E-15	2.68901E-16
ENSMUSG00000041959	94.67966667	257.407	1.442924755	1.31153E-78	2.3962E-80
ENSMUSG00000028064	3.173666667	8.589	1.436339563	7.33458E-27	4.41756E-28
ENSMUSG00000060063	29.18133333	78.79866667	1.433125415	3.36015E-32	1.68533E-33
ENSMUSG00000058099	6.073	16.36533333	1.430161714	9.98468E-30	5.40331E-31
ENSMUSG00000021951	8.301333333	22.34166667	1.428321832	6.32808E-16	6.58085E-17
ENSMUSG00000051262	0.323	0.868333333	1.426714802	0.04698551	0.022027214
ENSMUSG00000021384	2.614	7.023333333	1.425896767	1.32452E-07	2.71529E-08
ENSMUSG00000091477	0.665333333	1.786	1.424582861	0.004239422	0.001593281
ENSMUSG00000018378	17.05766667	45.76366667	1.423782335	1.56542E-80	2.77306E-82
ENSMUSG00000036986	11.02433333	29.535	1.421734197	2.83068E-78	5.19139E-80
ENSMUSG00000016477	5.522666667	14.79466667	1.421640234	3.88996E-44	1.36466E-45
ENSMUSG00000047921	8.454333333	22.63233333	1.42062243	1.50415E-64	3.44821E-66
ENSMUSG00000041187	9.222	24.686	1.420541517	8.0054E-53	2.23562E-54
ENSMUSG00000048497	13.51766667	36.161	1.41958843	1.41231E-16	1.42261E-17
ENSMUSG00000059429	0.259	0.692	1.41781994	0.000411208	0.000130233
ENSMUSG00000002603	69.94066667	186.7586667	1.416971741	9.68361E-40	3.9017E-41
ENSMUSG00000022309	64.978	172.9983333	1.412734895	3.4412E-132	3.2751E-134
ENSMUSG00000031562	4.347	11.567	1.411922737	1.91988E-12	2.57673E-13

ENSMUSG00000030761	5.507	14.64333333	1.410905485	3.85392E-30	2.05078E-31
ENSMUSG00000019866	2.351333333	6.251	1.410607927	4.4992E-28	2.58169E-29
ENSMUSG00000029478	18.38233333	48.75466667	1.407220412	1.45932E-81	2.54456E-83
ENSMUSG00000039953	3.33	8.806666667	1.403073883	2.08553E-25	1.32346E-26
ENSMUSG00000020534	16.293	42.898	1.396658119	1.49669E-56	3.97175E-58
ENSMUSG00000038770	0.462333333	1.217	1.396323881	0.002483261	0.000895446
ENSMUSG00000000223	0.087333333	0.228666667	1.388641765	0.049678899	0.02344002
ENSMUSG00000064247	0.222666667	0.583	1.388610281	0.033270445	0.015041616
ENSMUSG00000026879	10.74766667	28.11966667	1.38755601	3.72312E-21	2.90711E-22
ENSMUSG00000023036	1.289666667	3.368666667	1.385179451	0.000362007	0.000113669
ENSMUSG00000023259	2.133	5.548666667	1.379257171	5.64699E-12	7.834E-13
ENSMUSG00000001366	21.58766667	56.117	1.378230569	6.88364E-55	1.86975E-56
ENSMUSG00000052087	4.866333333	12.62166667	1.374995378	2.47957E-19	2.12215E-20
ENSMUSG00000029471	9.671666667	25.07666667	1.374509161	2.73019E-45	9.33141E-47
ENSMUSG00000004451	23.49633333	60.86233333	1.373114007	5.26508E-62	1.25455E-63
ENSMUSG00000030830	25.23466667	65.31666667	1.372044137	8.65995E-75	1.68446E-76
ENSMUSG00000039232	5.586333333	14.44233333	1.37033028	5.11959E-42	1.92762E-43
ENSMUSG00000021591	27.15833333	70.065	1.367299	1.1952E-24	7.92097E-26
ENSMUSG00000047986	0.313333333	0.808333333	1.367252085	0.033413833	0.015113405
ENSMUSG00000032038	18.83266667	48.56233333	1.366600445	1.66877E-31	8.52063E-33
ENSMUSG00000037722	9.867	25.43966667	1.366396353	1.45433E-32	7.18325E-34
ENSMUSG00000022051	25.343	65.12366667	1.361594615	8.98064E-37	3.96159E-38
ENSMUSG00000036743	6.799	17.467	1.361237368	7.94763E-18	7.45901E-19
ENSMUSG00000032902	14.43066667	37.06633333	1.360971457	2.05638E-41	7.88551E-43
ENSMUSG00000031482	2.175	5.577333333	1.358560096	1.87058E-15	2.02197E-16
ENSMUSG00000030341	6.151666667	15.71966667	1.353521389	8.96569E-25	5.91069E-26
ENSMUSG00000022307	27.21533333	69.31366667	1.348720132	1.45937E-37	6.29571E-39
ENSMUSG00000074825	10.82433333	27.45033333	1.342545496	1.17105E-37	5.02752E-39
ENSMUSG00000031445	0.413666667	1.048333333	1.341556902	0.001072122	0.000362861
ENSMUSG00000040139	1.845	4.671	1.340110629	0.000302425	9.37213E-05
ENSMUSG00000067370	0.554666667	1.402	1.337793417	0.018349651	0.007840827
ENSMUSG00000029162	16.31633333	41.23933333	1.337704125	1.59955E-35	7.30046E-37

ENSMUSG00000024965	88.567	222.7053333	1.330294951	3.41103E-96	4.6681E-98
ENSMUSG00000024589	15.32166667	38.48066667	1.328560554	4.62031E-43	1.68186E-44
ENSMUSG00000052632	1.203333333	3.016666667	1.325918955	1.75216E-11	2.52691E-12
ENSMUSG00000071470	3.130333333	7.841333333	1.324782699	9.05315E-06	2.28168E-06
ENSMUSG00000071669	0.895666667	2.240666667	1.322894221	0.000536232	0.000172822
ENSMUSG00000047959	7.230666667	18.08333333	1.322460062	2.18362E-08	4.13819E-09
ENSMUSG00000032741	11.39333333	28.467	1.321100566	1.85883E-33	8.94785E-35
ENSMUSG00000048827	0.951333333	2.376333333	1.320714386	6.02079E-12	8.36511E-13
ENSMUSG00000019102	0.956333333	2.387	1.319613099	0.001081195	0.000366232
ENSMUSG00000018377	33.41066667	83.21266667	1.316494383	9.57775E-84	1.61681E-85
ENSMUSG00000026509	31.67566667	78.765	1.314179713	8.3479E-45	2.88799E-46
ENSMUSG00000060224	4.480666667	11.128	1.312409017	1.22027E-20	9.7486E-22
ENSMUSG00000021697	14.47966667	35.94	1.311562018	2.67312E-50	7.92931E-52
ENSMUSG00000044080	33.03533333	81.87433333	1.309401352	2.87641E-34	1.35678E-35
ENSMUSG00000022075	1.823333333	4.504	1.304628495	7.77322E-19	6.92813E-20
ENSMUSG00000028086	4.566333333	11.251	1.300945153	2.36018E-24	1.57892E-25
ENSMUSG00000032508	14.13766667	34.826	1.300620747	6.38889E-34	3.02246E-35
ENSMUSG00000045576	6.223333333	15.31766667	1.299437122	1.41682E-20	1.13779E-21
ENSMUSG00000042105	0.805	1.978666667	1.297467903	0.000185972	5.60176E-05
ENSMUSG00000016496	12.60766667	30.94066667	1.295202985	6.97235E-29	3.91847E-30
ENSMUSG00000041911	3.783666667	9.285	1.295116914	4.04383E-14	4.7672E-15
ENSMUSG00000063382	3.473666667	8.492333333	1.28970168	3.68381E-27	2.19058E-28
ENSMUSG00000060791	5.659333333	13.82166667	1.288227572	0.000175852	5.27617E-05
ENSMUSG00000032047	27.43066667	66.81466667	1.284375142	1.66041E-95	2.28386E-97
ENSMUSG00000017776	16.80033333	40.909	1.283928414	3.54086E-52	9.98671E-54
ENSMUSG00000026447	1.666	4.046333333	1.280226772	3.14624E-26	1.94304E-27
ENSMUSG00000019916	16.79233333	40.66066667	1.27583116	3.26801E-23	2.30657E-24
ENSMUSG00000021930	3.678	8.905666667	1.275802134	1.72393E-13	2.13291E-14
ENSMUSG00000062591	0.589	1.425666667	1.275297167	0.003228809	0.001188121
ENSMUSG00000026854	9.345333333	22.51666667	1.26867524	3.0508E-30	1.61918E-31
ENSMUSG00000023087	422.473	1017.296	1.26780847	9.74689E-99	1.30004E-100
ENSMUSG00000040272	2.869	6.908333333	1.267789728	7.61858E-08	1.52477E-08

ENSMUSG00000034930	14.02466667	33.76133333	1.2674054	1.10731E-35	5.03847E-37
ENSMUSG00000029570	2.343333333	5.620666667	1.26217907	5.1325E-10	8.35391E-11
ENSMUSG00000028035	8.556666667	20.44433333	1.256580224	6.88079E-21	5.43485E-22
ENSMUSG00000002058	17.62233333	42.10433333	1.256563761	2.34018E-31	1.19813E-32
ENSMUSG00000034765	3.629	8.668333333	1.256182575	1.51949E-06	3.51925E-07
ENSMUSG00000032462	16.82566667	40.058	1.251426724	7.88492E-59	2.00478E-60
ENSMUSG00000002797	6.148666667	14.61133333	1.248742333	1.97662E-10	3.1074E-11
ENSMUSG00000004530	23.51733333	55.79366667	1.246376886	7.64945E-42	2.89611E-43
ENSMUSG00000037818	3.776333333	8.958333333	1.246244233	3.16535E-15	3.4831E-16
ENSMUSG00000042751	0.273333333	0.647666667	1.244590086	0.005298695	0.002023774
ENSMUSG00000068744	9.395	22.25266667	1.244013166	2.60493E-23	1.82409E-24
ENSMUSG00000001750	9.233	21.84433333	1.242387685	8.91659E-28	5.16599E-29
ENSMUSG00000002107	25.456	60.01533333	1.237325402	5.93134E-95	8.28203E-97
ENSMUSG00000052837	9.869333333	23.259	1.236764531	4.70957E-15	5.2314E-16
ENSMUSG00000044712	1204.300667	2837.766333	1.236560179	7.02275E-99	9.3181E-101
ENSMUSG00000001741	5.174333333	12.16033333	1.232737874	1.59815E-20	1.29006E-21
ENSMUSG00000000561	18.263	42.867	1.230943684	1.24135E-26	7.52829E-28
ENSMUSG00000032101	1.447	3.394666667	1.230204997	1.25756E-10	1.94902E-11
ENSMUSG00000041895	6.965	16.33233333	1.22953566	2.52028E-21	1.94164E-22
ENSMUSG00000033386	23.45266667	54.93266667	1.227912356	1.31487E-43	4.695E-45
ENSMUSG00000027637	52.24466667	122.332	1.227446164	3.35918E-60	8.28419E-62
ENSMUSG00000020570	45.61366667	106.753	1.226738562	6.91368E-83	1.1863E-84
ENSMUSG00000014226	27.519	64.27133333	1.223747356	1.82448E-45	6.18512E-47
ENSMUSG00000047766	3.87	9.033666667	1.222978114	1.55863E-15	1.67719E-16
ENSMUSG00000038375	1.992333333	4.642666667	1.22049466	5.72276E-07	1.26302E-07
ENSMUSG00000020901	1.586666667	3.694333333	1.219314971	3.56448E-11	5.27676E-12
ENSMUSG00000028212	6.216	14.46866667	1.21887357	5.921E-24	4.05565E-25
ENSMUSG00000038543	3.957666667	9.204	1.217610877	0.001222358	0.00041736
ENSMUSG00000028613	6.825666667	15.86166667	1.216502504	1.23001E-15	1.31247E-16
ENSMUSG00000041607	9.791666667	22.646	1.209629896	7.71459E-29	4.3624E-30
ENSMUSG00000027763	143.0216667	330.372	1.207857699	2.50039E-82	4.3251E-84
ENSMUSG00000027286	5.409	12.49333333	1.20772465	1.5965E-12	2.13605E-13

ENSMUSG00000021929	18.173	41.92666667	1.206071535	2.64926E-69	5.4476E-71
ENSMUSG00000046711	69.69233333	160.6903333	1.20521128	1.78587E-33	8.57266E-35
ENSMUSG00000038028	2.39	5.509	1.204779845	2.16366E-06	5.09537E-07
ENSMUSG00000024456	35.05433333	80.72733333	1.203464438	1.19313E-88	1.88149E-90
ENSMUSG00000114133	5.476666667	12.61066667	1.203274567	7.52995E-18	7.06178E-19
ENSMUSG00000005610	257.5393333	592.7973333	1.202746169	2.2128E-72	4.47326E-74
ENSMUSG00000004667	34.665	79.75433333	1.202083147	2.2829E-28	1.30202E-29
ENSMUSG00000027201	22.49	51.59066667	1.197826428	5.7034E-41	2.23065E-42
ENSMUSG00000034685	2.151333333	4.931333333	1.196746698	1.44935E-07	2.97825E-08
ENSMUSG00000038843	19.47366667	44.62833333	1.196435377	1.29704E-35	5.91081E-37
ENSMUSG00000038295	0.547666667	1.253	1.194016435	0.000159439	4.75936E-05
ENSMUSG00000023031	3.055	6.988	1.193707228	5.37769E-06	1.3221E-06
ENSMUSG00000028568	15.03233333	34.38233333	1.193598492	6.40757E-23	4.59369E-24
ENSMUSG00000026812	5.418333333	12.38	1.192090259	9.36077E-29	5.30628E-30
ENSMUSG00000047180	5.868	13.375	1.188598115	2.45434E-13	3.06729E-14
ENSMUSG00000092165	3.062666667	6.977666667	1.187956324	3.60954E-06	8.70101E-07
ENSMUSG00000005107	3.352666667	7.626333333	1.185680537	1.39217E-15	1.49033E-16
ENSMUSG00000042688	13.75466667	31.27433333	1.185057954	2.47519E-37	1.07639E-38
ENSMUSG00000031467	8.488666667	19.29333333	1.184492552	7.29945E-29	4.11751E-30
ENSMUSG00000054115	10.435	23.68533333	1.182563375	7.25189E-37	3.18388E-38
ENSMUSG00000023905	2.475333333	5.616666667	1.182091371	0.000205448	6.21553E-05
ENSMUSG00000078566	38.69966667	87.523	1.17734105	0.003375375	0.001245336
ENSMUSG00000008601	0.642666667	1.452	1.175898902	0.048986333	0.023055395
ENSMUSG00000035047	11.67533333	26.36333333	1.175069054	4.53949E-33	2.21692E-34
ENSMUSG00000014301	3.363666667	7.579666667	1.17209966	0.001983748	0.000704337
ENSMUSG00000115018	2.081333333	4.684666667	1.170438361	0.004652187	0.001760689
ENSMUSG00000017561	30.343	68.26633333	1.169810534	2.47095E-48	7.82278E-50
ENSMUSG00000026970	21.827	49.06933333	1.168707817	8.09229E-66	1.81015E-67
ENSMUSG00000052310	17.70033333	39.77833333	1.168206301	6.86657E-35	3.20074E-36
ENSMUSG00000029171	20.79466667	46.69866667	1.1671678	3.47897E-44	1.21806E-45
ENSMUSG00000023805	3.059	6.869	1.167039978	4.74872E-22	3.54298E-23
ENSMUSG00000046546	0.640666667	1.436	1.164409914	0.001112311	0.000377545

ENSMUSG00000024238	9.364666667	20.97933333	1.163669286	4.27432E-27	2.55064E-28
ENSMUSG00000005057	1.504	3.368	1.163087571	6.68496E-05	1.89659E-05
ENSMUSG00000030663	56.596	126.6863333	1.162488901	1.53464E-61	3.71E-63
ENSMUSG00000053192	3.281333333	7.334	1.160318109	1.14044E-06	2.59778E-07
ENSMUSG00000078249	167.697	374.4286667	1.158834013	9.34429E-51	2.74584E-52
ENSMUSG00000025010	1.754333333	3.916666667	1.158703457	2.09708E-09	3.60998E-10
ENSMUSG00000033623	2.789	6.218	1.156702682	3.61718E-23	2.55552E-24
ENSMUSG00000098923	14.051	31.29766667	1.155382294	7.08477E-27	4.25726E-28
ENSMUSG00000023277	21.54766667	47.994	1.155322405	1.56123E-32	7.7221E-34
ENSMUSG00000027210	2.275666667	5.061	1.153133223	5.40245E-07	1.18933E-07
ENSMUSG00000029762	10.16533333	22.59433333	1.152303466	1.45446E-14	1.66715E-15
ENSMUSG00000032340	7.819666667	17.37566667	1.151889317	2.58918E-36	1.15654E-37
ENSMUSG00000028410	72.537	161.109	1.151248105	5.61249E-36	2.5265E-37
ENSMUSG00000036249	12.21566667	27.118	1.150518181	1.10728E-29	6.00755E-31
ENSMUSG00000048537	3.17	7.035	1.150067583	2.81959E-15	3.09087E-16
ENSMUSG00000030102	18.80033333	41.70766667	1.149554362	2.7436E-41	1.0578E-42
ENSMUSG00000008450	10.91533333	24.21433333	1.149505095	5.70111E-06	1.40557E-06
ENSMUSG00000053158	12.408	27.51433333	1.148912781	2.5982E-24	1.73996E-25
ENSMUSG00000026979	32.50633333	72.04733333	1.148224202	4.95114E-72	1.00433E-73
ENSMUSG00000074480	1.160333333	2.571333333	1.147977332	5.73055E-11	8.65853E-12
ENSMUSG00000025608	4.259333333	9.428666667	1.146426131	2.08553E-25	1.32419E-26
ENSMUSG00000034006	8.181333333	18.109	1.146298994	4.77013E-14	5.67312E-15
ENSMUSG00000030879	23.255	51.467	1.146106751	1.56622E-12	2.09228E-13
ENSMUSG00000051444	3.762666667	8.312333333	1.143498022	3.45001E-13	4.36913E-14
ENSMUSG00000051498	1.08	2.385333333	1.143159574	0.001835558	0.000647641
ENSMUSG00000025574	32.585	71.86633333	1.141108086	2.97013E-45	1.01721E-46
ENSMUSG00000027953	13.75866667	30.329	1.140357263	1.53074E-12	2.04168E-13
ENSMUSG00000007036	23.06466667	50.80533333	1.139295511	5.72913E-24	3.92025E-25
ENSMUSG00000020142	6.032666667	13.26566667	1.136829406	1.48329E-19	1.25402E-20
ENSMUSG00000028645	17.25466667	37.83766667	1.132836521	2.39703E-27	1.41541E-28
ENSMUSG00000021549	11.41433333	24.98633333	1.130292606	1.21807E-39	4.93319E-41
ENSMUSG00000055116	2.738666667	5.991333333	1.12940342	1.40387E-08	2.62731E-09

ENSMUSG00000061186	8.143666667	17.791	1.127397188	4.52104E-46	1.50754E-47
ENSMUSG00000027963	4.780666667	10.43966667	1.126791926	3.37677E-18	3.08472E-19
ENSMUSG00000048922	15.27733333	33.36066667	1.126755377	3.20996E-44	1.12165E-45
ENSMUSG00000003526	12.28133333	26.78866667	1.12515558	1.83037E-27	1.07699E-28
ENSMUSG00000027489	0.554333333	1.209	1.124988577	0.046468909	0.021760814
ENSMUSG00000046668	11.883	25.82866667	1.120074063	7.48782E-24	5.14446E-25
ENSMUSG00000027669	0.727666667	1.581333333	1.11979188	0.000383538	0.000120803
ENSMUSG00000091512	25.182	54.63733333	1.117494203	4.19042E-30	2.23276E-31
ENSMUSG00000022610	13.289	28.78466667	1.11506796	3.63844E-29	2.01447E-30
ENSMUSG00000056737	56.963	123.345	1.114602201	0.000269666	8.31385E-05
ENSMUSG00000022299	5.795	12.548	1.114576868	1.28325E-12	1.70179E-13
ENSMUSG00000034771	0.877666667	1.900333333	1.11450748	0.028725701	0.012759439
ENSMUSG00000035678	5.771666667	12.46566667	1.110900154	7.7553E-09	1.41206E-09
ENSMUSG00000045038	4.413666667	9.53	1.110498537	3.81997E-18	3.51878E-19
ENSMUSG00000029655	8.211333333	17.703	1.108305457	0.000342283	0.000107072
ENSMUSG00000020014	1.393	3.002333333	1.107888903	1.24669E-13	1.53032E-14
ENSMUSG00000040549	43.52366667	93.67566667	1.105874238	1.53043E-58	3.90183E-60
ENSMUSG00000027130	12.98666667	27.93866667	1.105231995	1.95947E-32	9.7463E-34
ENSMUSG00000054027	3.596666667	7.732333333	1.104243373	5.88818E-14	7.04782E-15
ENSMUSG00000054814	4.108666667	8.814333333	1.101181166	1.64317E-13	2.02956E-14
ENSMUSG00000040296	7.713	16.543	1.100856869	3.32293E-17	3.23406E-18
ENSMUSG00000038267	4.802	10.283	1.098553914	4.69962E-31	2.41919E-32
ENSMUSG00000030403	43.354	92.797	1.097913058	3.94719E-25	2.55834E-26
ENSMUSG00000034101	16.18866667	34.63966667	1.09744088	4.00067E-50	1.19228E-51
ENSMUSG00000053477	9.644666667	20.60833333	1.095424551	5.31585E-24	3.62637E-25
ENSMUSG00000024943	48.161	102.741	1.093074768	4.25279E-90	6.52912E-92
ENSMUSG00000027327	26.605	56.74966667	1.092914515	1.271E-32	6.26007E-34
ENSMUSG00000079197	20.83966667	44.44833333	1.092797122	3.00944E-23	2.1157E-24
ENSMUSG00000027995	3.777	8.053333333	1.092345265	6.82661E-13	8.84021E-14
ENSMUSG00000030691	2.473666667	5.274333333	1.092337645	4.65054E-13	5.94765E-14
ENSMUSG00000024691	94.274	200.9873333	1.092172735	6.72337E-86	1.12095E-87
ENSMUSG00000044350	2.721333333	5.801	1.091987938	7.42223E-07	1.65975E-07

ENSMUSG00000025260	44.87466667	95.60166667	1.091134547	3.10456E-30	1.64987E-31
ENSMUSG00000030946	9.052	19.23833333	1.08767533	1.37596E-10	2.13921E-11
ENSMUSG00000037572	12.81033333	27.196	1.086086459	1.30531E-47	4.21652E-49
ENSMUSG00000022419	2.513666667	5.332333333	1.08497362	1.2084E-18	1.08542E-19
ENSMUSG00000027950	0.814	1.724333333	1.082937991	0.000426141	0.000135258
ENSMUSG00000032294	551.411	1167.120333	1.081753362	1.14832E-39	4.64275E-41
ENSMUSG00000004771	45.85866667	96.805	1.077887156	4.64811E-42	1.74365E-43
ENSMUSG00000019464	0.554	1.168333333	1.076494062	0.000964098	0.000323755
ENSMUSG00000002881	14.055	29.54433333	1.071797991	1.01591E-43	3.62045E-45
ENSMUSG00000040841	1.890333333	3.973333333	1.071709174	5.64645E-05	1.58174E-05
ENSMUSG00000032624	13.36166667	28.07366667	1.071117531	2.08063E-50	6.15734E-52
ENSMUSG00000018008	15.79933333	33.193	1.071015343	8.97862E-29	5.08341E-30
ENSMUSG00000063275	6.433	13.506	1.070036869	4.19161E-07	9.10244E-08
ENSMUSG00000023262	9.018666667	18.924	1.069231002	2.91033E-15	3.19641E-16
ENSMUSG00000080717	8.784	18.424	1.068636357	0.014907976	0.006244883
ENSMUSG00000028793	7.849	16.45533333	1.067974487	4.3655E-08	8.53388E-09
ENSMUSG00000062995	27.66533333	57.99333333	1.067807757	7.39822E-43	2.71876E-44
ENSMUSG00000027708	20.67566667	43.321	1.067132697	2.40346E-29	1.32403E-30
ENSMUSG00000032412	44.03866667	92.257	1.06688759	4.60819E-64	1.06922E-65
ENSMUSG00000116564	11.01333333	23.07133333	1.066850196	3.66762E-18	3.3657E-19
ENSMUSG00000078485	0.974666667	2.039333333	1.065116796	0.022396052	0.009756557
ENSMUSG00000061665	14.07133333	29.43633333	1.064838939	6.31509E-58	1.64512E-59
ENSMUSG00000027981	9.584666667	20.05033333	1.064826057	6.06694E-18	5.65602E-19
ENSMUSG00000022895	11.98233333	25.053	1.064074498	7.49554E-37	3.29606E-38
ENSMUSG00000002983	1.641666667	3.431333333	1.063608058	8.42101E-05	2.4213E-05
ENSMUSG00000026737	27.31666667	57.08166667	1.063246011	4.11365E-51	1.20023E-52
ENSMUSG00000014873	9.244666667	19.31566667	1.063078265	6.75386E-14	8.10275E-15
ENSMUSG00000044788	0.547	1.142666667	1.062791871	0.000153517	4.57191E-05
ENSMUSG00000004446	21.96066667	45.856	1.062188663	1.70403E-37	7.37488E-39
ENSMUSG00000007041	159.422	332.5943333	1.060912856	6.30332E-76	1.18666E-77
ENSMUSG00000000631	12.53266667	26.143	1.060731285	9.38026E-28	5.44173E-29
ENSMUSG00000029005	1.132666667	2.361666667	1.060082001	2.04103E-07	4.26498E-08

ENSMUSG00000035049	13.13	27.36433333	1.059429793	2.94089E-20	2.40665E-21
ENSMUSG00000030309	2.921	6.087666667	1.059427007	9.75777E-11	1.50349E-11
ENSMUSG00000032456	2.582333333	5.380333333	1.059020317	4.94181E-07	1.07899E-07
ENSMUSG00000028175	16.96366667	35.33866667	1.058799568	3.17984E-35	1.46014E-36
ENSMUSG00000025421	12.239	25.47233333	1.057445434	6.99459E-24	4.79587E-25
ENSMUSG00000057706	3.501666667	7.278666667	1.05563244	1.13034E-12	1.48802E-13
ENSMUSG00000007613	13.90666667	28.878	1.054194172	8.8411E-44	3.13845E-45
ENSMUSG00000037725	15.92633333	33.06066667	1.053701659	3.4493E-29	1.90736E-30
ENSMUSG00000079442	3.264	6.771666667	1.052869901	5.25018E-10	8.55274E-11
ENSMUSG00000021733	5.787333333	11.99833333	1.051863371	2.53312E-36	1.12974E-37
ENSMUSG00000002825	6.978	14.46266667	1.051448082	1.54521E-09	2.62348E-10
ENSMUSG00000001228	73.20133333	151.616	1.050480177	1.5772E-60	3.87862E-62
ENSMUSG00000010406	13.315	27.56966667	1.050029396	1.04123E-06	2.35805E-07
ENSMUSG00000045140	3.273	6.775666667	1.0497493	1.20019E-07	2.44956E-08
ENSMUSG00000026815	30.53833333	63.052	1.045920806	1.18318E-36	5.24397E-38
ENSMUSG00000027387	1.795	3.705666667	1.045749269	0.000202294	6.1145E-05
ENSMUSG00000029790	5.606	11.56866667	1.045178948	5.02179E-10	8.16672E-11
ENSMUSG00000022799	0.382	0.788	1.044622991	0.000255646	7.85141E-05
ENSMUSG00000062825	1370.614667	2825.492333	1.043679244	6.2595E-67	1.36974E-68
ENSMUSG00000057130	4.994	10.29166667	1.04320891	1.21884E-15	1.29885E-16
ENSMUSG00000024423	7.271333333	14.98233333	1.042970487	1.57589E-14	1.80962E-15
ENSMUSG00000038235	6.818666667	14.044	1.042392336	7.08915E-19	6.28663E-20
ENSMUSG00000068874	8.222666667	16.92633333	1.041591233	3.55951E-12	4.86388E-13
ENSMUSG00000026843	18.51333333	38.09266667	1.04094861	1.39705E-31	7.12357E-33
ENSMUSG00000032000	7.756	15.93266667	1.038603044	7.56414E-16	7.90518E-17
ENSMUSG00000034659	17.23633333	35.399	1.038255702	3.54648E-13	4.49623E-14
ENSMUSG00000020644	1.871666667	3.839333333	1.036532299	0.036111422	0.016453964
ENSMUSG00000037434	4.381	8.985333333	1.03631181	2.27209E-15	2.47355E-16
ENSMUSG00000043733	17.89333333	36.695	1.036161326	6.13082E-19	5.4004E-20
ENSMUSG00000006301	2.156666667	4.422333333	1.036004561	1.03939E-07	2.10767E-08
ENSMUSG00000066007	0.577666667	1.183666667	1.034953706	0.002398183	0.000863479
ENSMUSG00000046417	2.308333333	4.725	1.033462759	1.65022E-05	4.28061E-06

ENSMUSG00000027469	55.49433333	113.5513333	1.03293228	2.07377E-68	4.32185E-70
ENSMUSG00000068264	2.178666667	4.455666667	1.032195824	0.002020264	0.000718706
ENSMUSG00000005103	81.34133333	166.293	1.031666896	1.18053E-91	1.7304E-93
ENSMUSG00000053799	19.53333333	39.89166667	1.030149236	2.64752E-48	8.42352E-50
ENSMUSG00000017740	0.424333333	0.866	1.029169012	0.001144612	0.000389462
ENSMUSG00000000552	11.69066667	23.85033333	1.028652227	1.37873E-20	1.10529E-21
ENSMUSG00000028069	14.16566667	28.88433333	1.027888697	3.48745E-15	3.84721E-16
ENSMUSG00000021958	4.262	8.685	1.026995256	4.49236E-06	1.09539E-06
ENSMUSG00000074698	34.54933333	70.35066667	1.025906222	3.1978E-51	9.28574E-53
ENSMUSG00000049076	25.10366667	51.11166667	1.025754535	7.83634E-72	1.59503E-73
ENSMUSG00000047557	12.78866667	26.02466667	1.025013827	5.93557E-13	7.65294E-14
ENSMUSG00000025809	78.72933333	160.0776667	1.023798877	1.97279E-55	5.31742E-57
ENSMUSG00000048100	6.076	12.34233333	1.022421389	1.04776E-16	1.04885E-17
ENSMUSG00000021969	5.741666667	11.663	1.022397449	6.55419E-27	3.93388E-28
ENSMUSG00000041975	7.279333333	14.76966667	1.020759032	2.93078E-13	3.69121E-14
ENSMUSG00000061356	0.857333333	1.739	1.020329791	0.007814476	0.003089961
ENSMUSG00000031142	1.843333333	3.732333333	1.017760859	1.99092E-09	3.42307E-10
ENSMUSG00000033705	0.396333333	0.802333333	1.017487427	3.73064E-05	1.02006E-05
ENSMUSG00000074865	3.495666667	7.07	1.016142593	0.000262208	8.06205E-05
ENSMUSG00000022216	39.86266667	80.62033333	1.016105524	3.89362E-37	1.70135E-38
ENSMUSG00000056941	14.23633333	28.78566667	1.015773007	8.00044E-24	5.50777E-25
ENSMUSG00000074671	8.198	16.55833333	1.014213572	4.86675E-24	3.3031E-25
ENSMUSG00000060470	7.408333333	14.96166667	1.014049976	4.50729E-17	4.41179E-18
ENSMUSG00000021996	67.242	135.7126667	1.013120839	8.90371E-36	4.03899E-37
ENSMUSG00000038623	31.60966667	63.76466667	1.012391396	4.89372E-15	5.44955E-16
ENSMUSG00000033847	0.658666667	1.328	1.0116347	0.00236988	0.000852301
ENSMUSG00000032109	3.164	6.373666667	1.01037397	2.57664E-11	3.77323E-12
ENSMUSG00000042354	28.86866667	58.10966667	1.009273702	6.68831E-28	3.84712E-29
ENSMUSG00000043251	6.453	12.97466667	1.007655544	5.50996E-13	7.08506E-14
ENSMUSG00000115483	16.269	32.65066667	1.004986873	1.58825E-21	1.21367E-22
ENSMUSG00000113255	1.532	3.073666667	1.004544419	5.0331E-06	1.23319E-06
ENSMUSG00000096740	4.095	8.215	1.004397123	2.23665E-10	3.53793E-11

ENSMUSG00000042569	6.557333333	13.15066667	1.003954799	2.70407E-10	4.30172E-11
ENSMUSG00000027405	116.0983333	232.8246667	1.003896651	4.20948E-46	1.3978E-47
ENSMUSG00000033967	1.000666667	2.006666667	1.00383951	0.000238026	7.26561E-05
ENSMUSG00000034235	1.278	2.558	1.001128428	0.000485365	0.00015527
ENSMUSG00000020474	3.298	6.6	1.000874626	2.18569E-09	3.76859E-10
