# Supplementary material for: Mutations of RAS genes identified in acute myeloid leukemia affect glycerophospholipid metabolism pathway
Source: Front Oncol. 2023 Nov 14;13:1280192. doi: 10.3389/fonc.2023.1280192 (PMC10682766; doi:10.3389/fonc.2023.1280192)
Supplement: Supplementary file 2 [file DataSheet_2.pdf]

The down-regulated genes of KRAS（G12V）cell line

ENSEMBL	BaF3	BaF3 KRAS(G12V)	log2fc	FDR	Pvalue
ENSMUSG00000055917	10.64733333	5.322	-1.000451732	1.61535E-06	3.75138E-07
ENSMUSG00000046881	3.462	1.729666667	-1.00111169	1.55204E-09	2.63615E-10
ENSMUSG00000051335	6.763666667	3.377	-1.002063382	0.000441045	0.000140356
ENSMUSG00000022861	9.756	4.868	-1.00296059	3.10646E-10	4.96774E-11
ENSMUSG00000038181	13.275	6.621666667	-1.003445568	1.04977E-12	1.37976E-13
ENSMUSG00000030101	20.83166667	10.39	-1.003582615	1.26459E-16	1.26766E-17
ENSMUSG00000025981	14.83433333	7.384666667	-1.006335386	3.32746E-06	7.97713E-07
ENSMUSG00000036534	12.89333333	6.416666667	-1.006729349	1.09586E-11	1.55148E-12
ENSMUSG00000029428	13.34266667	6.64	-1.006791886	6.93652E-11	1.05337E-11
ENSMUSG00000015542	7.683666667	3.821666667	-1.007592984	0.000420157	0.000133183
ENSMUSG00000021366	11.55833333	5.748	-1.007801415	1.10971E-33	5.28065E-35
ENSMUSG00000027931	3.125	1.553666667	-1.008179178	0.000598384	0.000194002
ENSMUSG00000025195	14.08666667	7.002333333	-1.008422621	4.6582E-19	4.0741E-20
ENSMUSG00000060147	9.944333333	4.942333333	-1.00868234	3.01141E-05	8.12108E-06
ENSMUSG00000032855	6.471666667	3.216333333	-1.00872037	0.007381553	0.00290647
ENSMUSG00000064120	12.324	6.123666667	-1.009002929	3.93862E-10	6.35597E-11
ENSMUSG00000021182	22.42866667	11.14433333	-1.009033543	1.47507E-34	6.92706E-36
ENSMUSG00000037410	15.43733333	7.668	-1.009501319	9.85543E-23	7.11344E-24
ENSMUSG00000021785	30.01233333	14.897	-1.010533661	1.40083E-21	1.06558E-22
ENSMUSG00000024268	1.823333333	0.905	-1.010588635	0.000584795	0.000189271
ENSMUSG00000020605	18.27333333	9.069666667	-1.010618393	8.92263E-16	9.37201E-17
ENSMUSG00000058503	38.12766667	18.92033333	-1.010900738	2.07189E-20	1.68256E-21
ENSMUSG00000027438	1.751333333	0.869	-1.011025618	0.001611996	0.000562939
ENSMUSG00000034243	28.843	14.29433333	-1.012777893	3.78919E-45	1.30299E-46
ENSMUSG00000051355	29.80466667	14.76666667	-1.01319404	9.21327E-07	2.07563E-07
ENSMUSG00000052423	21.148	10.47766667	-1.013203761	5.46235E-10	8.91735E-11
ENSMUSG00000022442	16.218	8.031666667	-1.013824617	6.91181E-10	1.14084E-10
ENSMUSG00000024781	29.83833333	14.76966667	-1.014529687	2.88525E-08	5.51394E-09
ENSMUSG00000024831	13.167	6.514666667	-1.015163408	3.6096E-12	4.93985E-13
ENSMUSG00000057101	15.35066667	7.592333333	-1.015686073	6.08717E-10	9.98388E-11

ENSMUSG00000057265	1.520666667	0.751666667	-1.016539013	0.018115839	0.007725817
ENSMUSG00000002393	7.595333333	3.754333333	-1.016556534	1.1051E-06	2.51422E-07
ENSMUSG00000017747	5.152333333	2.546666667	-1.016615795	0.013273846	0.005493961
ENSMUSG00000005682	9.424333333	4.656333333	-1.017196227	4.94805E-11	7.41777E-12
ENSMUSG00000020717	87.28433333	43.07933333	-1.018726804	2.85531E-24	1.91412E-25
ENSMUSG00000042684	17.15833333	8.464	-1.019497892	7.71212E-12	1.08329E-12
ENSMUSG00000000532	13.039	6.431333333	-1.019643458	2.45881E-14	2.85766E-15
ENSMUSG00000042207	30.11066667	14.848	-1.020006035	6.35249E-13	8.21256E-14
ENSMUSG00000023055	33.13066667	16.337	-1.020024152	1.12168E-19	9.45966E-21
ENSMUSG00000021024	191.839	94.42766667	-1.022614517	5.88504E-41	2.30986E-42
ENSMUSG00000062190	14.40533333	7.086333333	-1.023491809	6.54248E-08	1.30077E-08
ENSMUSG00000034744	28.18133333	13.85	-1.024853895	3.85232E-14	4.5334E-15
ENSMUSG00000021258	32.49033333	15.94933333	-1.026514423	5.43109E-19	4.76518E-20
ENSMUSG00000028687	7.228333333	3.547666667	-1.026792575	0.002898969	0.001058088
ENSMUSG00000003123	6.111333333	2.998666667	-1.027166012	3.45469E-08	6.6622E-09
ENSMUSG00000085793	11.35633333	5.571	-1.02748888	1.34568E-10	2.08933E-11
ENSMUSG00000071647	54.29766667	26.60933333	-1.028957837	1.90213E-32	9.44789E-34
ENSMUSG00000053334	2.878333333	1.410333333	-1.029197492	0.000345025	0.000108025
ENSMUSG00000020752	14.09933333	6.907	-1.02949582	9.61582E-16	1.01602E-16
ENSMUSG00000005949	16.845	8.247	-1.030379116	2.01036E-09	3.4579E-10
ENSMUSG00000023027	99.29433333	48.592	-1.030992573	5.09867E-27	3.04964E-28
ENSMUSG00000053198	1.333	0.652333333	-1.030995525	0.005120955	0.001950552
ENSMUSG00000024777	22.925	11.21666667	-1.03127773	5.96497E-17	5.87588E-18
ENSMUSG00000046070	2.997333333	1.465666667	-1.032122503	0.000338021	0.000105529
ENSMUSG00000037337	16.87966667	8.252666667	-1.03235414	6.58596E-17	6.51047E-18
ENSMUSG00000027381	14.563	7.12	-1.032358437	2.47927E-16	2.53352E-17
ENSMUSG00000055725	4.118666667	2.011666667	-1.0337861	0.000339046	0.000105918
ENSMUSG00000031010	92.648	45.251	-1.033810158	5.46891E-68	1.16255E-69
ENSMUSG00000004270	42.07433333	20.535	-1.034855465	3.01423E-24	2.02694E-25
ENSMUSG00000024579	21.483	10.48333333	-1.035097957	4.05157E-14	4.77914E-15
ENSMUSG00000020823	21.76	10.61266667	-1.035891346	5.46567E-27	3.27295E-28
ENSMUSG00000028893	42.355	20.64766667	-1.036553533	6.83064E-25	4.47942E-26

ENSMUSG00000020289	14.30066667	6.967666667	-1.037334892	4.26186E-12	5.86802E-13
ENSMUSG00000015647	5.944333333	2.894666667	-1.038117793	5.50889E-14	6.57852E-15
ENSMUSG00000045482	25.001	12.17333333	-1.038261537	8.55322E-26	5.35356E-27
ENSMUSG00000021238	10.29866667	5.014333333	-1.038327759	7.06383E-12	9.90262E-13
ENSMUSG00000024773	19.89866667	9.681333333	-1.039394107	1.58478E-37	6.84775E-39
ENSMUSG00000049858	10.65166667	5.177333333	-1.040798075	3.34399E-09	5.87956E-10
ENSMUSG00000003528	78.784	38.27466667	-1.041512854	8.34846E-22	6.30412E-23
ENSMUSG00000025888	5.233	2.542	-1.041674229	0.012007804	0.004933251
ENSMUSG00000037890	3.376666667	1.64	-1.041903953	5.16381E-06	1.2672E-06
ENSMUSG00000041915	16.29166667	7.912	-1.04201987	2.99026E-20	2.44912E-21
ENSMUSG00000018425	26.70266667	12.96533333	-1.042324526	4.87089E-29	2.71376E-30
ENSMUSG00000066036	50.457	24.47766667	-1.043588391	5.36244E-39	2.2165E-40
ENSMUSG00000034868	165.366	80.09666667	-1.045848531	2.64511E-19	2.27302E-20
ENSMUSG00000029119	44.128	21.37233333	-1.045949939	1.33697E-39	5.42404E-41
ENSMUSG00000035840	13.992	6.776	-1.046096415	1.84786E-11	2.67263E-12
ENSMUSG00000033624	19.57	9.475666667	-1.046344402	3.26878E-19	2.83392E-20
ENSMUSG00000024339	71.69566667	34.69033333	-1.047352221	5.32734E-24	3.63791E-25
ENSMUSG00000038212	51.02533333	24.687	-1.047462173	7.6547E-31	3.96694E-32
ENSMUSG00000026672	18.32733333	8.86	-1.048618282	2.61996E-12	3.54182E-13
ENSMUSG00000038797	4.282666667	2.070333333	-1.048646325	0.011556759	0.004730224
ENSMUSG00000038936	21.32566667	10.298	-1.050226667	1.35804E-10	2.11041E-11
ENSMUSG00000041890	40.033	19.32633333	-1.050621783	6.00134E-30	3.21433E-31
ENSMUSG00000030231	29.05233333	14.00033333	-1.053192861	4.78571E-23	3.40103E-24
ENSMUSG00000038893	27.78766667	13.388	-1.05350424	0.003686686	0.001369359
ENSMUSG00000036918	30.03566667	14.441	-1.056506038	7.29794E-38	3.11284E-39
ENSMUSG00000024862	11.79133333	5.669	-1.056560689	3.56744E-10	5.73219E-11
ENSMUSG00000025094	139.5266667	67.06466667	-1.056916099	4.357E-41	1.69498E-42
ENSMUSG00000024006	47.59533333	22.87433333	-1.057090429	2.07633E-41	7.97644E-43
ENSMUSG00000028552	108.8133333	52.29	-1.057248371	1.01007E-56	2.6664E-58
ENSMUSG00000021144	59.20633333	28.41633333	-1.0590311	6.45425E-39	2.67675E-40
ENSMUSG00000013236	41.48733333	19.897	-1.060120006	4.545E-24	3.07841E-25
ENSMUSG00000038065	2.822333333	1.353333333	-1.060371162	0.000505476	0.000162265

ENSMUSG00000015944	4.609	2.208	-1.061713596	1.99802E-10	3.14243E-11
ENSMUSG00000021738	10.05066667	4.81	-1.0631824	5.05885E-17	4.96572E-18
ENSMUSG00000053581	10.99566667	5.259333333	-1.063983235	5.62945E-14	6.7264E-15
ENSMUSG00000070000	60.94233333	29.13933333	-1.064476867	1.10271E-26	6.67221E-28
ENSMUSG00000028878	19.324	9.231333333	-1.065782813	3.87206E-19	3.37309E-20
ENSMUSG00000044199	4.354666667	2.08	-1.065978762	0.000266143	8.19413E-05
ENSMUSG00000020577	16.12666667	7.702666667	-1.06601837	1.06106E-09	1.78084E-10
ENSMUSG00000039354	26.11533333	12.47166667	-1.066242844	2.8004E-25	1.80144E-26
ENSMUSG00000015599	2.959	1.413	-1.066348231	1.3041E-14	1.49299E-15
ENSMUSG00000036568	11.92466667	5.694	-1.066434541	6.67455E-11	1.01312E-11
ENSMUSG00000004931	21.853	10.43066667	-1.066999978	5.27071E-14	6.27579E-15
ENSMUSG00000025068	223.21	106.528	-1.067168982	6.51289E-58	1.70118E-59
ENSMUSG00000024127	27.37966667	13.06266667	-1.067655438	3.61854E-21	2.82043E-22
ENSMUSG00000036109	14.00033333	6.679	-1.067757157	6.40367E-20	5.3249E-21
ENSMUSG00000064368	914.4783333	436.1683333	-1.068063957	3.33975E-21	2.59848E-22
ENSMUSG00000029312	1.828666667	0.871	-1.070047498	0.003224942	0.00118625
ENSMUSG00000041491	20.318	9.676333333	-1.070226024	4.63789E-13	5.92826E-14
ENSMUSG00000010095	276.999	131.8943333	-1.070498186	1.18218E-32	5.8144E-34
ENSMUSG00000046761	11.89166667	5.662	-1.070567275	3.01683E-17	2.92148E-18
ENSMUSG00000020733	82.21	39.13466667	-1.070866737	9.52626E-31	4.9567E-32
ENSMUSG00000020048	831.2236667	395.63	-1.071084904	3.15638E-63	7.4113E-65
ENSMUSG00000061533	23.27166667	11.07366667	-1.071441537	8.2736E-20	6.94304E-21
ENSMUSG00000003458	43.287	20.57166667	-1.073275136	5.77576E-41	2.26296E-42
ENSMUSG00000029311	48.645	23.11666667	-1.073358143	2.1643E-12	2.91379E-13
ENSMUSG00000037822	27.12433333	12.88966667	-1.073372724	2.52813E-11	3.69868E-12
ENSMUSG00000028576	6.397	3.037333333	-1.074590237	7.67942E-07	1.71974E-07
ENSMUSG00000046324	25.20233333	11.94766667	-1.076828417	3.37498E-10	5.41122E-11
ENSMUSG00000004788	52.78133333	25.01833333	-1.077042112	1.25406E-29	6.83002E-31
ENSMUSG00000027309	6.767	3.206333333	-1.077591971	1.95556E-07	4.07957E-08
ENSMUSG00000024687	63.775	30.209	-1.078012566	1.5681E-46	5.16346E-48
ENSMUSG00000003762	5.397666667	2.556	-1.07844805	6.24869E-06	1.54622E-06
ENSMUSG00000040865	12.73433333	6.03	-1.078493527	2.70626E-33	1.31224E-34

ENSMUSG00000024666	18.86966667	8.932333333	-1.078959943	1.55368E-09	2.64002E-10
ENSMUSG00000013663	63.73466667	30.091	-1.082746247	4.07136E-49	1.26426E-50
ENSMUSG00000027366	37.893	17.89033333	-1.082751095	6.41668E-26	4.01182E-27
ENSMUSG00000003344	14.725	6.951	-1.082975184	2.24408E-07	4.71888E-08
ENSMUSG00000029826	130.4036667	61.53433333	-1.083520938	1.46311E-66	3.21183E-68
ENSMUSG00000106631	31.48	14.84766667	-1.084199314	4.09273E-18	3.78425E-19
ENSMUSG00000040616	8.194333333	3.864	-1.084531487	3.20261E-06	7.66002E-07
ENSMUSG00000024457	12.00133333	5.658333333	-1.084745623	1.9833E-14	2.28985E-15
ENSMUSG00000042249	4.76	2.243666667	-1.085103218	2.05125E-10	3.23185E-11
ENSMUSG00000020189	45.37566667	21.376	-1.085926927	0.00011169	3.26729E-05
ENSMUSG00000056749	16.34933333	7.694666667	-1.087301074	8.86454E-14	1.07335E-14
ENSMUSG00000021036	27.01333333	12.706	-1.088161749	7.93676E-35	3.70511E-36
ENSMUSG00000031887	10.945	5.148	-1.088187996	5.22175E-07	1.14664E-07
ENSMUSG00000030768	8.124666667	3.816666667	-1.089995432	7.62234E-13	9.8913E-14
ENSMUSG00000028458	11.89733333	5.586	-1.090750766	1.38895E-09	2.3495E-10
ENSMUSG00000024963	11.174	5.236333333	-1.093516881	5.16381E-06	1.26773E-06
ENSMUSG00000020284	4.623333333	2.166333333	-1.093678135	0.000129713	3.82965E-05
ENSMUSG00000020821	23.60233333	11.055	-1.094230467	2.37124E-37	1.02954E-38
ENSMUSG00000021176	7.642333333	3.577	-1.095263066	0.000772842	0.000255341
ENSMUSG00000024642	40.13133333	18.77366667	-1.09601864	2.37888E-42	8.84128E-44
ENSMUSG00000031782	27.94533333	13.05866667	-1.097599783	3.76281E-19	3.27269E-20
ENSMUSG00000063015	137.4226667	64.205	-1.097862426	1.45726E-44	5.05155E-46
ENSMUSG00000062590	5.055666667	2.359666667	-1.099318271	0.002581379	0.000933923
ENSMUSG00000069729	8.605	4.015	-1.099775205	2.57354E-21	1.98625E-22
ENSMUSG00000078865	9.19	4.286666667	-1.100208625	2.06386E-06	4.84074E-07
ENSMUSG00000040209	8.737333333	4.072666667	-1.101219291	4.16013E-20	3.41596E-21
ENSMUSG00000021009	4.753333333	2.213	-1.102936125	1.48049E-08	2.77689E-09
ENSMUSG00000117694	6.102	2.84	-1.10339125	0.027667477	0.012258643
ENSMUSG00000038025	11.77633333	5.480666667	-1.103467115	6.51546E-23	4.68008E-24
ENSMUSG00000035713	0.961	0.447	-1.1042616	0.005957052	0.002297158
ENSMUSG00000032575	250.3696667	116.1593333	-1.107954706	9.42496E-50	2.82192E-51
ENSMUSG00000024962	36.32033333	16.84066667	-1.108828192	7.77322E-19	6.9258E-20

ENSMUSG00000057219	7.063666667	3.274666667	-1.109069204	4.21108E-08	8.19691E-09
ENSMUSG00000025257	1.773666667	0.821333333	-1.110695147	0.045418005	0.021161414
ENSMUSG00000041000	5.920333333	2.736666667	-1.113258685	9.51373E-08	1.92191E-08
ENSMUSG00000018678	15.733	7.271333333	-1.113501955	8.68355E-18	8.16779E-19
ENSMUSG00000029598	14.766	6.824333333	-1.113519042	3.72942E-21	2.91462E-22
ENSMUSG00000001482	42.271	19.516	-1.115010854	1.13132E-26	6.85313E-28
ENSMUSG00000008393	20.73133333	9.566	-1.11582521	1.7046E-16	1.73006E-17
ENSMUSG00000031983	10.31166667	4.756	-1.116456913	3.03509E-08	5.82349E-09
ENSMUSG00000028850	6.113666667	2.819	-1.116854417	0.000236815	7.22374E-05
ENSMUSG00000002043	41.101	18.94633333	-1.117254824	1.23076E-13	1.50992E-14
ENSMUSG00000052296	138.865	63.90133333	-1.119765084	6.68568E-70	1.37011E-71
ENSMUSG00000029270	7.491	3.445666667	-1.12037518	3.72024E-05	1.01696E-05
ENSMUSG00000020097	29.132	13.397	-1.120694777	4.53964E-20	3.74019E-21
ENSMUSG00000103144	2.825	1.297	-1.123072388	1.35572E-05	3.49503E-06
ENSMUSG00000024921	57.74066667	26.50633333	-1.123250651	2.24114E-52	6.30539E-54
ENSMUSG00000095687	63.00033333	28.91833333	-1.123375055	5.04682E-24	3.43584E-25
ENSMUSG00000036820	13.613	6.244666667	-1.12428857	1.30689E-08	2.43857E-09
ENSMUSG00000063535	2.399666667	1.1	-1.125330494	1.7743E-05	4.62958E-06
ENSMUSG00000016534	71.54433333	32.761	-1.126858108	2.48441E-19	2.12974E-20
ENSMUSG00000029516	29.718	13.60333333	-1.127376817	1.32181E-37	5.68394E-39
ENSMUSG00000026307	32.64466667	14.94	-1.127667162	6.37646E-16	6.6356E-17
ENSMUSG00000030035	29.422	13.45566667	-1.128681447	4.62665E-14	5.49606E-15
ENSMUSG00000020919	33.96766667	15.526	-1.129475926	5.44785E-44	1.92633E-45
ENSMUSG00000026797	14.15666667	6.470333333	-1.129569665	7.37977E-20	6.17245E-21
ENSMUSG00000021420	14.988	6.846666667	-1.130334202	6.97122E-11	1.06009E-11
ENSMUSG00000089832	23.847	10.89166667	-1.130583048	2.17658E-24	1.45458E-25
ENSMUSG00000043372	15.65066667	7.146333333	-1.130948999	2.79404E-06	6.64785E-07
ENSMUSG00000039879	15.512	7.076	-1.132378755	1.85232E-20	1.50296E-21
ENSMUSG00000005362	25.889	11.795	-1.134163822	1.75596E-21	1.34426E-22
ENSMUSG00000038763	5.585666667	2.544666667	-1.134252795	6.07139E-10	9.95378E-11
ENSMUSG00000022964	14.95466667	6.809	-1.135080915	1.93586E-07	4.03443E-08
ENSMUSG00000030780	11.29766667	5.139666667	-1.136278139	5.4713E-13	7.03154E-14

ENSMUSG00000019977	58.72466667	26.7	-1.137126877	2.62808E-39	1.07351E-40
ENSMUSG00000018401	17.36833333	7.888	-1.138727863	3.27194E-17	3.18216E-18
ENSMUSG00000039021	4.483666667	2.033333333	-1.140832285	1.31456E-05	3.38708E-06
ENSMUSG00000039199	2.149333333	0.974	-1.141895566	0.005919229	0.002280106
ENSMUSG00000045466	6.909333333	3.131	-1.141923006	2.35368E-07	4.95752E-08
ENSMUSG00000032606	9.718666667	4.399666667	-1.143364175	1.61833E-09	2.75774E-10
ENSMUSG00000028496	11.84833333	5.361666667	-1.143930699	3.35405E-09	5.89959E-10
ENSMUSG00000063605	3.599	1.625333333	-1.146860477	0.002001979	0.000711088
ENSMUSG00000038248	2.298333333	1.037666667	-1.147244975	3.5109E-06	8.44371E-07
ENSMUSG00000021266	75.30033333	33.98633333	-1.147701528	1.09229E-25	6.85956E-27
ENSMUSG00000005893	21.43433333	9.669	-1.148484952	2.07706E-44	7.22896E-46
ENSMUSG00000001761	2.226666667	1.004333333	-1.14864743	5.89384E-05	1.65781E-05
ENSMUSG00000062901	17.62666667	7.945666667	-1.1495195	2.29656E-13	2.86532E-14
ENSMUSG00000059923	189.5623333	85.416	-1.150094081	1.5419E-49	4.67016E-51
ENSMUSG00000021120	14.11	6.355333333	-1.150678287	4.93376E-13	6.327E-14
ENSMUSG00000034361	88.09	39.659	-1.151329953	3.16986E-42	1.1803E-43
ENSMUSG00000040482	26.75233333	12.03866667	-1.151989112	1.30077E-16	1.30665E-17
ENSMUSG00000041949	2.79	1.253333333	-1.154494961	3.33233E-07	7.15079E-08
ENSMUSG00000036391	16.21966667	7.284333333	-1.154875324	1.38283E-24	9.19323E-26
ENSMUSG00000020462	28.17266667	12.64633333	-1.155576976	3.50196E-13	4.43736E-14
ENSMUSG00000068114	8.885	3.986	-1.156430066	4.2301E-08	8.23686E-09
ENSMUSG00000034175	13.38833333	6.003	-1.157220803	2.7431E-08	5.23467E-09
ENSMUSG00000019528	81.353	36.416	-1.159623085	4.76204E-26	2.96408E-27
ENSMUSG00000035151	9.444	4.217333333	-1.163066986	3.65189E-13	4.63495E-14
ENSMUSG00000016528	137.6013333	61.403	-1.164113401	1.61645E-64	3.71687E-66
ENSMUSG00000003849	6.813666667	3.035	-1.166734854	3.09001E-06	7.37781E-07
ENSMUSG00000020432	42.516	18.937	-1.166798074	9.71521E-16	1.0272E-16
ENSMUSG00000012117	35.29366667	15.70466667	-1.168215998	1.25207E-29	6.81049E-31
ENSMUSG00000094030	23.25033333	10.339	-1.169154747	4.7165E-15	5.24238E-16
ENSMUSG00000072915	1.407	0.625333333	-1.169925001	0.002154687	0.00076997
ENSMUSG00000020668	7.766333333	3.449333333	-1.170916076	1.79452E-10	2.81363E-11
ENSMUSG00000020715	22.05733333	9.796333333	-1.170944614	3.04088E-21	2.3575E-22

ENSMUSG00000021066	0.812666667	0.360333333	-1.173331603	0.001645971	0.00057629
ENSMUSG00000031570	37.298	16.51833333	-1.175030143	2.9424E-24	1.97454E-25
ENSMUSG00000021514	14.86933333	6.585	-1.17508462	6.89786E-13	8.94158E-14
ENSMUSG00000019158	28.91866667	12.79266667	-1.176684007	1.65292E-11	2.37805E-12
ENSMUSG00000041135	7.958	3.518666667	-1.177377049	1.37062E-11	1.95666E-12
ENSMUSG00000028641	16.842	7.443333333	-1.178042719	5.87001E-23	4.18791E-24
ENSMUSG00000091945	1.674666667	0.739666667	-1.178926797	1.75765E-05	4.58368E-06
ENSMUSG00000037470	62.823	27.73166667	-1.179758513	4.67205E-46	1.56114E-47
ENSMUSG00000079334	14.39866667	6.355666667	-1.179819855	1.24292E-12	1.64486E-13
ENSMUSG00000028779	39.67566667	17.48933333	-1.181779168	8.82873E-16	9.26725E-17
ENSMUSG00000047648	12.677	5.587	-1.182067648	1.62987E-16	1.64968E-17
ENSMUSG00000001441	47.576	20.957	-1.182801771	1.49985E-41	5.72017E-43
ENSMUSG00000058385	8.215333333	3.617333333	-1.183392566	0.001708979	0.000599538
ENSMUSG00000037685	17.731	7.804	-1.183988221	9.3517E-16	9.86495E-17
ENSMUSG00000041609	5.094333333	2.242	-1.184107083	7.22528E-07	1.6112E-07
ENSMUSG00000102748	7.313666667	3.214666667	-1.185925725	1.90394E-16	1.93899E-17
ENSMUSG00000023066	7.325666667	3.217333333	-1.187094642	7.44488E-28	4.28746E-29
ENSMUSG00000029499	3.303666667	1.450666667	-1.187352073	0.043674765	0.020282445
ENSMUSG00000037103	26.80766667	11.771	-1.187408764	7.6413E-24	5.25522E-25
ENSMUSG00000039740	29.71333333	13.04533333	-1.187576652	3.73309E-36	1.67269E-37
ENSMUSG00000028256	13.21566667	5.800666667	-1.187958581	2.48315E-11	3.63114E-12
ENSMUSG00000020647	19.727	8.655333333	-1.188510287	1.76133E-38	7.35364E-40
ENSMUSG00000017718	5.096666667	2.232	-1.191216974	8.51282E-06	2.14018E-06
ENSMUSG00000066894	3.588666667	1.569666667	-1.192989703	2.83901E-05	7.62013E-06
ENSMUSG00000020393	7.807	3.412666667	-1.193868764	6.60421E-17	6.5331E-18
ENSMUSG00000036091	2.149	0.938666667	-1.19498065	0.013934446	0.005795452
ENSMUSG00000038312	47.18433333	20.59133333	-1.196270669	1.40163E-41	5.33584E-43
ENSMUSG00000028145	6.151666667	2.681666667	-1.197847412	4.00045E-06	9.68778E-07
ENSMUSG00000023908	20.90366667	9.084666667	-1.202250539	1.41789E-20	1.13963E-21
ENSMUSG00000057778	1.826666667	0.792666667	-1.204427178	0.003923418	0.001467432
ENSMUSG00000020743	17.09366667	7.415	-1.204943297	9.70379E-13	1.26732E-13
ENSMUSG00000002763	19.89133333	8.618333333	-1.20665918	9.16536E-18	8.63371E-19

ENSMUSG00000046805	1.781	0.771	-1.207884751	0.017587592	0.007476101
ENSMUSG00000021900	13.88	6	-1.209973162	5.87286E-17	5.77698E-18
ENSMUSG00000031970	4.334	1.871333333	-1.21163259	0.001023663	0.000345414
ENSMUSG00000021234	1.265333333	0.546333333	-1.211664138	0.008144134	0.003237852
ENSMUSG00000031967	41.46266667	17.90033333	-1.211826453	1.68905E-49	5.12757E-51
ENSMUSG00000041168	108.687	46.914	-1.212088972	2.95988E-74	5.79845E-76
ENSMUSG00000045538	12.81266667	5.529	-1.212480294	7.56397E-14	9.10093E-15
ENSMUSG00000049807	4.153666667	1.792333333	-1.212546473	7.70759E-14	9.2791E-15
ENSMUSG00000029725	14.881	6.419333333	-1.212976096	6.25743E-07	1.38624E-07
ENSMUSG00000052751	7.350666667	3.169666667	-1.213543971	6.39368E-13	8.27026E-14
ENSMUSG00000028295	8.805	3.792333333	-1.215237226	0.000185993	5.6037E-05
ENSMUSG00000024614	32.89966667	14.168	-1.21543685	2.21825E-20	1.80604E-21
ENSMUSG00000039461	11.93066667	5.135666667	-1.216051189	7.54002E-09	1.37182E-09
ENSMUSG00000056185	5.100333333	2.193666667	-1.217247217	0.000129158	3.81149E-05
ENSMUSG00000016427	21.251	9.137666667	-1.217633011	0.000121883	3.58578E-05
ENSMUSG00000039616	5.648666667	2.426333333	-1.219132605	8.31313E-08	1.67071E-08
ENSMUSG00000034341	114.1893333	49.01566667	-1.220113041	4.24955E-58	1.10113E-59
ENSMUSG00000026223	50.47966667	21.65466667	-1.221024419	9.05308E-21	7.18838E-22
ENSMUSG00000027778	16.67066667	7.144666667	-1.22237319	2.74987E-26	1.69252E-27
ENSMUSG00000031629	25.64233333	10.97733333	-1.223999916	9.39184E-07	2.11846E-07
ENSMUSG00000029484	71.441	30.56466667	-1.224887441	0.017809394	0.007580282
ENSMUSG00000043987	11.83966667	5.062666667	-1.225659061	6.79428E-23	4.88508E-24
ENSMUSG00000029403	2.601666667	1.111	-1.227577315	0.001182081	0.000402868
ENSMUSG00000031974	27.85333333	11.88666667	-1.228505789	1.20392E-25	7.56895E-27
ENSMUSG00000038417	3.170666667	1.352666667	-1.22897985	0.000551433	0.000177899
ENSMUSG00000015533	0.422	0.18	-1.229246092	0.028485063	0.012646616
ENSMUSG00000028211	14.52666667	6.188333333	-1.231080881	6.16876E-13	7.97075E-14
ENSMUSG00000028830	21.70233333	9.239333333	-1.2319895	1.0647E-31	5.39929E-33
ENSMUSG00000033720	0.996	0.424	-1.232081478	0.010931351	0.004455313
ENSMUSG00000072707	1.644333333	0.698666667	-1.23482657	0.007909623	0.003133079
ENSMUSG00000033985	5.094666667	2.160333333	-1.237733824	8.15764E-09	1.48985E-09
ENSMUSG00000055210	1.726333333	0.732	-1.237795504	0.013570413	0.00563085

ENSMUSG00000035954	0.306666667	0.13	-1.238159737	0.045601189	0.021275275
ENSMUSG00000068129	74.93433333	31.76133333	-1.238355405	1.79419E-19	1.52434E-20
ENSMUSG00000035172	5.202666667	2.205	-1.238472621	3.32827E-08	6.40683E-09
ENSMUSG00000040359	23.23666667	9.841666667	-1.239428568	6.84504E-29	3.84216E-30
ENSMUSG00000029648	1.354333333	0.573333333	-1.2401368	3.4916E-06	8.39487E-07
ENSMUSG00000045980	8.965666667	3.783333333	-1.244752971	3.92835E-16	4.04433E-17
ENSMUSG00000001123	136.085	57.416	-1.244983323	1.5512E-26	9.43977E-28
ENSMUSG00000018654	2.697666667	1.138	-1.245211537	0.022514346	0.009815911
ENSMUSG00000051499	0.680666667	0.287	-1.245897723	0.042248175	0.019543633
ENSMUSG00000042148	27.29033333	11.50566667	-1.246045437	4.4186E-29	2.45563E-30
ENSMUSG00000060376	27.41333333	11.55566667	-1.24627727	2.55759E-17	2.45898E-18
ENSMUSG00000018841	22.404	9.443333333	-1.246388232	1.45108E-25	9.15305E-27
ENSMUSG00000073664	3.604333333	1.518666667	-1.246927193	1.30724E-25	8.2276E-27
ENSMUSG00000058756	19.73	8.296333333	-1.24984519	1.52962E-15	1.64385E-16
ENSMUSG00000019842	5.368333333	2.255333333	-1.251133578	5.84071E-06	1.44121E-06
ENSMUSG00000042349	8.681	3.646666667	-1.25128291	7.78815E-14	9.38149E-15
ENSMUSG00000032468	26.15633333	10.986	-1.251494117	4.80548E-42	1.80602E-43
ENSMUSG00000035311	27.498	11.52233333	-1.254893792	1.19252E-47	3.8439E-49
ENSMUSG00000036661	9.487	3.974666667	-1.255118073	2.60713E-21	2.01398E-22
ENSMUSG00000045795	16.75766667	7	-1.259394455	1.31919E-20	1.05664E-21
ENSMUSG00000022848	2.271666667	0.948666667	-1.259777995	0.000960012	0.000322249
ENSMUSG00000002996	51.70533333	21.57833333	-1.260729662	6.32042E-20	5.25129E-21
ENSMUSG00000022951	15.24666667	6.362	-1.260941588	9.5265E-15	1.07938E-15
ENSMUSG00000033862	24.99566667	10.42266667	-1.261953564	2.83739E-17	2.74179E-18
ENSMUSG00000037098	5.038	2.100333333	-1.262232813	2.39444E-12	3.23029E-13
ENSMUSG00000078624	0.910333333	0.378666667	-1.265466479	0.000777215	0.000257002
ENSMUSG00000073771	6.467	2.684	-1.268711939	4.41071E-06	1.07303E-06
ENSMUSG00000021676	38.64866667	16.03933333	-1.268804466	6.97653E-33	3.41678E-34
ENSMUSG00000004508	22.65966667	9.386666667	-1.271441805	1.62955E-56	4.33565E-58
ENSMUSG00000026209	86.70733333	35.89033333	-1.272558693	3.84354E-33	1.87438E-34
ENSMUSG00000005951	2.981666667	1.232	-1.275116726	8.29453E-05	2.38262E-05
ENSMUSG00000051457	69.25266667	28.60766667	-1.275467794	1.10244E-45	3.72969E-47

ENSMUSG00000084883	4.432666667	1.828666667	-1.277382755	4.87456E-09	8.68583E-10
ENSMUSG00000033701	74.04466667	30.48766667	-1.280170084	9.77831E-20	8.23294E-21
ENSMUSG00000086158	4.891	2.013666667	-1.280304579	0.010022581	0.004056378
ENSMUSG00000022394	35.64366667	14.66566667	-1.281203101	9.45666E-53	2.64747E-54
ENSMUSG00000049550	11.67366667	4.802333333	-1.281450328	2.34036E-27	1.38032E-28
ENSMUSG00000053040	2.757	1.133333333	-1.282527022	0.014219694	0.005927918
ENSMUSG00000015363	60.82966667	25.00333333	-1.282654657	1.77565E-40	7.03107E-42
ENSMUSG00000032479	44.406	18.24366667	-1.283358906	7.64361E-45	2.63902E-46
ENSMUSG00000021884	13.495	5.543333333	-1.283599309	1.78474E-13	2.21187E-14
ENSMUSG00000038290	23.924	9.822	-1.284369895	3.4602E-66	7.692E-68
ENSMUSG00000029165	12.429	5.086	-1.289106859	6.93901E-11	1.05471E-11
ENSMUSG00000044456	38.972	15.939	-1.289876852	4.11626E-52	1.16382E-53
ENSMUSG00000067889	1.697333333	0.694	-1.290262351	7.80114E-08	1.56348E-08
ENSMUSG00000038593	1.751333333	0.715333333	-1.291766124	1.25098E-05	3.21719E-06
ENSMUSG00000039156	30.97833333	12.64833333	-1.292312234	1.02093E-45	3.43974E-47
ENSMUSG00000029004	32.959	13.45133333	-1.292923287	1.61994E-42	5.99813E-44
ENSMUSG00000022359	12.09166667	4.931333333	-1.293963433	5.45666E-11	8.22953E-12
ENSMUSG00000051650	108.7096667	44.27	-1.296078955	2.11848E-23	1.47757E-24
ENSMUSG00000025283	71.308	29.036	-1.296221219	0.000173798	5.20729E-05
ENSMUSG00000003812	24.904	10.13933333	-1.296414684	1.53222E-17	1.45292E-18
ENSMUSG00000032297	2.410666667	0.981	-1.297107135	0.000185825	5.59605E-05
ENSMUSG00000021661	15.42666667	6.277333333	-1.297202639	1.9636E-13	2.44171E-14
ENSMUSG00000026239	69.83033333	28.395	-1.298216948	1.31049E-25	8.25715E-27
ENSMUSG00000034570	5.739666667	2.333666667	-1.298368448	3.3141E-09	5.82471E-10
ENSMUSG00000034723	22.765	9.254	-1.298668455	2.5768E-46	8.5028E-48
ENSMUSG00000001751	20.132	8.176	-1.300023402	2.04263E-26	1.25012E-27
ENSMUSG00000022742	37.81333333	15.352	-1.300468415	1.10248E-42	4.07445E-44
ENSMUSG00000095545	53.859	21.85833333	-1.301004042	3.01981E-35	1.38456E-36
ENSMUSG00000020818	13.62933333	5.529333333	-1.301537544	2.23014E-20	1.81882E-21
ENSMUSG00000039158	6.567333333	2.662666667	-1.302435851	1.98589E-17	1.89509E-18
ENSMUSG00000020873	58.72433333	23.766	-1.305059323	6.02022E-32	3.03625E-33
ENSMUSG00000066735	34.51566667	13.95833333	-1.30612466	4.08698E-29	2.26849E-30

ENSMUSG00000034993	110.0996667	44.52066667	-1.306263	3.73521E-49	1.15209E-50
ENSMUSG00000038732	29.14966667	11.77166667	-1.30816079	9.91989E-29	5.63701E-30
ENSMUSG00000056832	1.207	0.487	-1.309431999	0.00021077	6.39264E-05
ENSMUSG00000020590	13.464	5.422666667	-1.312032687	4.66836E-29	2.59767E-30
ENSMUSG00000066440	10.22133333	4.116666667	-1.312034861	3.3178E-43	1.20543E-44
ENSMUSG00000018427	9.545666667	3.843333333	-1.312487851	6.21756E-16	6.46159E-17
ENSMUSG00000031133	40.71566667	16.30433333	-1.320328573	2.84568E-28	1.62497E-29
ENSMUSG00000089876	7.383	2.955333333	-1.320886297	3.7881E-09	6.70254E-10
ENSMUSG00000026360	11.128	4.453333333	-1.321236818	0.028896685	0.012853454
ENSMUSG00000032750	3.84	1.534333333	-1.323494369	1.35382E-05	3.48918E-06
ENSMUSG00000048410	6.109333333	2.438	-1.325316832	2.42593E-15	2.64585E-16
ENSMUSG00000025571	23.68433333	9.447333333	-1.325953996	1.65881E-72	3.3303E-74
ENSMUSG00000042647	3.85	1.535666667	-1.325993349	5.09722E-11	7.65911E-12
ENSMUSG00000047617	6.037	2.408	-1.325996409	7.5601E-05	2.16063E-05
ENSMUSG00000035868	2.427	0.968	-1.326095156	0.004592172	0.001735584
ENSMUSG00000034430	8.162333333	3.251666667	-1.327802256	1.73742E-11	2.50444E-12
ENSMUSG00000038615	60.13	23.94733333	-1.328219946	1.62993E-50	4.81223E-52
ENSMUSG00000028757	165.6353333	65.92433333	-1.32912748	7.50593E-73	1.50171E-74
ENSMUSG00000039623	11.66466667	4.642	-1.329326653	1.48124E-12	1.97155E-13
ENSMUSG00000028124	107.4813333	42.71533333	-1.331260178	2.17776E-05	5.75957E-06
ENSMUSG00000033955	1.509666667	0.599666667	-1.331997353	0.010851928	0.004421435
ENSMUSG00000028689	20.88866667	8.295	-1.332406521	6.8553E-10	1.13104E-10
ENSMUSG00000029752	275.9253333	109.4563333	-1.333922486	1.63968E-42	6.08258E-44
ENSMUSG00000033581	4.047	1.603	-1.336078424	5.7896E-05	1.62567E-05
ENSMUSG00000070732	3.338333333	1.320666667	-1.337861636	0.000129829	3.83398E-05
ENSMUSG00000018931	9.695	3.835	-1.338014321	7.61387E-17	7.56891E-18
ENSMUSG00000037235	23.866	9.435333333	-1.338811396	3.7891E-17	3.70092E-18
ENSMUSG00000009145	3.259	1.286333333	-1.341164809	1.49642E-07	3.08536E-08
ENSMUSG00000016526	8.792	3.47	-1.341255723	1.02562E-12	1.34589E-13
ENSMUSG00000020986	23.57066667	9.299333333	-1.341793365	7.41217E-39	3.08433E-40
ENSMUSG00000045282	6.923666667	2.730666667	-1.342283055	9.18989E-05	2.65578E-05
ENSMUSG00000072825	18.81066667	7.417666667	-1.342513637	4.04828E-49	1.25428E-50

ENSMUSG00000030000	0.596666667	0.235	-1.344264425	0.022491337	0.009801192
ENSMUSG00000016206	11.17333333	4.396333333	-1.345686967	4.34909E-08	8.49576E-09
ENSMUSG00000031901	9.059666667	3.564666667	-1.345690794	7.43686E-09	1.35253E-09
ENSMUSG00000034371	17.39366667	6.835333333	-1.347478492	4.12562E-18	3.82039E-19
ENSMUSG00000047746	1.126666667	0.442666667	-1.3477681	0.000359626	0.000112872
ENSMUSG00000001014	5.498666667	2.160333333	-1.347827899	0.00110821	0.000375998
ENSMUSG00000029070	4.952	1.945333333	-1.347993932	3.67969E-07	7.94475E-08
ENSMUSG00000040297	27.06233333	10.612	-1.350589654	7.06008E-24	4.84568E-25
ENSMUSG00000034574	12.18066667	4.775666667	-1.350819049	5.16533E-35	2.40415E-36
ENSMUSG00000042350	40.66366667	15.91466667	-1.35338337	4.78934E-60	1.18444E-61
ENSMUSG00000001583	1.073333333	0.42	-1.353636955	0.028837966	0.012823329
ENSMUSG00000007837	2.867	1.121666667	-1.3538979	0.001093377	0.000370738
ENSMUSG00000008305	41.48733333	16.20833333	-1.355935179	2.64574E-43	9.57575E-45
ENSMUSG00000096463	17.52966667	6.846333333	-1.356395122	7.26072E-10	1.20096E-10
ENSMUSG00000041538	3.785	1.477666667	-1.356972438	0.002284084	0.0008197
ENSMUSG00000090353	4.050333333	1.58	-1.358116085	0.000478154	0.000152896
ENSMUSG00000040822	28.30733333	11.03933333	-1.358522798	5.71973E-15	6.40912E-16
ENSMUSG00000030671	26.759	10.43333333	-1.358824046	2.38024E-54	6.49834E-56
ENSMUSG00000032492	1.248666667	0.486666667	-1.35938253	0.039322259	0.018054295
ENSMUSG00000013155	17.202	6.699333333	-1.360486869	3.64166E-14	4.28297E-15
ENSMUSG00000047604	41.362	16.10433333	-1.360857008	1.35611E-32	6.68872E-34
ENSMUSG00000028799	28.46133333	11.078	-1.361305806	4.46253E-40	1.77943E-41
ENSMUSG00000041372	0.497666667	0.193666667	-1.361604097	0.033405723	0.015107416
ENSMUSG00000110277	6.686666667	2.598666667	-1.36351561	0.000126252	3.72048E-05
ENSMUSG00000061046	4.854333333	1.884333333	-1.365218981	4.00986E-05	1.10087E-05
ENSMUSG00000036854	6.390333333	2.471666667	-1.370406995	6.73257E-07	1.49758E-07
ENSMUSG00000022911	14.12066667	5.460333333	-1.370747273	3.20105E-21	2.48612E-22
ENSMUSG00000045679	31.00366667	11.98633333	-1.371048447	7.1339E-20	5.95689E-21
ENSMUSG00000052331	54.349	21.00233333	-1.371703872	1.37262E-17	1.29872E-18
ENSMUSG00000049791	5.770666667	2.228666667	-1.372557144	1.80634E-22	1.31633E-23
ENSMUSG00000024308	40.569	15.66266667	-1.37304788	5.31321E-38	2.24413E-39
ENSMUSG00000031103	16.13766667	6.214	-1.376837849	8.08915E-28	4.66412E-29

ENSMUSG00000074220	5.555	2.134	-1.380226735	0.002144691	0.000765951
ENSMUSG00000051185	10.485	4.027	-1.380549484	1.02733E-12	1.34884E-13
ENSMUSG00000020279	3.700333333	1.420666667	-1.381087145	7.18425E-08	1.43535E-08
ENSMUSG00000079083	7.170666667	2.750333333	-1.382500774	4.07567E-10	6.58846E-11
ENSMUSG00000021068	64.228	24.63066667	-1.382746696	1.5727E-114	1.7699E-116
ENSMUSG00000032425	16.66366667	6.386	-1.383721428	1.37547E-17	1.30238E-18
ENSMUSG00000020877	6.962333333	2.668	-1.383812221	1.28119E-08	2.38616E-09
ENSMUSG00000020659	21.35566667	8.181666667	-1.38415227	6.40461E-36	2.88752E-37
ENSMUSG00000061882	0.625666667	0.239333333	-1.386372901	0.017352926	0.007367911
ENSMUSG00000021123	4.129666667	1.579333333	-1.386709639	3.01637E-05	8.13655E-06
ENSMUSG00000043510	16.39766667	6.246666667	-1.392332086	7.92211E-05	2.27014E-05
ENSMUSG00000022244	3.230666667	1.229333333	-1.393955749	4.35073E-06	1.05753E-06
ENSMUSG00000044952	1.222333333	0.465	-1.394335144	0.001885544	0.000667243
ENSMUSG00000031822	54.89433333	20.861	-1.395848912	7.51071E-30	4.03841E-31
ENSMUSG00000030060	24.48433333	9.279	-1.399817675	2.80281E-18	2.55261E-19
ENSMUSG00000025505	6.145666667	2.323	-1.403580365	8.661E-07	1.9476E-07
ENSMUSG00000028188	2.716333333	1.026666667	-1.40369268	0.00162743	0.000568894
ENSMUSG00000029291	10.88166667	4.108333333	-1.405274395	3.03803E-22	2.2371E-23
ENSMUSG00000023883	113.839	42.93	-1.406936816	2.21864E-49	6.78155E-51
ENSMUSG00000026107	26.04666667	9.822333333	-1.406961067	3.2241E-25	2.08519E-26
ENSMUSG00000028439	7.578666667	2.853666667	-1.409127229	2.45394E-16	2.50594E-17
ENSMUSG00000054499	10.355	3.895	-1.41063232	4.59448E-09	8.1708E-10
ENSMUSG00000042524	80.48966667	30.16933333	-1.415720764	1.51582E-85	2.53777E-87
ENSMUSG00000004562	9.2	3.442666667	-1.418107361	1.5525E-17	1.47323E-18
ENSMUSG00000019986	10.952	4.097333333	-1.41843718	4.71457E-25	3.06226E-26
ENSMUSG00000045409	15.95533333	5.967333333	-1.418880476	2.46506E-25	1.57887E-26
ENSMUSG00000054920	55.418	20.726	-1.418912934	9.49575E-69	1.96577E-70
ENSMUSG00000035845	20.398	7.622	-1.420186191	6.03458E-23	4.31371E-24
ENSMUSG00000026313	9.287333333	3.469	-1.420744575	6.614E-12	9.23984E-13
ENSMUSG00000031865	117.057	43.68566667	-1.421979298	7.17395E-98	9.66826E-100
ENSMUSG00000039652	2.214	0.826	-1.422441535	4.03122E-09	7.1439E-10
ENSMUSG00000031995	2.252333333	0.839666667	-1.423531733	0.010851795	0.004420576

ENSMUSG00000031007	103.6226667	38.629	-1.42358338	1.92618E-82	3.31846E-84
ENSMUSG00000025978	2.676666667	0.997	-1.424772077	1.08091E-05	2.75202E-06
ENSMUSG00000029217	32.067	11.93166667	-1.426293811	1.30849E-26	7.95367E-28
ENSMUSG00000040913	13.396	4.983	-1.426715802	7.08915E-19	6.28888E-20
ENSMUSG00000094081	0.941333333	0.349333333	-1.430101372	0.001406312	0.000485151
ENSMUSG00000028849	23.28533333	8.627666667	-1.432379191	8.61576E-36	3.90238E-37
ENSMUSG00000033326	50.09833333	18.538	-1.434277004	1.19564E-80	2.1014E-82
ENSMUSG00000023952	51.89266667	19.16433333	-1.437106863	3.93354E-49	1.21599E-50
ENSMUSG00000023467	14.56966667	5.368666667	-1.440332133	9.98009E-13	1.30618E-13
ENSMUSG00000031066	35.04466667	12.89933333	-1.441898396	1.17298E-38	4.88912E-40
ENSMUSG00000034613	7.607333333	2.792	-1.44609188	7.85956E-19	7.016E-20
ENSMUSG00000003051	1.754333333	0.643666667	-1.446537231	0.02052076	0.00885809
ENSMUSG00000041096	20.99566667	7.681333333	-1.450662936	4.61646E-22	3.43469E-23
ENSMUSG00000031174	5.697666667	2.081333333	-1.452863185	1.6844E-11	2.42685E-12
ENSMUSG00000024646	40.98766667	14.95	-1.455044379	5.68368E-14	6.79514E-15
ENSMUSG00000021013	3.272666667	1.193333333	-1.455469579	1.05398E-05	2.67834E-06
ENSMUSG00000021904	0.772333333	0.281	-1.458653508	0.001374618	0.000473167
ENSMUSG00000032298	6.592333333	2.397333333	-1.459358672	1.00963E-07	2.04592E-08
ENSMUSG00000020227	63.12733333	22.934	-1.460776807	5.11626E-63	1.20487E-64
ENSMUSG00000037287	28.955	10.51566667	-1.461272181	2.21453E-62	5.24595E-64
ENSMUSG00000051224	5.414666667	1.966333333	-1.461364618	2.06254E-05	5.43324E-06
ENSMUSG00000051890	1.988	0.721666667	-1.461913233	0.000262956	8.08871E-05
ENSMUSG00000001911	10.70966667	3.886	-1.462555772	9.89156E-30	5.34605E-31
ENSMUSG00000022272	0.188333333	0.068333333	-1.462626958	0.03013852	0.013481404
ENSMUSG00000046962	19.47366667	7.060333333	-1.46371635	9.58616E-19	8.59725E-20
ENSMUSG00000024667	31.54166667	11.41633333	-1.46615953	1.91246E-24	1.27276E-25
ENSMUSG00000011148	18.97366667	6.863666667	-1.466947112	0.001877319	0.000663941
ENSMUSG00000043257	7.891666667	2.848333333	-1.470212029	5.22797E-13	6.71156E-14
ENSMUSG00000033107	53.243	19.21466667	-1.470383914	3.37E-35	1.5498E-36
ENSMUSG00000039477	24.165	8.713666667	-1.471567164	4.0321E-53	1.12042E-54
ENSMUSG00000001542	45.39733333	16.35366667	-1.472993416	2.1144E-57	5.53754E-59
ENSMUSG00000035967	85.89733333	30.941	-1.473093519	3.43739E-48	1.09605E-49

ENSMUSG00000024187	7.694333333	2.771	-1.47338962	1.19339E-08	2.21102E-09
ENSMUSG00000038954	15.94766667	5.740666667	-1.474055163	1.4734E-15	1.58037E-16
ENSMUSG00000042675	19.65033333	7.064666667	-1.475860389	3.68877E-09	6.52422E-10
ENSMUSG00000040502	6.676	2.391333333	-1.48116871	1.92424E-15	2.08131E-16
ENSMUSG00000020629	17.16133333	6.125666667	-1.486222877	4.06795E-12	5.5869E-13
ENSMUSG00000021572	5.137666667	1.831666667	-1.48795631	2.81443E-05	7.54825E-06
ENSMUSG00000024645	9.111	3.245	-1.489390931	1.05654E-12	1.3894E-13
ENSMUSG00000035504	9.307333333	3.312	-1.490665204	2.47639E-13	3.09828E-14
ENSMUSG00000063160	3.770333333	1.340333333	-1.492100242	2.87261E-07	6.1311E-08
ENSMUSG00000027175	2.062333333	0.732	-1.49436198	2.02695E-10	3.19215E-11
ENSMUSG00000072889	25.70433333	9.111	-1.496330281	1.05451E-42	3.88985E-44
ENSMUSG00000026484	72.43633333	25.67	-1.496630224	1.46804E-93	2.11104E-95
ENSMUSG00000033276	0.623666667	0.220666667	-1.498906436	0.001313604	0.00045034
ENSMUSG00000022114	4.407333333	1.558333333	-1.49990215	4.86964E-08	9.57013E-09
ENSMUSG00000052040	28.68933333	10.14233333	-1.500124849	6.44529E-64	1.49995E-65
ENSMUSG00000033857	6.000666667	2.121	-1.50037817	8.66518E-13	1.12987E-13
ENSMUSG00000017167	2.687333333	0.949333333	-1.501188637	9.83443E-09	1.80771E-09
ENSMUSG00000078864	2.579	0.908	-1.50604757	0.049477949	0.023324582
ENSMUSG00000057060	3.841333333	1.352333333	-1.506156358	4.38789E-07	9.54088E-08
ENSMUSG00000025026	167.3543333	58.803	-1.508944242	4.91041E-52	1.39177E-53
ENSMUSG00000049184	6.167666667	2.166	-1.509691554	2.07292E-06	4.86586E-07
ENSMUSG00000032051	31.557	11.047	-1.514305426	2.67999E-14	3.12029E-15
ENSMUSG00000075078	2.511333333	0.878	-1.516160688	0.000441733	0.000140606
ENSMUSG00000067629	10.83066667	3.782333333	-1.517773631	6.77662E-16	7.06144E-17
ENSMUSG00000023266	2.646	0.924	-1.517848305	0.030697491	0.013765357
ENSMUSG00000033467	10.542	3.679666667	-1.518501611	3.18269E-11	4.68946E-12
ENSMUSG00000038910	26.478	9.234	-1.519766516	1.78842E-43	6.39832E-45
ENSMUSG00000030499	1.844333333	0.642666667	-1.520956872	0.001620018	0.000565965
ENSMUSG00000052293	24.98833333	8.694333333	-1.523107367	1.18315E-11	1.67754E-12
ENSMUSG00000040274	92.73233333	32.22466667	-1.524907021	4.5634E-184	2.6946E-186
ENSMUSG00000018474	81.94666667	28.43933333	-1.52679762	1.58367E-33	7.58004E-35
ENSMUSG00000026456	61.987	21.50033333	-1.527606656	3.74039E-42	1.39794E-43

ENSMUSG00000004864	8.727666667	3.022666667	-1.529774112	5.99007E-08	1.18636E-08
ENSMUSG00000066687	25.59966667	8.829	-1.535803077	5.67163E-49	1.773E-50
ENSMUSG00000024975	41.651	14.34166667	-1.538138442	3.8992E-44	1.37061E-45
ENSMUSG00000024905	0.893	0.307	-1.54042152	0.032915886	0.014863991
ENSMUSG00000040613	3.668	1.258666667	-1.543097375	0.000152431	4.53747E-05
ENSMUSG00000028223	55.82333333	19.12366667	-1.545509109	1.48694E-74	2.9026E-76
ENSMUSG00000004044	9.029666667	3.088666667	-1.547688549	1.96635E-19	1.67334E-20
ENSMUSG00000105340	0.378333333	0.129	-1.552286826	0.021146427	0.009149007
ENSMUSG00000031767	14.97333333	5.104666667	-1.552506765	1.66253E-11	2.39303E-12
ENSMUSG00000006850	19.55866667	6.662333333	-1.553708581	9.0035E-20	7.56181E-21
ENSMUSG00000021140	9.951333333	3.388	-1.554455964	4.33659E-42	1.62377E-43
ENSMUSG00000008167	8.475666667	2.875666667	-1.559430395	6.67366E-12	9.32783E-13
ENSMUSG00000028494	50.76166667	17.19933333	-1.561386793	5.68539E-37	2.49217E-38
ENSMUSG00000028696	12.27566667	4.152333333	-1.563807209	8.70665E-19	7.79032E-20
ENSMUSG00000006464	2.047333333	0.692	-1.564902069	6.9843E-12	9.78142E-13
ENSMUSG00000073987	38.153	12.882	-1.566439905	5.50658E-15	6.16263E-16
ENSMUSG00000039081	4.614666667	1.555666667	-1.568693474	2.51469E-17	2.41599E-18
ENSMUSG00000001755	29.81866667	10.03633333	-1.570983457	1.36427E-37	5.87598E-39
ENSMUSG00000042275	25.39466667	8.546333333	-1.571148044	2.50596E-25	1.60681E-26
ENSMUSG00000059970	18.17466667	6.104	-1.574102037	2.90069E-23	2.03521E-24
ENSMUSG00000026923	2.14	0.718666667	-1.574216119	1.00258E-12	1.31425E-13
ENSMUSG00000037731	2.348666667	0.788	-1.575574439	1.66892E-05	4.3349E-06
ENSMUSG00000025939	38.59	12.92166667	-1.57843488	9.28197E-46	3.12086E-47
ENSMUSG00000031953	5.277	1.759666667	-1.584415818	3.12626E-15	3.43791E-16
ENSMUSG00000015312	6.399666667	2.131666667	-1.586014904	4.34101E-08	8.47093E-09
ENSMUSG00000034480	8.569666667	2.853333333	-1.586590793	9.00432E-23	6.48036E-24
ENSMUSG00000003500	44.34733333	14.76566667	-1.586600861	3.60736E-50	1.07256E-51
ENSMUSG00000071552	2.317	0.769333333	-1.590577321	0.032536292	0.014680321
ENSMUSG00000025351	382.136	126.691	-1.592772136	2.0559E-146	1.7995E-148
ENSMUSG00000039234	39.222	12.98066667	-1.595298623	1.04517E-59	2.59932E-61
ENSMUSG00000013611	3.058333333	1.012	-1.595536367	3.22607E-06	7.72061E-07
ENSMUSG00000039178	16.348	5.407666667	-1.596036018	8.28249E-28	4.78135E-29

ENSMUSG00000033557	24.13333333	7.981666667	-1.596265262	3.08187E-55	8.34964E-57
ENSMUSG00000056130	6.474666667	2.138666667	-1.59809428	2.65162E-22	1.94704E-23
ENSMUSG00000037221	16.41133333	5.42	-1.598327698	1.33891E-09	2.26019E-10
ENSMUSG00000006732	6.487333333	2.140333333	-1.599790072	1.21513E-08	2.25469E-09
ENSMUSG00000075254	5.218333333	1.718333333	-1.602579176	6.0728E-25	3.96134E-26
ENSMUSG00000030433	3.159666667	1.037	-1.607356473	0.000104973	3.05841E-05
ENSMUSG00000070424	1.188666667	0.389666667	-1.609031773	0.035067159	0.015940634
ENSMUSG00000041679	2.448333333	0.802333333	-1.609526349	0.000167452	5.01134E-05
ENSMUSG00000026017	5.608	1.834333333	-1.612230521	1.1954E-13	1.46404E-14
ENSMUSG00000024900	41.00533333	13.39766667	-1.613829802	4.04124E-75	7.72033E-77
ENSMUSG00000024352	9.046333333	2.950666667	-1.616292206	6.85566E-05	1.95168E-05
ENSMUSG00000033327	0.957666667	0.312333333	-1.61643704	5.56312E-08	1.09794E-08
ENSMUSG00000000078	47.12533333	15.346	-1.618640163	1.01187E-83	1.71515E-85
ENSMUSG00000022253	57.61266667	18.75933333	-1.618777478	2.22059E-73	4.39643E-75
ENSMUSG00000024856	66.26633333	21.536	-1.621525779	1.3186E-51	3.78312E-53
ENSMUSG00000020798	32.31666667	10.48833333	-1.623492955	8.0245E-46	2.68691E-47
ENSMUSG00000057069	20.99733333	6.812	-1.624055777	1.16656E-52	3.27399E-54
ENSMUSG00000021614	0.694	0.225	-1.625010661	4.10171E-05	1.12779E-05
ENSMUSG00000070737	10.668	3.456333333	-1.625975465	3.57346E-11	5.29254E-12
ENSMUSG00000024220	16.226	5.25	-1.627918066	8.95377E-26	5.61671E-27
ENSMUSG00000020732	26.03366667	8.422	-1.628143739	1.15812E-33	5.51907E-35
ENSMUSG00000040957	0.919666667	0.297333333	-1.629029841	0.005015617	0.001908339
ENSMUSG00000021127	18.00266667	5.812	-1.631104015	1.07842E-39	4.35263E-41
ENSMUSG00000062082	24.67633333	7.937333333	-1.636401741	0.048682789	0.022902386
ENSMUSG00000051166	29.282	9.413666667	-1.637185421	5.50897E-38	2.33447E-39
ENSMUSG00000025355	1.513	0.486333333	-1.637394605	0.002304599	0.000827863
ENSMUSG00000037108	14.68766667	4.718333333	-1.638255974	3.76894E-26	2.33808E-27
ENSMUSG00000059708	1.686	0.541	-1.639904037	1.86124E-13	2.31054E-14
ENSMUSG00000068758	25.92333333	8.31	-1.641330856	7.01922E-22	5.26624E-23
ENSMUSG00000005917	4.231333333	1.350666667	-1.647440668	6.0694E-07	1.34332E-07
ENSMUSG00000006463	17.68033333	5.639666667	-1.648463675	1.01029E-29	5.47428E-31
ENSMUSG00000036599	26.66533333	8.502333333	-1.649034637	5.45956E-38	2.30974E-39

ENSMUSG00000003863	1.517333333	0.479333333	-1.662436882	3.38721E-07	7.27798E-08
ENSMUSG00000020576	19.43066667	6.129666667	-1.664454874	5.99047E-75	1.16106E-76
ENSMUSG00000021236	23.289	7.339666667	-1.665862244	2.38188E-64	5.49347E-66
ENSMUSG00000074527	18.92033333	5.958333333	-1.666956765	0.009989141	0.004040068
ENSMUSG00000047227	2.558333333	0.804666667	-1.668741074	0.000667868	0.000218417
ENSMUSG00000020964	58.55466667	18.408	-1.669451266	8.43197E-95	1.18323E-96
ENSMUSG00000031727	1.296	0.407	-1.670965019	9.98005E-05	2.8973E-05
ENSMUSG00000001506	0.379333333	0.119	-1.672504578	0.017254541	0.007324939
ENSMUSG00000034579	1.102666667	0.345666667	-1.67354334	0.002576312	0.000931911
ENSMUSG00000030200	3.640333333	1.139	-1.676302812	0.000248771	7.62472E-05
ENSMUSG00000026411	37.788	11.76933333	-1.682895561	8.71597E-42	3.30595E-43
ENSMUSG00000034949	1.230333333	0.382333333	-1.686146346	6.81005E-07	1.51576E-07
ENSMUSG00000029512	11.292	3.496	-1.691523944	8.23193E-36	3.71709E-37
ENSMUSG00000055835	12.02633333	3.721333333	-1.692305323	4.8893E-13	6.2632E-14
ENSMUSG00000021240	7.588666667	2.347	-1.693028584	6.06673E-12	8.43316E-13
ENSMUSG00000020571	438.4896667	134.8886667	-1.700773707	3.6197E-183	2.1876E-185
ENSMUSG00000028756	13.79266667	4.238666667	-1.702218993	1.62602E-25	1.02678E-26
ENSMUSG00000036452	23.197	7.128333333	-1.702301531	4.13725E-22	3.06665E-23
ENSMUSG00000078653	1.535333333	0.471	-1.704752945	0.000421417	0.000133612
ENSMUSG00000078866	22.92066667	7.023333333	-1.706421193	2.15894E-25	1.37231E-26
ENSMUSG00000035329	23.697	7.239	-1.710842107	2.23012E-41	8.58277E-43
ENSMUSG00000032489	1.425666667	0.435	-1.7125494	0.000344616	0.000107874
ENSMUSG00000046179	80.21266667	24.46433333	-1.713150107	1.07E-148	9.2913E-151
ENSMUSG00000039166	4.451666667	1.357333333	-1.713570509	0.001429896	0.000494181
ENSMUSG00000026003	90.53533333	27.592	-1.714230911	1.5941E-106	1.9491E-108
ENSMUSG00000079427	8.659333333	2.637333333	-1.715176033	3.48202E-07	7.49377E-08
ENSMUSG00000074794	8.189666667	2.492666667	-1.716114762	7.27371E-32	3.67854E-33
ENSMUSG00000026670	51.87166667	15.77833333	-1.717001904	1.81101E-76	3.39683E-78
ENSMUSG00000036478	12.15066667	3.682666667	-1.722212748	4.93408E-20	4.07889E-21
ENSMUSG00000021027	43.79333333	13.261	-1.723521693	9.8335E-129	9.5637E-131
ENSMUSG00000026024	18.29766667	5.537666667	-1.724309566	6.6232E-80	1.18707E-81
ENSMUSG00000042203	26.002	7.836666667	-1.730310557	8.05003E-62	1.93491E-63

ENSMUSG00000022637	44.24366667	13.31833333	-1.732057399	1.23E-108	1.4782E-110
ENSMUSG00000034801	38.85566667	11.68066667	-1.734002398	1.5352E-111	1.7917E-113
ENSMUSG00000070733	37.118	11.07733333	-1.744508356	7.8687E-109	9.3473E-111
ENSMUSG00000026728	183.41	54.70566667	-1.745310114	0.006393453	0.002481877
ENSMUSG00000030104	73.333	21.81566667	-1.749097998	4.4071E-124	4.6229E-126
ENSMUSG00000030103	16.08066667	4.783333333	-1.749238982	3.89676E-18	3.59492E-19
ENSMUSG00000033318	3.272	0.971666667	-1.751639366	1.59853E-05	4.14097E-06
ENSMUSG00000026604	1.833	0.543333333	-1.754297322	6.81187E-21	5.37568E-22
ENSMUSG00000021271	3.682333333	1.091	-1.754969127	8.90983E-10	1.48177E-10
ENSMUSG00000037447	14.13166667	4.167	-1.76185062	4.26873E-11	6.3727E-12
ENSMUSG00000034793	36.70333333	10.817	-1.762610656	1.27448E-33	6.09128E-35
ENSMUSG00000081534	84.07966667	24.702	-1.767129095	1.11918E-94	1.57828E-96
ENSMUSG00000021959	4.064333333	1.192666667	-1.768827842	1.2444E-11	1.7713E-12
ENSMUSG00000036402	171.5086667	50.102	-1.77534138	6.53677E-48	2.0934E-49
ENSMUSG00000032965	20.895	6.089	-1.778880541	0.001117764	0.000379628
ENSMUSG00000034845	0.892	0.259	-1.784091612	0.016282981	0.006870637
ENSMUSG00000074657	0.919666667	0.267	-1.784271309	2.62351E-06	6.22571E-07
ENSMUSG00000042320	1.560666667	0.451666667	-1.788832083	1.79315E-05	4.68002E-06
ENSMUSG00000024334	30.25166667	8.753666667	-1.789055274	1.38807E-27	8.13845E-29
ENSMUSG00000026004	8.197666667	2.368666667	-1.79113814	7.15333E-14	8.59195E-15
ENSMUSG00000046982	16.54166667	4.771	-1.79374101	1.53675E-47	4.98548E-49
ENSMUSG00000053226	1.708	0.491666667	-1.796555521	0.000585085	0.000189406
ENSMUSG00000004328	1.962333333	0.564666667	-1.797098753	1.09939E-09	1.84976E-10
ENSMUSG00000022769	105.8836667	30.431	-1.798866412	4.75089E-49	1.48187E-50
ENSMUSG00000031309	68.90066667	19.725	-1.804492642	1.8047E-102	2.2942E-104
ENSMUSG00000054277	33.093	9.424666667	-1.812012584	1.13315E-67	2.42453E-69
ENSMUSG00000045102	0.797333333	0.227	-1.812490686	0.001763547	0.000620028
ENSMUSG00000006567	0.701666667	0.199666667	-1.813192325	2.11764E-05	5.59016E-06
ENSMUSG00000034614	5.628	1.601	-1.813649021	3.97787E-06	9.62481E-07
ENSMUSG00000029060	16.74933333	4.764333333	-1.813757415	1.5909E-33	7.62571E-35
ENSMUSG00000038777	1.974333333	0.56	-1.817866853	0.004254019	0.001599062
ENSMUSG00000039153	6.178666667	1.752333333	-1.818018309	9.71709E-23	7.00684E-24

ENSMUSG00000025931	9.655333333	2.737333333	-1.81855494	1.8311E-41	7.00895E-43
ENSMUSG00000037463	0.947666667	0.268333333	-1.820353411	0.01432881	0.005982365
ENSMUSG00000045414	27.838	7.871333333	-1.822375624	1.69097E-48	5.3331E-50
ENSMUSG00000034591	0.954666667	0.269	-1.827390914	0.007006711	0.002745248
ENSMUSG00000031628	341.411	96.117	-1.828646017	2.14011E-18	1.94164E-19
ENSMUSG00000057858	102.7256667	28.907	-1.829305896	4.10877E-86	6.79324E-88
ENSMUSG00000032607	1.285333333	0.361333333	-1.830740295	0.000966643	0.000324677
ENSMUSG00000004952	4.304	1.207666667	-1.833455773	3.52147E-10	5.65343E-11
ENSMUSG00000036181	31.55333333	8.851	-1.833880054	3.32542E-45	1.1412E-46
ENSMUSG00000031586	41.49166667	11.61966667	-1.836252927	3.33362E-15	3.67225E-16
ENSMUSG00000035513	4.958666667	1.380333333	-1.844935545	8.6637E-07	1.94881E-07
ENSMUSG00000041143	5.721666667	1.591666667	-1.845897219	2.12886E-07	4.46034E-08
ENSMUSG00000034818	0.293666667	0.081666667	-1.84636027	0.022237006	0.009672087
ENSMUSG00000029201	61.88766667	17.189	-1.848166313	2.08755E-90	3.14691E-92
ENSMUSG00000003363	23.238	6.451333333	-1.848816641	2.76635E-41	1.06849E-42
ENSMUSG00000031751	85.87866667	23.65866667	-1.859931023	1.4009E-126	1.4209E-128
ENSMUSG00000053693	1.426333333	0.392666667	-1.86093414	8.54502E-07	1.92033E-07
ENSMUSG00000030203	8.735	2.398666667	-1.864575017	9.1708E-31	4.76538E-32
ENSMUSG00000052565	0.313333333	0.086	-1.865289691	0.003379457	0.001247312
ENSMUSG00000036537	4.579666667	1.254333333	-1.868321806	5.08727E-07	1.11393E-07
ENSMUSG00000030559	43.25666667	11.828	-1.870716348	1.21343E-50	3.57413E-52
ENSMUSG00000035126	0.834666667	0.228	-1.872166332	0.002483261	0.000895492
ENSMUSG00000032115	211.0846667	57.642	-1.872629486	5.82064E-80	1.03918E-81
ENSMUSG00000024206	9.731666667	2.656666667	-1.873069682	3.13847E-32	1.56978E-33
ENSMUSG00000001420	1.34	0.365666667	-1.873631976	0.016707749	0.0070696
ENSMUSG00000052085	174.074	47.398	-1.876802646	7.1954E-213	3.549E-215
ENSMUSG00000021665	79.71533333	21.634	-1.881556819	4.60353E-67	1.00098E-68
ENSMUSG00000002204	33.55433333	9.102666667	-1.882137937	1.35417E-30	7.07422E-32
ENSMUSG00000035964	3.103666667	0.841333333	-1.883224211	4.17414E-05	1.14887E-05
ENSMUSG00000023272	112.7046667	30.52766667	-1.884358024	5.19195E-62	1.23352E-63
ENSMUSG00000045780	6.745333333	1.825666667	-1.885466359	5.10083E-29	2.84895E-30
ENSMUSG00000029254	2.818333333	0.759666667	-1.89140383	0.001023621	0.000345308

ENSMUSG00000025579	89.29266667	24.061	-1.891845092	1.3343E-154	1.0382E-156
ENSMUSG00000034121	10.19866667	2.746	-1.892977022	2.72969E-18	2.48412E-19
ENSMUSG00000118669	1.176666667	0.316666667	-1.893668765	0.000734541	0.000241615
ENSMUSG00000028528	67.208	18.08033333	-1.894211696	2.7863E-154	2.1872E-156
ENSMUSG00000028669	62.02833333	16.66233333	-1.896336916	2.88279E-64	6.66876E-66
ENSMUSG00000053604	39.454	10.563	-1.901151941	7.70206E-37	3.39222E-38
ENSMUSG00000045257	3.504666667	0.934	-1.907782779	0.001076863	0.000364615
ENSMUSG00000076435	11.243	2.996	-1.907917516	3.24629E-23	2.28897E-24
ENSMUSG00000024114	3.727333333	0.992666667	-1.908762591	1.24436E-06	2.85545E-07
ENSMUSG00000070604	0.965	0.256666667	-1.910632997	0.045555134	0.0212348
ENSMUSG00000033545	31.65566667	8.411	-1.912114538	2.87407E-18	2.6215E-19
ENSMUSG00000054843	14.61733333	3.878666667	-1.914047441	3.47951E-67	7.51738E-69
ENSMUSG00000047446	41.31233333	10.94166667	-1.916740036	1.59926E-66	3.52181E-68
ENSMUSG00000015016	17.41966667	4.610666667	-1.917669744	2.09996E-39	8.56323E-41
ENSMUSG00000030731	0.628666667	0.166333333	-1.918217955	0.010406912	0.004224216
ENSMUSG00000044328	16.467	4.350666667	-1.920269353	2.3187E-26	1.42392E-27
ENSMUSG00000028521	17.75966667	4.679666667	-1.924126829	5.82462E-39	2.41158E-40
ENSMUSG00000054469	41.81966667	10.99033333	-1.927946419	2.54747E-47	8.29983E-49
ENSMUSG00000023828	31.89233333	8.360333333	-1.931577283	0.001835351	0.000647441
ENSMUSG00000036955	35.277	9.243333333	-1.932242762	3.61822E-73	7.18869E-75
ENSMUSG00000025197	8.309666667	2.176333333	-1.932891065	6.31409E-08	1.25229E-08
ENSMUSG00000020057	0.707666667	0.185333333	-1.932947583	0.00439754	0.001656371
ENSMUSG00000029313	60.55966667	15.74233333	-1.943707871	5.4787E-127	5.4806E-129
ENSMUSG00000027378	5.524	1.435666667	-1.943992497	2.42453E-11	3.54205E-12
ENSMUSG00000039242	74.354	19.32233333	-1.944141034	8.2599E-131	7.9185E-133
ENSMUSG00000022558	51.04866667	13.256	-1.945227772	8.5596E-163	6.303E-165
ENSMUSG00000024873	3.272333333	0.847	-1.94988584	5.15472E-06	1.26442E-06
ENSMUSG00000031770	182.884	47.315	-1.950559333	1.9298E-129	1.8635E-131
ENSMUSG00000019850	5.293666667	1.369	-1.951144907	3.14079E-19	2.71642E-20
ENSMUSG00000034584	0.257	0.066333333	-1.953962429	0.00834102	0.003323081
ENSMUSG00000032501	2.141333333	0.548333333	-1.965384309	7.19181E-11	1.09513E-11
ENSMUSG00000032418	15.114	3.869	-1.965852894	2.72778E-44	9.51263E-46

ENSMUSG00000022844	11.91233333	3.046	-1.967468182	7.3049E-27	4.39461E-28
ENSMUSG00000104346	0.418666667	0.106666667	-1.972692654	0.020515682	0.008853311
ENSMUSG00000039770	25.54733333	6.507666667	-1.972960448	1.87375E-43	6.7166E-45
ENSMUSG00000019796	0.354666667	0.090333333	-1.973133394	0.037818929	0.017316052
ENSMUSG00000000632	3.171333333	0.807	-1.974448946	0.022809307	0.009950847
ENSMUSG00000048721	0.952333333	0.242333333	-1.974473767	0.001494504	0.000518586
ENSMUSG00000024594	50.76466667	12.854	-1.981607321	6.41433E-89	1.00704E-90
ENSMUSG00000041189	7.217333333	1.819666667	-1.987791691	1.20511E-17	1.13688E-18
ENSMUSG00000027737	3.860666667	0.970666667	-1.991802141	0.002005879	0.000712892
ENSMUSG00000031391	7.743333333	1.945	-1.993184593	1.19742E-06	2.73921E-07
ENSMUSG00000067851	121.1403333	30.30466667	-1.999067407	6.3431E-133	5.9928E-135
ENSMUSG00000099583	0.79	0.196	-2.010998999	0.033917808	0.015350783
ENSMUSG00000025278	64.87566667	16.081	-2.012320335	1.9043E-204	9.9215E-207
ENSMUSG00000007216	1.451333333	0.359666667	-2.012646543	1.3942E-05	3.59712E-06
ENSMUSG00000023045	14.65566667	3.63	-2.013417142	3.32805E-39	1.36405E-40
ENSMUSG00000018740	2.176333333	0.538666667	-2.014434843	1.94165E-05	5.0959E-06
ENSMUSG00000052684	10.835	2.678333333	-2.016291725	8.09774E-17	8.06117E-18
ENSMUSG00000064220	4.038333333	0.995333333	-2.020508334	0.040682083	0.018745694
ENSMUSG00000089901	4.307333333	1.057666667	-2.025909954	3.15154E-13	3.97583E-14
ENSMUSG00000031762	221.6886667	54.43266667	-2.025990397	1.31163E-06	3.01506E-07
ENSMUSG00000028680	16.98	4.159666667	-2.029296631	1.1671E-27	6.7956E-29
ENSMUSG00000034177	3.252333333	0.795666667	-2.031239062	1.89511E-09	3.25046E-10
ENSMUSG00000030156	23.31833333	5.682333333	-2.036909307	0.046361412	0.021694371
ENSMUSG00000074227	57.72566667	14.06133333	-2.037479528	4.30984E-53	1.20059E-54
ENSMUSG00000029769	4.992333333	1.216	-2.037571036	1.6143E-13	1.99166E-14
ENSMUSG00000025407	0.747666667	0.181666667	-2.041101485	0.009107823	0.003659579
ENSMUSG00000044566	1.796333333	0.436333333	-2.041552489	1.64512E-08	3.09481E-09
ENSMUSG00000050199	8.13	1.972666667	-2.043108156	2.89019E-43	1.04806E-44
ENSMUSG00000004187	0.419666667	0.101666667	-2.045397135	0.011067443	0.004515394
ENSMUSG00000073600	4.572	1.105666667	-2.047908892	3.63474E-18	3.32796E-19
ENSMUSG00000068220	495.6626667	119.127	-2.056858164	0.004176543	0.001568199
ENSMUSG00000078851	2.543333333	0.61	-2.059839409	6.44282E-06	1.59694E-06

ENSMUSG00000055447	336.1433333	80.58366667	-2.06051718	1.7404E-55	4.67895E-57
ENSMUSG00000020669	5.114	1.225	-2.061670411	1.64566E-09	2.8066E-10
ENSMUSG00000078716	0.593333333	0.142	-2.062951906	0.000569699	0.000184108
ENSMUSG00000047407	46.51066667	11.09366667	-2.067825337	3.29062E-65	7.43444E-67
ENSMUSG00000040022	6.516	1.550333333	-2.071408165	6.33054E-34	2.99046E-35
ENSMUSG00000048478	1.493	0.355	-2.072323236	7.77632E-05	2.22728E-05
ENSMUSG00000025823	218.9806667	51.98066667	-2.074756461	2.8921E-249	1.1251E-251
ENSMUSG00000024063	70.52033333	16.64466667	-2.082979315	1.0609E-163	7.5913E-166
ENSMUSG00000031925	16.271	3.839666667	-2.083249944	6.81722E-59	1.72858E-60
ENSMUSG00000032554	90.54333333	21.365	-2.083359103	4.13569E-58	1.06876E-59
ENSMUSG00000038352	0.627333333	0.147666667	-2.086888024	0.032046963	0.014426142
ENSMUSG00000024807	81.82866667	19.23766667	-2.088672519	1.6734E-115	1.8716E-117
ENSMUSG00000028542	15.08833333	3.541	-2.091204705	9.06936E-38	3.87472E-39
ENSMUSG00000063873	1.463666667	0.342333333	-2.096113353	1.05719E-07	2.14596E-08
ENSMUSG00000020653	5.375333333	1.257	-2.096369572	1.03787E-21	7.85885E-23
ENSMUSG00000056234	279.0896667	65.21033333	-2.09755621	4.35092E-08	8.50235E-09
ENSMUSG00000030365	37.10066667	8.660333333	-2.098950651	3.76412E-39	1.54539E-40
ENSMUSG00000045312	0.582666667	0.135666667	-2.102604485	0.029835423	0.013312464
ENSMUSG00000056888	68.40866667	15.901	-2.105061613	0.045971373	0.021472265
ENSMUSG00000000142	2.912	0.673	-2.113331946	1.31151E-17	1.23908E-18
ENSMUSG00000021109	138.423	31.86433333	-2.119069295	9.0458E-240	3.6447E-242
ENSMUSG00000118607	0.825	0.189333333	-2.12346569	0.002408086	0.000867212
ENSMUSG00000044072	0.315333333	0.071666667	-2.137503524	0.000248178	7.60308E-05
ENSMUSG00000021007	5.243333333	1.191333333	-2.137907131	5.2203E-11	7.86218E-12
ENSMUSG00000031162	61.649	13.92233333	-2.146676471	4.00571E-90	6.12196E-92
ENSMUSG00000033022	2.250333333	0.506666667	-2.151029895	4.30043E-05	1.18691E-05
ENSMUSG00000028420	7.873	1.771333333	-2.152077751	4.84909E-25	3.153E-26
ENSMUSG00000034714	4.995	1.121	-2.1556984	1.58389E-15	1.70658E-16
ENSMUSG00000039982	8.693	1.95	-2.156380021	1.31521E-20	1.05236E-21
ENSMUSG00000036257	73.48533333	16.48	-2.156740094	7.0097E-127	7.0609E-129
ENSMUSG00000096370	2.69	0.602333333	-2.158972167	0.024380931	0.010694351
ENSMUSG00000026864	1782.343	396.569	-2.16813119	1.6795E-153	1.3417E-155

ENSMUSG00000033590	0.481666667	0.107	-2.17042429	0.000143073	4.24696E-05
ENSMUSG00000015837	410.896	91.15533333	-2.172374313	3.5569E-154	2.8169E-156
ENSMUSG00000029314	1.070333333	0.237333333	-2.173073519	0.000457937	0.000146114
ENSMUSG00000054855	11.25933333	2.496	-2.173431568	2.55673E-21	1.9715E-22
ENSMUSG00000021257	7.871	1.730333333	-2.185496953	2.78505E-41	1.07765E-42
ENSMUSG00000024644	36.077	7.836	-2.202890073	6.37908E-61	1.5643E-62
ENSMUSG00000071637	16.21	3.504333333	-2.209672175	2.55939E-58	6.56072E-60
ENSMUSG00000030674	0.952666667	0.205333333	-2.21400366	0.003386516	0.001250153
ENSMUSG00000060131	75.86466667	16.31266667	-2.217435477	5.23567E-08	1.03113E-08
ENSMUSG00000093769	0.949	0.204	-2.217838935	0.004423374	0.001667331
ENSMUSG00000051495	57.82133333	12.405	-2.220680142	7.33627E-73	1.46267E-74
ENSMUSG00000039577	1.627333333	0.346	-2.233665851	3.0594E-11	4.4993E-12
ENSMUSG00000096446	0.549666667	0.116	-2.244432188	0.032266698	0.014538507
ENSMUSG00000067242	0.847	0.178666667	-2.24509147	0.038358902	0.017579276
ENSMUSG00000041141	0.994666667	0.209666667	-2.246115613	1.1138E-05	2.84118E-06
ENSMUSG00000039474	3.378	0.708333333	-2.253668988	1.16254E-15	1.23643E-16
ENSMUSG00000033486	0.578333333	0.121	-2.256894209	0.010669985	0.004337669
ENSMUSG00000021226	13.62033333	2.842	-2.260783551	5.05728E-40	2.02362E-41
ENSMUSG00000022995	7.952666667	1.658	-2.261994695	3.17342E-33	1.54096E-34
ENSMUSG00000071076	72.292	14.966	-2.272147324	3.05677E-58	7.85692E-60
ENSMUSG00000025420	1.161	0.240333333	-2.272259308	3.10778E-05	8.40041E-06
ENSMUSG00000032353	104.7533333	21.66533333	-2.273535814	1.9431E-128	1.9033E-130
ENSMUSG00000050860	13.78066667	2.835666667	-2.280885824	6.55785E-19	5.79478E-20
ENSMUSG00000042616	1.654666667	0.340333333	-2.281520249	1.02199E-05	2.59279E-06
ENSMUSG00000029456	6.076666667	1.248333333	-2.283276938	5.10083E-29	2.84626E-30
ENSMUSG00000026638	0.34	0.069666667	-2.286994305	0.0115084	0.004707291
ENSMUSG00000026655	239.9913333	48.88866667	-2.295410343	0.001246185	0.000426534
ENSMUSG00000050357	0.867333333	0.176333333	-2.298281335	1.34079E-06	3.08582E-07
ENSMUSG00000038213	5.648666667	1.138333333	-2.310987291	6.58895E-21	5.1906E-22
ENSMUSG00000071648	6.805	1.362333333	-2.320515419	3.04967E-14	3.57402E-15
ENSMUSG00000038074	3.364666667	0.673	-2.321785176	2.75617E-14	3.21856E-15
ENSMUSG00000049892	0.599333333	0.119666667	-2.324337272	0.042457055	0.019655006

ENSMUSG00000000730	4.095333333	0.816333333	-2.326750609	2.25579E-06	5.31706E-07
ENSMUSG00000020077	928.756	184.8316667	-2.329087675	1.58423E-09	2.69632E-10
ENSMUSG00000027843	10.441	2.065	-2.338046208	5.97512E-15	6.69944E-16
ENSMUSG00000069307	0.604666667	0.119333333	-2.341142963	0.048624466	0.022868193
ENSMUSG00000036644	104.9926667	20.706	-2.34216778	9.7882E-109	1.1696E-110
ENSMUSG00000027173	13.21033333	2.596666667	-2.346934138	1.63194E-20	1.31961E-21
ENSMUSG00000040998	1.315333333	0.257666667	-2.351850637	2.39669E-07	5.05645E-08
ENSMUSG00000017417	1.609	0.315	-2.352740592	0.000524967	0.00016885
ENSMUSG00000040488	14.11333333	2.731333333	-2.369381471	7.72792E-65	1.76623E-66
ENSMUSG00000032009	3.341	0.645	-2.372908917	4.86675E-24	3.30276E-25
ENSMUSG00000030124	5.917	1.14	-2.375832071	4.77767E-19	4.18191E-20
ENSMUSG00000042042	182.3963333	34.91533333	-2.385144076	7.9025E-293	2.4155E-295
ENSMUSG00000044968	2.474666667	0.472333333	-2.389356952	1.90501E-13	2.36621E-14
ENSMUSG00000028100	1.543	0.294333333	-2.39021522	0.00271652	0.000987723
ENSMUSG00000054733	9.476333333	1.8	-2.39633204	1.9833E-14	2.28986E-15
ENSMUSG00000002032	1.358333333	0.256333333	-2.405744556	0.010022581	0.004055868
ENSMUSG00000049090	38.606	7.263333333	-2.410121387	3.35411E-68	7.08336E-70
ENSMUSG00000022780	0.333333333	0.062666667	-2.411195433	0.003718459	0.001383024
ENSMUSG00000031877	183.3753333	34.11433333	-2.42634966	0	0
ENSMUSG00000007021	1.407333333	0.261666667	-2.42716202	6.05702E-05	1.70665E-05
ENSMUSG00000040013	1.372	0.254333333	-2.43148802	0.000834263	0.000277431
ENSMUSG00000021000	206.2533333	38.107	-2.436289495	0	0
ENSMUSG00000039308	57.503	10.59266667	-2.440571397	1.1169E-151	9.3105E-154
ENSMUSG00000054892	1.453666667	0.267	-2.444784843	1.45717E-05	3.76466E-06
ENSMUSG00000020191	1.787	0.326	-2.454595765	2.70185E-05	7.22808E-06
ENSMUSG00000020256	1.165	0.212333333	-2.455927178	3.66251E-09	6.47014E-10
ENSMUSG00000032715	28.382	5.167666667	-2.457391337	1.22506E-40	4.82535E-42
ENSMUSG00000032380	1.824666667	0.331	-2.462729812	1.04122E-05	2.64301E-06
ENSMUSG00000107705	17.50466667	3.138	-2.479822332	8.59423E-69	1.77318E-70
ENSMUSG00000032905	52.355	9.373666667	-2.481641926	1.51775E-77	2.81514E-79
ENSMUSG00000026544	4.459	0.789333333	-2.498013619	2.13697E-09	3.68161E-10
ENSMUSG00000030413	12.244	2.163666667	-2.500524789	1.09792E-09	1.84653E-10

ENSMUSG00000045502	1.093666667	0.193	-2.502500341	0.00610807	0.002363032
ENSMUSG00000000915	20.37766667	3.590333333	-2.504799168	1.1453E-151	9.6267E-154
ENSMUSG00000010154	12.74633333	2.239	-2.509155861	1.43567E-49	4.33844E-51
ENSMUSG00000018796	58.71466667	10.28466667	-2.513225891	2.3054E-207	1.1691E-209
ENSMUSG00000040231	1.118333333	0.195333333	-2.517340197	0.012763942	0.005262521
ENSMUSG00000032890	1.967333333	0.343	-2.519960939	9.50347E-14	1.15402E-14
ENSMUSG00000036067	2.713	0.472666667	-2.520994016	0.000100843	2.93036E-05
ENSMUSG00000047394	3.330333333	0.577	-2.52902336	0.0007343	0.000241443
ENSMUSG00000020395	0.415666667	0.072	-2.529358248	0.000177962	5.34319E-05
ENSMUSG00000022952	104.8183333	18.09233333	-2.534440688	3.45038E-93	5.00958E-95
ENSMUSG00000040867	1.056	0.182	-2.536599479	5.40913E-05	1.51208E-05
ENSMUSG00000046727	7.261333333	1.244	-2.545247996	6.07668E-12	8.45121E-13
ENSMUSG00000023032	0.960666667	0.164	-2.550340115	1.78033E-07	3.69918E-08
ENSMUSG00000039824	15.06433333	2.540666667	-2.567857816	1.07644E-21	8.15835E-23
ENSMUSG00000021065	206.7893333	34.653	-2.577109609	2.4135E-102	3.1017E-104
ENSMUSG00000032198	0.358666667	0.06	-2.579609266	2.86689E-05	7.70546E-06
ENSMUSG00000028476	3.875	0.645666667	-2.585334857	1.27037E-29	6.92766E-31
ENSMUSG00000036330	0.268	0.044	-2.606657572	0.033099408	0.014959691
ENSMUSG00000041058	68.66533333	11.27033333	-2.607051732	0	0
ENSMUSG00000024480	259.8293333	42.61266667	-2.60821007	3.48706E-26	2.15837E-27
ENSMUSG00000041040	27.16566667	4.438333333	-2.613694524	3.95309E-88	6.31615E-90
ENSMUSG00000038742	3.173666667	0.515666667	-2.621639912	9.96271E-10	1.66587E-10
ENSMUSG00000014496	104.323	16.76566667	-2.637475506	2.3874E-183	1.4263E-185
ENSMUSG00000036902	12.77666667	2.042	-2.645456726	1.295E-88	2.05112E-90
ENSMUSG00000028182	0.654333333	0.104	-2.653442239	0.020942465	0.009057853
ENSMUSG00000033233	14.47566667	2.297333333	-2.655597686	4.26571E-44	1.50241E-45
ENSMUSG00000031012	23.86233333	3.785333333	-2.656242868	1.69239E-72	3.40947E-74
ENSMUSG00000026944	76.14033333	12.01333333	-2.664024375	0	0
ENSMUSG00000055172	14.24966667	2.242666667	-2.667641062	6.38984E-29	3.57778E-30
ENSMUSG00000036052	6.645	1.039666667	-2.676148148	4.89844E-22	3.65809E-23
ENSMUSG00000116780	2.355	0.367333333	-2.680565337	0.004047237	0.001515992
ENSMUSG00000026483	51.94133333	8.099333333	-2.681007981	3.4605E-261	1.2501E-263

ENSMUSG00000006457	1.314	0.204333333	-2.684968797	1.24985E-05	3.21253E-06
ENSMUSG00000026880	14.50833333	2.224666667	-2.705220706	0.000406053	0.000128402
ENSMUSG00000046694	0.267666667	0.041	-2.706741672	0.048325889	0.0227177
ENSMUSG00000030546	1.140666667	0.174666667	-2.707201043	0.00016045	4.79175E-05
ENSMUSG00000073591	1.378666667	0.209333333	-2.719399721	3.08499E-09	5.4049E-10
ENSMUSG00000066952	0.441333333	0.067	-2.719635716	0.000119364	3.50835E-05
ENSMUSG00000055717	12.86	1.944	-2.725790519	5.15067E-44	1.81767E-45
ENSMUSG00000067220	2.462666667	0.372	-2.726846839	7.74973E-06	1.94187E-06
ENSMUSG00000022994	0.194333333	0.029333333	-2.727920455	0.025697356	0.011310764
ENSMUSG00000096054	30.66633333	4.625333333	-2.729026434	1.21134E-14	1.38511E-15
ENSMUSG00000051228	0.150666667	0.022666667	-2.732716121	0.014258981	0.005948258
ENSMUSG00000068699	1.027666667	0.153666667	-2.741496234	2.06808E-10	3.2598E-11
ENSMUSG00000022824	13.501	2.008	-2.749235095	3.78592E-31	1.94096E-32
ENSMUSG00000056458	0.569333333	0.084666667	-2.749407573	0.045152078	0.021024965
ENSMUSG00000060002	10.604	1.574666667	-2.751490206	6.92535E-54	1.90513E-55
ENSMUSG00000062232	21.516	3.189333333	-2.7540831	2.3156E-218	1.0938E-220
ENSMUSG00000032436	378.9706667	56.14433333	-2.754873861	7.01441E-09	1.27278E-09
ENSMUSG00000054200	1.522	0.224333333	-2.76225245	2.53875E-05	6.76353E-06
ENSMUSG00000087385	1.406666667	0.206	-2.771564256	4.08824E-05	1.12352E-05
ENSMUSG00000040747	188.3593333	27.48966667	-2.776526203	0.035592796	0.016207765
ENSMUSG00000026656	80.24766667	11.61833333	-2.788056317	0.008358865	0.003331352
ENSMUSG00000054136	9.291333333	1.292	-2.846279572	2.12485E-22	1.55139E-23
ENSMUSG00000027009	130.2753333	17.91033333	-2.862699853	0	0
ENSMUSG00000053574	0.372333333	0.051	-2.868025628	0.007071258	0.002772994
ENSMUSG00000063455	0.131333333	0.017666667	-2.894131365	0.00146689	0.000508495
ENSMUSG00000034401	5.492333333	0.738	-2.895726465	9.10157E-25	6.00659E-26
ENSMUSG00000021298	1.003333333	0.134	-2.90449608	5.63178E-06	1.38731E-06
ENSMUSG00000044022	0.549	0.072333333	-2.924073607	2.99224E-05	8.06314E-06
ENSMUSG00000034353	22.12166667	2.903333333	-2.92967796	0.006544314	0.002547259
ENSMUSG00000020836	1.520333333	0.197333333	-2.945681089	9.62229E-07	2.17245E-07
ENSMUSG00000009739	0.957666667	0.124	-2.949183466	2.91119E-08	5.56757E-09
ENSMUSG00000028977	1.706666667	0.220666667	-2.951240688	1.61874E-22	1.17512E-23

ENSMUSG00000026764	0.268333333	0.034666667	-2.952405255	0.0002431	7.4357E-05
ENSMUSG00000051682	1.780333333	0.229666667	-2.954533996	3.704E-06	8.94158E-07
ENSMUSG00000025432	22.44833333	2.880333333	-2.962300651	2.038E-96	2.76075E-98
ENSMUSG00000034647	38.78266667	4.975666667	-2.962450262	3.0094E-146	2.6551E-148
ENSMUSG00000070323	0.495	0.063333333	-2.966391607	0.025133481	0.011048605
ENSMUSG00000028758	0.701333333	0.089	-2.978223058	6.98289E-05	1.98936E-05
ENSMUSG00000024008	1.145333333	0.144666667	-2.984963089	6.54805E-10	1.07807E-10
ENSMUSG00000033029	1.588333333	0.198333333	-3.001514641	0.00319934	0.001176166
ENSMUSG00000043019	66.91433333	8.273666667	-3.015716535	0	0
ENSMUSG00000041991	0.272333333	0.033666667	-3.015980785	1.88226E-05	4.92697E-06
ENSMUSG00000008129	1.050666667	0.129666667	-3.018425474	4.64342E-06	1.13416E-06
ENSMUSG00000052435	74.201	9.029333333	-3.038747252	1.51913E-06	3.51737E-07
ENSMUSG00000066442	4.700333333	0.568333333	-3.047953833	5.19611E-09	9.29488E-10
ENSMUSG00000036158	4.725333333	0.571	-3.048853452	6.71362E-30	3.60516E-31
ENSMUSG00000035863	33.15866667	3.983	-3.057458611	3.24984E-17	3.15841E-18
ENSMUSG00000030824	404.162	47.65466667	-3.084244279	0	0
ENSMUSG00000004552	4.954666667	0.580666667	-3.093005878	1.1937E-12	1.57806E-13
ENSMUSG00000000686	13.622	1.579	-3.108855461	9.12159E-62	2.19881E-63
ENSMUSG00000001552	8.554666667	0.959	-3.157108921	1.62556E-39	6.60612E-41
ENSMUSG00000061451	5.487	0.611	-3.166773291	5.58534E-29	3.12344E-30
ENSMUSG00000056215	0.761666667	0.084	-3.180698526	1.40829E-10	2.19144E-11
ENSMUSG00000042333	9.531333333	1.049666667	-3.182746789	3.55416E-08	6.8639E-09
ENSMUSG00000003617	0.709333333	0.077666667	-3.191096291	1.85463E-06	4.32897E-07
ENSMUSG00000114456	0.856333333	0.093333333	-3.197708158	0.00031862	9.90943E-05
ENSMUSG00000078234	0.236333333	0.025666667	-3.202855277	6.17612E-05	1.74364E-05
ENSMUSG00000003814	3052.664	330.2	-3.208656779	0	0
ENSMUSG00000037913	41.235	4.417666667	-3.222512938	1.27035E-08	2.36244E-09
ENSMUSG00000028558	0.332	0.035333333	-3.232081478	0.019015058	0.008142328
ENSMUSG00000030657	11.286	1.199666667	-3.233828748	4.28785E-10	6.93741E-11
ENSMUSG00000032221	57.06966667	6.026	-3.243451558	8.0509E-150	6.8792E-152
ENSMUSG00000000594	0.319666667	0.033666667	-3.247175522	0.000490678	0.000157071
ENSMUSG00000035064	57.18733333	6.020666667	-3.247700484	9.62107E-22	7.27846E-23

ENSMUSG00000043008	0.317333333	0.033333333	-3.250961574	0.00983237	0.003972564
ENSMUSG00000022123	3.253333333	0.341666667	-3.251257238	0.019519367	0.008390819
ENSMUSG00000038604	90.30833333	9.434333333	-3.258866641	7.6051E-29	4.29521E-30
ENSMUSG00000048652	4.643	0.483666667	-3.262972262	1.10572E-15	1.17447E-16
ENSMUSG00000022475	57.87933333	6.015333333	-3.266331715	0	0
ENSMUSG00000041329	1.545	0.159	-3.280508168	5.51417E-11	8.3201E-12
ENSMUSG00000051736	1.894333333	0.194666667	-3.282612441	0.00035017	0.000109758
ENSMUSG00000017950	0.766	0.078666667	-3.283520033	9.20851E-07	2.07391E-07
ENSMUSG00000029718	3.402333333	0.349	-3.28522555	1.48784E-12	1.98137E-13
ENSMUSG00000026576	0.294333333	0.03	-3.294416531	0.011612061	0.004756952
ENSMUSG00000039765	1.265333333	0.127666667	-3.309063695	1.22923E-07	2.51225E-08
ENSMUSG00000019301	4.136	0.414333333	-3.31937239	9.8041E-13	1.28138E-13
ENSMUSG00000036377	14.21433333	1.423	-3.32033887	2.9258E-102	3.7805E-104
ENSMUSG00000039286	10.11366667	0.995	-3.345465799	4.26152E-86	7.0754E-88
ENSMUSG00000046006	72.23533333	7.080666667	-3.350747586	1.53074E-12	2.0417E-13
ENSMUSG00000028266	196.547	19.18333333	-3.356949011	0	0
ENSMUSG00000014905	77.61766667	7.48	-3.375274888	7.9906E-260	2.942E-262
ENSMUSG00000004612	7.932666667	0.757666667	-3.388170744	1.5623E-17	1.48361E-18
ENSMUSG00000036587	23.25033333	2.214666667	-3.392089922	1.12483E-86	1.84411E-88
ENSMUSG00000027962	0.231333333	0.022	-3.394397733	0.005779204	0.002222554
ENSMUSG00000033963	0.371	0.034666667	-3.419798159	0.032320304	0.014569396
ENSMUSG00000029322	496.4316667	45.61166667	-3.444120353	7.38875E-07	1.65054E-07
ENSMUSG00000019027	0.065333333	0.006	-3.444784843	0.004450086	0.001678018
ENSMUSG00000022221	0.676666667	0.061333333	-3.463702056	0.003613698	0.001340117
ENSMUSG00000037940	31.51733333	2.853	-3.465593822	2.05477E-21	1.57873E-22
ENSMUSG00000047822	11.508	1.038666667	-3.469832488	5.89234E-20	4.89152E-21
ENSMUSG00000027122	577.254	52.05633333	-3.471060663	0	0
ENSMUSG00000041608	1.489666667	0.133333333	-3.48188014	1.70113E-10	2.66249E-11
ENSMUSG00000032420	0.233	0.020666667	-3.494952335	0.004074715	0.0015277
ENSMUSG00000030470	1.628	0.144333333	-3.49562427	0.001237692	0.000423197
ENSMUSG00000098678	0.286	0.025333333	-3.496906324	0.023690898	0.010361785
ENSMUSG00000037705	0.091666667	0.008	-3.518325308	0.019418437	0.008339061

ENSMUSG00000032323	281.6316667	24.30766667	-3.534326241	0	0
ENSMUSG00000026748	33.48966667	2.873333333	-3.542918743	8.5114E-177	5.4989E-179
ENSMUSG00000030142	42.72433333	3.659666667	-3.545273826	0.001765481	0.00062085
ENSMUSG00000000409	14.265	1.213333333	-3.555431892	1.873E-49	5.69902E-51
ENSMUSG00000029359	1.170666667	0.097333333	-3.588252571	0.000622572	0.00020232
ENSMUSG00000030643	1.713	0.142333333	-3.589179677	4.4317E-08	8.66945E-09
ENSMUSG00000091898	0.702333333	0.058333333	-3.589763487	0.04967155	0.023429651
ENSMUSG00000042700	119.3723333	9.853333333	-3.598712831	0	0
ENSMUSG00000074028	0.640333333	0.051333333	-3.640855263	0.000706274	0.000231434
ENSMUSG00000019947	3.517	0.281333333	-3.643992932	0.000326125	0.000101519
ENSMUSG00000039716	0.208666667	0.016666667	-3.646162657	0.01505419	0.006312406
ENSMUSG00000024842	0.531	0.042333333	-3.648845865	0.006457124	0.002511527
ENSMUSG00000032348	21.67666667	1.704	-3.669145682	4.10009E-54	1.12222E-55
ENSMUSG00000026482	1.768666667	0.139	-3.669505387	2.57237E-19	2.20872E-20
ENSMUSG00000047492	0.883	0.068	-3.698806786	1.18978E-11	1.68775E-12
ENSMUSG00000031760	3.449666667	0.262	-3.718818247	9.608E-06	2.42753E-06
ENSMUSG00000067813	0.657	0.049666667	-3.725543541	0.000770035	0.000254307
ENSMUSG00000000127	0.167666667	0.012666667	-3.726487076	0.038960609	0.017876681
ENSMUSG00000045004	1.765333333	0.133333333	-3.726831217	1.53521E-11	2.20443E-12
ENSMUSG00000041235	0.544	0.041	-3.729910837	3.57933E-05	9.76453E-06
ENSMUSG00000026649	2.194666667	0.165	-3.733463905	3.38597E-05	9.20174E-06
ENSMUSG00000076757	8.324	0.625333333	-3.734579638	7.33308E-19	6.51546E-20
ENSMUSG00000059495	0.174333333	0.013	-3.745264917	1.94907E-05	5.11673E-06
ENSMUSG00000057751	0.076	0.005666667	-3.745427173	0.01197591	0.00491682
ENSMUSG00000053914	1.270666667	0.093	-3.772211092	7.69458E-10	1.27486E-10
ENSMUSG00000025408	52.203	3.813	-3.775134282	1.92836E-79	3.46957E-81
ENSMUSG00000029470	32.02666667	2.329	-3.781491106	1.56428E-87	2.52111E-89
ENSMUSG00000026564	0.332333333	0.024	-3.791524693	0.0001746	5.23619E-05
ENSMUSG00000002033	4.215666667	0.304333333	-3.792036533	0.008591545	0.003436021
ENSMUSG00000053702	9.305666667	0.660666667	-3.816115048	2.77336E-05	7.42709E-06
ENSMUSG00000007480	10.87033333	0.761333333	-3.835724125	0.001688909	0.000592262
ENSMUSG00000027072	3.540333333	0.246333333	-3.845201432	1.70267E-08	3.20663E-09

ENSMUSG00000031997	15.018	1.042	-3.849265514	2.04958E-91	3.03272E-93
ENSMUSG00000029638	45.484	3.145333333	-3.854074312	3.691E-190	2.1282E-192
ENSMUSG00000054619	115.955	8.015	-3.8547187	1.3704E-169	9.4247E-172
ENSMUSG00000030303	15.878	1.096333333	-3.856270789	4.6016E-125	4.795E-127
ENSMUSG00000030134	1.481	0.102	-3.859930583	5.92715E-10	9.70496E-11
ENSMUSG00000030851	5.344333333	0.364	-3.875999638	3.99791E-15	4.42145E-16
ENSMUSG00000030157	4.915333333	0.334	-3.879369248	1.21978E-13	1.49475E-14
ENSMUSG00000037661	2.087333333	0.141666667	-3.881088762	2.32556E-11	3.391E-12
ENSMUSG00000054191	31.14966667	2.096	-3.893506103	4.8893E-111	5.7401E-113
ENSMUSG00000036390	89.99133333	5.938666667	-3.921575106	7.6484E-164	5.4195E-166
ENSMUSG00000031503	0.494	0.032	-3.948367232	7.83097E-08	1.57109E-08
ENSMUSG00000026018	3.768	0.243666667	-3.950818154	6.37071E-23	4.56283E-24
ENSMUSG00000040969	0.239	0.015333333	-3.962267353	0.000118426	3.47998E-05
ENSMUSG00000028763	0.143	0.009	-3.989946335	5.11102E-05	1.42448E-05
ENSMUSG00000048924	3.463666667	0.217	-3.996533148	6.42955E-19	5.66801E-20
ENSMUSG00000032584	1.729666667	0.108	-4.001390818	5.56076E-16	5.75584E-17
ENSMUSG00000053820	2.025	0.126	-4.006426269	0.003176831	0.00116745
ENSMUSG00000064147	77.82466667	4.809	-4.016418661	1.12359E-05	2.86771E-06
ENSMUSG00000023411	1.959333333	0.118666667	-4.045376213	3.76455E-19	3.27682E-20
ENSMUSG00000040187	1.623666667	0.098333333	-4.045431124	1.14901E-12	1.51498E-13
ENSMUSG00000089951	0.566333333	0.034	-4.058044795	0.049325071	0.023232214
ENSMUSG00000040165	31.83166667	1.877666667	-4.083449821	8.25928E-21	6.55234E-22
ENSMUSG00000051212	5.107666667	0.299333333	-4.092839526	0.02860785	0.012703118
ENSMUSG00000039337	0.698	0.039	-4.161681007	0.000101801	2.96032E-05
ENSMUSG00000028838	5.136333333	0.285	-4.171705008	9.27628E-54	2.56475E-55
ENSMUSG00000052922	0.216666667	0.012	-4.174370906	0.039321693	0.018050555
ENSMUSG00000079033	1.732666667	0.095666667	-4.178833992	2.86773E-06	6.82917E-07
ENSMUSG00000062713	1.331333333	0.071333333	-4.222151631	0.000901645	0.000301789
ENSMUSG00000026837	12.24633333	0.644333333	-4.248398816	4.97191E-06	1.21716E-06
ENSMUSG00000036995	0.082666667	0.004333333	-4.253756592	0.045857326	0.021410704
ENSMUSG00000019982	316.9016667	16.552	-4.258957792	6.08605E-15	6.82805E-16
ENSMUSG00000032850	0.167333333	0.008666667	-4.271103836	0.022363263	0.009737613

ENSMUSG00000035085	8.054333333	0.410333333	-4.294896919	4.45969E-16	4.59755E-17
ENSMUSG00000042286	3.488333333	0.177666667	-4.295292968	3.19645E-06	7.64084E-07
ENSMUSG00000047658	0.150666667	0.007666667	-4.296617006	0.042233822	0.019534059
ENSMUSG00000022636	0.476333333	0.023666667	-4.331043082	0.002159275	0.000771759
ENSMUSG00000024935	0.102	0.005	-4.350497247	0.033016623	0.014915394
ENSMUSG00000027073	302.7496667	14.837	-4.350854054	3.04392E-13	3.83794E-14
ENSMUSG00000073755	0.136666667	0.006666667	-4.357552005	0.038635519	0.017716781
ENSMUSG00000025746	14.76766667	0.718	-4.36231424	9.89564E-10	1.6526E-10
ENSMUSG00000025997	101.5636667	4.937	-4.36260593	8.26574E-10	1.37293E-10
ENSMUSG00000025425	0.195666667	0.009333333	-4.389861771	0.03018	0.013503951
ENSMUSG00000044734	108.7276667	5.154666667	-4.398696146	1.89023E-07	3.93803E-08
ENSMUSG00000024827	0.345333333	0.016333333	-4.402098444	0.000206735	6.25734E-05
ENSMUSG00000004988	1.859333333	0.086	-4.434304964	6.69854E-05	1.90184E-05
ENSMUSG00000021573	0.081	0.003666667	-4.465380885	0.027388875	0.012119982
ENSMUSG00000067455	0.604666667	0.027333333	-4.467406736	0.01694548	0.007180787
ENSMUSG00000051839	12.976	0.562	-4.529131784	5.6429E-59	1.42297E-60
ENSMUSG00000024681	397.6493333	16.99366667	-4.548427676	8.84286E-05	2.55058E-05
ENSMUSG00000100838	1.812333333	0.076	-4.575705099	0.000340409	0.000106391
ENSMUSG00000114694	0.535333333	0.022333333	-4.583166987	0.00253188	0.000913904
ENSMUSG00000035615	0.097333333	0.004	-4.604862058	0.015662716	0.00658933
ENSMUSG00000071714	389.6726667	15.91233333	-4.61404543	2.75429E-19	2.37449E-20
ENSMUSG00000072419	0.242333333	0.009666667	-4.647830559	0.014922136	0.006251852
ENSMUSG00000090164	0.680666667	0.027	-4.655917148	0.000721929	0.000237015
ENSMUSG00000018102	7.418333333	0.290333333	-4.675312972	7.91898E-22	5.9578E-23
ENSMUSG00000071713	438.0576667	17.09933333	-4.679108817	4.38491E-11	6.54919E-12
ENSMUSG00000028445	0.803	0.031333333	-4.679629827	0.000855823	0.000285254
ENSMUSG00000025934	0.369	0.014333333	-4.686174752	0.049714097	0.023463534
ENSMUSG00000042082	36.479	1.389666667	-4.714255405	1.55583E-15	1.6731E-16
ENSMUSG00000047671	0.501	0.019	-4.72073928	0.030618148	0.013723396
ENSMUSG00000028555	3.849666667	0.145	-4.730608726	1.02049E-22	7.37274E-24
ENSMUSG00000048126	2.368333333	0.089	-4.733923002	1.20262E-05	3.08075E-06
ENSMUSG00000020009	219.3943333	8.239333333	-4.734854843	0	0

ENSMUSG00000063129	2.308	0.082	-4.814875504	3.85873E-11	5.74186E-12
ENSMUSG00000045382	443.0433333	15.477	-4.839250056	2.9155E-11	4.2816E-12
ENSMUSG00000062826	0.221	0.007666667	-4.849303104	0.000610512	0.000198146
ENSMUSG00000036658	0.233	0.008	-4.864186145	0.001907805	0.000675783
ENSMUSG00000024424	12.91833333	0.440333333	-4.874680081	1.39633E-62	3.29803E-64
ENSMUSG00000031860	3.906	0.132666667	-4.879814114	2.56418E-12	3.46463E-13
ENSMUSG00000040732	9.965333333	0.333333333	-4.901880564	1.55566E-05	4.02667E-06
ENSMUSG00000047842	0.779333333	0.026	-4.905656996	1.50165E-10	2.34192E-11
ENSMUSG00000078872	27.482	0.855333333	-5.005856425	2.58563E-68	5.42453E-70
ENSMUSG00000026435	0.753333333	0.023333333	-5.01282404	1.28664E-07	2.63406E-08
ENSMUSG00000014329	1.201333333	0.036	-5.060495794	1.05164E-14	1.19593E-15
ENSMUSG00000009350	222.6543333	6.52	-5.093789915	1.33838E-08	2.50104E-09
ENSMUSG00000014599	64.927	1.896	-5.097787681	7.82166E-09	1.42523E-09
ENSMUSG00000029925	2.126333333	0.061333333	-5.115552607	2.09682E-10	3.30801E-11
ENSMUSG00000000782	0.267	0.007666667	-5.122096476	0.006414326	0.002490425
ENSMUSG00000027360	114.2503333	3.275	-5.124559658	4.10171E-05	1.12769E-05
ENSMUSG00000018168	1.616666667	0.046	-5.135244575	0.005139719	0.001959127
ENSMUSG00000058252	10.52733333	0.299333333	-5.136243281	1.72524E-07	3.57871E-08
ENSMUSG00000039851	8.844333333	0.25	-5.1447534	3.58043E-08	6.92705E-09
ENSMUSG00000027834	22.27333333	0.600333333	-5.213409896	5.3477E-163	3.9007E-165
ENSMUSG00000063683	7.798666667	0.209333333	-5.219353525	3.55316E-07	7.65923E-08
ENSMUSG00000031712	10.74933333	0.283666667	-5.243906746	9.60854E-29	5.45341E-30
ENSMUSG00000039735	1.12	0.029333333	-5.254813899	1.19124E-15	1.26862E-16
ENSMUSG00000002565	337.2646667	8.615333333	-5.290830757	3.73557E-08	7.24019E-09
ENSMUSG00000078498	0.262	0.006666667	-5.296457407	0.001837807	0.000648563
ENSMUSG00000040229	12.739	0.323666667	-5.298599427	4.03257E-61	9.86081E-63
ENSMUSG00000030228	124.9216667	3.152	-5.308612378	3.00692E-16	3.08525E-17
ENSMUSG00000030154	4.986333333	0.123333333	-5.337344654	5.19455E-05	1.44921E-05
ENSMUSG00000026259	0.245333333	0.006	-5.353636955	0.008931747	0.003582626
ENSMUSG00000038146	0.041	0.001	-5.357552005	0.019736659	0.008492453
ENSMUSG00000004319	16.193	0.389	-5.379456326	1.48773E-11	2.13315E-12
ENSMUSG00000032265	52.607	1.251333333	-5.393716726	0	0

ENSMUSG00000015437	94.951	2.253666667	-5.396837143	2.59628E-09	4.509E-10
ENSMUSG00000021872	0.565	0.012333333	-5.517616192	0.000818311	0.000271785
ENSMUSG00000007682	1.896	0.041	-5.531191244	8.01794E-31	4.16075E-32
ENSMUSG00000030616	0.484	0.01	-5.596935142	1.00093E-05	2.53448E-06
ENSMUSG00000043286	0.196333333	0.004	-5.617161323	0.00070759	0.000231964
ENSMUSG00000030162	48.613	0.989	-5.619227837	1.38335E-08	2.58699E-09
ENSMUSG00000031714	3.916666667	0.078333333	-5.64385619	1.53779E-14	1.76479E-15
ENSMUSG00000029651	2.779333333	0.055333333	-5.650446225	0.006002455	0.002316335
ENSMUSG00000061928	0.844333333	0.016	-5.721668861	6.46148E-09	1.16659E-09
ENSMUSG00000040852	0.497666667	0.009333333	-5.736643528	6.80466E-09	1.23235E-09
ENSMUSG00000053338	2.439	0.044666667	-5.770947353	1.71698E-07	3.55921E-08
ENSMUSG00000000706	0.327666667	0.006	-5.771122605	0.000582325	0.000188391
ENSMUSG00000047496	0.057	0.001	-5.832890014	0.005690911	0.002187018
ENSMUSG00000039783	2.524	0.042333333	-5.897774009	3.42019E-17	3.33346E-18
ENSMUSG00000022885	0.603333333	0.01	-5.914883386	0.019481743	0.008370586
ENSMUSG00000020620	0.060666667	0.001	-5.922832139	0.014867073	0.006225683
ENSMUSG00000052234	724.6346667	11.773	-5.943699926	5.45802E-06	1.34261E-06
ENSMUSG00000035165	7.412666667	0.116	-5.997795927	3.72645E-18	3.42487E-19
ENSMUSG00000099974	3.055	0.047666667	-6.002047829	4.77535E-07	1.04165E-07
ENSMUSG00000008845	0.064333333	0.001	-6.007494537	0.047876522	0.022489826
ENSMUSG00000024070	21.22866667	0.295666667	-6.165898347	2.7735E-49	8.51606E-51
ENSMUSG00000040065	0.072333333	0.001	-6.176588732	0.039081583	0.017937619
ENSMUSG00000038068	1.86	0.025333333	-6.198121893	1.34757E-16	1.35553E-17
ENSMUSG00000051111	0.076666667	0.001	-6.26052755	0.001155296	0.000393338
ENSMUSG00000030717	2.817666667	0.036	-6.290360232	1.04285E-06	2.36243E-07
ENSMUSG00000001225	11.234	0.143	-6.295712751	4.14524E-46	1.37359E-47
ENSMUSG00000035200	0.079	0.001	-6.303780748	0.049573598	0.02337656
ENSMUSG00000118661	5.533333333	0.07	-6.304650103	2.704E-175	1.7845E-177
ENSMUSG00000091455	0.849666667	0.010666667	-6.315715658	6.52717E-17	6.44782E-18
ENSMUSG00000078606	1.680333333	0.021	-6.322214316	1.17574E-07	2.39641E-08
ENSMUSG00000052273	0.085666667	0.001	-6.420662048	0.00096229	0.000323081
ENSMUSG00000024171	3.720333333	0.043	-6.434951419	1.1947E-08	2.21429E-09

ENSMUSG00000026678	0.090666667	0.001	-6.502500341	0.013267991	0.005490616
ENSMUSG00000078796	0.096	0.001	-6.584962501	0.01213578	0.004988358
ENSMUSG00000030380	0.099333333	0.001	-6.63420602	0.038357683	0.017576052
ENSMUSG00000036295	12.521	0.126	-6.634782245	1.64123E-10	2.56645E-11
ENSMUSG00000044165	2.003	0.019666667	-6.670266157	2.04875E-05	5.39123E-06
ENSMUSG00000030739	0.105333333	0.001	-6.718818247	0.002109004	0.000752473
ENSMUSG00000001865	876.229	8.309	-6.720489296	2.69914E-14	3.14447E-15
ENSMUSG00000020961	12.37666667	0.114	-6.76244518	2.10554E-40	8.36658E-42
ENSMUSG00000034224	0.108666667	0.001	-6.763765654	0.036434621	0.016611353
ENSMUSG00000028836	0.112666667	0.001	-6.815916936	0.046073995	0.021537472
ENSMUSG00000039835	0.112666667	0.001	-6.815916936	0.001296075	0.00044415
ENSMUSG00000050108	1.546	0.013666667	-6.8217351	9.30176E-09	1.70592E-09
ENSMUSG00000028807	1.028666667	0.009	-6.836634844	7.89944E-07	1.77141E-07
ENSMUSG00000008540	12.24466667	0.101333333	-6.916900867	1.98589E-17	1.89553E-18
ENSMUSG00000089929	39.95833333	0.327333333	-6.931592075	1.79422E-58	4.58682E-60
ENSMUSG00000028626	0.125	0.001	-6.965784285	0.013707805	0.005693572
ENSMUSG00000042581	1.136	0.009	-6.979822118	1.33576E-13	1.64244E-14
ENSMUSG00000032773	0.129	0.001	-7.011227255	0.007861142	0.003112783
ENSMUSG00000031616	0.131	0.001	-7.033423002	0.004516448	0.001703982
ENSMUSG00000026204	0.133333333	0.001	-7.058893689	0.004021745	0.001506164
ENSMUSG00000020323	6.760666667	0.049333333	-7.098458937	2.16458E-22	1.5834E-23
ENSMUSG00000049456	0.139333333	0.001	-7.122396631	0.006299077	0.00244174
ENSMUSG00000067399	1.443333333	0.010333333	-7.125954999	2.95548E-07	6.31953E-08
ENSMUSG00000114942	0.140333333	0.001	-7.132713922	0.016041694	0.006757682
ENSMUSG00000028864	0.140333333	0.001	-7.132713922	0.006200986	0.002401132
ENSMUSG00000028071	2.621666667	0.018666667	-7.133876128	2.159E-14	2.50171E-15
ENSMUSG00000043969	0.152333333	0.001	-7.251087854	0.027625063	0.012236013
ENSMUSG00000038349	0.153	0.001	-7.257387843	2.34774E-05	6.23183E-06
ENSMUSG00000047462	0.154333333	0.001	-7.269905883	0.007697486	0.003038889
ENSMUSG00000035177	0.156	0.001	-7.285402219	0.001819844	0.000640959
ENSMUSG00000050377	0.157333333	0.001	-7.297680549	0.003741376	0.001392573
ENSMUSG00000024747	0.169333333	0.001	-7.403722186	0.01553558	0.006532606

ENSMUSG00000024659	1000.327	5.876333333	-7.411339734	7.85191E-14	9.46375E-15
ENSMUSG00000097084	0.174666667	0.001	-7.448460501	0.0035331	0.001307946
ENSMUSG00000070780	1.591333333	0.009	-7.466095349	7.92015E-09	1.44428E-09
ENSMUSG00000079162	0.179	0.001	-7.483815777	0.010657256	0.004330274
ENSMUSG00000022225	898.3516667	4.962333333	-7.500117851	1.77348E-10	2.77831E-11
ENSMUSG00000085957	0.181666667	0.001	-7.505149919	0.01197591	0.004915169
ENSMUSG00000042532	0.189333333	0.001	-7.564784619	0.00172233	0.00060458
ENSMUSG00000041052	10.57633333	0.053666667	-7.622597554	3.01574E-32	1.5063E-33
ENSMUSG00000089953	0.200333333	0.001	-7.64625868	0.009689433	0.003911448
ENSMUSG00000000869	6.52	0.032	-7.670656249	1.31689E-08	2.45905E-09
ENSMUSG00000040314	293.3983333	1.316	-7.800557376	0	0
ENSMUSG00000042182	0.224333333	0.001	-7.809500194	0.001873772	0.000662296
ENSMUSG00000024899	17.65933333	0.078	-7.822741041	1.33593E-49	4.02775E-51
ENSMUSG00000048216	0.228666667	0.001	-7.837102265	0.001744348	0.000612673
ENSMUSG00000078670	0.231	0.001	-7.851749041	0.000793979	0.000262987
ENSMUSG00000048992	0.234	0.001	-7.87036472	0.026006729	0.011455969
ENSMUSG00000056258	0.234333333	0.001	-7.872418378	6.29581E-09	1.13189E-09
ENSMUSG00000031075	0.241333333	0.001	-7.914883386	0.000977881	0.000328655
ENSMUSG00000056643	2.758333333	0.011333333	-7.927080755	3.04394E-09	5.33086E-10
ENSMUSG00000055541	8.248	0.033333333	-7.950934928	1.19066E-20	9.49547E-22
ENSMUSG00000027400	0.257333333	0.001	-8.007494537	0.000743358	0.000244722
ENSMUSG00000024680	309.9303333	1.137333333	-8.090145004	8.78939E-46	2.94913E-47
ENSMUSG00000017195	0.274	0.001	-8.098032083	0.014550206	0.006083897
ENSMUSG00000032068	0.276666667	0.001	-8.112005026	0.005537904	0.002122446
ENSMUSG00000054901	1.573	0.005666667	-8.116802615	1.34128E-09	2.26513E-10
ENSMUSG00000030785	17.59266667	0.062	-8.14849025	4.4954E-14	5.33077E-15
ENSMUSG00000023972	0.285	0.001	-8.154818109	5.16381E-06	1.26761E-06
ENSMUSG00000102037	53.75966667	0.183333333	-8.195911265	3.07709E-51	8.89247E-53
ENSMUSG00000021108	1.604333333	0.005333333	-8.232720708	4.73366E-10	7.68172E-11
ENSMUSG00000043639	0.311333333	0.001	-8.282316239	1.12563E-06	2.56248E-07
ENSMUSG00000041794	0.330333333	0.001	-8.367778746	0.001699563	0.000596116
ENSMUSG00000090202	0.334	0.001	-8.383704292	0.024364259	0.010685069

ENSMUSG00000041857	0.344333333	0.001	-8.427662038	0.019907948	0.008573072
ENSMUSG00000026981	0.354	0.001	-8.46760555	0.017919763	0.007637217
ENSMUSG00000021662	0.365666667	0.001	-8.51438531	2.10113E-07	4.39785E-08
ENSMUSG00000020383	0.384333333	0.001	-8.586214297	0.007792239	0.003079545
ENSMUSG00000040627	0.39	0.001	-8.607330314	0.000654234	0.000213472
ENSMUSG00000055567	0.434666667	0.001	-8.763765654	2.82704E-06	6.73029E-07
ENSMUSG00000069830	7.358333333	0.016666667	-8.786269628	4.23194E-24	2.86343E-25
ENSMUSG00000031074	0.448666667	0.001	-8.809500194	7.26653E-05	2.07471E-05
ENSMUSG00000001864	0.471	0.001	-8.87958325	1.09484E-06	2.48934E-07
ENSMUSG00000032035	20.30766667	0.041333333	-8.940503055	4.41619E-46	1.46951E-47
ENSMUSG00000109850	0.541666667	0.001	-9.081261502	0.026006729	0.011455969
ENSMUSG00000078907	0.57	0.001	-9.154818109	2.1432E-07	4.49187E-08
ENSMUSG00000002324	0.583333333	0.001	-9.188176706	3.32946E-06	7.98422E-07
ENSMUSG00000079451	0.584666667	0.001	-9.191470532	3.03896E-07	6.50648E-08
ENSMUSG00000056399	586.8093333	0.988	-9.21416506	1.63237E-32	8.08529E-34
ENSMUSG00000114582	0.599	0.001	-9.226412193	2.7299E-05	7.30692E-06
ENSMUSG00000079580	0.625333333	0.001	-9.288481612	0.004516448	0.001703982
ENSMUSG00000026836	0.649333333	0.001	-9.342815461	1.56669E-07	3.23569E-08
ENSMUSG00000026765	0.649666667	0.001	-9.343555875	1.17916E-06	2.69334E-07
ENSMUSG00000005339	616.7256667	0.926333333	-9.378881745	3.1978E-51	9.27101E-53
ENSMUSG00000015970	0.687333333	0.001	-9.424866117	4.77058E-10	7.74826E-11
ENSMUSG00000091649	0.701666667	0.001	-9.454642017	0.000174129	5.22084E-05
ENSMUSG00000027233	0.716	0.001	-9.483815777	2.32984E-07	4.9057E-08
ENSMUSG00000028121	0.724	0.001	-9.499845887	2.8795E-06	6.85918E-07
ENSMUSG00000030468	0.812333333	0.001	-9.665928035	4.45835E-08	8.72468E-09
ENSMUSG00000107417	0.865	0.001	-9.756556323	2.793E-05	7.48357E-06
ENSMUSG00000029811	0.974666667	0.001	-9.928765095	1.08512E-08	2.0029E-09
ENSMUSG00000041481	1.045	0.001	-10.02928723	3.58837E-06	8.64499E-07
ENSMUSG00000022157	776.0776667	0.729666667	-10.05474777	1.2482E-299	3.6417E-302
ENSMUSG00000031594	1.220333333	0.001	-10.25305956	3.10514E-06	7.41824E-07
ENSMUSG00000040118	1.331	0.001	-10.37829486	1.04383E-14	1.18632E-15
ENSMUSG00000053475	1.670333333	0.001	-10.70592032	2.34447E-09	4.06028E-10

ENSMUSG00000094083	1.880333333	0.001	-10.87677272	1.47608E-06	3.41461E-07
ENSMUSG00000058216	2.179333333	0.001	-11.08967116	6.32921E-07	1.40258E-07
