# Supplementary material for: Mutations of RAS genes identified in acute myeloid leukemia affect glycerophospholipid metabolism pathway
Source: Front Oncol. 2023 Nov 14;13:1280192. doi: 10.3389/fonc.2023.1280192 (PMC10682766; doi:10.3389/fonc.2023.1280192)
Supplement: Supplementary file 3 [file DataSheet_3.pdf]

**The up-regulated genes of NRAS（Q61K）cell line**

| ENSEMBL | BaF3 | BaF3 NRAS(Q61K) | log2fc | FDR | Pvalue |
|---|---|---|---|---|---|
| ENSMUSG00000028214 | 0.001 | 30.802 | 14.91073641 | 2.71578E-17 | 2.63628E-18 |
| ENSMUSG00000092586 | 0.001 | 27.93766667 | 14.76992391 | 6.10184E-19 | 5.4219E-20 |
| ENSMUSG00000030638 | 0.001 | 13.28033333 | 13.69700374 | 3.0547E-18 | 2.79445E-19 |
| ENSMUSG00000022583 | 0.001 | 13.144 | 13.68211676 | 2.2344E-09 | 3.78725E-10 |
| ENSMUSG00000042351 | 0.001 | 11.23166667 | 13.4552844 | 1.74269E-16 | 1.76146E-17 |
| ENSMUSG00000023993 | 0.001 | 8.491 | 13.05171876 | 9.63052E-11 | 1.45349E-11 |
| ENSMUSG00000061815 | 0.001 | 6.283333333 | 12.6173144 | 1.49489E-16 | 1.50583E-17 |
| ENSMUSG00000032258 | 0.001 | 5.390333333 | 12.39615878 | 2.89456E-09 | 4.95017E-10 |
| ENSMUSG00000034923 | 0.001 | 5.220333333 | 12.34992621 | 1.06346E-12 | 1.36347E-13 |
| ENSMUSG00000057596 | 0.001 | 5.138333333 | 12.32708477 | 5.40075E-16 | 5.62672E-17 |
| ENSMUSG00000044912 | 0.001 | 4.884666667 | 12.2540444 | 2.37931E-14 | 2.74006E-15 |
| ENSMUSG00000028536 | 0.001 | 3.545666667 | 11.7918412 | 3.46817E-10 | 5.54084E-11 |
| ENSMUSG00000108929 | 0.001 | 3.259666667 | 11.67050873 | 3.12182E-15 | 3.41194E-16 |
| ENSMUSG00000071561 | 0.001 | 3.104333333 | 11.60006776 | 2.21083E-07 | 4.49981E-08 |
| ENSMUSG00000000182 | 0.011666667 | 35.73133333 | 11.58058161 | 1.92145E-21 | 1.49906E-22 |
| ENSMUSG00000032690 | 0.001 | 2.936666667 | 11.5199638 | 3.15219E-15 | 3.4473E-16 |
| ENSMUSG00000026582 | 0.042 | 116.2293333 | 11.43429727 | 9.59769E-35 | 4.59092E-36 |
| ENSMUSG00000073001 | 0.001 | 2.544666667 | 11.31326097 | 4.94737E-07 | 1.04695E-07 |
| ENSMUSG00000028217 | 0.001 | 2.090666667 | 11.02974734 | 4.13828E-13 | 5.1657E-14 |
| ENSMUSG00000029553 | 0.001 | 1.815333333 | 10.82601877 | 1.25436E-09 | 2.08886E-10 |
| ENSMUSG00000022659 | 0.001 | 1.781 | 10.7984718 | 2.1652E-10 | 3.39041E-11 |
| ENSMUSG00000074491 | 0.001 | 1.724666667 | 10.75210184 | 1.7297E-08 | 3.1766E-09 |
| ENSMUSG00000031750 | 0.001 | 1.691666667 | 10.72422961 | 1.01905E-09 | 1.68575E-10 |
| ENSMUSG00000040328 | 0.001 | 1.648666667 | 10.68708402 | 2.6744E-10 | 4.23022E-11 |
| ENSMUSG00000114635 | 0.001 | 1.520666667 | 10.57048823 | 3.77382E-09 | 6.5216E-10 |
| ENSMUSG00000022053 | 0.001 | 1.25 | 10.28771238 | 4.79316E-13 | 6.02289E-14 |
| ENSMUSG00000074344 | 0.001 | 1.193 | 10.22037833 | 2.08747E-05 | 5.40746E-06 |
| ENSMUSG00000114432 | 0.001 | 1.09 | 10.09011242 | 3.26931E-06 | 7.66992E-07 |
| ENSMUSG00000073413 | 0.001 | 0.967 | 9.917372079 | 0.001050027 | 0.000347544 |
| ENSMUSG00000030742 | 0.099666667 | 92.311 | 9.855175779 | 7.63512E-58 | 2.02423E-59 |

| | | | | | |
|---|---|---|---|---|---|
| ENSMUSG00000030769 | 0.001 | 0.794 | 9.632995197 | 0.000624234 | 0.000198898 |
| ENSMUSG00000017724 | 0.001 | 0.741333333 | 9.533978572 | 8.50701E-08 | 1.66863E-08 |
| ENSMUSG00000032062 | 0.001 | 0.720666667 | 9.493188307 | 3.53726E-05 | 9.44394E-06 |
| ENSMUSG00000017897 | 0.001 | 0.718666667 | 9.489178962 | 9.74131E-08 | 1.92149E-08 |
| ENSMUSG00000069892 | 0.006666667 | 4.508 | 9.401306206 | 3.09247E-17 | 3.01457E-18 |
| ENSMUSG00000040759 | 0.001 | 0.647 | 9.337621902 | 0.000301113 | 9.13068E-05 |
| ENSMUSG00000027803 | 0.001 | 0.637666667 | 9.316658658 | 1.05161E-08 | 1.88846E-09 |
| ENSMUSG00000044303 | 0.001 | 0.634 | 9.30833903 | 0.002362618 | 0.000835986 |
| ENSMUSG00000022902 | 0.001 | 0.613666667 | 9.261311411 | 0.023890271 | 0.010350142 |
| ENSMUSG00000059336 | 0.036666667 | 22.38466667 | 9.253826002 | 2.25889E-37 | 9.85651E-39 |
| ENSMUSG00000029915 | 0.005333333 | 3.195 | 9.226562717 | 3.03125E-12 | 4.04544E-13 |
| ENSMUSG00000026572 | 0.001 | 0.583333333 | 9.188176706 | 5.29458E-07 | 1.12588E-07 |
| ENSMUSG00000033730 | 0.005 | 2.836333333 | 9.147883282 | 1.04135E-12 | 1.33441E-13 |
| ENSMUSG00000006522 | 0.001 | 0.557666667 | 9.123259229 | 1.47405E-07 | 2.95237E-08 |
| ENSMUSG00000022215 | 0.001 | 0.557 | 9.121533517 | 0.001051872 | 0.0003483 |
| ENSMUSG00000037833 | 0.001 | 0.539 | 9.074141463 | 2.48498E-06 | 5.73893E-07 |
| ENSMUSG00000024034 | 0.001 | 0.521333333 | 9.026062297 | 9.60968E-07 | 2.1158E-07 |
| ENSMUSG00000069893 | 0.001 | 0.491333333 | 8.940558308 | 2.56818E-06 | 5.9364E-07 |
| ENSMUSG00000050232 | 0.001 | 0.479 | 8.903881846 | 0.000255635 | 7.69163E-05 |
| ENSMUSG00000006389 | 0.9 | 429.525 | 8.898601387 | 0 | 0 |
| ENSMUSG00000023333 | 0.001 | 0.452666667 | 8.822305264 | 2.44083E-05 | 6.38182E-06 |
| ENSMUSG00000001020 | 0.161 | 66.71666667 | 8.694842711 | 6.96348E-31 | 3.7404E-32 |
| ENSMUSG00000097919 | 0.001 | 0.411 | 8.682994584 | 0.040422032 | 0.018466763 |
| ENSMUSG00000038246 | 0.001 | 0.404 | 8.658211483 | 0.001457784 | 0.00049559 |
| ENSMUSG00000035441 | 0.001 | 0.401 | 8.647458426 | 1.88647E-07 | 3.82269E-08 |
| ENSMUSG00000066975 | 0.001 | 0.37 | 8.531381461 | 0.025177499 | 0.010959965 |
| ENSMUSG00000005611 | 0.638333333 | 233.8053333 | 8.516782138 | 0 | 0 |
| ENSMUSG00000047343 | 0.001 | 0.363666667 | 8.506472886 | 0.005154639 | 0.001941709 |
| ENSMUSG00000034028 | 0.001 | 0.357333333 | 8.48112669 | 0.000414318 | 0.000128638 |
| ENSMUSG00000041287 | 0.001 | 0.352 | 8.459431619 | 0.045382324 | 0.021039927 |
| ENSMUSG00000029372 | 0.001 | 0.351333333 | 8.456696651 | 0.00833752 | 0.003269926 |
| ENSMUSG00000070469 | 0.001 | 0.330333333 | 8.367778746 | 8.65857E-09 | 1.54397E-09 |

| | | | | | |
|---|---|---|---|---|---|
| ENSMUSG00000062980 | 0.009333333 | 2.953333333 | 8.305736061 | 1.26934E-13 | 1.53073E-14 |
| ENSMUSG00000002076 | 0.001 | 0.310666667 | 8.279223644 | 0.000264192 | 7.96006E-05 |
| ENSMUSG00000000320 | 0.001 | 0.309 | 8.271463028 | 3.10171E-05 | 8.20614E-06 |
| ENSMUSG00000066510 | 0.001 | 0.307333333 | 8.26366044 | 0.002583526 | 0.000919859 |
| ENSMUSG00000026829 | 0.001 | 0.305666667 | 8.255815423 | 0.006487727 | 0.002488664 |
| ENSMUSG00000048480 | 0.001 | 0.301666667 | 8.236811481 | 0.016275408 | 0.006785601 |
| ENSMUSG00000027204 | 0.008333333 | 2.392666667 | 8.165510018 | 1.60083E-27 | 9.62666E-29 |
| ENSMUSG00000030465 | 0.001 | 0.284333333 | 8.151439431 | 0.003566794 | 0.001302702 |
| ENSMUSG00000020847 | 0.001 | 0.282 | 8.139551352 | 0.005837488 | 0.002215053 |
| ENSMUSG00000047867 | 0.001 | 0.282 | 8.139551352 | 0.003788689 | 0.001391854 |
| ENSMUSG00000022623 | 0.045666667 | 12.57866667 | 8.105621803 | 4.8176E-76 | 9.91193E-78 |
| ENSMUSG00000006344 | 0.005 | 1.335666667 | 8.061416199 | 4.28484E-07 | 9.00514E-08 |
| ENSMUSG00000031824 | 0.001 | 0.254666667 | 7.992466327 | 1.19629E-05 | 3.00312E-06 |
| ENSMUSG00000097565 | 0.001 | 0.248333333 | 7.956134115 | 0.020056799 | 0.008537018 |
| ENSMUSG00000027938 | 0.001 | 0.237 | 7.888743249 | 0.014819143 | 0.006114278 |
| ENSMUSG00000030350 | 0.014666667 | 3.433 | 7.870785024 | 1.40694E-12 | 1.82618E-13 |
| ENSMUSG00000002365 | 0.042666667 | 9.823333333 | 7.84695944 | 6.08295E-43 | 2.30988E-44 |
| ENSMUSG00000078921 | 0.028666667 | 6.563 | 7.838837464 | 4.56194E-24 | 3.18429E-25 |
| ENSMUSG00000022747 | 0.001 | 0.220333333 | 7.783543961 | 0.003675811 | 0.00134734 |
| ENSMUSG00000030410 | 0.008333333 | 1.83 | 7.778734244 | 3.11514E-09 | 5.34148E-10 |
| ENSMUSG00000029586 | 0.001 | 0.211666667 | 7.725650281 | 0.004207351 | 0.001559601 |
| ENSMUSG00000052414 | 0.001 | 0.209 | 7.707359132 | 0.037823275 | 0.01714112 |
| ENSMUSG00000078920 | 0.180333333 | 37.396 | 7.696074059 | 2.0265E-81 | 3.88959E-83 |
| ENSMUSG00000032261 | 0.001 | 0.184666667 | 7.528779665 | 0.00017724 | 5.21538E-05 |
| ENSMUSG00000078810 | 0.001 | 0.183666667 | 7.520946008 | 0.00680785 | 0.002622743 |
| ENSMUSG00000009772 | 0.007 | 1.201666667 | 7.423466121 | 1.37756E-08 | 2.50992E-09 |
| ENSMUSG00000005131 | 0.001 | 0.163 | 7.348728154 | 0.020469679 | 0.008733956 |
| ENSMUSG00000029861 | 0.001 | 0.162333333 | 7.342815461 | 0.000647677 | 0.000207038 |
| ENSMUSG00000057074 | 0.001 | 0.157 | 7.294620749 | 0.006751116 | 0.002599953 |
| ENSMUSG00000030409 | 0.069666667 | 10.829 | 7.280215772 | 8.4577E-24 | 6.00868E-25 |
| ENSMUSG00000022219 | 0.017666667 | 2.711333333 | 7.261828821 | 4.81373E-08 | 9.20604E-09 |
| ENSMUSG00000029166 | 0.001 | 0.148 | 7.209453366 | 0.033652838 | 0.015072215 |

| | | | | | |
|---|---|---|---|---|---|
| ENSMUSG00000089694 | 0.001 | 0.147333333 | 7.202940059 | 0.038931912 | 0.017691162 |
| ENSMUSG00000054675 | 0.418 | 56.53766667 | 7.079565591 | 5.0163E-171 | 4.1907E-173 |
| ENSMUSG00000020838 | 0.092666667 | 11.865 | 7.000445908 | 1.55376E-06 | 3.52288E-07 |
| ENSMUSG00000044258 | 0.423333333 | 51.94633333 | 6.939084018 | 2.1334E-109 | 3.0932E-111 |
| ENSMUSG00000074874 | 0.190666667 | 22.87033333 | 6.906280937 | 4.86607E-37 | 2.16359E-38 |
| ENSMUSG00000048378 | 0.001 | 0.116 | 6.857980995 | 0.049267278 | 0.023041739 |
| ENSMUSG00000022836 | 0.046 | 5.320666667 | 6.853829352 | 1.46932E-65 | 3.47953E-67 |
| ENSMUSG00000018500 | 0.022666667 | 2.567666667 | 6.823741869 | 2.35549E-09 | 3.99575E-10 |
| ENSMUSG00000020689 | 1.675666667 | 189.4046667 | 6.820592879 | 0 | 0 |
| ENSMUSG00000029298 | 0.007333333 | 0.819 | 6.803250524 | 1.69313E-07 | 3.40754E-08 |
| ENSMUSG00000020447 | 0.001 | 0.110333333 | 6.785724906 | 0.002057629 | 0.000718977 |
| ENSMUSG00000032280 | 0.001 | 0.11 | 6.781359714 | 0.016917999 | 0.007080703 |
| ENSMUSG00000047511 | 0.001 | 0.107 | 6.741466986 | 0.010512906 | 0.004197469 |
| ENSMUSG00000056656 | 0.001 | 0.104333333 | 6.705056346 | 0.024808265 | 0.010788957 |
| ENSMUSG00000026805 | 0.001 | 0.098333333 | 6.619608644 | 0.049337443 | 0.02307796 |
| ENSMUSG00000028197 | 0.001 | 0.096666667 | 6.594946589 | 0.000192988 | 5.71609E-05 |
| ENSMUSG00000040562 | 0.439 | 41.78666667 | 6.572677929 | 8.67161E-88 | 1.55064E-89 |
| ENSMUSG00000027800 | 0.007 | 0.666 | 6.572023445 | 0.001354427 | 0.000456993 |
| ENSMUSG00000029765 | 0.001666667 | 0.155666667 | 6.545350645 | 3.08517E-06 | 7.22301E-07 |
| ENSMUSG00000035638 | 0.589 | 53.887 | 6.515525827 | 8.8293E-225 | 4.7548E-227 |
| ENSMUSG00000074892 | 0.001 | 0.088 | 6.459431619 | 0.002268824 | 0.000798725 |
| ENSMUSG00000030787 | 0.014333333 | 1.260666667 | 6.458668893 | 1.00712E-08 | 1.80579E-09 |
| ENSMUSG00000022055 | 0.001 | 0.087333333 | 6.448460501 | 0.020360778 | 0.008676244 |
| ENSMUSG00000073802 | 0.014333333 | 1.246666667 | 6.4425578 | 4.24735E-06 | 1.01404E-06 |
| ENSMUSG00000031530 | 1.062333333 | 89.38166667 | 6.394670523 | 0 | 0 |
| ENSMUSG00000031026 | 0.025333333 | 2.074 | 6.355235166 | 6.20352E-34 | 3.05379E-35 |
| ENSMUSG00000017057 | 0.001 | 0.081333333 | 6.345774837 | 0.018700861 | 0.007897899 |
| ENSMUSG00000030054 | 0.376333333 | 30.32466667 | 6.332336897 | 5.80609E-56 | 1.60746E-57 |
| ENSMUSG00000022102 | 2.738666667 | 216.95 | 6.307745095 | 0 | 0 |
| ENSMUSG00000022012 | 0.001 | 0.078666667 | 6.297680549 | 0.049219325 | 0.023009117 |
| ENSMUSG00000057378 | 0.002666667 | 0.204666667 | 6.262094845 | 1.31664E-07 | 2.62255E-08 |
| ENSMUSG00000094151 | 0.011333333 | 0.841333333 | 6.214033354 | 1.43992E-05 | 3.66044E-06 |

| | | | | | |
|---|---|---|---|---|---|
| ENSMUSG00000062438 | 0.001 | 0.074 | 6.209453366 | 0.036485154 | 0.016486836 |
| ENSMUSG00000030107 | 0.128333333 | 9.418 | 6.197452872 | 4.20326E-32 | 2.18521E-33 |
| ENSMUSG00000030737 | 0.031333333 | 2.155333333 | 6.10406894 | 1.64107E-12 | 2.14254E-13 |
| ENSMUSG00000070345 | 0.001 | 0.068666667 | 6.101538026 | 0.03054353 | 0.013536241 |
| ENSMUSG00000074577 | 0.229333333 | 15.294 | 6.059375905 | 4.5344E-122 | 5.6351E-124 |
| ENSMUSG00000031639 | 0.005 | 0.333 | 6.057450272 | 4.40828E-05 | 1.19499E-05 |
| ENSMUSG00000021675 | 0.352666667 | 23.282 | 6.044765964 | 7.82327E-93 | 1.32873E-94 |
| ENSMUSG00000026726 | 0.005333333 | 0.346 | 6.019590728 | 3.55525E-09 | 6.12425E-10 |
| ENSMUSG00000026832 | 0.034666667 | 2.202 | 5.989121536 | 9.20831E-13 | 1.17552E-13 |
| ENSMUSG00000042106 | 1.489666667 | 93.16566667 | 5.966736943 | 1.02E-188 | 7.3947E-191 |
| ENSMUSG00000000386 | 0.084 | 5.201 | 5.9522559 | 3.04038E-08 | 5.71383E-09 |
| ENSMUSG00000079597 | 0.051666667 | 3.080666667 | 5.897864969 | 0.000131488 | 3.80285E-05 |
| ENSMUSG00000020599 | 0.015 | 0.872333333 | 5.861845113 | 2.95421E-06 | 6.89397E-07 |
| ENSMUSG00000038963 | 2.192 | 125.013 | 5.833686519 | 0 | 0 |
| ENSMUSG00000005824 | 1.592 | 86.93566667 | 5.771035945 | 1.982E-290 | 6.7052E-293 |
| ENSMUSG00000022438 | 0.402666667 | 21.16 | 5.715609768 | 1.5131E-144 | 1.5879E-146 |
| ENSMUSG00000033207 | 0.549666667 | 28.57966667 | 5.700288287 | 1.37E-211 | 8.3238E-214 |
| ENSMUSG00000042807 | 0.001 | 0.051 | 5.672425342 | 0.001364796 | 0.000460963 |
| ENSMUSG00000048148 | 0.02 | 1.019333333 | 5.671482096 | 1.01475E-21 | 7.81867E-23 |
| ENSMUSG00000037820 | 1.271666667 | 64.29933333 | 5.660011318 | 0 | 0 |
| ENSMUSG00000026579 | 0.48 | 23.83633333 | 5.633984112 | 1.2697E-260 | 5.0845E-263 |
| ENSMUSG00000036617 | 0.001 | 0.049 | 5.614709844 | 0.024389673 | 0.010590076 |
| ENSMUSG00000090958 | 0.005333333 | 0.260666667 | 5.611024797 | 0.000724577 | 0.000233372 |
| ENSMUSG00000090066 | 0.016666667 | 0.796 | 5.577730931 | 2.44202E-19 | 2.12437E-20 |
| ENSMUSG00000027636 | 1.603 | 74.92666667 | 5.54663294 | 0 | 0 |
| ENSMUSG00000039109 | 6.287333333 | 284.1973333 | 5.498300955 | 0 | 0 |
| ENSMUSG00000001025 | 0.029333333 | 1.322 | 5.494037344 | 0.000295939 | 8.96766E-05 |
| ENSMUSG00000069833 | 0.042333333 | 1.813333333 | 5.420706249 | 1.04664E-51 | 3.22289E-53 |
| ENSMUSG00000061751 | 0.148 | 6.267 | 5.404105912 | 3.97146E-41 | 1.57114E-42 |
| ENSMUSG00000059810 | 0.035666667 | 1.493 | 5.387493964 | 2.35141E-05 | 6.13828E-06 |
| ENSMUSG00000035168 | 0.011333333 | 0.474 | 5.386242908 | 1.42417E-10 | 2.19171E-11 |
| ENSMUSG00000042345 | 0.612 | 25.02633333 | 5.353771471 | 5.2733E-172 | 4.3325E-174 |

| | | | | | |
|---|---|---|---|---|---|
| ENSMUSG00000031698 | 0.366666667 | 14.88066667 | 5.342826234 | 1.18002E-98 | 1.89826E-100 |
| ENSMUSG00000026715 | 0.01 | 0.396 | 5.307428525 | 0.000650609 | 0.000208021 |
| ENSMUSG00000026581 | 6.330333333 | 245.0233333 | 5.274493863 | 3.5503E-132 | 3.9954E-134 |
| ENSMUSG00000005125 | 12.084 | 444.7006667 | 5.201664573 | 0 | 0 |
| ENSMUSG00000024401 | 0.315666667 | 11.6 | 5.19957907 | 1.90515E-49 | 6.09005E-51 |
| ENSMUSG00000062937 | 15.85266667 | 577.6716667 | 5.187452286 | 0 | 0 |
| ENSMUSG00000027765 | 5.311 | 187.55 | 5.142147923 | 0 | 0 |
| ENSMUSG00000021280 | 0.399666667 | 14.08733333 | 5.139457479 | 2.92069E-67 | 6.77542E-69 |
| ENSMUSG00000006445 | 0.010333333 | 0.359 | 5.118606224 | 6.18088E-05 | 1.7125E-05 |
| ENSMUSG00000037868 | 0.154666667 | 5.32 | 5.104192036 | 2.0492E-38 | 8.7152E-40 |
| ENSMUSG00000002847 | 0.01 | 0.342666667 | 5.098733954 | 0.00457822 | 0.001710037 |
| ENSMUSG00000057137 | 0.217333333 | 7.422333333 | 5.093891424 | 3.94973E-35 | 1.8516E-36 |
| ENSMUSG00000029561 | 0.276333333 | 9.100333333 | 5.041437884 | 2.34466E-54 | 6.78273E-56 |
| ENSMUSG00000007379 | 0.203666667 | 6.645666667 | 5.028132148 | 4.28329E-84 | 7.95502E-86 |
| ENSMUSG00000000244 | 2.242 | 72.58366667 | 5.016786755 | 3.1578E-218 | 1.8095E-220 |
| ENSMUSG00000029096 | 0.042666667 | 1.369666667 | 5.004571615 | 4.15088E-06 | 9.88141E-07 |
| ENSMUSG00000007035 | 0.207 | 6.587333333 | 4.991991883 | 3.93419E-10 | 6.32339E-11 |
| ENSMUSG00000025867 | 0.012 | 0.378 | 4.977279923 | 9.77866E-06 | 2.43116E-06 |
| ENSMUSG00000021279 | 0.003333333 | 0.100666667 | 4.916476644 | 0.006050871 | 0.002302706 |
| ENSMUSG00000026580 | 0.393666667 | 11.77366667 | 4.902445319 | 2.3288E-114 | 3.1514E-116 |
| ENSMUSG00000027200 | 0.006333333 | 0.188333333 | 4.894179544 | 0.000637146 | 0.00020354 |
| ENSMUSG00000027239 | 0.058 | 1.713666667 | 4.884889801 | 5.76772E-05 | 1.59206E-05 |
| ENSMUSG00000021190 | 2.613666667 | 76.849 | 4.877879423 | 3.3573E-214 | 1.9702E-216 |
| ENSMUSG00000017737 | 0.084333333 | 2.393 | 4.826573608 | 5.42674E-23 | 3.95279E-24 |
| ENSMUSG00000021624 | 0.068666667 | 1.879333333 | 4.774467235 | 1.53155E-10 | 2.36542E-11 |
| ENSMUSG00000000308 | 0.013333333 | 0.355 | 4.73470962 | 0.017401181 | 0.007294944 |
| ENSMUSG00000024402 | 0.029 | 0.751666667 | 4.695968222 | 0.000435205 | 0.000135543 |
| ENSMUSG00000022041 | 0.017 | 0.439333333 | 4.691709313 | 1.7411E-05 | 4.46936E-06 |
| ENSMUSG00000079110 | 0.310333333 | 7.970666667 | 4.682809824 | 1.10206E-32 | 5.60008E-34 |
| ENSMUSG00000050675 | 0.415333333 | 10.65033333 | 4.680485112 | 4.0592E-76 | 8.29552E-78 |
| ENSMUSG00000024679 | 0.326666667 | 8.278333333 | 4.663449187 | 1.30972E-26 | 8.1473E-28 |
| ENSMUSG00000047033 | 0.007333333 | 0.185 | 4.656912343 | 0.006570786 | 0.002525515 |

| | | | | | |
|---|---|---|---|---|---|
| ENSMUSG00000118672 | 0.001 | 0.024666667 | 4.624490865 | 0.026585817 | 0.011629918 |
| ENSMUSG00000039997 | 0.026333333 | 0.621 | 4.559631211 | 1.59217E-05 | 4.06507E-06 |
| ENSMUSG00000032014 | 0.522333333 | 12.29533333 | 4.556996264 | 7.2797E-12 | 1.00018E-12 |
| ENSMUSG00000047250 | 7.197666667 | 168.4303333 | 4.548478882 | 0 | 0 |
| ENSMUSG00000007659 | 4.664 | 106.5136667 | 4.513326954 | 0 | 0 |
| ENSMUSG00000035183 | 4.162666667 | 94.22333333 | 4.500504429 | 1.188E-41 | 4.65062E-43 |
| ENSMUSG00000033007 | 0.008 | 0.180666667 | 4.497186541 | 0.013215988 | 0.005383481 |
| ENSMUSG00000079563 | 0.352333333 | 7.856666667 | 4.478904477 | 1.34331E-34 | 6.48281E-36 |
| ENSMUSG00000049744 | 0.346 | 7.699333333 | 4.475889589 | 6.22988E-40 | 2.52912E-41 |
| ENSMUSG00000002602 | 0.076 | 1.663666667 | 4.452223174 | 3.71226E-20 | 3.09611E-21 |
| ENSMUSG00000012819 | 0.002 | 0.043666667 | 4.448460501 | 0.018171504 | 0.007654263 |
| ENSMUSG00000074899 | 0.064666667 | 1.409666667 | 4.446188003 | 5.49782E-43 | 2.0763E-44 |
| ENSMUSG00000110040 | 0.054666667 | 1.189 | 4.442943496 | 0.002876796 | 0.00103262 |
| ENSMUSG00000052334 | 0.377 | 8.182666667 | 4.439934654 | 1.66824E-39 | 6.8531E-41 |
| ENSMUSG00000028435 | 0.011333333 | 0.243333333 | 4.424289813 | 0.019914367 | 0.008458519 |
| ENSMUSG00000029862 | 0.014666667 | 0.309666667 | 4.400103168 | 0.000484546 | 0.000151881 |
| ENSMUSG00000059852 | 0.029 | 0.587666667 | 4.340873263 | 3.13537E-05 | 8.2995E-06 |
| ENSMUSG00000063450 | 0.005666667 | 0.113 | 4.317678622 | 0.007283486 | 0.002822074 |
| ENSMUSG00000039103 | 0.676333333 | 13.226 | 4.289498537 | 3.2463E-53 | 9.59277E-55 |
| ENSMUSG00000034997 | 0.003666667 | 0.071666667 | 4.288761231 | 0.022642191 | 0.00975784 |
| ENSMUSG00000041762 | 0.081666667 | 1.517 | 4.215329932 | 8.42031E-18 | 7.88896E-19 |
| ENSMUSG00000029373 | 0.152333333 | 2.810666667 | 4.205608797 | 0.000797935 | 0.000258872 |
| ENSMUSG00000036718 | 2.024666667 | 36.71933333 | 4.180783553 | 9.926E-255 | 4.386E-257 |
| ENSMUSG00000021071 | 0.011 | 0.199 | 4.177193002 | 0.005068926 | 0.001905922 |
| ENSMUSG00000013089 | 2.207 | 39.246 | 4.152387086 | 3.5916E-193 | 2.5293E-195 |
| ENSMUSG00000046916 | 1.453 | 25.77533333 | 4.148884477 | 7.4372E-130 | 8.5237E-132 |
| ENSMUSG00000031028 | 0.003333333 | 0.058333333 | 4.129283017 | 0.035578481 | 0.016040285 |
| ENSMUSG00000037580 | 5.249333333 | 91.305 | 4.12048775 | 0 | 0 |
| ENSMUSG00000032661 | 0.289 | 4.956 | 4.10003479 | 2.39023E-52 | 7.24463E-54 |
| ENSMUSG00000032268 | 0.012 | 0.199 | 4.05166212 | 0.045175739 | 0.020928556 |
| ENSMUSG00000024427 | 0.065333333 | 1.083333333 | 4.051514159 | 2.43261E-15 | 2.63684E-16 |
| ENSMUSG00000039131 | 0.026 | 0.429 | 4.044394119 | 0.005855804 | 0.002222553 |

| | | | | | |
|---|---|---|---|---|---|
| ENSMUSG00000020787 | 2.514666667 | 40.56 | 4.011618572 | 2.5244E-178 | 1.9695E-180 |
| ENSMUSG00000095620 | 0.349666667 | 5.631 | 4.009338974 | 0.02175557 | 0.00933669 |
| ENSMUSG00000032036 | 0.020333333 | 0.327 | 4.007371989 | 0.000613491 | 0.00019539 |
| ENSMUSG00000020990 | 0.059333333 | 0.95 | 4.001012773 | 1.21198E-05 | 3.04654E-06 |
| ENSMUSG00000020407 | 0.328666667 | 5.117666667 | 3.960789131 | 4.67677E-17 | 4.5883E-18 |
| ENSMUSG00000030257 | 0.768666667 | 11.881 | 3.950154353 | 5.0559E-147 | 5.0615E-149 |
| ENSMUSG00000029344 | 8.888666667 | 136.548 | 3.941297347 | 0 | 0 |
| ENSMUSG00000030137 | 8.467333333 | 129.7363333 | 3.937531075 | 0 | 0 |
| ENSMUSG00000020029 | 18.036 | 270.97 | 3.909181815 | 0 | 0 |
| ENSMUSG00000001211 | 5.18 | 76.21333333 | 3.879019413 | 1.4687E-140 | 1.5616E-142 |
| ENSMUSG00000002699 | 10.59533333 | 154.951 | 3.870311184 | 0 | 0 |
| ENSMUSG00000025991 | 0.007333333 | 0.107 | 3.866997868 | 0.034051314 | 0.015274191 |
| ENSMUSG00000021250 | 1.297666667 | 18.91566667 | 3.865589875 | 1.0324E-57 | 2.75135E-59 |
| ENSMUSG00000024042 | 0.932 | 13.263 | 3.830933375 | 6.92761E-79 | 1.38227E-80 |
| ENSMUSG00000027315 | 0.098333333 | 1.394666667 | 3.826095992 | 3.56441E-10 | 5.70444E-11 |
| ENSMUSG00000027351 | 5.288666667 | 74.42566667 | 3.814824286 | 0 | 0 |
| ENSMUSG00000044197 | 0.834333333 | 11.69166667 | 3.808712908 | 5.13396E-68 | 1.15908E-69 |
| ENSMUSG00000024885 | 0.084 | 1.168 | 3.797507136 | 2.42646E-07 | 4.95544E-08 |
| ENSMUSG00000031799 | 10.301 | 140.3486667 | 3.768159055 | 5.9934E-227 | 3.1862E-229 |
| ENSMUSG00000034664 | 34.154 | 463.0153333 | 3.760933514 | 0 | 0 |
| ENSMUSG00000003206 | 1.219 | 16.28433333 | 3.739714627 | 3.80337E-50 | 1.19742E-51 |
| ENSMUSG00000035547 | 0.183333333 | 2.362666667 | 3.68787508 | 1.02521E-27 | 6.11561E-29 |
| ENSMUSG00000047880 | 0.046666667 | 0.594333333 | 3.670807971 | 2.81602E-05 | 7.41723E-06 |
| ENSMUSG00000053522 | 0.189333333 | 2.380333333 | 3.652163283 | 0.000280849 | 8.49876E-05 |
| ENSMUSG00000037348 | 2.281333333 | 28.308 | 3.63326066 | 2.86642E-93 | 4.82883E-95 |
| ENSMUSG00000046768 | 0.189333333 | 2.336333333 | 3.625245789 | 0.003053434 | 0.001101083 |
| ENSMUSG00000033170 | 0.009 | 0.110333333 | 3.615799905 | 0.028819596 | 0.01269662 |
| ENSMUSG00000034041 | 10.57533333 | 127.4306667 | 3.590937467 | 3.4077E-297 | 1.1058E-299 |
| ENSMUSG00000033722 | 0.181333333 | 2.18 | 3.587612079 | 8.28445E-23 | 6.08579E-24 |
| ENSMUSG00000049577 | 22.72633333 | 270.084 | 3.570971332 | 0 | 0 |
| ENSMUSG00000074604 | 6.811333333 | 76.54166667 | 3.490236174 | 1.78075E-73 | 3.82363E-75 |
| ENSMUSG00000054568 | 0.023666667 | 0.265666667 | 3.488688794 | 0.00812615 | 0.003180329 |

| | | | | | |
|---|---|---|---|---|---|
| ENSMUSG00000031511 | 12.967 | 142.9803333 | 3.462900074 | 7.4212E-258 | 3.1255E-260 |
| ENSMUSG00000035969 | 0.103666667 | 1.14 | 3.45900984 | 1.9781E-13 | 2.42277E-14 |
| ENSMUSG00000021453 | 0.284666667 | 3.120666667 | 3.45450879 | 3.73609E-08 | 7.07288E-09 |
| ENSMUSG00000019960 | 7.791 | 85.21 | 3.451142331 | 1.5166E-246 | 7.539E-249 |
| ENSMUSG00000033191 | 6.222 | 66.97866667 | 3.428251357 | 0 | 0 |
| ENSMUSG00000074342 | 2.298666667 | 24.486 | 3.413087938 | 1.13805E-72 | 2.46719E-74 |
| ENSMUSG00000044786 | 1.709333333 | 17.702 | 3.372406701 | 7.31275E-63 | 1.81253E-64 |
| ENSMUSG00000032698 | 12.127 | 123.4863333 | 3.348056779 | 7.0245E-120 | 8.8753E-122 |
| ENSMUSG00000055485 | 1.477666667 | 14.926 | 3.336434824 | 6.8709E-208 | 4.2694E-210 |
| ENSMUSG00000000290 | 8.062666667 | 81.195 | 3.332061905 | 4.6722E-205 | 2.9677E-207 |
| ENSMUSG00000028332 | 1.246 | 12.50033333 | 3.326590593 | 5.63107E-48 | 1.86614E-49 |
| ENSMUSG00000030745 | 0.330666667 | 3.29 | 3.314638059 | 1.91252E-19 | 1.65847E-20 |
| ENSMUSG00000039384 | 1.726333333 | 17.14333333 | 3.31186469 | 3.74462E-91 | 6.43751E-93 |
| ENSMUSG00000089665 | 0.16 | 1.586 | 3.309248961 | 0.017434702 | 0.0073102 |
| ENSMUSG00000048376 | 25.21 | 249.221 | 3.305357615 | 0 | 0 |
| ENSMUSG00000034751 | 0.017 | 0.167666667 | 3.301989248 | 0.000361161 | 0.000111011 |
| ENSMUSG00000036687 | 0.056 | 0.533666667 | 3.25244017 | 0.000337786 | 0.000103243 |
| ENSMUSG00000027646 | 0.042333333 | 0.401666667 | 3.246132744 | 0.001514842 | 0.00051687 |
| ENSMUSG00000037003 | 0.290666667 | 2.757666667 | 3.246010542 | 1.16592E-17 | 1.10201E-18 |
| ENSMUSG00000034459 | 0.030666667 | 0.289 | 3.236326227 | 0.007922426 | 0.003090425 |
| ENSMUSG00000032402 | 1.881 | 17.39566667 | 3.209156215 | 1.3731E-146 | 1.3841E-148 |
| ENSMUSG00000055538 | 0.066 | 0.605666667 | 3.197986084 | 4.56455E-06 | 1.09292E-06 |
| ENSMUSG00000048612 | 0.309333333 | 2.819333333 | 3.18811985 | 8.72215E-29 | 5.00422E-30 |
| ENSMUSG00000037169 | 1.082 | 9.843666667 | 3.185495306 | 8.07825E-11 | 1.2114E-11 |
| ENSMUSG00000032596 | 1.720333333 | 15.56633333 | 3.177669121 | 6.51198E-71 | 1.42972E-72 |
| ENSMUSG00000051615 | 4.336333333 | 38.70066667 | 3.157810853 | 2.0113E-239 | 1.0137E-241 |
| ENSMUSG00000053560 | 14.055 | 125.2843333 | 3.15605066 | 1.3972E-115 | 1.8714E-117 |
| ENSMUSG00000078137 | 0.083333333 | 0.74 | 3.150559677 | 4.06412E-08 | 7.71071E-09 |
| ENSMUSG00000033880 | 0.064666667 | 0.573666667 | 3.14911854 | 0.000198389 | 5.88701E-05 |
| ENSMUSG00000028602 | 0.031 | 0.275 | 3.149091498 | 0.005917814 | 0.002248394 |
| ENSMUSG00000007030 | 0.094333333 | 0.833333333 | 3.143054137 | 2.1503E-10 | 3.36411E-11 |
| ENSMUSG00000036585 | 0.032666667 | 0.284 | 3.119999776 | 0.001231565 | 0.000412137 |

| | | | | | |
|---|---|---|---|---|---|
| ENSMUSG00000057191 | 3.087 | 26.83233333 | 3.119695128 | 6.53832E-74 | 1.39488E-75 |
| ENSMUSG00000062593 | 9.445 | 81.871 | 3.115729815 | 2.04894E-09 | 3.46299E-10 |
| ENSMUSG00000040447 | 16.93733333 | 146.7896667 | 3.115471757 | 5.9956E-169 | 5.0502E-171 |
| ENSMUSG00000032020 | 7.074666667 | 61.29233333 | 3.114972548 | 1.0584E-286 | 3.7269E-289 |
| ENSMUSG00000051590 | 0.183333333 | 1.561333333 | 3.090237552 | 1.4479E-19 | 1.24856E-20 |
| ENSMUSG00000061411 | 2.659 | 22.45366667 | 3.077995373 | 2.8044E-213 | 1.6652E-215 |
| ENSMUSG00000037754 | 0.485 | 4.066 | 3.067553563 | 1.34863E-51 | 4.1714E-53 |
| ENSMUSG00000026628 | 0.075 | 0.626 | 3.061200156 | 0.000900766 | 0.000294782 |
| ENSMUSG00000075122 | 0.072333333 | 0.595 | 3.040157126 | 4.63257E-05 | 1.26049E-05 |
| ENSMUSG00000042594 | 9.049666667 | 73.97266667 | 3.031055727 | 3.6997E-213 | 2.2223E-215 |
| ENSMUSG00000056220 | 21.49933333 | 175.578 | 3.029748256 | 9.0885E-219 | 5.1454E-221 |
| ENSMUSG00000040264 | 0.282666667 | 2.295333333 | 3.021530011 | 2.30707E-14 | 2.65207E-15 |
| ENSMUSG00000028862 | 1.388333333 | 11.17233333 | 3.008504623 | 9.89554E-76 | 2.05645E-77 |
| ENSMUSG00000000562 | 0.724333333 | 5.810333333 | 3.003895258 | 1.20654E-21 | 9.31311E-23 |
| ENSMUSG00000046223 | 2.371333333 | 18.98633333 | 3.001190938 | 3.10133E-52 | 9.44274E-54 |
| ENSMUSG00000066026 | 11.704 | 93.51166667 | 2.998144695 | 2.5214E-199 | 1.706E-201 |
| ENSMUSG00000042745 | 6.226 | 49.29066667 | 2.984937012 | 5.11281E-91 | 8.82492E-93 |
| ENSMUSG00000038037 | 0.132 | 1.024333333 | 2.956075432 | 5.60648E-07 | 1.19453E-07 |
| ENSMUSG00000072595 | 0.872666667 | 6.771666667 | 2.956008362 | 3.09247E-17 | 3.01476E-18 |
| ENSMUSG00000024399 | 3.726666667 | 28.85266667 | 2.952746977 | 9.51282E-70 | 2.1017E-71 |
| ENSMUSG00000029860 | 43.78166667 | 337.8756667 | 2.948093673 | 4.6706E-178 | 3.6761E-180 |
| ENSMUSG00000003420 | 0.065 | 0.5 | 2.943416472 | 0.005478006 | 0.002070704 |
| ENSMUSG00000022453 | 0.197333333 | 1.500666667 | 2.926896976 | 2.30011E-06 | 5.29133E-07 |
| ENSMUSG00000030659 | 0.204333333 | 1.535 | 2.909242177 | 1.15983E-06 | 2.58086E-07 |
| ENSMUSG00000034165 | 39.44733333 | 296.3066667 | 2.909091405 | 0 | 0 |
| ENSMUSG00000003153 | 23.241 | 174.472 | 2.908251475 | 1.3401E-258 | 5.5512E-261 |
| ENSMUSG00000025511 | 7.306333333 | 54.42033333 | 2.896926314 | 1.1563E-102 | 1.8122E-104 |
| ENSMUSG00000034066 | 0.048 | 0.354333333 | 2.88400088 | 0.002765316 | 0.000990504 |
| ENSMUSG00000028456 | 0.017 | 0.125 | 2.878321443 | 0.00588353 | 0.002234149 |
| ENSMUSG00000022957 | 0.429666667 | 3.151333333 | 2.874672601 | 8.98879E-21 | 7.26104E-22 |
| ENSMUSG00000045659 | 1.098666667 | 8.057 | 2.874489014 | 6.46823E-33 | 3.26895E-34 |
| ENSMUSG00000054385 | 0.738666667 | 5.413666667 | 2.873610679 | 9.96262E-25 | 6.71652E-26 |

| | | | | | |
|---|---|---|---|---|---|
| ENSMUSG00000070348 | 0.111 | 0.811666667 | 2.87032769 | 1.66079E-08 | 3.04204E-09 |
| ENSMUSG00000062861 | 2.729666667 | 19.814 | 2.859723465 | 6.36242E-85 | 1.17286E-86 |
| ENSMUSG00000034663 | 11.84833333 | 84.98933333 | 2.842597652 | 0 | 0 |
| ENSMUSG00000071656 | 0.412333333 | 2.929666667 | 2.828853527 | 3.7883E-18 | 3.49432E-19 |
| ENSMUSG00000032322 | 2.893 | 20.30766667 | 2.811386256 | 2.29592E-39 | 9.49498E-41 |
| ENSMUSG00000050721 | 7.780666667 | 53.46333333 | 2.78058411 | 1.2531E-151 | 1.2026E-153 |
| ENSMUSG00000043017 | 5.315 | 36.41933333 | 2.776562917 | 3.7585E-138 | 4.1259E-140 |
| ENSMUSG00000020101 | 0.097 | 0.661333333 | 2.769320967 | 0.002925439 | 0.001052302 |
| ENSMUSG00000002104 | 2.024333333 | 13.738 | 2.762653216 | 2.05269E-37 | 8.94259E-39 |
| ENSMUSG00000032643 | 6.48 | 43.39733333 | 2.743540677 | 4.4848E-112 | 6.3166E-114 |
| ENSMUSG00000030342 | 0.102333333 | 0.681666667 | 2.735790282 | 0.01114526 | 0.004470724 |
| ENSMUSG00000110206 | 4.137666667 | 27.543 | 2.734796377 | 1.45403E-49 | 4.63794E-51 |
| ENSMUSG00000031078 | 0.048666667 | 0.322666667 | 2.729038678 | 0.01151247 | 0.004630741 |
| ENSMUSG00000032366 | 23.66766667 | 156.8653333 | 2.728537171 | 7.6782E-204 | 5.0361E-206 |
| ENSMUSG00000030539 | 4.032 | 26.66166667 | 2.725199425 | 6.5512E-106 | 9.7698E-108 |
| ENSMUSG00000021876 | 0.393 | 2.596333333 | 2.7238744 | 3.11514E-09 | 5.34245E-10 |
| ENSMUSG00000026749 | 4.105666667 | 26.98866667 | 2.716665298 | 5.31311E-61 | 1.36092E-62 |
| ENSMUSG00000068227 | 0.308333333 | 2.010333333 | 2.704871964 | 1.55724E-10 | 2.40717E-11 |
| ENSMUSG00000098112 | 37.33433333 | 243.0506667 | 2.702682217 | 1.553E-252 | 7.0765E-255 |
| ENSMUSG00000059900 | 3.718333333 | 24.088 | 2.6955866 | 6.55165E-67 | 1.5289E-68 |
| ENSMUSG00000027368 | 1.28 | 8.276666667 | 2.692906045 | 6.93428E-24 | 4.89287E-25 |
| ENSMUSG00000040479 | 16.57866667 | 106.713 | 2.686336051 | 5.9849E-292 | 1.9834E-294 |
| ENSMUSG00000074305 | 6.839 | 43.98233333 | 2.685066849 | 9.3204E-256 | 3.9897E-258 |
| ENSMUSG00000033717 | 0.044333333 | 0.284666667 | 2.682809824 | 0.002256992 | 0.000794248 |
| ENSMUSG00000001473 | 8.156 | 52.35133333 | 2.682292601 | 5.88738E-96 | 9.71474E-98 |
| ENSMUSG00000029442 | 0.776333333 | 4.977333333 | 2.680624869 | 5.26016E-20 | 4.43794E-21 |
| ENSMUSG00000045827 | 0.480333333 | 3.062333333 | 2.672523493 | 1.53592E-19 | 1.32871E-20 |
| ENSMUSG00000032595 | 0.043 | 0.271666667 | 2.659428994 | 0.026080521 | 0.011394472 |
| ENSMUSG00000031778 | 0.291 | 1.820666667 | 2.645375755 | 1.18724E-11 | 1.65987E-12 |
| ENSMUSG00000032413 | 3.474666667 | 21.71666667 | 2.64385619 | 1.1768E-148 | 1.1537E-150 |
| ENSMUSG00000022148 | 37.89933333 | 235.5413333 | 2.635735873 | 1.9941E-290 | 6.8839E-293 |
| ENSMUSG00000031497 | 1.335 | 8.184666667 | 2.61608392 | 2.47486E-27 | 1.49852E-28 |

| | | | | | |
|---|---|---|---|---|---|
| ENSMUSG00000074364 | 10.401 | 63.54666667 | 2.611094208 | 1.0022E-151 | 9.5492E-154 |
| ENSMUSG00000079492 | 0.292333333 | 1.778333333 | 2.604839523 | 6.5452E-06 | 1.59156E-06 |
| ENSMUSG00000038172 | 3.781666667 | 22.92666667 | 2.599932507 | 1.1008E-120 | 1.3832E-122 |
| ENSMUSG00000022014 | 1.067666667 | 6.47 | 2.599304415 | 9.73302E-25 | 6.53841E-26 |
| ENSMUSG00000042842 | 0.732666667 | 4.408 | 2.588895338 | 5.58999E-19 | 4.95164E-20 |
| ENSMUSG00000038517 | 3.573333333 | 21.431 | 2.584356765 | 9.05916E-81 | 1.7638E-82 |
| ENSMUSG00000047953 | 0.213333333 | 1.275333333 | 2.579693063 | 1.1944E-06 | 2.66632E-07 |
| ENSMUSG00000027111 | 1.477333333 | 8.811 | 2.576310386 | 4.56867E-08 | 8.72161E-09 |
| ENSMUSG00000079481 | 0.081333333 | 0.484 | 2.5730884 | 2.35774E-10 | 3.69841E-11 |
| ENSMUSG00000037336 | 0.603666667 | 3.568666667 | 2.563561106 | 5.63067E-16 | 5.88181E-17 |
| ENSMUSG00000030555 | 0.287333333 | 1.692666667 | 2.558500621 | 7.18476E-06 | 1.75551E-06 |
| ENSMUSG00000027314 | 0.098333333 | 0.565 | 2.522498414 | 2.14179E-05 | 5.55853E-06 |
| ENSMUSG00000040283 | 5.063333333 | 29.038 | 2.519782722 | 9.7983E-127 | 1.1433E-128 |
| ENSMUSG00000022037 | 34.267 | 196.48 | 2.519490668 | 1.7897E-167 | 1.5569E-169 |
| ENSMUSG00000039005 | 1.574666667 | 9.025 | 2.518880468 | 3.47649E-50 | 1.0921E-51 |
| ENSMUSG00000054150 | 8.426 | 48.19133333 | 2.515853896 | 8.8215E-146 | 9.014E-148 |
| ENSMUSG00000112023 | 4.159333333 | 23.49833333 | 2.498134221 | 4.05546E-38 | 1.73597E-39 |
| ENSMUSG00000059895 | 46.43733333 | 261.726 | 2.494700217 | 4.3023E-122 | 5.317E-124 |
| ENSMUSG00000039431 | 0.754666667 | 4.225 | 2.485039884 | 3.37599E-21 | 2.6828E-22 |
| ENSMUSG00000040350 | 0.341 | 1.909 | 2.484973458 | 0.023566306 | 0.010196772 |
| ENSMUSG00000000184 | 26.86733333 | 149.3186667 | 2.47446949 | 5.1637E-110 | 7.3441E-112 |
| ENSMUSG00000027068 | 0.206666667 | 1.143666667 | 2.468289005 | 3.73026E-05 | 9.99785E-06 |
| ENSMUSG00000031302 | 0.041333333 | 0.228666667 | 2.467868456 | 0.045760196 | 0.02123723 |
| ENSMUSG00000006494 | 2.376333333 | 13.07966667 | 2.460516649 | 2.24789E-73 | 4.84217E-75 |
| ENSMUSG00000011263 | 0.090666667 | 0.493333333 | 2.443918619 | 0.005223058 | 0.001969645 |
| ENSMUSG00000074151 | 1.098333333 | 5.955 | 2.438785544 | 9.47163E-43 | 3.61628E-44 |
| ENSMUSG00000045092 | 1.052333333 | 5.704 | 2.438382222 | 2.98293E-31 | 1.58167E-32 |
| ENSMUSG00000028465 | 103.3193333 | 557.5326667 | 2.431946098 | 1.7739E-259 | 7.2261E-262 |
| ENSMUSG00000027078 | 2.588666667 | 13.94133333 | 2.429087434 | 8.10436E-22 | 6.21648E-23 |
| ENSMUSG00000030589 | 0.201 | 1.082333333 | 2.428877477 | 3.66903E-08 | 6.93833E-09 |
| ENSMUSG00000040498 | 0.055333333 | 0.297666667 | 2.427476934 | 0.043551541 | 0.020083784 |
| ENSMUSG00000006764 | 0.062333333 | 0.333666667 | 2.420331799 | 0.027681123 | 0.012151103 |

| | | | | | |
|---|---|---|---|---|---|
| ENSMUSG00000030144 | 2.137333333 | 11.43933333 | 2.420119147 | 2.74394E-15 | 2.98568E-16 |
| ENSMUSG00000075415 | 27.47833333 | 146.664 | 2.416148385 | 3.2914E-253 | 1.4771E-255 |
| ENSMUSG00000053310 | 4.764333333 | 25.36133333 | 2.412284336 | 4.78272E-43 | 1.80293E-44 |
| ENSMUSG00000021823 | 29.02333333 | 153.2853333 | 2.400934536 | 1.4074E-236 | 7.2879E-239 |
| ENSMUSG00000034107 | 0.109 | 0.575333333 | 2.400069924 | 0.005112646 | 0.001923773 |
| ENSMUSG00000059456 | 9.863666667 | 51.537 | 2.385412608 | 2.0606E-109 | 2.9733E-111 |
| ENSMUSG00000026657 | 3.013333333 | 15.73566667 | 2.384606125 | 1.0289E-113 | 1.3995E-115 |
| ENSMUSG00000037966 | 6.500666667 | 33.76466667 | 2.376854731 | 3.09908E-53 | 9.13634E-55 |
| ENSMUSG00000053113 | 1.703333333 | 8.840666667 | 2.375794376 | 1.15007E-25 | 7.39242E-27 |
| ENSMUSG00000096472 | 5.559 | 28.82866667 | 2.374606826 | 2.44352E-54 | 7.0856E-56 |
| ENSMUSG00000040253 | 0.451666667 | 2.339333333 | 2.372767096 | 1.85224E-15 | 1.99623E-16 |
| ENSMUSG00000052632 | 1.203333333 | 6.232333333 | 2.372736061 | 1.40187E-06 | 3.16205E-07 |
| ENSMUSG00000030032 | 0.163333333 | 0.838666667 | 2.360278268 | 3.38639E-08 | 6.3828E-09 |
| ENSMUSG00000070690 | 0.622666667 | 3.154666667 | 2.340955619 | 5.60125E-14 | 6.63224E-15 |
| ENSMUSG00000014158 | 0.04 | 0.200333333 | 2.324330585 | 0.042067273 | 0.019293903 |
| ENSMUSG00000025701 | 0.245666667 | 1.230333333 | 2.324275213 | 1.05353E-06 | 2.32977E-07 |
| ENSMUSG00000031342 | 0.174 | 0.869666667 | 2.321375232 | 6.00302E-06 | 1.45268E-06 |
| ENSMUSG00000027397 | 9.072 | 45.289 | 2.319668139 | 3.42323E-66 | 8.03575E-68 |
| ENSMUSG00000044367 | 0.82 | 4.092 | 2.31911033 | 1.35122E-19 | 1.16148E-20 |
| ENSMUSG00000038473 | 0.409666667 | 2.034 | 2.311797264 | 2.04855E-07 | 4.15961E-08 |
| ENSMUSG00000031827 | 52.00166667 | 256.5523333 | 2.302623379 | 6.6267E-113 | 9.0589E-115 |
| ENSMUSG00000037824 | 7.076 | 34.36033333 | 2.279738079 | 1.27354E-92 | 2.17181E-94 |
| ENSMUSG00000052142 | 11.63733333 | 56.391 | 2.276704421 | 7.0583E-139 | 7.6996E-141 |
| ENSMUSG00000045671 | 6.152666667 | 29.807 | 2.27636744 | 1.54758E-71 | 3.38706E-73 |
| ENSMUSG00000026180 | 2.351666667 | 11.326 | 2.267882948 | 9.47971E-21 | 7.68377E-22 |
| ENSMUSG00000055322 | 5.494666667 | 26.45466667 | 2.267418372 | 2.7076E-216 | 1.5703E-218 |
| ENSMUSG00000102752 | 0.337666667 | 1.621 | 2.263212417 | 6.24233E-12 | 8.52911E-13 |
| ENSMUSG00000030041 | 0.723 | 3.463 | 2.259954835 | 4.33344E-09 | 7.52459E-10 |
| ENSMUSG00000030263 | 11.379 | 54.25966667 | 2.253506407 | 2.31462E-98 | 3.73944E-100 |
| ENSMUSG00000040061 | 16.13166667 | 76.69166667 | 2.249174322 | 4.4656E-248 | 2.189E-250 |
| ENSMUSG00000053835 | 0.652333333 | 3.088666667 | 2.243302926 | 2.48398E-09 | 4.22057E-10 |
| ENSMUSG00000061143 | 2.085333333 | 9.860333333 | 2.241358407 | 2.30173E-81 | 4.43374E-83 |

| | | | | | |
|---|---|---|---|---|---|
| ENSMUSG00000074918 | 9.844333333 | 46.49666667 | 2.23976188 | 2.18938E-67 | 5.04869E-69 |
| ENSMUSG00000049493 | 0.055333333 | 0.26 | 2.232290882 | 0.020358692 | 0.00867395 |
| ENSMUSG00000047045 | 14.70066667 | 69.051 | 2.231780726 | 3.224E-127 | 3.7395E-129 |
| ENSMUSG00000037946 | 2.102333333 | 9.860666667 | 2.229693756 | 2.15105E-46 | 7.41077E-48 |
| ENSMUSG00000024887 | 1.663333333 | 7.796 | 2.228656776 | 1.02296E-47 | 3.41128E-49 |
| ENSMUSG00000028300 | 0.566 | 2.643666667 | 2.223666324 | 3.89212E-14 | 4.54671E-15 |
| ENSMUSG00000046806 | 3.287 | 15.33933333 | 2.222392421 | 2.02189E-74 | 4.25764E-76 |
| ENSMUSG00000027296 | 0.112666667 | 0.525666667 | 2.222087509 | 0.022222095 | 0.009556851 |
| ENSMUSG00000042228 | 17.62533333 | 82.04433333 | 2.21875315 | 3.1894E-189 | 2.2901E-191 |
| ENSMUSG00000052837 | 9.869333333 | 45.845 | 2.215739858 | 6.24218E-74 | 1.32739E-75 |
| ENSMUSG00000003545 | 1.718333333 | 7.906333333 | 2.201998854 | 3.08199E-14 | 3.58119E-15 |
| ENSMUSG00000039063 | 0.719666667 | 3.306 | 2.199685982 | 2.48672E-12 | 3.29124E-13 |
| ENSMUSG00000052353 | 0.019666667 | 0.090333333 | 2.199505992 | 0.044727608 | 0.020680394 |
| ENSMUSG00000005686 | 2.170333333 | 9.961666667 | 2.198470499 | 2.48088E-36 | 1.1339E-37 |
| ENSMUSG00000018909 | 4.955 | 22.70466667 | 2.196031894 | 1.1931E-118 | 1.5404E-120 |
| ENSMUSG00000055202 | 0.192 | 0.879666667 | 2.195850634 | 7.94732E-05 | 2.22661E-05 |
| ENSMUSG00000042622 | 0.769666667 | 3.518 | 2.19244981 | 1.29451E-09 | 2.16199E-10 |
| ENSMUSG00000038205 | 0.986333333 | 4.491333333 | 2.186996603 | 2.368E-23 | 1.70684E-24 |
| ENSMUSG00000025492 | 22.428 | 102.1 | 2.186609986 | 1.90415E-80 | 3.73363E-82 |
| ENSMUSG00000044551 | 1.522666667 | 6.922666667 | 2.184727733 | 3.25934E-23 | 2.35832E-24 |
| ENSMUSG00000035697 | 22.167 | 100.6593333 | 2.182995509 | 2.0804E-210 | 1.2783E-212 |
| ENSMUSG00000047798 | 1.535333333 | 6.960666667 | 2.180673578 | 6.33127E-17 | 6.23335E-18 |
| ENSMUSG00000091780 | 4.383666667 | 19.821 | 2.176819745 | 2.90456E-32 | 1.48998E-33 |
| ENSMUSG00000035783 | 5.176333333 | 23.35733333 | 2.173873145 | 1.18036E-37 | 5.11784E-39 |
| ENSMUSG00000037679 | 9.409666667 | 42.36666667 | 2.170714102 | 3.70802E-87 | 6.68181E-89 |
| ENSMUSG00000018166 | 8.932 | 40.17966667 | 2.169410439 | 1.63361E-90 | 2.84223E-92 |
| ENSMUSG00000020902 | 1.902 | 8.545 | 2.167563246 | 1.04401E-58 | 2.73184E-60 |
| ENSMUSG00000000823 | 5.337333333 | 23.84666667 | 2.159596597 | 1.04254E-69 | 2.31051E-71 |
| ENSMUSG00000069919 | 0.44 | 1.960333333 | 2.155523561 | 0.003487927 | 0.001271971 |
| ENSMUSG00000031557 | 21.45966667 | 95.096 | 2.147756992 | 6.36957E-64 | 1.54798E-65 |
| ENSMUSG00000006587 | 0.203666667 | 0.9 | 2.143715122 | 0.002460978 | 0.000872893 |
| ENSMUSG00000046688 | 1.622666667 | 7.112333333 | 2.131956273 | 1.08872E-20 | 8.86973E-22 |

| | | | | | |
|---|---|---|---|---|---|
| ENSMUSG00000030782 | 0.138666667 | 0.607 | 2.130075489 | 0.001532947 | 0.000523577 |
| ENSMUSG00000036931 | 0.881666667 | 3.856333333 | 2.128924539 | 2.08197E-12 | 2.73974E-13 |
| ENSMUSG00000062380 | 0.070333333 | 0.306666667 | 2.124390862 | 0.042797752 | 0.01968212 |
| ENSMUSG00000033306 | 2.084 | 9.053 | 2.119040677 | 2.3088E-89 | 4.06479E-91 |
| ENSMUSG00000022456 | 0.662333333 | 2.866666667 | 2.113744787 | 1.02798E-21 | 7.92774E-23 |
| ENSMUSG00000060216 | 36.29 | 157.0656667 | 2.113723891 | 2.9882E-155 | 2.7646E-157 |
| ENSMUSG00000039968 | 11.66666667 | 50.43666667 | 2.112080511 | 3.1056E-125 | 3.7522E-127 |
| ENSMUSG00000022094 | 4.172666667 | 17.92433333 | 2.102877881 | 7.73978E-82 | 1.4802E-83 |
| ENSMUSG00000063851 | 0.487666667 | 2.094 | 2.102294174 | 0.000582731 | 0.00018507 |
| ENSMUSG00000040483 | 0.319333333 | 1.368 | 2.09893317 | 0.001676244 | 0.000575413 |
| ENSMUSG00000039987 | 3.489666667 | 14.77333333 | 2.081834239 | 1.01102E-67 | 2.31744E-69 |
| ENSMUSG00000020604 | 0.348 | 1.473 | 2.081598219 | 8.64295E-08 | 1.69649E-08 |
| ENSMUSG00000038011 | 0.018 | 0.075666667 | 2.071660985 | 0.04448299 | 0.020552345 |
| ENSMUSG00000047419 | 0.359 | 1.507 | 2.069623668 | 9.26328E-19 | 8.2886E-20 |
| ENSMUSG00000020527 | 2.63 | 11.03766667 | 2.069300518 | 7.44158E-39 | 3.12892E-40 |
| ENSMUSG00000061859 | 2.525666667 | 10.53566667 | 2.060545454 | 5.64831E-21 | 4.53144E-22 |
| ENSMUSG00000038712 | 15.221 | 63.46733333 | 2.059951082 | 7.9583E-132 | 9.011E-134 |
| ENSMUSG00000028339 | 1.121666667 | 4.661 | 2.054995509 | 2.86446E-29 | 1.61773E-30 |
| ENSMUSG00000034738 | 1.818 | 7.529 | 2.05010606 | 2.82176E-18 | 2.57745E-19 |
| ENSMUSG00000052681 | 68.61566667 | 283.4276667 | 2.046370671 | 6.95848E-76 | 1.43647E-77 |
| ENSMUSG00000002997 | 31.75866667 | 130.8683333 | 2.042893796 | 2.3639E-115 | 3.1825E-117 |
| ENSMUSG00000032373 | 3.794666667 | 15.53833333 | 2.0337867 | 3.20805E-56 | 8.79311E-58 |
| ENSMUSG00000000791 | 1.850333333 | 7.567 | 2.031936253 | 3.7292E-33 | 1.86924E-34 |
| ENSMUSG00000045838 | 0.918333333 | 3.742333333 | 2.026848249 | 3.22569E-24 | 2.23598E-25 |
| ENSMUSG00000031749 | 2.864 | 11.663 | 2.025835534 | 5.83283E-52 | 1.784E-53 |
| ENSMUSG00000060600 | 13.20166667 | 53.55166667 | 2.0202114 | 3.40605E-55 | 9.571E-57 |
| ENSMUSG00000056515 | 17.62933333 | 71.167 | 2.013230502 | 2.7942E-108 | 4.0899E-110 |
| ENSMUSG00000028970 | 2.770333333 | 11.16333333 | 2.010636395 | 4.47128E-57 | 1.20395E-58 |
| ENSMUSG00000016552 | 0.079333333 | 0.319666667 | 2.010569242 | 0.001423304 | 0.000482689 |
| ENSMUSG00000034758 | 1.428666667 | 5.752666667 | 2.009561528 | 1.39261E-16 | 1.40087E-17 |
| ENSMUSG00000024236 | 10.35566667 | 41.66833333 | 2.008530963 | 1.66E-177 | 1.318E-179 |
| ENSMUSG00000039031 | 6.893333333 | 27.61966667 | 2.002422223 | 7.79451E-80 | 1.5391E-81 |

| | | | | | |
|---|---|---|---|---|---|
| ENSMUSG00000037706 | 3.151333333 | 12.52933333 | 1.991275384 | 3.05609E-21 | 2.42014E-22 |
| ENSMUSG00000029265 | 37.265 | 147.1696667 | 1.981587181 | 4.464E-174 | 3.606E-176 |
| ENSMUSG00000014606 | 46.905 | 185.2196667 | 1.981423667 | 4.8266E-147 | 4.7986E-149 |
| ENSMUSG00000013707 | 3.123666667 | 12.332 | 1.981094381 | 1.73869E-16 | 1.75622E-17 |
| ENSMUSG00000001227 | 2.271666667 | 8.937333333 | 1.976093277 | 7.22151E-38 | 3.1012E-39 |
| ENSMUSG00000054931 | 0.341333333 | 1.337333333 | 1.970105891 | 7.62271E-06 | 1.86989E-06 |
| ENSMUSG00000018199 | 2.558333333 | 10.01833333 | 1.969366364 | 6.55679E-69 | 1.46672E-70 |
| ENSMUSG00000042751 | 0.273333333 | 1.07 | 1.968877483 | 1.08471E-06 | 2.40398E-07 |
| ENSMUSG00000029370 | 0.108333333 | 0.423333333 | 1.966316874 | 0.035798356 | 0.016146828 |
| ENSMUSG00000024912 | 0.648 | 2.530333333 | 1.965261733 | 3.22113E-07 | 6.66509E-08 |
| ENSMUSG00000041920 | 13.942 | 54.43033333 | 1.964973338 | 2.4249E-109 | 3.5325E-111 |
| ENSMUSG00000097075 | 0.343333333 | 1.340333333 | 1.964909999 | 0.007103322 | 0.002747363 |
| ENSMUSG00000044229 | 0.463 | 1.806333333 | 1.963980048 | 1.22726E-08 | 2.22252E-09 |
| ENSMUSG00000074923 | 1.055 | 4.111 | 1.962246373 | 9.82337E-18 | 9.24417E-19 |
| ENSMUSG00000028771 | 26.558 | 103.3986667 | 1.960999171 | 3.975E-206 | 2.4974E-208 |
| ENSMUSG00000015745 | 10.80566667 | 41.86466667 | 1.953945055 | 2.78912E-41 | 1.09762E-42 |
| ENSMUSG00000031955 | 0.512666667 | 1.980333333 | 1.949650285 | 4.6913E-10 | 7.57916E-11 |
| ENSMUSG00000036622 | 7.480333333 | 28.863 | 1.948046795 | 1.75716E-61 | 4.47662E-63 |
| ENSMUSG00000079625 | 0.196 | 0.755333333 | 1.946259801 | 0.019448028 | 0.008240941 |
| ENSMUSG00000037681 | 0.045 | 0.173333333 | 1.945552216 | 0.045679312 | 0.021190231 |
| ENSMUSG00000005609 | 39.47933333 | 151.9703333 | 1.944620184 | 3.77018E-92 | 6.45543E-94 |
| ENSMUSG00000026566 | 27.36533333 | 105.0666667 | 1.940883699 | 4.3113E-147 | 4.2565E-149 |
| ENSMUSG00000094347 | 44.856 | 171.521 | 1.935012342 | 4.37428E-93 | 7.39919E-95 |
| ENSMUSG00000040533 | 0.305333333 | 1.156 | 1.920684395 | 0.002450452 | 0.000868587 |
| ENSMUSG00000024381 | 3.835333333 | 14.49833333 | 1.918463189 | 5.41162E-33 | 2.72748E-34 |
| ENSMUSG00000041220 | 7.843666667 | 29.644 | 1.918139999 | 8.2241E-113 | 1.1356E-114 |
| ENSMUSG00000059430 | 23.752 | 89.516 | 1.914096573 | 5.35173E-82 | 1.0198E-83 |
| ENSMUSG00000074802 | 3.829 | 14.34766667 | 1.905776568 | 2.8314E-39 | 1.17291E-40 |
| ENSMUSG00000028063 | 10.85966667 | 40.61433333 | 1.903009143 | 8.50156E-62 | 2.14828E-63 |
| ENSMUSG00000001128 | 0.554 | 2.071 | 1.902369672 | 5.64995E-05 | 1.55838E-05 |
| ENSMUSG00000050014 | 1.523666667 | 5.591666667 | 1.875731042 | 2.13235E-16 | 2.16578E-17 |
| ENSMUSG00000043881 | 0.139666667 | 0.511 | 1.871335548 | 0.000180435 | 5.31688E-05 |

| | | | | | |
|---|---|---|---|---|---|
| ENSMUSG00000032177 | 5.270333333 | 19.18133333 | 1.863736892 | 4.32151E-46 | 1.49779E-47 |
| ENSMUSG00000020788 | 49.502 | 177.6186667 | 1.843224488 | 2.813E-139 | 3.0297E-141 |
| ENSMUSG00000030123 | 10.47133333 | 37.51566667 | 1.841048041 | 8.52939E-79 | 1.70776E-80 |
| ENSMUSG00000030788 | 11.19466667 | 40.06466667 | 1.839518903 | 1.05817E-70 | 2.33055E-72 |
| ENSMUSG00000079184 | 5.480333333 | 19.56633333 | 1.836037874 | 1.24838E-49 | 3.96474E-51 |
| ENSMUSG00000031785 | 80.31666667 | 286.737 | 1.835956779 | 6.70542E-47 | 2.29162E-48 |
| ENSMUSG00000071669 | 0.895666667 | 3.195666667 | 1.835083111 | 1.77851E-07 | 3.59534E-08 |
| ENSMUSG00000074785 | 0.456333333 | 1.619 | 1.82694304 | 1.22203E-10 | 1.86291E-11 |
| ENSMUSG00000034595 | 23.21733333 | 82.309 | 1.825847911 | 5.4237E-143 | 5.7292E-145 |
| ENSMUSG00000039699 | 0.527 | 1.861666667 | 1.820719913 | 0.001583627 | 0.000541762 |
| ENSMUSG00000039191 | 24.63333333 | 86.537 | 1.812705244 | 3.7183E-119 | 4.775E-121 |
| ENSMUSG00000075706 | 58.95533333 | 206.3186667 | 1.807180119 | 5.59415E-66 | 1.3209E-67 |
| ENSMUSG00000038563 | 8.664 | 30.29066667 | 1.805768183 | 2.59543E-96 | 4.26478E-98 |
| ENSMUSG00000026727 | 50.13 | 175.028 | 1.803839596 | 5.4781E-103 | 8.4721E-105 |
| ENSMUSG00000006641 | 3.02 | 10.54066667 | 1.803345661 | 9.77308E-16 | 1.03102E-16 |
| ENSMUSG00000049916 | 3.54 | 12.314 | 1.798478208 | 2.25683E-16 | 2.29671E-17 |
| ENSMUSG00000022791 | 15.899 | 55.217 | 1.79617648 | 3.33392E-47 | 1.12788E-48 |
| ENSMUSG00000050914 | 1.419666667 | 4.922333333 | 1.793790129 | 9.80849E-05 | 2.78733E-05 |
| ENSMUSG00000049225 | 15.88633333 | 55.037 | 1.792615653 | 1.2573E-09 | 2.09723E-10 |
| ENSMUSG00000023031 | 3.055 | 10.555 | 1.788682294 | 1.51245E-14 | 1.71879E-15 |
| ENSMUSG00000002603 | 69.94066667 | 241.4503333 | 1.787523002 | 3.98128E-56 | 1.09675E-57 |
| ENSMUSG00000021322 | 0.245333333 | 0.845333333 | 1.784777074 | 0.00151268 | 0.000516028 |
| ENSMUSG00000054404 | 0.894333333 | 3.074333333 | 1.781389043 | 1.19987E-10 | 1.82665E-11 |
| ENSMUSG00000025006 | 9.193666667 | 31.55833333 | 1.779308751 | 1.22843E-45 | 4.30003E-47 |
| ENSMUSG00000031355 | 19.49333333 | 66.77366667 | 1.776298453 | 1.86802E-87 | 3.35325E-89 |
| ENSMUSG00000030494 | 0.140666667 | 0.481333333 | 1.774755838 | 0.006898079 | 0.002661313 |
| ENSMUSG00000028885 | 1.419666667 | 4.838666667 | 1.769057327 | 1.6674E-13 | 2.03072E-14 |
| ENSMUSG00000024300 | 1.008333333 | 3.434666667 | 1.768197451 | 2.9111E-16 | 2.98667E-17 |
| ENSMUSG00000019920 | 21.24333333 | 72.34 | 1.767783438 | 4.78989E-64 | 1.16076E-65 |
| ENSMUSG00000025477 | 5.818 | 19.8 | 1.766905229 | 4.3599E-49 | 1.40875E-50 |
| ENSMUSG00000078853 | 0.548666667 | 1.861666667 | 1.762592945 | 3.19966E-05 | 8.48956E-06 |
| ENSMUSG00000045216 | 8.578333333 | 29.05033333 | 1.759785436 | 3.6971E-32 | 1.9093E-33 |

| | | | | | |
|---|---|---|---|---|---|
| ENSMUSG00000025986 | 5.198 | 17.54833333 | 1.755305477 | 4.6569E-105 | 7.1056E-107 |
| ENSMUSG00000022255 | 63.24166667 | 213.221 | 1.753402241 | 3.6013E-184 | 2.7102E-186 |
| ENSMUSG00000021549 | 11.41433333 | 38.39166667 | 1.749946592 | 1.27224E-40 | 5.10336E-42 |
| ENSMUSG00000030096 | 7.984333333 | 26.75166667 | 1.744384919 | 5.38906E-66 | 1.26876E-67 |
| ENSMUSG00000020178 | 8.370666667 | 28.00233333 | 1.742132613 | 2.41674E-42 | 9.29385E-44 |
| ENSMUSG00000027195 | 25.71633333 | 85.696 | 1.73654291 | 7.87354E-86 | 1.44055E-87 |
| ENSMUSG00000105867 | 0.475333333 | 1.583666667 | 1.736257225 | 0.000144179 | 4.19079E-05 |
| ENSMUSG00000071113 | 0.202333333 | 0.671333333 | 1.730295262 | 0.013761202 | 0.005634074 |
| ENSMUSG00000057729 | 0.673333333 | 2.228 | 1.72635644 | 0.004232067 | 0.001569056 |
| ENSMUSG00000032318 | 0.270666667 | 0.890333333 | 1.717828343 | 0.048448195 | 0.022608489 |
| ENSMUSG00000050592 | 4.089666667 | 13.40833333 | 1.713074756 | 8.85632E-53 | 2.65983E-54 |
| ENSMUSG00000047996 | 2.006 | 6.571333333 | 1.711864519 | 1.1402E-15 | 1.20837E-16 |
| ENSMUSG00000029406 | 1.754333333 | 5.742 | 1.710630436 | 2.5371E-27 | 1.53796E-28 |
| ENSMUSG00000031328 | 77.68866667 | 252.181 | 1.698683527 | 3.8397E-223 | 2.0943E-225 |
| ENSMUSG00000060579 | 0.727666667 | 2.361 | 1.698048412 | 0.003864569 | 0.001422932 |
| ENSMUSG00000022106 | 1.924 | 6.242333333 | 1.697976598 | 1.3155E-10 | 2.01903E-11 |
| ENSMUSG00000044447 | 4.457666667 | 14.46233333 | 1.697939689 | 2.99581E-55 | 8.39752E-57 |
| ENSMUSG00000052485 | 0.376666667 | 1.22 | 1.695520876 | 0.013631734 | 0.005569774 |
| ENSMUSG00000023036 | 1.289666667 | 4.172666667 | 1.693971448 | 1.87752E-07 | 3.80326E-08 |
| ENSMUSG00000019102 | 0.956333333 | 3.089 | 1.691554404 | 3.15964E-05 | 8.37029E-06 |
| ENSMUSG00000037446 | 7.556333333 | 24.38666667 | 1.690334324 | 1.74847E-54 | 5.04597E-56 |
| ENSMUSG00000031938 | 5.594 | 18.03333333 | 1.688713934 | 6.50821E-31 | 3.48237E-32 |
| ENSMUSG00000032966 | 56.83533333 | 183.2133333 | 1.688664492 | 5.9916E-116 | 7.9425E-118 |
| ENSMUSG00000041406 | 16.69833333 | 53.732 | 1.686077425 | 4.25626E-67 | 9.90306E-69 |
| ENSMUSG00000026784 | 3.908 | 12.57466667 | 1.686017785 | 3.11921E-20 | 2.58212E-21 |
| ENSMUSG00000059429 | 0.259 | 0.832333333 | 1.684209318 | 1.38106E-06 | 3.11225E-07 |
| ENSMUSG00000022696 | 0.071 | 0.227 | 1.676801368 | 0.031431639 | 0.013975404 |
| ENSMUSG00000023905 | 2.475333333 | 7.888 | 1.672036737 | 2.53062E-09 | 4.30506E-10 |
| ENSMUSG00000031562 | 4.347 | 13.82633333 | 1.669326612 | 1.38824E-23 | 9.94888E-25 |
| ENSMUSG00000031163 | 0.604 | 1.920333333 | 1.668736302 | 0.032291444 | 0.014406746 |
| ENSMUSG00000050957 | 4.062666667 | 12.91666667 | 1.668734905 | 3.7758E-11 | 5.50572E-12 |
| ENSMUSG00000054342 | 8.337666667 | 26.32133333 | 1.658516971 | 1.29195E-47 | 4.32614E-49 |

| | | | | | |
|---|---|---|---|---|---|
| ENSMUSG00000001918 | 15.48766667 | 48.84866667 | 1.657199376 | 4.5723E-42 | 1.7678E-43 |
| ENSMUSG00000020134 | 8.26 | 25.992 | 1.653853962 | 6.30588E-44 | 2.31616E-45 |
| ENSMUSG00000009687 | 79.99533333 | 251.369 | 1.651818995 | 5.55283E-81 | 1.07729E-82 |
| ENSMUSG00000006262 | 11.118 | 34.72033333 | 1.642883511 | 7.3737E-103 | 1.1455E-104 |
| ENSMUSG00000032913 | 4.3 | 13.42266667 | 1.642262754 | 5.62692E-37 | 2.51354E-38 |
| ENSMUSG00000025494 | 3.759666667 | 11.73366667 | 1.64197725 | 1.8584E-24 | 1.27281E-25 |
| ENSMUSG00000021948 | 21.44 | 66.90166667 | 1.641737246 | 2.157E-112 | 3.0231E-114 |
| ENSMUSG00000050965 | 29.07033333 | 90.58433333 | 1.639713942 | 1.0112E-124 | 1.2288E-126 |
| ENSMUSG00000041396 | 4.901 | 15.207 | 1.633587518 | 1.33665E-20 | 1.09357E-21 |
| ENSMUSG00000024589 | 15.32166667 | 47.527 | 1.633174099 | 3.45984E-58 | 9.12496E-60 |
| ENSMUSG00000047986 | 0.313333333 | 0.971666667 | 1.632763222 | 0.006065037 | 0.002309772 |
| ENSMUSG00000050912 | 54.791 | 169.7183333 | 1.631131576 | 1.3534E-106 | 2.0089E-108 |
| ENSMUSG00000059248 | 27.425 | 84.72733333 | 1.627335842 | 4.1584E-59 | 1.08525E-60 |
| ENSMUSG00000032289 | 1.398333333 | 4.307666667 | 1.623198306 | 9.56845E-35 | 4.5649E-36 |
| ENSMUSG00000034187 | 30.95066667 | 95.31666667 | 1.622758015 | 2.313E-157 | 2.108E-159 |
| ENSMUSG00000027935 | 3.009333333 | 9.265333333 | 1.622398962 | 2.35674E-11 | 3.38769E-12 |
| ENSMUSG00000036461 | 48.46066667 | 149.0896667 | 1.621294112 | 1.34891E-93 | 2.26309E-95 |
| ENSMUSG00000003348 | 10.06566667 | 30.92933333 | 1.61953301 | 4.86804E-65 | 1.15954E-66 |
| ENSMUSG00000049709 | 0.701666667 | 2.156 | 1.619499446 | 4.48826E-11 | 6.59418E-12 |
| ENSMUSG00000048200 | 0.519666667 | 1.590333333 | 1.613670758 | 0.00139004 | 0.000470715 |
| ENSMUSG00000030281 | 4.13 | 12.63666667 | 1.613402269 | 2.37136E-17 | 2.29703E-18 |
| ENSMUSG00000044017 | 0.063 | 0.192666667 | 1.612683258 | 0.033316764 | 0.014903294 |
| ENSMUSG00000025036 | 1.175666667 | 3.587333333 | 1.609432731 | 2.40615E-13 | 2.96865E-14 |
| ENSMUSG00000041836 | 13.299 | 40.57366667 | 1.609225917 | 1.03899E-40 | 4.15338E-42 |
| ENSMUSG00000030830 | 25.23466667 | 76.63766667 | 1.602646607 | 1.2687E-112 | 1.7606E-114 |
| ENSMUSG00000018378 | 17.05766667 | 51.73033333 | 1.600590175 | 9.87409E-68 | 2.25651E-69 |
| ENSMUSG00000039953 | 3.33 | 10.08866667 | 1.599141436 | 6.24209E-31 | 3.33566E-32 |
| ENSMUSG00000067212 | 16.18166667 | 48.954 | 1.597066537 | 1.7744E-57 | 4.7533E-59 |
| ENSMUSG00000036634 | 1.681666667 | 5.085 | 1.596356005 | 9.11967E-13 | 1.16357E-13 |
| ENSMUSG00000024036 | 6.441 | 19.47466667 | 1.596242038 | 1.10119E-19 | 9.43509E-21 |
| ENSMUSG00000006362 | 27.74733333 | 83.864 | 1.595702516 | 1.6812E-67 | 3.86523E-69 |
| ENSMUSG00000038267 | 4.802 | 14.50566667 | 1.594909293 | 5.65061E-39 | 2.36027E-40 |

| | | | | | |
|---|---|---|---|---|---|
| ENSMUSG00000033446 | 1.229666667 | 3.707333333 | 1.592114548 | 3.57816E-20 | 2.97686E-21 |
| ENSMUSG00000049775 | 583.109 | 1754.804333 | 1.589472679 | 2.75874E-48 | 9.06628E-50 |
| ENSMUSG00000034685 | 2.151333333 | 6.462666667 | 1.586898505 | 1.89978E-18 | 1.71693E-19 |
| ENSMUSG00000034765 | 3.629 | 10.892 | 1.585624926 | 2.98902E-15 | 3.26266E-16 |
| ENSMUSG00000027639 | 14.391 | 43.175 | 1.585029332 | 6.79461E-91 | 1.17747E-92 |
| ENSMUSG00000030557 | 21.46666667 | 64.32033333 | 1.583176696 | 2.42875E-99 | 3.8903E-101 |
| ENSMUSG00000024371 | 0.641666667 | 1.922 | 1.582712391 | 0.000199992 | 5.93597E-05 |
| ENSMUSG00000066721 | 0.950333333 | 2.831666667 | 1.575145908 | 8.25175E-08 | 1.61628E-08 |
| ENSMUSG00000021951 | 8.301333333 | 24.669 | 1.571284254 | 3.61452E-20 | 3.00961E-21 |
| ENSMUSG00000040433 | 13.75266667 | 40.83233333 | 1.570000625 | 5.65271E-89 | 9.99098E-91 |
| ENSMUSG00000026988 | 3.609 | 10.708 | 1.569017996 | 1.44125E-13 | 1.74633E-14 |
| ENSMUSG00000031555 | 6.923666667 | 20.54166667 | 1.568945066 | 3.86812E-56 | 1.06291E-57 |
| ENSMUSG00000030844 | 7.267666667 | 21.53366667 | 1.56702984 | 7.63105E-22 | 5.84288E-23 |
| ENSMUSG00000060063 | 29.18133333 | 86.35533333 | 1.565239478 | 9.57538E-38 | 4.13849E-39 |
| ENSMUSG00000028793 | 7.849 | 23.216 | 1.56453866 | 7.36224E-23 | 5.39816E-24 |
| ENSMUSG00000043811 | 1.160666667 | 3.428666667 | 1.562693951 | 6.02669E-08 | 1.16173E-08 |
| ENSMUSG00000037434 | 4.381 | 12.912 | 1.559380364 | 1.72676E-27 | 1.03958E-28 |
| ENSMUSG00000015714 | 52.81033333 | 155.603 | 1.558977723 | 3.9198E-126 | 4.6549E-128 |
| ENSMUSG00000039621 | 6.094666667 | 17.90666667 | 1.554877581 | 3.76659E-33 | 1.89058E-34 |
| ENSMUSG00000027015 | 0.088666667 | 0.26 | 1.552047878 | 0.032267459 | 0.01439159 |
| ENSMUSG00000028656 | 180.5756667 | 528.6796667 | 1.549790344 | 2.6892E-193 | 1.8752E-195 |
| ENSMUSG00000021608 | 9.669666667 | 28.25233333 | 1.54683196 | 7.79053E-54 | 2.28058E-55 |
| ENSMUSG00000024965 | 88.567 | 258.7513333 | 1.54672514 | 7.2067E-113 | 9.9015E-115 |
| ENSMUSG00000026519 | 6.161 | 17.981 | 1.545236817 | 1.53885E-44 | 5.56722E-46 |
| ENSMUSG00000043157 | 1.073 | 3.108666667 | 1.534645853 | 1.03131E-05 | 2.57116E-06 |
| ENSMUSG00000047921 | 8.454333333 | 24.47633333 | 1.533624551 | 4.63708E-55 | 1.31903E-56 |
| ENSMUSG00000047959 | 7.230666667 | 20.926 | 1.533095992 | 4.50801E-30 | 2.4837E-31 |
| ENSMUSG00000029580 | 919.7383333 | 2659.285667 | 1.531743387 | 7.7654E-169 | 6.5945E-171 |
| ENSMUSG00000035158 | 3.580666667 | 10.34666667 | 1.530865931 | 4.12399E-35 | 1.93615E-36 |
| ENSMUSG00000029263 | 4.394 | 12.66166667 | 1.526860546 | 4.12754E-35 | 1.94066E-36 |
| ENSMUSG00000031445 | 0.413666667 | 1.191333333 | 1.526036519 | 0.000142818 | 4.14925E-05 |
| ENSMUSG00000025921 | 3.490333333 | 10.01633333 | 1.520917751 | 9.70028E-21 | 7.86926E-22 |

| | | | | | |
|---|---|---|---|---|---|
| ENSMUSG00000074340 | 0.254333333 | 0.729333333 | 1.519857776 | 0.013858809 | 0.005680734 |
| ENSMUSG00000037286 | 22.88433333 | 65.537 | 1.51794937 | 1.2288E-55 | 3.42749E-57 |
| ENSMUSG00000046879 | 9.337666667 | 26.73066667 | 1.517361825 | 5.15739E-37 | 2.30025E-38 |
| ENSMUSG00000059182 | 30.185 | 86.28466667 | 1.515272405 | 1.31669E-61 | 3.33627E-63 |
| ENSMUSG00000022020 | 2.682666667 | 7.612666667 | 1.504734104 | 2.51778E-25 | 1.64271E-26 |
| ENSMUSG00000001366 | 21.58766667 | 61.17866667 | 1.502821349 | 1.00913E-63 | 2.46639E-65 |
| ENSMUSG00000029478 | 18.38233333 | 52.07933333 | 1.502391076 | 3.41496E-47 | 1.16001E-48 |
| ENSMUSG00000029471 | 9.671666667 | 27.386 | 1.501602133 | 3.43143E-46 | 1.18693E-47 |
| ENSMUSG00000028086 | 4.566333333 | 12.90333333 | 1.498635724 | 3.21301E-41 | 1.26666E-42 |
| ENSMUSG00000041012 | 0.485333333 | 1.369333333 | 1.496425826 | 0.020039322 | 0.008522661 |
| ENSMUSG00000015702 | 2.017666667 | 5.667666667 | 1.490067061 | 5.17703E-10 | 8.38533E-11 |
| ENSMUSG00000022075 | 1.823333333 | 5.108333333 | 1.486274336 | 7.98525E-25 | 5.35327E-26 |
| ENSMUSG00000041959 | 94.67966667 | 265.144 | 1.48564957 | 2.13673E-50 | 6.66805E-52 |
| ENSMUSG00000037628 | 5.006333333 | 13.998 | 1.483394456 | 4.32252E-13 | 5.40763E-14 |
| ENSMUSG00000032038 | 18.83266667 | 52.64566667 | 1.483077489 | 1.3404E-47 | 4.50685E-49 |
| ENSMUSG00000037656 | 6.446333333 | 17.994 | 1.480965232 | 6.70474E-33 | 3.3931E-34 |
| ENSMUSG00000018169 | 1.523666667 | 4.248333333 | 1.479349649 | 4.93965E-08 | 9.45368E-09 |
| ENSMUSG00000019866 | 2.351333333 | 6.544333333 | 1.476767158 | 4.41822E-30 | 2.43118E-31 |
| ENSMUSG00000020255 | 25.08233333 | 69.72333333 | 1.47496998 | 1.5489E-75 | 3.22955E-77 |
| ENSMUSG00000028035 | 8.556666667 | 23.775 | 1.474324546 | 3.42888E-28 | 2.00515E-29 |
| ENSMUSG00000063382 | 3.473666667 | 9.608333333 | 1.467826882 | 1.374E-16 | 1.38027E-17 |
| ENSMUSG00000032741 | 11.39333333 | 31.49333333 | 1.466856568 | 1.02205E-34 | 4.91128E-36 |
| ENSMUSG00000039013 | 2.113333333 | 5.833 | 1.464717735 | 2.53568E-14 | 2.92714E-15 |
| ENSMUSG00000048827 | 0.951333333 | 2.624333333 | 1.463928143 | 2.47239E-13 | 3.05209E-14 |
| ENSMUSG00000024968 | 8.360333333 | 23.01466667 | 1.460921177 | 2.40085E-24 | 1.65593E-25 |
| ENSMUSG00000027939 | 0.092666667 | 0.254 | 1.454706115 | 0.021444538 | 0.00918544 |
| ENSMUSG00000054027 | 3.596666667 | 9.852666667 | 1.45385379 | 4.98694E-24 | 3.48783E-25 |
| ENSMUSG00000115018 | 2.081333333 | 5.678333333 | 1.447959505 | 0.000129785 | 3.75002E-05 |
| ENSMUSG00000041895 | 6.965 | 18.99466667 | 1.447399137 | 3.42741E-28 | 2.00193E-29 |
| ENSMUSG00000026509 | 31.67566667 | 86.33566667 | 1.4465817 | 1.9512E-109 | 2.802E-111 |
| ENSMUSG00000041187 | 9.222 | 25.12433333 | 1.445933744 | 4.03317E-33 | 2.02995E-34 |
| ENSMUSG00000020534 | 16.293 | 44.30466667 | 1.443206399 | 4.48589E-51 | 1.39681E-52 |

| | | | | | |
|---|---|---|---|---|---|
| ENSMUSG00000030921 | 5.798666667 | 15.735 | 1.440184065 | 4.20758E-29 | 2.3879E-30 |
| ENSMUSG00000037752 | 0.441 | 1.196666667 | 1.440170782 | 1.84517E-05 | 4.74923E-06 |
| ENSMUSG00000022022 | 2.627333333 | 7.121666667 | 1.438615662 | 4.08375E-09 | 7.0741E-10 |
| ENSMUSG00000027634 | 31.48333333 | 85.25466667 | 1.437190511 | 5.40614E-87 | 9.81645E-89 |
| ENSMUSG00000064125 | 0.152 | 0.411 | 1.43506707 | 0.014182123 | 0.005829906 |
| ENSMUSG00000025010 | 1.754333333 | 4.737 | 1.433050778 | 2.85188E-15 | 3.10509E-16 |
| ENSMUSG00000074570 | 2.025666667 | 5.469 | 1.432880271 | 1.11509E-06 | 2.47517E-07 |
| ENSMUSG00000060224 | 4.480666667 | 12.096 | 1.432744736 | 1.113E-28 | 6.40105E-30 |
| ENSMUSG00000045031 | 0.939666667 | 2.536 | 1.432333768 | 0.00411286 | 0.001521168 |
| ENSMUSG00000068740 | 1.434 | 3.857666667 | 1.427683464 | 4.13043E-11 | 6.03993E-12 |
| ENSMUSG00000050271 | 3.072666667 | 8.243666667 | 1.423794904 | 1.78382E-21 | 1.38799E-22 |
| ENSMUSG00000018293 | 560.1133333 | 1497.043667 | 1.418325627 | 1.4806E-103 | 2.2795E-105 |
| ENSMUSG00000026094 | 24.447 | 65.33233333 | 1.41813973 | 2.12328E-62 | 5.30673E-64 |
| ENSMUSG00000074652 | 0.378 | 1.006666667 | 1.413127909 | 1.47359E-05 | 3.7501E-06 |
| ENSMUSG00000039959 | 1.813 | 4.816666667 | 1.409656162 | 3.99577E-26 | 2.5215E-27 |
| ENSMUSG00000016477 | 5.522666667 | 14.672 | 1.409628586 | 7.40176E-41 | 2.95375E-42 |
| ENSMUSG00000028088 | 0.200666667 | 0.531333333 | 1.404816237 | 0.008143853 | 0.003188607 |
| ENSMUSG00000030672 | 1.494666667 | 3.952 | 1.40275917 | 0.011768206 | 0.00474417 |
| ENSMUSG00000030748 | 2.841333333 | 7.493666667 | 1.399103713 | 1.0477E-26 | 6.51013E-28 |
| ENSMUSG00000037960 | 19.723 | 51.98766667 | 1.398290393 | 4.5017E-19 | 3.96587E-20 |
| ENSMUSG00000028064 | 3.173666667 | 8.362 | 1.397697435 | 2.91729E-21 | 2.30418E-22 |
| ENSMUSG00000059762 | 0.105 | 0.275666667 | 1.392535501 | 0.030503048 | 0.013514089 |
| ENSMUSG00000029162 | 16.31633333 | 42.75133333 | 1.38965253 | 5.62682E-27 | 3.46917E-28 |
| ENSMUSG00000030701 | 15.25 | 39.931 | 1.388699959 | 8.28328E-40 | 3.37988E-41 |
| ENSMUSG00000021930 | 3.678 | 9.607 | 1.385164508 | 6.38085E-17 | 6.29538E-18 |
| ENSMUSG00000060470 | 7.408333333 | 19.327 | 1.383396797 | 5.19221E-12 | 7.06921E-13 |
| ENSMUSG00000054021 | 29.861 | 77.83333333 | 1.382125664 | 6.93868E-41 | 2.76417E-42 |
| ENSMUSG00000032902 | 14.43066667 | 37.61133333 | 1.3820295 | 1.73842E-54 | 5.00499E-56 |
| ENSMUSG00000044469 | 1.591 | 4.145 | 1.381438266 | 3.35494E-08 | 6.3189E-09 |
| ENSMUSG00000000561 | 18.263 | 47.46933333 | 1.378072017 | 8.87489E-43 | 3.38231E-44 |
| ENSMUSG00000020205 | 1.598333333 | 4.152333333 | 1.377353948 | 2.40985E-06 | 5.56042E-07 |
| ENSMUSG00000074793 | 0.448 | 1.162666667 | 1.375866902 | 0.001871443 | 0.000648493 |

| | | | | | |
|---|---|---|---|---|---|
| ENSMUSG00000028613 | 6.825666667 | 17.708 | 1.375359412 | 3.47301E-34 | 1.70006E-35 |
| ENSMUSG00000039232 | 5.586333333 | 14.43666667 | 1.369764106 | 1.39533E-34 | 6.74353E-36 |
| ENSMUSG00000032556 | 3.492 | 9.024 | 1.369713509 | 1.54005E-13 | 1.8703E-14 |
| ENSMUSG00000036381 | 15.27933333 | 39.47166667 | 1.36923584 | 1.26843E-24 | 8.6261E-26 |
| ENSMUSG00000042688 | 13.75466667 | 35.52166667 | 1.368778097 | 4.33224E-50 | 1.36691E-51 |
| ENSMUSG00000023088 | 13.30833333 | 34.34633333 | 1.367826184 | 9.69228E-79 | 1.94729E-80 |
| ENSMUSG00000020901 | 1.586666667 | 4.094333333 | 1.367629489 | 1.02423E-16 | 1.0197E-17 |
| ENSMUSG00000036185 | 0.640666667 | 1.652666667 | 1.367149935 | 0.039974795 | 0.018223803 |
| ENSMUSG00000000776 | 4.696 | 12.09366667 | 1.364747407 | 9.73107E-14 | 1.16431E-14 |
| ENSMUSG00000075054 | 4.207 | 10.83 | 1.36416952 | 2.81293E-17 | 2.73368E-18 |
| ENSMUSG00000028480 | 10.69133333 | 27.429 | 1.359260241 | 5.41053E-16 | 5.64064E-17 |
| ENSMUSG00000018377 | 33.41066667 | 85.714 | 1.359222094 | 2.9862E-105 | 4.5358E-107 |
| ENSMUSG00000029005 | 1.132666667 | 2.893333333 | 1.35300919 | 1.90456E-11 | 2.7193E-12 |
| ENSMUSG00000022089 | 13.96166667 | 35.634 | 1.351783266 | 5.13581E-28 | 3.02243E-29 |
| ENSMUSG00000048897 | 5.544333333 | 14.15033333 | 1.351750136 | 2.48088E-36 | 1.13331E-37 |
| ENSMUSG00000026970 | 21.827 | 55.694 | 1.351408059 | 1.95714E-47 | 6.59406E-49 |
| ENSMUSG00000037922 | 9.809666667 | 25.01 | 1.350229038 | 1.03708E-34 | 4.99064E-36 |
| ENSMUSG00000098923 | 14.051 | 35.747 | 1.347149362 | 2.21373E-57 | 5.94547E-59 |
| ENSMUSG00000030654 | 0.321666667 | 0.817 | 1.344769637 | 0.019922319 | 0.008464647 |
| ENSMUSG00000024456 | 35.05433333 | 89.01966667 | 1.344531301 | 6.59246E-68 | 1.49746E-69 |
| ENSMUSG00000045038 | 4.413666667 | 11.14566667 | 1.33643333 | 3.88269E-26 | 2.44478E-27 |
| ENSMUSG00000032462 | 16.82566667 | 42.45166667 | 1.335157527 | 1.41547E-79 | 2.80475E-81 |
| ENSMUSG00000057706 | 3.501666667 | 8.828 | 1.334044873 | 1.56757E-18 | 1.41346E-19 |
| ENSMUSG00000042590 | 14.07333333 | 35.475 | 1.333838606 | 9.80921E-18 | 9.22407E-19 |
| ENSMUSG00000048497 | 13.51766667 | 34.016 | 1.331367358 | 1.64261E-14 | 1.8735E-15 |
| ENSMUSG00000042155 | 3.959666667 | 9.958333333 | 1.330525321 | 8.00422E-28 | 4.7581E-29 |
| ENSMUSG00000037722 | 9.867 | 24.80533333 | 1.32996693 | 3.76861E-27 | 2.30269E-28 |
| ENSMUSG00000021591 | 27.15833333 | 68.248 | 1.329391822 | 2.28684E-30 | 1.25047E-31 |
| ENSMUSG00000029570 | 2.343333333 | 5.887666667 | 1.329133806 | 9.31509E-11 | 1.40395E-11 |
| ENSMUSG00000022309 | 64.978 | 162.883 | 1.325812795 | 9.57655E-39 | 4.03321E-40 |
| ENSMUSG00000046711 | 69.69233333 | 174.29 | 1.322417933 | 7.66942E-32 | 4.01898E-33 |
| ENSMUSG00000041607 | 9.791666667 | 24.48233333 | 1.322114712 | 3.83678E-39 | 1.59733E-40 |

| | | | | | |
|---|---|---|---|---|---|
| ENSMUSG00000004451 | 23.49633333 | 58.66233333 | 1.319998819 | 2.89037E-49 | 9.29932E-51 |
| ENSMUSG00000000552 | 11.69066667 | 29.18566667 | 1.31990282 | 1.35137E-21 | 1.04683E-22 |
| ENSMUSG00000030341 | 6.151666667 | 15.35166667 | 1.319346055 | 1.44266E-21 | 1.12054E-22 |
| ENSMUSG00000021733 | 5.787333333 | 14.43833333 | 1.31893357 | 1.84279E-46 | 6.33601E-48 |
| ENSMUSG00000034006 | 8.181333333 | 20.38266667 | 1.316934925 | 5.14059E-14 | 6.0584E-15 |
| ENSMUSG00000023805 | 3.059 | 7.598666667 | 1.312686185 | 5.24719E-27 | 3.22787E-28 |
| ENSMUSG00000041911 | 3.783666667 | 9.395666667 | 1.312210535 | 4.18815E-16 | 4.33736E-17 |
| ENSMUSG00000032101 | 1.447 | 3.589333333 | 1.310650987 | 1.30076E-10 | 1.99235E-11 |
| ENSMUSG00000027669 | 0.727666667 | 1.801333333 | 1.307715544 | 5.59454E-07 | 1.1916E-07 |
| ENSMUSG00000030761 | 5.507 | 13.631 | 1.307552892 | 1.20669E-19 | 1.0364E-20 |
| ENSMUSG00000017776 | 16.80033333 | 41.55233333 | 1.306439635 | 1.23781E-44 | 4.46106E-46 |
| ENSMUSG00000044080 | 33.03533333 | 81.70433333 | 1.306402694 | 5.00579E-28 | 2.93767E-29 |
| ENSMUSG00000074825 | 10.82433333 | 26.741 | 1.304775244 | 4.5969E-44 | 1.67893E-45 |
| ENSMUSG00000027763 | 143.0216667 | 353.0566667 | 1.303666038 | 1.40084E-85 | 2.57266E-87 |
| ENSMUSG00000004530 | 23.51733333 | 57.99233333 | 1.302137707 | 1.30149E-57 | 3.47746E-59 |
| ENSMUSG00000036743 | 6.799 | 16.70566667 | 1.296943082 | 1.38914E-14 | 1.56907E-15 |
| ENSMUSG00000029591 | 3.162666667 | 7.743 | 1.291751133 | 1.44322E-08 | 2.63156E-09 |
| ENSMUSG00000002825 | 6.978 | 17.07333333 | 1.29085925 | 3.0612E-14 | 3.55491E-15 |
| ENSMUSG00000052087 | 4.866333333 | 11.89366667 | 1.289286498 | 6.37942E-17 | 6.28957E-18 |
| ENSMUSG00000035678 | 5.771666667 | 14.105 | 1.289146779 | 2.31169E-11 | 3.31815E-12 |
| ENSMUSG00000028212 | 6.216 | 15.17466667 | 1.287606417 | 5.61564E-24 | 3.94305E-25 |
| ENSMUSG00000027712 | 25.64533333 | 62.575 | 1.286890063 | 6.60995E-31 | 3.54137E-32 |
| ENSMUSG00000038295 | 0.547666667 | 1.335 | 1.285469762 | 3.29933E-05 | 8.77225E-06 |
| ENSMUSG00000032508 | 14.13766667 | 34.427 | 1.283996437 | 1.85923E-31 | 9.8199E-33 |
| ENSMUSG00000020570 | 45.61366667 | 110.7993333 | 1.280411149 | 8.19089E-65 | 1.96799E-66 |
| ENSMUSG00000044350 | 2.721333333 | 6.602 | 1.278589457 | 8.55185E-12 | 1.18146E-12 |
| ENSMUSG00000034771 | 0.877666667 | 2.129 | 1.278430929 | 0.002041603 | 0.000712672 |
| ENSMUSG00000026447 | 1.666 | 4.041 | 1.278323951 | 4.05963E-24 | 2.83087E-25 |
| ENSMUSG00000027489 | 0.554333333 | 1.342 | 1.275559004 | 0.013611766 | 0.005560675 |
| ENSMUSG00000062175 | 0.588333333 | 1.424 | 1.275243464 | 0.002997724 | 0.001080373 |
| ENSMUSG00000078249 | 167.697 | 405.5803333 | 1.274130817 | 1.48315E-54 | 4.2598E-56 |
| ENSMUSG00000005057 | 1.504 | 3.636666667 | 1.273812129 | 3.61311E-05 | 9.66139E-06 |

| | | | | | |
|---|---|---|---|---|---|
| ENSMUSG00000028568 | 15.03233333 | 36.30433333 | 1.272072797 | 3.49077E-18 | 3.20782E-19 |
| ENSMUSG00000004667 | 34.665 | 83.43166667 | 1.267115305 | 7.59547E-37 | 3.41912E-38 |
| ENSMUSG00000030663 | 56.596 | 135.742 | 1.262095178 | 7.35071E-47 | 2.51723E-48 |
| ENSMUSG00000053192 | 3.281333333 | 7.857333333 | 1.259757608 | 7.91283E-10 | 1.30023E-10 |
| ENSMUSG00000006301 | 2.156666667 | 5.146666667 | 1.254835135 | 2.00573E-10 | 3.13101E-11 |
| ENSMUSG00000027201 | 22.49 | 53.65433333 | 1.254411032 | 2.75545E-42 | 1.06155E-43 |
| ENSMUSG00000024238 | 9.364666667 | 22.339 | 1.254265057 | 8.41046E-37 | 3.80341E-38 |
| ENSMUSG00000032047 | 27.43066667 | 65.396 | 1.253412709 | 6.82583E-44 | 2.51657E-45 |
| ENSMUSG00000030691 | 2.473666667 | 5.893 | 1.25235116 | 1.05301E-15 | 1.11306E-16 |
| ENSMUSG00000046546 | 0.640666667 | 1.525333333 | 1.251478716 | 0.000124648 | 3.59298E-05 |
| ENSMUSG00000022051 | 25.343 | 60.29433333 | 1.250435104 | 2.99582E-32 | 1.53886E-33 |
| ENSMUSG00000028015 | 0.984333333 | 2.341333333 | 1.250111488 | 7.55614E-06 | 1.85043E-06 |
| ENSMUSG00000003541 | 12.88833333 | 30.64733333 | 1.249695836 | 1.01699E-16 | 1.01109E-17 |
| ENSMUSG00000026854 | 9.345333333 | 22.218 | 1.249410927 | 1.72511E-38 | 7.32492E-40 |
| ENSMUSG00000010406 | 13.315 | 31.571 | 1.24554753 | 1.47781E-08 | 2.69715E-09 |
| ENSMUSG00000029762 | 10.16533333 | 24.05766667 | 1.2428392 | 6.18295E-10 | 1.00787E-10 |
| ENSMUSG00000050621 | 2.821333333 | 6.661333333 | 1.239433849 | 0.007174105 | 0.002777712 |
| ENSMUSG00000032340 | 7.819666667 | 18.457 | 1.238989061 | 1.56222E-29 | 8.7689E-31 |
| ENSMUSG00000002107 | 25.456 | 60.05833333 | 1.238358699 | 2.15877E-63 | 5.306E-65 |
| ENSMUSG00000079442 | 3.264 | 7.685666667 | 1.23552935 | 1.48265E-12 | 1.92855E-13 |
| ENSMUSG00000051444 | 3.762666667 | 8.848666667 | 1.233704596 | 1.13799E-14 | 1.28067E-15 |
| ENSMUSG00000092165 | 3.062666667 | 7.199666667 | 1.233141758 | 4.7571E-05 | 1.29733E-05 |
| ENSMUSG00000030004 | 0.778333333 | 1.826666667 | 1.230753343 | 0.019286888 | 0.008165368 |
| ENSMUSG00000021929 | 18.173 | 42.619 | 1.229700143 | 2.545E-55 | 7.11631E-57 |
| ENSMUSG00000030707 | 96.33533333 | 225.6926667 | 1.228222599 | 4.8176E-76 | 9.89481E-78 |
| ENSMUSG00000027387 | 1.795 | 4.196 | 1.225030834 | 8.37007E-06 | 2.06247E-06 |
| ENSMUSG00000002058 | 17.62233333 | 41.17066667 | 1.224211849 | 1.51102E-23 | 1.08392E-24 |
| ENSMUSG00000031467 | 8.488666667 | 19.807 | 1.222400514 | 4.21688E-25 | 2.78913E-26 |
| ENSMUSG00000078931 | 6.426 | 14.986 | 1.221622471 | 1.18524E-15 | 1.25774E-16 |
| ENSMUSG00000001435 | 0.256 | 0.597 | 1.221587121 | 0.003306622 | 0.001201287 |
| ENSMUSG00000031838 | 2.008333333 | 4.675666667 | 1.219173342 | 0.000412399 | 0.000128013 |
| ENSMUSG00000022895 | 11.98233333 | 27.88633333 | 1.218649379 | 4.15471E-48 | 1.36826E-49 |

| | | | | | |
|---|---|---|---|---|---|
| ENSMUSG00000047180 | 5.868 | 13.65266667 | 1.218241993 | 1.5615E-17 | 1.48992E-18 |
| ENSMUSG00000037818 | 3.776333333 | 8.778333333 | 1.216960938 | 1.17961E-09 | 1.95949E-10 |
| ENSMUSG00000021384 | 2.614 | 6.075 | 1.216625268 | 1.20642E-05 | 3.02937E-06 |
| ENSMUSG00000027210 | 2.275666667 | 5.278666667 | 1.213884315 | 3.50966E-09 | 6.03844E-10 |
| ENSMUSG00000022307 | 27.21533333 | 62.97633333 | 1.210390056 | 2.78654E-16 | 2.8531E-17 |
| ENSMUSG00000030309 | 2.921 | 6.739666667 | 1.206214882 | 1.27173E-13 | 1.53479E-14 |
| ENSMUSG00000021697 | 14.47966667 | 33.407 | 1.206122041 | 7.58743E-31 | 4.08602E-32 |
| ENSMUSG00000008450 | 10.91533333 | 25.17833333 | 1.2058266 | 9.90811E-07 | 2.18356E-07 |
| ENSMUSG00000052310 | 17.70033333 | 40.812 | 1.205216882 | 3.27708E-39 | 1.35979E-40 |
| ENSMUSG00000034930 | 14.02466667 | 32.312 | 1.204103569 | 5.04623E-28 | 2.96488E-29 |
| ENSMUSG00000035047 | 11.67533333 | 26.836 | 1.200705909 | 1.81723E-35 | 8.46884E-37 |
| ENSMUSG00000005610 | 257.5393333 | 591.9293333 | 1.200632164 | 4.75438E-87 | 8.60016E-89 |
| ENSMUSG00000062995 | 27.66533333 | 63.52166667 | 1.199169459 | 1.82552E-46 | 6.26404E-48 |
| ENSMUSG00000053477 | 9.644666667 | 22.14133333 | 1.19893882 | 1.9101E-24 | 1.30953E-25 |
| ENSMUSG00000004446 | 21.96066667 | 50.34266667 | 1.196859787 | 6.91601E-39 | 2.89445E-40 |
| ENSMUSG00000026974 | 15.16466667 | 34.75833333 | 1.19664512 | 1.41438E-41 | 5.54658E-43 |
| ENSMUSG00000046490 | 0.344333333 | 0.789 | 1.196219452 | 0.004439487 | 0.001654233 |
| ENSMUSG00000047766 | 3.87 | 8.848333333 | 1.193072169 | 5.18004E-15 | 5.7401E-16 |
| ENSMUSG00000026979 | 32.50633333 | 74.14833333 | 1.189693434 | 1.27936E-80 | 2.49971E-82 |
| ENSMUSG00000033386 | 23.45266667 | 53.453 | 1.188518948 | 5.91071E-58 | 1.56297E-59 |
| ENSMUSG00000112743 | 0.814666667 | 1.855 | 1.187137402 | 0.042081986 | 0.019303557 |
| ENSMUSG00000053799 | 19.53333333 | 44.44633333 | 1.186126243 | 2.53236E-32 | 1.29555E-33 |
| ENSMUSG00000029171 | 20.79466667 | 47.311 | 1.185962096 | 1.5449E-38 | 6.53841E-40 |
| ENSMUSG00000027637 | 52.24466667 | 118.8243333 | 1.185474633 | 6.91601E-39 | 2.89838E-40 |
| ENSMUSG00000038770 | 0.462333333 | 1.050333333 | 1.183841967 | 0.008862165 | 0.003494938 |
| ENSMUSG00000036986 | 11.02433333 | 25.035 | 1.18325504 | 1.04034E-49 | 3.29686E-51 |
| ENSMUSG00000046668 | 11.883 | 26.954 | 1.181600279 | 7.57303E-25 | 5.0717E-26 |
| ENSMUSG00000043733 | 17.89333333 | 40.504 | 1.178642219 | 4.43306E-55 | 1.25793E-56 |
| ENSMUSG00000007613 | 13.90666667 | 31.47766667 | 1.178551946 | 1.86596E-48 | 6.08072E-50 |
| ENSMUSG00000044252 | 6.907 | 15.60566667 | 1.175938861 | 6.65256E-18 | 6.18683E-19 |
| ENSMUSG00000031482 | 2.175 | 4.911 | 1.175001422 | 6.99162E-11 | 1.04363E-11 |
| ENSMUSG00000043251 | 6.453 | 14.56033333 | 1.174001453 | 1.06651E-16 | 1.06327E-17 |

| | | | | | |
|---|---|---|---|---|---|
| ENSMUSG00000058099 | 6.073 | 13.656 | 1.169053689 | 4.83922E-25 | 3.21078E-26 |
| ENSMUSG00000022323 | 12.39833333 | 27.85433333 | 1.16775559 | 2.35931E-14 | 2.71539E-15 |
| ENSMUSG00000025608 | 4.259333333 | 9.561 | 1.166533881 | 8.65235E-26 | 5.53168E-27 |
| ENSMUSG00000014686 | 1.006666667 | 2.258 | 1.165459437 | 0.003198209 | 0.00115881 |
| ENSMUSG00000003948 | 5.142666667 | 11.52833333 | 1.164595405 | 3.71881E-17 | 3.64075E-18 |
| ENSMUSG00000042510 | 0.365333333 | 0.818333333 | 1.163475226 | 0.015839211 | 0.006574519 |
| ENSMUSG00000007036 | 23.06466667 | 51.63533333 | 1.162674177 | 3.97998E-45 | 1.41789E-46 |
| ENSMUSG00000025421 | 12.239 | 27.372 | 1.161215167 | 5.82034E-30 | 3.21879E-31 |
| ENSMUSG00000091477 | 0.665333333 | 1.484 | 1.157341872 | 0.030114994 | 0.013323263 |
| ENSMUSG00000002983 | 1.641666667 | 3.661 | 1.15707655 | 0.00086923 | 0.000283802 |
| ENSMUSG00000027286 | 5.409 | 12.04066667 | 1.154481471 | 8.86141E-11 | 1.33374E-11 |
| ENSMUSG00000022299 | 5.795 | 12.89666667 | 1.154117662 | 6.83343E-19 | 6.09083E-20 |
| ENSMUSG00000033967 | 1.000666667 | 2.225333333 | 1.153059978 | 7.50697E-06 | 1.83683E-06 |
| ENSMUSG00000002881 | 14.055 | 31.23866667 | 1.152249424 | 4.98621E-42 | 1.93472E-43 |
| ENSMUSG00000033623 | 2.789 | 6.192333333 | 1.150735199 | 1.85042E-33 | 9.18569E-35 |
| ENSMUSG00000036249 | 12.21566667 | 27.07366667 | 1.148157689 | 6.25963E-29 | 3.57841E-30 |
| ENSMUSG00000023087 | 422.473 | 936.059 | 1.147740324 | 8.92667E-49 | 2.89667E-50 |
| ENSMUSG00000114133 | 5.476666667 | 12.12833333 | 1.14701133 | 1.43734E-17 | 1.36946E-18 |
| ENSMUSG00000004771 | 45.85866667 | 101.4943333 | 1.146132866 | 2.04159E-44 | 7.41422E-46 |
| ENSMUSG00000038059 | 18.53166667 | 41.00533333 | 1.145818928 | 6.14422E-24 | 4.31843E-25 |
| ENSMUSG00000025650 | 0.108333333 | 0.239333333 | 1.143544126 | 0.041200808 | 0.018850991 |
| ENSMUSG00000050908 | 0.256666667 | 0.566666667 | 1.142604395 | 0.049403069 | 0.023115479 |
| ENSMUSG00000007039 | 0.813333333 | 1.792666667 | 1.140188607 | 0.02068538 | 0.008834559 |
| ENSMUSG00000040111 | 4.485666667 | 9.865333333 | 1.137045381 | 5.63743E-33 | 2.84518E-34 |
| ENSMUSG00000022092 | 5.193 | 11.398 | 1.134140567 | 3.20511E-15 | 3.5096E-16 |
| ENSMUSG00000027327 | 26.605 | 58.37033333 | 1.133537903 | 2.78928E-29 | 1.57335E-30 |
| ENSMUSG00000014226 | 27.519 | 60.36966667 | 1.13339579 | 2.51959E-32 | 1.28728E-33 |
| ENSMUSG00000029050 | 10.96233333 | 24.04733333 | 1.13332201 | 4.10443E-19 | 3.60172E-20 |
| ENSMUSG00000030102 | 18.80033333 | 41.238 | 1.133216124 | 1.32352E-54 | 3.79219E-56 |
| ENSMUSG00000066278 | 10.699 | 23.46366667 | 1.132952522 | 4.82714E-27 | 2.96281E-28 |
| ENSMUSG00000007041 | 159.422 | 349.5736667 | 1.132745778 | 5.12463E-65 | 1.22419E-66 |
| ENSMUSG00000005107 | 3.352666667 | 7.347 | 1.131846222 | 6.60024E-17 | 6.51639E-18 |

| | | | | | |
|---|---|---|---|---|---|
| ENSMUSG00000001741 | 5.174333333 | 11.32266667 | 1.129768874 | 1.30719E-10 | 2.00537E-11 |
| ENSMUSG00000068744 | 9.395 | 20.52733333 | 1.127581155 | 9.37993E-17 | 9.29962E-18 |
| ENSMUSG00000021996 | 67.242 | 146.8786667 | 1.127190327 | 9.28155E-35 | 4.42161E-36 |
| ENSMUSG00000058186 | 1.26 | 2.750666667 | 1.126357587 | 1.94395E-08 | 3.58753E-09 |
| ENSMUSG00000023259 | 2.133 | 4.653666667 | 1.125483911 | 6.61919E-08 | 1.28371E-08 |
| ENSMUSG00000042354 | 28.86866667 | 62.819 | 1.121696501 | 2.43761E-43 | 9.12169E-45 |
| ENSMUSG00000025314 | 11.347 | 24.64933333 | 1.11923771 | 6.79398E-52 | 2.08735E-53 |
| ENSMUSG00000038028 | 2.39 | 5.189333333 | 1.118538591 | 5.24131E-07 | 1.11383E-07 |
| ENSMUSG00000071470 | 3.130333333 | 6.783333333 | 1.115678098 | 0.000468549 | 0.000146575 |
| ENSMUSG00000032109 | 3.164 | 6.851666667 | 1.114705366 | 1.43857E-12 | 1.86922E-13 |
| ENSMUSG00000074480 | 1.160333333 | 2.509333333 | 1.112764814 | 4.42297E-09 | 7.69093E-10 |
| ENSMUSG00000116564 | 11.01333333 | 23.81666667 | 1.112720325 | 1.05607E-17 | 9.9453E-19 |
| ENSMUSG00000020644 | 1.871666667 | 4.044333333 | 1.111578387 | 0.000994716 | 0.000327451 |
| ENSMUSG00000057176 | 0.321666667 | 0.695 | 1.111446536 | 0.026745348 | 0.011703398 |
| ENSMUSG00000054814 | 4.108666667 | 8.857333333 | 1.108202123 | 1.07522E-13 | 1.28872E-14 |
| ENSMUSG00000034101 | 16.18866667 | 34.88733333 | 1.10771916 | 5.35826E-53 | 1.60186E-54 |
| ENSMUSG00000003526 | 12.28133333 | 26.447 | 1.106636884 | 5.52094E-29 | 3.14088E-30 |
| ENSMUSG00000066007 | 0.577666667 | 1.243666667 | 1.106290706 | 0.001032101 | 0.000341112 |
| ENSMUSG00000026812 | 5.418333333 | 11.647 | 1.104037342 | 7.12404E-37 | 3.19706E-38 |
| ENSMUSG00000040236 | 9.393666667 | 20.18333333 | 1.103404153 | 1.69823E-24 | 1.15842E-25 |
| ENSMUSG00000027981 | 9.584666667 | 20.59266667 | 1.103330501 | 2.184E-22 | 1.63905E-23 |
| ENSMUSG00000026843 | 18.51333333 | 39.73633333 | 1.101894078 | 7.44678E-32 | 3.89203E-33 |
| ENSMUSG00000038612 | 45.81733333 | 98.32266667 | 1.101630551 | 2.5868E-67 | 5.983E-69 |
| ENSMUSG00000091512 | 25.182 | 53.97466667 | 1.09988956 | 2.83336E-22 | 2.13617E-23 |
| ENSMUSG00000001750 | 9.233 | 19.785 | 1.099535674 | 2.29723E-20 | 1.88898E-21 |
| ENSMUSG00000027130 | 12.98666667 | 27.82033333 | 1.099108529 | 2.70915E-35 | 1.26816E-36 |
| ENSMUSG00000030276 | 2.124333333 | 4.549 | 1.098539275 | 6.94872E-05 | 1.93724E-05 |
| ENSMUSG00000038843 | 19.47366667 | 41.63633333 | 1.096318472 | 1.1353E-22 | 8.37914E-24 |
| ENSMUSG00000042677 | 1.269666667 | 2.709666667 | 1.0936656 | 5.98926E-05 | 1.6561E-05 |
| ENSMUSG00000021958 | 4.262 | 9.074333333 | 1.090261063 | 2.83364E-07 | 5.8281E-08 |
| ENSMUSG00000096740 | 4.095 | 8.710666667 | 1.088919687 | 6.15928E-14 | 7.30575E-15 |
| ENSMUSG00000024691 | 94.274 | 200.477 | 1.088504884 | 6.49911E-35 | 3.07815E-36 |

| | | | | | |
|---|---|---|---|---|---|
| ENSMUSG00000026872 | 13.573 | 28.861 | 1.088381658 | 8.59443E-42 | 3.3585E-43 |
| ENSMUSG00000016496 | 12.60766667 | 26.77366667 | 1.086513433 | 5.55493E-14 | 6.5659E-15 |
| ENSMUSG00000071068 | 26.86766667 | 57.014 | 1.085445187 | 7.98491E-45 | 2.8612E-46 |
| ENSMUSG00000061665 | 14.07133333 | 29.85933333 | 1.085422917 | 2.02539E-52 | 6.12485E-54 |
| ENSMUSG00000032412 | 44.03866667 | 93.26933333 | 1.082632015 | 1.93909E-56 | 5.28817E-58 |
| ENSMUSG00000035049 | 13.13 | 27.713 | 1.077695978 | 2.74242E-23 | 1.98051E-24 |
| ENSMUSG00000037572 | 12.81033333 | 27.02133333 | 1.076790848 | 1.26277E-34 | 6.08545E-36 |
| ENSMUSG00000061186 | 8.143666667 | 17.15033333 | 1.074486201 | 2.24452E-45 | 7.90325E-47 |
| ENSMUSG00000025574 | 32.585 | 68.52766667 | 1.07247857 | 1.39345E-31 | 7.35014E-33 |
| ENSMUSG00000027405 | 116.0983333 | 243.969 | 1.071350581 | 3.85539E-81 | 7.45312E-83 |
| ENSMUSG00000027075 | 15.39866667 | 32.34933333 | 1.070930544 | 3.9216E-29 | 2.22289E-30 |
| ENSMUSG00000025260 | 44.87466667 | 94.223 | 1.070178045 | 4.14409E-37 | 1.83113E-38 |
| ENSMUSG00000020142 | 6.032666667 | 12.66233333 | 1.069675506 | 4.10519E-15 | 4.52353E-16 |
| ENSMUSG00000033502 | 6.130333333 | 12.86266667 | 1.069152344 | 4.27818E-12 | 5.77453E-13 |
| ENSMUSG00000037725 | 15.92633333 | 33.412 | 1.068952185 | 6.49173E-24 | 4.57164E-25 |
| ENSMUSG00000009621 | 1.190333333 | 2.493 | 1.06651725 | 8.48316E-06 | 2.09268E-06 |
| ENSMUSG00000030966 | 1.513333333 | 3.163333333 | 1.06371579 | 2.09432E-06 | 4.79333E-07 |
| ENSMUSG00000022216 | 39.86266667 | 83.24566667 | 1.062336951 | 7.42662E-39 | 3.1175E-40 |
| ENSMUSG00000014873 | 9.244666667 | 19.30533333 | 1.062306258 | 6.06476E-14 | 7.18945E-15 |
| ENSMUSG00000030725 | 0.725666667 | 1.515 | 1.061938887 | 0.013839087 | 0.005668828 |
| ENSMUSG00000001228 | 73.20133333 | 152.8076667 | 1.061775096 | 3.36442E-53 | 9.98826E-55 |
| ENSMUSG00000040296 | 7.713 | 16.097 | 1.061427822 | 2.64097E-15 | 2.8718E-16 |
| ENSMUSG00000025809 | 78.72933333 | 164.2873333 | 1.061248085 | 2.79863E-33 | 1.39893E-34 |
| ENSMUSG00000038866 | 5.512 | 11.486 | 1.059228674 | 1.8729E-13 | 2.28616E-14 |
| ENSMUSG00000030403 | 43.354 | 90.296 | 1.058496972 | 1.95623E-21 | 1.52754E-22 |
| ENSMUSG00000021868 | 30.00466667 | 62.46 | 1.057745667 | 3.06145E-19 | 2.67592E-20 |
| ENSMUSG00000054115 | 10.435 | 21.70866667 | 1.056840519 | 1.52072E-23 | 1.09193E-24 |
| ENSMUSG00000040272 | 2.869 | 5.966333333 | 1.056296614 | 1.88973E-08 | 3.48226E-09 |
| ENSMUSG00000061689 | 9.981666667 | 20.71133333 | 1.053067801 | 2.69282E-12 | 3.57704E-13 |
| ENSMUSG00000079197 | 20.83966667 | 43.21133333 | 1.052077546 | 8.75641E-23 | 6.44458E-24 |
| ENSMUSG00000019916 | 16.79233333 | 34.803 | 1.051408961 | 1.07374E-24 | 7.27242E-26 |
| ENSMUSG00000017561 | 30.343 | 62.85966667 | 1.050770891 | 1.54941E-26 | 9.65973E-28 |

| | | | | | |
|---|---|---|---|---|---|
| ENSMUSG00000008318 | 3.009333333 | 6.225 | 1.048629919 | 2.50474E-05 | 6.55894E-06 |
| ENSMUSG00000028069 | 14.16566667 | 29.29966667 | 1.048485752 | 9.89235E-21 | 8.0319E-22 |
| ENSMUSG00000044712 | 1204.300667 | 2490.522 | 1.048252534 | 1.78861E-37 | 7.76744E-39 |
| ENSMUSG00000027950 | 0.814 | 1.682666667 | 1.04764871 | 0.00158577 | 0.000542714 |
| ENSMUSG00000028410 | 72.537 | 149.9026667 | 1.047237064 | 5.81415E-44 | 2.12752E-45 |
| ENSMUSG00000064090 | 10.95433333 | 22.62633333 | 1.046501124 | 1.56565E-15 | 1.67872E-16 |
| ENSMUSG00000034235 | 1.278 | 2.639333333 | 1.04628573 | 1.15691E-05 | 2.89786E-06 |
| ENSMUSG00000031444 | 3.354 | 6.922 | 1.045306253 | 1.21733E-05 | 3.06097E-06 |
| ENSMUSG00000057541 | 12.507 | 25.80566667 | 1.044952124 | 6.1918E-27 | 3.83033E-28 |
| ENSMUSG00000023277 | 21.54766667 | 44.44066667 | 1.044348807 | 2.74084E-23 | 1.97748E-24 |
| ENSMUSG00000037936 | 15.78533333 | 32.54633333 | 1.043910293 | 2.02167E-18 | 1.83128E-19 |
| ENSMUSG00000033209 | 1.576 | 3.245666667 | 1.042247306 | 6.26582E-10 | 1.02181E-10 |
| ENSMUSG00000032041 | 6.210333333 | 12.74266667 | 1.036924612 | 1.97487E-12 | 2.59062E-13 |
| ENSMUSG00000063354 | 7.875666667 | 16.15866667 | 1.036834203 | 9.49878E-17 | 9.42738E-18 |
| ENSMUSG00000026879 | 10.74766667 | 22.02633333 | 1.035205871 | 1.35122E-19 | 1.1624E-20 |
| ENSMUSG00000024511 | 15.62366667 | 32.01733333 | 1.035120079 | 2.07331E-27 | 1.25252E-28 |
| ENSMUSG00000063275 | 6.433 | 13.12666667 | 1.028937017 | 9.53162E-07 | 2.09598E-07 |
| ENSMUSG00000024570 | 10.466 | 21.33766667 | 1.027692258 | 2.88968E-14 | 3.35174E-15 |
| ENSMUSG00000000631 | 12.53266667 | 25.54133333 | 1.02714042 | 5.54561E-31 | 2.95965E-32 |
| ENSMUSG00000038543 | 3.957666667 | 8.064 | 1.026845532 | 0.002428932 | 0.000860456 |
| ENSMUSG00000027963 | 4.780666667 | 9.727333333 | 1.02483254 | 2.59359E-15 | 2.81849E-16 |
| ENSMUSG00000001999 | 11.53 | 23.44566667 | 1.023928789 | 1.4686E-10 | 2.26312E-11 |
| ENSMUSG00000028581 | 100.635 | 204.6153333 | 1.023782111 | 5.0759E-27 | 3.119E-28 |
| ENSMUSG00000021840 | 20.40766667 | 41.46566667 | 1.022806048 | 8.97589E-24 | 6.39541E-25 |
| ENSMUSG00000030946 | 9.052 | 18.39066667 | 1.022665289 | 6.74307E-11 | 1.00466E-11 |
| ENSMUSG00000022797 | 55.525 | 112.638 | 1.02048423 | 1.32524E-59 | 3.42199E-61 |
| ENSMUSG00000037692 | 3.884 | 7.879 | 1.020469334 | 1.25363E-10 | 1.91628E-11 |
| ENSMUSG00000027953 | 13.75866667 | 27.88866667 | 1.019338295 | 6.8924E-11 | 1.02786E-11 |
| ENSMUSG00000115483 | 16.269 | 32.96933333 | 1.018999139 | 1.95469E-18 | 1.76926E-19 |
| ENSMUSG00000038235 | 6.818666667 | 13.81166667 | 1.018325856 | 3.80691E-14 | 4.44455E-15 |
| ENSMUSG00000028059 | 34.197 | 69.244 | 1.0178193 | 7.27536E-31 | 3.91294E-32 |
| ENSMUSG00000030879 | 23.255 | 47.07033333 | 1.01727713 | 2.93959E-11 | 4.2519E-12 |

| | | | | | |
|---|---|---|---|---|---|
| ENSMUSG00000038180 | 0.803333333 | 1.625333333 | 1.01666498 | 0.030628805 | 0.013584607 |
| ENSMUSG00000053907 | 65.18366667 | 131.7573333 | 1.015300848 | 4.6406E-43 | 1.74615E-44 |
| ENSMUSG00000026815 | 30.53833333 | 61.702 | 1.014695926 | 4.11156E-18 | 3.79817E-19 |
| ENSMUSG00000028645 | 17.25466667 | 34.862 | 1.014668734 | 9.36159E-28 | 5.57145E-29 |
| ENSMUSG00000005968 | 2.520666667 | 5.092666667 | 1.014615942 | 3.82974E-09 | 6.62351E-10 |
| ENSMUSG00000000628 | 23.86333333 | 48.20733333 | 1.014457048 | 4.60027E-47 | 1.56582E-48 |
| ENSMUSG00000020014 | 1.393 | 2.810333333 | 1.012546001 | 4.23627E-15 | 4.67089E-16 |
| ENSMUSG00000042055 | 11.351 | 22.891 | 1.011961087 | 2.05137E-17 | 1.97716E-18 |
| ENSMUSG00000040699 | 43.36533333 | 87.438 | 1.011718203 | 3.38978E-27 | 2.0642E-28 |
| ENSMUSG00000028175 | 16.96366667 | 34.20033333 | 1.011562347 | 7.27098E-20 | 6.17963E-21 |
| ENSMUSG00000032946 | 53.29533333 | 107.4256667 | 1.011257612 | 4.38128E-37 | 1.93897E-38 |
| ENSMUSG00000024383 | 2.949 | 5.941333333 | 1.01056091 | 5.35593E-28 | 3.16164E-29 |
| ENSMUSG00000074671 | 8.198 | 16.50733333 | 1.009763185 | 6.49341E-19 | 5.7788E-20 |
| ENSMUSG00000064179 | 3.986333333 | 8.020666667 | 1.008659804 | 8.69101E-05 | 2.44697E-05 |
| ENSMUSG00000052798 | 23.169 | 46.612 | 1.008505639 | 2.86972E-18 | 2.62325E-19 |
| ENSMUSG00000031142 | 1.843333333 | 3.706 | 1.007545902 | 3.38752E-10 | 5.40731E-11 |
| ENSMUSG00000074182 | 13.17666667 | 26.45266667 | 1.005427712 | 9.49878E-17 | 9.43057E-18 |
| ENSMUSG00000032456 | 2.582333333 | 5.184 | 1.005390479 | 8.60365E-05 | 2.42E-05 |
| ENSMUSG00000018008 | 15.79933333 | 31.70766667 | 1.004968031 | 1.89741E-20 | 1.55759E-21 |
| ENSMUSG00000026857 | 4.725666667 | 9.471 | 1.002998892 | 6.2373E-08 | 1.20491E-08 |
| ENSMUSG00000045140 | 3.273 | 6.556 | 1.002202252 | 2.27568E-06 | 5.23043E-07 |
