# Supplementary material for: Mutations of RAS genes identified in acute myeloid leukemia affect glycerophospholipid metabolism pathway
Source: Front Oncol. 2023 Nov 14;13:1280192. doi: 10.3389/fonc.2023.1280192 (PMC10682766; doi:10.3389/fonc.2023.1280192)
Supplement: Supplementary file 4 [file DataSheet_4.pdf]

**The down-regulated genes of NRAS（Q61K）cell line**

| ENSEMBL | BaF3 | BaF3 NRAS（Q61K） | log2fc | FDR | Pvalue |
|---|---|---|---|---|---|
| ENSMUSG00000034430 | 8.162333333 | 4.081 | -1.000058918 | 8.70301E-11 | 1.30749E-11 |
| ENSMUSG00000058503 | 38.12766667 | 19.06133333 | -1.000189205 | 3.65601E-18 | 3.36472E-19 |
| ENSMUSG00000019737 | 5.623 | 2.81 | -1.000769916 | 0.002643764 | 0.000943862 |
| ENSMUSG00000024614 | 32.89966667 | 16.43433333 | -1.001360033 | 0.002316977 | 0.000817917 |
| ENSMUSG00000001761 | 2.226666667 | 1.111666667 | -1.002161341 | 0.00063269 | 0.000201863 |
| ENSMUSG00000020747 | 20.84166667 | 10.394 | -1.003719688 | 1.18489E-12 | 1.52816E-13 |
| ENSMUSG00000045466 | 6.909333333 | 3.441 | -1.005718623 | 1.04502E-06 | 2.30953E-07 |
| ENSMUSG00000063873 | 1.463666667 | 0.728 | -1.007576678 | 0.006304633 | 0.002411466 |
| ENSMUSG00000055991 | 4.844666667 | 2.407666667 | -1.008761736 | 7.11223E-07 | 1.53401E-07 |
| ENSMUSG00000021266 | 75.30033333 | 37.40666667 | -1.009360839 | 2.26417E-16 | 2.30732E-17 |
| ENSMUSG00000029125 | 26.71766667 | 13.26866667 | -1.009770613 | 3.96955E-11 | 5.79921E-12 |
| ENSMUSG00000002845 | 35.024 | 17.388 | -1.010251859 | 1.95423E-22 | 1.46392E-23 |
| ENSMUSG00000025981 | 14.83433333 | 7.362333333 | -1.010705117 | 2.43262E-10 | 3.82092E-11 |
| ENSMUSG00000023882 | 3.677333333 | 1.825 | -1.010763492 | 0.001351001 | 0.000455744 |
| ENSMUSG00000032763 | 10.668 | 5.292666667 | -1.011223029 | 6.07662E-11 | 9.03689E-12 |
| ENSMUSG00000036223 | 8.483666667 | 4.205333333 | -1.012467777 | 3.88215E-06 | 9.20416E-07 |
| ENSMUSG00000043190 | 4.935666667 | 2.445666667 | -1.01301718 | 0.021554106 | 0.00923386 |
| ENSMUSG00000038147 | 35.23 | 17.45233333 | -1.013384541 | 1.30403E-17 | 1.23704E-18 |
| ENSMUSG00000021738 | 10.05066667 | 4.972333333 | -1.01529628 | 8.487E-11 | 1.27387E-11 |
| ENSMUSG00000030094 | 10.10833333 | 4.997 | -1.016411021 | 9.69974E-14 | 1.15923E-14 |
| ENSMUSG00000032855 | 6.471666667 | 3.197666667 | -1.017117745 | 0.008992843 | 0.003550198 |
| ENSMUSG00000055917 | 10.64733333 | 5.255 | -1.018729478 | 1.67449E-08 | 3.06944E-09 |
| ENSMUSG00000041153 | 16.77566667 | 8.275666667 | -1.019422659 | 3.43545E-12 | 4.60622E-13 |
| ENSMUSG00000042523 | 2.211666667 | 1.088 | -1.023455408 | 3.47129E-05 | 9.25581E-06 |
| ENSMUSG00000020437 | 55.881 | 27.48933333 | -1.023485919 | 2.76583E-25 | 1.81601E-26 |
| ENSMUSG00000028893 | 42.355 | 20.83266667 | -1.023684767 | 0.003545349 | 0.001294625 |
| ENSMUSG00000049807 | 4.153666667 | 2.042666667 | -1.023931647 | 8.46194E-09 | 1.50672E-09 |
| ENSMUSG00000021024 | 191.839 | 94.22833333 | -1.025663212 | 2.59271E-35 | 1.21186E-36 |
| ENSMUSG00000038025 | 11.77633333 | 5.776666667 | -1.027581259 | 4.46761E-13 | 5.5984E-14 |
| ENSMUSG00000022744 | 33.915 | 16.62433333 | -1.028627006 | 9.97569E-15 | 1.11989E-15 |

| | | | | | |
|---|---|---|---|---|---|
| ENSMUSG00000024544 | 4.980666667 | 2.441 | -1.028866566 | 3.78179E-06 | 8.95056E-07 |
| ENSMUSG00000029428 | 13.34266667 | 6.536 | -1.029567144 | 6.75892E-16 | 7.08838E-17 |
| ENSMUSG00000026853 | 12.35866667 | 6.053 | -1.029800848 | 6.79513E-11 | 1.01289E-11 |
| ENSMUSG00000020448 | 14.65866667 | 7.174666667 | -1.030770173 | 2.45782E-10 | 3.86389E-11 |
| ENSMUSG00000037103 | 26.80766667 | 13.11133333 | -1.031831248 | 1.19928E-12 | 1.54753E-13 |
| ENSMUSG00000032297 | 2.410666667 | 1.179 | -1.031868459 | 0.002869814 | 0.001029519 |
| ENSMUSG00000028124 | 107.4813333 | 52.54066667 | -1.032579712 | 1.63733E-17 | 1.56679E-18 |
| ENSMUSG00000020823 | 21.76 | 10.63033333 | -1.033491721 | 2.45799E-20 | 2.02457E-21 |
| ENSMUSG00000094484 | 2.698 | 1.318 | -1.033539978 | 0.004352035 | 0.001618342 |
| ENSMUSG00000020048 | 831.2236667 | 405.8776667 | -1.034191773 | 3.19454E-43 | 1.19762E-44 |
| ENSMUSG00000005774 | 11.07033333 | 5.401666667 | -1.035222143 | 5.18004E-15 | 5.73797E-16 |
| ENSMUSG00000117679 | 4.947333333 | 2.414 | -1.03522543 | 3.73947E-05 | 1.00251E-05 |
| ENSMUSG00000031399 | 10.49166667 | 5.116666667 | -1.035967722 | 4.52512E-08 | 8.63221E-09 |
| ENSMUSG00000021420 | 14.988 | 7.309333333 | -1.03599615 | 5.81863E-13 | 7.33556E-14 |
| ENSMUSG00000028687 | 7.228333333 | 3.523 | -1.036858563 | 0.009017105 | 0.003560399 |
| ENSMUSG00000032575 | 250.3696667 | 122.0056667 | -1.037111628 | 2.24793E-38 | 9.57589E-40 |
| ENSMUSG00000025453 | 17.69566667 | 8.621333333 | -1.037413203 | 0.012000591 | 0.004845309 |
| ENSMUSG00000011658 | 5.261666667 | 2.561666667 | -1.038437096 | 0.031342107 | 0.013933432 |
| ENSMUSG00000034292 | 8.708 | 4.239333333 | -1.038503999 | 3.41158E-12 | 4.5695E-13 |
| ENSMUSG00000034538 | 2.606666667 | 1.269 | -1.038514038 | 0.000769187 | 0.000248908 |
| ENSMUSG00000106631 | 31.48 | 15.304 | -1.040526762 | 2.49779E-14 | 2.88167E-15 |
| ENSMUSG00000039512 | 11.61433333 | 5.639666667 | -1.042224546 | 0.000578723 | 0.000183638 |
| ENSMUSG00000035798 | 13.43866667 | 6.523333333 | -1.042708751 | 6.78699E-14 | 8.06435E-15 |
| ENSMUSG00000003458 | 43.287 | 21.004 | -1.043269717 | 4.21826E-31 | 2.24252E-32 |
| ENSMUSG00000030231 | 29.05233333 | 14.096 | -1.043368209 | 2.90932E-21 | 2.29588E-22 |
| ENSMUSG00000017631 | 23.47533333 | 11.379 | -1.044771866 | 2.571E-25 | 1.68098E-26 |
| ENSMUSG00000033701 | 74.04466667 | 35.88666667 | -1.044947899 | 4.36146E-08 | 8.31701E-09 |
| ENSMUSG00000023908 | 20.90366667 | 10.12866667 | -1.045311754 | 9.60933E-11 | 1.44963E-11 |
| ENSMUSG00000024268 | 1.823333333 | 0.883 | -1.046092989 | 0.00184975 | 0.000640593 |
| ENSMUSG00000018507 | 21.99866667 | 10.648 | -1.046833609 | 6.40606E-21 | 5.14378E-22 |
| ENSMUSG00000072825 | 18.81066667 | 9.102 | -1.047295489 | 1.7662E-13 | 2.15349E-14 |
| ENSMUSG00000020577 | 16.12666667 | 7.800333333 | -1.047840587 | 3.54821E-10 | 5.67605E-11 |

| | | | | | |
|---|---|---|---|---|---|
| ENSMUSG00000056749 | 16.34933333 | 7.906 | -1.048211949 | 2.46202E-09 | 4.18156E-10 |
| ENSMUSG00000034445 | 26.43066667 | 12.77633333 | -1.048738957 | 3.71174E-18 | 3.42113E-19 |
| ENSMUSG00000033102 | 1.373333333 | 0.663666667 | -1.049151116 | 0.003180934 | 0.001151672 |
| ENSMUSG00000074358 | 14.41433333 | 6.961666667 | -1.049999471 | 9.29414E-08 | 1.8288E-08 |
| ENSMUSG00000002617 | 10.31633333 | 4.980666667 | -1.050519528 | 1.34926E-10 | 2.07271E-11 |
| ENSMUSG00000041135 | 7.958 | 3.842 | -1.05054838 | 2.44643E-07 | 5.00141E-08 |
| ENSMUSG00000034361 | 88.09 | 42.49833333 | -1.051571989 | 1.76047E-24 | 1.2033E-25 |
| ENSMUSG00000031666 | 24.78433333 | 11.94766667 | -1.05269956 | 3.3463E-25 | 2.20638E-26 |
| ENSMUSG00000021248 | 145.4706667 | 70.11433333 | -1.052946964 | 6.46665E-43 | 2.46004E-44 |
| ENSMUSG00000042148 | 27.29033333 | 13.146 | -1.053766126 | 1.83757E-25 | 1.19003E-26 |
| ENSMUSG00000038312 | 47.18433333 | 22.69333333 | -1.056039383 | 3.5116E-26 | 2.20869E-27 |
| ENSMUSG00000038248 | 2.298333333 | 1.105333333 | -1.056106545 | 5.38725E-05 | 1.48108E-05 |
| ENSMUSG00000038564 | 4.956666667 | 2.381666667 | -1.057398731 | 2.9519E-09 | 5.05027E-10 |
| ENSMUSG00000041571 | 37.967 | 18.23566667 | -1.057983064 | 7.61498E-12 | 1.0494E-12 |
| ENSMUSG00000062590 | 5.055666667 | 2.426333333 | -1.059123582 | 0.006053806 | 0.002304242 |
| ENSMUSG00000014075 | 10.638 | 5.104333333 | -1.059432491 | 6.77441E-05 | 1.8863E-05 |
| ENSMUSG00000100235 | 2.186 | 1.048 | -1.060654684 | 0.000358821 | 0.000110193 |
| ENSMUSG00000028295 | 8.805 | 4.217 | -1.062105982 | 0.001753751 | 0.000604683 |
| ENSMUSG00000028599 | 5.019333333 | 2.403666667 | -1.062258917 | 6.38395E-08 | 1.23545E-08 |
| ENSMUSG00000046324 | 25.20233333 | 12.05766667 | -1.063606559 | 3.67586E-32 | 1.89579E-33 |
| ENSMUSG00000024921 | 57.74066667 | 27.59566667 | -1.065146026 | 3.20226E-34 | 1.56531E-35 |
| ENSMUSG00000002342 | 13.87633333 | 6.631666667 | -1.065183004 | 6.51795E-06 | 1.58449E-06 |
| ENSMUSG00000028382 | 234.6713333 | 112.1426667 | -1.065306336 | 2.64122E-40 | 1.06677E-41 |
| ENSMUSG00000103144 | 2.825 | 1.349 | -1.066360519 | 6.60229E-05 | 1.83473E-05 |
| ENSMUSG00000044098 | 20.76 | 9.913333333 | -1.066364297 | 2.53521E-15 | 2.75155E-16 |
| ENSMUSG00000047746 | 1.126666667 | 0.537333333 | -1.068171503 | 0.001486967 | 0.000506641 |
| ENSMUSG00000029725 | 14.881 | 7.094 | -1.068800243 | 7.68893E-07 | 1.6637E-07 |
| ENSMUSG00000038954 | 15.94766667 | 7.598333333 | -1.069590448 | 1.03588E-08 | 1.85877E-09 |
| ENSMUSG00000037685 | 17.731 | 8.445333333 | -1.070047632 | 2.31231E-11 | 3.32063E-12 |
| ENSMUSG00000102748 | 7.313666667 | 3.483333333 | -1.070126338 | 3.54536E-11 | 5.15012E-12 |
| ENSMUSG00000028641 | 16.842 | 8.020333333 | -1.070329367 | 1.1084E-19 | 9.50454E-21 |
| ENSMUSG00000046753 | 17.592 | 8.375 | -1.070758414 | 2.26276E-16 | 2.30431E-17 |

| | | | | | |
|---|---|---|---|---|---|
| ENSMUSG00000034601 | 9.163333333 | 4.361666667 | -1.070992982 | 6.76287E-13 | 8.56331E-14 |
| ENSMUSG00000068206 | 13.88233333 | 6.605666667 | -1.071474 | 2.47131E-12 | 3.26914E-13 |
| ENSMUSG00000028878 | 19.324 | 9.189666667 | -1.072309321 | 1.07746E-13 | 1.29214E-14 |
| ENSMUSG00000052751 | 7.350666667 | 3.495 | -1.072582645 | 3.56712E-09 | 6.14962E-10 |
| ENSMUSG00000041308 | 3.352666667 | 1.593666667 | -1.072959148 | 3.65125E-10 | 5.84846E-11 |
| ENSMUSG00000060477 | 3.856666667 | 1.832666667 | -1.073410052 | 0.000426209 | 0.000132477 |
| ENSMUSG00000034574 | 12.18066667 | 5.785666667 | -1.074037984 | 1.35208E-12 | 1.75031E-13 |
| ENSMUSG00000037470 | 62.823 | 29.83066667 | -1.074496618 | 6.99178E-30 | 3.87628E-31 |
| ENSMUSG00000015027 | 17.51166667 | 8.314333333 | -1.074643904 | 1.79716E-17 | 1.72222E-18 |
| ENSMUSG00000057969 | 2.311333333 | 1.097 | -1.07516181 | 0.000113597 | 3.2611E-05 |
| ENSMUSG00000039021 | 4.483666667 | 2.128 | -1.075180876 | 3.24204E-05 | 8.61321E-06 |
| ENSMUSG00000020319 | 4.520333333 | 2.145333333 | -1.075227337 | 1.25169E-05 | 3.15689E-06 |
| ENSMUSG00000042050 | 4.35 | 2.061333333 | -1.077437582 | 2.24093E-07 | 4.56416E-08 |
| ENSMUSG00000061046 | 4.854333333 | 2.297 | -1.079522321 | 0.000585296 | 0.000185926 |
| ENSMUSG00000026987 | 28.45033333 | 13.458 | -1.079981529 | 3.98551E-26 | 2.51227E-27 |
| ENSMUSG00000005986 | 2.695333333 | 1.274 | -1.081098425 | 0.000574979 | 0.000182331 |
| ENSMUSG00000017747 | 5.152333333 | 2.434333333 | -1.081699203 | 0.002885477 | 0.001036732 |
| ENSMUSG00000094030 | 23.25033333 | 10.97633333 | -1.0828552 | 5.64935E-12 | 7.69941E-13 |
| ENSMUSG00000019841 | 13.41533333 | 6.331333333 | -1.083301644 | 2.0791E-34 | 1.00768E-35 |
| ENSMUSG00000026307 | 32.64466667 | 15.39933333 | -1.083979415 | 2.53768E-15 | 2.75599E-16 |
| ENSMUSG00000021120 | 14.11 | 6.650333333 | -1.085219428 | 4.42735E-14 | 5.1842E-15 |
| ENSMUSG00000095687 | 63.00033333 | 29.68133333 | -1.08580356 | 3.21529E-25 | 2.11555E-26 |
| ENSMUSG00000034868 | 165.366 | 77.89866667 | -1.0859921 | 6.15203E-37 | 2.75661E-38 |
| ENSMUSG00000059923 | 189.5623333 | 89.14066667 | -1.088516669 | 7.87829E-24 | 5.58617E-25 |
| ENSMUSG00000024579 | 21.483 | 10.101 | -1.088697346 | 4.36715E-12 | 5.90368E-13 |
| ENSMUSG00000034175 | 13.38833333 | 6.295 | -1.088698093 | 2.25953E-08 | 4.1903E-09 |
| ENSMUSG00000004931 | 21.853 | 10.27066667 | -1.089301518 | 4.38569E-14 | 5.13239E-15 |
| ENSMUSG00000094724 | 97.59466667 | 45.84733333 | -1.089964487 | 2.20872E-21 | 1.72776E-22 |
| ENSMUSG00000005893 | 21.43433333 | 10.06633333 | -1.090385269 | 6.08824E-26 | 3.86715E-27 |
| ENSMUSG00000052609 | 21.80866667 | 10.23966667 | -1.090732818 | 6.78504E-25 | 4.5346E-26 |
| ENSMUSG00000027215 | 19.63833333 | 9.219 | -1.090990323 | 1.14201E-12 | 1.46969E-13 |
| ENSMUSG00000005949 | 16.845 | 7.905333333 | -1.091422227 | 3.44627E-17 | 3.3668E-18 |

| | | | | | |
|---|---|---|---|---|---|
| ENSMUSG00000021066 | 0.812666667 | 0.381333333 | -1.091611074 | 0.002672599 | 0.000955307 |
| ENSMUSG00000021234 | 1.265333333 | 0.593666667 | -1.091792476 | 0.008388433 | 0.003293635 |
| ENSMUSG00000034156 | 4.371333333 | 2.049666667 | -1.092684088 | 1.41399E-05 | 3.58965E-06 |
| ENSMUSG00000046792 | 19.41666667 | 9.099333333 | -1.093462794 | 4.43075E-09 | 7.71191E-10 |
| ENSMUSG00000034371 | 17.39366667 | 8.147333333 | -1.094162252 | 4.9075E-12 | 6.66734E-13 |
| ENSMUSG00000064368 | 914.4783333 | 428.339 | -1.094195949 | 2.77114E-14 | 3.2085E-15 |
| ENSMUSG00000031458 | 6.543333333 | 3.063666667 | -1.094766429 | 0.003840482 | 0.001413268 |
| ENSMUSG00000061536 | 9.160666667 | 4.288666667 | -1.094923406 | 3.35267E-09 | 5.75675E-10 |
| ENSMUSG00000031791 | 9.019333333 | 4.217666667 | -1.096575721 | 1.6403E-06 | 3.72364E-07 |
| ENSMUSG00000042684 | 17.15833333 | 8.023333333 | -1.096635783 | 4.83492E-11 | 7.13021E-12 |
| ENSMUSG00000033720 | 0.996 | 0.465666667 | -1.096848127 | 0.006501736 | 0.002494936 |
| ENSMUSG00000004788 | 52.78133333 | 24.672 | -1.097153125 | 1.02628E-30 | 5.54095E-32 |
| ENSMUSG00000052369 | 26.063 | 12.17433333 | -1.098160383 | 1.18638E-13 | 1.42769E-14 |
| ENSMUSG00000024944 | 15.99466667 | 7.461 | -1.100150013 | 1.7806E-07 | 3.60079E-08 |
| ENSMUSG00000099689 | 0.956333333 | 0.446 | -1.100469852 | 0.034347817 | 0.015428535 |
| ENSMUSG00000005682 | 9.424333333 | 4.391 | -1.101841033 | 5.97672E-15 | 6.63118E-16 |
| ENSMUSG00000055150 | 2.757 | 1.284 | -1.102454065 | 0.012632599 | 0.005120546 |
| ENSMUSG00000012117 | 35.29366667 | 16.42666667 | -1.103369564 | 3.64534E-27 | 2.22234E-28 |
| ENSMUSG00000063659 | 16.824 | 7.827 | -1.103989405 | 3.23504E-18 | 2.96843E-19 |
| ENSMUSG00000028378 | 19.86633333 | 9.225666667 | -1.106600551 | 1.068E-13 | 1.27933E-14 |
| ENSMUSG00000035151 | 9.444 | 4.381333333 | -1.108028062 | 1.6372E-10 | 2.5399E-11 |
| ENSMUSG00000069633 | 3.765666667 | 1.746333333 | -1.108576338 | 2.7464E-05 | 7.22059E-06 |
| ENSMUSG00000036934 | 29.50466667 | 13.68166667 | -1.108699173 | 1.24294E-11 | 1.74119E-12 |
| ENSMUSG00000017718 | 5.096666667 | 2.362666667 | -1.109137898 | 1.81751E-05 | 4.67555E-06 |
| ENSMUSG00000021366 | 11.55833333 | 5.348333333 | -1.111772093 | 2.34455E-21 | 1.83724E-22 |
| ENSMUSG00000026585 | 14.27066667 | 6.595 | -1.113608169 | 7.0778E-23 | 5.18472E-24 |
| ENSMUSG00000031822 | 54.89433333 | 25.35933333 | -1.11414041 | 7.10264E-24 | 5.01657E-25 |
| ENSMUSG00000031967 | 41.46266667 | 19.14833333 | -1.11459408 | 2.63458E-21 | 2.07362E-22 |
| ENSMUSG00000025068 | 223.21 | 102.988 | -1.115925416 | 1.05506E-42 | 4.0355E-44 |
| ENSMUSG00000015599 | 2.959 | 1.365 | -1.116208745 | 5.39571E-07 | 1.14813E-07 |
| ENSMUSG00000038615 | 60.13 | 27.71366667 | -1.117487359 | 2.37585E-19 | 2.06353E-20 |
| ENSMUSG00000070709 | 2.415333333 | 1.113 | -1.117768712 | 0.043425983 | 0.020019006 |

| | | | | | |
|---|---|---|---|---|---|
| ENSMUSG00000024642 | 40.13133333 | 18.48766667 | -1.118165936 | 1.15134E-32 | 5.85845E-34 |
| ENSMUSG00000027931 | 3.125 | 1.439333333 | -1.118455447 | 2.67126E-06 | 6.19495E-07 |
| ENSMUSG00000040822 | 28.30733333 | 13.03733333 | -1.118527038 | 1.06041E-11 | 1.47376E-12 |
| ENSMUSG00000052593 | 25.649 | 11.801 | -1.119993463 | 1.52214E-24 | 1.03725E-25 |
| ENSMUSG00000078865 | 9.19 | 4.228 | -1.120089485 | 3.03236E-07 | 6.25984E-08 |
| ENSMUSG00000018599 | 4.568666667 | 2.101666667 | -1.120239316 | 5.47614E-05 | 1.50779E-05 |
| ENSMUSG00000030035 | 29.422 | 13.531 | -1.120626854 | 4.50803E-16 | 4.67486E-17 |
| ENSMUSG00000021585 | 70.31933333 | 32.32433333 | -1.121300777 | 2.79776E-18 | 2.55167E-19 |
| ENSMUSG00000031570 | 37.298 | 17.137 | -1.121983698 | 1.26006E-20 | 1.0283E-21 |
| ENSMUSG00000020821 | 23.60233333 | 10.84166667 | -1.122342936 | 1.22588E-27 | 7.33799E-29 |
| ENSMUSG00000084883 | 4.432666667 | 2.033 | -1.124564662 | 8.71188E-07 | 1.90249E-07 |
| ENSMUSG00000051650 | 108.7096667 | 49.82633333 | -1.125499917 | 4.21291E-26 | 2.66143E-27 |
| ENSMUSG00000031629 | 25.64233333 | 11.742 | -1.126849385 | 7.28769E-07 | 1.57336E-07 |
| ENSMUSG00000038212 | 51.02533333 | 23.35133333 | -1.127708774 | 5.77718E-27 | 3.56587E-28 |
| ENSMUSG00000027381 | 14.563 | 6.663666667 | -1.127919443 | 1.10956E-13 | 1.33218E-14 |
| ENSMUSG00000024127 | 27.37966667 | 12.524 | -1.128409469 | 2.46711E-28 | 1.43421E-29 |
| ENSMUSG00000020283 | 15.367 | 7.017666667 | -1.130772217 | 1.10144E-12 | 1.41368E-13 |
| ENSMUSG00000035133 | 34.29133333 | 15.65333333 | -1.131374093 | 1.37859E-30 | 7.48115E-32 |
| ENSMUSG00000087403 | 4.486 | 2.047666667 | -1.131448738 | 3.3733E-08 | 6.3558E-09 |
| ENSMUSG00000047648 | 12.677 | 5.785 | -1.131824509 | 6.82416E-18 | 6.36055E-19 |
| ENSMUSG00000059689 | 8.333 | 3.799666667 | -1.132963119 | 0.001963705 | 0.000682633 |
| ENSMUSG00000048478 | 1.493 | 0.680666667 | -1.1331938 | 0.007606601 | 0.002954622 |
| ENSMUSG00000038170 | 9.977 | 4.544666667 | -1.134431592 | 8.85883E-22 | 6.80131E-23 |
| ENSMUSG00000029165 | 12.429 | 5.661 | -1.134581397 | 1.80134E-08 | 3.31191E-09 |
| ENSMUSG00000042249 | 4.76 | 2.167666667 | -1.13481865 | 6.61428E-14 | 7.85458E-15 |
| ENSMUSG00000019977 | 58.72466667 | 26.74066667 | -1.134931186 | 2.14607E-39 | 8.86048E-41 |
| ENSMUSG00000028576 | 6.397 | 2.912 | -1.135385128 | 1.78872E-07 | 3.61967E-08 |
| ENSMUSG00000018425 | 26.70266667 | 12.15166667 | -1.135829623 | 3.40789E-32 | 1.75524E-33 |
| ENSMUSG00000036534 | 12.89333333 | 5.867 | -1.135930397 | 3.13732E-16 | 3.22309E-17 |
| ENSMUSG00000027366 | 37.893 | 17.232 | -1.136841208 | 9.98504E-28 | 5.94939E-29 |
| ENSMUSG00000048481 | 5.001333333 | 2.274 | -1.137080508 | 2.53371E-05 | 6.63865E-06 |
| ENSMUSG00000016528 | 137.6013333 | 62.54033333 | -1.137635636 | 4.74612E-64 | 1.14688E-65 |

| | | | | | |
|---|---|---|---|---|---|
| ENSMUSG00000042647 | 3.85 | 1.749 | -1.138328157 | 3.56592E-09 | 6.14508E-10 |
| ENSMUSG00000008393 | 20.73133333 | 9.417666667 | -1.138371341 | 3.37595E-20 | 2.80164E-21 |
| ENSMUSG00000040359 | 23.23666667 | 10.555 | -1.138476548 | 1.15345E-23 | 8.2344E-25 |
| ENSMUSG00000024052 | 15.21066667 | 6.907666667 | -1.138813015 | 1.31629E-17 | 1.25049E-18 |
| ENSMUSG00000026672 | 18.32733333 | 8.319333333 | -1.139457058 | 8.69372E-15 | 9.72972E-16 |
| ENSMUSG00000025888 | 5.233 | 2.372666667 | -1.141128827 | 0.000381802 | 0.000117699 |
| ENSMUSG00000041491 | 20.318 | 9.208666667 | -1.14169421 | 8.49067E-21 | 6.8528E-22 |
| ENSMUSG00000038936 | 21.32566667 | 9.660333333 | -1.142445967 | 2.81293E-17 | 2.73447E-18 |
| ENSMUSG00000061533 | 23.27166667 | 10.54133333 | -1.142517178 | 2.13235E-16 | 2.1671E-17 |
| ENSMUSG00000067889 | 1.697333333 | 0.768333333 | -1.143465669 | 1.59977E-07 | 3.2119E-08 |
| ENSMUSG00000052296 | 138.865 | 62.78866667 | -1.145106942 | 6.53556E-52 | 2.00344E-53 |
| ENSMUSG00000020393 | 7.807 | 3.528 | -1.145917709 | 1.38634E-11 | 1.95259E-12 |
| ENSMUSG00000020097 | 29.132 | 13.161 | -1.146335638 | 2.7728E-32 | 1.42048E-33 |
| ENSMUSG00000034243 | 28.843 | 13.02366667 | -1.147085549 | 1.76746E-32 | 9.00568E-34 |
| ENSMUSG00000001173 | 18.04433333 | 8.136 | -1.149154258 | 6.93943E-14 | 8.25027E-15 |
| ENSMUSG00000022244 | 3.230666667 | 1.454666667 | -1.151143303 | 0.000213539 | 6.36607E-05 |
| ENSMUSG00000032182 | 11.95666667 | 5.382 | -1.151600948 | 4.71483E-09 | 8.22264E-10 |
| ENSMUSG00000003123 | 6.111333333 | 2.749666667 | -1.152230437 | 2.67606E-09 | 4.56541E-10 |
| ENSMUSG00000063535 | 2.399666667 | 1.079333333 | -1.152693533 | 5.27756E-06 | 1.27093E-06 |
| ENSMUSG00000058756 | 19.73 | 8.872 | -1.153059685 | 2.958E-14 | 3.43302E-15 |
| ENSMUSG00000025094 | 139.5266667 | 62.69266667 | -1.154172278 | 2.80925E-18 | 2.56409E-19 |
| ENSMUSG00000028552 | 108.8133333 | 48.87133333 | -1.154794976 | 3.46793E-53 | 1.03195E-54 |
| ENSMUSG00000035713 | 0.961 | 0.431333333 | -1.15573322 | 0.003622059 | 0.001324887 |
| ENSMUSG00000089832 | 23.847 | 10.70333333 | -1.155747619 | 1.24785E-21 | 9.64922E-23 |
| ENSMUSG00000039354 | 26.11533333 | 11.71466667 | -1.156581214 | 3.39728E-21 | 2.70206E-22 |
| ENSMUSG00000039166 | 4.451666667 | 1.996666667 | -1.156752068 | 0.045018924 | 0.020837259 |
| ENSMUSG00000021235 | 13.96833333 | 6.256333333 | -1.158770607 | 1.06364E-08 | 1.91152E-09 |
| ENSMUSG00000021036 | 27.01333333 | 12.07633333 | -1.161489189 | 5.36363E-35 | 2.53665E-36 |
| ENSMUSG00000020284 | 4.623333333 | 2.066666667 | -1.161627667 | 4.82221E-05 | 1.31675E-05 |
| ENSMUSG00000024646 | 40.98766667 | 18.30033333 | -1.163319936 | 0.01839888 | 0.007761472 |
| ENSMUSG00000036006 | 19.65833333 | 8.770333333 | -1.164437432 | 1.21232E-22 | 8.96436E-24 |
| ENSMUSG00000003849 | 6.813666667 | 3.037666667 | -1.165467804 | 3.36299E-06 | 7.90364E-07 |

| | | | | | |
|---|---|---|---|---|---|
| ENSMUSG00000030780 | 11.29766667 | 5.035 | -1.165961157 | 2.94324E-15 | 3.21066E-16 |
| ENSMUSG00000059883 | 7.071666667 | 3.150666667 | -1.166393144 | 5.43372E-10 | 8.81985E-11 |
| ENSMUSG00000021365 | 20.33266667 | 9.056666667 | -1.166747375 | 6.65193E-29 | 3.80727E-30 |
| ENSMUSG00000002763 | 19.89133333 | 8.852 | -1.168064628 | 1.40302E-22 | 1.04132E-23 |
| ENSMUSG00000024845 | 28.05533333 | 12.485 | -1.168079233 | 1.74998E-11 | 2.48409E-12 |
| ENSMUSG00000028701 | 2.067 | 0.919333333 | -1.168878433 | 9.03709E-06 | 2.23993E-06 |
| ENSMUSG00000035172 | 5.202666667 | 2.313333333 | -1.169278115 | 1.48043E-08 | 2.70349E-09 |
| ENSMUSG00000026027 | 8.023 | 3.567333333 | -1.169295769 | 3.15548E-10 | 5.02166E-11 |
| ENSMUSG00000049858 | 10.65166667 | 4.733666667 | -1.170049163 | 5.33296E-08 | 1.02432E-08 |
| ENSMUSG00000066735 | 34.51566667 | 15.337 | -1.170235039 | 1.0478E-20 | 8.52912E-22 |
| ENSMUSG00000026209 | 86.70733333 | 38.50933333 | -1.170945868 | 2.07435E-28 | 1.20015E-29 |
| ENSMUSG00000033634 | 0.734666667 | 0.325666667 | -1.173693757 | 0.021639762 | 0.009275038 |
| ENSMUSG00000042275 | 25.39466667 | 11.254 | -1.174087669 | 4.31501E-12 | 5.83021E-13 |
| ENSMUSG00000022442 | 16.218 | 7.180333333 | -1.175473193 | 3.61338E-12 | 4.85227E-13 |
| ENSMUSG00000045980 | 8.965666667 | 3.963666667 | -1.177575222 | 1.20721E-17 | 1.1427E-18 |
| ENSMUSG00000035126 | 0.834666667 | 0.369 | -1.17757934 | 0.042250318 | 0.019392441 |
| ENSMUSG00000031398 | 0.825666667 | 0.365 | -1.177662999 | 0.000684997 | 0.000220009 |
| ENSMUSG00000032492 | 1.248666667 | 0.551666667 | -1.178519683 | 0.013564666 | 0.005538624 |
| ENSMUSG00000022999 | 6.205 | 2.740666667 | -1.178904339 | 4.23581E-08 | 8.05694E-09 |
| ENSMUSG00000021123 | 4.129666667 | 1.823666667 | -1.179183282 | 0.001575268 | 0.000538793 |
| ENSMUSG00000021113 | 4.827333333 | 2.130666667 | -1.179921543 | 2.19418E-08 | 4.0675E-09 |
| ENSMUSG00000046387 | 0.486 | 0.214333333 | -1.181100077 | 0.047350291 | 0.022034035 |
| ENSMUSG00000057103 | 2.711666667 | 1.195666667 | -1.181364601 | 0.02780993 | 0.012217246 |
| ENSMUSG00000036109 | 14.00033333 | 6.169666667 | -1.182196726 | 4.61649E-24 | 3.22555E-25 |
| ENSMUSG00000020715 | 22.05733333 | 9.716333333 | -1.182774494 | 6.36861E-20 | 5.39072E-21 |
| ENSMUSG00000045795 | 16.75766667 | 7.379666667 | -1.183193725 | 2.19693E-17 | 2.122E-18 |
| ENSMUSG00000007837 | 2.867 | 1.26 | -1.186118171 | 0.006814334 | 0.002626182 |
| ENSMUSG00000039199 | 2.149333333 | 0.942333333 | -1.189579861 | 0.001277213 | 0.000428824 |
| ENSMUSG00000055341 | 1.097333333 | 0.480666667 | -1.190893171 | 0.019969607 | 0.008486118 |
| ENSMUSG00000085793 | 11.35633333 | 4.970333333 | -1.192082587 | 4.57129E-14 | 5.36221E-15 |
| ENSMUSG00000041168 | 108.687 | 47.549 | -1.192692485 | 1.02205E-24 | 6.90826E-26 |
| ENSMUSG00000021203 | 9.947 | 4.349 | -1.193577768 | 2.81646E-09 | 4.80883E-10 |

| | | | | | |
|---|---|---|---|---|---|
| ENSMUSG00000039158 | 6.567333333 | 2.869666667 | -1.194424516 | 1.93144E-13 | 2.36029E-14 |
| ENSMUSG00000020733 | 82.21 | 35.87333333 | -1.196402088 | 2.8141E-45 | 9.94766E-47 |
| ENSMUSG00000029270 | 7.491 | 3.261666667 | -1.199548972 | 1.65134E-07 | 3.31887E-08 |
| ENSMUSG00000043510 | 16.39766667 | 7.137666667 | -1.199966105 | 4.23126E-07 | 8.87794E-08 |
| ENSMUSG00000033955 | 1.509666667 | 0.657 | -1.200264763 | 0.000101485 | 2.89096E-05 |
| ENSMUSG00000002043 | 41.101 | 17.88133333 | -1.20071918 | 2.31192E-15 | 2.50282E-16 |
| ENSMUSG00000025571 | 23.68433333 | 10.30366667 | -1.200775237 | 2.52027E-37 | 1.10318E-38 |
| ENSMUSG00000020635 | 18.97733333 | 8.255333333 | -1.200878907 | 0.000304598 | 9.24687E-05 |
| ENSMUSG00000022861 | 9.756 | 4.242666667 | -1.201318423 | 5.73403E-16 | 5.99373E-17 |
| ENSMUSG00000042759 | 3.625333333 | 1.574666667 | -1.203067187 | 1.18673E-08 | 2.14503E-09 |
| ENSMUSG00000024457 | 12.00133333 | 5.210666667 | -1.203654825 | 8.27928E-16 | 8.71142E-17 |
| ENSMUSG00000020895 | 6.133 | 2.662666667 | -1.203721119 | 0.007768995 | 0.003024673 |
| ENSMUSG00000020668 | 7.766333333 | 3.369 | -1.204913201 | 3.94887E-10 | 6.34971E-11 |
| ENSMUSG00000039156 | 30.97833333 | 13.42633333 | -1.206194162 | 3.96365E-32 | 2.05243E-33 |
| ENSMUSG00000028799 | 28.46133333 | 12.32 | -1.208000994 | 3.83887E-29 | 2.17334E-30 |
| ENSMUSG00000057156 | 3.985333333 | 1.725 | -1.208104034 | 0.005655519 | 0.002141319 |
| ENSMUSG00000047227 | 2.558333333 | 1.107 | -1.208549028 | 0.040591124 | 0.018549617 |
| ENSMUSG00000060147 | 9.944333333 | 4.297666667 | -1.210321067 | 4.05658E-06 | 9.64497E-07 |
| ENSMUSG00000022394 | 35.64366667 | 15.403 | -1.210434387 | 3.84897E-39 | 1.60507E-40 |
| ENSMUSG00000034744 | 28.18133333 | 12.17566667 | -1.210739103 | 4.25285E-19 | 3.7349E-20 |
| ENSMUSG00000031901 | 9.059666667 | 3.914 | -1.210814214 | 5.69084E-07 | 1.21369E-07 |
| ENSMUSG00000041890 | 40.033 | 17.29233333 | -1.211057181 | 1.69514E-22 | 1.26398E-23 |
| ENSMUSG00000049285 | 2.729 | 1.178 | -1.212032855 | 0.003865625 | 0.001423588 |
| ENSMUSG00000038520 | 12.85466667 | 5.541666667 | -1.21390036 | 2.64888E-09 | 4.51119E-10 |
| ENSMUSG00000034917 | 14.672 | 6.320333333 | -1.214992991 | 4.47698E-13 | 5.61323E-14 |
| ENSMUSG00000095545 | 53.859 | 23.19466667 | -1.21539433 | 1.705E-25 | 1.09829E-26 |
| ENSMUSG00000029403 | 2.601666667 | 1.120333333 | -1.21550809 | 1.64267E-06 | 3.73014E-07 |
| ENSMUSG00000044456 | 38.972 | 16.76133333 | -1.217301054 | 1.41047E-19 | 1.21532E-20 |
| ENSMUSG00000055210 | 1.726333333 | 0.742333333 | -1.217572 | 0.002606083 | 0.000928251 |
| ENSMUSG00000064120 | 12.324 | 5.291666667 | -1.219676496 | 3.40522E-12 | 4.55863E-13 |
| ENSMUSG00000020908 | 2.919333333 | 1.253333333 | -1.219868788 | 1.01491E-07 | 2.00685E-08 |
| ENSMUSG00000022911 | 14.12066667 | 6.061666667 | -1.220021778 | 3.21464E-18 | 2.94742E-19 |

| | | | | | |
|---|---|---|---|---|---|
| ENSMUSG00000001583 | 1.073333333 | 0.460333333 | -1.221347369 | 0.008077673 | 0.003158792 |
| ENSMUSG00000032298 | 6.592333333 | 2.825666667 | -1.222197906 | 3.03963E-06 | 7.10381E-07 |
| ENSMUSG00000021514 | 14.86933333 | 6.368 | -1.223427725 | 6.9093E-12 | 9.47858E-13 |
| ENSMUSG00000096463 | 17.52966667 | 7.506 | -1.223682368 | 2.06256E-09 | 3.48743E-10 |
| ENSMUSG00000070732 | 3.338333333 | 1.426666667 | -1.226479719 | 3.52453E-06 | 8.30762E-07 |
| ENSMUSG00000032468 | 26.15633333 | 11.17333333 | -1.227100666 | 9.85169E-36 | 4.57079E-37 |
| ENSMUSG00000037221 | 16.41133333 | 7.007 | -1.227823654 | 1.5327E-06 | 3.4709E-07 |
| ENSMUSG00000015363 | 60.82966667 | 25.971 | -1.227873533 | 1.70126E-38 | 7.21193E-40 |
| ENSMUSG00000020659 | 21.35566667 | 9.107666667 | -1.22946554 | 2.53506E-20 | 2.0898E-21 |
| ENSMUSG00000013663 | 63.73466667 | 27.17266667 | -1.229922144 | 4.71449E-62 | 1.18481E-63 |
| ENSMUSG00000001123 | 136.085 | 58.00933333 | -1.23015111 | 1.04319E-25 | 6.69102E-27 |
| ENSMUSG00000021238 | 10.29866667 | 4.388333333 | -1.230712548 | 1.14442E-15 | 1.21363E-16 |
| ENSMUSG00000021182 | 22.42866667 | 9.555666667 | -1.230915424 | 8.16899E-18 | 7.63093E-19 |
| ENSMUSG00000020877 | 6.962333333 | 2.965666667 | -1.231214435 | 1.13147E-07 | 2.24123E-08 |
| ENSMUSG00000039623 | 11.66466667 | 4.951666667 | -1.236158977 | 4.43224E-13 | 5.55101E-14 |
| ENSMUSG00000038763 | 5.585666667 | 2.369666667 | -1.237045347 | 1.14285E-13 | 1.37372E-14 |
| ENSMUSG00000024781 | 29.83833333 | 12.642 | -1.238942234 | 1.24665E-30 | 6.75657E-32 |
| ENSMUSG00000008305 | 41.48733333 | 17.55366667 | -1.240898512 | 2.82997E-25 | 1.86007E-26 |
| ENSMUSG00000042207 | 30.11066667 | 12.73466667 | -1.241513453 | 3.16585E-52 | 9.66104E-54 |
| ENSMUSG00000024006 | 47.59533333 | 20.12866667 | -1.241568515 | 6.30902E-40 | 2.5656E-41 |
| ENSMUSG00000038811 | 5.216333333 | 2.206 | -1.241603272 | 0.013658838 | 0.005585436 |
| ENSMUSG00000025893 | 1.265333333 | 0.534333333 | -1.243705567 | 0.010830707 | 0.004334825 |
| ENSMUSG00000028256 | 13.21566667 | 5.578333333 | -1.244343154 | 3.7758E-11 | 5.50564E-12 |
| ENSMUSG00000034993 | 110.0996667 | 46.468 | -1.244500644 | 7.2792E-54 | 2.12084E-55 |
| ENSMUSG00000003344 | 14.725 | 6.212333333 | -1.245060487 | 7.08737E-13 | 8.98888E-14 |
| ENSMUSG00000045282 | 6.923666667 | 2.915333333 | -1.247875422 | 4.02927E-05 | 1.0866E-05 |
| ENSMUSG00000024963 | 11.174 | 4.700333333 | -1.249310748 | 7.66996E-08 | 1.49597E-08 |
| ENSMUSG00000021884 | 13.495 | 5.676333333 | -1.24939376 | 7.30632E-09 | 1.28986E-09 |
| ENSMUSG00000029004 | 32.959 | 13.81533333 | -1.254402099 | 2.14283E-48 | 7.01259E-50 |
| ENSMUSG00000030671 | 26.759 | 11.21333333 | -1.254808998 | 5.47038E-25 | 3.6371E-26 |
| ENSMUSG00000021900 | 13.88 | 5.807 | -1.257142629 | 1.25191E-18 | 1.12192E-19 |
| ENSMUSG00000001441 | 47.576 | 19.89266667 | -1.257997294 | 7.12114E-36 | 3.28917E-37 |

| | | | | | |
|---|---|---|---|---|---|
| ENSMUSG00000037410 | 15.43733333 | 6.443 | -1.260619061 | 1.39583E-35 | 6.49539E-37 |
| ENSMUSG00000020873 | 58.72433333 | 24.49233333 | -1.261628207 | 1.04769E-30 | 5.6638E-32 |
| ENSMUSG00000042766 | 4.077666667 | 1.698333333 | -1.2636242 | 8.16913E-07 | 1.77551E-07 |
| ENSMUSG00000024143 | 13.30533333 | 5.54 | -1.264046773 | 2.06858E-25 | 1.34249E-26 |
| ENSMUSG00000046070 | 2.997333333 | 1.248 | -1.264061601 | 7.55988E-06 | 1.85187E-06 |
| ENSMUSG00000062901 | 17.62666667 | 7.335333333 | -1.264825244 | 2.67522E-25 | 1.75097E-26 |
| ENSMUSG00000035311 | 27.498 | 11.393 | -1.271179005 | 2.86504E-31 | 1.51718E-32 |
| ENSMUSG00000021611 | 1.225666667 | 0.507666667 | -1.271613235 | 0.00142144 | 0.000481861 |
| ENSMUSG00000034723 | 22.765 | 9.423 | -1.272559111 | 1.30845E-49 | 4.16457E-51 |
| ENSMUSG00000089876 | 7.383 | 3.055666667 | -1.272719986 | 4.60272E-07 | 9.71134E-08 |
| ENSMUSG00000045775 | 1.634666667 | 0.676333333 | -1.273190114 | 0.015240566 | 0.006301833 |
| ENSMUSG00000016534 | 71.54433333 | 29.55466667 | -1.275451554 | 8.08768E-32 | 4.24933E-33 |
| ENSMUSG00000021176 | 7.642333333 | 3.154666667 | -1.276525612 | 0.000102618 | 2.92606E-05 |
| ENSMUSG00000006732 | 6.487333333 | 2.674666667 | -1.278266464 | 1.19302E-06 | 2.66132E-07 |
| ENSMUSG00000022604 | 12.13233333 | 4.998 | -1.279434235 | 1.53029E-18 | 1.37561E-19 |
| ENSMUSG00000001482 | 42.271 | 17.39066667 | -1.281355003 | 4.70107E-22 | 3.56378E-23 |
| ENSMUSG00000021140 | 9.951333333 | 4.090666667 | -1.282553857 | 8.35551E-38 | 3.59395E-39 |
| ENSMUSG00000091945 | 1.674666667 | 0.687 | -1.285491959 | 3.97429E-07 | 8.30309E-08 |
| ENSMUSG00000028779 | 39.67566667 | 16.26833333 | -1.286188008 | 2.70791E-25 | 1.77543E-26 |
| ENSMUSG00000021661 | 15.42666667 | 6.321 | -1.287201644 | 1.70735E-15 | 1.83772E-16 |
| ENSMUSG00000020919 | 33.96766667 | 13.913 | -1.287728583 | 3.00515E-51 | 9.33664E-53 |
| ENSMUSG00000002032 | 1.358333333 | 0.556333333 | -1.287816105 | 0.027754502 | 0.012189063 |
| ENSMUSG00000056531 | 6.489 | 2.657333333 | -1.288016957 | 1.40433E-13 | 1.69966E-14 |
| ENSMUSG00000001542 | 45.39733333 | 18.589 | -1.288158393 | 9.11428E-38 | 3.93291E-39 |
| ENSMUSG00000079710 | 3.066 | 1.255333333 | -1.288287198 | 0.012115744 | 0.004893476 |
| ENSMUSG00000024666 | 18.86966667 | 7.722333333 | -1.288960203 | 4.83009E-14 | 5.68246E-15 |
| ENSMUSG00000035967 | 85.89733333 | 35.118 | -1.290402659 | 7.91283E-33 | 4.00995E-34 |
| ENSMUSG00000066894 | 3.588666667 | 1.466666667 | -1.290906902 | 1.34999E-08 | 2.45317E-09 |
| ENSMUSG00000004508 | 22.65966667 | 9.251666667 | -1.292341446 | 4.19811E-21 | 3.35351E-22 |
| ENSMUSG00000054793 | 1.661 | 0.677666667 | -1.293404359 | 0.007353218 | 0.002851124 |
| ENSMUSG00000031103 | 16.13766667 | 6.581666667 | -1.293907128 | 1.1596E-22 | 8.56649E-24 |
| ENSMUSG00000022742 | 37.81333333 | 15.412 | -1.294840941 | 6.63048E-44 | 2.43997E-45 |

| | | | | | |
|---|---|---|---|---|---|
| ENSMUSG00000026223 | 50.47966667 | 20.574 | -1.294880074 | 2.97815E-38 | 1.27071E-39 |
| ENSMUSG00000038797 | 4.282666667 | 1.744333333 | -1.295833634 | 0.00102729 | 0.000339096 |
| ENSMUSG00000036214 | 6.008 | 2.446 | -1.296460409 | 0.000329507 | 0.000100531 |
| ENSMUSG00000039633 | 4.577666667 | 1.862 | -1.297759341 | 5.36314E-10 | 8.69418E-11 |
| ENSMUSG00000024308 | 40.569 | 16.49366667 | -1.298465585 | 3.38634E-47 | 1.14795E-48 |
| ENSMUSG00000040209 | 8.737333333 | 3.551333333 | -1.298832251 | 1.35549E-21 | 1.05096E-22 |
| ENSMUSG00000029516 | 29.718 | 12.07266667 | -1.299592646 | 1.5383E-12 | 2.00306E-13 |
| ENSMUSG00000022359 | 12.09166667 | 4.909333333 | -1.300414082 | 3.20933E-12 | 4.29196E-13 |
| ENSMUSG00000033467 | 10.542 | 4.276 | -1.301814839 | 3.47072E-10 | 5.5473E-11 |
| ENSMUSG00000079083 | 7.170666667 | 2.903333333 | -1.304397036 | 1.94857E-14 | 2.2292E-15 |
| ENSMUSG00000074794 | 8.189666667 | 3.311333333 | -1.306392487 | 1.36318E-11 | 1.91621E-12 |
| ENSMUSG00000040441 | 0.907 | 0.366666667 | -1.306633433 | 0.013641423 | 0.005574674 |
| ENSMUSG00000039740 | 29.71333333 | 11.981 | -1.310362132 | 1.74412E-39 | 7.17683E-41 |
| ENSMUSG00000039081 | 4.614666667 | 1.858333333 | -1.312217136 | 1.14401E-12 | 1.47305E-13 |
| ENSMUSG00000029311 | 48.645 | 19.581 | -1.312837079 | 2.37245E-20 | 1.95247E-21 |
| ENSMUSG00000016206 | 11.17333333 | 4.493333333 | -1.314201653 | 1.77794E-10 | 2.76805E-11 |
| ENSMUSG00000038065 | 2.822333333 | 1.134333333 | -1.315043738 | 6.33073E-07 | 1.35627E-07 |
| ENSMUSG00000031970 | 4.334 | 1.740333333 | -1.316335495 | 5.14955E-05 | 1.41254E-05 |
| ENSMUSG00000060376 | 27.41333333 | 10.987 | -1.319080252 | 3.47188E-30 | 1.90565E-31 |
| ENSMUSG00000045559 | 0.346333333 | 0.138666667 | -1.320540221 | 0.008083551 | 0.003161648 |
| ENSMUSG00000013155 | 17.202 | 6.879666667 | -1.32216574 | 1.60168E-14 | 1.82462E-15 |
| ENSMUSG00000026923 | 2.14 | 0.855666667 | -1.322490001 | 1.68811E-09 | 2.83567E-10 |
| ENSMUSG00000074272 | 12.85433333 | 5.138666667 | -1.322788813 | 9.36955E-17 | 9.28287E-18 |
| ENSMUSG00000072889 | 25.70433333 | 10.26966667 | -1.32362224 | 3.92387E-36 | 1.80426E-37 |
| ENSMUSG00000031865 | 117.057 | 46.76033333 | -1.323854091 | 5.77255E-34 | 2.83765E-35 |
| ENSMUSG00000040502 | 6.676 | 2.664666667 | -1.325028883 | 2.85048E-11 | 4.12103E-12 |
| ENSMUSG00000003934 | 0.664666667 | 0.265 | -1.326638644 | 0.023673863 | 0.010246579 |
| ENSMUSG00000018474 | 81.94666667 | 32.66 | -1.327160475 | 2.56535E-76 | 5.22493E-78 |
| ENSMUSG00000035504 | 9.307333333 | 3.703666667 | -1.329413616 | 2.72643E-10 | 4.3144E-11 |
| ENSMUSG00000033862 | 24.99566667 | 9.932333333 | -1.331473421 | 1.02574E-19 | 8.7815E-21 |
| ENSMUSG00000071552 | 2.317 | 0.919666667 | -1.333075088 | 0.043020819 | 0.019808467 |
| ENSMUSG00000022802 | 1.497333333 | 0.593666667 | -1.334670411 | 3.27896E-07 | 6.79154E-08 |

| | | | | | |
|---|---|---|---|---|---|
| ENSMUSG00000037971 | 6.118 | 2.422666667 | -1.336464188 | 0.000121364 | 3.49413E-05 |
| ENSMUSG00000037822 | 27.12433333 | 10.711 | -1.3404945 | 1.40731E-12 | 1.82764E-13 |
| ENSMUSG00000003762 | 5.397666667 | 2.13 | -1.341482455 | 1.63176E-08 | 2.9866E-09 |
| ENSMUSG00000032606 | 9.718666667 | 3.829333333 | -1.343665151 | 3.41455E-11 | 4.95303E-12 |
| ENSMUSG00000029291 | 10.88166667 | 4.278 | -1.346891153 | 1.18E-16 | 1.18049E-17 |
| ENSMUSG00000040297 | 27.06233333 | 10.62633333 | -1.348642361 | 9.11722E-46 | 3.17882E-47 |
| ENSMUSG00000018841 | 22.404 | 8.784666667 | -1.350696884 | 7.88154E-26 | 5.02799E-27 |
| ENSMUSG00000039616 | 5.648666667 | 2.214666667 | -1.350820796 | 1.52193E-11 | 2.15197E-12 |
| ENSMUSG00000019158 | 28.91866667 | 11.323 | -1.352744789 | 1.57141E-16 | 1.58399E-17 |
| ENSMUSG00000040616 | 8.194333333 | 3.208 | -1.352952439 | 2.02905E-10 | 3.17022E-11 |
| ENSMUSG00000028757 | 165.6353333 | 64.691 | -1.356373542 | 3.85775E-75 | 8.07028E-77 |
| ENSMUSG00000020629 | 17.16133333 | 6.692666667 | -1.358508579 | 4.29913E-13 | 5.3754E-14 |
| ENSMUSG00000066440 | 10.22133333 | 3.978 | -1.361468221 | 7.97893E-32 | 4.18668E-33 |
| ENSMUSG00000037098 | 5.038 | 1.956333333 | -1.364698915 | 2.87136E-15 | 3.12828E-16 |
| ENSMUSG00000037235 | 23.866 | 9.265666667 | -1.3649901 | 3.74634E-37 | 1.6528E-38 |
| ENSMUSG00000072915 | 1.407 | 0.545666667 | -1.366530507 | 9.0587E-05 | 2.55925E-05 |
| ENSMUSG00000067629 | 10.83066667 | 4.196 | -1.368035466 | 2.7206E-24 | 1.88211E-25 |
| ENSMUSG00000068129 | 74.93433333 | 29.00266667 | -1.369441327 | 7.48089E-32 | 3.91502E-33 |
| ENSMUSG00000111375 | 2.737333333 | 1.058 | -1.371431499 | 1.22992E-10 | 1.87739E-11 |
| ENSMUSG00000015533 | 0.422 | 0.163 | -1.372371034 | 0.005570903 | 0.002107358 |
| ENSMUSG00000032479 | 44.406 | 17.15033333 | -1.372518005 | 2.08102E-41 | 8.17523E-43 |
| ENSMUSG00000031727 | 1.296 | 0.499666667 | -1.375027836 | 0.002554047 | 0.000909011 |
| ENSMUSG00000022964 | 14.95466667 | 5.754666667 | -1.377791485 | 2.50443E-21 | 1.96599E-22 |
| ENSMUSG00000051457 | 69.25266667 | 26.60533333 | -1.380154146 | 2.16177E-50 | 6.76112E-52 |
| ENSMUSG00000030930 | 2.228333333 | 0.856 | -1.380282358 | 2.50952E-08 | 4.68499E-09 |
| ENSMUSG00000049550 | 11.67366667 | 4.481666667 | -1.381150524 | 6.25124E-20 | 5.28705E-21 |
| ENSMUSG00000026360 | 11.128 | 4.268666667 | -1.382336911 | 2.09343E-24 | 1.43782E-25 |
| ENSMUSG00000044199 | 4.354666667 | 1.668666667 | -1.3838665 | 1.69902E-07 | 3.42291E-08 |
| ENSMUSG00000001751 | 20.132 | 7.697 | -1.387122351 | 1.32035E-17 | 1.25527E-18 |
| ENSMUSG00000018931 | 9.695 | 3.705666667 | -1.387507785 | 1.2879E-21 | 9.96779E-23 |
| ENSMUSG00000037890 | 3.376666667 | 1.290333333 | -1.387855961 | 2.45212E-08 | 4.56936E-09 |
| ENSMUSG00000030060 | 24.48433333 | 9.348666667 | -1.389026391 | 2.3395E-21 | 1.83167E-22 |

| | | | | | |
|---|---|---|---|---|---|
| ENSMUSG00000023952 | 51.89266667 | 19.79366667 | -1.390491787 | 1.93903E-42 | 7.44338E-44 |
| ENSMUSG00000034584 | 0.257 | 0.098 | -1.390914705 | 0.049740546 | 0.023307725 |
| ENSMUSG00000027394 | 0.719666667 | 0.273666667 | -1.394909116 | 0.007633095 | 0.002966494 |
| ENSMUSG00000023883 | 113.839 | 43.274 | -1.395422507 | 3.65723E-36 | 1.67661E-37 |
| ENSMUSG00000048485 | 7.923666667 | 3.008666667 | -1.397043912 | 5.53801E-09 | 9.71178E-10 |
| ENSMUSG00000073633 | 2.477666667 | 0.937666667 | -1.401835055 | 0.017742478 | 0.007452723 |
| ENSMUSG00000020743 | 17.09366667 | 6.466 | -1.402516482 | 5.19277E-16 | 5.40645E-17 |
| ENSMUSG00000054920 | 55.418 | 20.962 | -1.402578274 | 2.11437E-43 | 7.8975E-45 |
| ENSMUSG00000034570 | 5.739666667 | 2.169 | -1.403936901 | 1.60272E-10 | 2.48215E-11 |
| ENSMUSG00000036181 | 31.55333333 | 11.923 | -1.404045137 | 3.10534E-18 | 2.84292E-19 |
| ENSMUSG00000048410 | 6.109333333 | 2.305666667 | -1.405831002 | 1.74434E-15 | 1.87874E-16 |
| ENSMUSG00000031974 | 27.85333333 | 10.51066667 | -1.405995813 | 7.90257E-49 | 2.5589E-50 |
| ENSMUSG00000031066 | 35.04466667 | 13.219 | -1.406581859 | 3.57896E-55 | 1.00816E-56 |
| ENSMUSG00000020986 | 23.57066667 | 8.886333333 | -1.4073324 | 8.05607E-37 | 3.63202E-38 |
| ENSMUSG00000001911 | 10.70966667 | 4.034666667 | -1.408392186 | 3.66982E-13 | 4.56573E-14 |
| ENSMUSG00000019528 | 81.353 | 30.647 | -1.408449691 | 5.62478E-50 | 1.77862E-51 |
| ENSMUSG00000020432 | 42.516 | 16.00866667 | -1.409152718 | 7.27044E-32 | 3.79484E-33 |
| ENSMUSG00000034613 | 7.607333333 | 2.864 | -1.409359329 | 3.75173E-28 | 2.19913E-29 |
| ENSMUSG00000029925 | 2.126333333 | 0.799333333 | -1.41149862 | 0.003254469 | 0.001180786 |
| ENSMUSG00000118669 | 1.176666667 | 0.442 | -1.412587408 | 0.001607762 | 0.000550906 |
| ENSMUSG00000041372 | 0.497666667 | 0.186666667 | -1.414715433 | 0.030772801 | 0.013652722 |
| ENSMUSG00000013611 | 3.058333333 | 1.144333333 | -1.418238301 | 1.68098E-05 | 4.30343E-06 |
| ENSMUSG00000025283 | 71.308 | 26.66866667 | -1.418918243 | 5.1527E-25 | 3.42232E-26 |
| ENSMUSG00000068758 | 25.92333333 | 9.693666667 | -1.419136859 | 3.24279E-11 | 4.69493E-12 |
| ENSMUSG00000017167 | 2.687333333 | 1.004 | -1.420416013 | 1.25506E-09 | 2.09217E-10 |
| ENSMUSG00000031767 | 14.97333333 | 5.58 | -1.4240584 | 3.37468E-15 | 3.69994E-16 |
| ENSMUSG00000093769 | 0.949 | 0.353 | -1.426739904 | 0.029712041 | 0.013130408 |
| ENSMUSG00000028680 | 16.98 | 6.307333333 | -1.428734374 | 0.009042959 | 0.003571856 |
| ENSMUSG00000024975 | 41.651 | 15.45833333 | -1.429966353 | 5.85511E-43 | 2.21931E-44 |
| ENSMUSG00000037447 | 14.13166667 | 5.242333333 | -1.43065063 | 1.57548E-13 | 1.9155E-14 |
| ENSMUSG00000079334 | 14.39866667 | 5.334 | -1.432645493 | 8.62014E-14 | 1.02842E-14 |
| ENSMUSG00000020818 | 13.62933333 | 5.045666667 | -1.43359819 | 5.62871E-18 | 5.22688E-19 |

| | | | | | |
|---|---|---|---|---|---|
| ENSMUSG00000022951 | 15.24666667 | 5.637 | -1.435494392 | 1.39208E-12 | 1.80594E-13 |
| ENSMUSG00000021185 | 21.00633333 | 7.766 | -1.435580748 | 6.25411E-42 | 2.43532E-43 |
| ENSMUSG00000029752 | 275.9253333 | 102.002 | -1.43568048 | 2.0937E-99 | 3.3392E-101 |
| ENSMUSG00000042524 | 80.48966667 | 29.71133333 | -1.437790231 | 3.22548E-37 | 1.42078E-38 |
| ENSMUSG00000052331 | 54.349 | 20.052 | -1.438507351 | 1.76762E-44 | 6.40709E-46 |
| ENSMUSG00000038290 | 23.924 | 8.824 | -1.438953927 | 3.11084E-49 | 1.00301E-50 |
| ENSMUSG00000035845 | 20.398 | 7.516666667 | -1.440262772 | 8.61163E-24 | 6.12398E-25 |
| ENSMUSG00000021676 | 38.64866667 | 14.23133333 | -1.441347809 | 1.95407E-58 | 5.14017E-60 |
| ENSMUSG00000040913 | 13.396 | 4.929333333 | -1.442337833 | 4.44058E-11 | 6.518E-12 |
| ENSMUSG00000039577 | 1.627333333 | 0.598666667 | -1.442684945 | 0.000122356 | 3.52437E-05 |
| ENSMUSG00000033326 | 50.09833333 | 18.41866667 | -1.443593981 | 1.30666E-47 | 4.3844E-49 |
| ENSMUSG00000020189 | 45.37566667 | 16.67966667 | -1.443828382 | 4.72246E-86 | 8.60763E-88 |
| ENSMUSG00000030203 | 8.735 | 3.209 | -1.444683914 | 9.22283E-35 | 4.38727E-36 |
| ENSMUSG00000021068 | 64.228 | 23.55833333 | -1.446964896 | 4.06232E-55 | 1.14993E-56 |
| ENSMUSG00000003500 | 44.34733333 | 16.24933333 | -1.448466826 | 2.38369E-19 | 2.07199E-20 |
| ENSMUSG00000033985 | 5.094666667 | 1.865666667 | -1.44929651 | 6.62592E-11 | 9.86752E-12 |
| ENSMUSG00000032425 | 16.66366667 | 6.088 | -1.452665622 | 3.56751E-16 | 3.67982E-17 |
| ENSMUSG00000042349 | 8.681 | 3.164666667 | -1.455807693 | 2.14831E-18 | 1.94897E-19 |
| ENSMUSG00000028188 | 2.716333333 | 0.99 | -1.4561601 | 0.025594117 | 0.011153691 |
| ENSMUSG00000051185 | 10.485 | 3.814333333 | -1.458824031 | 2.20937E-16 | 2.2469E-17 |
| ENSMUSG00000051435 | 1.747 | 0.634333333 | -1.461566547 | 0.000599617 | 0.000190682 |
| ENSMUSG00000031133 | 40.71566667 | 14.77733333 | -1.462198076 | 6.85529E-74 | 1.46723E-75 |
| ENSMUSG00000021127 | 18.00266667 | 6.523 | -1.46460309 | 8.30865E-31 | 4.48016E-32 |
| ENSMUSG00000042350 | 40.66366667 | 14.728 | -1.465178778 | 2.86104E-74 | 6.04445E-76 |
| ENSMUSG00000026239 | 69.83033333 | 25.271 | -1.466371105 | 4.76403E-41 | 1.89456E-42 |
| ENSMUSG00000028849 | 23.28533333 | 8.416 | -1.468214925 | 6.10596E-28 | 3.61282E-29 |
| ENSMUSG00000036599 | 26.66533333 | 9.628666667 | -1.469557423 | 1.37756E-22 | 1.02052E-23 |
| ENSMUSG00000033857 | 6.000666667 | 2.163333333 | -1.471866814 | 1.66261E-09 | 2.79052E-10 |
| ENSMUSG00000043257 | 7.891666667 | 2.838666667 | -1.475116571 | 2.08026E-13 | 2.55221E-14 |
| ENSMUSG00000038893 | 27.78766667 | 9.984 | -1.476754857 | 1.82849E-34 | 8.84958E-36 |
| ENSMUSG00000036537 | 4.579666667 | 1.643333333 | -1.478617449 | 6.60813E-05 | 1.83681E-05 |
| ENSMUSG00000037287 | 28.955 | 10.38133333 | -1.479820748 | 4.44078E-48 | 1.46554E-49 |

| | | | | | |
|---|---|---|---|---|---|
| ENSMUSG00000029499 | 3.303666667 | 1.184 | -1.480399048 | 0.002572633 | 0.000915804 |
| ENSMUSG00000041598 | 5.452666667 | 1.953 | -1.481270014 | 3.5588E-08 | 6.72005E-09 |
| ENSMUSG00000033557 | 24.13333333 | 8.64 | -1.481923979 | 3.58163E-45 | 1.26855E-46 |
| ENSMUSG00000117694 | 6.102 | 2.176666667 | -1.487161689 | 0.00502831 | 0.001889956 |
| ENSMUSG00000041096 | 20.99566667 | 7.488 | -1.487439258 | 3.87079E-23 | 2.80608E-24 |
| ENSMUSG00000027175 | 2.062333333 | 0.735 | -1.488461378 | 2.20109E-10 | 3.44966E-11 |
| ENSMUSG00000030200 | 3.640333333 | 1.295666667 | -1.490375952 | 3.80313E-05 | 1.02142E-05 |
| ENSMUSG00000042675 | 19.65033333 | 6.967 | -1.495944317 | 4.1645E-11 | 6.09551E-12 |
| ENSMUSG00000024900 | 41.00533333 | 14.52866667 | -1.496909256 | 5.80126E-42 | 2.25498E-43 |
| ENSMUSG00000008167 | 8.475666667 | 3.002333333 | -1.497242689 | 3.91541E-13 | 4.8821E-14 |
| ENSMUSG00000023473 | 0.452 | 0.16 | -1.498250868 | 0.044976109 | 0.020808127 |
| ENSMUSG00000027438 | 1.751333333 | 0.619666667 | -1.49888943 | 6.69143E-06 | 1.62943E-06 |
| ENSMUSG00000078624 | 0.910333333 | 0.322 | -1.49933422 | 0.000310234 | 9.42653E-05 |
| ENSMUSG00000044952 | 1.222333333 | 0.432333333 | -1.499421786 | 0.00167715 | 0.00057584 |
| ENSMUSG00000049184 | 6.167666667 | 2.181 | -1.499735027 | 1.50459E-05 | 3.83626E-06 |
| ENSMUSG00000027907 | 75.02133333 | 26.523 | -1.500056937 | 5.13581E-28 | 3.02461E-29 |
| ENSMUSG00000045409 | 15.95533333 | 5.638 | -1.500783366 | 3.76576E-22 | 2.84694E-23 |
| ENSMUSG00000038732 | 29.14966667 | 10.297 | -1.501255312 | 2.16207E-49 | 6.92627E-51 |
| ENSMUSG00000094081 | 0.941333333 | 0.332333333 | -1.502074679 | 0.001299454 | 0.00043665 |
| ENSMUSG00000052605 | 3.709333333 | 1.309333333 | -1.50232749 | 0.0002277 | 6.81026E-05 |
| ENSMUSG00000030433 | 3.159666667 | 1.114666667 | -1.503160021 | 0.000164844 | 4.82332E-05 |
| ENSMUSG00000039234 | 39.222 | 13.82366667 | -1.504522768 | 5.67647E-46 | 1.97132E-47 |
| ENSMUSG00000047604 | 41.362 | 14.55933333 | -1.50636165 | 1.02383E-38 | 4.31898E-40 |
| ENSMUSG00000028689 | 20.88866667 | 7.347 | -1.507493228 | 5.75343E-13 | 7.24541E-14 |
| ENSMUSG00000046805 | 1.781 | 0.626 | -1.508452954 | 4.01976E-06 | 9.55263E-07 |
| ENSMUSG00000021572 | 5.137666667 | 1.803 | -1.510713894 | 0.000132682 | 3.8392E-05 |
| ENSMUSG00000033327 | 0.957666667 | 0.336 | -1.511062354 | 6.67045E-08 | 1.29412E-08 |
| ENSMUSG00000039477 | 24.165 | 8.476 | -1.511463502 | 3.01285E-37 | 1.32296E-38 |
| ENSMUSG00000057060 | 3.841333333 | 1.345666667 | -1.513286074 | 4.97752E-07 | 1.05434E-07 |
| ENSMUSG00000038417 | 3.170666667 | 1.110666667 | -1.513360314 | 2.56913E-06 | 5.94036E-07 |
| ENSMUSG00000029512 | 11.292 | 3.954 | -1.513916258 | 4.01821E-25 | 2.65495E-26 |
| ENSMUSG00000036820 | 13.613 | 4.766333333 | -1.514033285 | 1.05706E-15 | 1.11808E-16 |

| | | | | | |
|---|---|---|---|---|---|
| ENSMUSG00000027778 | 16.67066667 | 5.836333333 | -1.514177611 | 5.25977E-28 | 3.10125E-29 |
| ENSMUSG00000005917 | 4.231333333 | 1.478666667 | -1.516815477 | 2.0423E-06 | 4.66581E-07 |
| ENSMUSG00000047515 | 2.983 | 1.040666667 | -1.519255941 | 0.000414503 | 0.000128724 |
| ENSMUSG00000075254 | 5.218333333 | 1.819333333 | -1.520179209 | 1.67167E-30 | 9.0947E-32 |
| ENSMUSG00000028696 | 12.27566667 | 4.27 | -1.523493402 | 3.34043E-21 | 2.65223E-22 |
| ENSMUSG00000038608 | 14.826 | 5.154666667 | -1.524178375 | 6.21499E-27 | 3.84897E-28 |
| ENSMUSG00000002996 | 51.70533333 | 17.96066667 | -1.525472199 | 1.9368E-32 | 9.88191E-34 |
| ENSMUSG00000066687 | 25.59966667 | 8.866333333 | -1.529715518 | 5.60949E-24 | 3.93485E-25 |
| ENSMUSG00000039652 | 2.214 | 0.766 | -1.531238925 | 1.22037E-08 | 2.2092E-09 |
| ENSMUSG00000110277 | 6.686666667 | 2.313 | -1.531521934 | 8.22059E-07 | 1.78726E-07 |
| ENSMUSG00000079554 | 0.286333333 | 0.099 | -1.5321952 | 0.027027776 | 0.011838181 |
| ENSMUSG00000040613 | 3.668 | 1.263333333 | -1.537758291 | 1.3676E-07 | 2.72877E-08 |
| ENSMUSG00000038910 | 26.478 | 9.106 | -1.539904789 | 8.62498E-57 | 2.34025E-58 |
| ENSMUSG00000058818 | 0.575 | 0.197333333 | -1.542927281 | 0.013141467 | 0.005352217 |
| ENSMUSG00000089901 | 4.307333333 | 1.478 | -1.543148705 | 4.59315E-11 | 6.75464E-12 |
| ENSMUSG00000000078 | 47.12533333 | 16.15066667 | -1.544909105 | 5.59029E-68 | 1.26596E-69 |
| ENSMUSG00000038593 | 1.751333333 | 0.6 | -1.545419294 | 4.17395E-08 | 7.93062E-09 |
| ENSMUSG00000028439 | 7.578666667 | 2.594666667 | -1.546394844 | 2.98887E-10 | 4.7524E-11 |
| ENSMUSG00000045679 | 31.00366667 | 10.61066667 | -1.546923543 | 9.87998E-26 | 6.33017E-27 |
| ENSMUSG00000029254 | 2.818333333 | 0.961333333 | -1.55173359 | 3.45008E-07 | 7.16503E-08 |
| ENSMUSG00000040274 | 92.73233333 | 31.59 | -1.553604519 | 1.7819E-84 | 3.29708E-86 |
| ENSMUSG00000019986 | 10.952 | 3.73 | -1.553946816 | 5.92254E-30 | 3.2794E-31 |
| ENSMUSG00000032051 | 31.557 | 10.74266667 | -1.554607901 | 1.88848E-19 | 1.63632E-20 |
| ENSMUSG00000031751 | 85.87866667 | 29.205 | -1.556084408 | 1.26942E-39 | 5.18848E-41 |
| ENSMUSG00000051166 | 29.282 | 9.958 | -1.556086174 | 0.006200695 | 0.002368713 |
| ENSMUSG00000023467 | 14.56966667 | 4.942 | -1.559800955 | 1.44551E-14 | 1.63773E-15 |
| ENSMUSG00000003051 | 1.754333333 | 0.595 | -1.559961321 | 0.001160823 | 0.000386781 |
| ENSMUSG00000026484 | 72.43633333 | 24.55033333 | -1.560970907 | 4.05869E-82 | 7.70603E-84 |
| ENSMUSG00000029070 | 4.952 | 1.678 | -1.561268599 | 1.78098E-08 | 3.27325E-09 |
| ENSMUSG00000020462 | 28.17266667 | 9.529 | -1.563899401 | 4.98689E-35 | 2.35503E-36 |
| ENSMUSG00000053040 | 2.757 | 0.930333333 | -1.567279644 | 0.01048616 | 0.004184618 |
| ENSMUSG00000031953 | 5.277 | 1.779333333 | -1.568381179 | 4.27194E-18 | 3.95222E-19 |

| | | | | | |
|---|---|---|---|---|---|
| ENSMUSG00000046962 | 19.47366667 | 6.563 | -1.569097214 | 1.08698E-16 | 1.08593E-17 |
| ENSMUSG00000001014 | 5.498666667 | 1.853 | -1.569218951 | 9.23964E-05 | 2.61484E-05 |
| ENSMUSG00000026411 | 37.788 | 12.718 | -1.571056349 | 3.87364E-45 | 1.37733E-46 |
| ENSMUSG00000029312 | 1.828666667 | 0.614666667 | -1.572915966 | 1.38015E-06 | 3.10924E-07 |
| ENSMUSG00000028223 | 55.82333333 | 18.701 | -1.577752856 | 4.55932E-57 | 1.2308E-58 |
| ENSMUSG00000028669 | 62.02833333 | 20.73766667 | -1.580673785 | 2.2605E-25 | 1.47173E-26 |
| ENSMUSG00000036452 | 23.197 | 7.753666667 | -1.580987618 | 2.63871E-40 | 1.06394E-41 |
| ENSMUSG00000019842 | 5.368333333 | 1.793333333 | -1.581830583 | 1.88372E-12 | 2.46585E-13 |
| ENSMUSG00000035868 | 2.427 | 0.810333333 | -1.582586716 | 0.000148236 | 4.31589E-05 |
| ENSMUSG00000020227 | 63.12733333 | 21.054 | -1.584170456 | 4.77864E-37 | 2.12142E-38 |
| ENSMUSG00000024220 | 16.226 | 5.411333333 | -1.584251376 | 1.01734E-16 | 1.01214E-17 |
| ENSMUSG00000030546 | 1.140666667 | 0.380333333 | -1.584540968 | 0.004248736 | 0.001575822 |
| ENSMUSG00000059970 | 18.17466667 | 6.050333333 | -1.586842372 | 2.65538E-37 | 1.16416E-38 |
| ENSMUSG00000056043 | 0.285666667 | 0.095 | -1.588333285 | 0.011239951 | 0.004511811 |
| ENSMUSG00000021959 | 4.064333333 | 1.348 | -1.592198232 | 1.72921E-08 | 3.17451E-09 |
| ENSMUSG00000074220 | 5.555 | 1.841666667 | -1.592774948 | 1.85733E-05 | 4.78437E-06 |
| ENSMUSG00000016526 | 8.792 | 2.913666667 | -1.593355549 | 1.5104E-14 | 1.71433E-15 |
| ENSMUSG00000016427 | 21.251 | 7.042333333 | -1.59340531 | 2.47719E-08 | 4.61951E-09 |
| ENSMUSG00000090353 | 4.050333333 | 1.341666667 | -1.594014361 | 5.02926E-05 | 1.37642E-05 |
| ENSMUSG00000044566 | 1.796333333 | 0.595 | -1.594093512 | 8.18722E-06 | 2.01515E-06 |
| ENSMUSG00000001755 | 29.81866667 | 9.875666667 | -1.594265702 | 4.68952E-37 | 2.07862E-38 |
| ENSMUSG00000079427 | 8.659333333 | 2.862 | -1.597232287 | 0.00022503 | 6.72729E-05 |
| ENSMUSG00000072707 | 1.644333333 | 0.542333333 | -1.600251036 | 1.02826E-05 | 2.56284E-06 |
| ENSMUSG00000006464 | 2.047333333 | 0.675 | -1.600786604 | 1.31953E-10 | 2.02613E-11 |
| ENSMUSG00000056185 | 5.100333333 | 1.679666667 | -1.602416582 | 3.83561E-08 | 7.26924E-09 |
| ENSMUSG00000044072 | 0.315333333 | 0.103333333 | -1.609571968 | 0.003883975 | 0.00143115 |
| ENSMUSG00000046179 | 80.21266667 | 26.219 | -1.613217414 | 2.52534E-63 | 6.24186E-65 |
| ENSMUSG00000024905 | 0.893 | 0.291666667 | -1.614339659 | 0.046064296 | 0.021391084 |
| ENSMUSG00000021240 | 7.588666667 | 2.472666667 | -1.617778658 | 3.92189E-16 | 4.05349E-17 |
| ENSMUSG00000039474 | 3.378 | 1.100333333 | -1.61822869 | 4.88354E-11 | 7.20527E-12 |
| ENSMUSG00000074527 | 18.92033333 | 6.161 | -1.618701065 | 0.000170409 | 4.99908E-05 |
| ENSMUSG00000026456 | 61.987 | 20.18366667 | -1.618777398 | 2.09151E-45 | 7.35003E-47 |

| | | | | | |
|---|---|---|---|---|---|
| ENSMUSG00000041538 | 3.785 | 1.231666667 | -1.619681437 | 2.92928E-08 | 5.50303E-09 |
| ENSMUSG00000020732 | 26.03366667 | 8.471 | -1.619774325 | 2.13158E-28 | 1.23474E-29 |
| ENSMUSG00000026024 | 18.29766667 | 5.947 | -1.621425706 | 1.01397E-55 | 2.81426E-57 |
| ENSMUSG00000020590 | 13.464 | 4.374333333 | -1.621972016 | 2.42393E-46 | 8.36761E-48 |
| ENSMUSG00000030104 | 73.333 | 23.81933333 | -1.622329525 | 5.38414E-74 | 1.14121E-75 |
| ENSMUSG00000032750 | 3.84 | 1.247 | -1.622644846 | 1.02053E-06 | 2.25258E-07 |
| ENSMUSG00000034614 | 5.628 | 1.827333333 | -1.622882501 | 2.17807E-13 | 2.68123E-14 |
| ENSMUSG00000003812 | 24.904 | 8.065666667 | -1.626511793 | 3.08223E-26 | 1.93437E-27 |
| ENSMUSG00000035329 | 23.697 | 7.671666667 | -1.627092486 | 1.96627E-45 | 6.89633E-47 |
| ENSMUSG00000029217 | 32.067 | 10.37166667 | -1.628441645 | 5.24448E-36 | 2.41513E-37 |
| ENSMUSG00000056832 | 1.207 | 0.390333333 | -1.628647101 | 5.27224E-06 | 1.26928E-06 |
| ENSMUSG00000025351 | 382.136 | 123.2726667 | -1.632233231 | 5.0545E-88 | 9.00345E-90 |
| ENSMUSG00000031174 | 5.697666667 | 1.836333333 | -1.633543259 | 3.56214E-11 | 5.17942E-12 |
| ENSMUSG00000042320 | 1.560666667 | 0.501333333 | -1.638320367 | 3.44662E-05 | 9.18528E-06 |
| ENSMUSG00000020798 | 32.31666667 | 10.38033333 | -1.638425625 | 1.73579E-36 | 7.8856E-38 |
| ENSMUSG00000036478 | 12.15066667 | 3.901666667 | -1.638873038 | 3.93039E-44 | 1.43007E-45 |
| ENSMUSG00000037108 | 14.68766667 | 4.709666667 | -1.640908363 | 6.33523E-22 | 4.82009E-23 |
| ENSMUSG00000024873 | 3.272333333 | 1.048333333 | -1.642222198 | 3.8006E-05 | 1.02047E-05 |
| ENSMUSG00000067049 | 0.696666667 | 0.222666667 | -1.645582934 | 0.01046601 | 0.004173686 |
| ENSMUSG00000052040 | 28.68933333 | 9.166333333 | -1.646097789 | 1.92267E-26 | 1.20134E-27 |
| ENSMUSG00000033540 | 5.064666667 | 1.618 | -1.646255713 | 4.29228E-16 | 4.44816E-17 |
| ENSMUSG00000026604 | 1.833 | 0.585 | -1.647698256 | 3.33261E-17 | 3.25346E-18 |
| ENSMUSG00000020964 | 58.55466667 | 18.66 | -1.649835168 | 6.2309E-105 | 9.5503E-107 |
| ENSMUSG00000003363 | 23.238 | 7.403333333 | -1.650239015 | 1.37277E-43 | 5.08014E-45 |
| ENSMUSG00000028756 | 13.79266667 | 4.394 | -1.650294639 | 1.73888E-25 | 1.12492E-26 |
| ENSMUSG00000031007 | 103.6226667 | 32.99433333 | -1.651049443 | 5.71832E-79 | 1.13703E-80 |
| ENSMUSG00000026003 | 90.53533333 | 28.78966667 | -1.652929859 | 5.54517E-83 | 1.03752E-84 |
| ENSMUSG00000053574 | 0.372333333 | 0.118333333 | -1.653738256 | 0.040573277 | 0.01853866 |
| ENSMUSG00000006463 | 17.68033333 | 5.604 | -1.657616614 | 1.13952E-30 | 6.16806E-32 |
| ENSMUSG00000034793 | 36.70333333 | 11.57433333 | -1.664981994 | 3.43014E-26 | 2.15508E-27 |
| ENSMUSG00000032204 | 14.40233333 | 4.538666667 | -1.665962121 | 5.62982E-18 | 5.2318E-19 |
| ENSMUSG00000021614 | 0.694 | 0.218666667 | -1.666202349 | 8.0675E-11 | 1.20923E-11 |

| | | | | | |
|---|---|---|---|---|---|
| ENSMUSG00000032115 | 211.0846667 | 66.49533333 | -1.666496784 | 6.52587E-83 | 1.22552E-84 |
| ENSMUSG00000041143 | 5.721666667 | 1.797333333 | -1.670577456 | 4.25812E-10 | 6.86755E-11 |
| ENSMUSG00000048347 | 0.239 | 0.075 | -1.672048117 | 0.040151223 | 0.018323639 |
| ENSMUSG00000038777 | 1.974333333 | 0.617333333 | -1.677243988 | 0.000102745 | 2.93041E-05 |
| ENSMUSG00000009145 | 3.259 | 1.018666667 | -1.677747309 | 2.79961E-08 | 5.24783E-09 |
| ENSMUSG00000026200 | 1.355666667 | 0.421666667 | -1.684827606 | 0.000401286 | 0.000124314 |
| ENSMUSG00000025978 | 2.676666667 | 0.832333333 | -1.685204166 | 4.26332E-09 | 7.39694E-10 |
| ENSMUSG00000043987 | 11.83966667 | 3.680333333 | -1.68572012 | 2.66034E-27 | 1.6145E-28 |
| ENSMUSG00000018740 | 2.176333333 | 0.676333333 | -1.686093176 | 0.000260329 | 7.84006E-05 |
| ENSMUSG00000030708 | 1.183666667 | 0.366666667 | -1.690721837 | 0.027684069 | 0.012154308 |
| ENSMUSG00000033107 | 53.243 | 16.48266667 | -1.691642193 | 2.74657E-49 | 8.81771E-51 |
| ENSMUSG00000020279 | 3.700333333 | 1.144 | -1.693568185 | 2.68996E-12 | 3.57139E-13 |
| ENSMUSG00000021027 | 43.79333333 | 13.50733333 | -1.696968384 | 5.1416E-117 | 6.7447E-119 |
| ENSMUSG00000045114 | 0.945666667 | 0.291333333 | -1.698660966 | 0.037318568 | 0.016896932 |
| ENSMUSG00000022253 | 57.61266667 | 17.72433333 | -1.700654672 | 5.06383E-55 | 1.44391E-56 |
| ENSMUSG00000025026 | 167.3543333 | 51.417 | -1.702588566 | 9.56604E-95 | 1.5983E-96 |
| ENSMUSG00000041827 | 1.270333333 | 0.39 | -1.703661078 | 0.000818867 | 0.000265945 |
| ENSMUSG00000028494 | 50.76166667 | 15.58366667 | -1.703704714 | 3.49605E-42 | 1.34928E-43 |
| ENSMUSG00000036411 | 0.815666667 | 0.25 | -1.706051601 | 0.006347119 | 0.002430784 |
| ENSMUSG00000006850 | 19.55866667 | 5.974333333 | -1.710958385 | 4.83764E-30 | 2.66865E-31 |
| ENSMUSG00000021298 | 1.003333333 | 0.306 | -1.713197428 | 0.003211461 | 0.001164055 |
| ENSMUSG00000030000 | 0.596666667 | 0.181666667 | -1.715631452 | 0.008733072 | 0.003439205 |
| ENSMUSG00000039178 | 16.348 | 4.967333333 | -1.718570681 | 2.86587E-28 | 1.66998E-29 |
| ENSMUSG00000045362 | 2.657333333 | 0.805333333 | -1.722321256 | 5.43718E-07 | 1.15771E-07 |
| ENSMUSG00000075078 | 2.511333333 | 0.757333333 | -1.729453198 | 2.98476E-06 | 6.96938E-07 |
| ENSMUSG00000022637 | 44.24366667 | 13.327 | -1.731118897 | 1.17339E-31 | 6.18127E-33 |
| ENSMUSG00000031162 | 61.649 | 18.55666667 | -1.732139911 | 3.48709E-33 | 1.74547E-34 |
| ENSMUSG00000054277 | 33.093 | 9.947666667 | -1.734096012 | 2.27566E-33 | 1.13594E-34 |
| ENSMUSG00000057069 | 20.99733333 | 6.297333333 | -1.737393177 | 9.4833E-58 | 2.52076E-59 |
| ENSMUSG00000026564 | 0.332333333 | 0.099666667 | -1.73744802 | 0.045343993 | 0.021019026 |
| ENSMUSG00000020576 | 19.43066667 | 5.810333333 | -1.741642564 | 1.41292E-89 | 2.47777E-91 |
| ENSMUSG00000023979 | 0.797333333 | 0.238333333 | -1.742202243 | 0.042232938 | 0.019381548 |

| | | | | | |
|---|---|---|---|---|---|
| ENSMUSG00000030922 | 3.353333333 | 1.001666667 | -1.743193409 | 0.000916891 | 0.000300313 |
| ENSMUSG00000073987 | 38.153 | 11.38533333 | -1.744619971 | 5.31422E-56 | 1.46761E-57 |
| ENSMUSG00000053226 | 1.708 | 0.509333333 | -1.745625932 | 0.000587751 | 0.000186746 |
| ENSMUSG00000055835 | 12.02633333 | 3.582666667 | -1.747091126 | 7.06569E-13 | 8.95651E-14 |
| ENSMUSG00000021904 | 0.772333333 | 0.229666667 | -1.749682156 | 0.000170269 | 4.99291E-05 |
| ENSMUSG00000050357 | 0.867333333 | 0.257 | -1.754818197 | 8.17879E-05 | 2.29485E-05 |
| ENSMUSG00000053178 | 3.053666667 | 0.902333333 | -1.758810202 | 8.72992E-05 | 2.45853E-05 |
| ENSMUSG00000025939 | 38.59 | 11.37666667 | -1.76214913 | 2.52711E-69 | 5.61813E-71 |
| ENSMUSG00000034480 | 8.569666667 | 2.524333333 | -1.763336661 | 4.80695E-32 | 2.50569E-33 |
| ENSMUSG00000024856 | 66.26633333 | 19.444 | -1.768951055 | 5.85944E-46 | 2.03891E-47 |
| ENSMUSG00000031770 | 182.884 | 53.61533333 | -1.770211305 | 7.6792E-108 | 1.1346E-109 |
| ENSMUSG00000028211 | 14.52666667 | 4.248333333 | -1.773734823 | 3.95123E-55 | 1.11575E-56 |
| ENSMUSG00000049090 | 38.606 | 11.29033333 | -1.773737003 | 7.21951E-08 | 1.40462E-08 |
| ENSMUSG00000006567 | 0.701666667 | 0.204666667 | -1.777509673 | 7.15119E-06 | 1.74682E-06 |
| ENSMUSG00000048721 | 0.952333333 | 0.277666667 | -1.778112636 | 0.001785433 | 0.000616347 |
| ENSMUSG00000020571 | 438.4896667 | 127.7506667 | -1.779212025 | 1.0077E-126 | 1.1827E-128 |
| ENSMUSG00000078866 | 22.92066667 | 6.677666667 | -1.779233022 | 5.0243E-20 | 4.23201E-21 |
| ENSMUSG00000034801 | 38.85566667 | 11.308 | -1.780781227 | 2.6506E-105 | 4.0078E-107 |
| ENSMUSG00000037731 | 2.348666667 | 0.683333333 | -1.781180565 | 2.67718E-06 | 6.21052E-07 |
| ENSMUSG00000022848 | 2.271666667 | 0.660333333 | -1.782484777 | 2.68937E-06 | 6.24439E-07 |
| ENSMUSG00000024667 | 31.54166667 | 9.126 | -1.789204335 | 9.59769E-35 | 4.5921E-36 |
| ENSMUSG00000042203 | 26.002 | 7.514333333 | -1.790905576 | 1.45556E-53 | 4.28105E-55 |
| ENSMUSG00000038527 | 0.931 | 0.268333333 | -1.794754885 | 0.001127416 | 0.000375183 |
| ENSMUSG00000056130 | 6.474666667 | 1.860666667 | -1.798986297 | 1.40434E-24 | 9.56006E-26 |
| ENSMUSG00000024644 | 36.077 | 10.35366667 | -1.800937599 | 3.07144E-06 | 7.18451E-07 |
| ENSMUSG00000023272 | 112.7046667 | 32.33033333 | -1.801586966 | 9.85666E-63 | 2.45668E-64 |
| ENSMUSG00000015016 | 17.41966667 | 4.994666667 | -1.802256714 | 6.56051E-24 | 4.6246E-25 |
| ENSMUSG00000110444 | 4.631 | 1.323 | -1.807510695 | 0.02574386 | 0.011229613 |
| ENSMUSG00000063605 | 3.599 | 1.024666667 | -1.812441438 | 7.14734E-09 | 1.2613E-09 |
| ENSMUSG00000025579 | 89.29266667 | 25.36633333 | -1.815626701 | 1.69635E-68 | 3.81808E-70 |
| ENSMUSG00000028100 | 1.543 | 0.437 | -1.820032877 | 0.037994436 | 0.017222634 |
| ENSMUSG00000051224 | 5.414666667 | 1.527666667 | -1.825542742 | 4.71133E-07 | 9.95352E-08 |

| | | | | | |
|---|---|---|---|---|---|
| ENSMUSG00000054469 | 41.81966667 | 11.77233333 | -1.828781265 | 1.96935E-82 | 3.7255E-84 |
| ENSMUSG00000041679 | 2.448333333 | 0.687 | -1.833417986 | 5.75493E-06 | 1.39026E-06 |
| ENSMUSG00000040969 | 0.239 | 0.067 | -1.834777618 | 0.019885013 | 0.008444678 |
| ENSMUSG00000004562 | 9.2 | 2.578333333 | -1.83519507 | 7.55103E-23 | 5.5418E-24 |
| ENSMUSG00000018427 | 9.545666667 | 2.671333333 | -1.83728595 | 7.60522E-13 | 9.65617E-14 |
| ENSMUSG00000022844 | 11.91233333 | 3.331333333 | -1.838284407 | 5.98162E-25 | 3.9894E-26 |
| ENSMUSG00000053604 | 39.454 | 11.02066667 | -1.839960075 | 7.37288E-54 | 2.15322E-55 |
| ENSMUSG00000022769 | 105.8836667 | 29.56566667 | -1.840485347 | 5.36775E-69 | 1.19703E-70 |
| ENSMUSG00000040022 | 6.516 | 1.813333333 | -1.845342453 | 5.18208E-24 | 3.62789E-25 |
| ENSMUSG00000026107 | 26.04666667 | 7.248333333 | -1.845377547 | 8.68952E-38 | 3.74362E-39 |
| ENSMUSG00000031925 | 16.271 | 4.525666667 | -1.846100687 | 7.32109E-61 | 1.88031E-62 |
| ENSMUSG00000024114 | 3.727333333 | 1.036 | -1.847119841 | 2.07863E-07 | 4.22212E-08 |
| ENSMUSG00000004044 | 9.029666667 | 2.506 | -1.849286316 | 8.39183E-32 | 4.41492E-33 |
| ENSMUSG00000003863 | 1.517333333 | 0.421 | -1.849645919 | 4.42297E-09 | 7.69532E-10 |
| ENSMUSG00000030499 | 1.844333333 | 0.508666667 | -1.858306962 | 1.23583E-05 | 3.11288E-06 |
| ENSMUSG00000054733 | 9.476333333 | 2.604 | -1.863599498 | 1.5872E-15 | 1.70292E-16 |
| ENSMUSG00000024807 | 81.82866667 | 22.42766667 | -1.867326812 | 5.49916E-43 | 2.0806E-44 |
| ENSMUSG00000033276 | 0.623666667 | 0.170333333 | -1.872414362 | 5.34293E-06 | 1.28815E-06 |
| ENSMUSG00000038213 | 5.648666667 | 1.541 | -1.874043506 | 3.99594E-15 | 4.39763E-16 |
| ENSMUSG00000026546 | 1.518 | 0.413333333 | -1.876794171 | 0.03056089 | 0.013546045 |
| ENSMUSG00000051890 | 1.988 | 0.540666667 | -1.878506438 | 1.54051E-06 | 3.48965E-07 |
| ENSMUSG00000026670 | 51.87166667 | 14.102 | -1.879046939 | 2.78846E-95 | 4.62048E-97 |
| ENSMUSG00000033486 | 0.578333333 | 0.157 | -1.881136698 | 0.013040316 | 0.005303818 |
| ENSMUSG00000028167 | 0.837 | 0.227 | -1.882535325 | 0.035075071 | 0.015791531 |
| ENSMUSG00000000594 | 0.319666667 | 0.086666667 | -1.883019192 | 0.013385361 | 0.005459867 |
| ENSMUSG00000001281 | 9.539 | 2.58 | -1.886466967 | 1.51037E-28 | 8.70723E-30 |
| ENSMUSG00000070737 | 10.668 | 2.87 | -1.894167088 | 1.56035E-14 | 1.77431E-15 |
| ENSMUSG00000049791 | 5.770666667 | 1.552 | -1.894609441 | 1.79726E-37 | 7.81742E-39 |
| ENSMUSG00000029060 | 16.74933333 | 4.501333333 | -1.895679365 | 6.33778E-38 | 2.71732E-39 |
| ENSMUSG00000099517 | 1.845 | 0.494 | -1.901037869 | 0.042377443 | 0.019468345 |
| ENSMUSG00000028528 | 67.208 | 17.96033333 | -1.903818846 | 4.4714E-90 | 7.81044E-92 |
| ENSMUSG00000057858 | 102.7256667 | 27.44566667 | -1.904146405 | 1.3218E-78 | 2.66477E-80 |

| | | | | | |
|---|---|---|---|---|---|
| ENSMUSG00000024206 | 9.731666667 | 2.593333333 | -1.907879251 | 2.23147E-34 | 1.08307E-35 |
| ENSMUSG00000052085 | 174.074 | 46.154 | -1.915173144 | 2.202E-128 | 2.5389E-130 |
| ENSMUSG00000024352 | 9.046333333 | 2.398333333 | -1.91530097 | 5.41257E-07 | 1.1521E-07 |
| ENSMUSG00000038742 | 3.173666667 | 0.840666667 | -1.916544834 | 1.69888E-07 | 3.42145E-08 |
| ENSMUSG00000019850 | 5.293666667 | 1.398 | -1.920902993 | 3.66293E-20 | 3.05244E-21 |
| ENSMUSG00000021665 | 79.71533333 | 21.022 | -1.922957323 | 6.82981E-83 | 1.28731E-84 |
| ENSMUSG00000090812 | 0.622 | 0.163666667 | -1.926154057 | 0.010691429 | 0.004277605 |
| ENSMUSG00000044328 | 16.467 | 4.322666667 | -1.92958425 | 1.82937E-27 | 1.10262E-28 |
| ENSMUSG00000036854 | 6.390333333 | 1.676 | -1.930869038 | 1.60849E-11 | 2.27881E-12 |
| ENSMUSG00000031309 | 68.90066667 | 17.973 | -1.938686703 | 1.3435E-166 | 1.1873E-168 |
| ENSMUSG00000011148 | 18.97366667 | 4.927333333 | -1.945119529 | 8.56716E-36 | 3.96299E-37 |
| ENSMUSG00000045414 | 27.838 | 7.225666667 | -1.945852958 | 1.84095E-52 | 5.55438E-54 |
| ENSMUSG00000029484 | 71.441 | 18.519 | -1.947746078 | 2.76667E-59 | 7.20129E-61 |
| ENSMUSG00000029769 | 4.992333333 | 1.292333333 | -1.94973603 | 4.56107E-19 | 4.02133E-20 |
| ENSMUSG00000030156 | 23.31833333 | 6.035666667 | -1.949879639 | 3.29938E-30 | 1.80869E-31 |
| ENSMUSG00000047407 | 46.51066667 | 12.02833333 | -1.951124865 | 8.45146E-25 | 5.67165E-26 |
| ENSMUSG00000049280 | 0.629 | 0.162666667 | -1.95114137 | 0.006265145 | 0.002394632 |
| ENSMUSG00000028521 | 17.75966667 | 4.59 | -1.952038445 | 1.64174E-43 | 6.10948E-45 |
| ENSMUSG00000070733 | 37.118 | 9.581 | -1.953870829 | 3.39477E-41 | 1.34066E-42 |
| ENSMUSG00000114456 | 0.856333333 | 0.221 | -1.954126115 | 0.009977555 | 0.003964432 |
| ENSMUSG00000021007 | 5.243333333 | 1.353 | -1.954322426 | 1.98098E-14 | 2.26902E-15 |
| ENSMUSG00000050199 | 8.13 | 2.091 | -1.95906229 | 1.26808E-44 | 4.5789E-46 |
| ENSMUSG00000033581 | 4.047 | 1.040666667 | -1.959344812 | 1.54659E-11 | 2.18897E-12 |
| ENSMUSG00000024187 | 7.694333333 | 1.978333333 | -1.959510802 | 7.01048E-19 | 6.25832E-20 |
| ENSMUSG00000028040 | 0.920333333 | 0.236333333 | -1.961333355 | 0.010090805 | 0.004016822 |
| ENSMUSG00000039153 | 6.178666667 | 1.584333333 | -1.963419643 | 6.60386E-20 | 5.60353E-21 |
| ENSMUSG00000081534 | 84.07966667 | 21.537 | -1.964939646 | 1.74258E-74 | 3.65744E-76 |
| ENSMUSG00000004864 | 8.727666667 | 2.234333333 | -1.965751569 | 1.3517E-15 | 1.43905E-16 |
| ENSMUSG00000031628 | 341.411 | 86.81433333 | -1.97550438 | 4.5479E-118 | 5.9031E-120 |
| ENSMUSG00000030103 | 16.08066667 | 4.087 | -1.97621307 | 1.01531E-54 | 2.90208E-56 |
| ENSMUSG00000029201 | 61.88766667 | 15.66366667 | -1.982229961 | 4.11773E-95 | 6.85151E-97 |
| ENSMUSG00000021236 | 23.289 | 5.894 | -1.982329728 | 5.4136E-62 | 1.36424E-63 |

| | | | | | |
|---|---|---|---|---|---|
| ENSMUSG00000074227 | 57.72566667 | 14.57533333 | -1.985684052 | 2.72418E-54 | 7.91825E-56 |
| ENSMUSG00000036091 | 2.149 | 0.542333333 | -1.986413732 | 0.000517947 | 0.000162994 |
| ENSMUSG00000025931 | 9.655333333 | 2.436 | -1.986811933 | 4.6419E-48 | 1.53512E-49 |
| ENSMUSG00000026017 | 5.608 | 1.414666667 | -1.987024194 | 1.94822E-15 | 2.10102E-16 |
| ENSMUSG00000062082 | 24.67633333 | 6.208666667 | -1.990772657 | 1.22243E-22 | 9.04756E-24 |
| ENSMUSG00000031586 | 41.49166667 | 10.402 | -1.995960667 | 4.13704E-35 | 1.94799E-36 |
| ENSMUSG00000023045 | 14.65566667 | 3.672666667 | -1.996558728 | 6.50768E-40 | 2.65088E-41 |
| ENSMUSG00000020653 | 5.375333333 | 1.347 | -1.996604371 | 2.07646E-27 | 1.25586E-28 |
| ENSMUSG00000033545 | 31.65566667 | 7.931 | -1.996889091 | 6.21495E-44 | 2.27847E-45 |
| ENSMUSG00000029313 | 60.55966667 | 15.14966667 | -1.999071214 | 6.3426E-110 | 9.0646E-112 |
| ENSMUSG00000052293 | 24.98833333 | 6.194666667 | -2.012156122 | 5.95407E-16 | 6.23195E-17 |
| ENSMUSG00000004328 | 1.962333333 | 0.486333333 | -2.012552744 | 1.37366E-11 | 1.93284E-12 |
| ENSMUSG00000004952 | 4.304 | 1.066333333 | -2.013019586 | 3.20862E-16 | 3.30299E-17 |
| ENSMUSG00000018927 | 2.064333333 | 0.511 | -2.014280749 | 6.01008E-05 | 1.6631E-05 |
| ENSMUSG00000090273 | 0.711333333 | 0.176 | -2.014950341 | 0.036913862 | 0.016703497 |
| ENSMUSG00000024253 | 1.189333333 | 0.292666667 | -2.02282277 | 0.003887438 | 0.001432694 |
| ENSMUSG00000036955 | 35.277 | 8.674 | -2.023958529 | 7.30096E-89 | 1.29546E-90 |
| ENSMUSG00000036257 | 73.48533333 | 17.97533333 | -2.031437812 | 1.3745E-115 | 1.8316E-117 |
| ENSMUSG00000025278 | 64.87566667 | 15.859 | -2.032375655 | 1.9945E-140 | 2.1344E-142 |
| ENSMUSG00000021109 | 138.423 | 33.76733333 | -2.035383521 | 3.0799E-188 | 2.254E-190 |
| ENSMUSG00000028542 | 15.08833333 | 3.680333333 | -2.035525109 | 8.82723E-34 | 4.35145E-35 |
| ENSMUSG00000071076 | 72.292 | 17.596 | -2.038588498 | 3.51742E-72 | 7.64973E-74 |
| ENSMUSG00000051736 | 1.894333333 | 0.461 | -2.038851559 | 0.006136372 | 0.002340329 |
| ENSMUSG00000018570 | 0.750333333 | 0.182333333 | -2.040953319 | 0.012565429 | 0.005089849 |
| ENSMUSG00000053693 | 1.426333333 | 0.346 | -2.043467235 | 4.74936E-09 | 8.28613E-10 |
| ENSMUSG00000030559 | 43.25666667 | 10.47133333 | -2.046477341 | 2.18377E-63 | 5.38253E-65 |
| ENSMUSG00000018654 | 2.697666667 | 0.651 | -2.050982646 | 4.60272E-07 | 9.70974E-08 |
| ENSMUSG00000025537 | 1.192666667 | 0.287333333 | -2.053393613 | 0.027519452 | 0.012074435 |
| ENSMUSG00000021257 | 7.871 | 1.895666667 | -2.053841636 | 2.05899E-39 | 8.48671E-41 |
| ENSMUSG00000007216 | 1.451333333 | 0.348666667 | -2.057458556 | 6.38065E-05 | 1.77005E-05 |
| ENSMUSG00000054855 | 11.25933333 | 2.703333333 | -2.058310089 | 8.44313E-24 | 5.9925E-25 |
| ENSMUSG00000022773 | 1.350666667 | 0.324 | -2.059605955 | 0.021922705 | 0.009415986 |

| | | | | | |
|---|---|---|---|---|---|
| ENSMUSG00000039770 | 25.54733333 | 6.122666667 | -2.060940662 | 2.56744E-45 | 9.058E-47 |
| ENSMUSG00000033318 | 3.272 | 0.784 | -2.061247189 | 1.33107E-06 | 2.99009E-07 |
| ENSMUSG00000026864 | 1782.343 | 426.741 | -2.062342368 | 1.6901E-173 | 1.3769E-175 |
| ENSMUSG00000032965 | 20.895 | 4.999 | -2.063446327 | 5.70485E-47 | 1.94573E-48 |
| ENSMUSG00000036402 | 171.5086667 | 40.818 | -2.07100408 | 4.9358E-135 | 5.4865E-137 |
| ENSMUSG00000064220 | 4.038333333 | 0.953666667 | -2.082203002 | 0.01189139 | 0.004797935 |
| ENSMUSG00000024594 | 50.76466667 | 11.93466667 | -2.088666426 | 6.8118E-108 | 1.0017E-109 |
| ENSMUSG00000029456 | 6.076666667 | 1.427666667 | -2.089620979 | 3.4832E-25 | 2.29905E-26 |
| ENSMUSG00000040488 | 14.11333333 | 3.304333333 | -2.094627634 | 2.33932E-34 | 1.13703E-35 |
| ENSMUSG00000076435 | 11.243 | 2.623666667 | -2.099370701 | 1.47153E-26 | 9.16401E-28 |
| ENSMUSG00000039242 | 74.354 | 17.29633333 | -2.103944125 | 2.9858E-119 | 3.793E-121 |
| ENSMUSG00000045780 | 6.745333333 | 1.552666667 | -2.1191416 | 1.62235E-39 | 6.65339E-41 |
| ENSMUSG00000030731 | 0.628666667 | 0.144666667 | -2.119562728 | 0.006328849 | 0.002422476 |
| ENSMUSG00000051228 | 0.150666667 | 0.034666667 | -2.119739244 | 0.039389726 | 0.017932605 |
| ENSMUSG00000043487 | 0.359333333 | 0.082 | -2.131626957 | 0.013891335 | 0.005695591 |
| ENSMUSG00000054843 | 14.61733333 | 3.333333333 | -2.132642643 | 1.28578E-76 | 2.60992E-78 |
| ENSMUSG00000027173 | 13.21033333 | 3.007 | -2.135270097 | 2.62918E-24 | 1.81705E-25 |
| ENSMUSG00000063954 | 3.784 | 0.86 | -2.137503524 | 0.007828274 | 0.003051456 |
| ENSMUSG00000032489 | 1.425666667 | 0.324 | -2.137570988 | 0.000183502 | 5.41231E-05 |
| ENSMUSG00000067851 | 121.1403333 | 27.524 | -2.137917232 | 4.5715E-223 | 2.525E-225 |
| ENSMUSG00000103567 | 0.358333333 | 0.081333333 | -2.139383607 | 0.011639798 | 0.004685171 |
| ENSMUSG00000024063 | 70.52033333 | 15.92866667 | -2.146413785 | 1.47825E-59 | 3.8375E-61 |
| ENSMUSG00000026576 | 0.294333333 | 0.066333333 | -2.149645007 | 0.017580514 | 0.007378621 |
| ENSMUSG00000033590 | 0.481666667 | 0.108333333 | -2.152557869 | 1.84708E-05 | 4.75542E-06 |
| ENSMUSG00000021271 | 3.682333333 | 0.827 | -2.154660994 | 1.12351E-13 | 1.3497E-14 |
| ENSMUSG00000047446 | 41.31233333 | 9.262333333 | -2.157124963 | 9.56262E-76 | 1.98066E-77 |
| ENSMUSG00000025823 | 218.9806667 | 49.082 | -2.157537561 | 1.7717E-149 | 1.7247E-151 |
| ENSMUSG00000041235 | 0.544 | 0.121666667 | -2.160672688 | 0.006545761 | 0.002515445 |
| ENSMUSG00000051495 | 57.82133333 | 12.91566667 | -2.162479764 | 1.6876E-96 | 2.7614E-98 |
| ENSMUSG00000032174 | 0.279 | 0.062 | -2.169925001 | 0.034231353 | 0.015366767 |
| ENSMUSG00000044968 | 2.474666667 | 0.547333333 | -2.176742583 | 9.73746E-13 | 1.24508E-13 |
| ENSMUSG00000040957 | 0.919666667 | 0.203333333 | -2.177264309 | 1.1779E-05 | 2.95451E-06 |

| | | | | | |
|---|---|---|---|---|---|
| ENSMUSG00000034121 | 10.19866667 | 2.254333333 | -2.177607794 | 2.34506E-34 | 1.14144E-35 |
| ENSMUSG00000056481 | 0.344 | 0.076 | -2.178337241 | 0.04293247 | 0.019752968 |
| ENSMUSG00000027378 | 5.524 | 1.218333333 | -2.180804414 | 1.06636E-17 | 1.00496E-18 |
| ENSMUSG00000021226 | 13.62033333 | 2.993333333 | -2.185937162 | 4.98671E-35 | 2.35151E-36 |
| ENSMUSG00000066760 | 0.685333333 | 0.150333333 | -2.188640926 | 0.001172678 | 0.000390974 |
| ENSMUSG00000022558 | 51.04866667 | 11.186 | -2.190179045 | 2.73E-126 | 3.223E-128 |
| ENSMUSG00000041189 | 7.217333333 | 1.578 | -2.193368682 | 1.18048E-23 | 8.43551E-25 |
| ENSMUSG00000025197 | 8.309666667 | 1.816333333 | -2.193761616 | 4.5615E-22 | 3.45482E-23 |
| ENSMUSG00000015837 | 410.896 | 89.778 | -2.194339424 | 2.4881E-145 | 2.5767E-147 |
| ENSMUSG00000061577 | 0.551666667 | 0.120333333 | -2.196760475 | 0.005612285 | 0.002124175 |
| ENSMUSG00000071637 | 16.21 | 3.533 | -2.197918437 | 9.59055E-69 | 2.15198E-70 |
| ENSMUSG00000055447 | 336.1433333 | 73.14833333 | -2.200179639 | 1.2091E-111 | 1.7113E-113 |
| ENSMUSG00000040867 | 1.056 | 0.228333333 | -2.209396442 | 0.000684713 | 0.00021987 |
| ENSMUSG00000022272 | 0.188333333 | 0.040666667 | -2.21136972 | 0.00987656 | 0.003920212 |
| ENSMUSG00000010154 | 12.74633333 | 2.752 | -2.21152992 | 5.72648E-36 | 2.64104E-37 |
| ENSMUSG00000033022 | 2.250333333 | 0.484666667 | -2.21507395 | 4.41536E-05 | 1.19743E-05 |
| ENSMUSG00000032009 | 3.341 | 0.719 | -2.216216307 | 6.01701E-29 | 3.43556E-30 |
| ENSMUSG00000022780 | 0.333333333 | 0.071666667 | -2.217591435 | 0.003827764 | 0.001408059 |
| ENSMUSG00000031995 | 2.252333333 | 0.482666667 | -2.222321253 | 5.15226E-12 | 7.01126E-13 |
| ENSMUSG00000096370 | 2.69 | 0.575333333 | -2.225136209 | 0.042232938 | 0.019381455 |
| ENSMUSG00000032607 | 1.285333333 | 0.274333333 | -2.228140716 | 7.99012E-05 | 2.23915E-05 |
| ENSMUSG00000032418 | 15.114 | 3.215 | -2.232994885 | 4.1235E-64 | 9.93581E-66 |
| ENSMUSG00000031877 | 183.3753333 | 38.72766667 | -2.243363101 | 2.5505E-204 | 1.6376E-206 |
| ENSMUSG00000034845 | 0.892 | 0.186666667 | -2.256579384 | 0.002115278 | 0.000741603 |
| ENSMUSG00000028420 | 7.873 | 1.638333333 | -2.264684562 | 7.11961E-28 | 4.22733E-29 |
| ENSMUSG00000020191 | 1.787 | 0.371666667 | -2.265458425 | 9.02912E-05 | 2.55027E-05 |
| ENSMUSG00000078851 | 2.543333333 | 0.528 | -2.268110722 | 3.28167E-08 | 6.17609E-09 |
| ENSMUSG00000063160 | 3.770333333 | 0.780666667 | -2.271913502 | 4.07575E-16 | 4.21532E-17 |
| ENSMUSG00000037463 | 0.947666667 | 0.195666667 | -2.275981691 | 0.001093918 | 0.000363204 |
| ENSMUSG00000041849 | 0.789666667 | 0.161666667 | -2.288221546 | 0.001356595 | 0.000457912 |
| ENSMUSG00000032715 | 28.382 | 5.805333333 | -2.289525444 | 4.00463E-45 | 1.42944E-46 |
| ENSMUSG00000052013 | 0.316666667 | 0.064666667 | -2.291870861 | 0.041772576 | 0.019147206 |

| | | | | | |
|---|---|---|---|---|---|
| ENSMUSG00000031391 | 7.743333333 | 1.579666667 | -2.293334588 | 7.43839E-30 | 4.14442E-31 |
| ENSMUSG00000029314 | 1.070333333 | 0.218 | -2.295660124 | 6.59017E-06 | 1.60295E-06 |
| ENSMUSG00000004730 | 15.666 | 3.159 | -2.310097021 | 5.38302E-65 | 1.28964E-66 |
| ENSMUSG00000045102 | 0.797333333 | 0.16 | -2.317111079 | 7.84515E-05 | 2.19582E-05 |
| ENSMUSG00000024620 | 0.178666667 | 0.035666667 | -2.324622204 | 0.027573483 | 0.012100045 |
| ENSMUSG00000046056 | 0.835 | 0.166333333 | -2.327698883 | 0.0263116 | 0.011497246 |
| ENSMUSG00000042616 | 1.654666667 | 0.328666667 | -2.331843564 | 1.08654E-05 | 2.71411E-06 |
| ENSMUSG00000041141 | 0.994666667 | 0.197333333 | -2.333578455 | 1.69249E-07 | 3.40508E-08 |
| ENSMUSG00000039308 | 57.503 | 11.38733333 | -2.336207286 | 9.0743E-126 | 1.0901E-127 |
| ENSMUSG00000022995 | 7.952666667 | 1.552333333 | -2.357000321 | 2.07486E-47 | 7.00501E-49 |
| ENSMUSG00000020836 | 1.520333333 | 0.293333333 | -2.373774741 | 7.85015E-07 | 1.70238E-07 |
| ENSMUSG00000032353 | 104.7533333 | 20.19066667 | -2.3752357 | 7.9254E-135 | 8.8644E-137 |
| ENSMUSG00000066442 | 4.700333333 | 0.904666667 | -2.377304852 | 1.26857E-06 | 2.84123E-07 |
| ENSMUSG00000032554 | 90.54333333 | 17.405 | -2.379106606 | 7.2236E-161 | 6.4336E-163 |
| ENSMUSG00000104346 | 0.418666667 | 0.080333333 | -2.381731413 | 0.001178468 | 0.000393148 |
| ENSMUSG00000002204 | 33.55433333 | 6.430333333 | -2.383533663 | 2.603E-65 | 6.18221E-67 |
| ENSMUSG00000020669 | 5.114 | 0.979333333 | -2.384580265 | 8.35522E-15 | 9.3278E-16 |
| ENSMUSG00000036902 | 12.77666667 | 2.434333333 | -2.391912862 | 2.35962E-66 | 5.52271E-68 |
| ENSMUSG00000020077 | 928.756 | 176.837 | -2.392879461 | 1.49084E-22 | 1.10753E-23 |
| ENSMUSG00000030124 | 5.917 | 1.126 | -2.393659068 | 3.33065E-20 | 2.76174E-21 |
| ENSMUSG00000034579 | 1.102666667 | 0.209333333 | -2.39712277 | 4.74268E-05 | 1.29307E-05 |
| ENSMUSG00000021065 | 206.7893333 | 39.14133333 | -2.401396963 | 8.1735E-131 | 9.3112E-133 |
| ENSMUSG00000036644 | 104.9926667 | 19.85566667 | -2.402665859 | 4.8731E-199 | 3.3308E-201 |
| ENSMUSG00000042042 | 182.3963333 | 34.40433333 | -2.406414534 | 1.1743E-182 | 9.0803E-185 |
| ENSMUSG00000074417 | 0.589333333 | 0.110333333 | -2.417215153 | 0.003852052 | 0.001418057 |
| ENSMUSG00000030365 | 37.10066667 | 6.903666667 | -2.426010398 | 6.84504E-45 | 2.44803E-46 |
| ENSMUSG00000078716 | 0.593333333 | 0.110333333 | -2.426974119 | 0.000289095 | 8.75229E-05 |
| ENSMUSG00000028977 | 1.706666667 | 0.316666667 | -2.430144392 | 3.81963E-20 | 3.19094E-21 |
| ENSMUSG00000009739 | 0.957666667 | 0.176666667 | -2.438493728 | 5.16353E-08 | 9.89998E-09 |
| ENSMUSG00000032380 | 1.824666667 | 0.336 | -2.441099796 | 2.14887E-05 | 5.57988E-06 |
| ENSMUSG00000040231 | 1.118333333 | 0.205666667 | -2.442970372 | 0.013061387 | 0.005314192 |
| ENSMUSG00000045312 | 0.582666667 | 0.107 | -2.445059982 | 0.001327066 | 0.000446753 |

| | | | | | |
|---|---|---|---|---|---|
| ENSMUSG00000034714 | 4.995 | 0.915666667 | -2.447590268 | 3.38618E-22 | 2.55764E-23 |
| ENSMUSG00000023828 | 31.89233333 | 5.844 | -2.44818157 | 9.05274E-73 | 1.9563E-74 |
| ENSMUSG00000025407 | 0.747666667 | 0.137 | -2.448219321 | 0.000344109 | 0.0001052 |
| ENSMUSG00000024334 | 30.25166667 | 5.535666667 | -2.45018565 | 3.30685E-53 | 9.79453E-55 |
| ENSMUSG00000024827 | 0.345333333 | 0.062666667 | -2.462219436 | 0.003890866 | 0.001434495 |
| ENSMUSG00000056234 | 279.0896667 | 50.50366667 | -2.46626867 | 7.22E-155 | 6.7295E-157 |
| ENSMUSG00000057132 | 0.831 | 0.148666667 | -2.482767268 | 0.000945477 | 0.000310394 |
| ENSMUSG00000074657 | 0.919666667 | 0.164 | -2.487415236 | 1.41591E-10 | 2.17803E-11 |
| ENSMUSG00000047394 | 3.330333333 | 0.584 | -2.51162631 | 0.000200376 | 5.94876E-05 |
| ENSMUSG00000035513 | 4.958666667 | 0.862 | -2.524192473 | 1.26158E-15 | 1.3405E-16 |
| ENSMUSG00000073600 | 4.572 | 0.784333333 | -2.543286584 | 1.40418E-27 | 8.42471E-29 |
| ENSMUSG00000000915 | 20.37766667 | 3.478333333 | -2.550520766 | 1.9826E-151 | 1.9164E-153 |
| ENSMUSG00000020057 | 0.707666667 | 0.12 | -2.56003556 | 0.000188716 | 5.57718E-05 |
| ENSMUSG00000018796 | 58.71466667 | 9.926333333 | -2.56438812 | 1.4766E-155 | 1.3559E-157 |
| ENSMUSG00000026944 | 76.14033333 | 12.72533333 | -2.580957438 | 1.4758E-135 | 1.6302E-137 |
| ENSMUSG00000021000 | 206.2533333 | 34.265 | -2.589609843 | 1.5109E-187 | 1.1266E-189 |
| ENSMUSG00000039982 | 8.693 | 1.442333333 | -2.591449525 | 6.06151E-57 | 1.64051E-58 |
| ENSMUSG00000032850 | 0.167333333 | 0.027666667 | -2.596504123 | 0.016275408 | 0.006785915 |
| ENSMUSG00000022952 | 104.8183333 | 17.32566667 | -2.596908303 | 7.39751E-63 | 1.83865E-64 |
| ENSMUSG00000026728 | 183.41 | 30.264 | -2.599397714 | 1.779E-145 | 1.8301E-147 |
| ENSMUSG00000078653 | 1.535333333 | 0.253333333 | -2.599443087 | 3.03913E-07 | 6.27801E-08 |
| ENSMUSG00000039824 | 15.06433333 | 2.481666667 | -2.601755575 | 1.51936E-27 | 9.12622E-29 |
| ENSMUSG00000006457 | 1.314 | 0.216333333 | -2.602637393 | 1.47241E-07 | 2.94808E-08 |
| ENSMUSG00000032905 | 52.355 | 8.594666667 | -2.606813731 | 3.6303E-119 | 4.6369E-121 |
| ENSMUSG00000026544 | 4.459 | 0.731333333 | -2.608119175 | 3.73457E-13 | 4.65403E-14 |
| ENSMUSG00000031258 | 0.256333333 | 0.042 | -2.609559864 | 0.030114994 | 0.013324605 |
| ENSMUSG00000020256 | 1.165 | 0.19 | -2.616258631 | 5.47777E-11 | 8.11983E-12 |
| ENSMUSG00000055172 | 14.24966667 | 2.316333333 | -2.621013386 | 4.61192E-60 | 1.18769E-61 |
| ENSMUSG00000041040 | 27.16566667 | 4.406 | -2.624243052 | 6.7749E-167 | 5.9405E-169 |
| ENSMUSG00000089984 | 0.338 | 0.054666667 | -2.628289932 | 0.045480807 | 0.021088726 |
| ENSMUSG00000054892 | 1.453666667 | 0.233 | -2.64129463 | 0.000108267 | 3.10061E-05 |
| ENSMUSG00000040998 | 1.315333333 | 0.210333333 | -2.644679046 | 2.65393E-09 | 4.52215E-10 |

| | | | | | |
|---|---|---|---|---|---|
| ENSMUSG00000026004 | 8.197666667 | 1.310333333 | -2.645279465 | 1.34885E-42 | 5.16853E-44 |
| ENSMUSG00000032890 | 1.967333333 | 0.314333333 | -2.645874245 | 7.26331E-22 | 5.55631E-23 |
| ENSMUSG00000062232 | 21.516 | 3.437 | -2.646188137 | 4.84E-171 | 4.0099E-173 |
| ENSMUSG00000028476 | 3.875 | 0.616333333 | -2.652413586 | 2.19025E-33 | 1.0918E-34 |
| ENSMUSG00000038074 | 3.364666667 | 0.534333333 | -2.654651661 | 9.40983E-18 | 8.83552E-19 |
| ENSMUSG00000058624 | 0.164666667 | 0.026 | -2.662965013 | 0.026994479 | 0.011819869 |
| ENSMUSG00000028763 | 0.143 | 0.022333333 | -2.678744647 | 0.000185785 | 5.48478E-05 |
| ENSMUSG00000071648 | 6.805 | 1.057333333 | -2.686164891 | 6.62644E-20 | 5.62726E-21 |
| ENSMUSG00000041058 | 68.66533333 | 10.64433333 | -2.689496323 | 4.2526E-204 | 2.7599E-206 |
| ENSMUSG00000041073 | 0.391 | 0.060333333 | -2.696141411 | 0.029494801 | 0.013018521 |
| ENSMUSG00000059708 | 1.686 | 0.259333333 | -2.700724977 | 8.72356E-15 | 9.76914E-16 |
| ENSMUSG00000027843 | 10.441 | 1.599666667 | -2.706416677 | 3.79304E-45 | 1.34605E-46 |
| ENSMUSG00000050860 | 13.78066667 | 2.105 | -2.710753545 | 9.04015E-21 | 7.31501E-22 |
| ENSMUSG00000031762 | 221.6886667 | 33.83766667 | -2.711833024 | 2.83429E-62 | 7.10333E-64 |
| ENSMUSG00000114694 | 0.535333333 | 0.081666667 | -2.712618239 | 0.036893241 | 0.016683977 |
| ENSMUSG00000056215 | 0.761666667 | 0.116 | -2.715034954 | 7.64942E-11 | 1.1434E-11 |
| ENSMUSG00000047822 | 11.508 | 1.74 | -2.725477915 | 2.87601E-10 | 4.56103E-11 |
| ENSMUSG00000107705 | 17.50466667 | 2.642333333 | -2.727855209 | 5.76692E-08 | 1.11126E-08 |
| ENSMUSG00000029061 | 0.476 | 0.071 | -2.745070644 | 0.028697269 | 0.012633116 |
| ENSMUSG00000039716 | 0.208666667 | 0.031 | -2.750860036 | 0.033365894 | 0.014932182 |
| ENSMUSG00000014496 | 104.323 | 15.43133333 | -2.757122635 | 1.4397E-168 | 1.2325E-170 |
| ENSMUSG00000079227 | 0.097 | 0.014333333 | -2.758610588 | 0.014077994 | 0.005780298 |
| ENSMUSG00000074627 | 0.153666667 | 0.022666667 | -2.761160099 | 0.04473689 | 0.020688186 |
| ENSMUSG00000060131 | 75.86466667 | 11.08566667 | -2.774732586 | 1.6568E-116 | 2.1849E-118 |
| ENSMUSG00000026483 | 51.94133333 | 7.583666667 | -2.77591559 | 3.5917E-250 | 1.6863E-252 |
| ENSMUSG00000029716 | 0.870666667 | 0.126 | -2.788696757 | 6.85312E-06 | 1.67117E-06 |
| ENSMUSG00000027737 | 3.860666667 | 0.558666667 | -2.788790348 | 6.33446E-48 | 2.10361E-49 |
| ENSMUSG00000044022 | 0.549 | 0.079 | -2.796881591 | 2.45979E-05 | 6.43648E-06 |
| ENSMUSG00000068220 | 495.6626667 | 70.31133333 | -2.817529441 | 1.4264E-96 | 2.32416E-98 |
| ENSMUSG00000055717 | 12.86 | 1.822333333 | -2.819031863 | 1.65861E-43 | 6.1837E-45 |
| ENSMUSG00000054200 | 1.522 | 0.214 | -2.830285657 | 0.000127648 | 3.68384E-05 |
| ENSMUSG00000099583 | 0.79 | 0.111 | -2.831292977 | 0.004302902 | 0.001598586 |

| | | | | | |
|---|---|---|---|---|---|
| ENSMUSG00000060002 | 10.604 | 1.471333333 | -2.849412541 | 1.66603E-61 | 4.23294E-63 |
| ENSMUSG00000026748 | 33.48966667 | 4.643 | -2.85058683 | 8.382E-126 | 1.0012E-127 |
| ENSMUSG00000031012 | 23.86233333 | 3.298333333 | -2.854926009 | 6.6949E-145 | 6.9797E-147 |
| ENSMUSG00000096054 | 30.66633333 | 4.237 | -2.855540645 | 3.4295E-239 | 1.7522E-241 |
| ENSMUSG00000019027 | 0.065333333 | 0.009 | -2.859822342 | 0.011160675 | 0.004478448 |
| ENSMUSG00000024480 | 259.8293333 | 35.65833333 | -2.865253134 | 0 | 0 |
| ENSMUSG00000038352 | 0.627333333 | 0.084666667 | -2.889366226 | 0.010013059 | 0.003982687 |
| ENSMUSG00000025355 | 1.513 | 0.202333333 | -2.902606067 | 1.15347E-08 | 2.08013E-09 |
| ENSMUSG00000047246 | 0.371666667 | 0.049666667 | -2.903659474 | 0.011395231 | 0.004578076 |
| ENSMUSG00000034949 | 1.230333333 | 0.164333333 | -2.904352186 | 1.7243E-07 | 3.4786E-08 |
| ENSMUSG00000103332 | 0.390333333 | 0.052 | -2.908123142 | 3.87928E-05 | 1.04321E-05 |
| ENSMUSG00000024842 | 0.531 | 0.07 | -2.923285034 | 0.013969967 | 0.00573112 |
| ENSMUSG00000087385 | 1.406666667 | 0.182 | -2.950270143 | 1.71384E-06 | 3.8953E-07 |
| ENSMUSG00000031503 | 0.494 | 0.063666667 | -2.955900904 | 3.4791E-07 | 7.23011E-08 |
| ENSMUSG00000036158 | 4.725333333 | 0.592333333 | -2.995934923 | 1.37187E-38 | 5.79665E-40 |
| ENSMUSG00000000127 | 0.167666667 | 0.021 | -2.997134666 | 0.048941047 | 0.02286889 |
| ENSMUSG00000022824 | 13.501 | 1.671666667 | -3.013707164 | 1.23938E-11 | 1.73534E-12 |
| ENSMUSG00000034818 | 0.293666667 | 0.036333333 | -3.014813884 | 0.003150312 | 0.00113928 |
| ENSMUSG00000045257 | 3.504666667 | 0.433 | -3.016838304 | 3.07007E-06 | 7.17918E-07 |
| ENSMUSG00000034177 | 3.252333333 | 0.401666667 | -3.017404482 | 6.07681E-27 | 3.755E-28 |
| ENSMUSG00000001552 | 8.554666667 | 1.056333333 | -3.017646482 | 9.04828E-53 | 2.72373E-54 |
| ENSMUSG00000027009 | 130.2753333 | 16.072 | -3.018942572 | 2.0182E-196 | 1.3934E-198 |
| ENSMUSG00000061451 | 5.487 | 0.676 | -3.020922424 | 7.20874E-42 | 2.81202E-43 |
| ENSMUSG00000019301 | 4.136 | 0.505 | -3.033880893 | 8.96151E-11 | 1.35004E-11 |
| ENSMUSG00000000706 | 0.327666667 | 0.04 | -3.034157011 | 0.016796279 | 0.007026281 |
| ENSMUSG00000040528 | 0.929 | 0.112666667 | -3.043617851 | 0.000854508 | 0.000278582 |
| ENSMUSG00000034145 | 0.403333333 | 0.048666667 | -3.050966773 | 0.000399223 | 0.000123538 |
| ENSMUSG00000063455 | 0.131333333 | 0.015666667 | -3.067462968 | 0.000611113 | 0.000194506 |
| ENSMUSG00000041991 | 0.272333333 | 0.032333333 | -3.074279426 | 4.43512E-07 | 9.3271E-08 |
| ENSMUSG00000014905 | 77.61766667 | 9.185 | -3.079033437 | 1.9689E-249 | 9.3794E-252 |
| ENSMUSG00000023032 | 0.960666667 | 0.113666667 | -3.079226691 | 9.51102E-08 | 1.87476E-08 |
| ENSMUSG00000037940 | 31.51733333 | 3.662 | -3.105441778 | 2.55022E-25 | 1.66564E-26 |

| | | | | | |
|---|---|---|---|---|---|
| ENSMUSG00000032198 | 0.358666667 | 0.041666667 | -3.105678078 | 6.41168E-06 | 1.55688E-06 |
| ENSMUSG00000043019 | 66.91433333 | 7.687666667 | -3.121697587 | 2.4286E-278 | 9.0546E-281 |
| ENSMUSG00000048216 | 0.228666667 | 0.026 | -3.136662547 | 0.03062209 | 0.013579514 |
| ENSMUSG00000000982 | 3.984666667 | 0.450333333 | -3.145393868 | 1.71894E-07 | 3.46542E-08 |
| ENSMUSG00000032446 | 0.376 | 0.041666667 | -3.173767068 | 0.000243795 | 7.31014E-05 |
| ENSMUSG00000040013 | 1.372 | 0.151333333 | -3.18047878 | 3.144E-05 | 8.32453E-06 |
| ENSMUSG00000030824 | 404.162 | 44.27733333 | -3.190293442 | 3.2349E-270 | 1.2284E-272 |
| ENSMUSG00000063129 | 2.308 | 0.251 | -3.200883955 | 3.11507E-09 | 5.33803E-10 |
| ENSMUSG00000023935 | 0.577666667 | 0.062666667 | -3.204467087 | 0.014772065 | 0.006093834 |
| ENSMUSG00000047528 | 0.404 | 0.043333333 | -3.22080617 | 0.032153953 | 0.014332085 |
| ENSMUSG00000022636 | 0.476333333 | 0.050666667 | -3.232862688 | 0.005109069 | 0.001922074 |
| ENSMUSG00000028266 | 196.547 | 20.79633333 | -3.240473253 | 2.6179E-252 | 1.211E-254 |
| ENSMUSG00000044719 | 0.284 | 0.03 | -3.242856524 | 0.026369735 | 0.011526291 |
| ENSMUSG00000036052 | 6.645 | 0.699666667 | -3.247529534 | 4.98995E-37 | 2.22212E-38 |
| ENSMUSG00000015083 | 0.504333333 | 0.052666667 | -3.259415524 | 0.026657281 | 0.011663021 |
| ENSMUSG00000032221 | 57.06966667 | 5.936666667 | -3.264999121 | 8.6976E-146 | 8.8273E-148 |
| ENSMUSG00000035863 | 33.15866667 | 3.441333333 | -3.26834645 | 5.3702E-121 | 6.7109E-123 |
| ENSMUSG00000027962 | 0.231333333 | 0.024 | -3.268866851 | 0.007332642 | 0.002842133 |
| ENSMUSG00000034401 | 5.492333333 | 0.568 | -3.273456351 | 1.22619E-23 | 8.77058E-25 |
| ENSMUSG00000026655 | 239.9913333 | 24.79333333 | -3.274958155 | 0 | 0 |
| ENSMUSG00000033233 | 14.47566667 | 1.494666667 | -3.27573411 | 2.15865E-61 | 5.51436E-63 |
| ENSMUSG00000038604 | 90.30833333 | 9.210333333 | -3.293533845 | 3.8023E-221 | 2.1264E-223 |
| ENSMUSG00000032323 | 281.6316667 | 28.458 | -3.30690338 | 0 | 0 |
| ENSMUSG00000003814 | 3052.664 | 307.322 | -3.312245851 | 0 | 0 |
| ENSMUSG00000078234 | 0.236333333 | 0.023666667 | -3.319894698 | 3.72105E-05 | 9.96802E-06 |
| ENSMUSG00000031962 | 0.240333333 | 0.024 | -3.323930448 | 0.007728703 | 0.003007919 |
| ENSMUSG00000028758 | 0.701333333 | 0.07 | -3.324673472 | 2.07354E-06 | 4.74292E-07 |
| ENSMUSG00000034647 | 38.78266667 | 3.862333333 | -3.327867421 | 6.8242E-255 | 2.9683E-257 |
| ENSMUSG00000017417 | 1.609 | 0.16 | -3.330020516 | 2.00741E-12 | 2.63609E-13 |
| ENSMUSG00000000730 | 4.095333333 | 0.406666667 | -3.332062236 | 4.55703E-12 | 6.1698E-13 |
| ENSMUSG00000030413 | 12.244 | 1.212666667 | -3.335820003 | 1.49003E-19 | 1.28592E-20 |
| ENSMUSG00000032436 | 378.9706667 | 37.48166667 | -3.337829174 | 0 | 0 |

| | | | | | |
|---|---|---|---|---|---|
| ENSMUSG00000000686 | 13.622 | 1.339 | -3.346710672 | 1.38826E-59 | 3.59429E-61 |
| ENSMUSG00000028121 | 0.724 | 0.070666667 | -3.356887933 | 0.000222717 | 6.65507E-05 |
| ENSMUSG00000000632 | 3.171333333 | 0.304666667 | -3.379785955 | 6.34399E-22 | 4.83114E-23 |
| ENSMUSG00000023972 | 0.285 | 0.027333333 | -3.382228605 | 0.000711792 | 0.000229057 |
| ENSMUSG00000047492 | 0.883 | 0.083333333 | -3.405447844 | 1.7788E-11 | 2.52623E-12 |
| ENSMUSG00000059854 | 0.028333333 | 0.002666667 | -3.409390936 | 0.047200946 | 0.021954762 |
| ENSMUSG00000018102 | 7.418333333 | 0.694666667 | -3.416702318 | 5.52935E-23 | 4.03271E-24 |
| ENSMUSG00000028445 | 0.803 | 0.075 | -3.420437487 | 0.001927228 | 0.000669021 |
| ENSMUSG00000073591 | 1.378666667 | 0.128333333 | -3.425305835 | 6.93715E-16 | 7.28008E-17 |
| ENSMUSG00000042333 | 9.531333333 | 0.885666667 | -3.427842319 | 1.55457E-12 | 2.02532E-13 |
| ENSMUSG00000025408 | 52.203 | 4.770333333 | -3.451970733 | 2.5745E-105 | 3.8572E-107 |
| ENSMUSG00000067455 | 0.604666667 | 0.054666667 | -3.467406736 | 0.043949224 | 0.020284491 |
| ENSMUSG00000036377 | 14.21433333 | 1.283333333 | -3.469378587 | 4.94414E-97 | 8.02176E-99 |
| ENSMUSG00000025491 | 193.3856667 | 17.417 | -3.472912817 | 2.4482E-177 | 1.9607E-179 |
| ENSMUSG00000029638 | 45.484 | 4.073 | -3.481195415 | 1.02609E-16 | 1.02227E-17 |
| ENSMUSG00000002033 | 4.215666667 | 0.369 | -3.514068076 | 8.40436E-12 | 1.1605E-12 |
| ENSMUSG00000025420 | 1.161 | 0.100333333 | -3.532495081 | 4.16854E-07 | 8.7377E-08 |
| ENSMUSG00000051839 | 12.976 | 1.12 | -3.534275087 | 3.64578E-43 | 1.36931E-44 |
| ENSMUSG00000030306 | 0.054333333 | 0.004666667 | -3.541373232 | 0.040088925 | 0.018287721 |
| ENSMUSG00000000142 | 2.912 | 0.248333333 | -3.551660526 | 4.04926E-24 | 2.82084E-25 |
| ENSMUSG00000095648 | 0.596333333 | 0.050333333 | -3.566532933 | 0.002045466 | 0.000714162 |
| ENSMUSG00000026482 | 1.768666667 | 0.149 | -3.569277939 | 2.09597E-17 | 2.02159E-18 |
| ENSMUSG00000028555 | 3.849666667 | 0.323333333 | -3.57363938 | 5.05505E-26 | 3.20391E-27 |
| ENSMUSG00000039286 | 10.11366667 | 0.838333333 | -3.592638331 | 5.8773E-153 | 5.5186E-155 |
| ENSMUSG00000046727 | 7.261333333 | 0.596 | -3.606850246 | 3.2038E-17 | 3.12549E-18 |
| ENSMUSG00000026880 | 14.50833333 | 1.176333333 | -3.624512963 | 1.80184E-100 | 2.8612E-102 |
| ENSMUSG00000041329 | 1.545 | 0.124 | -3.639194812 | 3.48765E-12 | 4.67862E-13 |
| ENSMUSG00000029470 | 32.02666667 | 2.557666667 | -3.646373491 | 1.1801E-117 | 1.5399E-119 |
| ENSMUSG00000004552 | 4.954666667 | 0.395333333 | -3.647646492 | 2.32587E-25 | 1.51589E-26 |
| ENSMUSG00000042532 | 0.189333333 | 0.015 | -3.657894023 | 0.023979156 | 0.010391961 |
| ENSMUSG00000030851 | 5.344333333 | 0.421666667 | -3.663835109 | 3.6104E-18 | 3.32025E-19 |
| ENSMUSG00000007021 | 1.407333333 | 0.110333333 | -3.673023457 | 9.7631E-08 | 1.92714E-08 |

| | | | | | |
|---|---|---|---|---|---|
| ENSMUSG00000054136 | 9.291333333 | 0.722 | -3.6858149 | 5.37737E-31 | 2.86615E-32 |
| ENSMUSG00000039765 | 1.265333333 | 0.097666667 | -3.695507423 | 9.22924E-16 | 9.71734E-17 |
| ENSMUSG00000039942 | 0.151333333 | 0.011666667 | -3.69726547 | 0.026923157 | 0.011784922 |
| ENSMUSG00000026764 | 0.268333333 | 0.020666667 | -3.698648663 | 1.72968E-05 | 4.43647E-06 |
| ENSMUSG00000030643 | 1.713 | 0.131666667 | -3.701563094 | 8.18197E-10 | 1.34558E-10 |
| ENSMUSG00000025432 | 22.44833333 | 1.700333333 | -3.722718832 | 7.3271E-152 | 6.9305E-154 |
| ENSMUSG00000035064 | 57.18733333 | 4.301 | -3.732951597 | 3.5782E-264 | 1.4082E-266 |
| ENSMUSG00000046006 | 72.23533333 | 5.271333333 | -3.776464863 | 5.83498E-37 | 2.61051E-38 |
| ENSMUSG00000114582 | 0.599 | 0.043666667 | -3.777951692 | 0.001585234 | 0.000542421 |
| ENSMUSG00000056888 | 68.40866667 | 4.984 | -3.778803138 | 1.4627E-157 | 1.323E-159 |
| ENSMUSG00000022475 | 57.87933333 | 4.091333333 | -3.822405317 | 1.4012E-282 | 5.1274E-285 |
| ENSMUSG00000027122 | 577.254 | 40.503 | -3.83310559 | 0 | 0 |
| ENSMUSG00000051682 | 1.780333333 | 0.122333333 | -3.863257916 | 6.78812E-09 | 1.19603E-09 |
| ENSMUSG00000000409 | 14.265 | 0.97 | -3.878351189 | 4.50078E-78 | 9.10472E-80 |
| ENSMUSG00000068699 | 1.027666667 | 0.069666667 | -3.882760042 | 4.62838E-25 | 3.06769E-26 |
| ENSMUSG00000004319 | 16.193 | 1.086 | -3.898274283 | 8.49493E-07 | 1.85101E-07 |
| ENSMUSG00000042265 | 3.645333333 | 0.243666667 | -3.903069932 | 5.27397E-27 | 3.24799E-28 |
| ENSMUSG00000020620 | 0.060666667 | 0.004 | -3.922832139 | 0.038931912 | 0.017691925 |
| ENSMUSG00000033029 | 1.588333333 | 0.104 | -3.93285828 | 1.59755E-05 | 4.08102E-06 |
| ENSMUSG00000005677 | 0.445333333 | 0.029 | -3.940760797 | 0.00151187 | 0.00051558 |
| ENSMUSG00000036390 | 89.99133333 | 5.856666667 | -3.941634378 | 2.1265E-189 | 1.5122E-191 |
| ENSMUSG00000059495 | 0.174333333 | 0.011333333 | -3.943204295 | 5.40477E-06 | 1.30343E-06 |
| ENSMUSG00000031760 | 3.449666667 | 0.221666667 | -3.959993219 | 4.59429E-06 | 1.10036E-06 |
| ENSMUSG00000028864 | 0.140333333 | 0.009 | -3.962788921 | 0.02021851 | 0.008608641 |
| ENSMUSG00000052435 | 74.201 | 4.707333333 | -3.978456709 | 5.6164E-201 | 3.7613E-203 |
| ENSMUSG00000020395 | 0.415666667 | 0.026333333 | -3.980465002 | 4.2138E-05 | 1.13869E-05 |
| ENSMUSG00000042700 | 119.3723333 | 7.501 | -3.992241753 | 0 | 0 |
| ENSMUSG00000037913 | 41.235 | 2.586 | -3.995075227 | 4.394E-139 | 4.7629E-141 |
| ENSMUSG00000030657 | 11.286 | 0.707333333 | -3.996000194 | 1.1707E-187 | 8.6481E-190 |
| ENSMUSG00000030303 | 15.878 | 0.994 | -3.997639539 | 1.7265E-124 | 2.1098E-126 |
| ENSMUSG00000054619 | 115.955 | 7.254666667 | -3.998511893 | 0 | 0 |
| ENSMUSG00000032348 | 21.67666667 | 1.350333333 | -4.004755434 | 7.32676E-53 | 2.1954E-54 |

| | | | | | |
|---|---|---|---|---|---|
| ENSMUSG00000089951 | 0.566333333 | 0.035 | -4.01622462 | 0.043829656 | 0.020226279 |
| ENSMUSG00000004891 | 0.103666667 | 0.006333333 | -4.032843257 | 0.012287771 | 0.004969743 |
| ENSMUSG00000054191 | 31.14966667 | 1.893 | -4.040470409 | 1.0303E-102 | 1.6075E-104 |
| ENSMUSG00000107877 | 1.152 | 0.069666667 | -4.04752837 | 4.2194E-07 | 8.85013E-08 |
| ENSMUSG00000067220 | 2.462666667 | 0.148 | -4.056552285 | 9.29299E-15 | 1.04132E-15 |
| ENSMUSG00000030142 | 42.72433333 | 2.564666667 | -4.058214746 | 1.24923E-33 | 6.18404E-35 |
| ENSMUSG00000017195 | 0.274 | 0.016 | -4.098032083 | 0.039031777 | 0.017745392 |
| ENSMUSG00000025804 | 3.565666667 | 0.207333333 | -4.104147855 | 4.67646E-27 | 2.86709E-28 |
| ENSMUSG00000028182 | 0.654333333 | 0.037 | -4.144428591 | 0.002313812 | 0.00081616 |
| ENSMUSG00000008129 | 1.050666667 | 0.059 | -4.154446269 | 1.91031E-07 | 3.87418E-08 |
| ENSMUSG00000029468 | 5.371333333 | 0.301 | -4.157444863 | 1.68148E-56 | 4.57403E-58 |
| ENSMUSG00000040165 | 31.83166667 | 1.778 | -4.162135467 | 1.6489E-101 | 2.607E-103 |
| ENSMUSG00000026638 | 0.34 | 0.018666667 | -4.186998515 | 5.53023E-05 | 1.52345E-05 |
| ENSMUSG00000025425 | 0.195666667 | 0.010666667 | -4.197216693 | 0.026331955 | 0.011507959 |
| ENSMUSG00000004612 | 7.932666667 | 0.431 | -4.202046153 | 1.07434E-17 | 1.01322E-18 |
| ENSMUSG00000026435 | 0.753333333 | 0.039333333 | -4.259464008 | 2.36576E-07 | 4.82496E-08 |
| ENSMUSG00000031860 | 3.906 | 0.201333333 | -4.278033995 | 3.25663E-13 | 4.04268E-14 |
| ENSMUSG00000024008 | 1.145333333 | 0.058333333 | -4.295303209 | 3.18255E-18 | 2.9158E-19 |
| ENSMUSG00000035085 | 8.054333333 | 0.405333333 | -4.312584452 | 6.32755E-17 | 6.22533E-18 |
| ENSMUSG00000043639 | 0.311333333 | 0.015666667 | -4.312689888 | 8.76648E-05 | 2.47052E-05 |
| ENSMUSG00000021573 | 0.081 | 0.004 | -4.339850003 | 0.023939824 | 0.010373263 |
| ENSMUSG00000026259 | 0.245333333 | 0.012 | -4.353636955 | 0.022721334 | 0.009795085 |
| ENSMUSG00000045004 | 1.765333333 | 0.086333333 | -4.353879119 | 3.68323E-13 | 4.58497E-14 |
| ENSMUSG00000039304 | 0.163666667 | 0.008 | -4.354616714 | 0.003411816 | 0.001242331 |
| ENSMUSG00000026656 | 80.24766667 | 3.909333333 | -4.359464936 | 2.8136E-249 | 1.3598E-251 |
| ENSMUSG00000037661 | 2.087333333 | 0.101 | -4.36923381 | 1.246E-11 | 1.74632E-12 |
| ENSMUSG00000066952 | 0.441333333 | 0.021333333 | -4.370687407 | 5.76459E-05 | 1.59079E-05 |
| ENSMUSG00000035448 | 0.124666667 | 0.006 | -4.376969458 | 0.023853131 | 0.010332404 |
| ENSMUSG00000032584 | 1.729666667 | 0.082 | -4.398726315 | 3.57471E-21 | 2.85059E-22 |
| ENSMUSG00000040187 | 1.623666667 | 0.076666667 | -4.404512217 | 2.6352E-11 | 3.8007E-12 |
| ENSMUSG00000036944 | 1.950666667 | 0.091333333 | -4.416681971 | 4.7078E-16 | 4.88852E-17 |
| ENSMUSG00000047496 | 0.057 | 0.002666667 | -4.417852515 | 0.018179176 | 0.00765875 |

| | | | | | |
|---|---|---|---|---|---|
| ENSMUSG00000028838 | 5.136333333 | 0.237666667 | -4.433727351 | 1.0407E-48 | 3.38422E-50 |
| ENSMUSG00000028280 | 0.173 | 0.008 | -4.434628228 | 0.031700198 | 0.014110134 |
| ENSMUSG00000031616 | 0.131 | 0.006 | -4.448460501 | 0.01555819 | 0.006447173 |
| ENSMUSG00000027233 | 0.716 | 0.032666667 | -4.454068434 | 1.37294E-05 | 3.48069E-06 |
| ENSMUSG00000072419 | 0.242333333 | 0.011 | -4.461417435 | 0.012937812 | 0.005254981 |
| ENSMUSG00000036330 | 0.268 | 0.012 | -4.48112669 | 0.003187737 | 0.001154795 |
| ENSMUSG00000034833 | 7.022666667 | 0.299666667 | -4.550588439 | 5.73816E-29 | 3.27238E-30 |
| ENSMUSG00000048652 | 4.643 | 0.198 | -4.551484945 | 1.23297E-18 | 1.10409E-19 |
| ENSMUSG00000029651 | 2.779333333 | 0.118333333 | -4.553810442 | 1.22543E-11 | 1.71496E-12 |
| ENSMUSG00000034353 | 22.12166667 | 0.938666667 | -4.558703345 | 2.6506E-105 | 3.996E-107 |
| ENSMUSG00000079343 | 0.350666667 | 0.014333333 | -4.612654235 | 0.001464644 | 0.000498428 |
| ENSMUSG00000030134 | 1.481 | 0.060333333 | -4.617472539 | 3.93658E-12 | 5.29986E-13 |
| ENSMUSG00000067399 | 1.443333333 | 0.057666667 | -4.645523082 | 7.52049E-08 | 1.46578E-08 |
| ENSMUSG00000025934 | 0.369 | 0.014666667 | -4.653007888 | 0.044127956 | 0.020376123 |
| ENSMUSG00000041794 | 0.330333333 | 0.013 | -4.667339028 | 0.006576083 | 0.002528006 |
| ENSMUSG00000030470 | 1.628 | 0.063 | -4.691605061 | 6.99129E-05 | 1.9499E-05 |
| ENSMUSG00000030674 | 0.952666667 | 0.036 | -4.725902699 | 4.39156E-06 | 1.0512E-06 |
| ENSMUSG00000031997 | 15.018 | 0.566 | -4.729746834 | 3.75516E-10 | 6.02008E-11 |
| ENSMUSG00000029322 | 496.4316667 | 18.27766667 | -4.763441334 | 0 | 0 |
| ENSMUSG00000036587 | 23.25033333 | 0.853 | -4.768561848 | 1.3613E-112 | 1.8986E-114 |
| ENSMUSG00000053338 | 2.439 | 0.089 | -4.776340612 | 2.8735E-09 | 4.90931E-10 |
| ENSMUSG00000078872 | 27.482 | 0.999666667 | -4.780896073 | 1.25047E-71 | 2.72817E-73 |
| ENSMUSG00000026649 | 2.194666667 | 0.076666667 | -4.839258569 | 3.66533E-06 | 8.65468E-07 |
| ENSMUSG00000079033 | 1.732666667 | 0.060333333 | -4.843895031 | 5.22082E-07 | 1.10912E-07 |
| ENSMUSG00000053101 | 32.034 | 1.108333333 | -4.85314021 | 2.8418E-183 | 2.1582E-185 |
| ENSMUSG00000045868 | 0.292 | 0.01 | -4.867896464 | 0.045765946 | 0.021243058 |
| ENSMUSG00000004988 | 1.859333333 | 0.061666667 | -4.914150759 | 0.000109296 | 3.13384E-05 |
| ENSMUSG00000039851 | 8.844333333 | 0.292333333 | -4.919067153 | 2.56601E-52 | 7.79511E-54 |
| ENSMUSG00000020009 | 219.3943333 | 7.117666667 | -4.945978083 | 0 | 0 |
| ENSMUSG00000040747 | 188.3593333 | 5.987666667 | -4.975349803 | 0 | 0 |
| ENSMUSG00000022221 | 0.676666667 | 0.021333333 | -4.987264012 | 0.002421039 | 0.000857159 |
| ENSMUSG00000070601 | 0.032 | 0.001 | -5 | 0.01651553 | 0.006900855 |

| | | | | | |
|---|---|---|---|---|---|
| ENSMUSG00000074028 | 0.640333333 | 0.02 | -5.000751208 | 0.000173685 | 5.10239E-05 |
| ENSMUSG00000019982 | 316.9016667 | 9.737 | -5.024414095 | 0 | 0 |
| ENSMUSG00000062713 | 1.331333333 | 0.040333333 | -5.04475538 | 2.01641E-15 | 2.17735E-16 |
| ENSMUSG00000042286 | 3.488333333 | 0.105666667 | -5.044945661 | 4.04467E-38 | 1.72856E-39 |
| ENSMUSG00000022885 | 0.603333333 | 0.017333333 | -5.121334264 | 0.020531565 | 0.008763196 |
| ENSMUSG00000007480 | 10.87033333 | 0.304333333 | -5.158600011 | 6.3987E-102 | 1.0072E-103 |
| ENSMUSG00000026837 | 12.24633333 | 0.341 | -5.166434308 | 1.12077E-83 | 2.08926E-85 |
| ENSMUSG00000024070 | 21.22866667 | 0.585 | -5.181433326 | 1.1605E-182 | 8.8936E-185 |
| ENSMUSG00000030468 | 0.812333333 | 0.022 | -5.206496416 | 1.13689E-06 | 2.52669E-07 |
| ENSMUSG00000078498 | 0.262 | 0.007 | -5.226068079 | 0.001552264 | 0.000530496 |
| ENSMUSG00000053063 | 5.457666667 | 0.145666667 | -5.227541599 | 2.83444E-30 | 1.55186E-31 |
| ENSMUSG00000036067 | 2.713 | 0.072333333 | -5.229084601 | 8.03036E-16 | 8.43842E-17 |
| ENSMUSG00000041608 | 1.489666667 | 0.039666667 | -5.230918566 | 6.12729E-15 | 6.81091E-16 |
| ENSMUSG00000041481 | 1.045 | 0.026666667 | -5.292321633 | 6.03996E-05 | 1.67221E-05 |
| ENSMUSG00000071714 | 389.6726667 | 9.936333333 | -5.293405356 | 0 | 0 |
| ENSMUSG00000076757 | 8.324 | 0.211666667 | -5.297410969 | 4.16694E-21 | 3.32573E-22 |
| ENSMUSG00000025997 | 101.5636667 | 2.516333333 | -5.33491753 | 0 | 0 |
| ENSMUSG00000030616 | 0.484 | 0.011666667 | -5.374542721 | 3.48162E-06 | 8.19686E-07 |
| ENSMUSG00000079259 | 0.041666667 | 0.001 | -5.380821784 | 0.007482341 | 0.002903772 |
| ENSMUSG00000031712 | 10.74933333 | 0.257666667 | -5.382597464 | 6.67107E-31 | 3.57872E-32 |
| ENSMUSG00000050108 | 1.546 | 0.037 | -5.384871238 | 2.43227E-11 | 3.5013E-12 |
| ENSMUSG00000053914 | 1.270666667 | 0.030333333 | -5.388537764 | 7.46091E-10 | 1.22288E-10 |
| ENSMUSG00000039783 | 2.524 | 0.059333333 | -5.410725265 | 1.72622E-18 | 1.55889E-19 |
| ENSMUSG00000027834 | 22.27333333 | 0.508333333 | -5.453398835 | 2.1056E-202 | 1.3956E-204 |
| ENSMUSG00000015437 | 94.951 | 2.159666667 | -5.458302632 | 0.001634846 | 0.000560525 |
| ENSMUSG00000026018 | 3.768 | 0.085 | -5.470192313 | 1.7258E-30 | 9.41302E-32 |
| ENSMUSG00000023411 | 1.959333333 | 0.044 | -5.476715524 | 8.18855E-17 | 8.10148E-18 |
| ENSMUSG00000019947 | 3.517 | 0.076666667 | -5.519602069 | 1.08553E-53 | 3.18524E-55 |
| ENSMUSG00000078907 | 0.57 | 0.012 | -5.569855608 | 1.36246E-05 | 3.45224E-06 |
| ENSMUSG00000024424 | 12.91833333 | 0.270666667 | -5.576758915 | 7.00272E-64 | 1.70668E-65 |
| ENSMUSG00000039735 | 1.12 | 0.023333333 | -5.584962501 | 5.1525E-15 | 5.70247E-16 |
| ENSMUSG00000032265 | 52.607 | 1.095666667 | -5.58537392 | 0 | 0 |

| | | | | | |
|---|---|---|---|---|---|
| ENSMUSG00000042082 | 36.479 | 0.757333333 | -5.589993942 | 0 | 0 |
| ENSMUSG00000058252 | 10.52733333 | 0.212 | -5.63393196 | 1.16786E-47 | 3.90255E-49 |
| ENSMUSG00000073008 | 3.908 | 0.077666667 | -5.652991108 | 3.07416E-13 | 3.81404E-14 |
| ENSMUSG00000070323 | 0.495 | 0.009666667 | -5.678266221 | 0.00083798 | 0.000272847 |
| ENSMUSG00000040229 | 12.739 | 0.222666667 | -5.83822262 | 1.09345E-58 | 2.86877E-60 |
| ENSMUSG00000030157 | 4.915333333 | 0.084 | -5.870756118 | 6.09282E-15 | 6.76839E-16 |
| ENSMUSG00000031714 | 3.916666667 | 0.066666667 | -5.876516947 | 1.04602E-45 | 3.6543E-47 |
| ENSMUSG00000014599 | 64.927 | 1.090333333 | -5.895977386 | 1.05944E-63 | 2.59665E-65 |
| ENSMUSG00000017950 | 0.766 | 0.012666667 | -5.918235569 | 4.84763E-09 | 8.46093E-10 |
| ENSMUSG00000118661 | 5.533333333 | 0.091 | -5.92613848 | 4.41222E-31 | 2.34868E-32 |
| ENSMUSG00000067219 | 0.064333333 | 0.001 | -6.007494537 | 0.040201847 | 0.018352873 |
| ENSMUSG00000071713 | 438.0576667 | 6.807666667 | -6.007816593 | 0 | 0 |
| ENSMUSG00000003617 | 0.709333333 | 0.011 | -6.010888316 | 4.22752E-06 | 1.00755E-06 |
| ENSMUSG00000045382 | 443.0433333 | 6.466 | -6.098430496 | 0 | 0 |
| ENSMUSG00000038068 | 1.86 | 0.026666667 | -6.124121312 | 3.71103E-18 | 3.41792E-19 |
| ENSMUSG00000039337 | 0.698 | 0.009666667 | -6.174064732 | 3.74605E-05 | 1.00453E-05 |
| ENSMUSG00000040065 | 0.072333333 | 0.001 | -6.176588732 | 0.032487192 | 0.014501335 |
| ENSMUSG00000040732 | 9.965333333 | 0.133333333 | -6.223808659 | 2.55825E-18 | 2.32793E-19 |
| ENSMUSG00000058216 | 2.179333333 | 0.029 | -6.231690166 | 1.537E-05 | 3.92102E-06 |
| ENSMUSG00000057751 | 0.076 | 0.001 | -6.247927513 | 0.002016558 | 0.000703373 |
| ENSMUSG00000026836 | 0.649333333 | 0.008333333 | -6.283921772 | 4.74672E-06 | 1.13752E-06 |
| ENSMUSG00000020323 | 6.760666667 | 0.086666667 | -6.285544489 | 2.21733E-22 | 1.6656E-23 |
| ENSMUSG00000035200 | 0.079 | 0.001 | -6.303780748 | 0.041691903 | 0.019100635 |
| ENSMUSG00000042581 | 1.136 | 0.014333333 | -6.308444866 | 5.6838E-11 | 8.44343E-12 |
| ENSMUSG00000020961 | 12.37666667 | 0.154666667 | -6.3223167 | 4.27354E-13 | 5.34045E-14 |
| ENSMUSG00000053702 | 9.305666667 | 0.113333333 | -6.359465359 | 5.8115E-13 | 7.32255E-14 |
| ENSMUSG00000102428 | 0.082333333 | 0.001 | -6.363404731 | 0.006706752 | 0.002581479 |
| ENSMUSG00000027073 | 302.7496667 | 3.593333333 | -6.396658789 | 7.24293E-10 | 1.18615E-10 |
| ENSMUSG00000021662 | 0.365666667 | 0.004333333 | -6.398908092 | 6.2931E-06 | 1.52765E-06 |
| ENSMUSG00000052273 | 0.085666667 | 0.001 | -6.420662048 | 0.000729973 | 0.000235311 |
| ENSMUSG00000047842 | 0.779333333 | 0.009 | -6.436171712 | 1.6806E-08 | 3.0818E-09 |
| ENSMUSG00000048924 | 3.463666667 | 0.039333333 | -6.460403832 | 1.63742E-18 | 1.47757E-19 |

| | | | | | |
|---|---|---|---|---|---|
| ENSMUSG00000030228 | 124.9216667 | 1.384333333 | -6.495688541 | 0 | 0 |
| ENSMUSG00000061928 | 0.844333333 | 0.009333333 | -6.49927644 | 5.13707E-08 | 9.83859E-09 |
| ENSMUSG00000026678 | 0.090666667 | 0.001 | -6.502500341 | 0.010643604 | 0.004254796 |
| ENSMUSG00000037159 | 0.096 | 0.001 | -6.584962501 | 0.042309844 | 0.019425605 |
| ENSMUSG00000035615 | 0.097333333 | 0.001 | -6.604862058 | 0.002836439 | 0.001016763 |
| ENSMUSG00000063683 | 7.798666667 | 0.078333333 | -6.637457327 | 1.31942E-43 | 4.87358E-45 |
| ENSMUSG00000024935 | 0.102 | 0.001 | -6.672425342 | 0.007828274 | 0.003051535 |
| ENSMUSG00000037541 | 0.102 | 0.001 | -6.672425342 | 0.004659503 | 0.001742006 |
| ENSMUSG00000051212 | 5.107666667 | 0.049666667 | -6.684242641 | 3.1041E-28 | 1.81094E-29 |
| ENSMUSG00000008540 | 12.24466667 | 0.118 | -6.697222831 | 3.95401E-20 | 3.30866E-21 |
| ENSMUSG00000022123 | 3.253333333 | 0.031 | -6.713506621 | 2.07851E-16 | 2.10677E-17 |
| ENSMUSG00000034224 | 0.108666667 | 0.001 | -6.763765654 | 0.03028883 | 0.013412908 |
| ENSMUSG00000014329 | 1.201333333 | 0.011 | -6.770989176 | 9.596E-12 | 1.32902E-12 |
| ENSMUSG00000028071 | 2.621666667 | 0.023333333 | -6.811948033 | 2.53731E-16 | 2.59617E-17 |
| ENSMUSG00000028836 | 0.112666667 | 0.001 | -6.815916936 | 0.038726467 | 0.01758269 |
| ENSMUSG00000039835 | 0.112666667 | 0.001 | -6.815916936 | 0.000983819 | 0.000323598 |
| ENSMUSG00000064147 | 77.82466667 | 0.667 | -6.866396921 | 0 | 0 |
| ENSMUSG00000118607 | 0.825 | 0.006666667 | -6.951284715 | 9.83604E-07 | 2.16632E-07 |
| ENSMUSG00000028626 | 0.125 | 0.001 | -6.965784285 | 0.011009149 | 0.004413085 |
| ENSMUSG00000026204 | 0.133333333 | 0.001 | -7.058893689 | 0.00311846 | 0.001126469 |
| ENSMUSG00000029718 | 3.402333333 | 0.024666667 | -7.107817911 | 1.78552E-09 | 3.00545E-10 |
| ENSMUSG00000049456 | 0.139333333 | 0.001 | -7.122396631 | 0.004913856 | 0.001843431 |
| ENSMUSG00000005364 | 0.147333333 | 0.001 | -7.202940059 | 0.037453322 | 0.016963117 |
| ENSMUSG00000053279 | 0.150333333 | 0.001 | -7.232021123 | 0.021062216 | 0.009004228 |
| ENSMUSG00000048230 | 0.150666667 | 0.001 | -7.235216462 | 0.007341095 | 0.002845916 |
| ENSMUSG00000036172 | 44.12833333 | 0.292 | -7.23959308 | 6.322E-124 | 7.7694E-126 |
| ENSMUSG00000043969 | 0.152333333 | 0.001 | -7.251087854 | 0.022655662 | 0.00976521 |
| ENSMUSG00000038349 | 0.153 | 0.001 | -7.257387843 | 1.74523E-05 | 4.48117E-06 |
| ENSMUSG00000047462 | 0.154333333 | 0.001 | -7.269905883 | 0.006061936 | 0.002307892 |
| ENSMUSG00000035177 | 0.156 | 0.001 | -7.285402219 | 0.001392309 | 0.000471697 |
| ENSMUSG00000050377 | 0.157333333 | 0.001 | -7.297680549 | 0.002864743 | 0.001027304 |
| ENSMUSG00000044734 | 108.7276667 | 0.651666667 | -7.382369176 | 3.26808E-31 | 1.73512E-32 |

| | | | | | |
|---|---|---|---|---|---|
| ENSMUSG00000078606 | 1.680333333 | 0.01 | -7.392603643 | 7.25899E-22 | 5.54798E-23 |
| ENSMUSG00000027072 | 3.540333333 | 0.021 | -7.397352063 | 3.95234E-08 | 7.4959E-09 |
| ENSMUSG00000024747 | 0.169333333 | 0.001 | -7.403722186 | 0.012579993 | 0.005096617 |
| ENSMUSG00000048126 | 2.368333333 | 0.013666667 | -7.43706693 | 4.15225E-32 | 2.15582E-33 |
| ENSMUSG00000097084 | 0.174666667 | 0.001 | -7.448460501 | 0.002727555 | 0.000976602 |
| ENSMUSG00000079162 | 0.179 | 0.001 | -7.483815777 | 0.008430881 | 0.003311959 |
| ENSMUSG00000030160 | 0.189 | 0.001 | -7.562242424 | 0.002286486 | 0.000805101 |
| ENSMUSG00000030154 | 4.986333333 | 0.025666667 | -7.601939574 | 1.79167E-11 | 2.54822E-12 |
| ENSMUSG00000000248 | 0.197333333 | 0.001 | -7.624490865 | 0.003489843 | 0.001272911 |
| ENSMUSG00000089953 | 0.200333333 | 0.001 | -7.64625868 | 0.00770788 | 0.002999283 |
| ENSMUSG00000053388 | 0.202666667 | 0.001 | -7.662965013 | 0.013920187 | 0.005709737 |
| ENSMUSG00000044165 | 2.003 | 0.009666667 | -7.694928211 | 7.64883E-06 | 1.87683E-06 |
| ENSMUSG00000024659 | 1000.327 | 4.642333333 | -7.751405853 | 8.2165E-158 | 7.3747E-160 |
| ENSMUSG00000007682 | 1.896 | 0.008666667 | -7.773266031 | 1.3771E-17 | 1.31111E-18 |
| ENSMUSG00000027360 | 114.2503333 | 0.518 | -7.785030561 | 9.43463E-36 | 4.37078E-37 |
| ENSMUSG00000062826 | 0.221 | 0.001 | -7.787902559 | 8.88411E-06 | 2.19895E-06 |
| ENSMUSG00000042182 | 0.224333333 | 0.001 | -7.809500194 | 0.001435888 | 0.000487473 |
| ENSMUSG00000002565 | 337.2646667 | 1.484333333 | -7.827922262 | 2.29664E-14 | 2.6385E-15 |
| ENSMUSG00000034391 | 0.228666667 | 0.001 | -7.837102265 | 0.041691903 | 0.019100635 |
| ENSMUSG00000022225 | 898.3516667 | 3.928333333 | -7.837219147 | 7.87824E-35 | 3.74222E-36 |
| ENSMUSG00000078670 | 0.231 | 0.001 | -7.851749041 | 0.000600978 | 0.000191156 |
| ENSMUSG00000032420 | 0.233 | 0.001 | -7.864186145 | 3.79962E-05 | 1.01995E-05 |
| ENSMUSG00000048992 | 0.234 | 0.001 | -7.87036472 | 0.02131189 | 0.009118323 |
| ENSMUSG00000056258 | 0.234333333 | 0.001 | -7.872418378 | 4.97891E-09 | 8.70038E-10 |
| ENSMUSG00000031075 | 0.241333333 | 0.001 | -7.914883386 | 0.000742002 | 0.000239547 |
| ENSMUSG00000056643 | 2.758333333 | 0.011 | -7.970149477 | 2.8735E-09 | 4.91019E-10 |
| ENSMUSG00000070780 | 1.591333333 | 0.006333333 | -7.973055338 | 7.59331E-09 | 1.34471E-09 |
| ENSMUSG00000027400 | 0.257333333 | 0.001 | -8.007494537 | 0.000561432 | 0.000177686 |
| ENSMUSG00000044052 | 0.257666667 | 0.001 | -8.009362103 | 0.005477285 | 0.002070054 |
| ENSMUSG00000025746 | 14.76766667 | 0.057 | -8.01726426 | 6.17065E-25 | 4.11973E-26 |
| ENSMUSG00000000782 | 0.267 | 0.001 | -8.060695932 | 0.00085606 | 0.000279207 |
| ENSMUSG00000032068 | 0.276666667 | 0.001 | -8.112005026 | 0.004294434 | 0.00159503 |

| | | | | | |
|---|---|---|---|---|---|
| ENSMUSG00000021999 | 0.294 | 0.001 | -8.199672345 | 0.004849926 | 0.001817215 |
| ENSMUSG00000055541 | 8.248 | 0.026333333 | -8.29101037 | 2.83891E-27 | 1.72483E-28 |
| ENSMUSG00000031775 | 0.320333333 | 0.001 | -8.32343012 | 0.049888225 | 0.023383814 |
| ENSMUSG00000055567 | 0.434666667 | 0.001333333 | -8.348728154 | 5.07233E-05 | 1.3903E-05 |
| ENSMUSG00000024899 | 17.65933333 | 0.053333333 | -8.371177665 | 1.05411E-55 | 2.93293E-57 |
| ENSMUSG00000028558 | 0.332 | 0.001 | -8.375039431 | 0.000533504 | 0.000168258 |
| ENSMUSG00000044556 | 0.337 | 0.001 | -8.396604781 | 0.045662261 | 0.021179168 |
| ENSMUSG00000030162 | 48.613 | 0.142 | -8.419307428 | 9.9778E-168 | 8.6111E-170 |
| ENSMUSG00000041857 | 0.344333333 | 0.001 | -8.427662038 | 0.016205088 | 0.006748763 |
| ENSMUSG00000081058 | 0.359 | 0.001 | -8.487840034 | 0.032206971 | 0.014362388 |
| ENSMUSG00000020383 | 0.384333333 | 0.001 | -8.586214297 | 0.006043145 | 0.002299199 |
| ENSMUSG00000094638 | 0.388333333 | 0.001 | -8.601151739 | 0.02399638 | 0.010401082 |
| ENSMUSG00000040627 | 0.39 | 0.001 | -8.607330314 | 0.000493539 | 0.000154768 |
| ENSMUSG00000052234 | 724.6346667 | 1.787333333 | -8.663301297 | 7.87346E-10 | 1.29267E-10 |
| ENSMUSG00000024681 | 397.6493333 | 0.934666667 | -8.732829093 | 8.76713E-11 | 1.31834E-11 |
| ENSMUSG00000001865 | 876.229 | 1.989333333 | -8.782879118 | 7.32952E-30 | 4.0787E-31 |
| ENSMUSG00000031074 | 0.448666667 | 0.001 | -8.809500194 | 5.41106E-05 | 1.48875E-05 |
| ENSMUSG00000009350 | 222.6543333 | 0.494666667 | -8.814133289 | 6.97997E-68 | 1.5903E-69 |
| ENSMUSG00000001864 | 0.471 | 0.001 | -8.87958325 | 8.42508E-07 | 1.83295E-07 |
| ENSMUSG00000018924 | 0.476333333 | 0.001 | -8.8958277 | 5.9781E-06 | 1.44623E-06 |
| ENSMUSG00000036295 | 12.521 | 0.025333333 | -8.949097156 | 1.29014E-39 | 5.28204E-41 |
| ENSMUSG00000040852 | 0.497666667 | 0.001 | -8.959035949 | 3.74398E-10 | 5.99957E-11 |
| ENSMUSG00000040118 | 1.331 | 0.002666667 | -8.963257357 | 4.13271E-12 | 5.56963E-13 |
| ENSMUSG00000021872 | 0.565 | 0.001 | -9.142107057 | 5.78955E-05 | 1.59848E-05 |
| ENSMUSG00000002324 | 0.583333333 | 0.001 | -9.188176706 | 2.52405E-06 | 5.83089E-07 |
| ENSMUSG00000079451 | 0.584666667 | 0.001 | -9.191470532 | 2.39689E-07 | 4.89295E-08 |
| ENSMUSG00000030785 | 17.59266667 | 0.029 | -9.244705565 | 3.46928E-13 | 4.31384E-14 |
| ENSMUSG00000041052 | 10.57633333 | 0.017333333 | -9.253074714 | 2.37058E-17 | 2.29464E-18 |
| ENSMUSG00000040314 | 293.3983333 | 0.471333333 | -9.281897246 | 5.27794E-18 | 4.89751E-19 |
| ENSMUSG00000026765 | 0.649666667 | 0.001 | -9.343555875 | 8.97884E-07 | 1.96637E-07 |
| ENSMUSG00000067813 | 0.657 | 0.001 | -9.35974956 | 3.96309E-06 | 9.41161E-07 |
| ENSMUSG00000090164 | 0.680666667 | 0.001 | -9.41080465 | 1.05137E-05 | 2.62333E-06 |

| | | | | | |
|---|---|---|---|---|---|
| ENSMUSG00000015970 | 0.687333333 | 0.001 | -9.424866117 | 4.01059E-10 | 6.45449E-11 |
| ENSMUSG00000091649 | 0.701666667 | 0.001 | -9.454642017 | 0.000127919 | 3.69256E-05 |
| ENSMUSG00000091898 | 0.702333333 | 0.001 | -9.456012098 | 0.003261859 | 0.001184349 |
| ENSMUSG00000102037 | 53.75966667 | 0.071 | -9.564489453 | 5.64915E-28 | 3.33863E-29 |
| ENSMUSG00000024680 | 309.9303333 | 0.402666667 | -9.588142196 | 8.21781E-26 | 5.24819E-27 |
| ENSMUSG00000089929 | 39.95833333 | 0.048666667 | -9.681346731 | 1.3798E-19 | 1.18794E-20 |
| ENSMUSG00000091455 | 0.849666667 | 0.001 | -9.730753157 | 1.22636E-13 | 1.47664E-14 |
| ENSMUSG00000107417 | 0.865 | 0.001 | -9.756556323 | 2.06886E-05 | 5.35642E-06 |
| ENSMUSG00000029811 | 0.974666667 | 0.001 | -9.928765095 | 8.65857E-09 | 1.54412E-09 |
| ENSMUSG00000028807 | 1.028666667 | 0.001 | -10.00655985 | 1.17828E-08 | 2.12894E-09 |
| ENSMUSG00000045502 | 1.093666667 | 0.001 | -10.09495738 | 2.92128E-08 | 5.48396E-09 |
| ENSMUSG00000005339 | 616.7256667 | 0.551 | -10.12836085 | 1.2624E-269 | 4.881E-272 |
| ENSMUSG00000029359 | 1.170666667 | 0.001 | -10.19311463 | 5.51879E-06 | 1.33131E-06 |
| ENSMUSG00000031594 | 1.220333333 | 0.001 | -10.25305956 | 2.31571E-06 | 5.32883E-07 |
| ENSMUSG00000056399 | 586.8093333 | 0.438666667 | -10.38555102 | 2.6367E-284 | 9.466E-287 |
| ENSMUSG00000069830 | 7.358333333 | 0.005333333 | -10.43012582 | 5.66943E-17 | 5.57392E-18 |
| ENSMUSG00000022157 | 776.0776667 | 0.505 | -10.58570194 | 3.3575E-236 | 1.7618E-238 |
| ENSMUSG00000054901 | 1.573 | 0.001 | -10.61930296 | 7.17635E-12 | 9.85485E-13 |
| ENSMUSG00000021108 | 1.604333333 | 0.001 | -10.64775821 | 2.22192E-12 | 2.92544E-13 |
| ENSMUSG00000018168 | 1.616666667 | 0.001 | -10.65880653 | 1.26119E-12 | 1.63091E-13 |
| ENSMUSG00000053475 | 1.670333333 | 0.001 | -10.70592032 | 1.94629E-09 | 3.28366E-10 |
| ENSMUSG00000001225 | 11.234 | 0.006666667 | -10.71861849 | 1.8571E-16 | 1.87967E-17 |
| ENSMUSG00000100838 | 1.812333333 | 0.001 | -10.82363261 | 4.27492E-06 | 1.02121E-06 |
| ENSMUSG00000094083 | 1.880333333 | 0.001 | -10.87677272 | 1.12857E-06 | 2.50742E-07 |
| ENSMUSG00000053820 | 2.025 | 0.001 | -10.98370619 | 3.92858E-05 | 1.05809E-05 |
| ENSMUSG00000038521 | 2.078333333 | 0.001 | -11.02121134 | 1.31125E-12 | 1.69655E-13 |
| ENSMUSG00000032035 | 20.30766667 | 0.007666667 | -11.37113741 | 9.74322E-25 | 6.55198E-26 |
| ENSMUSG00000030717 | 2.817666667 | 0.001 | -11.46028523 | 1.61493E-08 | 2.95469E-09 |
| ENSMUSG00000099974 | 3.055 | 0.001 | -11.57695666 | 3.73191E-09 | 6.44658E-10 |
| ENSMUSG00000024171 | 3.720333333 | 0.001 | -11.86121617 | 7.75081E-11 | 1.1607E-11 |
| ENSMUSG00000000869 | 6.52 | 0.001 | -12.67065625 | 9.81335E-11 | 1.48244E-11 |
| ENSMUSG00000035165 | 7.412666667 | 0.001 | -12.85577692 | 1.17699E-13 | 1.41557E-14 |
