# Supplementary material for: Mutations of RAS genes identified in acute myeloid leukemia affect glycerophospholipid metabolism pathway
Source: Front Oncol. 2023 Nov 14;13:1280192. doi: 10.3389/fonc.2023.1280192 (PMC10682766; doi:10.3389/fonc.2023.1280192)
Supplement: Supplementary file 5 [file DataSheet_5.pdf]

Common differential expressed genes

ENSEMBL	og2fc BaF3.vs.BaF3 KRAS(G12V	fdr BaF3.vs.BaF3 KRAS(G12V	value BaF3.vs.BaF3 KRAS(G12V	og2fc BaF3.vs.BaF3 NRAS(Q61K	fdr BaF3.vs.BaF3 NRAS(Q61K	value BaF3.vs.BaF3 NRAS(Q61K	exp_BaF3	exp_BaF3 KRAS(G12V)	exp_BaF3 NRAS(Q61K)
ENSMUSG00000000078	-1.618640163	1.01E-83	1.72E-85	-1.544909105	5.59E-68	1.27E-69	47.1253	15.346	16.1507
ENSMUSG00000000127	-3.726487076	0.038960609	0.017876681	-2.997134666	0.048941047	0.02286889	0.1677	0.0127	0.021
ENSMUSG00000000142	-2.113331946	1.31E-17	1.24E-18	-3.551660526	4.05E-24	2.82E-25	2.912	0.673	0.2483
ENSMUSG00000000182	11.30541036	1.55E-20	1.25E-21	11.58058161	1.92E-21	1.5E-22	0.0117	29.5267	35.7313
ENSMUSG00000000184	2.269869775	5.75E-31	2.97E-32	2.47446949	5.16E-110	7.34E-112	26.8673	129.5757	149.3187
ENSMUSG00000000244	5.022387612	1.12E-283	3.59E-286	5.016786755	3.16E-218	1.81E-220	2.242	72.866	72.5837
ENSMUSG00000000290	3.276806122	1.23E-294	3.68E-297	3.332061905	4.67E-205	2.97E-207	8.0627	78.144	81.195
ENSMUSG00000000308	4.632268215	0.026774794	0.011818481	4.73470962	0.017401181	0.007294944	0.0133	0.3307	0.355
ENSMUSG00000000320	7.691161905	0.000731483	0.000240457	8.271463028	0.000031	0.00000821	0	0.2067	0.309
ENSMUSG00000000386	4.574428338	4.25E-14	5.03E-15	5.9522559	3.04E-08	5.71E-09	0.084	2.0013	5.201
ENSMUSG00000000409	-3.555431892	1.87E-49	5.7E-51	-3.878351189	4.5E-78	9.1E-80	14.265	1.2133	0.97
ENSMUSG00000000552	1.028652227	1.38E-20	1.11E-21	1.31990282	1.35E-21	1.05E-22	11.6907	23.8503	29.1857
ENSMUSG00000000561	1.230943684	1.24E-26	7.53E-28	1.378072017	8.87E-43	3.38E-44	18.263	42.867	47.4693
ENSMUSG00000000562	2.134149858	3.55E-09	6.27E-10	3.003895258	1.21E-21	9.31E-23	0.7243	3.1797	5.8103
ENSMUSG00000000594	-3.247175522	0.000490678	0.000157071	-1.883019192	0.013385361	0.005459867	0.3197	0.0337	0.0867
ENSMUSG00000000631	1.060731285	9.38E-28	5.44E-29	1.02714042	5.55E-31	2.96E-32	12.5327	26.143	25.5413
ENSMUSG00000000632	-1.974448946	0.022809307	0.009950847	-3.379785955	6.34E-22	4.83E-23	3.1713	0.807	0.3047
ENSMUSG00000000686	-3.108855461	9.12E-62	2.2E-63	-3.346710672	1.39E-59	3.59E-61	13.622	1.579	1.339
ENSMUSG00000000706	-5.771122605	0.000582325	0.000188391	-3.034157011	0.016796279	0.007026281	0.3277	0.006	0.04
ENSMUSG00000000730	-2.326750609	0.00000226	0.000000532	-3.332062236	4.56E-12	6.17E-13	4.0953	0.8163	0.4067
ENSMUSG00000000782	-5.122096476	0.006414326	0.002490425	-8.060695932	0.00085606	0.000279207	0.267	0.0077	0
ENSMUSG00000000791	1.979584163	1.23E-24	8.17E-26	2.031936253	3.73E-33	1.87E-34	1.8503	7.2973	7.567
ENSMUSG00000000823	2.094315407	2.92E-86	4.81E-88	2.159596597	1.04E-69	2.31E-71	5.3373	22.7917	23.8467
ENSMUSG00000000869	-7.670656249	1.32E-08	2.46E-09	-12.67065625	9.81E-11	1.48E-11	6.52	0.032	0
ENSMUSG00000000915	-2.504799168	1.15E-151	9.63E-154	-2.550520766	1.98E-151	1.92E-153	20.3777	3.5903	3.4783
ENSMUSG00000001014	-1.347827899	0.00110821	0.000375998	-1.569218951	0.0000924	0.0000261	5.4987	2.1603	1.853
ENSMUSG00000001020	9.221817253	1.17E-15	1.25E-16	8.694842711	6.96E-31	3.74E-32	0.161	96.1323	66.7167
ENSMUSG00000001025	7.008046569	0.00000357	0.00000086	5.494037344	0.000295939	0.0000897	0.0293	3.7757	1.322
ENSMUSG00000001123	-1.244983323	1.55E-26	9.44E-28	-1.23015111	1.04E-25	6.69E-27	136.085	57.416	58.0093
ENSMUSG00000001211	4.065035724	1.05E-48	3.29E-50	3.879019413	1.47E-140	1.56E-142	5.18	86.7017	76.2133
ENSMUSG00000001225	-6.295712751	4.15E-46	1.37E-47	-10.71861849	1.86E-16	1.88E-17	11.234	0.143	0.0067
ENSMUSG00000001227	2.12017568	1.02E-32	4.98E-34	1.976093277	7.22E-38	3.1E-39	2.2717	9.876	8.9373
ENSMUSG00000001228	1.050480177	1.58E-60	3.88E-62	1.061775096	3.36E-53	9.99E-55	73.2013	151.616	152.8077
ENSMUSG00000001366	1.378230569	6.88E-55	1.87E-56	1.502821349	1.01E-63	2.47E-65	21.5877	56.117	61.1787
ENSMUSG00000001435	1.880093667	0.001875704	0.000663109	1.221587121	0.003306622	0.001201287	0.256	0.9423	0.597
ENSMUSG00000001441	-1.182801771	1.5E-41	5.72E-43	-1.257997294	7.12E-36	3.29E-37	47.576	20.957	19.8927
ENSMUSG00000001473	2.7136142	3.03E-74	5.96E-76	2.682292601	5.89E-96	9.71E-98	8.156	53.5003	52.3513
ENSMUSG00000001482	-1.115010854	1.13E-26	6.85E-28	-1.281355003	4.7E-22	3.56E-23	42.271	19.516	17.3907
ENSMUSG00000001542	-1.472993416	2.11E-57	5.54E-59	-1.288158393	9.11E-38	3.93E-39	45.3973	16.3537	18.589
ENSMUSG00000001552	-3.157108921	1.63E-39	6.61E-41	-3.017646482	9.05E-53	2.72E-54	8.5547	0.959	1.0563
ENSMUSG00000001583	-1.353636955	0.028837966	0.012823329	-1.221347369	0.008077673	0.003158792	1.0733	0.42	0.4603
ENSMUSG00000001741	1.232737874	1.6E-20	1.29E-21	1.129768874	1.31E-10	2.01E-11	5.1743	12.1603	11.3227
ENSMUSG00000001750	1.242387685	8.92E-28	5.17E-29	1.099535674	2.3E-20	1.89E-21	9.233	21.8443	19.785
ENSMUSG00000001751	-1.300023402	2.04E-26	1.25E-27	-1.387122351	1.32E-17	1.26E-18	20.132	8.176	7.697
ENSMUSG00000001755	-1.570983457	1.36E-37	5.88E-39	-1.594265702	4.69E-37	2.08E-38	29.8187	10.0363	9.8757
ENSMUSG00000001761	-1.14864743	0.0000589	0.0000166	-1.002161341	0.00063269	0.000201863	2.2267	1.0043	1.1117
ENSMUSG00000001864	-8.87958325	0.00000109	0.000000249	-8.87958325	0.000000843	0.000000183	0.471	0	0
ENSMUSG00000001865	-6.720489296	2.7E-14	3.14E-15	-8.782879118	7.33E-30	4.08E-31	876.229	8.309	1.9893
ENSMUSG00000001911	-1.462555772	9.89E-30	5.35E-31	-1.408392186	3.67E-13	4.57E-14	10.7097	3.886	4.0347
ENSMUSG00000001918	1.60539322	1.36E-55	3.66E-57	1.657199376	4.57E-42	1.77E-43	15.4877	47.1257	48.8487
ENSMUSG00000002032	-2.405744556	0.010022581	0.004055868	-1.287816105	0.027754502	0.012189063	1.3583	0.2563	0.5563
ENSMUSG00000002033	-3.792036533	0.008591545	0.003436021	-3.514068076	8.4E-12	1.16E-12	4.2157	0.3043	0.369
ENSMUSG00000002043	-1.117254824	1.23E-13	1.51E-14	-1.20071918	2.31E-15	2.5E-16	41.101	18.9463	17.8813
ENSMUSG00000002058	1.256563761	2.34E-31	1.2E-32	1.224211849	1.51E-23	1.08E-24	17.6223	42.1043	41.1707
ENSMUSG00000002076	9.539805035	0.000000999	0.000000226	8.279223644	0.000264192	0.0000796	0	0.7443	0.3107
ENSMUSG00000002104	2.608252235	2.46E-32	1.23E-33	2.762653216	2.05E-37	8.94E-39	2.0243	12.3437	13.738
ENSMUSG00000002107	1.237325402	5.93E-95	8.28E-97	1.238358699	2.16E-63	5.31E-65	25.456	60.0153	60.0583
ENSMUSG00000002204	-1.882137937	1.35E-30	7.07E-32	-2.383533663	2.6E-65	6.18E-67	33.5543	9.1027	6.4303
ENSMUSG00000002324	-9.188176706	0.00000333	0.000000798	-9.188176706	0.00000252	0.000000583	0.5833	0	0
ENSMUSG00000002365	7.622337858	5.01E-40	2E-41	7.84695944	6.08E-43	2.31E-44	0.0427	8.407	9.8233
ENSMUSG00000002565	-5.290830757	3.74E-08	7.24E-09	-7.827922262	2.3E-14	2.64E-15	337.2647	8.6153	1.4843
ENSMUSG00000002602	4.906890596	7.05E-19	6.25E-20	4.452223174	3.71E-20	3.1E-21	0.076	2.28	1.6637
ENSMUSG00000002603	1.416971741	9.68E-40	3.9E-41	1.787523002	3.98E-56	1.1E-57	69.9407	186.7587	241.4503
ENSMUSG00000002699	3.860751681	0	0	3.870311184	0	0	10.5953	153.9277	154.951
ENSMUSG00000002763	-1.20665918	9.17E-18	8.63E-19	-1.168064628	1.4E-22	1.04E-23	19.8913	8.6183	8.852
ENSMUSG00000002825	1.051448082	1.55E-09	2.62E-10	1.29085925	3.06E-14	3.55E-15	6.978	14.4627	17.0733
ENSMUSG00000002847	4.71332923	0.020457039	0.008826584	5.098733954	0.00457822	0.001710037	0.01	0.2623	0.3427
ENSMUSG00000002881	1.071797991	1.02E-43	3.62E-45	1.152249424	4.99E-42	1.93E-43	14.055	29.5443	31.2387
ENSMUSG00000002983	1.063608058	0.0000842	0.0000242	1.15707655	0.00086923	0.000283802	1.6417	3.4313	3.661
ENSMUSG00000002996	-1.260729662	6.32E-20	5.25E-21	-1.525472199	1.94E-32	9.88E-34	51.7053	21.5783	17.9607
ENSMUSG00000002997	1.74146642	1.29E-107	1.56E-109	2.042893796	2.36E-115	3.18E-117	31.7587	106.193	130.8683
ENSMUSG00000003051	-1.446537231	0.02052076	0.00885809	-1.559961321	0.001160823	0.000386781	1.7543	0.6437	0.595
ENSMUSG00000003123	-1.027166012	3.45E-08	6.66E-09	-1.152230437	2.68E-09	4.57E-10	6.1113	2.9987	2.7497
ENSMUSG00000003153	3.04504169	1.65E-275	5.51E-278	2.908251475	1.34E-258	5.55E-261	23.241	191.8243	174.472
ENSMUSG00000003206	3.96050725	5.37E-58	1.4E-59	3.739714627	3.8E-50	1.2E-51	1.219	18.9773	16.2843
ENSMUSG00000003344	-1.082975184	0.000000224	4.72E-08	-1.245060487	7.09E-13	8.99E-14	14.725	6.951	6.2123
ENSMUSG00000003348	1.566676254	1.05E-63	2.45E-65	1.61953301	4.87E-65	1.16E-66	10.0657	29.8167	30.9293
ENSMUSG00000003363	-1.848816641	2.77E-41	1.07E-42	-1.650239015	1.37E-43	5.08E-45	23.238	6.4513	7.4033
ENSMUSG00000003420	4.023846742	0.0000634	0.0000179	2.943416472	0.005478006	0.002070704	0.065	1.0573	0.5
ENSMUSG00000003458	-1.073275136	5.78E-41	2.26E-42	-1.043269717	4.22E-31	2.24E-32	43.287	20.5717	21.004
ENSMUSG00000003500	-1.586600861	3.61E-50	1.07E-51	-1.448466826	2.38E-19	2.07E-20	44.3473	14.7657	16.2493
ENSMUSG00000003526	1.12515558	1.83E-27	1.08E-28	1.106636884	5.52E-29	3.14E-30	12.2813	26.7887	26.447
ENSMUSG00000003617	-3.191096291	0.00000185	0.000000433	-6.010888316	0.00000423	0.00000101	0.7093	0.0777	0.011
ENSMUSG00000003762	-1.07844805	0.00000625	0.00000155	-1.341482455	1.63E-08	2.99E-09	5.3977	2.556	2.13
ENSMUSG00000003812	-1.296414684	1.53E-17	1.45E-18	-1.626511793	3.08E-26	1.93E-27	24.904	10.1393	8.0657
ENSMUSG00000003814	-3.208656779	0	0	-3.312245851	0	0	3052.664	330.2	307.322
ENSMUSG00000003849	-1.166734854	0.00000309	0.000000738	-1.165467804	0.00000336	0.00000079	6.8137	3.035	3.0377
ENSMUSG00000003863	-1.662436882	0.000000339	7.28E-08	-1.849645919	4.42E-09	7.7E-10	1.5173	0.4793	0.421
ENSMUSG00000004044	-1.547688549	1.97E-19	1.67E-20	-1.849286316	8.39E-32	4.41E-33	9.0297	3.0887	2.506
ENSMUSG00000004319	-5.379456326	1.49E-11	2.13E-12	-3.898274283	0.000000849	0.000000185	16.193	0.389	1.086
ENSMUSG00000004328	-1.797098753	1.1E-09	1.85E-10	-2.012552744	1.37E-11	1.93E-12	1.9623	0.5647	0.4863
ENSMUSG00000004446	1.062188663	1.7E-37	7.37E-39	1.196859787	6.92E-39	2.89E-40	21.9607	45.856	50.3427
ENSMUSG00000004451	1.373114007	5.27E-62	1.25E-63	1.319998819	2.89E-49	9.3E-51	23.4963	60.8623	58.6623
ENSMUSG00000004508	-1.271441805	1.63E-56	4.34E-58	-1.292341446	4.2E-21	3.35E-22	22.6597	9.3867	9.2517
ENSMUSG00000004530	1.246376886	7.65E-42	2.9E-43	1.302137707	1.3E-57	3.48E-59	23.5173	55.7937	57.9923
ENSMUSG00000004552	-3.093005878	1.19E-12	1.58E-13	-3.647646492	2.33E-25	1.52E-26	4.9547	0.5807	0.3953
ENSMUSG00000004562	-1.418107361	1.55E-17	1.47E-18	-1.83519507	7.55E-23	5.54E-24	9.2	3.4427	2.5783
ENSMUSG00000004612	-3.388170744	1.56E-17	1.48E-18	-4.202046153	1.07E-17	1.01E-18	7.9327	0.7577	0.431
ENSMUSG00000004667	1.202083147	2.28E-28	1.3E-29	1.267115305	7.6E-37	3.42E-38	34.665	79.7543	83.4317
ENSMUSG00000004771	1.077887156	4.65E-42	1.74E-43	1.146132866	2.04E-44	7.41E-46	45.8587	96.805	101.4943
ENSMUSG00000004788	-1.077042112	1.25E-29	6.83E-31	-1.097153125	1.03E-30	5.54E-32	52.7813	25.0183	24.672
ENSMUSG00000004864	-1.529774112	5.99E-08	1.19E-08	-1.965751569	1.35E-15	1.44E-16	8.7277	3.0227	2.2343
ENSMUSG00000004931	-1.066999978	5.27E-14	6.28E-15	-1.089301518	4.39E-14	5.13E-15	21.853	10.4307	10.2707
ENSMUSG00000004952	-1.833455773	3.52E-10	5.65E-11	-2.013019586	3.21E-16	3.3E-17	4.304	1.2077	1.0663
ENSMUSG00000004988	-4.434304964	0.000067	0.000019	-4.914150759	0.000109296	0.0000313	1.8593	0.086	0.0617
ENSMUSG00000005057	1.163087571	0.0000668	0.000019	1.273812129	0.0000361	0.00000966	1.504	3.368	3.6367
ENSMUSG00000005107	1.185680537	1.39E-15	1.49E-16	1.131846222	6.6E-17	6.52E-18	3.3527	7.6263	7.347
ENSMUSG00000005125	5.406523692	0	0	5.201664573	0	0	12.084	512.5503	444.7007
ENSMUSG00000005131	7.839203788	0.011832545	0.004852206	7.348728154	0.020469679	0.008733956	0	0.229	0.163
ENSMUSG00000005339	-9.378881745	3.2E-51	9.27E-53	-10.12836085	1.26E-269	4.88E-272	616.7257	0.9263	0.551
ENSMUSG00000005609	2.141329376	7.44E-237	3.1E-239	1.944620184	3.77E-92	6.46E-94	39.4793	174.1703	151.9703
ENSMUSG00000005610	1.202746169	2.21E-72	4.47E-74	1.200632164	4.75E-87	8.6E-89	257.5393	592.7973	591.9293
ENSMUSG00000005611	8.422703777	2.22E-155	1.68E-157	8.516782138	0	0	0.6383	219.0453	233.8053
ENSMUSG00000005682	-1.017196227	4.95E-11	7.42E-12	-1.101841033	5.98E-15	6.63E-16	9.4243	4.6563	4.391
ENSMUSG00000005686	2.51171198	1.73E-68	3.59E-70	2.198470499	2.48E-36	1.13E-37	2.1703	12.3773	9.9617
ENSMUSG00000005824	5.699044866	1.64E-271	5.59E-274	5.771035945	1.98E-290	6.71E-293	1.592	82.704	86.9357
ENSMUSG00000005893	-1.148484952	2.08E-44	7.23E-46	-1.090385269	6.09E-26	3.87E-27	21.4343	9.669	10.0663
ENSMUSG00000005917	-1.647440668	0.000000607	0.000000134	-1.516815477	0.00000204	0.000000467	4.2313	1.3507	1.4787
ENSMUSG00000005949	-1.030379116	2.01E-09	3.46E-10	-1.091422227	3.45E-17	3.37E-18	16.845	8.247	7.9053
ENSMUSG00000005968	1.454741166	1.56E-09	2.65E-10	1.014615942	3.83E-09	6.62E-10	2.5207	6.9093	5.0927
ENSMUSG00000006262	1.480146362	5.27E-42	1.99E-43	1.642883511	7.37E-103	1.15E-104	11.118	31.0167	34.7203
ENSMUSG00000006301	1.036004561	0.000000104	2.11E-08	1.254835135	2.01E-10	3.13E-11	2.1567	4.4223	5.1467
ENSMUSG00000006344	8.193771743	0.000000065	1.29E-08	8.061416199	0.000000428	9.01E-08	0.005	1.464	1.3357
ENSMUSG00000006362	1.460719864	3.58E-90	5.45E-92	1.595702516	1.68E-67	3.87E-69	27.7473	76.3733	83.864
ENSMUSG00000006389	8.865472095	0	0	8.898601387	0	0	0.9	419.774	429.525
ENSMUSG00000006445	5.640128294	0.00000385	0.000000932	5.118606224	0.0000618	0.0000171	0.0103	0.5153	0.359
ENSMUSG00000006457	-2.684968797	0.0000125	0.00000321	-2.602637393	0.000000147	2.95E-08	1.314	0.2043	0.2163
ENSMUSG00000006463	-1.648463675	1.01E-29	5.47E-31	-1.657616614	1.14E-30	6.17E-32	17.6803	5.6397	5.604
ENSMUSG00000006464	-1.564902069	6.98E-12	9.78E-13	-1.600786604	1.32E-10	2.03E-11	2.0473	0.692	0.675
ENSMUSG00000006494	2.679980508	3.29E-65	7.45E-67	2.460516649	2.25E-73	4.84E-75	2.3763	15.2287	13.0797
ENSMUSG00000006522	9.734145075	0.00000105	0.000000238	9.123259229	0.000000147	2.95E-08	0	0.8517	0.5577
ENSMUSG00000006567	-1.813192325	0.0000212	0.00000559	-1.777509673	0.00000715	0.00000175	0.7017	0.1997	0.2047
ENSMUSG00000006587	1.813077906	0.02525626	0.011106087	2.143715122	0.002460978	0.000872893	0.2037	0.7157	0.9
ENSMUSG00000006641	1.763800822	6.36E-17	6.28E-18	1.803345661	9.77E-16	1.03E-16	3.02	10.2557	10.5407
ENSMUSG00000006732	-1.599790072	1.22E-08	2.25E-09	-1.278266464	0.00000119	0.000000266	6.4873	2.1403	2.6747
ENSMUSG00000006850	-1.553708581	9E-20	7.56E-21	-1.710958385	4.84E-30	2.67E-31	19.5587	6.6623	5.9743
ENSMUSG00000007021	-2.42716202	0.0000606	0.0000171	-3.673023457	9.76E-08	1.93E-08	1.4073	0.2617	0.1103
ENSMUSG00000007030	3.020248684	1.82E-08	3.43E-09	3.143054137	2.15E-10	3.36E-11	0.0943	0.7653	0.8333
ENSMUSG00000007035	4.416778833	5.29E-08	1.04E-08	4.991991883	3.93E-10	6.32E-11	0.207	4.4213	6.5873
ENSMUSG00000007036	1.139295511	5.73E-24	3.92E-25	1.162674177	3.98E-45	1.42E-46	23.0647	50.8053	51.6353
ENSMUSG00000007041	1.060912856	6.3E-76	1.19E-77	1.132745778	5.12E-65	1.22E-66	159.422	332.5943	349.5737
ENSMUSG00000007216	-2.012646543	0.0000139	0.0000036	-2.057458556	0.0000638	0.0000177	1.4513	0.3597	0.3487
ENSMUSG00000007379	4.991637814	6.24E-74	1.23E-75	5.028132148	4.28E-84	7.96E-86	0.2037	6.4797	6.6457
ENSMUSG00000007480	-3.835724125	0.001688909	0.000592262	-5.158600011	6.4E-102	1.01E-103	10.8703	0.7613	0.3043
ENSMUSG00000007613	1.054194172	8.84E-44	3.14E-45	1.178551946	1.87E-48	6.08E-50	13.9067	28.878	31.4777

ENSMUSG00000007659	4.372945927	0	0	4.513326954	0	0	4.664	96.6377	106.5137
ENSMUSG00000007682	-5.531191244	8.02E-31	4.16E-32	-7.773266031	1.38E-17	1.31E-18	1.896	0.041	0.0087
ENSMUSG00000007837	-1.3538979	0.001093377	0.000370738	-1.186118171	0.006814334	0.002626182	2.867	1.1217	1.26
ENSMUSG00000008129	-3.018425474	0.00000464	0.00000113	-4.154446269	0.000000191	3.87E-08	1.0507	0.1297	0.059
ENSMUSG00000008167	-1.559430395	6.67E-12	9.33E-13	-1.497242689	3.92E-13	4.88E-14	8.4757	2.8757	3.0023
ENSMUSG00000008305	-1.355935179	2.65E-43	9.58E-45	-1.240898512	2.83E-25	1.86E-26	41.4873	16.2083	17.5537
ENSMUSG00000008318	1.887196991	0.000000138	2.84E-08	1.048629919	0.000025	0.00000656	3.0093	11.132	6.225
ENSMUSG00000008393	-1.11582521	1.7E-16	1.73E-17	-1.138371341	3.38E-20	2.8E-21	20.7313	9.566	9.4177
ENSMUSG00000008450	1.149505095	0.0000057	0.00000141	1.2058266	0.000000991	0.000000218	10.9153	24.2143	25.1783
ENSMUSG00000008540	-6.916900867	1.99E-17	1.9E-18	-6.697222831	3.95E-20	3.31E-21	12.2447	0.1013	0.118
ENSMUSG00000009145	-1.341164809	0.00000015	3.09E-08	-1.677747309	0.000000028	5.25E-09	3.259	1.2863	1.0187
ENSMUSG00000009350	-5.093789915	1.34E-08	2.5E-09	-8.814133289	6.98E-68	1.59E-69	222.6543	6.52	0.4947
ENSMUSG00000009687	1.666789632	2.4E-102	3.07E-104	1.651818995	5.55E-81	1.08E-82	79.9953	253.991	251.369
ENSMUSG00000009739	-2.949183466	2.91E-08	5.57E-09	-2.438493728	5.16E-08	9.9E-09	0.9577	0.124	0.1767
ENSMUSG00000009772	7.095020192	0.000000171	3.54E-08	7.423466121	1.38E-08	2.51E-09	0.007	0.957	1.2017
ENSMUSG00000010154	-2.509155861	1.44E-49	4.34E-51	-2.21152992	5.73E-36	2.64E-37	12.7463	2.239	2.752
ENSMUSG00000010406	1.050029396	0.00000104	0.000000236	1.24554753	1.48E-08	2.7E-09	13.315	27.5697	31.571
ENSMUSG00000011148	-1.466947112	0.001877319	0.000663941	-1.945119529	8.57E-36	3.96E-37	18.9737	6.8637	4.9273
ENSMUSG00000012117	-1.168215998	1.25E-29	6.81E-31	-1.103369564	3.65E-27	2.22E-28	35.2937	15.7047	16.4267
ENSMUSG00000013089	4.057520285	2.48E-220	1.15E-222	4.152387086	3.59E-193	2.53E-195	2.207	36.7483	39.246
ENSMUSG00000013155	-1.360486869	3.64E-14	4.28E-15	-1.32216574	1.6E-14	1.82E-15	17.202	6.6993	6.8797
ENSMUSG00000013611	-1.595536367	0.00000323	0.000000772	-1.418238301	0.0000168	0.0000043	3.0583	1.012	1.1443
ENSMUSG00000013663	-1.082746247	4.07E-49	1.26E-50	-1.229922144	4.71E-62	1.18E-63	63.7347	30.091	27.1727
ENSMUSG00000013707	1.91588416	2.06E-14	2.39E-15	1.981094381	1.74E-16	1.76E-17	3.1237	11.787	12.332
ENSMUSG00000014226	1.223747356	1.82E-45	6.19E-47	1.13339579	2.52E-32	1.29E-33	27.519	64.2713	60.3697
ENSMUSG00000014329	-5.060495794	1.05E-14	1.2E-15	-6.770989176	9.6E-12	1.33E-12	1.2013	0.036	0.011
ENSMUSG00000014496	-2.637475506	2.39E-183	1.43E-185	-2.757122635	1.44E-168	1.23E-170	104.323	16.7657	15.4313
ENSMUSG00000014599	-5.097787681	7.82E-09	1.43E-09	-5.895977386	1.06E-63	2.6E-65	64.927	1.896	1.0903
ENSMUSG00000014606	1.990632093	5.52E-190	3.22E-192	1.981423667	4.83E-147	4.8E-149	46.905	186.4057	185.2197
ENSMUSG00000014873	1.063078265	6.75E-14	8.1E-15	1.062306258	6.06E-14	7.19E-15	9.2447	19.3157	19.3053
ENSMUSG00000014905	-3.375274888	7.99E-260	2.94E-262	-3.079033437	1.97E-249	9.38E-252	77.6177	7.48	9.185
ENSMUSG00000015016	-1.917669744	2.1E-39	8.56E-41	-1.802256714	6.56E-24	4.62E-25	17.4197	4.6107	4.9947
ENSMUSG00000015363	-1.282654657	1.78E-40	7.03E-42	-1.227873533	1.7E-38	7.21E-40	60.8297	25.0033	25.971
ENSMUSG00000015437	-5.396837143	2.6E-09	4.51E-10	-5.458302632	0.001634846	0.000560525	94.951	2.2537	2.1597
ENSMUSG00000015533	-1.229246092	0.028485063	0.012646616	-1.372371034	0.005570903	0.002107358	0.422	0.18	0.163
ENSMUSG00000015599	-1.066348231	1.3E-14	1.49E-15	-1.116208745	0.00000054	0.000000115	2.959	1.413	1.365
ENSMUSG00000015702	1.651036276	9.21E-12	1.3E-12	1.490067061	5.18E-10	8.39E-11	2.0177	6.3367	5.6677
ENSMUSG00000015714	1.590167882	4.17E-75	8E-77	1.558977723	3.92E-126	4.65E-128	52.8103	159.0037	155.603
ENSMUSG00000015745	1.745025017	3.89E-31	2E-32	1.953945055	2.79E-41	1.1E-42	10.8057	36.2207	41.8647
ENSMUSG00000015837	-2.172374313	3.56E-154	2.82E-156	-2.194339424	2.49E-145	2.58E-147	410.896	91.1553	89.778
ENSMUSG00000015970	-9.424866117	4.77E-10	7.75E-11	-9.424866117	4.01E-10	6.45E-11	0.6873	0	0
ENSMUSG00000016206	-1.345686967	4.35E-08	8.5E-09	-1.314201653	1.78E-10	2.77E-11	11.1733	4.3963	4.4933
ENSMUSG00000016427	-1.217633011	0.000121883	0.0000359	-1.59340531	2.48E-08	4.62E-09	21.251	9.1377	7.0423
ENSMUSG00000016477	1.421640234	3.89E-44	1.36E-45	1.409628586	7.4E-41	2.95E-42	5.5227	14.7947	14.672
ENSMUSG00000016496	1.295202985	6.97E-29	3.92E-30	1.086513433	5.55E-14	6.57E-15	12.6077	30.9407	26.7737
ENSMUSG00000016526	-1.341255723	1.03E-12	1.35E-13	-1.593355549	1.51E-14	1.71E-15	8.792	3.47	2.9137
ENSMUSG00000016528	-1.164113401	1.62E-64	3.72E-66	-1.137635636	4.75E-64	1.15E-65	137.6013	61.403	62.5403
ENSMUSG00000016534	-1.126858108	2.48E-19	2.13E-20	-1.275451554	8.09E-32	4.25E-33	71.5443	32.761	29.5547
ENSMUSG00000016552	2.191318462	0.000647563	0.000210981	2.010569242	0.001423304	0.000482689	0.0793	0.3623	0.3197
ENSMUSG00000017167	-1.501188637	9.83E-09	1.81E-09	-1.420416013	1.26E-09	2.09E-10	2.6873	0.9493	1.004
ENSMUSG00000017195	-8.098032083	0.014550206	0.006083897	-4.098032083	0.039031777	0.017745392	0.274	0	0.016
ENSMUSG00000017417	-2.352740592	0.000524967	0.00016885	-3.330020516	2.01E-12	2.64E-13	1.609	0.315	0.16
ENSMUSG00000017561	1.169810534	2.47E-48	7.82E-50	1.050770891	1.55E-26	9.66E-28	30.343	68.2663	62.8597
ENSMUSG00000017718	-1.191216974	0.00000851	0.00000214	-1.109137898	0.0000182	0.00000468	5.0967	2.232	2.3627
ENSMUSG00000017724	9.425565605	0.000000441	0.000000096	9.533978572	8.51E-08	1.67E-08	0	0.6877	0.7413
ENSMUSG00000017737	5.470920058	1.22E-10	1.89E-11	4.826573608	5.43E-23	3.95E-24	0.0843	3.7403	2.393
ENSMUSG00000017747	-1.016615795	0.013273846	0.005493961	-1.081699203	0.002885477	0.001036732	5.1523	2.5467	2.4343
ENSMUSG00000017776	1.283928414	3.54E-52	9.99E-54	1.306439635	1.24E-44	4.46E-46	16.8003	40.909	41.5523
ENSMUSG00000017897	10.13570929	3.1E-09	5.43E-10	9.489178962	9.74E-08	1.92E-08	0	1.125	0.7187
ENSMUSG00000017950	-3.283520033	0.000000921	0.000000207	-5.918235569	4.85E-09	8.46E-10	0.766	0.0787	0.0127
ENSMUSG00000018008	1.071015343	8.98E-29	5.08E-30	1.004968031	1.9E-20	1.56E-21	15.7993	33.193	31.7077
ENSMUSG00000018102	-4.675312972	7.92E-22	5.96E-23	-3.416702318	5.53E-23	4.03E-24	7.4183	0.2903	0.6947
ENSMUSG00000018166	2.127023047	2.85E-14	3.33E-15	2.169410439	1.63E-90	2.84E-92	8.932	39.0163	40.1797
ENSMUSG00000018168	-5.135244575	0.005139719	0.001959127	-10.65880653	1.26E-12	1.63E-13	1.6167	0.046	0
ENSMUSG00000018169	1.776133937	3.68E-11	5.47E-12	1.479349649	4.94E-08	9.45E-09	1.5237	5.2187	4.2483
ENSMUSG00000018199	1.969558359	2.38E-94	3.38E-96	1.969366364	6.56E-69	1.47E-70	2.5583	10.0197	10.0183
ENSMUSG00000018293	1.45116882	2.21E-67	4.74E-69	1.418325627	1.48E-103	2.28E-105	560.1133	1531.515	1497.0437
ENSMUSG00000018377	1.316494383	9.58E-84	1.62E-85	1.359222094	2.99E-105	4.54E-107	33.4107	83.2127	85.714
ENSMUSG00000018378	1.423782335	1.57E-80	2.77E-82	1.600590175	9.87E-68	2.26E-69	17.0577	45.7637	51.7303
ENSMUSG00000018425	-1.042324526	4.87E-29	2.71E-30	-1.135829623	3.41E-32	1.76E-33	26.7027	12.9653	12.1517
ENSMUSG00000018427	-1.312487851	6.22E-16	6.46E-17	-1.83728595	7.61E-13	9.66E-14	9.5457	3.8433	2.6713
ENSMUSG00000018474	-1.52679762	1.58E-33	7.58E-35	-1.327160475	2.57E-76	5.22E-78	81.9467	28.4393	32.66
ENSMUSG00000018500	6.896421451	8.08E-10	1.34E-10	6.823741869	2.36E-09	4E-10	0.0227	2.7003	2.5677
ENSMUSG00000018654	-1.245211537	0.022514346	0.009815911	-2.050982646	0.00000046	9.71E-08	2.6977	1.138	0.651
ENSMUSG00000018740	-2.014434843	0.0000194	0.0000051	-1.686093176	0.000260329	0.0000784	2.1763	0.5387	0.6763
ENSMUSG00000018796	-2.513225891	2.31E-207	1.17E-209	-2.56438812	1.48E-155	1.36E-157	58.7147	10.2847	9.9263
ENSMUSG00000018841	-1.246388232	1.45E-25	9.15E-27	-1.350696884	7.88E-26	5.03E-27	22.404	9.4433	8.7847
ENSMUSG00000018909	2.152337888	4.02E-119	4.41E-121	2.196031894	1.19E-118	1.54E-120	4.955	22.0273	22.7047
ENSMUSG00000018931	-1.338014321	7.61E-17	7.57E-18	-1.387507785	1.29E-21	9.97E-23	9.695	3.835	3.7057
ENSMUSG00000019027	-3.444784843	0.004450086	0.001678018	-2.859822342	0.011160675	0.004478448	0.0653	0.006	0.009
ENSMUSG00000019102	1.319613099	0.001081195	0.000366232	1.691554404	0.0000316	0.00000837	0.9563	2.387	3.089
ENSMUSG00000019158	-1.176684007	1.65E-11	2.38E-12	-1.352744789	1.57E-16	1.58E-17	28.9187	12.7927	11.323
ENSMUSG00000019301	-3.31937239	9.8E-13	1.28E-13	-3.033880893	8.96E-11	1.35E-11	4.136	0.4143	0.505
ENSMUSG00000019528	-1.159623085	4.76E-26	2.96E-27	-1.408449691	5.62E-50	1.78E-51	81.353	36.416	30.647
ENSMUSG00000019842	-1.251133578	0.00000584	0.00000144	-1.581830583	1.88E-12	2.47E-13	5.3683	2.2553	1.7933
ENSMUSG00000019850	-1.951144907	3.14E-19	2.72E-20	-1.920902993	3.66E-20	3.05E-21	5.2937	1.369	1.398
ENSMUSG00000019866	1.410607927	4.5E-28	2.58E-29	1.476767158	4.42E-30	2.43E-31	2.3513	6.251	6.5443
ENSMUSG00000019916	1.27583116	3.27E-23	2.31E-24	1.051408961	1.07E-24	7.27E-26	16.7923	40.6607	34.803
ENSMUSG00000019920	1.8154751	1.08E-151	8.97E-154	1.767783438	4.79E-64	1.16E-65	21.2433	74.7713	72.34
ENSMUSG00000019947	-3.643992932	0.000326125	0.000101519	-5.519602069	1.09E-53	3.19E-55	3.517	0.2813	0.0767
ENSMUSG00000019960	3.378189906	6.52E-150	5.52E-152	3.451142331	1.52E-246	7.54E-249	7.791	81.0083	85.21
ENSMUSG00000019977	-1.137126877	2.63E-39	1.07E-40	-1.134931186	2.15E-39	8.86E-41	58.7247	26.7	26.7407
ENSMUSG00000019982	-4.258957792	6.09E-15	6.83E-16	-5.024414095	0	0	316.9017	16.552	9.737
ENSMUSG00000019986	-1.41843718	4.71E-25	3.06E-26	-1.553946816	5.92E-30	3.28E-31	10.952	4.0973	3.73
ENSMUSG00000020009	-4.734854843	0	0	-4.945978083	0	0	219.3943	8.2393	7.1177
ENSMUSG00000020014	1.107888903	1.25E-13	1.53E-14	1.012546001	4.24E-15	4.67E-16	1.393	3.0023	2.8103
ENSMUSG00000020029	3.766046747	0	0	3.909181815	0	0	18.036	245.3767	270.97
ENSMUSG00000020048	-1.071084904	3.16E-63	7.41E-65	-1.034191773	3.19E-43	1.2E-44	831.2237	395.63	405.8777
ENSMUSG00000020057	-1.932947583	0.00439754	0.001656371	-2.56003556	0.000188716	0.0000558	0.7077	0.1853	0.12
ENSMUSG00000020077	-2.329087675	1.58E-09	2.7E-10	-2.392879461	1.49E-22	1.11E-23	928.756	184.8317	176.837
ENSMUSG00000020097	-1.120694777	4.54E-20	3.74E-21	-1.146335638	2.77E-32	1.42E-33	29.132	13.397	13.161
ENSMUSG00000020101	2.91315674	0.009252529	0.003720294	2.769320967	0.002925439	0.001052302	0.097	0.7307	0.6613
ENSMUSG00000020134	1.821408789	1.97E-91	2.9E-93	1.653853962	6.31E-44	2.32E-45	8.26	29.193	25.992
ENSMUSG00000020142	1.136829406	1.48E-19	1.25E-20	1.069675506	4.11E-15	4.52E-16	6.0327	13.2657	12.6623
ENSMUSG00000020178	1.481798789	1.32E-31	6.71E-33	1.742132613	2.42E-42	9.29E-44	8.3707	23.379	28.0023
ENSMUSG00000020189	-1.085926927	0.00011169	0.0000327	-1.443828382	4.72E-86	8.61E-88	45.3757	21.376	16.6797
ENSMUSG00000020191	-2.454595765	0.000027	0.00000723	-2.265458425	0.0000903	0.0000255	1.787	0.326	0.3717
ENSMUSG00000020227	-1.460776807	5.12E-63	1.2E-64	-1.584170456	4.78E-37	2.12E-38	63.1273	22.934	21.054
ENSMUSG00000020255	1.657468108	4.01E-201	2.14E-203	1.47496998	1.55E-75	3.23E-77	25.0823	79.1253	69.7233
ENSMUSG00000020256	-2.455927178	3.66E-09	6.47E-10	-2.616258631	5.48E-11	8.12E-12	1.165	0.2123	0.19
ENSMUSG00000020279	-1.381087145	7.18E-08	1.44E-08	-1.693568185	2.69E-12	3.57E-13	3.7003	1.4207	1.144
ENSMUSG00000020284	-1.093678135	0.000129713	0.0000383	-1.161627667	0.0000482	0.0000132	4.6233	2.1663	2.0667
ENSMUSG00000020323	-7.098458937	2.16E-22	1.58E-23	-6.285544489	2.22E-22	1.67E-23	6.7607	0.0493	0.0867
ENSMUSG00000020383	-8.586214297	0.007792239	0.003079545	-8.586214297	0.006043145	0.002299199	0.3843	0	0
ENSMUSG00000020393	-1.193868764	6.6E-17	6.53E-18	-1.145917709	1.39E-11	1.95E-12	7.807	3.4127	3.528
ENSMUSG00000020395	-2.529358248	0.000177962	0.0000534	-3.980465002	0.0000421	0.0000114	0.4157	0.072	0.0263
ENSMUSG00000020407	3.851406959	4.85E-14	5.78E-15	3.960789131	4.68E-17	4.59E-18	0.3287	4.744	5.1177
ENSMUSG00000020432	-1.166798074	9.72E-16	1.03E-16	-1.409152718	7.27E-32	3.79E-33	42.516	18.937	16.0087
ENSMUSG00000020447	7.324930583	0.000290522	0.0000898	6.785724906	0.002057629	0.000718977	0	0.1603	0.1103
ENSMUSG00000020462	-1.155576976	3.5E-13	4.44E-14	-1.563899401	4.99E-35	2.36E-36	28.1727	12.6463	9.529
ENSMUSG00000020527	1.936403572	2.34E-38	9.8E-40	2.069300518	7.44E-39	3.13E-40	2.63	10.0663	11.0377
ENSMUSG00000020534	1.396658119	1.5E-56	3.97E-58	1.443206399	4.49E-51	1.4E-52	16.293	42.898	44.3047
ENSMUSG00000020570	1.226738562	6.91E-83	1.19E-84	1.280411149	8.19E-65	1.97E-66	45.6137	106.753	110.7993
ENSMUSG00000020571	-1.700773707	3.62E-183	2.19E-185	-1.779212025	1.01E-126	1.18E-128	438.4897	134.8887	127.7507
ENSMUSG00000020576	-1.664454874	5.99E-75	1.16E-76	-1.741642564	1.41E-89	2.48E-91	19.4307	6.1297	5.8103
ENSMUSG00000020577	-1.06601837	1.06E-09	1.78E-10	-1.047840587	3.55E-10	5.68E-11	16.1267	7.7027	7.8003
ENSMUSG00000020590	-1.312032687	4.67E-29	2.6E-30	-1.621972016	2.42E-46	8.37E-48	13.464	5.4227	4.3743
ENSMUSG00000020599	4.781942503	0.000412081	0.000130537	5.861845113	0.00000295	0.000000689	0.015	0.4127	0.8723
ENSMUSG00000020604	1.846306938	0.000167378	0.0000501	2.081598219	8.64E-08	0.000000017	0.348	1.2513	1.473
ENSMUSG00000020620	-5.922832139	0.014867073	0.006225683	-3.922832139	0.038931912	0.017691925	0.0607	0	0.004
ENSMUSG00000020629	-1.486222877	4.07E-12	5.59E-13	-1.358508579	4.3E-13	5.38E-14	17.1613	6.1257	6.6927
ENSMUSG00000020644	1.036532299	0.036111422	0.016453964	1.111578387	0.000994716	0.000327451	1.8717	3.8393	4.0443
ENSMUSG00000020653	-2.096369572	1.04E-21	7.86E-23	-1.996604371	2.08E-27	1.26E-28	5.3753	1.257	1.347
ENSMUSG00000020659	-1.38415227	6.4E-36	2.89E-37	-1.22946554	2.54E-20	2.09E-21	21.3557	8.1817	9.1077
ENSMUSG00000020668	-1.170916076	1.79E-10	2.81E-11	-1.204913201	3.95E-10	6.35E-11	7.7663	3.4493	3.369
ENSMUSG00000020669	-2.061670411	1.65E-09	2.81E-10	-2.384580265	8.36E-15	9.33E-16	5.114	1.225	0.9793
ENSMUSG00000020689	6.593772424	9.16E-166	6.43E-168	6.820592879	0	0	1.6757	161.8493	189.4047
ENSMUSG00000020715	-1.170944614	3.04E-21	2.36E-22	-1.182774494	6.37E-20	5.39E-21	22.0573	9.7963	9.7163
ENSMUSG00000020732	-1.628143739	1.16E-33	5.52E-35	-1.619774325	2.13E-28	1.23E-29	26.0337	8.422	8.471
ENSMUSG00000020733	-1.070866737	9.53E-31	4.96E-32	-1.196402088	2.81E-45	9.95E-47	82.21	39.1347	35.8733
ENSMUSG00000020743	-1.204943297	9.7E-13	1.27E-13	-1.402516482	5.19E-16	5.41E-17	17.0937	7.415	6.466
ENSMUSG00000020787	3.937630471	4.21E-213	2.05E-215	4.011618572	2.52E-178	1.97E-180	2.5147	38.5323	40.56
ENSMUSG00000020788	1.723270062	1.69E-216	8.11E-219	1.843224488	2.81E-139	3.03E-141	49.502	163.4477	177.6187
ENSMUSG00000020798	-1.623492955	8.02E-46	2.69E-47	-1.638425625	1.74E-36	7.89E-38	32.3167	10.4883	10.3803
ENSMUSG00000020818	-1.301537544	2.23E-20	1.82E-21	-1.43359819	5.63E-18	5.23E-19	13.6293	5.5293	5.0457
ENSMUSG00000020821	-1.094230467	2.37E-37	1.03E-38	-1.122342936	1.23E-27	7.34E-29	23.6023	11.055	10.8417
ENSMUSG00000020823	-1.035891346	5.47E-27	3.27E-28	-1.033491721	2.46E-20	2.02E-21	21.76	10.6127	10.6303

ENSMUSG00000020836	-2.945681089	0.000000962	0.000000217	-2.373774741	0.000000785	0.00000017	1.5203	0.1973	0.2933
ENSMUSG00000020838	6.306995998	1.28E-08	2.38E-09	7.000445908	0.00000155	0.000000352	0.0927	7.337	11.865
ENSMUSG00000020873	-1.305059323	6.02E-32	3.04E-33	-1.261628207	1.05E-30	5.66E-32	58.7243	23.766	24.4923
ENSMUSG00000020877	-1.383812221	1.28E-08	2.39E-09	-1.231214435	0.000000113	2.24E-08	6.9623	2.668	2.9657
ENSMUSG00000020901	1.219314971	3.56E-11	5.28E-12	1.367629489	1.02E-16	1.02E-17	1.5867	3.6943	4.0943
ENSMUSG00000020902	1.656001838	1.85E-27	1.09E-28	2.167563246	1.04E-58	2.73E-60	1.902	5.994	8.545
ENSMUSG00000020919	-1.129475926	5.45E-44	1.93E-45	-1.287728583	3.01E-51	9.34E-53	33.9677	15.526	13.913
ENSMUSG00000020961	-6.76244518	2.11E-40	8.37E-42	-6.3223167	4.27E-13	5.34E-14	12.3767	0.114	0.1547
ENSMUSG00000020964	-1.669451266	8.43E-95	1.18E-96	-1.649835168	6.23E-105	9.55E-107	58.5547	18.408	18.66
ENSMUSG00000020986	-1.341793365	7.41E-39	3.08E-40	-1.4073324	8.06E-37	3.63E-38	23.5707	9.2993	8.8863
ENSMUSG00000020990	4.450933198	0.000000374	8.09E-08	4.001012773	0.0000121	0.00000305	0.0593	1.2977	0.95
ENSMUSG00000021000	-2.436289495	0	0	-2.589609843	1.51E-187	1.13E-189	206.2533	38.107	34.265
ENSMUSG00000021007	-2.137907131	5.22E-11	7.86E-12	-1.954322426	1.98E-14	2.27E-15	5.2433	1.1913	1.353
ENSMUSG00000021024	-1.022614517	5.89E-41	2.31E-42	-1.025663212	2.59E-35	1.21E-36	191.839	94.4277	94.2283
ENSMUSG00000021027	-1.723521693	9.83E-129	9.56E-131	-1.696968384	5.14E-117	6.74E-119	43.7933	13.261	13.5073
ENSMUSG00000021036	-1.088161749	7.94E-35	3.71E-36	-1.161489189	5.36E-35	2.54E-36	27.0133	12.706	12.0763
ENSMUSG00000021065	-2.577109609	2.41E-102	3.1E-104	-2.401396963	8.17E-131	9.31E-133	206.7893	34.653	39.1413
ENSMUSG00000021066	-1.173331603	0.001645971	0.00057629	-1.091611074	0.002672599	0.000955307	0.8127	0.3603	0.3813
ENSMUSG00000021068	-1.382746696	1.57E-114	1.77E-116	-1.446964896	4.06E-55	1.15E-56	64.228	24.6307	23.5583
ENSMUSG00000021071	4.751645489	0.000808019	0.00026803	4.177193002	0.005068926	0.001905922	0.011	0.2963	0.199
ENSMUSG00000021108	-8.232720708	4.73E-10	7.68E-11	-10.64775821	2.22E-12	2.93E-13	1.6043	0.0053	0
ENSMUSG00000021109	-2.119069295	9.05E-240	3.64E-242	-2.055383521	3.08E-188	2.25E-190	138.423	31.8643	33.7673
ENSMUSG00000021120	-1.150678287	4.93E-13	6.33E-14	-1.085219428	4.43E-14	5.18E-15	14.11	6.3553	6.6503
ENSMUSG00000021123	-1.386709639	0.0000302	0.00000814	-1.179183282	0.001575268	0.000538793	4.1297	1.5793	1.8237
ENSMUSG00000021127	-1.631104015	1.08E-39	4.35E-41	-1.46460309	8.31E-31	4.48E-32	18.0027	5.812	6.523
ENSMUSG00000021140	-1.554455964	4.34E-42	1.62E-43	-1.282553857	8.36E-38	3.59E-39	9.9513	3.388	4.0907
ENSMUSG00000021176	-1.095263066	0.000772842	0.000255341	-1.276525612	0.000102618	0.0000293	7.6423	3.577	3.1547
ENSMUSG00000021182	-1.009033543	1.48E-34	6.93E-36	-1.230915424	8.17E-18	7.63E-19	22.4287	11.1443	9.5557
ENSMUSG00000021190	4.533646286	3.34E-191	1.9E-193	4.877879423	3.36E-214	1.97E-216	2.6137	60.536	76.849
ENSMUSG00000021226	-2.260783551	5.06E-40	2.02E-41	-2.185937162	4.99E-35	2.35E-36	13.6203	2.842	2.9933
ENSMUSG00000021234	-1.211664138	0.008144134	0.003237852	-1.091792476	0.008388433	0.003293635	1.2653	0.5463	0.5937
ENSMUSG00000021236	-1.665862244	2.38E-64	5.49E-66	-1.982329728	5.41E-62	1.36E-63	23.289	7.3397	5.894
ENSMUSG00000021238	-1.038327759	7.06E-12	9.9E-13	-1.230712548	1.14E-15	1.21E-16	10.2987	5.0143	4.3883
ENSMUSG00000021240	-1.693028584	6.07E-12	8.43E-13	-1.617778658	3.92E-16	4.05E-17	7.5887	2.347	2.4727
ENSMUSG00000021250	2.410327999	5.58E-16	5.78E-17	3.865589875	1.03E-57	2.75E-59	1.2977	6.8983	18.9157
ENSMUSG00000021257	-2.185496953	2.79E-41	1.08E-42	-2.053841636	2.06E-39	8.49E-41	7.871	1.7303	1.8957
ENSMUSG00000021266	-1.147701528	1.09E-25	6.86E-27	-1.009360839	2.26E-16	2.31E-17	75.3003	33.9863	37.4067
ENSMUSG00000021271	-1.754969127	8.91E-10	1.48E-10	-2.154660994	1.12E-13	1.35E-14	3.6823	1.091	0.827
ENSMUSG00000021280	5.428836853	3.96E-78	7.29E-80	5.139457479	2.92E-67	6.78E-69	0.3997	17.2163	14.0873
ENSMUSG00000021298	-2.90449608	0.00000563	0.00000139	-1.713197428	0.003211461	0.001164055	1.0033	0.134	0.306
ENSMUSG00000021322	1.857980995	0.000466252	0.000148928	1.784777074	0.00151268	0.000516028	0.2453	0.8893	0.8453
ENSMUSG00000021366	-1.007801415	1.11E-33	5.28E-35	-1.111772093	2.34E-21	1.84E-22	11.5583	5.748	5.3483
ENSMUSG00000021384	1.425896767	0.000000132	2.72E-08	1.216625268	0.0000121	0.00000303	2.614	7.0233	6.075
ENSMUSG00000021420	-1.130334202	6.97E-11	1.06E-11	-1.03599615	5.82E-13	7.34E-14	14.988	6.8467	7.3093
ENSMUSG00000021453	4.175358182	1E-14	1.14E-15	3.45450879	3.74E-08	7.07E-09	0.2847	5.1433	3.1207
ENSMUSG00000021514	-1.17508462	6.9E-13	8.94E-14	-1.223427725	6.91E-12	9.48E-13	14.8693	6.585	6.368
ENSMUSG00000021549	1.130292606	1.22E-39	4.93E-41	1.749946592	1.27E-40	5.1E-42	11.4143	24.9863	38.3917
ENSMUSG00000021572	-1.48795631	0.0000281	0.00000755	-1.510713894	0.000132682	0.0000384	5.1377	1.8317	1.803
ENSMUSG00000021573	-4.465380885	0.027388875	0.012119982	-4.339850003	0.023939824	0.010373263	0.081	0.0037	0.004
ENSMUSG00000021591	1.367299	1.2E-24	7.92E-26	1.329391822	2.29E-30	1.25E-31	27.1583	70.065	68.248
ENSMUSG00000021608	1.574213454	4.63E-65	1.05E-66	1.54683196	7.79E-54	2.28E-55	9.6697	28.7937	28.2523
ENSMUSG00000021614	-1.625010661	0.000041	0.0000113	-1.666202349	8.07E-11	1.21E-11	0.694	0.225	0.2187
ENSMUSG00000021624	3.512558296	0.000285935	0.0000883	4.774467235	1.53E-10	2.37E-11	0.0687	0.7837	1.8793
ENSMUSG00000021661	-1.297202639	1.96E-13	2.44E-14	-1.287201644	1.71E-15	1.84E-16	15.4267	6.2773	6.321
ENSMUSG00000021662	-8.51438531	0.00000021	0.000000044	-6.398908092	0.00000629	0.00000153	0.3657	0	0.0043
ENSMUSG00000021665	-1.881556819	4.6E-67	1E-68	-1.922957323	6.83E-83	1.29E-84	79.7153	21.634	21.022
ENSMUSG00000021675	5.386005073	4.66E-89	7.26E-91	6.044765964	7.82E-93	1.33E-94	0.3527	14.7473	23.282
ENSMUSG00000021676	-1.268804466	6.98E-33	3.42E-34	-1.441347809	1.95E-58	5.14E-60	38.6487	16.0393	14.2313
ENSMUSG00000021697	1.311562018	2.67E-50	7.93E-52	1.206122041	7.59E-31	4.09E-32	14.4797	35.94	33.407
ENSMUSG00000021733	1.051863371	2.53E-36	1.13E-37	1.31893357	1.84E-46	6.34E-48	5.7873	11.9983	14.4383
ENSMUSG00000021738	-1.0631824	5.06E-17	4.97E-18	-1.01529628	8.49E-11	1.27E-11	10.0507	4.81	4.9723
ENSMUSG00000021823	2.351734546	2.03E-117	2.26E-119	2.400934536	1.41E-236	7.29E-239	29.0233	148.146	153.2853
ENSMUSG00000021872	-5.517616192	0.000818311	0.000271785	-9.142107057	0.0000579	0.000016	0.565	0.0123	0
ENSMUSG00000021876	2.838995377	1.82E-09	3.12E-10	2.7238744	3.12E-09	5.34E-10	0.393	2.812	2.5963
ENSMUSG00000021884	-1.283599309	1.78E-13	2.21E-14	-1.24939376	7.31E-09	1.29E-09	13.495	5.5433	5.6763
ENSMUSG00000021900	-1.209973162	5.87E-17	5.78E-18	-1.257142629	1.25E-18	1.12E-19	13.88	6	5.807
ENSMUSG00000021904	-1.458653508	0.001374618	0.000473167	-1.749682156	0.000170269	0.0000499	0.7723	0.281	0.2297
ENSMUSG00000021929	1.206071535	2.65E-69	5.45E-71	1.229700143	2.55E-55	7.12E-57	18.173	41.9267	42.619
ENSMUSG00000021930	1.275802134	1.72E-13	2.13E-14	1.385164508	6.38E-17	6.3E-18	3.678	8.9057	9.607
ENSMUSG00000021948	1.516703567	4.44E-87	7.24E-89	1.641737246	2.16E-112	3.02E-114	21.44	61.3477	66.9017
ENSMUSG00000021951	1.428321832	6.33E-16	6.58E-17	1.571284254	3.61E-20	3.01E-21	8.3013	22.3417	24.669
ENSMUSG00000021958	1.026995256	0.00000449	0.0000011	1.090261063	0.000000283	5.83E-08	4.262	8.685	9.0743
ENSMUSG00000021959	-1.768827842	1.24E-11	1.77E-12	-1.592198232	1.73E-08	3.17E-09	4.0643	1.1927	1.348
ENSMUSG00000021996	1.013120839	8.9E-36	4.04E-37	1.127190327	9.28E-35	4.42E-36	67.242	135.7127	146.8787
ENSMUSG00000022014	2.620919609	4.57E-12	6.3E-13	2.599304415	9.73E-25	6.54E-26	1.0677	6.5677	6.47
ENSMUSG00000022020	1.71170289	4.89E-31	2.52E-32	1.504734104	2.52E-25	1.64E-26	2.6827	8.787	7.6127
ENSMUSG00000022022	1.458133138	1.94E-09	3.32E-10	1.438615662	4.08E-09	7.07E-10	2.6273	7.2187	7.1217
ENSMUSG00000022037	2.416507674	1.77E-199	9.59E-202	2.519490668	1.79E-167	1.56E-169	34.267	182.9437	196.48
ENSMUSG00000022041	4.08912589	0.001473353	0.000510838	4.691709313	0.0000174	0.00000447	0.017	0.2893	0.4393
ENSMUSG00000022051	1.361594615	8.98E-37	3.96E-38	1.250435104	3E-32	1.54E-33	25.343	65.1237	60.2943
ENSMUSG00000022053	11.52698821	2.65E-17	2.56E-18	10.28771238	4.79E-13	6.02E-14	0	2.951	1.25
ENSMUSG00000022055	6.491853096	0.026191085	0.011544455	6.448460501	0.020360778	0.008676244	0	0.09	0.0873
ENSMUSG00000022075	1.304628495	7.77E-19	6.93E-20	1.486274336	7.99E-25	5.35E-26	1.8233	4.504	5.1083
ENSMUSG00000022089	1.498188379	6.72E-52	1.91E-53	1.351783266	5.14E-28	3.02E-29	13.9617	39.44	35.634
ENSMUSG00000022094	1.917805601	5.2E-75	1E-76	2.102877881	7.74E-82	1.48E-83	4.1727	15.7663	17.9243
ENSMUSG00000022102	6.278829442	0	0	6.307745095	0	0	2.7387	212.645	216.95
ENSMUSG00000022106	1.705659926	1.76E-08	3.33E-09	1.697976598	1.32E-10	2.02E-11	1.924	6.2757	6.2423
ENSMUSG00000022123	-3.251257238	0.019519367	0.008390819	-6.715306621	2.08E-16	2.11E-17	3.2533	0.3417	0.031
ENSMUSG00000022148	2.585068237	7.61E-93	1.11E-94	2.635735873	1.99E-290	6.88E-293	37.8993	227.4127	235.5413
ENSMUSG00000022157	-10.05474777	1.25E-299	3.64E-302	-10.58570194	3.36E-236	1.76E-238	776.0777	0.7297	0.505
ENSMUSG00000022215	9.508455064	0.000204355	0.0000618	9.121533517	0.001051872	0.0003483	0	0.7283	0.557
ENSMUSG00000022216	1.016105524	3.89E-37	1.7E-38	1.062336951	7.43E-39	3.12E-40	39.8627	80.6203	83.2457
ENSMUSG00000022219	7.544855109	9.96E-09	1.83E-09	7.261828821	4.81E-08	9.21E-09	0.0177	3.299	2.7113
ENSMUSG00000022221	-3.463702056	0.003613698	0.001340117	-4.987264012	0.002421039	0.000857159	0.6767	0.0613	0.0213
ENSMUSG00000022225	-7.500117851	1.77E-10	2.78E-11	-7.837219147	7.88E-35	3.74E-36	898.3517	4.9623	3.9283
ENSMUSG00000022244	-1.393955749	0.00000435	0.00000106	-1.151143303	0.000213539	0.0000637	3.2307	1.2293	1.4547
ENSMUSG00000022253	-1.618777478	2.22E-73	4.4E-75	-1.700654672	5.06E-55	1.44E-56	57.6127	18.7593	17.7243
ENSMUSG00000022255	1.644842261	3.74E-149	3.22E-151	1.753402241	3.6E-184	2.71E-186	63.2417	197.7653	213.221
ENSMUSG00000022272	-1.462626958	0.03013852	0.013481404	-2.21136972	0.00987656	0.003920212	0.1883	0.0683	0.0407
ENSMUSG00000022299	1.114576868	1.28E-12	1.7E-13	1.154117662	6.83E-19	6.09E-20	5.795	12.548	12.8967
ENSMUSG00000022307	1.348720132	1.46E-37	6.3E-39	1.210390056	2.79E-16	2.85E-17	27.2153	69.3137	62.9763
ENSMUSG00000022309	1.412734895	3.44E-132	3.28E-134	1.325812795	9.58E-39	4.03E-40	64.978	172.9983	162.883
ENSMUSG00000022359	-1.293963433	5.46E-11	8.23E-12	-1.300414082	3.21E-12	4.29E-13	12.0917	4.9313	4.9093
ENSMUSG00000022394	-1.281203101	9.46E-53	2.65E-54	-1.210434387	3.85E-39	1.61E-40	35.6437	14.6657	15.403
ENSMUSG00000022438	4.885554971	4.42E-80	7.86E-82	5.715609768	1.51E-144	1.59E-146	0.4027	11.9027	21.16
ENSMUSG00000022442	-1.013824617	6.91E-10	1.14E-10	-1.175473193	3.61E-12	4.85E-13	16.218	8.0317	7.1803
ENSMUSG00000022453	2.944098666	0.0000112	0.00000285	2.926896976	0.0000023	0.000000529	0.1973	1.5187	1.5007
ENSMUSG00000022456	2.518343789	6.46E-27	3.88E-28	2.113744787	1.03E-21	7.93E-23	0.6623	3.7947	2.8667
ENSMUSG00000022475	-3.266331715	0	0	-3.822405317	1.4E-282	5.13E-285	57.8793	6.0153	4.0913
ENSMUSG00000022558	-1.945227772	8.56E-163	6.3E-165	-2.190179045	2.73E-126	3.22E-128	51.0487	13.256	11.186
ENSMUSG00000022583	13.08015131	7.73E-13	1E-13	13.68211676	2.23E-09	3.79E-10	0	8.66	13.144
ENSMUSG00000022623	8.52563674	4.33E-88	6.95E-90	8.105621803	4.82E-76	9.91E-78	0.0457	16.8287	12.5787
ENSMUSG00000022636	-4.331043082	0.002159275	0.000771759	-3.232862688	0.005109069	0.001922074	0.4763	0.0237	0.0507
ENSMUSG00000022637	-1.732057399	1.23E-108	1.48E-110	-1.731118897	1.17E-31	6.18E-33	44.2437	13.3183	13.327
ENSMUSG00000022659	10.74511405	2.17E-10	3.43E-11	10.7984718	2.17E-10	3.39E-11	0	1.7163	1.781
ENSMUSG00000022742	-1.300468415	1.1E-42	4.07E-44	-1.294840941	6.63E-44	2.44E-45	37.8133	15.352	15.412
ENSMUSG00000022769	-1.798866412	4.75E-49	1.48E-50	-1.840485347	5.37E-69	1.2E-70	105.8837	30.431	29.5657
ENSMUSG00000022780	-2.411195433	0.003718459	0.001383024	-2.217591435	0.003827764	0.001408059	0.3333	0.0627	0.0717
ENSMUSG00000022791	1.788868363	2.2E-55	5.93E-57	1.79617648	3.33E-47	1.13E-48	15.899	54.938	55.217
ENSMUSG00000022824	-2.749235095	3.79E-31	1.94E-32	-3.013707164	1.24E-11	1.74E-12	13.501	2.008	1.6717
ENSMUSG00000022836	6.970210117	5.27E-67	1.15E-68	6.853829352	1.47E-65	3.48E-67	0.046	5.7677	5.3207
ENSMUSG00000022844	-1.967468182	7.3E-27	4.39E-28	-1.838284407	5.98E-25	3.99E-26	11.9123	3.046	3.3313
ENSMUSG00000022848	-1.259777995	0.000960012	0.000322249	-1.782484777	0.00000269	0.000000624	2.2717	0.9487	0.6603
ENSMUSG00000022861	-1.00296059	3.11E-10	4.97E-11	-1.201318423	5.73E-16	5.99E-17	9.756	4.868	4.2427
ENSMUSG00000022885	-5.914883386	0.019481743	0.008370586	-5.121334264	0.020531565	0.008763196	0.6033	0.01	0.0173
ENSMUSG00000022895	1.064074498	7.5E-37	3.3E-38	1.218649379	4.15E-48	1.37E-49	11.9823	25.053	27.8863
ENSMUSG00000022911	-1.370747273	3.2E-21	2.49E-22	-1.220021778	3.21E-18	2.95E-19	14.1207	5.4603	6.0617
ENSMUSG00000022951	-1.260941588	9.53E-15	1.08E-15	-1.435494392	1.39E-12	1.81E-13	15.2467	6.362	5.637
ENSMUSG00000022952	-2.534440688	3.45E-93	5.01E-95	-2.596908303	7.4E-63	1.84E-64	104.8183	18.0923	17.3257
ENSMUSG00000022957	3.183416907	0.00000192	0.000000449	2.874672601	8.99E-21	7.26E-22	0.4297	3.9033	3.1513
ENSMUSG00000022964	-1.135080915	0.000000194	4.03E-08	-1.377791485	2.5E-21	1.97E-22	14.9547	6.809	5.7547
ENSMUSG00000022995	-2.261994695	3.17E-33	1.54E-34	-2.357000321	2.07E-47	7.01E-49	7.9527	1.658	1.5523
ENSMUSG00000023031	1.193707228	0.00000538	0.00000132	1.788682294	1.51E-14	1.72E-15	3.055	6.988	10.555
ENSMUSG00000023032	-2.550340115	0.000000178	0.000000037	-3.079226691	9.51E-08	1.87E-08	0.9607	0.164	0.1137
ENSMUSG00000023036	1.385179451	0.000362007	0.000113669	1.693971448	0.000000188	0.000000038	1.2897	3.3687	4.1727
ENSMUSG00000023045	-2.013417142	3.33E-39	1.36E-40	-1.996558728	6.51E-40	2.65E-41	14.6557	3.63	3.6727
ENSMUSG00000023087	1.26780847	9.75E-99	1.3E-100	1.147740324	8.93E-49	2.9E-50	422.473	1017.296	936.059
ENSMUSG00000023088	1.535473376	6.29E-114	7.21E-116	1.367826184	9.69E-79	1.95E-80	13.3083	38.5787	34.3463
ENSMUSG00000023259	1.379257171	5.65E-12	7.83E-13	1.125483911	6.62E-08	1.28E-08	2.133	5.5487	4.6537
ENSMUSG00000023272	-1.884358024	5.19E-62	1.23E-63	-1.801586966	9.86E-63	2.46E-64	112.7047	30.5277	32.3303
ENSMUSG00000023277	1.155322405	1.56E-32	7.72E-34	1.044348807	2.74E-23	1.98E-24	21.5477	47.994	44.4407
ENSMUSG00000023333	9.1275645	0.00000475	0.00000116	8.822305264	0.0000244	0.00000638	0	0.5593	0.4527
ENSMUSG00000023411	-4.045376213	3.76E-19	3.28E-20	-5.476715524	8.19E-17	8.1E-18	1.9593	0.1187	0.044
ENSMUSG00000023467	-1.440332133	9.98E-13	1.31E-13	-1.559800955	1.45E-14	1.64E-15	14.5697	5.3687	4.942
ENSMUSG00000023805	1.167039978	4.75E-22	3.54E-23	1.312686185	5.25E-27	3.23E-28	3.059	6.869	7.5987
ENSMUSG00000023828	-1.931577283	0.001835351	0.000647441	-2.44818157	9.05E-73	1.96E-74	31.8923	8.3603	5.844
ENSMUSG00000023883	-1.406936816	2.22E-49	6.78E-51	-1.395422507	3.66E-36	1.68E-37	113.839	42.93	43.274

ENSMUSG00000023905	1.182091371	0.000205448	0.0000622	1.672036737	2.53E-09	4.31E-10	2.4753	5.6167	7.888
ENSMUSG00000023908	-1.202250539	1.42E-20	1.14E-21	-1.045311754	9.61E-11	1.45E-11	20.9037	9.0847	10.1287
ENSMUSG00000023952	-1.437106863	3.93E-49	1.22E-50	-1.390491787	1.94E-42	7.44E-44	51.8927	19.1643	19.7937
ENSMUSG00000023972	-8.154818109	0.00000516	0.00000127	-3.382228605	0.000711792	0.000229057	0.285	0	0.0273
ENSMUSG00000023993	12.05776614	5.44E-12	7.53E-13	13.05171876	9.63E-11	1.45E-11	0	4.2633	8.491
ENSMUSG00000024006	-1.057090429	2.08E-41	7.98E-43	-1.241568515	6.31E-40	2.57E-41	47.5953	22.8743	20.1287
ENSMUSG00000024008	-2.984963089	6.55E-10	1.08E-10	-4.295303209	3.18E-18	2.92E-19	1.1453	0.1447	0.0583
ENSMUSG00000024034	8.930737338	0.00000185	0.000000433	9.026062297	0.000000961	0.000000212	0	0.488	0.5213
ENSMUSG00000024042	4.07909178	2.16E-177	1.38E-179	3.830933375	6.93E-79	1.38E-80	0.932	15.7523	13.263
ENSMUSG00000024063	-2.082979315	1.06E-163	7.59E-166	-2.146413785	1.48E-59	3.84E-61	70.5203	16.6447	15.9287
ENSMUSG00000024070	-6.165898347	2.77E-49	8.52E-51	-5.181433326	1.16E-182	8.89E-185	21.2287	0.2957	0.585
ENSMUSG00000024114	-1.908762591	0.00000124	0.000000286	-1.847119841	0.000000208	4.22E-08	3.7273	0.9927	1.036
ENSMUSG00000024127	-1.067655438	3.62E-21	2.82E-22	-1.128409469	2.47E-28	1.43E-29	27.3797	13.0627	12.524
ENSMUSG00000024171	-6.434951419	1.19E-08	2.21E-09	-11.86121617	7.75E-11	1.16E-11	3.7203	0.043	0
ENSMUSG00000024187	-1.47338962	1.19E-08	2.21E-09	-1.959510802	7.01E-19	6.26E-20	7.6943	2.771	1.9783
ENSMUSG00000024206	-1.873069682	3.14E-32	1.57E-33	-1.907879251	2.23E-34	1.08E-35	9.7317	2.6567	2.5933
ENSMUSG00000024220	-1.627918066	8.95E-26	5.62E-27	-1.584251376	1.02E-16	1.01E-17	16.226	5.25	5.4113
ENSMUSG00000024236	1.999965171	9.52E-60	2.36E-61	2.008530963	1.66E-177	1.32E-179	10.3557	41.4217	41.6683
ENSMUSG00000024238	1.163669286	4.27E-27	2.55E-28	1.254265057	8.41E-37	3.8E-38	9.3647	20.9793	22.339
ENSMUSG00000024268	-1.010588635	0.000584795	0.000189271	-1.046092989	0.00184975	0.000640593	1.8233	0.905	0.883
ENSMUSG00000024300	2.419739453	1.06E-15	1.13E-16	1.768197451	2.91E-16	2.99E-17	1.0083	5.3953	3.4347
ENSMUSG00000024308	-1.37304788	5.31E-38	2.24E-39	-1.298465585	3.39E-47	1.15E-48	40.569	15.6627	16.4937
ENSMUSG00000024334	-1.789055274	1.39E-27	8.14E-29	-2.45018565	3.31E-53	9.79E-55	30.2517	8.7537	5.5357
ENSMUSG00000024352	-1.616292206	0.0000686	0.0000195	-1.91530097	0.000000541	0.000000115	9.0463	2.9507	2.3983
ENSMUSG00000024371	1.513261035	0.000408798	0.000129412	1.582712391	0.000199992	0.0000594	0.6417	1.8317	1.922
ENSMUSG00000024381	1.98933418	4.13E-37	1.81E-38	1.918463189	5.41E-33	2.73E-34	3.8353	15.2283	14.4983
ENSMUSG00000024399	3.051026494	4.62E-43	1.69E-44	2.952746977	9.51E-70	2.1E-71	3.7267	30.8867	28.8527
ENSMUSG00000024401	4.456797824	1.09E-30	5.69E-32	5.19957907	1.91E-49	6.09E-51	0.3157	6.932	11.6
ENSMUSG00000024402	4.799039654	0.000480715	0.000153749	4.695968222	0.000435205	0.000135543	0.029	0.8073	0.7517
ENSMUSG00000024424	-4.874680081	1.4E-62	3.3E-64	-5.576758915	7E-64	1.71E-65	12.9183	0.4403	0.2707
ENSMUSG00000024427	4.085729874	3.03E-14	3.55E-15	4.051514159	2.43E-15	2.64E-16	0.0653	1.1093	1.0833
ENSMUSG00000024456	1.203464438	1.19E-88	1.88E-90	1.344531301	6.59E-68	1.5E-69	35.0543	80.7273	89.0197
ENSMUSG00000024457	-1.084745623	1.98E-14	2.29E-15	-1.203654825	8.28E-16	8.71E-17	12.0013	5.6583	5.2107
ENSMUSG00000024480	-2.60821007	3.49E-26	2.16E-27	-2.865253134	0	0	259.8293	42.6127	35.6583
ENSMUSG00000024579	-1.035097957	4.05E-14	4.78E-15	-1.088697346	4.37E-12	5.9E-13	21.483	10.4833	10.101
ENSMUSG00000024589	1.328560554	4.62E-43	1.68E-44	1.633174099	3.46E-58	9.12E-60	15.3217	38.4807	47.527
ENSMUSG00000024594	-1.981607321	6.41E-89	1.01E-90	-2.088666426	6.81E-108	1E-109	50.7647	12.854	11.9347
ENSMUSG00000024614	-1.21543685	2.22E-20	1.81E-21	-1.001360033	0.002316977	0.000817917	32.8997	14.168	16.4343
ENSMUSG00000024642	-1.09601864	2.38E-42	8.84E-44	-1.118165936	1.15E-32	5.86E-34	40.1313	18.7737	18.4877
ENSMUSG00000024644	-2.202890073	6.38E-61	1.56E-62	-1.800937599	0.00000307	0.000000718	36.077	7.836	10.3537
ENSMUSG00000024646	-1.455044379	5.68E-14	6.8E-15	-1.163319936	0.01839888	0.007761472	40.9877	14.95	18.3003
ENSMUSG00000024659	-7.411339734	7.85E-14	9.46E-15	-7.751405853	8.22E-158	7.37E-160	1000.327	5.8763	4.6423
ENSMUSG00000024666	-1.078959943	1.55E-09	2.64E-10	-1.288960203	4.83E-14	5.68E-15	18.8697	8.9323	7.7223
ENSMUSG00000024667	-1.46615953	1.91E-24	1.27E-25	-1.789204335	9.6E-35	4.59E-36	31.5417	11.4163	9.126
ENSMUSG00000024679	4.810610979	7.97E-34	3.78E-35	4.663449187	1.31E-26	8.15E-28	0.3267	9.1673	8.2783
ENSMUSG00000024680	-8.090145004	8.79E-46	2.95E-47	-9.588142196	8.22E-26	5.25E-27	309.9303	1.1373	0.4027
ENSMUSG00000024681	-4.548427676	0.0000884	0.0000255	-8.732829093	8.77E-11	1.32E-11	397.6493	16.9937	0.9347
ENSMUSG00000024691	1.092172735	6.72E-86	1.12E-87	1.088504884	6.5E-35	3.08E-36	94.274	200.9873	200.477
ENSMUSG00000024747	-7.403722186	0.01553558	0.006532606	-7.403722186	0.012579993	0.005096617	0.1693	0	0
ENSMUSG00000024781	-1.014529687	2.89E-08	5.51E-09	-1.238942234	1.25E-30	6.76E-32	29.8383	14.7697	12.642
ENSMUSG00000024807	-2.088672519	1.67E-115	1.87E-117	-1.867326812	5.5E-43	2.08E-44	81.8287	19.2377	22.4277
ENSMUSG00000024827	-4.402098444	0.000206735	0.0000626	-2.462219436	0.003890866	0.001434495	0.3453	0.0163	0.0627
ENSMUSG00000024842	-3.648845865	0.006457124	0.002511527	-2.923285034	0.013969967	0.00573112	0.531	0.0423	0.07
ENSMUSG00000024856	-1.621525779	1.32E-51	3.78E-53	-1.768951055	5.86E-46	2.04E-47	66.2663	21.536	19.444
ENSMUSG00000024873	-1.94988584	0.00000515	0.00000126	-1.642222198	0.000038	0.0000102	3.2723	0.847	1.0483
ENSMUSG00000024885	4.278633826	0.000000146	0.00000003	3.797507136	0.000000243	4.96E-08	0.084	1.6303	1.168
ENSMUSG00000024887	2.190149284	9.94E-47	3.27E-48	2.228656776	1.02E-47	3.41E-49	1.6633	7.5907	7.796
ENSMUSG00000024899	-7.822741041	1.34E-49	4.03E-51	-8.371177665	1.05E-55	2.93E-57	17.6593	0.078	0.0533
ENSMUSG00000024900	-1.613829802	4.04E-75	7.72E-77	-1.496909256	5.8E-42	2.25E-43	41.0053	13.3977	14.5287
ENSMUSG00000024905	-1.54042152	0.032915886	0.014863991	-1.614339659	0.046064296	0.021391084	0.893	0.307	0.2917
ENSMUSG00000024912	1.679277358	0.006509665	0.002532416	1.965261733	0.000000322	6.67E-08	0.648	2.0753	2.5303
ENSMUSG00000024921	-1.123250651	2.24E-52	6.31E-54	-1.065146026	3.2E-34	1.57E-35	57.7407	26.5063	27.5957
ENSMUSG00000024935	-4.350497247	0.033016623	0.014915394	-6.672425342	0.007828274	0.003051535	0.102	0.005	0
ENSMUSG00000024963	-1.093516881	0.00000516	0.00000127	-1.249310748	7.67E-08	0.000000015	11.174	5.2363	4.7003
ENSMUSG00000024965	1.330294951	3.41E-96	4.67E-98	1.54672514	7.21E-113	9.9E-115	88.567	222.7053	258.7513
ENSMUSG00000024968	1.509894855	7.35E-39	3.05E-40	1.460921177	2.4E-24	1.66E-25	8.3603	23.8093	23.0147
ENSMUSG00000024975	-1.538138442	3.9E-44	1.37E-45	-1.429966353	5.86E-43	2.22E-44	41.651	14.3417	15.4583
ENSMUSG00000025006	1.567402777	7.89E-51	2.31E-52	1.779308751	1.23E-45	4.3E-47	9.1937	27.2473	31.5583
ENSMUSG00000025010	1.158703457	2.1E-09	3.61E-10	1.433050778	2.85E-15	3.11E-16	1.7543	3.9167	4.737
ENSMUSG00000025026	-1.508944242	4.91E-52	1.39E-53	-1.702588566	9.57E-95	1.6E-96	167.3543	58.803	51.417
ENSMUSG00000025036	1.480070117	0.000000238	5.01E-08	1.609432731	2.41E-13	2.97E-14	1.1757	3.2797	3.5873
ENSMUSG00000025068	-1.067168982	6.51E-58	1.7E-59	-1.115925416	1.06E-42	4.04E-44	223.21	106.528	102.988
ENSMUSG00000025094	-1.056916099	4.36E-41	1.69E-42	-1.154172278	2.81E-18	2.56E-19	139.5267	67.0647	62.6927
ENSMUSG00000025197	-1.932891065	6.31E-08	1.25E-08	-2.193761616	4.56E-22	3.45E-23	8.3097	2.1763	1.8163
ENSMUSG00000025260	1.091134547	3.1E-30	1.65E-31	1.070178045	4.14E-37	1.83E-38	44.8747	95.6017	94.223
ENSMUSG00000025278	-2.012320335	1.9E-204	9.92E-207	-2.032375655	1.99E-140	2.13E-142	64.8757	16.081	15.859
ENSMUSG00000025283	-1.296221219	0.000173798	0.0000521	-1.418918243	5.15E-25	3.42E-26	71.308	29.036	26.6687
ENSMUSG00000025351	-1.592772136	2.06E-146	1.8E-148	-1.632233231	5.05E-88	9E-90	382.136	126.691	123.2727
ENSMUSG00000025355	-1.637394605	0.002304599	0.000827863	-2.902606067	1.15E-08	2.08E-09	1.513	0.4863	0.2023
ENSMUSG00000025407	-2.041101485	0.009107823	0.003659579	-2.448219321	0.000344109	0.0001052	0.7477	0.1817	0.137
ENSMUSG00000025408	-3.775134282	1.93E-79	3.47E-81	-3.451970733	2.57E-105	3.86E-107	52.203	3.813	4.7703
ENSMUSG00000025420	-2.272259308	0.0000311	0.0000084	-3.532495081	0.000000417	8.74E-08	1.161	0.2403	0.1003
ENSMUSG00000025421	1.057445434	6.99E-24	4.8E-25	1.161215167	5.82E-30	3.22E-31	12.239	25.4723	27.372
ENSMUSG00000025425	-4.389861771	0.03018	0.013503951	-4.197216693	0.026331955	0.011507959	0.1957	0.0093	0.0107
ENSMUSG00000025432	-2.962300651	2.04E-96	2.76E-98	-3.722718832	7.33E-152	6.93E-154	22.4483	2.8803	1.7003
ENSMUSG00000025477	1.672590646	2.47E-27	1.46E-28	1.766905229	4.36E-49	1.41E-50	5.818	18.547	19.8
ENSMUSG00000025492	2.373481342	5.75E-44	2.04E-45	2.186609986	1.9E-80	3.73E-82	22.428	116.2197	102.1
ENSMUSG00000025494	1.571641201	5.96E-23	4.26E-24	1.64197725	1.86E-24	1.27E-25	3.7597	11.1753	11.7337
ENSMUSG00000025511	2.704741935	1.47E-59	3.67E-61	2.896926314	1.16E-102	1.81E-104	7.3063	47.633	54.4203
ENSMUSG00000025571	-1.325953996	1.66E-72	3.33E-74	-1.200775237	2.52E-37	1.1E-38	23.6843	9.4473	10.3037
ENSMUSG00000025574	1.141108086	2.97E-45	1.02E-46	1.07247857	1.39E-31	7.35E-33	32.585	71.8663	68.5277
ENSMUSG00000025579	-1.891845092	1.33E-154	1.04E-156	-1.815626701	1.7E-68	3.82E-70	89.2927	24.061	25.3663
ENSMUSG00000025608	1.146426131	2.09E-25	1.32E-26	1.166533881	8.65E-26	5.53E-27	4.2593	9.4287	9.561
ENSMUSG00000025701	2.21805138	0.0000288	0.00000774	2.324275213	0.00000105	0.000000233	0.2457	1.143	1.2303
ENSMUSG00000025746	-4.36231424	9.9E-10	1.65E-10	-8.01726426	6.17E-25	4.12E-26	14.7677	0.718	0.057
ENSMUSG00000025809	1.023798877	1.97E-55	5.32E-57	1.061248085	2.8E-33	1.4E-34	78.7293	160.0777	164.2873
ENSMUSG00000025823	-2.074756461	2.89E-249	1.13E-251	-2.157537561	1.77E-149	1.72E-151	218.9807	51.9807	49.082
ENSMUSG00000025867	3.624490865	0.007206789	0.00283165	4.977279923	0.00000978	0.00000243	0.012	0.148	0.378
ENSMUSG00000025888	-1.041674229	0.012007804	0.004933251	-1.141128827	0.000381802	0.000117699	5.233	2.542	2.3727
ENSMUSG00000025921	1.596444289	1.62E-18	1.46E-19	1.520917751	9.7E-21	7.87E-22	3.4903	10.5547	10.0163
ENSMUSG00000025931	-1.81855494	1.83E-41	7.01E-43	-1.986811933	4.64E-48	1.54E-49	9.6553	2.7373	2.436
ENSMUSG00000025934	-4.686174752	0.049714097	0.023463534	-4.653007888	0.044127956	0.020376123	0.369	0.0143	0.0147
ENSMUSG00000025939	-1.57843488	9.28E-46	3.12E-47	-1.76214913	2.53E-69	5.62E-71	38.59	12.9217	11.3767
ENSMUSG00000025978	-1.424772077	0.0000108	0.00000275	-1.685204166	4.26E-09	7.4E-10	2.6767	0.997	0.8323
ENSMUSG00000025981	-1.006335386	0.00000333	0.000000798	-1.010705117	2.43E-10	3.82E-11	14.8343	7.3847	7.3623
ENSMUSG00000025986	1.709902187	3.27E-100	4.24E-102	1.755305477	4.66E-105	7.11E-107	5.198	17.0047	17.5483
ENSMUSG00000025997	-4.36260593	8.27E-10	1.37E-10	-5.33491753	0	0	101.5637	4.937	2.5163
ENSMUSG00000026003	-1.714230911	1.59E-106	1.95E-108	-1.652929859	5.55E-83	1.04E-84	90.5353	27.592	28.7897
ENSMUSG00000026004	-1.79113814	7.15E-14	8.59E-15	-2.645279465	1.35E-42	5.17E-44	8.1977	2.3687	1.3103
ENSMUSG00000026017	-1.612230521	1.2E-13	1.46E-14	-1.987024194	1.95E-15	2.1E-16	5.608	1.8343	1.4147
ENSMUSG00000026018	-3.950818154	6.37E-23	4.56E-24	-5.470192313	1.73E-30	9.41E-32	3.768	0.2437	0.085
ENSMUSG00000026024	-1.724309566	6.62E-80	1.19E-81	-1.621425706	1.01E-55	2.81E-57	18.2977	5.5377	5.947
ENSMUSG00000026094	1.695253894	4.63E-65	1.05E-66	1.41813973	2.12E-62	5.31E-64	24.447	79.1677	65.3323
ENSMUSG00000026107	-1.406961067	3.22E-25	2.09E-26	-1.845377547	8.69E-38	3.74E-39	26.0467	9.8223	7.2483
ENSMUSG00000026180	2.255045639	0.00000637	0.00000158	2.267882948	9.48E-21	7.68E-22	2.3517	11.2257	11.326
ENSMUSG00000026204	-7.058893689	0.004021745	0.001506164	-7.058893689	0.00311846	0.001126469	0.1333	0	0
ENSMUSG00000026209	-1.272558693	3.84E-33	1.87E-34	-1.170945868	2.07E-28	1.2E-29	86.7073	35.8903	38.5093
ENSMUSG00000026223	-1.221024419	9.05E-21	7.19E-22	-1.294880074	2.98E-38	1.27E-39	50.4797	21.6547	20.574
ENSMUSG00000026239	-1.298216948	1.31E-25	8.26E-27	-1.466371105	4.76E-41	1.89E-42	69.8303	28.395	25.271
ENSMUSG00000026259	-5.353636955	0.008931747	0.003582626	-4.353636955	0.022721334	0.009795085	0.2453	0.006	0.012
ENSMUSG00000026307	-1.127667162	6.38E-16	6.64E-17	-1.083979415	2.54E-15	2.76E-16	32.6447	14.94	15.3993
ENSMUSG00000026360	-1.321236818	0.028896685	0.012853454	-1.382336911	2.09E-24	1.44E-25	11.128	4.4533	4.2687
ENSMUSG00000026411	-1.682895561	8.72E-42	3.31E-43	-1.571056349	3.87E-45	1.38E-46	37.788	11.7693	12.718
ENSMUSG00000026435	-5.01282404	0.000000129	2.63E-08	-4.259464008	0.000000237	4.82E-08	0.7533	0.0233	0.0393
ENSMUSG00000026447	1.280226772	3.15E-26	1.94E-27	1.278323951	4.06E-24	2.83E-25	1.666	4.0463	4.041
ENSMUSG00000026456	-1.527606656	3.74E-42	1.4E-43	-1.618777398	2.09E-45	7.35E-47	61.987	21.5003	20.1837
ENSMUSG00000026482	-3.669505387	2.57E-19	2.21E-20	-3.569277939	2.1E-17	2.02E-18	1.7687	0.139	0.149
ENSMUSG00000026483	-2.681007981	3.46E-261	1.25E-263	-2.77591559	3.59E-250	1.69E-252	51.9413	8.0993	7.5837
ENSMUSG00000026484	-1.496630224	1.47E-93	2.11E-95	-1.560970907	4.06E-82	7.71E-84	72.4363	25.67	24.5503
ENSMUSG00000026509	1.314179713	8.35E-45	2.89E-46	1.4465817	1.95E-109	2.8E-111	31.6757	78.765	86.3357
ENSMUSG00000026519	1.556797618	1.5E-30	7.85E-32	1.545236817	1.54E-44	5.57E-46	6.161	18.1257	17.981
ENSMUSG00000026544	-2.498013619	2.14E-09	3.68E-10	-2.608119175	3.73E-13	4.65E-14	4.459	0.7893	0.7313
ENSMUSG00000026564	-3.791524693	0.0001746	0.0000524	-1.73744802	0.045343993	0.021019026	0.3323	0.024	0.0997
ENSMUSG00000026566	2.108524457	2.42E-172	1.63E-174	1.940883699	4.31E-147	4.26E-149	27.3653	118.013	105.0667
ENSMUSG00000026572	9.312882955	0.000000542	0.000000119	9.188176706	0.000000529	0.000000113	0	0.636	0.5833
ENSMUSG00000026576	-3.294416531	0.011612061	0.004756952	-2.149645007	0.017580514	0.007378621	0.2943	0.03	0.0663
ENSMUSG00000026579	5.335737711	1.86E-181	1.15E-183	5.633984112	1.27E-260	5.08E-263	0.48	19.3847	23.8363
ENSMUSG00000026580	4.891745126	5.4E-112	6.27E-114	4.902445319	2.33E-114	3.15E-116	0.3937	11.6867	11.7737
ENSMUSG00000026581	4.907733586	2.38E-95	3.3E-97	5.274493863	3.55E-132	4E-134	6.3303	190.021	245.0233
ENSMUSG00000026582	11.11556495	5.44E-52	1.55E-53	11.43429727	9.6E-35	4.59E-36	0.042	93.1897	116.2293
ENSMUSG00000026604	-1.754297322	6.81E-21	5.38E-22	-1.647698256	3.33E-17	3.25E-18	1.833	0.5433	0.585
ENSMUSG00000026638	-2.286994305	0.0115084	0.004707291	-4.186998515	0.0000553	0.0000152	0.34	0.0697	0.0187
ENSMUSG00000026649	-3.733463905	0.0000339	0.0000092	-4.839258569	0.00000367	0.000000865	2.1947	0.165	0.0767
ENSMUSG00000026655	-2.295410343	0.001246185	0.000426534	-3.274958155	0	0	239.9913	48.8887	24.7933
ENSMUSG00000026656	-2.788056317	0.008358865	0.003331352	-4.359464936	2.81E-249	1.36E-251	80.2477	11.6183	3.9093
ENSMUSG00000026657	2.291478384	5.98E-81	1.05E-82	2.384606125	1.03E-113	1.4E-115	3.0133	14.752	15.7357
ENSMUSG00000026670	-1.717001904	1.81E-76	3.4E-78	-1.879046939	2.79E-95	4.62E-97	51.8717	15.7783	14.102

ENSMUSG00000026672	-1.048618282	2.62E-12	3.54E-13	-1.139457058	8.69E-15	9.73E-16	18.3273	8.86	8.3193
ENSMUSG00000026678	-6.502500341	0.013267991	0.005490616	-6.502500341	0.010643604	0.004254796	0.0907	0	0
ENSMUSG00000026715	5.744161096	0.005136387	0.001957144	5.307428525	0.000650609	0.000208021	0.01	0.536	0.396
ENSMUSG00000026727	1.774557772	1.27E-120	1.37E-122	1.803839596	5.48E-103	8.47E-105	50.13	171.5113	175.028
ENSMUSG00000026728	-1.745310114	0.006393453	0.002481877	-2.599397714	1.78E-145	1.83E-147	183.41	54.7057	30.264
ENSMUSG00000026748	-3.542918743	8.51E-177	5.5E-179	-2.85058683	8.38E-126	1E-127	33.4897	2.8733	4.643
ENSMUSG00000026749	2.705503468	1.24E-76	2.32E-78	2.716665298	5.31E-61	1.36E-62	4.1057	26.7807	26.9887
ENSMUSG00000026764	-2.952405255	0.0002431	0.0000744	-3.698648663	0.0000173	0.00000444	0.2683	0.0347	0.0207
ENSMUSG00000026765	-9.343555875	0.00000118	0.000000269	-9.343555875	0.000000898	0.000000197	0.6497	0	0
ENSMUSG00000026784	1.738072663	5.55E-16	5.74E-17	1.686017785	3.12E-20	2.58E-21	3.908	13.0367	12.5747
ENSMUSG00000026805	6.745954377	0.019046607	0.008158484	6.619608644	0.049337443	0.02307796	0	0.1073	0.0983
ENSMUSG00000026812	1.192090259	9.36E-29	5.31E-30	1.104037342	7.12E-37	3.2E-38	5.4183	12.38	11.647
ENSMUSG00000026815	1.045920806	1.18E-36	5.24E-38	1.014695926	4.11E-18	3.8E-19	30.5383	63.052	61.702
ENSMUSG00000026829	9.501837185	0.0000339	0.00000922	8.255815423	0.006487727	0.002488664	0	0.725	0.3057
ENSMUSG00000026832	6.039973514	1.09E-31	5.53E-33	5.989121536	9.21E-13	1.18E-13	0.0347	2.281	2.202
ENSMUSG00000026836	-9.342815461	0.000000157	3.24E-08	-6.283921772	0.00000475	0.00000114	0.6493	0	0.0083
ENSMUSG00000026837	-4.248398816	0.00000497	0.00000122	-5.166434308	1.12E-83	2.09E-85	12.2463	0.6443	0.341
ENSMUSG00000026843	1.04094861	1.4E-31	7.12E-33	1.101894078	7.45E-32	3.89E-33	18.5133	38.0927	39.7363
ENSMUSG00000026854	1.26867524	3.05E-30	1.62E-31	1.249410927	1.73E-38	7.32E-40	9.3453	22.5167	22.218
ENSMUSG00000026864	-2.16813119	1.68E-153	1.34E-155	-2.062342368	1.69E-173	1.38E-175	1782.343	396.569	426.741
ENSMUSG00000026879	1.38755601	3.72E-21	2.91E-22	1.035205871	1.35E-19	1.16E-20	10.7477	28.1197	22.0263
ENSMUSG00000026880	-2.705220706	0.000406053	0.000128402	-3.624512963	1.8E-100	2.86E-102	14.5083	2.2247	1.1763
ENSMUSG00000026923	-1.574216119	1E-12	1.31E-13	-1.322490001	1.69E-09	2.84E-10	2.14	0.7187	0.8557
ENSMUSG00000026944	-2.664024375	0	0	-2.580957438	1.48E-135	1.63E-137	76.1403	12.0133	12.7253
ENSMUSG00000026970	1.168707817	8.09E-66	1.81E-67	1.351408059	1.96E-47	6.59E-49	21.827	49.0693	55.694
ENSMUSG00000026979	1.148224202	4.95E-72	1E-73	1.189693434	1.28E-80	2.5E-82	32.5063	72.0473	74.1483
ENSMUSG00000026988	1.608182177	1.22E-13	1.5E-14	1.569017996	1.44E-13	1.75E-14	3.609	11.0027	10.708
ENSMUSG00000027009	-2.862699853	0	0	-3.018942572	2.02E-196	1.39E-198	130.2753	17.9103	16.072
ENSMUSG00000027072	-3.845201432	0.000000017	3.21E-09	-7.397352063	3.95E-08	7.5E-09	3.5403	0.2463	0.021
ENSMUSG00000027073	-4.350854054	3.04E-13	3.84E-14	-6.396658789	7.24E-10	1.19E-10	302.7497	14.837	3.5933
ENSMUSG00000027078	2.229526922	1.87E-22	1.36E-23	2.429087434	8.1E-22	6.22E-23	2.5887	12.1403	13.9413
ENSMUSG00000027111	1.970734071	5.11E-20	4.23E-21	2.576310386	4.57E-08	8.72E-09	1.4773	5.7907	8.811
ENSMUSG00000027122	-3.471060663	0	0	-3.83310559	0	0	577.254	52.0563	40.503
ENSMUSG00000027130	1.105231995	1.96E-32	9.75E-34	1.099108529	2.71E-35	1.27E-36	12.9867	27.9387	27.8203
ENSMUSG00000027173	-2.346934138	1.63E-20	1.32E-21	-2.135270097	2.63E-24	1.82E-25	13.2103	2.5967	3.007
ENSMUSG00000027175	-1.49436198	2.03E-10	3.19E-11	-1.488461378	2.2E-10	3.45E-11	2.0623	0.732	0.735
ENSMUSG00000027195	1.75108221	8.97E-135	8.35E-137	1.73654291	7.87E-86	1.44E-87	25.7163	86.564	85.696
ENSMUSG00000027201	1.197826428	5.7E-41	2.23E-42	1.254411032	2.76E-42	1.06E-43	22.49	51.5907	53.6543
ENSMUSG00000027204	7.542567608	3.64E-22	2.69E-23	8.165510018	1.6E-27	9.63E-29	0.0083	1.5537	2.3927
ENSMUSG00000027210	1.153133223	0.00000054	0.000000119	1.213884315	3.51E-09	6.04E-10	2.2757	5.061	5.2787
ENSMUSG00000027233	-9.483815777	0.000000233	4.91E-08	-4.454068434	0.0000137	0.00000348	0.716	0	0.0327
ENSMUSG00000027239	5.137786156	0.0000235	0.00000625	4.884889801	0.0000577	0.0000159	0.058	2.042	1.7137
ENSMUSG00000027286	1.20772465	1.6E-12	2.14E-13	1.154481471	8.86E-11	1.33E-11	5.409	12.4933	12.0407
ENSMUSG00000027296	2.263567848	0.023167871	0.010115322	2.222087509	0.022222095	0.009556851	0.1127	0.541	0.5257
ENSMUSG00000027314	1.86823139	0.015368093	0.00645257	2.522498414	0.0000214	0.00000556	0.0983	0.359	0.565
ENSMUSG00000027315	4.14829991	5.1E-12	7.05E-13	3.826095992	3.56E-10	5.7E-11	0.0983	1.7437	1.3947
ENSMUSG00000027327	1.092914515	1.27E-32	6.26E-34	1.133537903	2.79E-29	1.57E-30	26.605	56.7497	58.3703
ENSMUSG00000027351	3.633635917	0	0	3.814824286	0	0	5.2887	65.6417	74.4257
ENSMUSG00000027360	-5.124559658	0.000041	0.0000113	-7.785030561	9.43E-36	4.37E-37	114.2503	3.275	0.518
ENSMUSG00000027366	-1.082751095	6.42E-26	4.01E-27	-1.136841208	9.99E-28	5.95E-29	37.893	17.8903	17.232
ENSMUSG00000027368	1.852893583	0.000000548	0.000000121	2.692906045	6.93E-24	4.89E-25	1.28	4.6237	8.2767
ENSMUSG00000027378	-1.943992497	2.42E-11	3.54E-12	-2.180804414	1.07E-17	1E-18	5.524	1.4357	1.2183
ENSMUSG00000027381	-1.032358437	2.48E-16	2.53E-17	-1.127919443	1.11E-13	1.33E-14	14.563	7.12	6.6637
ENSMUSG00000027387	1.045749269	0.000202294	0.0000611	1.225030834	0.00000837	0.00000206	1.795	3.7057	4.196
ENSMUSG00000027397	2.123881136	3.08E-67	6.63E-69	2.319668139	3.42E-66	8.04E-68	9.072	39.5417	45.289
ENSMUSG00000027400	-8.007494537	0.000743358	0.000244722	-8.007494537	0.000561432	0.000177686	0.2573	0	0
ENSMUSG00000027405	1.003896651	4.21E-46	1.4E-47	1.071350581	3.86E-81	7.45E-83	116.0983	232.8247	243.969
ENSMUSG00000027438	-1.011025618	0.001611996	0.000562939	-1.49888943	0.00000669	0.00000163	1.7513	0.869	0.6197
ENSMUSG00000027489	1.124988577	0.046468909	0.021760814	1.275559004	0.013611766	0.005560675	0.5543	1.209	1.342
ENSMUSG00000027634	1.484411794	2.3E-126	2.35E-128	1.437190511	5.41E-87	9.82E-89	31.4833	88.0913	85.2547
ENSMUSG00000027636	5.65543754	0	0	5.54663294	0	0	1.603	80.796	74.9267
ENSMUSG00000027637	1.227446164	3.36E-60	8.28E-62	1.185474633	6.92E-39	2.9E-40	52.2447	122.332	118.8243
ENSMUSG00000027639	1.593967958	1.43E-80	2.53E-82	1.585029332	6.79E-91	1.18E-92	14.391	43.4433	43.175
ENSMUSG00000027646	2.562062099	0.029263426	0.013036912	3.246132744	0.001514842	0.00051687	0.0423	0.25	0.4017
ENSMUSG00000027669	1.11979188	0.000383538	0.000120803	1.307715544	0.000000559	0.000000119	0.7277	1.5813	1.8013
ENSMUSG00000027712	1.520140166	4.01E-32	2.01E-33	1.286890063	6.61E-31	3.54E-32	25.6453	73.5557	62.575
ENSMUSG00000027737	-1.991802141	0.002005879	0.000712892	-2.788790348	6.33E-48	2.1E-49	3.8607	0.9707	0.5587
ENSMUSG00000027763	1.207857699	2.5E-82	4.33E-84	1.303666038	1.4E-85	2.57E-87	143.0217	330.372	353.0567
ENSMUSG00000027765	5.060023281	0	0	5.142147923	0	0	5.311	177.172	187.55
ENSMUSG00000027778	-1.22237319	2.75E-26	1.69E-27	-1.514177611	5.26E-28	3.1E-29	16.6707	7.1447	5.8363
ENSMUSG00000027803	6.279223644	0.016439423	0.006943501	9.316658658	1.05E-08	1.89E-09	0	0.0777	0.6377
ENSMUSG00000027834	-5.213409896	5.35E-163	3.9E-165	-5.453398835	2.11E-202	1.4E-204	22.2733	0.6003	0.5083
ENSMUSG00000027843	-2.338046208	5.98E-15	6.7E-16	-2.706416677	3.79E-45	1.35E-46	10.441	2.065	1.5997
ENSMUSG00000027931	-1.008179178	0.000598384	0.000194002	-1.118455447	0.00000267	0.000000619	3.125	1.5537	1.4393
ENSMUSG00000027935	1.563875035	1.9E-10	2.99E-11	1.622398962	2.36E-11	3.39E-12	3.0093	8.897	9.2653
ENSMUSG00000027939	1.610679671	0.018161638	0.007747872	1.454706115	0.021444538	0.00918544	0.0927	0.283	0.254
ENSMUSG00000027950	1.082937991	0.000426141	0.000135258	1.04764871	0.00158577	0.000542714	0.814	1.7243	1.6827
ENSMUSG00000027953	1.140357263	1.53E-12	2.04E-13	1.019338295	6.89E-11	1.03E-11	13.7587	30.329	27.8887
ENSMUSG00000027962	-3.394397733	0.005779204	0.002222554	-3.268866851	0.007332642	0.002842133	0.2313	0.022	0.024
ENSMUSG00000027963	1.126791926	3.38E-18	3.08E-19	1.02483254	2.59E-15	2.82E-16	4.7807	10.4397	9.7273
ENSMUSG00000027981	1.064826057	6.07E-18	5.66E-19	1.103330501	2.18E-22	1.64E-23	9.5847	20.0503	20.5927
ENSMUSG00000028035	1.256580224	6.88E-21	5.43E-22	1.474324546	3.43E-28	2.01E-29	8.5567	20.4443	23.775
ENSMUSG00000028063	1.735428156	5.94E-49	1.86E-50	1.903009143	8.5E-62	2.15E-63	10.8597	36.1603	40.6143
ENSMUSG00000028064	1.436339563	7.33E-27	4.42E-28	1.397697435	2.92E-21	2.3E-22	3.1737	8.589	8.362
ENSMUSG00000028069	1.027888697	3.49E-15	3.85E-16	1.048485752	9.89E-21	8.03E-22	14.1657	28.8843	29.2997
ENSMUSG00000028071	-7.133876128	2.16E-14	2.5E-15	-6.811948033	2.54E-16	2.6E-17	2.6217	0.0187	0.0233
ENSMUSG00000028086	1.300945153	2.36E-24	1.58E-25	1.498635724	3.21E-41	1.27E-42	4.5663	11.251	12.9033
ENSMUSG00000028088	1.500243043	0.010669985	0.004336937	1.404816237	0.008143853	0.003188607	0.2007	0.5677	0.5313
ENSMUSG00000028100	-2.39021522	0.00271652	0.000987723	-1.820032877	0.037994436	0.017222634	1.543	0.2943	0.437
ENSMUSG00000028121	-9.499845887	0.00000288	0.000000686	-3.356887933	0.000222717	0.0000666	0.724	0	0.0707
ENSMUSG00000028124	-1.331260178	0.0000218	0.00000576	-1.032579712	1.64E-17	1.57E-18	107.4813	42.7153	52.5407
ENSMUSG00000028175	1.058799568	3.18E-35	1.46E-36	1.011562347	7.27E-20	6.18E-21	16.9637	35.3387	34.2003
ENSMUSG00000028182	-2.653442239	0.020942465	0.009057853	-4.144428591	0.002313812	0.00081616	0.6543	0.104	0.037
ENSMUSG00000028188	-1.40369268	0.00162743	0.000568894	-1.4561601	0.025594117	0.011153691	2.7163	1.0267	0.99
ENSMUSG00000028197	6.426264755	0.00064453	0.000209858	6.594946589	0.000192988	0.0000572	0	0.086	0.0967
ENSMUSG00000028211	-1.231080881	6.17E-13	7.97E-14	-1.773734823	3.95E-55	1.12E-56	14.5267	6.1883	4.2483
ENSMUSG00000028212	1.21887357	5.92E-24	4.06E-25	1.287606417	5.62E-24	3.94E-25	6.216	14.4687	15.1747
ENSMUSG00000028214	15.1694553	7.28E-17	7.21E-18	14.91073641	2.72E-17	2.64E-18	0	36.852	30.802
ENSMUSG00000028217	11.32755264	8.15E-14	9.83E-15	11.02974734	4.14E-13	5.17E-14	0	2.57	2.0907
ENSMUSG00000028223	-1.545509109	1.49E-74	2.9E-76	-1.577752856	4.56E-57	1.23E-58	55.8233	19.1237	18.701
ENSMUSG00000028256	-1.187958581	2.48E-11	3.63E-12	-1.244343154	3.78E-11	5.51E-12	13.2157	5.8007	5.5783
ENSMUSG00000028266	-3.356949011	0	0	-3.240473253	2.62E-252	1.21E-254	196.547	19.1833	20.7963
ENSMUSG00000028295	-1.215237226	0.000185993	0.000056	-1.062105982	0.001753751	0.000604683	8.805	3.7923	4.217
ENSMUSG00000028300	2.134952338	1.16E-12	1.53E-13	2.223666324	3.89E-14	4.55E-15	0.566	2.486	2.6437
ENSMUSG00000028332	3.808181729	1.99E-51	5.74E-53	3.326590593	5.63E-48	1.87E-49	1.246	17.454	12.5003
ENSMUSG00000028339	1.689016633	6.68E-19	5.91E-20	2.054995509	2.86E-29	1.62E-30	1.1217	3.6167	4.661
ENSMUSG00000028410	1.151248105	5.61E-36	2.53E-37	1.047237064	5.81E-44	2.13E-45	72.537	161.109	149.9027
ENSMUSG00000028420	-2.152077751	4.85E-25	3.15E-26	-2.264684562	7.12E-28	4.23E-29	7.873	1.7713	1.6383
ENSMUSG00000028435	4.28104362	0.025106183	0.011034861	4.424289813	0.019914367	0.008458519	0.0113	0.2203	0.2433
ENSMUSG00000028439	-1.409127229	2.45E-16	2.51E-17	-1.546394844	2.99E-10	4.75E-11	7.5787	2.8537	2.5947
ENSMUSG00000028445	-4.679629827	0.000855823	0.000285254	-3.420437487	0.001927228	0.000669021	0.803	0.0313	0.075
ENSMUSG00000028456	3.304854582	0.001242009	0.000424759	2.878321443	0.00588353	0.002234149	0.017	0.168	0.125
ENSMUSG00000028465	2.298206646	2.03E-244	8.02E-247	2.431946098	1.77E-259	7.23E-262	103.3193	508.172	557.5327
ENSMUSG00000028476	-2.585334857	1.27E-29	6.93E-31	-2.652413586	2.19E-33	1.09E-34	3.875	0.6457	0.6163
ENSMUSG00000028480	1.51069517	5.98E-32	3.01E-33	1.359260241	5.41E-16	5.64E-17	10.6913	30.4647	27.429
ENSMUSG00000028494	-1.561386793	5.69E-37	2.49E-38	-1.703704714	3.5E-42	1.35E-43	50.7617	17.1993	15.5837
ENSMUSG00000028521	-1.924126829	5.82E-39	2.41E-40	-1.952038445	1.64E-43	6.11E-45	17.7597	4.6797	4.59
ENSMUSG00000028528	-1.894211696	2.79E-154	2.19E-156	-1.903818846	4.47E-90	7.81E-92	67.208	18.0803	17.9603
ENSMUSG00000028536	11.2848241	6.31E-09	1.14E-09	11.7918412	3.47E-10	5.54E-11	0	2.495	3.5457
ENSMUSG00000028542	-2.091204705	9.07E-38	3.87E-39	-2.035525109	8.83E-34	4.35E-35	15.0883	3.541	3.6803
ENSMUSG00000028552	-1.057248371	1.01E-56	2.67E-58	-1.154794976	3.47E-53	1.03E-54	108.8133	52.29	48.8713
ENSMUSG00000028555	-4.730608726	1.02E-22	7.37E-24	-3.57363938	5.06E-26	3.2E-27	3.8497	0.145	0.3233
ENSMUSG00000028558	-3.232081478	0.019015058	0.008142328	-8.375039431	0.000533504	0.000168258	0.332	0.0353	0
ENSMUSG00000028568	1.193598492	6.41E-23	4.59E-24	1.272072797	3.49E-18	3.21E-19	15.0323	34.3823	36.3043
ENSMUSG00000028576	-1.074590237	0.000000768	0.000000172	-1.135385128	0.000000179	3.62E-08	6.397	3.0373	2.912
ENSMUSG00000028602	3.355658952	0.002951453	0.001078065	3.149091498	0.005917814	0.002248394	0.031	0.3173	0.275
ENSMUSG00000028613	1.216502504	1.23E-15	1.31E-16	1.375359412	3.47E-34	1.7E-35	6.8257	15.8617	17.708
ENSMUSG00000028626	-6.965784285	0.013707805	0.005693572	-6.965784285	0.011009149	0.004413085	0.125	0	0
ENSMUSG00000028641	-1.178042719	5.87E-23	4.19E-24	-1.070329367	1.11E-19	9.5E-21	16.842	7.4433	8.0203
ENSMUSG00000028645	1.132836521	2.4E-27	1.42E-28	1.014668734	9.36E-28	5.57E-29	17.2547	37.8377	34.862
ENSMUSG00000028656	1.447298558	2.67E-98	3.57E-100	1.549790344	2.69E-193	1.88E-195	180.5757	492.4243	528.6797
ENSMUSG00000028669	-1.896336916	2.88E-64	6.67E-66	-1.580673785	2.26E-25	1.47E-26	62.0283	16.6623	20.7377
ENSMUSG00000028680	-2.029296631	1.17E-27	6.8E-29	-1.428734374	0.009042959	0.003571856	16.98	4.1597	6.3073
ENSMUSG00000028687	-1.026792575	0.002898969	0.001058088	-1.036858563	0.009017105	0.003560399	7.2283	3.5477	3.523
ENSMUSG00000028689	-1.332406521	6.86E-10	1.13E-10	-1.507493228	5.75E-13	7.25E-14	20.8887	8.295	7.347
ENSMUSG00000028696	-1.563807209	8.71E-19	7.79E-20	-1.523493402	3.34E-21	2.65E-22	12.2757	4.1523	4.27
ENSMUSG00000028756	-1.702218993	1.63E-25	1.03E-26	-1.650294639	1.74E-25	1.12E-26	13.7927	4.2387	4.394
ENSMUSG00000028757	-1.32912748	7.51E-73	1.5E-74	-1.356373542	3.86E-75	8.07E-77	165.6353	65.9243	64.691
ENSMUSG00000028758	-2.978223058	0.0000698	0.0000199	-3.324673472	0.00000207	0.000000474	0.7013	0.089	0.07
ENSMUSG00000028763	-3.989946335	0.0000511	0.0000142	-2.678744647	0.000185785	0.0000548	0.143	0.009	0.0223
ENSMUSG00000028771	1.851250569	8.92E-104	1.11E-105	1.960999171	3.97E-206	2.5E-208	26.558	95.8247	103.3987
ENSMUSG00000028779	-1.181779168	8.83E-16	9.27E-17	-1.286188008	2.71E-25	1.78E-26	39.6757	17.4893	16.2683
ENSMUSG00000028793	1.067974487	4.37E-08	8.53E-09	1.56453866	7.36E-23	5.4E-24	7.849	16.4553	23.216
ENSMUSG00000028799	-1.361305806	4.46E-40	1.78E-41	-1.208000994	3.84E-29	2.17E-30	28.4613	11.078	12.32
ENSMUSG00000028807	-6.836634844	0.00000079	0.000000177	-10.00655985	1.18E-08	2.13E-09	1.0287	0.009	0
ENSMUSG00000028836	-6.815916936	0.046073995	0.021537472	-6.815916936	0.038726467	0.01758269	0.1127	0	0
ENSMUSG00000028838	-4.171705008	9.28E-54	2.56E-55	-4.433727351	1.04E-48	3.38E-50	5.1363	0.285	0.2377

ENSMUSG00000028849	-1.432379191	8.62E-36	3.9E-37	-1.468214925	6.11E-28	3.61E-29	23.2853	8.6277	8.416
ENSMUSG00000028862	3.185428739	8.26E-85	1.39E-86	3.008504623	9.9E-76	2.06E-77	1.3883	12.63	11.1723
ENSMUSG00000028864	-7.132713922	0.006200986	0.002401132	-3.962788921	0.02021851	0.008608641	0.1403	0	0.009
ENSMUSG00000028878	-1.065782813	3.87E-19	3.37E-20	-1.072309321	1.08E-13	1.29E-14	19.324	9.2313	9.1897
ENSMUSG00000028885	1.689480336	1.47E-11	2.11E-12	1.769057327	1.67E-13	2.03E-14	1.4197	4.579	4.8387
ENSMUSG00000028893	-1.036553533	6.83E-25	4.48E-26	-1.023684767	0.003545349	0.001294625	42.355	20.6477	20.8327
ENSMUSG00000028970	2.120422684	1.57E-44	5.46E-46	2.010636395	4.47E-57	1.2E-58	2.7703	12.046	11.1633
ENSMUSG00000028977	-2.951240688	1.62E-22	1.18E-23	-2.430144392	3.82E-20	3.19E-21	1.7067	0.2207	0.3167
ENSMUSG00000029004	-1.292923287	1.62E-42	6E-44	-1.254402099	2.14E-48	7.01E-50	32.959	13.4513	13.8153
ENSMUSG00000029005	1.060082001	0.000000204	4.26E-08	1.35300919	1.9E-11	2.72E-12	1.1327	2.3617	2.8933
ENSMUSG00000029060	-1.813757415	1.59E-33	7.63E-35	-1.895679365	6.34E-38	2.72E-39	16.7493	4.7643	4.5013
ENSMUSG00000029070	-1.347993932	0.000000368	7.94E-08	-1.561268599	1.78E-08	3.27E-09	4.952	1.9453	1.678
ENSMUSG00000029096	4.727495069	0.0000843	0.0000243	5.004571615	0.00000415	0.000000988	0.0427	1.1303	1.3697
ENSMUSG00000029162	1.337704125	1.6E-35	7.3E-37	1.38965253	5.63E-27	3.47E-28	16.3163	41.2393	42.7513
ENSMUSG00000029165	-1.289106859	6.94E-11	1.05E-11	-1.134581397	0.000000018	3.31E-09	12.429	5.086	5.661
ENSMUSG00000029171	1.1671678	3.48E-44	1.22E-45	1.185962096	1.54E-38	6.54E-40	20.7947	46.6987	47.311
ENSMUSG00000029201	-1.848166313	2.09E-90	3.15E-92	-1.982229961	4.12E-95	6.85E-97	61.8877	17.189	15.6637
ENSMUSG00000029217	-1.426293811	1.31E-26	7.95E-28	-1.628441645	5.24E-36	2.42E-37	32.067	11.9317	10.3717
ENSMUSG00000029254	-1.89140383	0.001023621	0.000345308	-1.55173359	0.000000345	7.17E-08	2.8183	0.7597	0.9613
ENSMUSG00000029263	1.517181061	5.74E-47	1.88E-48	1.526860546	4.13E-35	1.94E-36	4.394	12.577	12.6617
ENSMUSG00000029265	1.710680491	1.7E-61	4.11E-63	1.981587181	4.46E-174	3.61E-176	37.265	121.974	147.1697
ENSMUSG00000029270	-1.12037518	0.0000372	0.0000102	-1.199548972	0.000000165	3.32E-08	7.491	3.4457	3.2617
ENSMUSG00000029291	-1.405274395	3.04E-22	2.24E-23	-1.346891153	1.18E-16	1.18E-17	10.8817	4.1083	4.278
ENSMUSG00000029298	6.560853882	0.000000476	0.000000104	6.803250524	0.000000169	3.41E-08	0.0073	0.6923	0.819
ENSMUSG00000029311	-1.073358143	2.16E-12	2.91E-13	-1.312837079	2.37E-20	1.95E-21	48.645	23.1167	19.581
ENSMUSG00000029312	-1.070047498	0.003224942	0.00118625	-1.572915966	0.00000138	0.000000311	1.8287	0.871	0.6147
ENSMUSG00000029313	-1.943707871	5.48E-127	5.48E-129	-1.999071214	6.34E-110	9.06E-112	60.5597	15.7423	15.1497
ENSMUSG00000029314	-2.173073519	0.000457937	0.000146114	-2.295660124	0.00000659	0.0000016	1.0703	0.2373	0.218
ENSMUSG00000029322	-3.444120353	0.000000739	0.000000165	-4.763441334	0	0	496.4317	45.6117	18.2777
ENSMUSG00000029344	3.744232154	1.3E-221	5.76E-224	3.941297347	0	0	8.8887	119.114	136.548
ENSMUSG00000029359	-3.588252571	0.000622572	0.00020232	-10.19311463	0.00000552	0.00000133	1.1707	0.0973	0
ENSMUSG00000029372	10.32568023	0.0000018	0.00000042	8.456696651	0.00833752	0.003269926	0	1.2833	0.3513
ENSMUSG00000029373	4.917688712	0.000000498	0.000000109	4.205608797	0.000797935	0.000258872	0.1523	4.6043	2.8107
ENSMUSG00000029403	-1.227577315	0.001182081	0.000402868	-1.21550809	0.00000164	0.000000373	2.6017	1.111	1.1203
ENSMUSG00000029406	1.512735397	5.62E-24	3.84E-25	1.710630436	2.54E-27	1.54E-28	1.7543	5.006	5.742
ENSMUSG00000029428	-1.006791886	6.94E-11	1.05E-11	-1.029567144	6.76E-16	7.09E-17	13.3427	6.64	6.536
ENSMUSG00000029442	2.075571576	1.48E-15	1.59E-16	2.680624869	5.26E-20	4.44E-21	0.7763	3.2723	4.9773
ENSMUSG00000029456	-2.283276938	5.1E-29	2.85E-30	-2.089620979	3.48E-25	2.3E-26	6.0767	1.2483	1.4277
ENSMUSG00000029470	-3.781491106	1.56E-87	2.52E-89	-3.646373491	1.18E-117	1.54E-119	32.0267	2.329	2.5577
ENSMUSG00000029471	1.374509161	2.73E-45	9.33E-47	1.501602133	3.43E-46	1.19E-47	9.6717	25.0767	27.386
ENSMUSG00000029478	1.407220412	1.46E-81	2.54E-83	1.502391076	3.41E-47	1.16E-48	18.3823	48.7547	52.0793
ENSMUSG00000029484	-1.224887441	0.017809394	0.007580282	-1.947746078	2.77E-59	7.2E-61	71.441	30.5647	18.519
ENSMUSG00000029499	-1.187352073	0.043674765	0.020282445	-1.480399048	0.002572633	0.000915804	3.3037	1.4507	1.184
ENSMUSG00000029512	-1.691523944	8.23E-36	3.72E-37	-1.513916258	4.02E-25	2.65E-26	11.292	3.496	3.954
ENSMUSG00000029516	-1.127376817	1.32E-37	5.68E-39	-1.299592646	1.54E-12	2E-13	29.718	13.6033	12.0727
ENSMUSG00000029553	10.53203118	3.42E-09	6.03E-10	10.82601877	1.25E-09	2.09E-10	0	1.4807	1.8153
ENSMUSG00000029561	4.6124706	1.53E-41	5.85E-43	5.041437884	2.34E-54	6.78E-56	0.2763	6.7597	9.1003
ENSMUSG00000029570	1.26217907	5.13E-10	8.35E-11	1.329133806	9.32E-11	1.4E-11	2.3433	5.6207	5.8877
ENSMUSG00000029580	1.694314732	1.15E-127	1.13E-129	1.531743387	7.77E-169	6.59E-171	919.7383	2976.486	2659.2857
ENSMUSG00000029586	9.262877855	0.00000472	0.00000115	7.725650281	0.004207351	0.001559601	0	0.6143	0.2117
ENSMUSG00000029638	-3.854074312	3.69E-190	2.13E-192	-3.481195415	1.03E-16	1.02E-17	45.484	3.1453	4.073
ENSMUSG00000029651	-5.650446225	0.006002455	0.002316335	-4.553810442	1.23E-11	1.71E-12	2.7793	0.0553	0.1183
ENSMUSG00000029718	-3.28522555	1.49E-12	1.98E-13	-7.107817911	1.79E-09	3.01E-10	3.4023	0.349	0.0247
ENSMUSG00000029725	-1.212976096	0.000000626	0.000000139	-1.068800243	0.000000769	0.000000166	14.881	6.4193	7.094
ENSMUSG00000029752	-1.333922486	1.64E-42	6.08E-44	-1.43568048	2.09E-99	3.34E-101	275.9253	109.4563	102.002
ENSMUSG00000029762	1.152303466	1.45E-14	1.67E-15	1.2428392	6.18E-10	1.01E-10	10.1653	22.5943	24.0577
ENSMUSG00000029765	6.402585758	0.00000668	0.00000166	6.545350645	0.00000309	0.000000722	0.0017	0.141	0.1557
ENSMUSG00000029769	-2.037571036	1.61E-13	1.99E-14	-1.94973603	4.56E-19	4.02E-20	4.9923	1.216	1.2923
ENSMUSG00000029811	-9.928765095	1.09E-08	0.000000002	-9.928765095	8.66E-09	1.54E-09	0.9747	0	0
ENSMUSG00000029860	2.705039747	4.77E-176	3.12E-178	2.948093673	4.67E-178	3.68E-180	43.7817	285.4897	337.8757
ENSMUSG00000029861	6.523561956	0.011223283	0.004581314	7.342815461	0.000647677	0.000207038	0	0.092	0.1623
ENSMUSG00000029862	4.054295977	0.002444011	0.000880829	4.400103168	0.000484546	0.000151881	0.0147	0.2437	0.3097
ENSMUSG00000029925	-5.115552607	2.1E-10	3.31E-11	-1.41149862	0.003254469	0.001180786	2.1263	0.0613	0.7993
ENSMUSG00000030000	-1.344264425	0.022491337	0.009801192	-1.715631452	0.008733072	0.003439205	0.5967	0.235	0.1817
ENSMUSG00000030032	2.562211268	6.24E-10	1.02E-10	2.360278268	3.39E-08	6.38E-09	0.1633	0.9647	0.8387
ENSMUSG00000030035	-1.128681447	4.63E-14	5.5E-15	-1.120626854	4.51E-16	4.67E-17	29.422	13.4557	13.531
ENSMUSG00000030041	2.89330223	2.76E-12	3.74E-13	2.259954835	4.33E-09	7.52E-10	0.723	5.3717	3.463
ENSMUSG00000030054	6.091553679	2.01E-49	6.12E-51	6.332336897	5.81E-56	1.61E-57	0.3763	25.6633	30.3247
ENSMUSG00000030060	-1.399817675	2.8E-18	2.55E-19	-1.389026391	2.34E-21	1.83E-22	24.4843	9.279	9.3487
ENSMUSG00000030096	1.651235966	7.05E-57	1.86E-58	1.744384919	5.39E-66	1.27E-67	7.9843	25.079	26.7517
ENSMUSG00000030102	1.149554362	2.74E-41	1.06E-42	1.133216124	1.32E-54	3.79E-56	18.8003	41.7077	41.238
ENSMUSG00000030103	-1.749238982	3.9E-18	3.59E-19	-1.97621307	1.02E-54	2.9E-56	16.0807	4.7833	4.087
ENSMUSG00000030104	-1.749097998	4.41E-124	4.62E-126	-1.622329525	5.38E-74	1.14E-75	73.333	21.8157	23.8193
ENSMUSG00000030107	6.413441103	9.5E-38	4.06E-39	6.197452872	4.2E-32	2.19E-33	0.1283	10.939	9.418
ENSMUSG00000030123	1.675402904	1.46E-122	1.56E-124	1.841048041	8.53E-79	1.71E-80	10.4713	33.4463	37.5157
ENSMUSG00000030124	-2.375832071	4.78E-19	4.18E-20	-2.393659068	3.33E-20	2.76E-21	5.917	1.14	1.126
ENSMUSG00000030134	-3.859930583	5.93E-10	9.7E-11	-4.617472539	3.94E-12	5.3E-13	1.481	0.102	0.0603
ENSMUSG00000030137	3.881859264	4.01E-255	1.5E-257	3.937531075	0	0	8.4673	124.8253	129.7363
ENSMUSG00000030142	-3.545273826	0.001765481	0.00062085	-4.058214746	1.25E-33	6.18E-35	42.7243	3.6597	2.5647
ENSMUSG00000030144	2.172223162	1.03E-09	1.72E-10	2.420119147	2.74E-15	2.99E-16	2.1373	9.6333	11.4393
ENSMUSG00000030154	-5.337344654	0.0000519	0.0000145	-7.601939574	1.79E-11	2.55E-12	4.9863	0.1233	0.0257
ENSMUSG00000030156	-2.036909307	0.046361412	0.021694371	-1.949879639	3.3E-30	1.81E-31	23.3183	5.6823	6.0357
ENSMUSG00000030157	-3.879369248	1.22E-13	1.49E-14	-5.870756118	6.09E-15	6.77E-16	4.9153	0.334	0.084
ENSMUSG00000030162	-5.619227837	1.38E-08	2.59E-09	-8.419307428	9.98E-168	8.61E-170	48.613	0.989	0.142
ENSMUSG00000030200	-1.676302812	0.000248771	0.0000762	-1.490375952	0.000038	0.0000102	3.6403	1.139	1.2957
ENSMUSG00000030203	-1.864575017	9.17E-31	4.77E-32	-1.444683914	9.22E-35	4.39E-36	8.735	2.3987	3.209
ENSMUSG00000030228	-5.308612378	3.01E-16	3.09E-17	-6.495688541	0	0	124.9217	3.152	1.3843
ENSMUSG00000030231	-1.053192861	4.79E-23	3.4E-24	-1.043368209	2.91E-21	2.3E-22	29.0523	14.0003	14.096
ENSMUSG00000030257	3.798884143	3.08E-146	2.74E-148	3.950154353	5.06E-147	5.06E-149	0.7687	10.6983	11.881
ENSMUSG00000030263	2.252548896	9.88E-152	8.1E-154	2.253506407	2.31E-98	3.74E-100	11.379	54.2237	54.2597
ENSMUSG00000030281	1.748724586	1.06E-14	1.2E-15	1.613402269	2.37E-17	2.3E-18	4.13	13.8793	12.6367
ENSMUSG00000030303	-3.856270789	4.6E-125	4.8E-127	-3.997639539	1.73E-124	2.11E-126	15.878	1.0963	0.994
ENSMUSG00000030309	1.059427007	9.76E-11	1.5E-11	1.206214882	1.27E-13	1.53E-14	2.921	6.0877	6.7397
ENSMUSG00000030341	1.353521389	8.97E-25	5.91E-26	1.319346055	1.44E-21	1.12E-22	6.1517	15.7197	15.3517
ENSMUSG00000030350	7.953402821	3E-12	4.07E-13	7.870785024	1.41E-12	1.83E-13	0.0147	3.6353	3.433
ENSMUSG00000030365	-2.098950651	3.76E-39	1.55E-40	-2.426010398	6.85E-45	2.45E-46	37.1007	8.6603	6.9037
ENSMUSG00000030403	1.097913058	3.95E-25	2.56E-26	1.058496972	1.96E-21	1.53E-22	43.354	92.797	90.296
ENSMUSG00000030410	8.944858446	4.17E-08	8.12E-09	7.778734244	3.12E-09	5.34E-10	0.0083	4.1067	1.83
ENSMUSG00000030413	-2.500524789	1.1E-09	1.85E-10	-3.335820003	1.49E-19	1.29E-20	12.244	2.1637	1.2127
ENSMUSG00000030433	-1.607356473	0.000104973	0.0000306	-1.503160021	0.000164844	0.0000482	3.1597	1.037	1.1147
ENSMUSG00000030465	8.664150952	6.15E-09	1.1E-09	8.151439431	0.003566794	0.001302702	0	0.4057	0.2843
ENSMUSG00000030468	-9.665928035	4.46E-08	8.72E-09	-5.206496416	0.00000114	0.000000253	0.8123	0	0.022
ENSMUSG00000030470	-3.49562427	0.001237692	0.000423197	-4.691605061	0.0000699	0.0000195	1.628	0.1443	0.063
ENSMUSG00000030499	-1.520956872	0.001620018	0.000565965	-1.858306962	0.0000124	0.00000311	1.8443	0.6427	0.5087
ENSMUSG00000030539	2.631186682	1.73E-83	2.96E-85	2.725199425	6.55E-106	9.77E-108	4.032	24.9797	26.6617
ENSMUSG00000030546	-2.707201043	0.00016045	0.0000479	-1.584540968	0.004248736	0.001575822	1.1407	0.1747	0.3803
ENSMUSG00000030555	2.406750045	0.000000636	0.000000141	2.558500621	0.00000718	0.00000176	0.2873	1.5237	1.6927
ENSMUSG00000030557	1.465130845	4.5E-67	9.75E-69	1.583176696	2.43E-99	3.89E-101	21.4667	59.267	64.3203
ENSMUSG00000030559	-1.870716348	1.21E-50	3.57E-52	-2.046477341	2.18E-63	5.38E-65	43.2567	11.828	10.4713
ENSMUSG00000030589	3.179727576	4.47E-08	8.75E-09	2.428877477	3.67E-08	6.94E-09	0.201	1.8213	1.0823
ENSMUSG00000030616	-5.596935142	0.00001	0.00000253	-5.374542721	0.00000348	0.00000082	0.484	0.01	0.0117
ENSMUSG00000030638	13.23227101	1E-16	1E-17	13.69700374	3.05E-18	2.79E-19	0	9.623	13.2803
ENSMUSG00000030643	-3.589179677	4.43E-08	8.67E-09	-3.701563094	8.18E-10	1.35E-10	1.713	0.1423	0.1317
ENSMUSG00000030657	-3.233828748	4.29E-10	6.94E-11	-3.996000194	1.17E-187	8.65E-190	11.286	1.1997	0.7073
ENSMUSG00000030659	2.273099647	0.001765612	0.000621122	2.909242177	0.00000116	0.000000258	0.2043	0.9877	1.535
ENSMUSG00000030663	1.162488901	1.53E-61	3.71E-63	1.262095178	7.35E-47	2.52E-48	56.596	126.6863	135.742
ENSMUSG00000030671	-1.358824046	2.38E-54	6.5E-56	-1.254808998	5.47E-25	3.64E-26	26.759	10.4333	11.2133
ENSMUSG00000030672	1.750447776	0.0000869	0.000025	1.40275917	0.011768206	0.00474417	1.4947	5.029	3.952
ENSMUSG00000030674	-2.21400366	0.003386516	0.001250153	-4.725902699	0.00000439	0.00000105	0.9527	0.2053	0.036
ENSMUSG00000030691	1.092337645	4.65E-13	5.95E-14	1.25235116	1.05E-15	1.11E-16	2.4737	5.2743	5.893
ENSMUSG00000030701	1.695341567	2.25E-49	6.91E-51	1.388699959	8.28E-40	3.38E-41	15.25	49.3877	39.931
ENSMUSG00000030707	1.487046865	1.2E-140	1.08E-142	1.228222599	4.82E-76	9.89E-78	96.3353	270.042	225.6927
ENSMUSG00000030717	-6.290360232	0.00000104	0.000000236	-11.46028523	1.61E-08	2.95E-09	2.8177	0.036	0
ENSMUSG00000030731	-1.918217955	0.010406912	0.004224216	-2.119562728	0.006328849	0.002422476	0.6287	0.1663	0.1447
ENSMUSG00000030737	6.637087295	6.61E-14	7.92E-15	6.10406894	1.64E-12	2.14E-13	0.0313	3.1187	2.1553
ENSMUSG00000030742	9.651477722	2.17E-56	5.78E-58	9.855175779	7.64E-58	2.02E-59	0.0997	80.1557	92.311
ENSMUSG00000030745	3.380495977	5.23E-21	4.1E-22	3.314638059	1.91E-19	1.66E-20	0.3307	3.4437	3.29
ENSMUSG00000030748	1.544049689	7.98E-22	6.01E-23	1.399103713	1.05E-26	6.51E-28	2.8413	8.2857	7.4937
ENSMUSG00000030761	1.410905485	3.85E-30	2.05E-31	1.307552892	1.21E-19	1.04E-20	5.507	14.6433	13.631
ENSMUSG00000030769	8.053473413	0.001441274	0.000498721	9.632995197	0.000624234	0.000198898	0	0.2657	0.794
ENSMUSG00000030780	-1.136278139	5.47E-13	7.03E-14	-1.165961157	2.94E-15	3.21E-16	11.2977	5.1397	5.035
ENSMUSG00000030782	1.706828046	0.049924298	0.023580084	2.130075489	0.001532947	0.000523577	0.1387	0.4527	0.607
ENSMUSG00000030785	-8.14849025	4.5E-14	5.33E-15	-9.244705565	3.47E-13	4.31E-14	17.5927	0.062	0.029
ENSMUSG00000030787	6.913306924	7.99E-10	1.33E-10	6.458668893	1.01E-08	1.81E-09	0.0143	1.7277	1.2607
ENSMUSG00000030788	2.13388076	2.32E-133	2.17E-135	1.839518903	1.06E-70	2.33E-72	11.1947	49.133	40.0647
ENSMUSG00000030824	-3.084244279	0	0	-3.190293442	3.23E-270	1.23E-272	404.162	47.6547	44.2773
ENSMUSG00000030830	1.372044137	8.66E-75	1.68E-76	1.602646607	1.27E-112	1.76E-114	25.2347	65.3167	76.6377
ENSMUSG00000030844	1.443651413	2.46E-15	2.69E-16	1.56702984	7.63E-22	5.84E-23	7.2677	19.7687	21.5337
ENSMUSG00000030851	-3.875999638	4E-15	4.42E-16	-3.663835109	3.61E-18	3.32E-19	5.3443	0.364	0.4217
ENSMUSG00000030879	1.146106751	1.57E-12	2.09E-13	1.01727713	2.94E-11	4.25E-12	23.255	51.467	47.0703
ENSMUSG00000030921	1.526026193	6.57E-31	3.4E-32	1.440184065	4.21E-29	2.39E-30	5.7987	16.6997	15.735
ENSMUSG00000030946	1.08767533	1.38E-10	2.14E-11	1.022665289	6.74E-11	1E-11	9.052	19.2383	18.3907
ENSMUSG00000031007	-1.42358338	1.93E-82	3.32E-84	-1.651049443	5.72E-79	1.14E-80	103.6227	38.629	32.9943
ENSMUSG00000031012	-2.656242868	1.69E-72	3.41E-74	-2.854926009	6.69E-145	6.98E-147	23.8623	3.7853	3.2983
ENSMUSG00000031026	6.629164862	2.83E-37	1.24E-38	6.355235166	6.2E-34	3.05E-35	0.0253	2.5077	2.074
ENSMUSG00000031028	5.040015679	0.011238334	0.004588238	4.129283017	0.035578481	0.016040285	0.0033	0.1097	0.0583
ENSMUSG00000031066	-1.441898396	1.17E-38	4.89E-40	-1.406581859	3.58E-55	1.01E-56	35.0447	12.8993	13.219

ENSMUSG00000031074	-8.809500194	0.0000727	0.0000207	-8.809500194	0.0000541	0.0000149	0.4487	0	0
ENSMUSG00000031075	-7.914883386	0.000977881	0.000328655	-7.914883386	0.000742002	0.000239547	0.2413	0	0
ENSMUSG00000031078	3.06638413	0.022413546	0.009765735	2.729038678	0.01151247	0.004630741	0.0487	0.4077	0.3227
ENSMUSG00000031103	-1.376837849	8.09E-28	4.66E-29	-1.293907128	1.16E-22	8.57E-24	16.1377	6.214	6.5817
ENSMUSG00000031133	-1.320328573	2.85E-28	1.62E-29	-1.462198076	6.86E-74	1.47E-75	40.7157	16.3043	14.7773
ENSMUSG00000031142	1.017760859	1.99E-09	3.42E-10	1.007545902	3.39E-10	5.41E-11	1.8433	3.7323	3.706
ENSMUSG00000031162	-2.146676471	4.01E-90	6.12E-92	-1.732139911	3.49E-33	1.75E-34	61.649	13.9223	18.5567
ENSMUSG00000031174	-1.452863185	1.68E-11	2.43E-12	-1.633543259	3.56E-11	5.18E-12	5.6977	2.0813	1.8363
ENSMUSG00000031309	-1.804492642	1.8E-102	2.29E-104	-1.938686703	1.34E-166	1.19E-168	68.9007	19.725	17.973
ENSMUSG00000031328	1.81763792	7.37E-183	4.51E-185	1.698683527	3.84E-223	2.09E-225	77.6887	273.8553	252.181
ENSMUSG00000031342	2.821890026	5.56E-10	9.09E-11	2.321375232	0.000006	0.00000145	0.174	1.2303	0.8697
ENSMUSG00000031355	1.810298207	8.03E-126	8.31E-128	1.776298453	1.87E-87	3.35E-89	19.4933	68.366	66.7737
ENSMUSG00000031391	-1.993184593	0.0000012	0.000000274	-2.293334588	7.44E-30	4.14E-31	7.7433	1.945	1.5797
ENSMUSG00000031445	1.341556902	0.001072122	0.000362861	1.526036519	0.000142818	0.0000415	0.4137	1.0483	1.1913
ENSMUSG00000031467	1.184492552	7.3E-29	4.12E-30	1.222400514	4.22E-25	2.79E-26	8.4887	19.2933	19.807
ENSMUSG00000031482	1.358560096	1.87E-15	2.02E-16	1.175001422	6.99E-11	1.04E-11	2.175	5.5773	4.911
ENSMUSG00000031497	2.46253929	3.02E-23	2.13E-24	2.61608392	2.47E-27	1.5E-28	1.335	7.3583	8.1847
ENSMUSG00000031503	-3.948367232	7.83E-08	1.57E-08	-2.955900904	0.000000348	7.23E-08	0.494	0.032	0.0637
ENSMUSG00000031511	3.106850301	0	0	3.462900074	7.42E-258	3.13E-260	12.967	111.7107	142.9803
ENSMUSG00000031530	6.216580753	0	0	6.394670523	0	0	1.0623	79.002	89.3817
ENSMUSG00000031555	1.651385116	1.29E-78	2.35E-80	1.568945066	3.87E-56	1.06E-57	6.9237	21.7497	20.5417
ENSMUSG00000031557	2.152790009	1.7E-65	3.82E-67	2.147756992	6.37E-64	1.55E-65	21.4597	95.4283	95.096
ENSMUSG00000031562	1.411922737	1.92E-12	2.58E-13	1.669326612	1.39E-23	9.95E-25	4.347	11.567	13.8263
ENSMUSG00000031570	-1.175030143	2.94E-24	1.97E-25	-1.121983698	1.26E-20	1.03E-21	37.298	16.5183	17.137
ENSMUSG00000031586	-1.836252927	3.33E-15	3.67E-16	-1.995960667	4.14E-35	1.95E-36	41.4917	11.6197	10.402
ENSMUSG00000031594	-10.25305956	0.00000311	0.000000742	-10.25305956	0.00000232	0.000000533	1.2203	0	0
ENSMUSG00000031616	-7.033423002	0.004516448	0.001703982	-4.448460501	0.01555819	0.006447173	0.131	0	0.006
ENSMUSG00000031628	-1.828646017	2.14E-18	1.94E-19	-1.97550438	4.55E-118	5.9E-120	341.411	96.117	86.8143
ENSMUSG00000031629	-1.223999916	0.000000939	0.000000212	-1.126849385	0.000000729	0.000000157	25.6423	10.9773	11.742
ENSMUSG00000031639	6.269282553	0.000026	0.00000695	6.057450272	0.0000441	0.0000119	0.005	0.3857	0.333
ENSMUSG00000031698	4.753575365	3.4E-71	6.94E-73	5.342826234	1.18E-98	1.9E-100	0.3667	9.891	14.8807
ENSMUSG00000031712	-5.243906746	9.61E-29	5.45E-30	-5.382597464	6.67E-31	3.58E-32	10.7493	0.2837	0.2577
ENSMUSG00000031714	-5.64385619	1.54E-14	1.76E-15	-5.876516947	1.05E-45	3.65E-47	3.9167	0.0783	0.0667
ENSMUSG00000031727	-1.670965019	0.0000998	0.000029	-1.375027836	0.002554047	0.000909011	1.296	0.407	0.4997
ENSMUSG00000031749	2.117529443	4.04E-38	1.7E-39	2.025835534	5.83E-52	1.78E-53	2.864	12.4283	11.663
ENSMUSG00000031750	9.64084744	0.0000014	0.000000324	10.72422961	1.02E-09	1.69E-10	0	0.7983	1.6917
ENSMUSG00000031751	-1.859931023	1.4E-126	1.42E-128	-1.556084408	1.27E-39	5.19E-41	85.8787	23.6587	29.205
ENSMUSG00000031760	-3.718818247	0.00000961	0.00000243	-3.959993219	0.00000459	0.0000011	3.4497	0.262	0.2217
ENSMUSG00000031762	-2.025990397	0.00000131	0.000000302	-2.711833024	2.83E-62	7.1E-64	221.6887	54.4327	33.8377
ENSMUSG00000031767	-1.552506765	1.66E-11	2.39E-12	-1.4240584	3.37E-15	3.7E-16	14.9733	5.1047	5.58
ENSMUSG00000031770	-1.950559333	1.93E-129	1.86E-131	-1.770211305	7.68E-108	1.13E-109	182.884	47.315	53.6153
ENSMUSG00000031778	1.98128774	0.0000192	0.00000504	2.645375755	1.19E-11	1.66E-12	0.291	1.149	1.8207
ENSMUSG00000031785	1.56587023	5.01E-36	2.25E-37	1.835956779	6.71E-47	2.29E-48	80.3167	237.7823	286.737
ENSMUSG00000031799	3.951179741	0	0	3.768159055	5.99E-227	3.19E-229	10.301	159.332	140.3487
ENSMUSG00000031822	-1.395848912	7.51E-30	4.04E-31	-1.11414041	7.1E-24	5.02E-25	54.8943	20.861	25.3593
ENSMUSG00000031824	7.294620749	0.000361725	0.000113556	7.992466327	0.000012	0.000003	0	0.157	0.2547
ENSMUSG00000031827	2.497759344	6.23E-239	2.56E-241	2.302623379	6.63E-113	9.06E-115	52.0017	293.7093	256.5523
ENSMUSG00000031860	-4.879814114	2.56E-12	3.46E-13	-4.278033995	3.26E-13	4.04E-14	3.906	0.1327	0.2013
ENSMUSG00000031865	-1.421979298	7.17E-98	9.67E-100	-1.323854091	5.77E-34	2.84E-35	117.057	43.6857	46.7603
ENSMUSG00000031877	-2.42634966	0	0	-2.243363101	2.55E-204	1.64E-206	183.3753	34.1143	38.7277
ENSMUSG00000031901	-1.345690794	7.44E-09	1.35E-09	-1.210814214	0.000000569	0.000000121	9.0597	3.5647	3.914
ENSMUSG00000031925	-2.083249944	6.82E-59	1.73E-60	-1.846100687	7.32E-61	1.88E-62	16.271	3.8397	4.5257
ENSMUSG00000031938	1.508404781	1.03E-26	6.25E-28	1.688713934	6.51E-31	3.48E-32	5.594	15.9147	18.0333
ENSMUSG00000031953	-1.584415818	3.13E-15	3.44E-16	-1.568381179	4.27E-18	3.95E-19	5.277	1.7597	1.7793
ENSMUSG00000031955	1.622308922	0.00000115	0.000000261	1.949650285	4.69E-10	7.58E-11	0.5127	1.5783	1.9803
ENSMUSG00000031967	-1.211826453	1.69E-49	5.13E-51	-1.11459408	2.63E-21	2.07E-22	41.4627	17.9003	19.1483
ENSMUSG00000031970	-1.21163259	0.001023663	0.000345414	-1.316335495	0.0000515	0.0000141	4.334	1.8713	1.7403
ENSMUSG00000031974	-1.228505789	1.2E-25	7.57E-27	-1.405995813	7.9E-49	2.56E-50	27.8533	11.8867	10.5107
ENSMUSG00000031995	-1.423531733	0.010851795	0.004420576	-2.222321253	5.15E-12	7.01E-13	2.2523	0.8397	0.4827
ENSMUSG00000031997	-3.849265514	2.05E-91	3.03E-93	-4.729746834	3.76E-10	6.02E-11	15.018	1.042	0.566
ENSMUSG00000032009	-2.372908917	4.87E-24	3.3E-25	-2.216216307	6.02E-29	3.44E-30	3.341	0.645	0.719
ENSMUSG00000032014	4.18882787	4.38E-35	2.03E-36	4.556996264	7.28E-12	1E-12	0.5223	9.526	12.2953
ENSMUSG00000032020	2.943818146	3.43E-221	1.57E-223	3.114972548	1.06E-286	3.73E-289	7.0747	54.4357	61.2923
ENSMUSG00000032035	-8.940503055	4.42E-46	1.47E-47	-11.37113741	9.74E-25	6.55E-26	20.3077	0.0413	0.0077
ENSMUSG00000032036	4.91082701	0.0000107	0.00000272	4.007371989	0.000613491	0.00019539	0.0203	0.6117	0.327
ENSMUSG00000032038	1.366600445	1.67E-31	8.52E-33	1.483077489	1.34E-47	4.51E-49	18.8327	48.5623	52.6457
ENSMUSG00000032047	1.284375142	1.66E-95	2.28E-97	1.253412709	6.83E-44	2.52E-45	27.4307	66.8147	65.396
ENSMUSG00000032051	-1.514305426	2.68E-14	3.12E-15	-1.554607901	1.89E-19	1.64E-20	31.557	11.047	10.7427
ENSMUSG00000032062	10.07992917	0.00000108	0.000000246	9.493188307	0.0000354	0.00000944	0	1.0823	0.7207
ENSMUSG00000032068	-8.112005026	0.005537904	0.002122446	-8.112005026	0.004294434	0.00159503	0.2767	0	0
ENSMUSG00000032101	1.230204997	1.26E-10	1.95E-11	1.310650987	1.3E-10	1.99E-11	1.447	3.3947	3.5893
ENSMUSG00000032109	1.01037397	2.58E-11	3.77E-12	1.114705366	1.44E-12	1.87E-13	3.164	6.3737	6.8517
ENSMUSG00000032115	-1.872629486	5.82E-80	1.04E-81	-1.666496784	6.53E-83	1.23E-84	211.0847	57.642	66.4953
ENSMUSG00000032177	1.558286379	1.63E-51	4.69E-53	1.863736892	4.32E-46	1.5E-47	5.2703	15.5213	19.1813
ENSMUSG00000032198	-2.579609266	0.0000287	0.00000771	-3.105678078	0.00000641	0.00000156	0.3587	0.06	0.0417
ENSMUSG00000032221	-3.243451558	8.05E-150	6.88E-152	-3.264999121	8.7E-146	8.83E-148	57.0697	6.026	5.9367
ENSMUSG00000032258	10.04894091	8.88E-11	1.36E-11	12.39615878	2.89E-09	4.95E-10	0	1.0593	5.3903
ENSMUSG00000032261	5.763765654	0.04894244	0.023030849	7.528779665	0.00017724	0.0000522	0	0.0543	0.1847
ENSMUSG00000032265	-5.393716726	0	0	-5.58537392	0	0	52.607	1.2513	1.0957
ENSMUSG00000032289	1.61939762	4.15E-32	2.09E-33	1.623198306	9.57E-35	4.56E-36	1.3983	4.2963	4.3077
ENSMUSG00000032297	-1.297107135	0.000185825	0.000056	-1.031868459	0.002869814	0.001029519	2.4107	0.981	1.179
ENSMUSG00000032298	-1.459358672	0.000000101	2.05E-08	-1.222197906	0.00000304	0.00000071	6.5923	2.3973	2.8257
ENSMUSG00000032322	3.1941196	2.64E-66	5.84E-68	2.811386256	2.3E-39	9.49E-41	2.893	26.4773	20.3077
ENSMUSG00000032323	-3.534326241	0	0	-3.30690338	0	0	281.6317	24.3077	28.458
ENSMUSG00000032340	1.151889317	2.59E-36	1.16E-37	1.238989061	1.56E-29	8.77E-31	7.8197	17.3757	18.457
ENSMUSG00000032348	-3.669145682	4.1E-54	1.12E-55	-4.004755434	7.33E-53	2.2E-54	21.6767	1.704	1.3503
ENSMUSG00000032353	-2.273535814	1.94E-128	1.9E-130	-2.3752357	7.93E-135	8.86E-137	104.7533	21.6653	20.1907
ENSMUSG00000032366	2.582759618	2.5E-163	1.81E-165	2.728537171	7.68E-204	5.04E-206	23.6677	141.7893	156.8653
ENSMUSG00000032373	1.919809736	2.66E-47	8.69E-49	2.0337867	3.21E-56	8.79E-58	3.7947	14.358	15.5383
ENSMUSG00000032380	-2.462729812	0.0000104	0.00000264	-2.441099796	0.0000215	0.00000558	1.8247	0.331	0.336
ENSMUSG00000032402	3.071569755	9.2E-124	9.71E-126	3.209156215	1.37E-146	1.38E-148	1.881	15.8133	17.3957
ENSMUSG00000032412	1.06688759	4.61E-64	1.07E-65	1.082632015	1.94E-56	5.29E-58	44.0387	92.257	93.2693
ENSMUSG00000032413	2.536119713	2.47E-88	3.93E-90	2.64385619	1.18E-148	1.15E-150	3.4747	20.154	21.7167
ENSMUSG00000032418	-1.965852894	2.73E-44	9.51E-46	-2.232994885	4.12E-64	9.94E-66	15.114	3.869	3.215
ENSMUSG00000032420	-3.494952335	0.004074715	0.0015277	-7.864186145	0.000038	0.0000102	0.233	0.0207	0
ENSMUSG00000032425	-1.383721428	1.38E-17	1.3E-18	-1.452665622	3.57E-16	3.68E-17	16.6637	6.386	6.088
ENSMUSG00000032436	-2.754873861	7.01E-09	1.27E-09	-3.337829174	0	0	378.9707	56.1443	37.4817
ENSMUSG00000032456	1.059020317	0.000000494	0.000000108	1.005390479	0.000086	0.0000242	2.5823	5.3803	5.184
ENSMUSG00000032462	1.251426724	7.88E-59	2E-60	1.335157527	1.42E-79	2.8E-81	16.8257	40.058	42.4517
ENSMUSG00000032468	-1.251494117	4.81E-42	1.81E-43	-1.227100666	9.85E-36	4.57E-37	26.1563	10.986	11.1733
ENSMUSG00000032479	-1.283358906	7.64E-45	2.64E-46	-1.372518005	2.08E-41	8.18E-43	44.406	18.2437	17.1503
ENSMUSG00000032489	-1.7125494	0.000344616	0.000107874	-2.137570988	0.000183502	0.0000541	1.4257	0.435	0.324
ENSMUSG00000032492	-1.35938253	0.039322259	0.018054295	-1.178519683	0.013564666	0.005538624	1.2487	0.4867	0.5517
ENSMUSG00000032508	1.300620747	6.39E-34	3.02E-35	1.283996437	1.86E-31	9.82E-33	14.1377	34.826	34.427
ENSMUSG00000032554	-2.083359103	4.14E-58	1.07E-59	-2.379106606	7.22E-161	6.43E-163	90.5433	21.365	17.405
ENSMUSG00000032556	1.588676004	1.99E-09	3.41E-10	1.369713509	1.54E-13	1.87E-14	3.492	10.503	9.024
ENSMUSG00000032575	-1.107954706	9.42E-50	2.82E-51	-1.037111628	2.25E-38	9.58E-40	250.3697	116.1593	122.0057
ENSMUSG00000032584	-4.001390818	5.56E-16	5.76E-17	-4.398726315	3.57E-21	2.85E-22	1.7297	0.108	0.082
ENSMUSG00000032595	2.55672882	0.011980391	0.004920271	2.659428994	0.026080521	0.011394472	0.043	0.253	0.2717
ENSMUSG00000032596	3.052966508	2.19E-95	3.03E-97	3.177669121	6.51E-71	1.43E-72	1.7203	14.2773	15.5663
ENSMUSG00000032606	-1.143364175	1.62E-09	2.76E-10	-1.343665151	3.41E-11	4.95E-12	9.7187	4.3997	3.8293
ENSMUSG00000032607	-1.830740295	0.000966643	0.000324677	-2.228140716	0.0000799	0.0000224	1.2853	0.3613	0.2743
ENSMUSG00000032643	2.668660323	2.17E-90	3.29E-92	2.743540677	4.48E-112	6.32E-114	6.48	41.2023	43.3973
ENSMUSG00000032661	3.958751714	1.08E-32	5.32E-34	4.10003479	2.39E-52	7.24E-54	0.289	4.4937	4.956
ENSMUSG00000032690	11.47910658	5.46E-15	6.11E-16	11.5199638	3.15E-15	3.45E-16	0	2.8547	2.9367
ENSMUSG00000032698	3.309146294	7.55E-180	4.77E-182	3.348056779	7.02E-120	8.88E-122	12.127	120.2003	123.4863
ENSMUSG00000032715	-2.457391337	1.23E-40	4.83E-42	-2.289525444	4E-45	1.43E-46	28.382	5.1677	5.8053
ENSMUSG00000032741	1.321100566	1.86E-33	8.95E-35	1.466856568	1.02E-34	4.91E-36	11.3933	28.467	31.4933
ENSMUSG00000032750	-1.323494369	0.0000135	0.00000349	-1.622644846	0.00000102	0.000000225	3.84	1.5343	1.247
ENSMUSG00000032850	-4.271103836	0.022363263	0.009737613	-2.596504123	0.016275408	0.006785915	0.1673	0.0087	0.0277
ENSMUSG00000032855	-1.00872037	0.007381553	0.00290647	-1.017117745	0.008992843	0.003550198	6.4717	3.2163	3.1977
ENSMUSG00000032890	-2.519960939	9.5E-14	1.15E-14	-2.645874245	7.26E-22	5.56E-23	1.9673	0.343	0.3143
ENSMUSG00000032902	1.360971457	2.06E-41	7.89E-43	1.3820295	1.74E-54	5E-56	14.4307	37.0663	37.6113
ENSMUSG00000032905	-2.481641926	1.52E-77	2.82E-79	-2.606813731	3.63E-119	4.64E-121	52.355	9.3737	8.5947
ENSMUSG00000032913	1.544320516	9.29E-36	4.22E-37	1.642262754	5.63E-37	2.51E-38	4.3	12.5417	13.4227
ENSMUSG00000032965	-1.778880541	0.001117764	0.000379628	-2.063446327	5.7E-47	1.95E-48	20.895	6.089	4.999
ENSMUSG00000032966	1.651244561	7.49E-126	7.7E-128	1.688664492	5.99E-116	7.94E-118	56.8353	178.5223	183.2133
ENSMUSG00000033007	4.950312876	0.003878667	0.001449616	4.497186541	0.013215988	0.005383481	0.008	0.2473	0.1807
ENSMUSG00000033022	-2.151029895	0.000043	0.0000119	-2.21507395	0.0000442	0.000012	2.2503	0.5067	0.4847
ENSMUSG00000033029	-3.001514641	0.00319934	0.001176166	-3.93285828	0.000016	0.00000408	1.5883	0.1983	0.104
ENSMUSG00000033107	-1.470383914	3.37E-35	1.55E-36	-1.691642193	2.75E-49	8.82E-51	53.243	19.2147	16.4827
ENSMUSG00000033191	3.392604471	3.16E-280	1.03E-282	3.428251357	0	0	6.222	65.344	66.9787
ENSMUSG00000033207	5.785616532	5.18E-232	2.23E-234	5.700288287	1.37E-211	8.32E-214	0.5497	30.321	28.5797
ENSMUSG00000033233	-2.655597686	4.27E-44	1.5E-45	-3.27573411	2.16E-61	5.51E-63	14.4757	2.2973	1.4947
ENSMUSG00000033276	-1.498906436	0.001313604	0.00045034	-1.872414362	0.00000534	0.00000129	0.6237	0.2207	0.1703
ENSMUSG00000033306	2.336347359	2.19E-99	2.86E-101	2.119040677	2.31E-89	4.06E-91	2.084	10.5247	9.053
ENSMUSG00000033318	-1.751639366	0.000016	0.00000414	-2.061247189	0.00000133	0.000000299	3.272	0.9717	0.784
ENSMUSG00000033326	-1.434277004	1.2E-80	2.1E-82	-1.443593981	1.31E-47	4.38E-49	50.0983	18.538	18.4187
ENSMUSG00000033327	-1.61643704	5.56E-08	0.000000011	-1.511062354	6.67E-08	1.29E-08	0.9577	0.3123	0.336
ENSMUSG00000033386	1.227912356	1.31E-43	4.7E-45	1.188518948	5.91E-58	1.56E-59	23.4527	54.9327	53.453
ENSMUSG00000033446	1.539137785	8.7E-19	7.78E-20	1.592114548	3.58E-20	2.98E-21	1.2297	3.5737	3.7073
ENSMUSG00000033467	-1.518501611	3.18E-11	4.69E-12	-1.301814839	3.47E-10	5.55E-11	10.542	3.6797	4.276
ENSMUSG00000033486	-2.256894209	0.010669985	0.004337669	-1.881136698	0.013040316	0.005303818	0.5783	0.121	0.157
ENSMUSG00000033545	-1.912114538	2.87E-18	2.62E-19	-1.996889091	6.21E-44	2.28E-45	31.6557	8.411	7.931
ENSMUSG00000033557	-1.596265262	3.08E-55	8.35E-57	-1.481923979	3.58E-45	1.27E-46	24.1333	7.9817	8.64
ENSMUSG00000033581	-1.336078424	0.0000579	0.0000163	-1.959344812	1.55E-11	2.19E-12	4.047	1.603	1.0407
ENSMUSG00000033590	-2.17042429	0.000143073	0.0000425	-2.152557869	0.0000185	0.00000476	0.4817	0.107	0.1083

ENSMUSG00000033623	1.156702682	3.62E-23	2.56E-24	1.150735199	1.85E-33	9.19E-35	2.789	6.218	6.1923
ENSMUSG00000033701	-1.280170084	9.78E-20	8.23E-21	-1.044947899	4.36E-08	8.32E-09	74.0447	30.4877	35.8867
ENSMUSG00000033720	-1.232081478	0.010931351	0.004455313	-1.096848127	0.006501736	0.002494936	0.996	0.424	0.4657
ENSMUSG00000033722	3.411884792	1.31E-18	1.18E-19	3.587612079	8.28E-23	6.09E-24	0.1813	1.93	2.18
ENSMUSG00000033730	8.722921349	9.54E-12	1.35E-12	9.147883282	1.04E-12	1.33E-13	0.005	2.1127	2.8363
ENSMUSG00000033857	-1.50037817	8.67E-13	1.13E-13	-1.471866814	1.66E-09	2.79E-10	6.0007	2.121	2.1633
ENSMUSG00000033862	-1.261953564	2.84E-17	2.74E-18	-1.331473421	1.03E-19	8.78E-21	24.9957	10.4227	9.9323
ENSMUSG00000033955	-1.331997353	0.010851928	0.004421435	-1.200264763	0.000101485	0.0000289	1.5097	0.5997	0.657
ENSMUSG00000033967	1.00383951	0.000238026	0.0000727	1.153059978	0.00000751	0.00000184	1.0007	2.0067	2.2253
ENSMUSG00000033985	-1.237733824	8.16E-09	1.49E-09	-1.44929651	6.63E-11	9.87E-12	5.0947	2.1603	1.8657
ENSMUSG00000034006	1.146298994	4.77E-14	5.67E-15	1.316934925	5.14E-14	6.06E-15	8.1813	18.109	20.3827
ENSMUSG00000034041	3.396386977	9.91E-305	2.82E-307	3.590937467	3.41E-297	1.11E-299	10.5753	111.3547	127.4307
ENSMUSG00000034066	2.760812336	0.004296419	0.001615597	2.88400088	0.002765316	0.000990504	0.048	0.3253	0.3543
ENSMUSG00000034101	1.09744088	4E-50	1.19E-51	1.10771916	5.36E-53	1.6E-54	16.1887	34.6397	34.8873
ENSMUSG00000034107	2.057304526	0.015583368	0.006553783	2.400069924	0.005112646	0.001923773	0.109	0.4537	0.5753
ENSMUSG00000034121	-1.892977022	2.73E-18	2.48E-19	-2.177607794	2.35E-34	1.14E-35	10.1987	2.746	2.2543
ENSMUSG00000034165	2.809112823	0	0	2.909091405	0	0	39.4473	276.468	296.3067
ENSMUSG00000034175	-1.157220803	2.74E-08	5.23E-09	-1.088698093	2.26E-08	4.19E-09	13.3883	6.003	6.295
ENSMUSG00000034177	-2.031239062	1.9E-09	3.25E-10	-3.017404482	6.08E-27	3.76E-28	3.2523	0.7957	0.4017
ENSMUSG00000034187	1.601098337	1.02E-83	1.74E-85	1.622758015	2.31E-157	2.11E-159	30.9507	93.8963	95.3167
ENSMUSG00000034224	-6.763765654	0.036434621	0.016611353	-6.763765654	0.03028883	0.013412908	0.1087	0	0
ENSMUSG00000034235	1.001128428	0.000485365	0.00015527	1.04628573	0.0000116	0.0000029	1.278	2.558	2.6393
ENSMUSG00000034243	-1.012777893	3.79E-45	1.3E-46	-1.147085549	1.77E-32	9.01E-34	28.843	14.2943	13.0237
ENSMUSG00000034353	-2.92967796	0.006544314	0.002547259	-4.558703345	2.65E-105	4E-107	22.1217	2.9033	0.9387
ENSMUSG00000034361	-1.151329953	3.17E-42	1.18E-43	-1.051571989	1.76E-24	1.2E-25	88.09	39.659	42.4983
ENSMUSG00000034371	-1.347478492	4.13E-18	3.82E-19	-1.094162252	4.91E-12	6.67E-13	17.3937	6.8353	8.1473
ENSMUSG00000034401	-2.895726465	9.1E-25	6.01E-26	-3.273456351	1.23E-23	8.77E-25	5.4923	0.738	0.568
ENSMUSG00000034430	-1.327802256	1.74E-11	2.5E-12	-1.000058918	8.7E-11	1.31E-11	8.1623	3.2517	4.081
ENSMUSG00000034459	3.314065977	0.01179609	0.004835618	3.236326227	0.007922426	0.003090425	0.0307	0.305	0.289
ENSMUSG00000034480	-1.586590793	9E-23	6.48E-24	-1.763336661	4.81E-32	2.51E-33	8.5697	2.8533	2.5243
ENSMUSG00000034570	-1.298368448	3.31E-09	5.82E-10	-1.403936901	1.6E-10	2.48E-11	5.7397	2.3337	2.169
ENSMUSG00000034574	-1.350819049	5.17E-35	2.4E-36	-1.074037984	1.35E-12	1.75E-13	12.1807	4.7757	5.7857
ENSMUSG00000034579	-1.67354334	0.002576312	0.000931911	-2.39712277	0.0000474	0.0000129	1.1027	0.3457	0.2093
ENSMUSG00000034584	-1.953962429	0.00834102	0.003323081	-1.390914705	0.049740546	0.023307725	0.257	0.0663	0.098
ENSMUSG00000034595	1.660486057	4.01E-103	5.02E-105	1.825847911	5.42E-143	5.73E-145	23.2173	73.3953	82.309
ENSMUSG00000034613	-1.44609188	7.86E-19	7.02E-20	-1.409359329	3.75E-28	2.2E-29	7.6073	2.792	2.864
ENSMUSG00000034614	-1.813649021	0.00000398	0.000000962	-1.622882501	2.18E-13	2.68E-14	5.628	1.601	1.8273
ENSMUSG00000034647	-2.962450262	3.01E-146	2.66E-148	-3.327867421	6.82E-255	2.97E-257	38.7827	4.9757	3.8623
ENSMUSG00000034663	2.591967166	8.95E-42	3.4E-43	2.842597652	0	0	11.8483	71.436	84.9893
ENSMUSG00000034664	3.502504068	0	0	3.760933514	0	0	34.154	387.0797	463.0153
ENSMUSG00000034685	1.196746698	0.000000145	2.98E-08	1.586898505	1.9E-18	1.72E-19	2.1513	4.9313	6.4627
ENSMUSG00000034714	-2.1556984	1.58E-15	1.71E-16	-2.447590268	3.39E-22	2.56E-23	4.995	1.121	0.9157
ENSMUSG00000034723	-1.298668455	2.58E-46	8.5E-48	-1.272559111	1.31E-49	4.16E-51	22.765	9.254	9.423
ENSMUSG00000034738	2.13150328	1.15E-17	1.08E-18	2.05010606	2.82E-18	2.58E-19	1.818	7.966	7.529
ENSMUSG00000034744	-1.024853895	3.85E-14	4.53E-15	-1.210739103	4.25E-19	3.73E-20	28.1813	13.85	12.1757
ENSMUSG00000034758	2.143046866	4.66E-18	4.32E-19	2.009561528	1.39E-16	1.4E-17	1.4287	6.3103	5.7527
ENSMUSG00000034765	1.256182575	0.00000152	0.000000352	1.585624926	2.99E-15	3.26E-16	3.629	8.6683	10.892
ENSMUSG00000034771	1.11450748	0.028725701	0.012759439	1.278430929	0.002041603	0.000712672	0.8777	1.9003	2.129
ENSMUSG00000034793	-1.762610656	1.27E-33	6.09E-35	-1.664981994	3.43E-26	2.16E-27	36.7033	10.817	11.5743
ENSMUSG00000034801	-1.734002398	1.54E-111	1.79E-113	-1.780781227	2.65E-105	4.01E-107	38.8557	11.6807	11.308
ENSMUSG00000034818	-1.84636027	0.022237006	0.009672087	-3.014813884	0.003150312	0.00113928	0.2937	0.0817	0.0363
ENSMUSG00000034845	-1.784091612	0.016282981	0.006870637	-2.256579384	0.002115278	0.000741603	0.892	0.259	0.1867
ENSMUSG00000034868	-1.045848531	2.65E-19	2.27E-20	-1.0859921	6.15E-37	2.76E-38	165.366	80.0967	77.8987
ENSMUSG00000034923	11.30206767	8.14E-09	1.49E-09	12.34992621	1.06E-12	1.36E-13	0	2.525	5.2203
ENSMUSG00000034930	1.2674054	1.11E-35	5.04E-37	1.204103569	5.05E-28	2.96E-29	14.0247	33.7613	32.312
ENSMUSG00000034949	-1.686146346	0.000000681	0.000000152	-2.904352186	0.000000172	3.48E-08	1.2303	0.3823	0.1643
ENSMUSG00000034993	-1.306263	3.74E-49	1.15E-50	-1.244500644	7.28E-54	2.12E-55	110.0997	44.5207	46.468
ENSMUSG00000035047	1.175069054	4.54E-33	2.22E-34	1.200705909	1.82E-35	8.47E-37	11.6753	26.3633	26.836
ENSMUSG00000035049	1.059429793	2.94E-20	2.41E-21	1.077695978	2.74E-23	1.98E-24	13.13	27.3643	27.713
ENSMUSG00000035064	-3.247700484	9.62E-22	7.28E-23	-3.732951597	3.58E-264	1.41E-266	57.1873	6.0207	4.301
ENSMUSG00000035085	-4.294896919	4.46E-16	4.6E-17	-4.312584452	6.33E-17	6.23E-18	8.0543	0.4103	0.4053
ENSMUSG00000035126	-1.872166332	0.002483261	0.000895492	-1.17757934	0.042250318	0.019392441	0.8347	0.228	0.369
ENSMUSG00000035151	-1.163066986	3.65E-13	4.63E-14	-1.108028062	1.64E-10	2.54E-11	9.444	4.2173	4.3813
ENSMUSG00000035158	1.609333154	5.37E-40	2.15E-41	1.530865931	4.12E-35	1.94E-36	3.5807	10.925	10.3467
ENSMUSG00000035165	-5.997795927	3.73E-18	3.42E-19	-12.85577692	1.18E-13	1.42E-14	7.4127	0.116	0
ENSMUSG00000035168	4.064822001	0.0000657	0.0000186	5.386242908	1.42E-10	2.19E-11	0.0113	0.1897	0.474
ENSMUSG00000035172	-1.238472621	3.33E-08	6.41E-09	-1.169278115	1.48E-08	2.7E-09	5.2027	2.205	2.3133
ENSMUSG00000035177	-7.285402219	0.001819844	0.000640959	-7.285402219	0.001392309	0.000471697	0.156	0	0
ENSMUSG00000035183	4.124071622	8.8E-263	3.12E-265	4.500504429	1.19E-41	4.65E-43	4.1627	72.584	94.2233
ENSMUSG00000035200	-6.303780748	0.049573598	0.02337656	-6.303780748	0.041691903	0.019100635	0.079	0	0
ENSMUSG00000035311	-1.254893792	1.19E-47	3.84E-49	-1.271179005	2.87E-31	1.52E-32	27.498	11.5223	11.393
ENSMUSG00000035329	-1.710842107	2.23E-41	8.58E-43	-1.627092486	1.97E-45	6.9E-47	23.697	7.239	7.6717
ENSMUSG00000035441	7.963859408	0.00000868	0.00000219	8.647458426	0.000000189	3.82E-08	0	0.2497	0.401
ENSMUSG00000035504	-1.490665204	2.48E-13	3.1E-14	-1.329413616	2.73E-10	4.31E-11	9.3073	3.312	3.7037
ENSMUSG00000035513	-1.844935545	0.000000866	0.000000195	-2.524192473	1.26E-15	1.34E-16	4.9587	1.3803	0.862
ENSMUSG00000035547	4.271072484	4.51E-20	3.71E-21	3.68787508	1.03E-27	6.12E-29	0.1833	3.5397	2.3627
ENSMUSG00000035615	-4.604862058	0.015662716	0.00658933	-6.604862058	0.002836439	0.001016763	0.0973	0.004	0
ENSMUSG00000035638	6.582476944	3.71E-58	9.56E-60	6.515525827	8.83E-225	4.75E-227	0.589	56.4467	53.887
ENSMUSG00000035678	1.110900154	7.76E-09	1.41E-09	1.289146779	2.31E-11	3.32E-12	5.7717	12.4657	14.105
ENSMUSG00000035697	1.983664228	2.59E-155	1.98E-157	2.182995509	2.08E-210	1.28E-212	22.167	87.6697	100.6593
ENSMUSG00000035713	-1.1042616	0.005957052	0.002297158	-1.15573322	0.003622059	0.001324887	0.961	0.447	0.4313
ENSMUSG00000035783	3.153167398	6.59E-146	5.91E-148	2.173873145	1.18E-37	5.12E-39	5.1763	46.049	23.3573
ENSMUSG00000035845	-1.420186191	6.03E-23	4.31E-24	-1.440262772	8.61E-24	6.12E-25	20.398	7.622	7.5167
ENSMUSG00000035863	-3.057458611	3.25E-17	3.16E-18	-3.26834645	5.37E-121	6.71E-123	33.1587	3.983	3.4413
ENSMUSG00000035868	-1.326095156	0.004592172	0.001735584	-1.582586716	0.000148236	0.0000432	2.427	0.968	0.8103
ENSMUSG00000035967	-1.473093519	3.44E-48	1.1E-49	-1.290402659	7.91E-33	4.01E-34	85.8973	30.941	35.118
ENSMUSG00000035969	3.674879137	0.0000122	0.00000314	3.45900984	1.98E-13	2.42E-14	0.1037	1.324	1.14
ENSMUSG00000036052	-2.676148148	4.9E-22	3.66E-23	-3.247529534	4.99E-37	2.22E-38	6.645	1.0397	0.6997
ENSMUSG00000036067	-2.520994016	0.000100843	0.0000293	-5.229084601	8.03E-16	8.44E-17	2.713	0.4727	0.0723
ENSMUSG00000036091	-1.19498065	0.013934446	0.005795452	-1.986413732	0.000517947	0.000162994	2.149	0.9387	0.5423
ENSMUSG00000036109	-1.067757157	6.4E-20	5.32E-21	-1.182196726	4.62E-24	3.23E-25	14.0003	6.679	6.1697
ENSMUSG00000036158	-3.048853452	6.71E-30	3.61E-31	-2.995934923	1.37E-38	5.8E-40	4.7253	0.571	0.5923
ENSMUSG00000036181	-1.833880054	3.33E-45	1.14E-46	-1.404045137	3.11E-18	2.84E-19	31.5533	8.851	11.923
ENSMUSG00000036249	1.150518181	1.11E-29	6.01E-31	1.148157689	6.26E-29	3.58E-30	12.2157	27.118	27.0737
ENSMUSG00000036257	-2.156740094	7.01E-127	7.06E-129	-2.031437812	1.37E-115	1.83E-117	73.4853	16.48	17.9753
ENSMUSG00000036295	-6.634782245	1.64E-10	2.57E-11	-8.949097156	1.29E-39	5.28E-41	12.521	0.126	0.0253
ENSMUSG00000036330	-2.606657572	0.033099408	0.014959691	-4.48112669	0.003187737	0.001154795	0.268	0.044	0.012
ENSMUSG00000036377	-3.32033887	2.93E-102	3.78E-104	-3.469378587	4.94E-97	8.02E-99	14.2143	1.423	1.2833
ENSMUSG00000036381	1.51877823	8.91E-44	3.17E-45	1.36923584	1.27E-24	8.63E-26	15.2793	43.7827	39.4717
ENSMUSG00000036390	-3.921575106	7.65E-164	5.42E-166	-3.941634378	2.13E-189	1.51E-191	89.9913	5.9387	5.8567
ENSMUSG00000036402	-1.77534138	6.54E-48	2.09E-49	-2.07100408	4.94E-135	5.49E-137	171.5087	50.102	40.818
ENSMUSG00000036452	-1.702301531	4.14E-22	3.07E-23	-1.580987618	2.64E-40	1.06E-41	23.197	7.1283	7.7537
ENSMUSG00000036461	1.680766093	4.53E-197	2.51E-199	1.621294112	1.35E-93	2.26E-95	48.4607	155.364	149.0897
ENSMUSG00000036478	-1.722212748	4.93E-20	4.08E-21	-1.638873038	3.93E-44	1.43E-45	12.1507	3.6827	3.9017
ENSMUSG00000036534	-1.006729349	1.1E-11	1.55E-12	-1.135930397	3.14E-16	3.22E-17	12.8933	6.4167	5.867
ENSMUSG00000036537	-1.868321806	0.000000509	0.000000111	-1.478617449	0.0000661	0.0000184	4.5797	1.2543	1.6433
ENSMUSG00000036585	2.983342656	0.015354423	0.006445765	3.119999776	0.001231565	0.000412137	0.0327	0.2583	0.284
ENSMUSG00000036587	-3.392089922	1.12E-86	1.84E-88	-4.768561848	1.36E-112	1.9E-114	23.2503	2.2147	0.853
ENSMUSG00000036599	-1.649034637	5.46E-38	2.31E-39	-1.469557423	1.38E-22	1.02E-23	26.6653	8.5023	9.6287
ENSMUSG00000036622	1.78997546	6.28E-68	1.34E-69	1.948046795	1.76E-61	4.48E-63	7.4803	25.8677	28.863
ENSMUSG00000036634	1.570785448	1.88E-11	2.72E-12	1.596356005	9.12E-13	1.16E-13	1.6817	4.9957	5.085
ENSMUSG00000036644	-2.34216778	9.79E-109	1.17E-110	-2.402665859	4.87E-199	3.33E-201	104.9927	20.706	19.8557
ENSMUSG00000036687	2.5070395	0.020838264	0.009008441	3.25244017	0.000337786	0.000103243	0.056	0.3183	0.5337
ENSMUSG00000036718	3.977347785	9.67E-199	5.31E-201	4.180783553	9.93E-255	4.39E-257	2.0247	31.89	36.7193
ENSMUSG00000036743	1.361237368	7.95E-18	7.46E-19	1.296943082	1.39E-14	1.57E-15	6.799	17.467	16.7057
ENSMUSG00000036820	-1.12428857	1.31E-08	2.44E-09	-1.514033285	1.06E-15	1.12E-16	13.613	6.2447	4.7663
ENSMUSG00000036854	-1.370406995	0.000000673	0.00000015	-1.930869038	1.61E-11	2.28E-12	6.3903	2.4717	1.676
ENSMUSG00000036902	-2.645456726	1.29E-88	2.05E-90	-2.391912862	2.36E-66	5.52E-68	12.7767	2.042	2.4343
ENSMUSG00000036931	2.109717612	7.03E-12	9.85E-13	2.128924539	2.08E-12	2.74E-13	0.8817	3.8053	3.8563
ENSMUSG00000036955	-1.932242762	3.62E-73	7.19E-75	-2.023958529	7.3E-89	1.3E-90	35.277	9.2433	8.674
ENSMUSG00000036986	1.421734197	2.83E-78	5.19E-80	1.18325504	1.04E-49	3.3E-51	11.0243	29.535	25.035
ENSMUSG00000037003	2.673981647	4.28E-14	5.06E-15	3.246010542	1.17E-17	1.1E-18	0.2907	1.855	2.7577
ENSMUSG00000037098	-1.262232813	2.39E-12	3.23E-13	-1.364698915	2.87E-15	3.13E-16	5.038	2.1003	1.9563
ENSMUSG00000037103	-1.187408764	7.64E-24	5.26E-25	-1.031831248	1.2E-12	1.55E-13	26.8077	11.771	13.1113
ENSMUSG00000037108	-1.638255974	3.77E-26	2.34E-27	-1.640908363	6.34E-22	4.82E-23	14.6877	4.7183	4.7097
ENSMUSG00000037169	2.64730103	3.38E-19	2.93E-20	3.185495306	8.08E-11	1.21E-11	1.082	6.7787	9.8437
ENSMUSG00000037221	-1.598327698	1.34E-09	2.26E-10	-1.227823654	0.00000153	0.000000347	16.4113	5.42	7.007
ENSMUSG00000037235	-1.338811396	3.79E-17	3.7E-18	-1.3649901	3.75E-37	1.65E-38	23.866	9.4353	9.2657
ENSMUSG00000037286	1.588900805	2.35E-114	2.66E-116	1.51794937	1.23E-55	3.43E-57	22.8843	68.8407	65.537
ENSMUSG00000037287	-1.461272181	2.21E-62	5.25E-64	-1.479820748	4.44E-48	1.47E-49	28.955	10.5157	10.3813
ENSMUSG00000037336	2.199796982	1.48E-09	2.51E-10	2.563561106	5.63E-16	5.88E-17	0.6037	2.7733	3.5687
ENSMUSG00000037348	3.433248248	3.22E-104	3.99E-106	3.63326066	2.87E-93	4.83E-95	2.2813	24.6433	28.308
ENSMUSG00000037410	-1.009501319	9.86E-23	7.11E-24	-1.260619061	1.4E-35	6.5E-37	15.4373	7.668	6.443
ENSMUSG00000037434	1.03631181	2.27E-15	2.47E-16	1.559380364	1.73E-27	1.04E-28	4.381	8.9853	12.912
ENSMUSG00000037446	1.521249631	6.06E-43	2.22E-44	1.690334324	1.75E-54	5.05E-56	7.5563	21.6897	24.3867
ENSMUSG00000037447	-1.76185062	4.27E-11	6.37E-12	-1.43065063	1.58E-13	1.92E-14	14.1317	4.167	5.2423
ENSMUSG00000037463	-1.820353411	0.01432881	0.005982365	-2.275981691	0.001093918	0.000363204	0.9477	0.2683	0.1957
ENSMUSG00000037470	-1.179758513	4.67E-46	1.56E-47	-1.074496618	6.99E-30	3.88E-31	62.823	27.7317	29.8307
ENSMUSG00000037572	1.086086459	1.31E-47	4.22E-49	1.076790848	1.26E-34	6.09E-36	12.8103	27.196	27.0213
ENSMUSG00000037580	4.126222573	0	0	4.12048775	0	0	5.2493	91.6687	91.305
ENSMUSG00000037628	1.600060388	3.23E-14	3.8E-15	1.483394456	4.32E-13	5.41E-14	5.0063	15.177	13.998
ENSMUSG00000037661	-3.881088762	2.33E-11	3.39E-12	-4.36923381	1.25E-11	1.75E-12	2.0873	0.1417	0.101
ENSMUSG00000037679	1.886014757	3E-68	6.32E-70	2.170714102	3.71E-87	6.68E-89	9.4097	34.7793	42.3667
ENSMUSG00000037685	-1.183988221	9.35E-16	9.86E-17	-1.070047632	2.31E-11	3.32E-12	17.731	7.804	8.4453
ENSMUSG00000037706	2.257804694	1.17E-27	6.8E-29	1.991275384	3.06E-21	2.42E-22	3.1513	15.0717	12.5293
ENSMUSG00000037722	1.366396353	1.45E-32	7.18E-34	1.32996693	3.77E-27	2.3E-28	9.867	25.4397	24.8053
ENSMUSG00000037725	1.053701659	3.45E-29	1.91E-30	1.068952185	6.49E-24	4.57E-25	15.9263	33.0607	33.412

ENSMUSG00000037731	-1.575574439	0.0000167	0.00000433	-1.781180565	0.00000268	0.000000621	2.3487	0.788	0.6833
ENSMUSG00000037752	1.465268692	0.0000341	0.00000928	1.440170782	0.0000185	0.00000475	0.441	1.2177	1.1967
ENSMUSG00000037754	2.811315652	3.09E-49	9.5E-51	3.067553563	1.35E-51	4.17E-53	0.485	3.4043	4.066
ENSMUSG00000037818	1.246244233	3.17E-15	3.48E-16	1.216960938	1.18E-09	1.96E-10	3.7763	8.9583	8.7783
ENSMUSG00000037820	5.831801553	0	0	5.660011318	0	0	1.2717	72.4303	64.2993
ENSMUSG00000037822	-1.073372724	2.53E-11	3.7E-12	-1.3404945	1.41E-12	1.83E-13	27.1243	12.8897	10.711
ENSMUSG00000037824	2.22685643	2.27E-54	6.17E-56	2.279738079	1.27E-92	2.17E-94	7.076	33.1237	34.3603
ENSMUSG00000037833	9.22881869	0.000000861	0.000000194	9.074141463	0.00000248	0.000000574	0	0.6	0.539
ENSMUSG00000037868	3.646341453	1.03E-11	1.45E-12	5.104192036	2.05E-38	8.72E-40	0.1547	1.9367	5.32
ENSMUSG00000037890	-1.041903953	0.00000516	0.00000127	-1.387855961	2.45E-08	4.57E-09	3.3767	1.64	1.2903
ENSMUSG00000037913	-3.222512938	1.27E-08	2.36E-09	-3.995075227	4.39E-139	4.76E-141	41.235	4.4177	2.586
ENSMUSG00000037922	1.498217462	1.03E-53	2.85E-55	1.350229038	1.04E-34	4.99E-36	9.8097	27.7117	25.01
ENSMUSG00000037940	-3.465593822	2.05E-21	1.58E-22	-3.105441778	2.55E-25	1.67E-26	31.5173	2.853	3.662
ENSMUSG00000037946	2.194595241	3.35E-42	1.25E-43	2.229693756	2.15E-46	7.41E-48	2.1023	9.6237	9.8607
ENSMUSG00000037960	1.653451443	1.34E-27	7.84E-29	1.398290393	4.5E-19	3.97E-20	19.723	62.0457	51.9877
ENSMUSG00000037966	2.441471713	5.47E-59	1.38E-60	2.376854731	3.1E-53	9.14E-55	6.5007	35.3113	33.7647
ENSMUSG00000038025	-1.103467115	6.52E-23	4.68E-24	-1.027581259	4.47E-13	5.6E-14	11.7763	5.4807	5.7767
ENSMUSG00000038028	1.204779845	0.00000216	0.00000051	1.118538591	0.000000524	0.000000111	2.39	5.509	5.1893
ENSMUSG00000038037	2.902999305	0.000000182	0.000000038	2.956075432	0.000000561	0.000000119	0.132	0.9873	1.0243
ENSMUSG00000038065	-1.060371162	0.000505476	0.000162265	-1.315043738	0.000000633	0.000000136	2.8223	1.3533	1.1343
ENSMUSG00000038068	-6.198121893	1.35E-16	1.36E-17	-6.124121312	3.71E-18	3.42E-19	1.86	0.0253	0.0267
ENSMUSG00000038074	-2.321785176	2.76E-14	3.22E-15	-2.654651661	9.41E-18	8.84E-19	3.3647	0.673	0.5343
ENSMUSG00000038172	2.370517202	1.98E-118	2.19E-120	2.599932507	1.1E-120	1.38E-122	3.7817	19.556	22.9267
ENSMUSG00000038205	2.136550247	3.66E-17	3.57E-18	2.186996603	2.37E-23	1.71E-24	0.9863	4.337	4.4913
ENSMUSG00000038212	-1.047462173	7.65E-31	3.97E-32	-1.127708774	5.78E-27	3.57E-28	51.0253	24.687	23.3513
ENSMUSG00000038213	-2.310987291	6.59E-21	5.19E-22	-1.874043506	4E-15	4.4E-16	5.6487	1.1383	1.541
ENSMUSG00000038235	1.042392336	7.09E-19	6.29E-20	1.018325856	3.81E-14	4.44E-15	6.8187	14.044	13.8117
ENSMUSG00000038248	-1.147244975	0.00000351	0.000000844	-1.056106545	0.0000539	0.0000148	2.2983	1.0377	1.1053
ENSMUSG00000038267	1.098553914	4.7E-31	2.42E-32	1.594909293	5.65E-39	2.36E-40	4.802	10.283	14.5057
ENSMUSG00000038290	-1.284369895	3.46E-66	7.69E-68	-1.438953927	3.11E-49	1E-50	23.924	9.822	8.824
ENSMUSG00000038295	1.194016435	0.000159439	0.0000476	1.285469762	0.000033	0.00000877	0.5477	1.253	1.335
ENSMUSG00000038312	-1.196270669	1.4E-41	5.34E-43	-1.056039383	3.51E-26	2.21E-27	47.1843	20.5913	22.6933
ENSMUSG00000038349	-7.257387843	0.0000235	0.00000623	-7.257387843	0.0000175	0.00000448	0.153	0	0
ENSMUSG00000038352	-2.086888024	0.032046963	0.014426142	-2.889366226	0.010013059	0.003982687	0.6273	0.1477	0.0847
ENSMUSG00000038417	-1.22897985	0.000551433	0.000177899	-1.513360314	0.00000257	0.000000594	3.1707	1.3527	1.1107
ENSMUSG00000038473	1.800125881	0.000788652	0.000261058	2.311797264	0.000000205	4.16E-08	0.4097	1.4267	2.034
ENSMUSG00000038517	2.473408924	9.46E-78	1.75E-79	2.584356765	9.06E-81	1.76E-82	3.5733	19.8447	21.431
ENSMUSG00000038543	1.217610877	0.001222358	0.00041736	1.026845532	0.002428932	0.000860456	3.9577	9.204	8.064
ENSMUSG00000038563	1.766924975	5.93E-89	9.28E-91	1.805768183	2.6E-96	4.26E-98	8.664	29.486	30.2907
ENSMUSG00000038593	-1.291766124	0.0000125	0.00000322	-1.545419294	4.17E-08	7.93E-09	1.7513	0.7153	0.6
ENSMUSG00000038604	-3.258866641	7.61E-29	4.3E-30	-3.293533845	3.8E-221	2.13E-223	90.3083	9.4343	9.2103
ENSMUSG00000038615	-1.328219946	1.63E-50	4.81E-52	-1.117487359	2.38E-19	2.06E-20	60.13	23.9473	27.7137
ENSMUSG00000038712	1.727904359	1.05E-56	2.77E-58	2.059951082	7.96E-132	9.01E-134	15.221	50.419	63.4673
ENSMUSG00000038732	-1.30816079	9.92E-29	5.64E-30	-1.501255312	2.16E-49	6.93E-51	29.1497	11.7717	10.297
ENSMUSG00000038742	-2.621639912	9.96E-10	1.67E-10	-1.916544834	0.00000017	3.42E-08	3.1737	0.5157	0.8407
ENSMUSG00000038763	-1.134252795	6.07E-10	9.95E-11	-1.237045347	1.14E-13	1.37E-14	5.5857	2.5447	2.3697
ENSMUSG00000038770	1.396323881	0.002483261	0.000895446	1.183841967	0.008862165	0.003494938	0.4623	1.217	1.0503
ENSMUSG00000038777	-1.817866853	0.004254019	0.001599062	-1.677243988	0.000102745	0.0000293	1.9743	0.56	0.6173
ENSMUSG00000038797	-1.048646325	0.011556759	0.004730224	-1.295833634	0.00102729	0.000339096	4.2827	2.0703	1.7443
ENSMUSG00000038843	1.196435377	1.3E-35	5.91E-37	1.096318472	1.14E-22	8.38E-24	19.4737	44.6283	41.6363
ENSMUSG00000038893	-1.05350424	0.003686686	0.001369359	-1.476754857	1.83E-34	8.85E-36	27.7877	13.388	9.984
ENSMUSG00000038910	-1.519766516	1.79E-43	6.4E-45	-1.539904789	8.62E-57	2.34E-58	26.478	9.234	9.106
ENSMUSG00000038936	-1.050226667	1.36E-10	2.11E-11	-1.142445967	2.81E-17	2.73E-18	21.3257	10.298	9.6603
ENSMUSG00000038954	-1.474055163	1.47E-15	1.58E-16	-1.069590448	1.04E-08	1.86E-09	15.9477	5.7407	7.5983
ENSMUSG00000038963	5.743839345	0	0	5.833686519	0	0	2.192	117.465	125.013
ENSMUSG00000039005	2.378053455	9.94E-50	2.98E-51	2.518880468	3.48E-50	1.09E-51	1.5747	8.1857	9.025
ENSMUSG00000039013	1.900383055	4.18E-11	6.24E-12	1.464717735	2.54E-14	2.93E-15	2.1133	7.8893	5.833
ENSMUSG00000039021	-1.140832285	0.0000131	0.00000339	-1.075180876	0.0000324	0.00000861	4.4837	2.0333	2.128
ENSMUSG00000039031	2.05941821	6.85E-120	7.47E-122	2.002422223	7.79E-80	1.54E-81	6.8933	28.7327	27.6197
ENSMUSG00000039063	1.869844896	0.000000148	3.05E-08	2.199685982	2.49E-12	3.29E-13	0.7197	2.6303	3.306
ENSMUSG00000039081	-1.568693474	2.51E-17	2.42E-18	-1.312217136	1.14E-12	1.47E-13	4.6147	1.5557	1.8583
ENSMUSG00000039103	4.112671362	3.46E-51	1.01E-52	4.289498537	3.25E-53	9.59E-55	0.6763	11.7003	13.226
ENSMUSG00000039109	5.417622589	0	0	5.498300955	0	0	6.2873	268.7407	284.1973
ENSMUSG00000039131	4.111202562	0.010264253	0.004160606	4.044394119	0.005855804	0.002222553	0.026	0.4493	0.429
ENSMUSG00000039153	-1.818018309	9.72E-23	7.01E-24	-1.963419643	6.6E-20	5.6E-21	6.1787	1.7523	1.5843
ENSMUSG00000039156	-1.292312234	1.02E-45	3.44E-47	-1.206194162	3.96E-32	2.05E-33	30.9783	12.6483	13.4263
ENSMUSG00000039158	-1.302435851	1.99E-17	1.9E-18	-1.194424516	1.93E-13	2.36E-14	6.5673	2.6627	2.8697
ENSMUSG00000039166	-1.713570509	0.001429896	0.000494181	-1.156752068	0.045018924	0.020837259	4.4517	1.3573	1.9967
ENSMUSG00000039178	-1.596036018	8.28E-28	4.78E-29	-1.718570681	2.87E-28	1.67E-29	16.348	5.4077	4.9673
ENSMUSG00000039191	1.586328412	5.1E-139	4.67E-141	1.812705244	3.72E-119	4.78E-121	24.6333	73.97	86.537
ENSMUSG00000039199	-1.141895566	0.005919229	0.002280106	-1.189579861	0.001277213	0.000428824	2.1493	0.974	0.9423
ENSMUSG00000039232	1.37033028	5.12E-42	1.93E-43	1.369764106	1.4E-34	6.74E-36	5.5863	14.4423	14.4367
ENSMUSG00000039234	-1.595298623	1.05E-59	2.6E-61	-1.504522768	5.68E-46	1.97E-47	39.222	12.9807	13.8237
ENSMUSG00000039242	-1.944141034	8.26E-131	7.92E-133	-2.103944125	2.99E-119	3.79E-121	74.354	19.3223	17.2963
ENSMUSG00000039286	-3.345465799	4.26E-86	7.08E-88	-3.592638331	5.88E-153	5.52E-155	10.1137	0.995	0.8383
ENSMUSG00000039308	-2.440571397	1.12E-151	9.31E-154	-2.336207286	9.07E-126	1.09E-127	57.503	10.5927	11.3873
ENSMUSG00000039337	-4.161681007	0.000101801	0.0000296	-6.174064732	0.0000375	0.00001	0.698	0.039	0.0097
ENSMUSG00000039354	-1.066242844	2.8E-25	1.8E-26	-1.156581214	3.4E-21	2.7E-22	26.1153	12.4717	11.7147
ENSMUSG00000039384	3.061320745	1.75E-63	4.09E-65	3.31186469	3.74E-91	6.44E-93	1.7263	14.4103	17.1433
ENSMUSG00000039431	2.666415356	4.35E-22	3.23E-23	2.485039884	3.38E-21	2.68E-22	0.7547	4.791	4.225
ENSMUSG00000039474	-2.253668988	1.16E-15	1.24E-16	-1.61822869	4.88E-11	7.21E-12	3.378	0.7083	1.1003
ENSMUSG00000039477	-1.471567164	4.03E-53	1.12E-54	-1.511463502	3.01E-37	1.32E-38	24.165	8.7137	8.476
ENSMUSG00000039577	-2.233665851	3.06E-11	4.5E-12	-1.442684945	0.000122356	0.0000352	1.6273	0.346	0.5987
ENSMUSG00000039616	-1.219132605	8.31E-08	1.67E-08	-1.350820796	1.52E-11	2.15E-12	5.6487	2.4263	2.2147
ENSMUSG00000039621	1.4522694	2.11E-45	7.17E-47	1.554877581	3.77E-33	1.89E-34	6.0947	16.6773	17.9067
ENSMUSG00000039623	-1.329326653	1.48E-12	1.97E-13	-1.236158977	4.43E-13	5.55E-14	11.6647	4.642	4.9517
ENSMUSG00000039652	-1.422441535	4.03E-09	7.14E-10	-1.531238925	1.22E-08	2.21E-09	2.214	0.826	0.766
ENSMUSG00000039699	2.382857094	0.00004	0.000011	1.820719913	0.001583627	0.000541762	0.527	2.7487	1.8617
ENSMUSG00000039716	-3.646162657	0.01505419	0.006312406	-2.750860036	0.033365894	0.014932182	0.2087	0.0167	0.031
ENSMUSG00000039735	-5.254813899	1.19E-15	1.27E-16	-5.584962501	5.15E-15	5.7E-16	1.12	0.0293	0.0233
ENSMUSG00000039740	-1.187576652	3.73E-36	1.67E-37	-1.310362132	1.74E-39	7.18E-41	29.7133	13.0453	11.981
ENSMUSG00000039765	-3.309063695	0.000000123	2.51E-08	-3.695507423	9.23E-16	9.72E-17	1.2653	0.1277	0.0977
ENSMUSG00000039770	-1.972960448	1.87E-43	6.72E-45	-2.060940662	2.57E-45	9.06E-47	25.5473	6.5077	6.1227
ENSMUSG00000039783	-5.897774009	3.42E-17	3.33E-18	-5.410725265	1.73E-18	1.56E-19	2.524	0.0423	0.0593
ENSMUSG00000039824	-2.567857816	1.08E-21	8.16E-23	-2.601755575	1.52E-27	9.13E-29	15.0643	2.5407	2.4817
ENSMUSG00000039835	-6.815916936	0.001296075	0.00044415	-6.815916936	0.000983819	0.000323598	0.1127	0	0
ENSMUSG00000039851	-5.1447534	3.58E-08	6.93E-09	-4.919067153	2.57E-52	7.8E-54	8.8443	0.25	0.2923
ENSMUSG00000039953	1.403073883	2.09E-25	1.32E-26	1.599141436	6.24E-31	3.34E-32	3.33	8.8067	10.0887
ENSMUSG00000039959	1.659988351	1.32E-27	7.71E-29	1.409656162	4E-26	2.52E-27	1.813	5.7293	4.8167
ENSMUSG00000039968	2.176559859	3.66E-140	3.33E-142	2.112080511	3.11E-125	3.75E-127	11.6667	52.742	50.4367
ENSMUSG00000039982	-2.156380021	1.32E-20	1.05E-21	-2.591449525	6.06E-57	1.64E-58	8.693	1.95	1.4423
ENSMUSG00000039987	2.032363292	2.46E-79	4.44E-81	2.081834239	1.01E-67	2.32E-69	3.4897	14.2753	14.7733
ENSMUSG00000040013	-2.43148802	0.000834263	0.000277431	-3.18047878	0.0000314	0.00000832	1.372	0.2543	0.1513
ENSMUSG00000040022	-2.071408165	6.33E-34	2.99E-35	-1.845342453	5.18E-24	3.63E-25	6.516	1.5503	1.8133
ENSMUSG00000040061	2.122041282	5.04E-99	6.66E-101	2.249174322	4.47E-248	2.19E-250	16.1317	70.2227	76.6917
ENSMUSG00000040065	-6.176588732	0.039081583	0.017937619	-6.176588732	0.032487192	0.014501335	0.0723	0	0
ENSMUSG00000040118	-10.37829486	1.04E-14	1.19E-15	-8.963257357	4.13E-12	5.57E-13	1.331	0	0.0027
ENSMUSG00000040165	-4.083449821	8.26E-21	6.55E-22	-4.162135467	1.65E-101	2.61E-103	31.8317	1.8777	1.778
ENSMUSG00000040187	-4.045431124	1.15E-12	1.51E-13	-4.404512217	2.64E-11	3.8E-12	1.6237	0.0983	0.0767
ENSMUSG00000040209	-1.101219291	4.16E-20	3.42E-21	-1.298832251	1.36E-21	1.05E-22	8.7373	4.0727	3.5513
ENSMUSG00000040229	-5.298599427	4.03E-61	9.86E-63	-5.83822262	1.09E-58	2.87E-60	12.739	0.3237	0.2227
ENSMUSG00000040231	-2.517340197	0.012763942	0.005262521	-2.442970372	0.013061387	0.005314192	1.1183	0.1953	0.2057
ENSMUSG00000040253	2.218661082	9.98E-12	1.41E-12	2.372767096	1.85E-15	2E-16	0.4517	2.1023	2.3393
ENSMUSG00000040264	2.909383668	1.27E-11	1.81E-12	3.021530011	2.31E-14	2.65E-15	0.2827	2.1237	2.2953
ENSMUSG00000040272	1.267789728	7.62E-08	1.52E-08	1.056296614	1.89E-08	3.48E-09	2.869	6.9083	5.9663
ENSMUSG00000040274	-1.524907021	4.56E-184	2.69E-186	-1.553604519	1.78E-84	3.3E-86	92.7323	32.2247	31.59
ENSMUSG00000040283	2.681495902	3.05E-204	1.61E-206	2.519782722	9.8E-127	1.14E-128	5.0633	32.4823	29.038
ENSMUSG00000040296	1.100856869	3.32E-17	3.23E-18	1.061427822	2.64E-15	2.87E-16	7.713	16.543	16.097
ENSMUSG00000040297	-1.350589654	7.06E-24	4.85E-25	-1.348642361	9.12E-46	3.18E-47	27.0623	10.612	10.6263
ENSMUSG00000040314	-7.800557376	0	0	-9.281897246	5.28E-18	4.9E-19	293.3983	1.316	0.4713
ENSMUSG00000040328	10.88874325	8.44E-11	1.29E-11	10.68708402	2.67E-10	4.23E-11	0	1.896	1.6487
ENSMUSG00000040350	1.770834212	0.001669788	0.000585093	2.484973458	0.023566306	0.010196772	0.341	1.1637	1.909
ENSMUSG00000040359	-1.239428568	6.85E-29	3.84E-30	-1.138476548	1.15E-23	8.23E-25	23.2367	9.8417	10.555
ENSMUSG00000040433	1.561448496	2.71E-96	3.69E-98	1.570000625	5.65E-89	9.99E-91	13.7527	40.591	40.8323
ENSMUSG00000040447	3.107933436	0	0	3.115471757	6E-169	5.05E-171	16.9373	146.0247	146.7897
ENSMUSG00000040479	2.613696017	6.07E-250	2.32E-252	2.686336051	5.98E-292	1.98E-294	16.5787	101.473	106.713
ENSMUSG00000040483	2.381520373	0.009232224	0.003711489	2.09893317	0.001676244	0.000575413	0.3193	1.664	1.368
ENSMUSG00000040488	-2.369381471	7.73E-65	1.77E-66	-2.094627634	2.34E-34	1.14E-35	14.1133	2.7313	3.3043
ENSMUSG00000040498	2.74130453	0.021148261	0.009152738	2.427476934	0.043551541	0.020083784	0.0553	0.37	0.2977
ENSMUSG00000040502	-1.48116871	1.92E-15	2.08E-16	-1.325028883	2.85E-11	4.12E-12	6.676	2.3913	2.6647
ENSMUSG00000040533	2.578912353	0.00000264	0.000000626	1.920684395	0.002450452	0.000868587	0.3053	1.8243	1.156
ENSMUSG00000040562	6.733109287	5.97E-91	8.88E-93	6.572677929	8.67E-88	1.55E-89	0.439	46.7017	41.7867
ENSMUSG00000040613	-1.543097375	0.000152431	0.0000454	-1.537758291	0.000000137	2.73E-08	3.668	1.2587	1.2633
ENSMUSG00000040616	-1.084531487	0.0000032	0.000000766	-1.352952439	2.03E-10	3.17E-11	8.1943	3.864	3.208
ENSMUSG00000040627	-8.607330314	0.000654234	0.000213472	-8.607330314	0.000493539	0.000154768	0.39	0	0
ENSMUSG00000040732	-4.901880564	0.0000156	0.00000403	-6.223808659	2.56E-18	2.33E-19	9.9653	0.3333	0.1333
ENSMUSG00000040747	-2.776526203	0.035592796	0.016207765	-4.975349803	0	0	188.3593	27.4897	5.9877
ENSMUSG00000040759	9.573015949	0.000351937	0.000110336	9.337621902	0.000301113	0.0000913	0	0.7617	0.647
ENSMUSG00000040822	-1.358522798	5.72E-15	6.41E-16	-1.118527038	1.06E-11	1.47E-12	28.3073	11.0393	13.0373
ENSMUSG00000040852	-5.736643528	6.8E-09	1.23E-09	-8.959035949	3.74E-10	6E-11	0.4977	0.0093	0
ENSMUSG00000040867	-2.536599479	0.0000541	0.0000151	-2.209396442	0.000684713	0.00021987	1.056	0.182	0.2283
ENSMUSG00000040913	-1.426715802	7.09E-19	6.29E-20	-1.442337833	4.44E-11	6.52E-12	13.396	4.983	4.9293
ENSMUSG00000040957	-1.629029841	0.005015617	0.001908339	-2.177264309	0.0000118	0.00000295	0.9197	0.2973	0.2033
ENSMUSG00000040969	-3.962267353	0.000118426	0.0000348	-1.834777618	0.019885013	0.008444678	0.239	0.0153	0.067
ENSMUSG00000040998	-2.351850637	0.00000024	5.06E-08	-2.644679046	2.65E-09	4.52E-10	1.3153	0.2577	0.2103
ENSMUSG00000041012	1.988559848	0.004369142	0.001644461	1.496425826	0.020039322	0.008522661	0.4853	1.926	1.3693
ENSMUSG00000041040	-2.613694524	3.95E-88	6.32E-90	-2.624243052	6.77E-167	5.94E-169	27.1657	4.4383	4.406

ENSMUSG00000041052	-7.622597554	3.02E-32	1.51E-33	-9.253074714	2.37E-17	2.29E-18	10.5763	0.0537	0.0173
ENSMUSG00000041058	-2.607051732	0	0	-2.689496323	4.25E-204	2.76E-206	68.6653	11.2703	10.6443
ENSMUSG00000041096	-1.450662936	4.62E-22	3.43E-23	-1.487439258	3.87E-23	2.81E-24	20.9957	7.6813	7.488
ENSMUSG00000041135	-1.177377049	1.37E-11	1.96E-12	-1.05054838	0.000000245	0.00000005	7.958	3.5187	3.842
ENSMUSG00000041141	-2.246115613	0.0000111	0.00000284	-2.333578455	0.000000169	3.41E-08	0.9947	0.2097	0.1973
ENSMUSG00000041143	-1.845897219	0.000000213	4.46E-08	-1.670577456	4.26E-10	6.87E-11	5.7217	1.5917	1.7973
ENSMUSG00000041168	-1.212088972	2.96E-74	5.8E-76	-1.192692485	1.02E-24	6.91E-26	108.687	46.914	47.549
ENSMUSG00000041187	1.420541517	8.01E-53	2.24E-54	1.445933744	4.03E-33	2.03E-34	9.222	24.686	25.1243
ENSMUSG00000041189	-1.987791691	1.21E-17	1.14E-18	-2.193368682	1.18E-23	8.44E-25	7.2173	1.8197	1.578
ENSMUSG00000041220	1.901070734	3.21E-93	4.64E-95	1.918139999	8.22E-113	1.14E-114	7.8437	29.2953	29.644
ENSMUSG00000041235	-3.729910837	0.0000358	0.00000976	-2.160672688	0.006545761	0.002515445	0.544	0.041	0.1217
ENSMUSG00000041329	-3.280508168	5.51E-11	8.32E-12	-3.639194812	3.49E-12	4.68E-13	1.545	0.159	0.124
ENSMUSG00000041372	-1.361604097	0.033405723	0.015107416	-1.414715433	0.030772801	0.013652722	0.4977	0.1937	0.1867
ENSMUSG00000041396	1.49512232	5.85E-19	5.15E-20	1.633587518	1.34E-20	1.09E-21	4.901	13.8153	15.207
ENSMUSG00000041406	1.545066633	7.73E-91	1.15E-92	1.686077425	4.26E-67	9.9E-69	16.6983	48.7287	53.732
ENSMUSG00000041481	-10.02928723	0.00000359	0.000000864	-5.292321633	0.0000604	0.0000167	1.045	0	0.0267
ENSMUSG00000041491	-1.070226024	4.64E-13	5.93E-14	-1.14169421	8.49E-21	6.85E-22	20.318	9.6763	9.2087
ENSMUSG00000041538	-1.356972438	0.002284084	0.0008197	-1.619681437	2.93E-08	5.5E-09	3.785	1.4777	1.2317
ENSMUSG00000041607	1.209629896	7.71E-29	4.36E-30	1.322114712	3.84E-39	1.6E-40	9.7917	22.646	24.4823
ENSMUSG00000041608	-3.48188014	1.7E-10	2.66E-11	-5.230918566	6.13E-15	6.81E-16	1.4897	0.1333	0.0397
ENSMUSG00000041679	-1.609526349	0.000167452	0.0000501	-1.833417986	0.00000575	0.00000139	2.4483	0.8023	0.687
ENSMUSG00000041762	4.396237792	1.64E-19	1.39E-20	4.215329932	8.42E-18	7.89E-19	0.0817	1.7197	1.517
ENSMUSG00000041794	-8.367778746	0.001699563	0.000596116	-4.667339028	0.006576083	0.002528006	0.3303	0	0.013
ENSMUSG00000041836	1.65318574	5.41E-94	7.7E-96	1.609225917	1.04E-40	4.15E-42	13.299	41.829	40.5737
ENSMUSG00000041857	-8.427662038	0.019907948	0.008573072	-8.427662038	0.016205088	0.006748763	0.3443	0	0
ENSMUSG00000041890	-1.050621783	6E-30	3.21E-31	-1.211057181	1.7E-22	1.26E-23	40.033	19.3263	17.2923
ENSMUSG00000041895	1.22953566	2.52E-21	1.94E-22	1.447399137	3.43E-28	2E-29	6.965	16.3323	18.9947
ENSMUSG00000041911	1.295116914	4.04E-14	4.77E-15	1.312210535	4.19E-16	4.34E-17	3.7837	9.285	9.3957
ENSMUSG00000041920	1.818301701	2.69E-99	3.53E-101	1.964973338	2.42E-109	3.53E-111	13.942	49.1687	54.4303
ENSMUSG00000041959	1.442924755	1.31E-78	2.4E-80	1.48564957	2.14E-50	6.67E-52	94.6797	257.407	265.144
ENSMUSG00000041991	-3.015980785	0.0000188	0.00000493	-3.074279426	0.000000444	9.33E-08	0.2723	0.0337	0.0323
ENSMUSG00000042042	-2.385144076	7.9E-293	2.42E-295	-2.406414534	1.17E-182	9.08E-185	182.3963	34.9153	34.4043
ENSMUSG00000042082	-4.714255405	1.56E-15	1.67E-16	-5.589993942	0	0	36.479	1.3897	0.7573
ENSMUSG00000042106	5.957815713	4.78E-193	2.69E-195	5.966736943	1.02E-188	7.39E-191	1.4897	92.5913	93.1657
ENSMUSG00000042148	-1.246045437	4.42E-29	2.46E-30	-1.053766126	1.84E-25	1.19E-26	27.2903	11.5057	13.146
ENSMUSG00000042155	1.472740495	4.27E-36	1.92E-37	1.330525321	8E-28	4.76E-29	3.9597	10.99	9.9583
ENSMUSG00000042182	-7.809500194	0.001873772	0.000662296	-7.809500194	0.001435888	0.000487473	0.2243	0	0
ENSMUSG00000042203	-1.730310557	8.05E-62	1.93E-63	-1.790905576	1.46E-53	4.28E-55	26.002	7.8367	7.5143
ENSMUSG00000042207	-1.020006035	6.35E-13	8.21E-14	-1.241513453	3.17E-52	9.66E-54	30.1107	14.848	12.7347
ENSMUSG00000042228	2.317939049	3.66E-266	1.27E-268	2.21875315	3.19E-189	2.29E-191	17.6253	87.8833	82.0443
ENSMUSG00000042249	-1.085103218	2.05E-10	3.23E-11	-1.13481865	6.61E-14	7.85E-15	4.76	2.2437	2.1677
ENSMUSG00000042275	-1.571148044	2.51E-25	1.61E-26	-1.174087669	4.32E-12	5.83E-13	25.3947	8.5463	11.254
ENSMUSG00000042286	-4.295292968	0.0000032	0.000000764	-5.044945661	4.04E-38	1.73E-39	3.4883	0.1777	0.1057
ENSMUSG00000042320	-1.788832083	0.0000179	0.00000468	-1.638320367	0.0000345	0.00000919	1.5607	0.4517	0.5013
ENSMUSG00000042333	-3.182746789	3.55E-08	6.86E-09	-3.427842319	1.55E-12	2.03E-13	9.5313	1.0497	0.8857
ENSMUSG00000042345	5.220349346	7.94E-153	6.4E-155	5.353771471	5.27E-172	4.33E-174	0.612	22.8157	25.0263
ENSMUSG00000042349	-1.25128291	7.79E-14	9.38E-15	-1.455807693	2.15E-18	1.95E-19	8.681	3.6467	3.1647
ENSMUSG00000042350	-1.35338337	4.79E-60	1.18E-61	-1.465178778	2.86E-74	6.04E-76	40.6637	15.9147	14.728
ENSMUSG00000042351	13.24014535	3.9E-20	3.2E-21	13.4552844	1.74E-16	1.76E-17	0	9.6757	11.2317
ENSMUSG00000042354	1.009273702	6.69E-28	3.85E-29	1.121696501	2.44E-43	9.12E-45	28.8687	58.1097	62.819
ENSMUSG00000042524	-1.415720764	1.52E-85	2.54E-87	-1.437790231	3.23E-37	1.42E-38	80.4897	30.1693	29.7113
ENSMUSG00000042532	-7.564784619	0.00172233	0.00060458	-3.657894023	0.023979156	0.010391961	0.1893	0	0.015
ENSMUSG00000042581	-6.979822118	1.34E-13	1.64E-14	-6.308444866	5.68E-11	8.44E-12	1.136	0.009	0.0143
ENSMUSG00000042590	1.659176065	5.84E-48	1.87E-49	1.333838606	9.81E-18	9.22E-19	14.0733	44.4487	35.475
ENSMUSG00000042594	2.742023606	2.84E-205	1.46E-207	3.031055727	3.7E-213	2.22E-215	9.0497	60.543	73.9727
ENSMUSG00000042616	-2.281520249	0.0000102	0.00000259	-2.331843564	0.0000109	0.00000271	1.6547	0.3403	0.3287
ENSMUSG00000042622	1.90509835	0.000000224	4.71E-08	2.19244981	1.29E-09	2.16E-10	0.7697	2.8827	3.518
ENSMUSG00000042647	-1.325993349	5.1E-11	7.66E-12	-1.138328157	3.57E-09	6.15E-10	3.85	1.5357	1.749
ENSMUSG00000042675	-1.475860389	3.69E-09	6.52E-10	-1.495944317	4.16E-11	6.1E-12	19.6503	7.0647	6.967
ENSMUSG00000042684	-1.019497892	7.71E-12	1.08E-12	-1.096635783	4.83E-11	7.13E-12	17.1583	8.464	8.0233
ENSMUSG00000042688	1.185057954	2.48E-37	1.08E-38	1.368778097	4.33E-50	1.37E-51	13.7547	31.2743	35.5217
ENSMUSG00000042700	-3.598712831	0	0	-3.992241753	0	0	119.3723	9.8533	7.501
ENSMUSG00000042745	2.974194369	3.11E-87	5.06E-89	2.984937012	5.11E-91	8.82E-93	6.226	48.925	49.2907
ENSMUSG00000042751	1.244590086	0.005298695	0.002023774	1.968877483	0.00000108	0.00000024	0.2733	0.6477	1.07
ENSMUSG00000042807	6.273018494	0.0000547	0.0000153	5.672425342	0.001364796	0.000460963	0	0.0773	0.051
ENSMUSG00000042842	2.636116589	8.68E-18	8.16E-19	2.588895338	5.59E-19	4.95E-20	0.7327	4.5547	4.408
ENSMUSG00000043017	2.819787365	2.56E-152	2.08E-154	2.776562917	3.76E-138	4.13E-140	5.315	37.527	36.4193
ENSMUSG00000043019	-3.015716535	0	0	-3.121697587	2.43E-278	9.05E-281	66.9143	8.2737	7.6877
ENSMUSG00000043157	1.790935239	0.00000036	7.76E-08	1.534645853	0.0000103	0.00000257	1.073	3.713	3.1087
ENSMUSG00000043251	1.007655544	5.51E-13	7.09E-14	1.174001453	1.07E-16	1.06E-17	6.453	12.9747	14.5603
ENSMUSG00000043257	-1.470212029	5.23E-13	6.71E-14	-1.475116571	2.08E-13	2.55E-14	7.8917	2.8483	2.8387
ENSMUSG00000043510	-1.392332086	0.0000792	0.0000227	-1.199966105	0.000000423	8.88E-08	16.3977	6.2467	7.1377
ENSMUSG00000043639	-8.282316239	0.00000113	0.000000256	-4.312689888	0.0000877	0.0000247	0.3113	0	0.0157
ENSMUSG00000043733	1.036161326	6.13E-19	5.4E-20	1.178642219	4.43E-55	1.26E-56	17.8933	36.695	40.504
ENSMUSG00000043811	2.078514881	4.18E-13	5.32E-14	1.562693951	6.03E-08	1.16E-08	1.1607	4.9023	3.4287
ENSMUSG00000043881	2.049913514	0.0000659	0.0000187	1.871335548	0.000180435	0.0000532	0.1397	0.5783	0.511
ENSMUSG00000043969	-7.251087854	0.027625063	0.012236013	-7.251087854	0.022655662	0.00976521	0.1523	0	0
ENSMUSG00000043987	-1.225659061	6.79E-23	4.89E-24	-1.68572012	2.66E-27	1.61E-28	11.8397	5.0627	3.6803
ENSMUSG00000044017	1.6398814	0.03158015	0.014196259	1.612683258	0.033316764	0.014903294	0.063	0.1963	0.1927
ENSMUSG00000044022	-2.924073607	0.0000299	0.00000806	-2.796881591	0.0000246	0.00000644	0.549	0.0723	0.079
ENSMUSG00000044072	-2.137503524	0.000248178	0.000076	-1.609571968	0.003883975	0.00143115	0.3153	0.0717	0.1033
ENSMUSG00000044080	1.309401352	2.88E-34	1.36E-35	1.306402694	5.01E-28	2.94E-29	33.0353	81.8743	81.7043
ENSMUSG00000044165	-6.670266157	0.0000205	0.00000539	-7.694928211	0.00000765	0.00000188	2.003	0.0197	0.0097
ENSMUSG00000044197	3.681112739	1.84E-65	4.14E-67	3.808712908	5.13E-68	1.16E-69	0.8343	10.702	11.6917
ENSMUSG00000044199	-1.065978762	0.000266143	0.0000819	-1.3838665	0.00000017	3.42E-08	4.3547	2.08	1.6687
ENSMUSG00000044229	2.012410356	1.15E-08	2.13E-09	1.963980048	1.23E-08	2.22E-09	0.463	1.868	1.8063
ENSMUSG00000044258	6.326946071	1.06E-90	1.59E-92	6.939084018	2.13E-109	3.09E-111	0.4233	33.9847	51.9463
ENSMUSG00000044303	8.317412614	0.033188624	0.015002318	9.30833903	0.002362618	0.000835986	0	0.319	0.634
ENSMUSG00000044328	-1.920269353	2.32E-26	1.42E-27	-1.92958425	1.83E-27	1.1E-28	16.467	4.3507	4.3227
ENSMUSG00000044350	1.091987938	0.000000742	0.000000166	1.278589457	8.55E-12	1.18E-12	2.7213	5.801	6.602
ENSMUSG00000044367	2.124650922	1.88E-16	1.91E-17	2.31911033	1.35E-19	1.16E-20	0.82	3.576	4.092
ENSMUSG00000044447	1.576342264	2.17E-38	9.07E-40	1.697939689	3E-55	8.4E-57	4.4577	13.2933	14.4623
ENSMUSG00000044456	-1.289876852	4.12E-52	1.16E-53	-1.217301054	1.41E-19	1.22E-20	38.972	15.939	16.7613
ENSMUSG00000044469	1.603383116	4.42E-13	5.64E-14	1.381438266	3.35E-08	6.32E-09	1.591	4.8343	4.145
ENSMUSG00000044551	2.226559817	6.07E-22	4.55E-23	2.184727733	3.26E-23	2.36E-24	1.5227	7.1263	6.9227
ENSMUSG00000044566	-2.041552489	1.65E-08	3.09E-09	-1.594093512	0.00000819	0.00000202	1.7963	0.4363	0.595
ENSMUSG00000044712	1.236560179	7.02E-99	9.32E-101	1.048252534	1.79E-37	7.77E-39	1204.3007	2837.7663	2490.522
ENSMUSG00000044734	-4.398696146	0.000000189	3.94E-08	-7.382369176	3.27E-31	1.74E-32	108.7277	5.1547	0.6517
ENSMUSG00000044786	2.862127174	8.83E-28	5.11E-29	3.372406701	7.31E-63	1.81E-64	1.7093	12.4283	17.702
ENSMUSG00000044912	11.70116204	1.28E-15	1.37E-16	12.2540444	2.38E-14	2.74E-15	0	3.3297	4.8847
ENSMUSG00000044952	-1.394335144	0.001885544	0.000667243	-1.499421786	0.00167715	0.00057584	1.2223	0.465	0.4323
ENSMUSG00000044968	-2.389356952	1.91E-13	2.37E-14	-2.176742583	9.74E-13	1.25E-13	2.4747	0.4723	0.5473
ENSMUSG00000045004	-3.726831217	1.54E-11	2.2E-12	-4.353879119	3.68E-13	4.58E-14	1.7653	0.1333	0.0863
ENSMUSG00000045038	1.110498537	3.82E-18	3.52E-19	1.33643333	3.88E-26	2.44E-27	4.4137	9.53	11.1457
ENSMUSG00000045092	2.8668256	5.35E-43	1.96E-44	2.438382222	2.98E-31	1.58E-32	1.0523	7.6763	5.704
ENSMUSG00000045102	-1.812490686	0.001763547	0.000620028	-2.317111079	0.0000785	0.000022	0.7973	0.227	0.16
ENSMUSG00000045140	1.0497493	0.00000012	2.45E-08	1.002202252	0.00000228	0.000000523	3.273	6.7757	6.556
ENSMUSG00000045216	1.478603474	1.21E-34	5.64E-36	1.759785436	3.7E-32	1.91E-33	8.5783	23.906	29.0503
ENSMUSG00000045257	-1.907782779	0.001076863	0.000364615	-3.016838304	0.00000307	0.000000718	3.5047	0.934	0.433
ENSMUSG00000045282	-1.342283055	0.0000919	0.0000266	-1.247875422	0.0000403	0.0000109	6.9237	2.7307	2.9153
ENSMUSG00000045312	-2.102604485	0.029835423	0.013312464	-2.445059982	0.001327066	0.000446753	0.5827	0.1357	0.107
ENSMUSG00000045382	-4.839250056	2.92E-11	4.28E-12	-6.098430496	0	0	443.0433	15.477	6.466
ENSMUSG00000045409	-1.418880476	2.47E-25	1.58E-26	-1.500783366	3.77E-22	2.85E-23	15.9553	5.9673	5.638
ENSMUSG00000045414	-1.822375624	1.69E-48	5.33E-50	-1.945852958	1.84E-52	5.55E-54	27.838	7.8713	7.2257
ENSMUSG00000045466	-1.141923006	0.000000235	4.96E-08	-1.005718623	0.00000105	0.000000231	6.9093	3.131	3.441
ENSMUSG00000045502	-2.502500341	0.00610807	0.002363032	-10.09495738	2.92E-08	5.48E-09	1.0937	0.193	0
ENSMUSG00000045659	2.966736429	4.51E-66	1.01E-67	2.874489014	6.47E-33	3.27E-34	1.0987	8.589	8.057
ENSMUSG00000045671	2.099030144	8.39E-90	1.29E-91	2.27636744	1.55E-71	3.39E-73	6.1527	26.3593	29.807
ENSMUSG00000045679	-1.371048447	7.13E-20	5.96E-21	-1.546923543	9.88E-26	6.33E-27	31.0037	11.9863	10.6107
ENSMUSG00000045780	-1.885466359	5.1E-29	2.85E-30	-2.1191416	1.62E-39	6.65E-41	6.7453	1.8257	1.5527
ENSMUSG00000045795	-1.259394455	1.32E-20	1.06E-21	-1.183193725	2.2E-17	2.12E-18	16.7577	7	7.3797
ENSMUSG00000045827	2.826111785	1.3E-22	9.42E-24	2.672523493	1.54E-19	1.33E-20	0.4803	3.4063	3.0623
ENSMUSG00000045838	1.952624462	1.49E-19	1.26E-20	2.026848249	3.23E-24	2.24E-25	0.9183	3.5547	3.7423
ENSMUSG00000045980	-1.244752971	3.93E-16	4.04E-17	-1.177575222	1.21E-17	1.14E-18	8.9657	3.7833	3.9637
ENSMUSG00000046006	-3.350747586	1.53E-12	2.04E-13	-3.776464863	5.83E-37	2.61E-38	72.2353	7.0807	5.2713
ENSMUSG00000046070	-1.032122503	0.000338021	0.000105529	-1.264061601	0.00000756	0.00000185	2.9973	1.4657	1.248
ENSMUSG00000046179	-1.713150107	1.07E-148	9.29E-151	-1.613217414	2.53E-63	6.24E-65	80.2127	24.4643	26.219
ENSMUSG00000046223	2.829748586	2.11E-45	7.19E-47	3.001190938	3.1E-52	9.44E-54	2.3713	16.859	18.9863
ENSMUSG00000046324	-1.076828417	3.37E-10	5.41E-11	-1.063606559	3.68E-32	1.9E-33	25.2023	11.9477	12.0577
ENSMUSG00000046546	1.164409914	0.001112311	0.000377545	1.251478716	0.000124648	0.0000359	0.6407	1.436	1.5253
ENSMUSG00000046668	1.120074063	7.49E-24	5.14E-25	1.181600279	7.57E-25	5.07E-26	11.883	25.8287	26.954
ENSMUSG00000046688	2.224148361	2.65E-22	1.95E-23	2.131956273	1.09E-20	8.87E-22	1.6227	7.5817	7.1123
ENSMUSG00000046711	1.20521128	1.79E-33	8.57E-35	1.322417933	7.67E-32	4.02E-33	69.6923	160.6903	174.29
ENSMUSG00000046727	-2.545247996	6.08E-12	8.45E-13	-3.606850246	3.2E-17	3.13E-18	7.2613	1.244	0.596
ENSMUSG00000046768	4.104248054	2.13E-11	3.09E-12	3.625245789	0.003053434	0.001101083	0.1893	3.2563	2.3363
ENSMUSG00000046805	-1.207884751	0.017587592	0.007476101	-1.508452954	0.00000402	0.000000955	1.781	0.771	0.626
ENSMUSG00000046806	2.361895302	4.17E-75	8.02E-77	2.222392421	2.02E-74	4.26E-76	3.287	16.8967	15.3393
ENSMUSG00000046879	1.491165109	1.92E-43	6.89E-45	1.517361825	5.16E-37	2.3E-38	9.3377	26.2497	26.7307
ENSMUSG00000046916	4.136404889	2.86E-122	3.08E-124	4.148884477	7.44E-130	8.52E-132	1.453	25.5533	25.7753
ENSMUSG00000046962	-1.46371635	9.59E-19	8.6E-20	-1.569097214	1.09E-16	1.09E-17	19.4737	7.0603	6.563
ENSMUSG00000047045	2.058820411	6.66E-180	4.16E-182	2.231780726	3.22E-127	3.74E-129	14.7007	61.2497	69.051
ENSMUSG00000047180	1.188598115	2.45E-13	3.07E-14	1.218241993	1.56E-17	1.49E-18	5.868	13.375	13.6527
ENSMUSG00000047227	-1.668741074	0.000667868	0.000218417	-1.208549028	0.040591124	0.018549617	2.5583	0.8047	1.107
ENSMUSG00000047250	4.550467569	0	0	4.548478882	0	0	7.1977	168.6627	168.4303
ENSMUSG00000047343	7.876516947	0.041805649	0.019312787	8.506472886	0.005154639	0.001941709	0	0.235	0.3637
ENSMUSG00000047394	-2.52902336	0.0007343	0.000241443	-2.51162631	0.000200376	0.0000595	3.3303	0.577	0.584
ENSMUSG00000047407	-2.067825337	3.29E-65	7.43E-67	-1.951124865	8.45E-25	5.67E-26	46.5107	11.0937	12.0283

ENSMUSG00000047419	2.196324145	4.75E-26	2.95E-27	2.069623668	9.26E-19	8.29E-20	0.359	1.6453	1.507
ENSMUSG00000047446	-1.916740036	1.6E-66	3.52E-68	-2.157124963	9.56E-76	1.98E-77	41.3123	10.9417	9.2623
ENSMUSG00000047462	-7.269905883	0.007697486	0.003038889	-7.269905883	0.006061936	0.002307892	0.1543	0	0
ENSMUSG00000047492	-3.698806786	1.19E-11	1.69E-12	-3.405447844	1.78E-11	2.53E-12	0.883	0.068	0.0833
ENSMUSG00000047496	-5.832890014	0.005690911	0.002187018	-4.417852515	0.018179176	0.00765875	0.057	0	0.0027
ENSMUSG00000047604	-1.360857008	1.36E-32	6.69E-34	-1.50636165	1.02E-38	4.32E-40	41.362	16.1043	14.5593
ENSMUSG00000047648	-1.182067648	1.63E-16	1.65E-17	-1.131824509	6.82E-18	6.36E-19	12.677	5.587	5.785
ENSMUSG00000047746	-1.3477681	0.000359626	0.000112872	-1.068171503	0.001486967	0.000506641	1.1267	0.4427	0.5373
ENSMUSG00000047766	1.222978114	1.56E-15	1.68E-16	1.193072169	5.18E-15	5.74E-16	3.87	9.0337	8.8483
ENSMUSG00000047798	2.258014148	1.34E-18	1.2E-19	2.180673578	6.33E-17	6.23E-18	1.5353	7.344	6.9607
ENSMUSG00000047822	-3.469832488	5.89E-20	4.89E-21	-2.725477915	2.88E-10	4.56E-11	11.508	1.0387	1.74
ENSMUSG00000047842	-4.905656996	1.5E-10	2.34E-11	-6.436171712	1.68E-08	3.08E-09	0.7793	0.026	0.009
ENSMUSG00000047880	3.181329765	0.001829704	0.000645195	3.670807971	0.0000282	0.00000742	0.0467	0.4233	0.5943
ENSMUSG00000047921	1.42062243	1.5E-64	3.45E-66	1.533624551	4.64E-55	1.32E-56	8.4543	22.6323	24.4763
ENSMUSG00000047953	1.930737338	0.002956689	0.001080182	2.579693063	0.00000119	0.000000267	0.2133	0.8133	1.2753
ENSMUSG00000047959	1.322460062	2.18E-08	4.14E-09	1.533095992	4.51E-30	2.48E-31	7.2307	18.0833	20.926
ENSMUSG00000047986	1.367252085	0.033413833	0.015113405	1.632763222	0.006065037	0.002309772	0.3133	0.8083	0.9717
ENSMUSG00000047996	1.785685796	4.65E-20	3.84E-21	1.711864519	1.14E-15	1.21E-16	2.006	6.9163	6.5713
ENSMUSG00000048126	-4.733923002	0.000012	0.00000308	-7.43706693	4.15E-32	2.16E-33	2.3683	0.089	0.0137
ENSMUSG00000048148	5.693486957	1.51E-20	1.21E-21	5.671482096	1.01E-21	7.82E-23	0.02	1.035	1.0193
ENSMUSG00000048200	1.919849481	0.000130198	0.0000385	1.613670758	0.00139004	0.000470715	0.5197	1.9663	1.5903
ENSMUSG00000048216	-7.837102265	0.001744348	0.000612673	-3.136662547	0.03062209	0.013579514	0.2287	0	0.026
ENSMUSG00000048376	3.09162655	0	0	3.305357615	0	0	25.21	214.9043	249.221
ENSMUSG00000048410	-1.325316832	2.43E-15	2.65E-16	-1.405831002	1.74E-15	1.88E-16	6.1093	2.438	2.3057
ENSMUSG00000048478	-2.072323236	0.0000778	0.0000223	-1.1331938	0.007606601	0.002954622	1.493	0.355	0.6807
ENSMUSG00000048497	1.41958843	1.41E-16	1.42E-17	1.331367358	1.64E-14	1.87E-15	13.5177	36.161	34.016
ENSMUSG00000048612	3.012576379	5.11E-21	4.01E-22	3.18811985	8.72E-29	5E-30	0.3093	2.4963	2.8193
ENSMUSG00000048652	-3.262972262	1.11E-15	1.17E-16	-4.551484945	1.23E-18	1.1E-19	4.643	0.4837	0.198
ENSMUSG00000048721	-1.974473767	0.001494504	0.000518586	-1.778112636	0.001785433	0.000616347	0.9523	0.2423	0.2777
ENSMUSG00000048827	1.320714386	6.02E-12	8.37E-13	1.463928143	2.47E-13	3.05E-14	0.9513	2.3763	2.6243
ENSMUSG00000048897	1.452610614	5.31E-38	2.24E-39	1.351750136	2.48E-36	1.13E-37	5.5443	15.175	14.1503
ENSMUSG00000048924	-3.996533148	6.43E-19	5.67E-20	-6.460403832	1.64E-18	1.48E-19	3.4637	0.217	0.0393
ENSMUSG00000048992	-7.87036472	0.026006729	0.011455969	-7.87036472	0.02131189	0.009118323	0.234	0	0
ENSMUSG00000049090	-2.410121387	3.35E-68	7.08E-70	-1.773737003	7.22E-08	0.000000014	38.606	7.2633	11.2903
ENSMUSG00000049184	-1.509691554	0.00000207	0.000000487	-1.499735027	0.000015	0.00000384	6.1677	2.166	2.181
ENSMUSG00000049225	1.955050486	9.21E-08	1.86E-08	1.792615653	1.26E-09	2.1E-10	15.8863	61.596	55.037
ENSMUSG00000049456	-7.122396631	0.006299077	0.00244174	-7.122396631	0.004913856	0.001843431	0.1393	0	0
ENSMUSG00000049493	2.767067626	0.022995868	0.010035432	2.232290882	0.020358692	0.00867395	0.0553	0.3767	0.26
ENSMUSG00000049550	-1.281450328	2.34E-27	1.38E-28	-1.381150524	6.25E-20	5.29E-21	11.6737	4.8023	4.4817
ENSMUSG00000049577	3.276751027	0	0	3.570971332	0	0	22.7263	220.257	270.084
ENSMUSG00000049709	1.928702276	2.06E-12	2.76E-13	1.619499446	4.49E-11	6.59E-12	0.7017	2.6713	2.156
ENSMUSG00000049744	4.51811302	4.45E-14	5.27E-15	4.475889589	6.23E-40	2.53E-41	0.346	7.928	7.6993
ENSMUSG00000049775	1.645811515	1.07E-89	1.65E-91	1.589472679	2.76E-48	9.07E-50	583.109	1824.687	1754.8043
ENSMUSG00000049791	-1.372557144	1.81E-22	1.32E-23	-1.894609441	1.8E-37	7.82E-39	5.7707	2.2287	1.552
ENSMUSG00000049807	-1.212546473	7.71E-14	9.28E-15	-1.023931647	8.46E-09	1.51E-09	4.1537	1.7923	2.0427
ENSMUSG00000049858	-1.040798075	3.34E-09	5.88E-10	-1.170049163	5.33E-08	1.02E-08	10.6517	5.1773	4.7337
ENSMUSG00000049916	2.09851193	1.58E-18	1.43E-19	1.798478208	2.26E-16	2.3E-17	3.54	15.1607	12.314
ENSMUSG00000050014	2.059285004	1.23E-23	8.54E-25	1.875731042	2.13E-16	2.17E-17	1.5237	6.3503	5.5917
ENSMUSG00000050108	-6.8217351	9.3E-09	1.71E-09	-5.384871238	2.43E-11	3.5E-12	1.546	0.0137	0.037
ENSMUSG00000050199	-2.043108156	2.89E-43	1.05E-44	-1.95906229	1.27E-44	4.58E-46	8.13	1.9727	2.091
ENSMUSG00000050357	-2.298281335	0.00000134	0.000000309	-1.754818197	0.0000818	0.0000229	0.8673	0.1763	0.257
ENSMUSG00000050377	-7.297680549	0.003741376	0.001392573	-7.297680549	0.002864743	0.001027304	0.1573	0	0
ENSMUSG00000050592	1.682298898	2.04E-43	7.34E-45	1.713074756	8.86E-53	2.66E-54	4.0897	13.1253	13.4083
ENSMUSG00000050675	4.49272766	5.44E-68	1.15E-69	4.680485112	4.06E-76	8.3E-78	0.4153	9.3507	10.6503
ENSMUSG00000050721	2.607306859	1.79E-168	1.24E-170	2.78058411	1.25E-151	1.2E-153	7.7807	47.4127	53.4633
ENSMUSG00000050860	-2.280885824	6.56E-19	5.79E-20	-2.710753545	9.04E-21	7.32E-22	13.7807	2.8357	2.105
ENSMUSG00000050912	1.66818708	2.37E-223	1.04E-225	1.631131576	1.35E-106	2.01E-108	54.791	174.134	169.7183
ENSMUSG00000050914	2.332121954	0.000000255	5.39E-08	1.793790129	0.0000981	0.0000279	1.4197	7.1487	4.9223
ENSMUSG00000050957	1.575660249	3.1E-09	5.43E-10	1.668734905	3.78E-11	5.51E-12	4.0627	12.1097	12.9167
ENSMUSG00000050965	1.453752365	4.96E-43	1.81E-44	1.639713942	1.01E-124	1.23E-126	29.0703	79.6293	90.5843
ENSMUSG00000051166	-1.637185421	5.51E-38	2.33E-39	-1.556086174	0.006200695	0.002368713	29.282	9.4137	9.958
ENSMUSG00000051185	-1.380549484	1.03E-12	1.35E-13	-1.458824031	2.21E-16	2.25E-17	10.485	4.027	3.8143
ENSMUSG00000051212	-4.092839526	0.02860785	0.012703118	-6.684242641	3.1E-28	1.81E-29	5.1077	0.2993	0.0497
ENSMUSG00000051224	-1.461364618	0.0000206	0.00000543	-1.825542742	0.000000471	9.95E-08	5.4147	1.9663	1.5277
ENSMUSG00000051228	-2.732716121	0.014258981	0.005948258	-2.119739244	0.039389726	0.017932605	0.1507	0.0227	0.0347
ENSMUSG00000051444	1.143498022	3.45E-13	4.37E-14	1.233704596	1.14E-14	1.28E-15	3.7627	8.3123	8.8487
ENSMUSG00000051457	-1.275467794	1.1E-45	3.73E-47	-1.380154146	2.16E-50	6.76E-52	69.2527	28.6077	26.6053
ENSMUSG00000051495	-2.220680142	7.34E-73	1.46E-74	-2.162479764	1.69E-96	2.76E-98	57.8213	12.405	12.9157
ENSMUSG00000051590	2.827079939	7.16E-15	8.07E-16	3.090237552	1.45E-19	1.25E-20	0.1833	1.301	1.5613
ENSMUSG00000051615	2.948249143	1.68E-236	7.12E-239	3.157810853	2.01E-239	1.01E-241	4.3363	33.4683	38.7007
ENSMUSG00000051650	-1.296078955	2.12E-23	1.48E-24	-1.125499917	4.21E-26	2.66E-27	108.7097	44.27	49.8263
ENSMUSG00000051682	-2.954533996	0.0000037	0.000000894	-3.863257916	6.79E-09	1.2E-09	1.7803	0.2297	0.1223
ENSMUSG00000051736	-3.282612441	0.00035017	0.000109758	-2.038851559	0.006136372	0.002340329	1.8943	0.1947	0.461
ENSMUSG00000051839	-4.529131784	5.64E-59	1.42E-60	-3.534275087	3.65E-43	1.37E-44	12.976	0.562	1.12
ENSMUSG00000051890	-1.461913233	0.000262956	0.0000809	-1.878506438	0.00000154	0.000000349	1.988	0.7217	0.5407
ENSMUSG00000052040	-1.500124849	6.45E-64	1.5E-65	-1.646097789	1.92E-26	1.2E-27	28.6893	10.1423	9.1663
ENSMUSG00000052085	-1.876802646	7.2E-213	3.55E-215	-1.915173144	2.2E-128	2.54E-130	174.074	47.398	46.154
ENSMUSG00000052087	1.374995378	2.48E-19	2.12E-20	1.289286498	6.38E-17	6.29E-18	4.8663	12.6217	11.8937
ENSMUSG00000052142	2.202628882	8.57E-172	5.83E-174	2.276704421	7.06E-139	7.7E-141	11.6373	53.5687	56.391
ENSMUSG00000052234	-5.943699926	0.00000546	0.00000134	-8.663301297	7.87E-10	1.29E-10	724.6347	11.773	1.7873
ENSMUSG00000052273	-6.420662048	0.00096229	0.000323081	-6.420662048	0.000729973	0.000235311	0.0857	0	0
ENSMUSG00000052293	-1.523107367	1.18E-11	1.68E-12	-2.012156122	5.95E-16	6.23E-17	24.9883	8.6943	6.1947
ENSMUSG00000052296	-1.119765084	6.69E-70	1.37E-71	-1.145106942	6.54E-52	2E-53	138.865	63.9013	62.7887
ENSMUSG00000052310	1.168206301	6.87E-35	3.2E-36	1.205216882	3.28E-39	1.36E-40	17.7003	39.7783	40.812
ENSMUSG00000052331	-1.371703872	1.37E-17	1.3E-18	-1.438507351	1.77E-44	6.41E-46	54.349	21.0023	20.052
ENSMUSG00000052334	4.366453633	9.88E-35	4.62E-36	4.439934654	1.67E-39	6.85E-41	0.377	7.7763	8.1827
ENSMUSG00000052353	2.85544921	0.004188666	0.001573041	2.199505992	0.044727608	0.020680394	0.0197	0.1423	0.0903
ENSMUSG00000052435	-3.038747252	0.00000152	0.000000352	-3.978456709	5.62E-201	3.76E-203	74.201	9.0293	4.7073
ENSMUSG00000052485	1.606085792	0.039641024	0.018216421	1.695520876	0.013631734	0.005569774	0.3767	1.1467	1.22
ENSMUSG00000052632	1.325918955	1.75E-11	2.53E-12	2.372736061	0.0000014	0.000000316	1.2033	3.0167	6.2323
ENSMUSG00000052681	1.970481293	1.37E-75	2.6E-77	2.046370671	6.96E-76	1.44E-77	68.6157	268.904	283.4277
ENSMUSG00000052751	-1.213543971	6.39E-13	8.27E-14	-1.072582645	3.57E-09	6.15E-10	7.3507	3.1697	3.495
ENSMUSG00000052837	1.236764531	4.71E-15	5.23E-16	2.215739858	6.24E-74	1.33E-75	9.8693	23.259	45.845
ENSMUSG00000053040	-1.282527022	0.014219694	0.005927918	-1.567279644	0.01048616	0.004184618	2.757	1.1333	0.9303
ENSMUSG00000053113	1.898774412	3.64E-17	3.55E-18	2.375794376	1.15E-25	7.39E-27	1.7033	6.3517	8.8407
ENSMUSG00000053192	1.160318109	0.00000114	0.00000026	1.259757608	7.91E-10	1.3E-10	3.2813	7.334	7.8573
ENSMUSG00000053226	-1.796555521	0.000585085	0.000189406	-1.745625932	0.000587751	0.000186746	1.708	0.4917	0.5093
ENSMUSG00000053310	2.491150851	4.95E-39	2.04E-40	2.412284336	4.78E-43	1.8E-44	4.7643	26.7863	25.3613
ENSMUSG00000053338	-5.770947353	0.000000172	3.56E-08	-4.776340612	2.87E-09	4.91E-10	2.439	0.0447	0.089
ENSMUSG00000053475	-10.70592032	2.34E-09	4.06E-10	-10.70592032	1.95E-09	3.28E-10	1.6703	0	0
ENSMUSG00000053477	1.095424551	5.32E-24	3.63E-25	1.19893882	1.91E-24	1.31E-25	9.6447	20.6083	22.1413
ENSMUSG00000053560	2.504768853	4.19E-19	3.66E-20	3.15605066	1.4E-115	1.87E-117	14.055	79.7703	125.2843
ENSMUSG00000053574	-2.868025628	0.007071258	0.002772994	-1.653738256	0.040573277	0.01853866	0.3723	0.051	0.1183
ENSMUSG00000053604	-1.901151941	7.7E-37	3.39E-38	-1.839960075	7.37E-54	2.15E-55	39.454	10.563	11.0207
ENSMUSG00000053693	-1.86093414	0.000000855	0.000000192	-2.043467235	4.75E-09	8.29E-10	1.4263	0.3927	0.346
ENSMUSG00000053702	-3.816115048	0.0000277	0.00000743	-6.359465359	5.81E-13	7.32E-14	9.3057	0.6607	0.1133
ENSMUSG00000053799	1.030149236	2.65E-48	8.42E-50	1.186126243	2.53E-32	1.3E-33	19.5333	39.8917	44.4463
ENSMUSG00000053820	-4.006426269	0.003176831	0.00116745	-10.98370619	0.0000393	0.0000106	2.025	0.126	0
ENSMUSG00000053835	1.944580773	0.000000114	2.32E-08	2.243302926	2.48E-09	4.22E-10	0.6523	2.511	3.0887
ENSMUSG00000053914	-3.772211092	7.69E-10	1.27E-10	-5.388537764	7.46E-10	1.22E-10	1.2707	0.093	0.0303
ENSMUSG00000054021	1.532674208	2.68E-61	6.52E-63	1.382125664	6.94E-41	2.76E-42	29.861	86.3943	77.8333
ENSMUSG00000054027	1.104243373	5.89E-14	7.05E-15	1.45385379	4.99E-24	3.49E-25	3.5967	7.7323	9.8527
ENSMUSG00000054115	1.182563375	7.25E-37	3.18E-38	1.056840519	1.52E-23	1.09E-24	10.435	23.6853	21.7087
ENSMUSG00000054136	-2.846279572	2.12E-22	1.55E-23	-3.6858149	5.38E-31	2.87E-32	9.2913	1.292	0.722
ENSMUSG00000054150	2.308983075	1.42E-137	1.31E-139	2.515853896	8.82E-146	9.01E-148	8.426	41.7537	48.1913
ENSMUSG00000054191	-3.893506103	4.89E-111	5.74E-113	-4.040470409	1.03E-102	1.61E-104	31.1497	2.096	1.893
ENSMUSG00000054200	-2.76225245	0.0000254	0.00000676	-2.830285657	0.000127648	0.0000368	1.522	0.2243	0.214
ENSMUSG00000054277	-1.812012584	1.13E-67	2.42E-69	-1.734096012	2.28E-33	1.14E-34	33.093	9.4247	9.9477
ENSMUSG00000054342	1.600394996	3.15E-32	1.58E-33	1.658516971	1.29E-47	4.33E-49	8.3377	25.282	26.3213
ENSMUSG00000054385	2.872099771	5.41E-28	3.11E-29	2.873610679	9.96E-25	6.72E-26	0.7387	5.408	5.4137
ENSMUSG00000054404	2.19086279	4.61E-09	8.21E-10	1.781389043	1.2E-10	1.83E-11	0.8943	4.0833	3.0743
ENSMUSG00000054469	-1.927946419	2.55E-47	8.3E-49	-1.828781265	1.97E-82	3.73E-84	41.8197	10.9903	11.7723
ENSMUSG00000054568	3.52973298	0.029915553	0.013358609	3.488688794	0.00812615	0.003180329	0.0237	0.2733	0.2657
ENSMUSG00000054619	-3.8547187	1.37E-169	9.42E-172	-3.998511893	0	0	115.955	8.015	7.2547
ENSMUSG00000054675	6.135596791	1.06E-37	4.55E-39	7.079565591	5.02E-171	4.19E-173	0.418	29.3883	56.5377
ENSMUSG00000054733	-2.39633204	1.98E-14	2.29E-15	-1.863599498	1.59E-15	1.7E-16	9.4763	1.8	2.604
ENSMUSG00000054814	1.101181166	1.64E-13	2.03E-14	1.108202123	1.08E-13	1.29E-14	4.1087	8.8143	8.8573
ENSMUSG00000054843	-1.914047441	3.48E-67	7.52E-69	-2.132642643	1.29E-76	2.61E-78	14.6173	3.8787	3.3333
ENSMUSG00000054855	-2.173431568	2.56E-21	1.97E-22	-2.058310089	8.44E-24	5.99E-25	11.2593	2.496	2.7033
ENSMUSG00000054892	-2.444784843	0.0000146	0.00000376	-2.64129463	0.000108267	0.000031	1.4537	0.267	0.233
ENSMUSG00000054901	-8.116802615	1.34E-09	2.27E-10	-10.61930296	7.18E-12	9.85E-13	1.573	0.0057	0
ENSMUSG00000054920	-1.418912934	9.5E-69	1.97E-70	-1.402578274	2.11E-43	7.9E-45	55.418	20.726	20.962
ENSMUSG00000054931	1.462502273	0.003007608	0.00109983	1.970105891	0.00000762	0.00000187	0.3413	0.9407	1.3373
ENSMUSG00000055172	-2.667641062	6.39E-29	3.58E-30	-2.621013386	4.61E-60	1.19E-61	14.2497	2.2427	2.3163
ENSMUSG00000055202	1.894817763	0.000653198	0.000212998	2.195850634	0.0000795	0.0000223	0.192	0.714	0.8797
ENSMUSG00000055210	-1.237795504	0.013570413	0.00563085	-1.217572	0.002606083	0.000928251	1.7263	0.732	0.7423
ENSMUSG00000055322	2.287510157	4.65E-155	3.59E-157	2.267418372	2.71E-216	1.57E-218	5.4947	26.8257	26.4547
ENSMUSG00000055447	-2.06051718	1.74E-55	4.68E-57	-2.200179639	1.21E-111	1.71E-113	336.1433	80.5837	73.1483
ENSMUSG00000055485	3.187607159	5.34E-103	6.72E-105	3.336434824	6.87E-208	4.27E-210	1.4777	13.463	14.926
ENSMUSG00000055538	2.975196609	0.000046	0.0000127	3.197986084	0.00000456	0.00000109	0.066	0.519	0.6057
ENSMUSG00000055541	-7.950934928	1.19E-20	9.5E-22	-8.29101037	2.84E-27	1.72E-28	8.248	0.0333	0.0263
ENSMUSG00000055567	-8.763765654	0.00000283	0.000000673	-8.348728154	0.0000507	0.0000139	0.4347	0	0.0013
ENSMUSG00000055717	-2.725790519	5.15E-44	1.82E-45	-2.819031863	1.66E-43	6.18E-45	12.86	1.944	1.8223
ENSMUSG00000055835	-1.692305323	4.89E-13	6.26E-14	-1.747091126	7.07E-13	8.96E-14	12.0263	3.7213	3.5827
ENSMUSG00000055917	-1.000451732	0.00000162	0.000000375	-1.018729478	1.67E-08	3.07E-09	10.6473	5.322	5.255
ENSMUSG00000056130	-1.59809428	2.65E-22	1.95E-23	-1.798986297	1.4E-24	9.56E-26	6.4747	2.1387	1.8607
ENSMUSG00000056185	-1.217247217	0.000129158	0.0000381	-1.602416582	3.84E-08	7.27E-09	5.1003	2.1937	1.6797

ENSMUSG00000056215	-3.180698526	1.41E-10	2.19E-11	-2.715034954	7.65E-11	1.14E-11	0.7617	0.084	0.116
ENSMUSG00000056220	3.079311038	0	0	3.029748256	9.09E-219	5.15E-221	21.4993	181.7147	175.578
ENSMUSG00000056234	-2.09755621	4.35E-08	8.5E-09	-2.46626867	7.22E-155	6.73E-157	279.0897	65.2103	50.5037
ENSMUSG00000056258	-7.872418378	6.3E-09	1.13E-09	-7.872418378	4.98E-09	8.7E-10	0.2343	0	0
ENSMUSG00000056399	-9.21416506	1.63E-32	8.09E-34	-10.38555102	2.64E-284	9.47E-287	586.8093	0.988	0.4387
ENSMUSG00000056515	1.840012619	5.27E-175	3.51E-177	2.013230502	2.79E-108	4.09E-110	17.6293	63.1153	71.167
ENSMUSG00000056643	-7.927080755	3.04E-09	5.33E-10	-7.970149477	2.87E-09	4.91E-10	2.7583	0.0113	0.011
ENSMUSG00000056656	7.22881869	0.006977306	0.002732758	6.705056346	0.024808265	0.010788957	0	0.15	0.1043
ENSMUSG00000056749	-1.087301074	8.86E-14	1.07E-14	-1.048211949	2.46E-09	4.18E-10	16.3493	7.6947	7.906
ENSMUSG00000056832	-1.309431999	0.00021077	0.0000639	-1.628647101	0.00000527	0.00000127	1.207	0.487	0.3903
ENSMUSG00000056888	-2.105061613	0.045971373	0.021472265	-3.778803138	1.46E-157	1.32E-159	68.4087	15.901	4.984
ENSMUSG00000057060	-1.506156358	0.000000439	9.54E-08	-1.513286074	0.000000498	0.000000105	3.8413	1.3523	1.3457
ENSMUSG00000057069	-1.624055777	1.17E-52	3.27E-54	-1.737393177	9.48E-58	2.52E-59	20.9973	6.812	6.2973
ENSMUSG00000057074	6.727920455	0.044073148	0.020482762	7.294620749	0.006751116	0.002599953	0	0.106	0.157
ENSMUSG00000057137	4.847643442	4.85E-35	2.25E-36	5.093891424	3.95E-35	1.85E-36	0.2173	6.2577	7.4223
ENSMUSG00000057191	3.385737351	9.97E-76	1.88E-77	3.119695128	6.54E-74	1.39E-75	3.087	32.266	26.8323
ENSMUSG00000057378	4.942514505	0.000209732	0.0000635	6.262094845	0.000000132	2.62E-08	0.0027	0.082	0.2047
ENSMUSG00000057596	12.41159926	2.4E-16	2.45E-17	12.32708477	5.4E-16	5.63E-17	0	5.4483	5.1383
ENSMUSG00000057706	1.05563244	1.13E-12	1.49E-13	1.334044873	1.57E-18	1.41E-19	3.5017	7.2787	8.828
ENSMUSG00000057729	2.762590899	0.00000548	0.00000135	1.72635644	0.004232067	0.001569056	0.6733	4.5693	2.228
ENSMUSG00000057751	-3.745427173	0.01197591	0.00491682	-6.247927513	0.002016558	0.000703373	0.076	0.0057	0
ENSMUSG00000057858	-1.829305896	4.11E-86	6.79E-88	-1.904146405	1.32E-78	2.66E-80	102.7257	28.907	27.4457
ENSMUSG00000058099	1.430161714	9.98E-30	5.4E-31	1.169053689	4.84E-25	3.21E-26	6.073	16.3653	13.656
ENSMUSG00000058186	1.510292176	5.3E-15	5.92E-16	1.126357587	1.94E-08	3.59E-09	1.26	3.5893	2.7507
ENSMUSG00000058216	-11.08967116	0.000000633	0.00000014	-6.231690166	0.0000154	0.00000392	2.1793	0	0.029
ENSMUSG00000058252	-5.136243281	0.000000173	3.58E-08	-5.63393196	1.17E-47	3.9E-49	10.5273	0.2993	0.212
ENSMUSG00000058503	-1.010900738	2.07E-20	1.68E-21	-1.000189205	3.66E-18	3.36E-19	38.1277	18.9203	19.0613
ENSMUSG00000058756	-1.24984519	1.53E-15	1.64E-16	-1.153059685	2.96E-14	3.43E-15	19.73	8.2963	8.872
ENSMUSG00000059182	1.511220437	2.55E-107	3.1E-109	1.515272405	1.32E-61	3.34E-63	30.185	86.0427	86.2847
ENSMUSG00000059248	1.629411704	6.75E-62	1.62E-63	1.627335842	4.16E-59	1.09E-60	27.425	84.8493	84.7273
ENSMUSG00000059336	8.259144067	9.91E-37	4.38E-38	9.253826002	2.26E-37	9.86E-39	0.0367	11.2337	22.3847
ENSMUSG00000059429	1.41781994	0.000411208	0.000130233	1.684209318	0.00000138	0.000000311	0.259	0.692	0.8323
ENSMUSG00000059430	2.220847064	6.94E-54	1.91E-55	1.914096573	5.35E-82	1.02E-83	23.752	110.724	89.516
ENSMUSG00000059456	2.120591118	6.21E-109	7.34E-111	2.385412608	2.06E-109	2.97E-111	9.8637	42.8943	51.537
ENSMUSG00000059495	-3.745264917	0.0000195	0.00000512	-3.943204295	0.0000054	0.0000013	0.1743	0.013	0.0113
ENSMUSG00000059708	-1.639904037	1.86E-13	2.31E-14	-2.700724977	8.72E-15	9.77E-16	1.686	0.541	0.2593
ENSMUSG00000059810	6.278992155	7.01E-09	1.27E-09	5.387493964	0.0000235	0.00000614	0.0357	2.7697	1.493
ENSMUSG00000059852	3.779851407	0.000994165	0.000334957	4.340873263	0.0000314	0.0000083	0.029	0.3983	0.5877
ENSMUSG00000059895	2.374138815	4.59E-123	4.88E-125	2.494700217	4.3E-122	5.32E-124	46.4373	240.7433	261.726
ENSMUSG00000059900	2.398125641	1.23E-33	5.85E-35	2.6955866	6.55E-67	1.53E-68	3.7183	19.6	24.088
ENSMUSG00000059923	-1.150094081	1.54E-49	4.67E-51	-1.088516669	7.88E-24	5.59E-25	189.5623	85.416	89.1407
ENSMUSG00000059970	-1.574102037	2.9E-23	2.04E-24	-1.586842372	2.66E-37	1.16E-38	18.1747	6.104	6.0503
ENSMUSG00000060002	-2.751490206	6.93E-54	1.91E-55	-2.849412541	1.67E-61	4.23E-63	10.604	1.5747	1.4713
ENSMUSG00000060063	1.433125415	3.36E-32	1.69E-33	1.565239478	9.58E-38	4.14E-39	29.1813	78.7987	86.3553
ENSMUSG00000060131	-2.217435477	5.24E-08	1.03E-08	-2.774732586	1.66E-116	2.18E-118	75.8647	16.3127	11.0857
ENSMUSG00000060147	-1.00868234	0.0000301	0.00000812	-1.210321067	0.00000406	0.000000964	9.9443	4.9423	4.2977
ENSMUSG00000060216	2.134340772	3.16E-161	2.35E-163	2.113723891	2.99E-155	2.76E-157	36.29	159.3263	157.0657
ENSMUSG00000060224	1.312409017	1.22E-20	9.75E-22	1.432744736	1.11E-28	6.4E-30	4.4807	11.128	12.096
ENSMUSG00000060376	-1.24627727	2.56E-17	2.46E-18	-1.319080252	3.47E-30	1.91E-31	27.4133	11.5557	10.987
ENSMUSG00000060470	1.014049976	4.51E-17	4.41E-18	1.383396797	5.19E-12	7.07E-13	7.4083	14.9617	19.327
ENSMUSG00000060579	1.825835029	0.001865152	0.000658731	1.698048412	0.003864569	0.001422932	0.7277	2.5797	2.361
ENSMUSG00000060600	2.284113619	2.35E-66	5.19E-68	2.0202114	3.41E-55	9.57E-57	13.2017	64.3007	53.5517
ENSMUSG00000061046	-1.365218981	0.0000401	0.000011	-1.079522321	0.000585296	0.000185926	4.8543	1.8843	2.297
ENSMUSG00000061143	1.99884649	1.26E-76	2.36E-78	2.241358407	2.3E-81	4.43E-83	2.0853	8.3347	9.8603
ENSMUSG00000061186	1.127397188	4.52E-46	1.51E-47	1.074486201	2.24E-45	7.9E-47	8.1437	17.791	17.1503
ENSMUSG00000061411	2.870220954	3.35E-127	3.33E-129	3.077995373	2.8E-213	1.67E-215	2.659	19.442	22.4537
ENSMUSG00000061451	-3.166773291	5.59E-29	3.12E-30	-3.020922424	7.21E-42	2.81E-43	5.487	0.611	0.676
ENSMUSG00000061533	-1.071441537	8.27E-20	6.94E-21	-1.142517178	2.13E-16	2.17E-17	23.2717	11.0737	10.5413
ENSMUSG00000061665	1.064838939	6.32E-58	1.65E-59	1.085422917	2.03E-52	6.12E-54	14.0713	29.4363	29.8593
ENSMUSG00000061751	5.126309153	3.8E-28	2.18E-29	5.404105912	3.97E-41	1.57E-42	0.148	5.1693	6.267
ENSMUSG00000061815	12.33882922	1.09E-15	1.15E-16	12.6173144	1.49E-16	1.51E-17	0	5.1803	6.2833
ENSMUSG00000061859	2.213169183	2.23E-37	9.65E-39	2.060545454	5.65E-21	4.53E-22	2.5257	11.7113	10.5357
ENSMUSG00000061928	-5.721668861	6.46E-09	1.17E-09	-6.49927644	5.14E-08	9.84E-09	0.8443	0.016	0.0093
ENSMUSG00000062082	-1.636401741	0.048682789	0.022902386	-1.990772657	1.22E-22	9.05E-24	24.6763	7.9373	6.2087
ENSMUSG00000062232	-2.7540831	2.32E-218	1.09E-220	-2.646188137	4.84E-171	4.01E-173	21.516	3.1893	3.437
ENSMUSG00000062590	-1.099318271	0.002581379	0.000933923	-1.059123582	0.006053806	0.002304242	5.0557	2.3597	2.4263
ENSMUSG00000062593	2.49811794	0.000154528	0.0000461	3.115729815	2.05E-09	3.46E-10	9.445	53.3593	81.871
ENSMUSG00000062713	-4.222151631	0.000901645	0.000301789	-5.04475538	2.02E-15	2.18E-16	1.3313	0.0713	0.0403
ENSMUSG00000062826	-4.849303104	0.000610512	0.000198146	-7.787902559	0.00000888	0.0000022	0.221	0.0077	0
ENSMUSG00000062861	2.74945502	2.11E-87	3.42E-89	2.859723465	6.36E-85	1.17E-86	2.7297	18.356	19.814
ENSMUSG00000062901	-1.1495195	2.3E-13	2.87E-14	-1.264825244	2.68E-25	1.75E-26	17.6267	7.9457	7.3353
ENSMUSG00000062937	5.199112165	0	0	5.187452286	0	0	15.8527	582.3593	577.6717
ENSMUSG00000062980	8.686625582	2.46E-15	2.68E-16	8.305736061	1.27E-13	1.53E-14	0.0093	3.8457	2.9533
ENSMUSG00000062995	1.067807757	7.4E-43	2.72E-44	1.199169459	1.83E-46	6.26E-48	27.6653	57.9933	63.5217
ENSMUSG00000063129	-4.814875504	3.86E-11	5.74E-12	-3.200883955	3.12E-09	5.34E-10	2.308	0.082	0.251
ENSMUSG00000063160	-1.492100242	0.000000287	6.13E-08	-2.271913502	4.08E-16	4.22E-17	3.7703	1.3403	0.7807
ENSMUSG00000063275	1.070036869	0.000000419	0.000000091	1.028937017	0.000000953	0.00000021	6.433	13.506	13.1267
ENSMUSG00000063382	1.28970168	3.68E-27	2.19E-28	1.467826882	1.37E-16	1.38E-17	3.4737	8.4923	9.6083
ENSMUSG00000063450	4.296241451	0.019240483	0.008253559	4.317678622	0.007283486	0.002822074	0.0057	0.1113	0.113
ENSMUSG00000063455	-2.894131365	0.00146689	0.000508495	-3.067462968	0.000611113	0.000194506	0.1313	0.0177	0.0157
ENSMUSG00000063535	-1.125330494	0.0000177	0.00000463	-1.152693533	0.00000528	0.00000127	2.3997	1.1	1.0793
ENSMUSG00000063605	-1.146860477	0.002001979	0.000711088	-1.812441438	7.15E-09	1.26E-09	3.599	1.6253	1.0247
ENSMUSG00000063683	-5.219353525	0.000000355	7.66E-08	-6.637457327	1.32E-43	4.87E-45	7.7987	0.2093	0.0783
ENSMUSG00000063851	1.508693789	0.017775502	0.007563386	2.102294174	0.000582731	0.00018507	0.4877	1.3877	2.094
ENSMUSG00000063873	-2.096113353	0.000000106	2.15E-08	-1.007576678	0.006304633	0.002411466	1.4637	0.3423	0.728
ENSMUSG00000064120	-1.009002929	3.94E-10	6.36E-11	-1.219676496	3.41E-12	4.56E-13	12.324	6.1237	5.2917
ENSMUSG00000064125	1.611089528	0.003104977	0.00113824	1.43506707	0.014182123	0.005829906	0.152	0.4643	0.411
ENSMUSG00000064147	-4.016418661	0.0000112	0.00000287	-6.866396921	0	0	77.8247	4.809	0.667
ENSMUSG00000064179	1.484685582	1.28E-08	2.39E-09	1.008659804	0.0000869	0.0000245	3.9863	11.156	8.0207
ENSMUSG00000064220	-2.020508334	0.040682083	0.018745694	-2.082203002	0.01189139	0.004797935	4.0383	0.9953	0.9537
ENSMUSG00000064368	-1.068063957	3.34E-21	2.6E-22	-1.094195949	2.77E-14	3.21E-15	914.4783	436.1683	428.339
ENSMUSG00000066007	1.034953706	0.002398183	0.000863479	1.106290706	0.001032101	0.000341112	0.5777	1.1837	1.2437
ENSMUSG00000066026	2.818324795	1.57E-113	1.81E-115	2.998144695	2.52E-199	1.71E-201	11.704	82.5533	93.5117
ENSMUSG00000066440	-1.312034861	3.32E-43	1.21E-44	-1.361468221	7.98E-32	4.19E-33	10.2213	4.1167	3.978
ENSMUSG00000066442	-3.047953833	5.2E-09	9.29E-10	-2.377304852	0.00000127	0.000000284	4.7003	0.5683	0.9047
ENSMUSG00000066510	8.044394119	0.006919439	0.002709132	8.26366044	0.002583526	0.000919859	0	0.264	0.3073
ENSMUSG00000066687	-1.535803077	5.67E-49	1.77E-50	-1.529715518	5.61E-24	3.93E-25	25.5997	8.829	8.8663
ENSMUSG00000066721	1.948225864	1.48E-12	1.97E-13	1.575145908	8.25E-08	1.62E-08	0.9503	3.6673	2.8317
ENSMUSG00000066735	-1.30612466	4.09E-29	2.27E-30	-1.170235039	1.05E-20	8.53E-22	34.5157	13.9583	15.337
ENSMUSG00000066894	-1.192989703	0.0000284	0.00000762	-1.290906902	1.35E-08	2.45E-09	3.5887	1.5697	1.4667
ENSMUSG00000066952	-2.719635716	0.000119364	0.0000351	-4.370687407	0.0000576	0.0000159	0.4413	0.067	0.0213
ENSMUSG00000067212	1.496718093	8.22E-52	2.35E-53	1.597066537	1.77E-57	4.75E-59	16.1817	45.6647	48.954
ENSMUSG00000067220	-2.726846839	0.00000775	0.00000194	-4.056552285	9.29E-15	1.04E-15	2.4627	0.372	0.148
ENSMUSG00000067399	-7.125954999	0.000000296	6.32E-08	-4.645523082	7.52E-08	1.47E-08	1.4433	0.0103	0.0577
ENSMUSG00000067455	-4.467406736	0.01694548	0.007180787	-3.467406736	0.043949224	0.020284491	0.6047	0.0273	0.0547
ENSMUSG00000067629	-1.517773631	6.78E-16	7.06E-17	-1.368035466	2.72E-24	1.88E-25	10.8307	3.7823	4.196
ENSMUSG00000067813	-3.725543541	0.000770035	0.000254307	-9.35974956	0.00000396	0.000000941	0.657	0.0497	0
ENSMUSG00000067851	-1.999067407	6.34E-133	5.99E-135	-2.137917232	4.57E-223	2.52E-225	121.1403	30.3047	27.524
ENSMUSG00000067889	-1.290262351	0.000000078	1.56E-08	-1.143465669	0.00000016	3.21E-08	1.6973	0.694	0.7683
ENSMUSG00000068129	-1.238355405	1.79E-19	1.52E-20	-1.369441327	7.48E-32	3.92E-33	74.9343	31.7613	29.0027
ENSMUSG00000068220	-2.056858164	0.004176543	0.001568199	-2.817529441	1.43E-96	2.32E-98	495.6627	119.127	70.3113
ENSMUSG00000068227	3.247188533	3.18E-11	4.69E-12	2.704871964	1.56E-10	2.41E-11	0.3083	2.9277	2.0103
ENSMUSG00000068699	-2.741496234	2.07E-10	3.26E-11	-3.882760042	4.63E-25	3.07E-26	1.0277	0.1537	0.0697
ENSMUSG00000068744	1.244013166	2.6E-23	1.82E-24	1.127581155	9.38E-17	9.3E-18	9.395	22.2527	20.5273
ENSMUSG00000068758	-1.641330856	7.02E-22	5.27E-23	-1.419136859	3.24E-11	4.69E-12	25.9233	8.31	9.6937
ENSMUSG00000069830	-8.786269628	4.23E-24	2.86E-25	-10.43012582	5.67E-17	5.57E-18	7.3583	0.0167	0.0053
ENSMUSG00000069833	5.07099776	1.87E-40	7.43E-42	5.420706249	1.05E-51	3.22E-53	0.0423	1.423	1.8133
ENSMUSG00000069892	9.781031791	6.56E-19	5.79E-20	9.401306206	3.09E-17	3.01E-18	0.0067	5.8653	4.508
ENSMUSG00000069893	9.243967327	0.00000143	0.000000332	8.940558308	0.00000257	0.000000594	0	0.6063	0.4913
ENSMUSG00000070323	-2.966391607	0.025133481	0.011048605	-5.678266221	0.00083798	0.000272847	0.495	0.0633	0.0097
ENSMUSG00000070348	3.087207971	9.45E-10	1.58E-10	2.87032769	1.66E-08	3.04E-09	0.111	0.9433	0.8117
ENSMUSG00000070469	8.071462363	6.37E-08	1.26E-08	8.367778746	8.66E-09	1.54E-09	0	0.269	0.3303
ENSMUSG00000070690	1.929166519	2.06E-08	3.9E-09	2.340955619	5.6E-14	6.63E-15	0.6227	2.3713	3.1547
ENSMUSG00000070732	-1.337861636	0.000129829	0.0000383	-1.226479719	0.00000352	0.000000831	3.3383	1.3207	1.4267
ENSMUSG00000070733	-1.744508356	7.87E-109	9.35E-111	-1.953870829	3.39E-41	1.34E-42	37.118	11.0773	9.581
ENSMUSG00000070737	-1.625975465	3.57E-11	5.29E-12	-1.894167088	1.56E-14	1.77E-15	10.668	3.4563	2.87
ENSMUSG00000070780	-7.466095349	7.92E-09	1.44E-09	-7.973055338	7.59E-09	1.34E-09	1.5913	0.009	0.0063
ENSMUSG00000071076	-2.272147324	3.06E-58	7.86E-60	-2.038588498	3.52E-72	7.65E-74	72.292	14.966	17.596
ENSMUSG00000071113	1.66058629	0.048180041	0.022642444	1.730295262	0.013761202	0.005634074	0.2023	0.6397	0.6713
ENSMUSG00000071470	1.324782699	0.00000905	0.00000228	1.115678098	0.000468549	0.000146575	3.1303	7.8413	6.7833
ENSMUSG00000071552	-1.590577321	0.032536292	0.014680321	-1.333075088	0.043020819	0.019808467	2.317	0.7693	0.9197
ENSMUSG00000071637	-2.209672175	2.56E-58	6.56E-60	-2.197918437	9.59E-69	2.15E-70	16.21	3.5043	3.533
ENSMUSG00000071648	-2.320515419	3.05E-14	3.57E-15	-2.686164891	6.63E-20	5.63E-21	6.805	1.3623	1.0573
ENSMUSG00000071656	2.438176649	4.98E-11	7.48E-12	2.828853527	3.79E-18	3.49E-19	0.4123	2.2347	2.9297
ENSMUSG00000071669	1.322894221	0.000536232	0.000172822	1.835083111	0.000000178	0.000000036	0.8957	2.2407	3.1957
ENSMUSG00000071713	-4.679108817	4.38E-11	6.55E-12	-6.007816593	0	0	438.0577	17.0993	6.8077
ENSMUSG00000071714	-4.61404543	2.75E-19	2.37E-20	-5.293405356	0	0	389.6727	15.9123	9.9363
ENSMUSG00000072419	-4.647830559	0.014922136	0.006251852	-4.461417435	0.012937812	0.005254981	0.2423	0.0097	0.011
ENSMUSG00000072595	2.94660363	6.89E-15	7.76E-16	2.956008362	3.09E-17	3.01E-18	0.8727	6.7277	6.7717
ENSMUSG00000072707	-1.23482657	0.007909623	0.003133079	-1.600251036	0.0000103	0.00000256	1.6443	0.6987	0.5423
ENSMUSG00000072825	-1.342513637	4.05E-49	1.25E-50	-1.047295489	1.77E-13	2.15E-14	18.8107	7.4177	9.102
ENSMUSG00000072889	-1.496330281	1.05E-42	3.89E-44	-1.32362224	3.92E-36	1.8E-37	25.7043	9.111	10.2697
ENSMUSG00000072915	-1.169925001	0.002154687	0.00076997	-1.366530507	0.0000906	0.0000256	1.407	0.6253	0.5457
ENSMUSG00000073001	11.32211593	0.00000417	0.00000101	11.31326097	0.000000495	0.000000105	0	2.5603	2.5447
ENSMUSG00000073413	9.330169948	0.011859877	0.004864239	9.917372079	0.001050027	0.000347544	0	0.6437	0.967
ENSMUSG00000073591	-2.719399721	3.08E-09	5.4E-10	-3.425305835	6.94E-16	7.28E-17	1.3787	0.2093	0.1283
ENSMUSG00000073600	-2.047908892	3.63E-18	3.33E-19	-2.543286584	1.4E-27	8.42E-29	4.572	1.1057	0.7843

ENSMUSG00000073802	6.194871359	0.0000171	0.00000444	6.4425578	0.00000425	0.00000101	0.0143	1.05	1.2467
ENSMUSG00000073987	-1.566439905	5.51E-15	6.16E-16	-1.744619971	5.31E-56	1.47E-57	38.153	12.882	11.3853
ENSMUSG00000074028	-3.640855263	0.000706274	0.000231434	-5.000751208	0.000173685	0.000051	0.6403	0.0513	0.02
ENSMUSG00000074151	2.16275577	2.17E-29	1.19E-30	2.438785544	9.47E-43	3.62E-44	1.0983	4.918	5.955
ENSMUSG00000074220	-1.380226735	0.002144691	0.000765951	-1.592774948	0.0000186	0.00000478	5.555	2.134	1.8417
ENSMUSG00000074227	-2.037479528	4.31E-53	1.2E-54	-1.985684052	2.72E-54	7.92E-56	57.7257	14.0613	14.5753
ENSMUSG00000074305	2.579808441	2.6E-208	1.3E-210	2.685066849	9.32E-256	3.99E-258	6.839	40.8877	43.9823
ENSMUSG00000074342	3.189365949	9.93E-17	9.93E-18	3.413087938	1.14E-72	2.47E-74	2.2987	20.9687	24.486
ENSMUSG00000074344	10.35571815	0.00000726	0.00000181	10.22037833	0.0000209	0.00000541	0	1.3103	1.193
ENSMUSG00000074364	2.633821498	2.97E-158	2.23E-160	2.611094208	1E-151	9.55E-154	10.401	64.5557	63.5467
ENSMUSG00000074480	1.147977332	5.73E-11	8.66E-12	1.112764814	4.42E-09	7.69E-10	1.1603	2.5713	2.5093
ENSMUSG00000074491	9.674192268	0.0000491	0.0000137	10.75210184	1.73E-08	3.18E-09	0	0.817	1.7247
ENSMUSG00000074527	-1.666956765	0.009989141	0.004040068	-1.618701065	0.000170409	0.00005	18.9203	5.9583	6.161
ENSMUSG00000074577	5.806268598	1.22E-93	1.74E-95	6.059375905	4.53E-122	5.64E-124	0.2293	12.833	15.294
ENSMUSG00000074604	3.560252971	1.26E-105	1.55E-107	3.490236174	1.78E-73	3.82E-75	6.8113	80.348	76.5417
ENSMUSG00000074652	1.462435549	0.0000118	0.00000302	1.413127909	0.0000147	0.00000375	0.378	1.0417	1.0067
ENSMUSG00000074657	-1.784271309	0.00000262	0.000000623	-2.487415236	1.42E-10	2.18E-11	0.9197	0.267	0.164
ENSMUSG00000074671	1.014213572	4.87E-24	3.3E-25	1.009763185	6.49E-19	5.78E-20	8.198	16.5583	16.5073
ENSMUSG00000074785	1.97930253	4.21E-15	4.66E-16	1.82694304	1.22E-10	1.86E-11	0.4563	1.7993	1.619
ENSMUSG00000074793	1.494903193	0.000572573	0.000185077	1.375866902	0.001871443	0.000648493	0.448	1.2627	1.1627
ENSMUSG00000074794	-1.716114762	7.27E-32	3.68E-33	-1.306392487	1.36E-11	1.92E-12	8.1897	2.4927	3.3113
ENSMUSG00000074802	1.874571873	1.81E-26	1.11E-27	1.905776568	2.83E-39	1.17E-40	3.829	14.0407	14.3477
ENSMUSG00000074825	1.342545496	1.17E-37	5.03E-39	1.304775244	4.6E-44	1.68E-45	10.8243	27.4503	26.741
ENSMUSG00000074874	6.457481333	6.56E-32	3.31E-33	6.906280937	4.87E-37	2.16E-38	0.1907	16.756	22.8703
ENSMUSG00000074899	4.297932614	1.46E-40	5.78E-42	4.446188003	5.5E-43	2.08E-44	0.0647	1.272	1.4097
ENSMUSG00000074918	1.949304575	3.69E-12	5.05E-13	2.23976188	2.19E-67	5.05E-69	9.8443	38.0177	46.4967
ENSMUSG00000074923	1.713251036	2.31E-12	3.11E-13	1.962246373	9.82E-18	9.24E-19	1.055	3.4593	4.111
ENSMUSG00000075054	1.504455717	1.08E-15	1.15E-16	1.36416952	2.81E-17	2.73E-18	4.207	11.936	10.83
ENSMUSG00000075078	-1.516160688	0.000441733	0.000140606	-1.729453198	0.00000298	0.000000697	2.5113	0.878	0.7573
ENSMUSG00000075122	3.309240577	0.000278228	0.0000858	3.040157126	0.0000463	0.0000126	0.0723	0.717	0.595
ENSMUSG00000075254	-1.602579176	6.07E-25	3.96E-26	-1.520179209	1.67E-30	9.09E-32	5.2183	1.7183	1.8193
ENSMUSG00000075415	2.411544011	2.38E-290	7.44E-293	2.416148385	3.29E-253	1.48E-255	27.4783	146.1967	146.664
ENSMUSG00000075706	1.72751501	1.38E-102	1.75E-104	1.807180119	5.59E-66	1.32E-67	58.9553	195.2347	206.3187
ENSMUSG00000076435	-1.907917516	3.25E-23	2.29E-24	-2.099370701	1.47E-26	9.16E-28	11.243	2.996	2.6237
ENSMUSG00000076757	-3.734579638	7.33E-19	6.52E-20	-5.297410969	4.17E-21	3.33E-22	8.324	0.6253	0.2117
ENSMUSG00000078137	2.6617496	0.0000129	0.00000333	3.150559677	4.06E-08	7.71E-09	0.0833	0.5273	0.74
ENSMUSG00000078234	-3.202855277	0.0000618	0.0000174	-3.319894698	0.0000372	0.00000997	0.2363	0.0257	0.0237
ENSMUSG00000078249	1.158834013	9.34E-51	2.75E-52	1.274130817	1.48E-54	4.26E-56	167.697	374.4287	405.5803
ENSMUSG00000078498	-5.296457407	0.001837807	0.000648563	-5.226068079	0.001552264	0.000530496	0.262	0.0067	0.007
ENSMUSG00000078606	-6.322214316	0.000000118	0.000000024	-7.392603643	7.26E-22	5.55E-23	1.6803	0.021	0.01
ENSMUSG00000078624	-1.265466479	0.000777215	0.000257002	-1.49933422	0.000310234	0.0000943	0.9103	0.3787	0.322
ENSMUSG00000078653	-1.704752945	0.000421417	0.000133612	-2.599443087	0.000000304	6.28E-08	1.5353	0.471	0.2533
ENSMUSG00000078670	-7.851749041	0.000793979	0.000262987	-7.851749041	0.000600978	0.000191156	0.231	0	0
ENSMUSG00000078716	-2.062951906	0.000569699	0.000184108	-2.426974119	0.000289095	0.0000875	0.5933	0.142	0.1103
ENSMUSG00000078851	-2.059839409	0.00000644	0.0000016	-2.268110722	3.28E-08	6.18E-09	2.5433	0.61	0.528
ENSMUSG00000078853	1.854167782	0.0000442	0.0000122	1.762592945	0.000032	0.00000849	0.5487	1.9837	1.8617
ENSMUSG00000078865	-1.100208625	0.00000206	0.000000484	-1.120089485	0.000000303	6.26E-08	9.19	4.2867	4.228
ENSMUSG00000078866	-1.706421193	2.16E-25	1.37E-26	-1.779233022	5.02E-20	4.23E-21	22.9207	7.0233	6.6777
ENSMUSG00000078872	-5.005856425	2.59E-68	5.42E-70	-4.780896073	1.25E-71	2.73E-73	27.482	0.8553	0.9997
ENSMUSG00000078907	-9.154818109	0.000000214	4.49E-08	-5.569855608	0.0000136	0.00000345	0.57	0	0.012
ENSMUSG00000078920	7.492756535	4.72E-82	8.2E-84	7.696074059	2.03E-81	3.89E-83	0.1803	32.4803	37.396
ENSMUSG00000078921	7.604747153	4.08E-21	3.19E-22	7.838837464	4.56E-24	3.18E-25	0.0287	5.58	6.563
ENSMUSG00000079033	-4.178833992	0.00000287	0.000000683	-4.843895031	0.000000522	0.000000111	1.7327	0.0957	0.0603
ENSMUSG00000079083	-1.382500774	4.08E-10	6.59E-11	-1.304397036	1.95E-14	2.23E-15	7.1707	2.7503	2.9033
ENSMUSG00000079110	4.459502054	4.84E-26	3.02E-27	4.682809824	1.1E-32	5.6E-34	0.3103	6.8277	7.9707
ENSMUSG00000079162	-7.483815777	0.010657256	0.004330274	-7.483815777	0.008430881	0.003311959	0.179	0	0
ENSMUSG00000079184	1.817834215	3.05E-61	7.43E-63	1.836037874	1.25E-49	3.96E-51	5.4803	19.321	19.5663
ENSMUSG00000079197	1.092797122	3.01E-23	2.12E-24	1.052077546	8.76E-23	6.44E-24	20.8397	44.4483	43.2113
ENSMUSG00000079334	-1.179819855	1.24E-12	1.64E-13	-1.432645493	8.62E-14	1.03E-14	14.3987	6.3557	5.334
ENSMUSG00000079427	-1.715176033	0.000000348	7.49E-08	-1.597232287	0.00022503	0.0000673	8.6593	2.6373	2.862
ENSMUSG00000079442	1.052869901	5.25E-10	8.55E-11	1.23552935	1.48E-12	1.93E-13	3.264	6.7717	7.6857
ENSMUSG00000079451	-9.191470532	0.000000304	6.51E-08	-9.191470532	0.00000024	4.89E-08	0.5847	0	0
ENSMUSG00000079481	3.541953502	0.000109677	0.0000321	2.5730884	2.36E-10	3.7E-11	0.0813	0.9473	0.484
ENSMUSG00000079492	2.608080929	0.0000843	0.0000242	2.604839523	0.00000655	0.00000159	0.2923	1.7823	1.7783
ENSMUSG00000079563	4.71475239	1.99E-45	6.77E-47	4.478904477	1.34E-34	6.48E-36	0.3523	9.252	7.8567
ENSMUSG00000079625	2.521426844	0.000852091	0.000283892	1.946259801	0.019448028	0.008240941	0.196	1.1253	0.7553
ENSMUSG00000081534	-1.767129095	1.12E-94	1.58E-96	-1.964939646	1.74E-74	3.66E-76	84.0797	24.702	21.537
ENSMUSG00000084883	-1.277382755	4.87E-09	8.69E-10	-1.124564662	0.000000871	0.00000019	4.4327	1.8287	2.033
ENSMUSG00000085793	-1.02748888	1.35E-10	2.09E-11	-1.192082587	4.57E-14	5.36E-15	11.3563	5.571	4.9703
ENSMUSG00000087385	-2.771564256	0.0000409	0.0000112	-2.950270143	0.00000171	0.00000039	1.4067	0.206	0.182
ENSMUSG00000089665	3.392031145	0.013468654	0.005582077	3.309248961	0.017434702	0.0073102	0.16	1.6797	1.586
ENSMUSG00000089694	7.626925794	0.031893128	0.014352461	7.202940059	0.038931912	0.017691162	0	0.1977	0.1473
ENSMUSG00000089832	-1.130583048	2.18E-24	1.45E-25	-1.155747619	1.25E-21	9.65E-23	23.847	10.8917	10.7033
ENSMUSG00000089876	-1.320886297	3.79E-09	6.7E-10	-1.272719986	0.00000046	9.71E-08	7.383	2.9553	3.0557
ENSMUSG00000089901	-2.025909954	3.15E-13	3.98E-14	-1.543148705	4.59E-11	6.75E-12	4.3073	1.0577	1.478
ENSMUSG00000089929	-6.931592075	1.79E-58	4.59E-60	-9.681346731	1.38E-19	1.19E-20	39.9583	0.3273	0.0487
ENSMUSG00000089951	-4.058044795	0.049325071	0.023232214	-4.01622462	0.043829656	0.020226279	0.5663	0.034	0.035
ENSMUSG00000089953	-7.64625868	0.009689433	0.003911448	-7.64625868	0.00770788	0.002999283	0.2003	0	0
ENSMUSG00000090066	4.715893371	5.59E-13	7.2E-14	5.577730931	2.44E-19	2.12E-20	0.0167	0.438	0.796
ENSMUSG00000090164	-4.655917148	0.000721929	0.000237015	-9.41080465	0.0000105	0.00000262	0.6807	0.027	0
ENSMUSG00000090353	-1.358116085	0.000478154	0.000152896	-1.594014361	0.0000503	0.0000138	4.0503	1.58	1.3417
ENSMUSG00000090958	5.813781191	0.000536232	0.000172846	5.611024797	0.000724577	0.000233372	0.0053	0.3	0.2607
ENSMUSG00000091455	-6.315715658	6.53E-17	6.45E-18	-9.730753157	1.23E-13	1.48E-14	0.8497	0.0107	0
ENSMUSG00000091477	1.424582861	0.004239422	0.001593281	1.157341872	0.030114994	0.013323263	0.6653	1.786	1.484
ENSMUSG00000091512	1.117494203	4.19E-30	2.23E-31	1.09988956	2.83E-22	2.14E-23	25.182	54.6373	53.9747
ENSMUSG00000091649	-9.454642017	0.000174129	0.0000522	-9.454642017	0.000127919	0.0000369	0.7017	0	0
ENSMUSG00000091780	2.141054502	4.91E-30	2.62E-31	2.176819745	2.9E-32	1.49E-33	4.3837	19.3357	19.821
ENSMUSG00000091898	-3.589763487	0.04967155	0.023429651	-9.456012098	0.003261859	0.001184349	0.7023	0.0583	0
ENSMUSG00000091945	-1.178926797	0.0000176	0.00000458	-1.285491959	0.000000397	0.000000083	1.6747	0.7397	0.687
ENSMUSG00000092165	1.187956324	0.00000361	0.00000087	1.233141758	0.0000476	0.000013	3.0627	6.9777	7.1997
ENSMUSG00000092586	14.44236039	5.14E-11	7.73E-12	14.76992391	6.1E-19	5.42E-20	0	22.263	27.9377
ENSMUSG00000093769	-2.217838935	0.004423374	0.001667331	-1.426739904	0.029712041	0.013130408	0.949	0.204	0.353
ENSMUSG00000094030	-1.169154747	4.72E-15	5.24E-16	-1.0828552	5.65E-12	7.7E-13	23.2503	10.339	10.9763
ENSMUSG00000094081	-1.430101372	0.001406312	0.000485151	-1.502074679	0.001299454	0.00043665	0.9413	0.3493	0.3323
ENSMUSG00000094083	-10.87677272	0.00000148	0.000000341	-10.87677272	0.00000113	0.000000251	1.8803	0	0
ENSMUSG00000094151	5.508726915	0.000539571	0.00017396	6.214033354	0.0000144	0.00000366	0.0113	0.516	0.8413
ENSMUSG00000094347	2.114821442	1.6E-221	7.21E-224	1.935012342	4.37E-93	7.4E-95	44.856	194.2877	171.521
ENSMUSG00000095545	-1.301004042	3.02E-35	1.38E-36	-1.21539433	1.71E-25	1.1E-26	53.859	21.8583	23.1947
ENSMUSG00000095687	-1.123375055	5.05E-24	3.44E-25	-1.08580356	3.22E-25	2.12E-26	63.0003	28.9183	29.6813
ENSMUSG00000096054	-2.729026434	1.21E-14	1.39E-15	-2.855540645	3.43E-239	1.75E-241	30.6663	4.6253	4.237
ENSMUSG00000096370	-2.158972167	0.024380931	0.010694351	-2.225136209	0.042232938	0.019381455	2.69	0.6023	0.5753
ENSMUSG00000096463	-1.356395122	7.26E-10	1.2E-10	-1.223682368	2.06E-09	3.49E-10	17.5297	6.8463	7.506
ENSMUSG00000096472	2.449496245	1.6E-75	3.05E-77	2.374606826	2.44E-54	7.09E-56	5.559	30.3647	28.8287
ENSMUSG00000096740	1.004397123	2.24E-10	3.54E-11	1.088919687	6.16E-14	7.31E-15	4.095	8.215	8.7107
ENSMUSG00000097075	2.41966979	0.000807295	0.000267678	1.964909999	0.007103322	0.002747363	0.3433	1.837	1.3403
ENSMUSG00000097084	-7.448460501	0.0035331	0.001307946	-7.448460501	0.002727555	0.000976602	0.1747	0	0
ENSMUSG00000098112	2.615146413	3.61E-114	4.11E-116	2.702682217	1.55E-252	7.08E-255	37.3343	228.742	243.0507
ENSMUSG00000098923	1.155382294	7.08E-27	4.26E-28	1.347149362	2.21E-57	5.95E-59	14.051	31.2977	35.747
ENSMUSG00000099583	-2.010998999	0.033917808	0.015350783	-2.831292977	0.004302902	0.001598586	0.79	0.196	0.111
ENSMUSG00000099974	-6.002047829	0.000000478	0.000000104	-11.57695666	3.73E-09	6.45E-10	3.055	0.0477	0
ENSMUSG00000100838	-4.575705099	0.000340409	0.000106391	-10.82363261	0.00000427	0.00000102	1.8123	0.076	0
ENSMUSG00000102037	-8.195911265	3.08E-51	8.89E-53	-9.564489453	5.65E-28	3.34E-29	53.7597	0.1833	0.071
ENSMUSG00000102748	-1.185925725	1.9E-16	1.94E-17	-1.070126338	3.55E-11	5.15E-12	7.3137	3.2147	3.4833
ENSMUSG00000102752	1.877056216	0.00000213	0.000000502	2.263212417	6.24E-12	8.53E-13	0.3377	1.2403	1.621
ENSMUSG00000103144	-1.123072388	0.0000136	0.0000035	-1.066360519	0.000066	0.0000183	2.825	1.297	1.349
ENSMUSG00000104346	-1.972692654	0.020515682	0.008853311	-2.381731413	0.001178468	0.000393148	0.4187	0.1067	0.0803
ENSMUSG00000105867	1.843888025	0.000051	0.0000142	1.736257225	0.000144179	0.0000419	0.4753	1.7063	1.5837
ENSMUSG00000106631	-1.084199314	4.09E-18	3.78E-19	-1.040526762	2.5E-14	2.88E-15	31.48	14.8477	15.304
ENSMUSG00000107417	-9.756556323	0.0000279	0.00000748	-9.756556323	0.0000207	0.00000536	0.865	0	0
ENSMUSG00000107705	-2.479822332	8.59E-69	1.77E-70	-2.727855209	5.77E-08	1.11E-08	17.5047	3.138	2.6423
ENSMUSG00000108929	10.87190524	9.8E-13	1.28E-13	11.67050873	3.12E-15	3.41E-16	0	1.874	3.2597
ENSMUSG00000110206	2.639213694	6.93E-51	2.03E-52	2.734796377	1.45E-49	4.64E-51	4.1377	25.7773	27.543
ENSMUSG00000110277	-1.36351561	0.000126252	0.0000372	-1.531521934	0.000000822	0.000000179	6.6867	2.5987	2.313
ENSMUSG00000112023	1.768924521	2.69E-09	4.69E-10	2.498134221	4.06E-38	1.74E-39	4.1593	14.175	23.4983
ENSMUSG00000114133	1.203274567	7.53E-18	7.06E-19	1.14701133	1.44E-17	1.37E-18	5.4767	12.6107	12.1283
ENSMUSG00000114456	-3.197708158	0.00031862	0.0000991	-1.954126115	0.009977555	0.003964432	0.8563	0.0933	0.221
ENSMUSG00000114582	-9.226412193	0.0000273	0.00000731	-3.777951692	0.001585234	0.000542421	0.599	0	0.0437
ENSMUSG00000114635	10.76182822	1.45E-09	2.45E-10	10.57048823	3.77E-09	6.52E-10	0	1.7363	1.5207
ENSMUSG00000114694	-4.583166987	0.00253188	0.000913904	-2.712618239	0.036893241	0.016683977	0.5353	0.0223	0.0817
ENSMUSG00000115018	1.170438361	0.004652187	0.001760689	1.447959505	0.000129785	0.0000375	2.0813	4.6847	5.6783
ENSMUSG00000115483	1.004986873	1.59E-21	1.21E-22	1.018999139	1.95E-18	1.77E-19	16.269	32.6507	32.9693
ENSMUSG00000116564	1.066850196	3.67E-18	3.37E-19	1.112720325	1.06E-17	9.95E-19	11.0133	23.0713	23.8167
ENSMUSG00000117694	-1.10339125	0.027667477	0.012258643	-1.487161689	0.00502831	0.001889956	6.102	2.84	2.1767
ENSMUSG00000118607	-2.12346569	0.002408086	0.000867212	-6.951284715	0.000000984	0.000000217	0.825	0.1893	0.0067
ENSMUSG00000118661	-6.304650103	2.7E-175	1.78E-177	-5.92613848	4.41E-31	2.35E-32	5.5333	0.07	0.091
ENSMUSG00000118669	-1.893668765	0.000734541	0.000241615	-1.412587408	0.001607762	0.000550906	1.1767	0.3167	0.442
ENSMUSG00000118672	5.129283017	0.004386291	0.001651829	4.624490865	0.026585817	0.011629918	0	0.035	0.0247
