# Supplementary material for: Mutations of RAS genes identified in acute myeloid leukemia affect glycerophospholipid metabolism pathway
Source: Front Oncol. 2023 Nov 14;13:1280192. doi: 10.3389/fonc.2023.1280192 (PMC10682766; doi:10.3389/fonc.2023.1280192)
Supplement: Supplementary file 6 [file DataSheet_6.pdf]

Genes involved in metabolic process

ENSEMBL	exp_BaF3	exp_BaF3 KRAS(G12V)	exp_BaF3 NRAS(Q61K)
ENSMUSG00000000078	47.1253	15.346	16.1507
ENSMUSG00000000127	0.1677	0.0127	0.021
ENSMUSG00000000142	2.912	0.673	0.2483
ENSMUSG00000000182	0.0117	29.5267	35.7313
ENSMUSG00000000184	26.8673	129.5757	149.3187
ENSMUSG00000000244	2.242	72.866	72.5837
ENSMUSG00000000290	8.0627	78.144	81.195
ENSMUSG00000000308	0.0133	0.3307	0.355
ENSMUSG00000000320	0	0.2067	0.309
ENSMUSG00000000409	14.265	1.2133	0.97
ENSMUSG00000000552	11.6907	23.8503	29.1857
ENSMUSG00000000561	18.263	42.867	47.4693
ENSMUSG00000000562	0.7243	3.1797	5.8103
ENSMUSG00000000594	0.3197	0.0337	0.0867
ENSMUSG00000000686	13.622	1.579	1.339
ENSMUSG00000000706	0.3277	0.006	0.04
ENSMUSG00000000730	4.0953	0.8163	0.4067
ENSMUSG00000000782	0.267	0.0077	0
ENSMUSG00000000791	1.8503	7.2973	7.567
ENSMUSG00000000823	5.3373	22.7917	23.8467
ENSMUSG00000000869	6.52	0.032	0
ENSMUSG00000000915	20.3777	3.5903	3.4783
ENSMUSG00000001123	136.085	57.416	58.0093
ENSMUSG00000001211	5.18	86.7017	76.2133
ENSMUSG00000001228	73.2013	151.616	152.8077
ENSMUSG00000001366	21.5877	56.117	61.1787
ENSMUSG00000001441	47.576	20.957	19.8927
ENSMUSG00000001542	45.3973	16.3537	18.589
ENSMUSG00000001552	8.5547	0.959	1.0563
ENSMUSG00000001583	1.0733	0.42	0.4603

ENSMUSG00000001741	5.1743	12.1603	11.3227
ENSMUSG00000001750	9.233	21.8443	19.785
ENSMUSG00000001751	20.132	8.176	7.697
ENSMUSG00000001755	29.8187	10.0363	9.8757
ENSMUSG00000001761	2.2267	1.0043	1.1117
ENSMUSG00000001865	876.229	8.309	1.9893
ENSMUSG00000001911	10.7097	3.886	4.0347
ENSMUSG00000002058	17.6223	42.1043	41.1707
ENSMUSG00000002076	0	0.7443	0.3107
ENSMUSG00000002107	25.456	60.0153	60.0583
ENSMUSG00000002204	33.5543	9.1027	6.4303
ENSMUSG00000002324	0.5833	0	0
ENSMUSG00000002365	0.0427	8.407	9.8233
ENSMUSG00000002602	0.076	2.28	1.6637
ENSMUSG00000002603	69.9407	186.7587	241.4503
ENSMUSG00000002699	10.5953	153.9277	154.951
ENSMUSG00000002763	19.8913	8.6183	8.852
ENSMUSG00000002825	6.978	14.4627	17.0733
ENSMUSG00000002847	0.01	0.2623	0.3427
ENSMUSG00000002881	14.055	29.5443	31.2387
ENSMUSG00000002983	1.6417	3.4313	3.661
ENSMUSG00000002996	51.7053	21.5783	17.9607
ENSMUSG00000002997	31.7587	106.193	130.8683
ENSMUSG00000003051	1.7543	0.6437	0.595
ENSMUSG00000003123	6.1113	2.9987	2.7497
ENSMUSG00000003348	10.0657	29.8167	30.9293
ENSMUSG00000003363	23.238	6.4513	7.4033
ENSMUSG00000003458	43.287	20.5717	21.004
ENSMUSG00000003500	44.3473	14.7657	16.2493
ENSMUSG00000003526	12.2813	26.7887	26.447
ENSMUSG00000003762	5.3977	2.556	2.13
ENSMUSG00000003812	24.904	10.1393	8.0657

ENSMUSG00000003814	3052.664	330.2	307.322
ENSMUSG00000003849	6.8137	3.035	3.0377
ENSMUSG00000004044	9.0297	3.0887	2.506
ENSMUSG00000004319	16.193	0.389	1.086
ENSMUSG00000004328	1.9623	0.5647	0.4863
ENSMUSG00000004446	21.9607	45.856	50.3427
ENSMUSG00000004451	23.4963	60.8623	58.6623
ENSMUSG00000004530	23.5173	55.7937	57.9923
ENSMUSG00000004552	4.9547	0.5807	0.3953
ENSMUSG00000004667	34.665	79.7543	83.4317
ENSMUSG00000004788	52.7813	25.0183	24.672
ENSMUSG00000004864	8.7277	3.0227	2.2343
ENSMUSG00000004931	21.853	10.4307	10.2707
ENSMUSG00000005057	1.504	3.368	3.6367
ENSMUSG00000005107	3.3527	7.6263	7.347
ENSMUSG00000005339	616.7257	0.9263	0.551
ENSMUSG00000005609	39.4793	174.1703	151.9703
ENSMUSG00000005610	257.5393	592.7973	591.9293
ENSMUSG00000005682	9.4243	4.6563	4.391
ENSMUSG00000005686	2.1703	12.3773	9.9617
ENSMUSG00000005824	1.592	82.704	86.9357
ENSMUSG00000005893	21.4343	9.669	10.0663
ENSMUSG00000005917	4.2313	1.3507	1.4787
ENSMUSG00000005949	16.845	8.247	7.9053
ENSMUSG00000006262	11.118	31.0167	34.7203
ENSMUSG00000006344	0.005	1.464	1.3357
ENSMUSG00000006362	27.7473	76.3733	83.864
ENSMUSG00000006389	0.9	419.774	429.525
ENSMUSG00000006445	0.0103	0.5153	0.359
ENSMUSG00000006457	1.314	0.2043	0.2163
ENSMUSG00000006463	17.6803	5.6397	5.604
ENSMUSG00000006464	2.0473	0.692	0.675

ENSMUSG00000006494	2.3763	15.2287	13.0797
ENSMUSG00000006522	0	0.8517	0.5577
ENSMUSG00000006567	0.7017	0.1997	0.2047
ENSMUSG00000006587	0.2037	0.7157	0.9
ENSMUSG00000006732	6.4873	2.1403	2.6747
ENSMUSG00000007035	0.207	4.4213	6.5873
ENSMUSG00000007036	23.0647	50.8053	51.6353
ENSMUSG00000007216	1.4513	0.3597	0.3487
ENSMUSG00000007480	10.8703	0.7613	0.3043
ENSMUSG00000007613	13.9067	28.878	31.4777
ENSMUSG00000007659	4.664	96.6377	106.5137
ENSMUSG00000007682	1.896	0.041	0.0087
ENSMUSG00000008129	1.0507	0.1297	0.059
ENSMUSG00000008305	41.4873	16.2083	17.5537
ENSMUSG00000008393	20.7313	9.566	9.4177
ENSMUSG00000008450	10.9153	24.2143	25.1783
ENSMUSG00000008540	12.2447	0.1013	0.118
ENSMUSG00000009350	222.6543	6.52	0.4947
ENSMUSG00000009739	0.9577	0.124	0.1767
ENSMUSG00000009772	0.007	0.957	1.2017
ENSMUSG00000010154	12.7463	2.239	2.752
ENSMUSG00000010406	13.315	27.5697	31.571
ENSMUSG00000011148	18.9737	6.8637	4.9273
ENSMUSG00000012117	35.2937	15.7047	16.4267
ENSMUSG00000013089	2.207	36.7483	39.246
ENSMUSG00000013663	63.7347	30.091	27.1727
ENSMUSG00000014226	27.519	64.2713	60.3697
ENSMUSG00000014599	64.927	1.896	1.0903
ENSMUSG00000014606	46.905	186.4057	185.2197
ENSMUSG00000014905	77.6177	7.48	9.185
ENSMUSG00000015016	17.4197	4.6107	4.9947
ENSMUSG00000015437	94.951	2.2537	2.1597

ENSMUSG00000015533	0.422	0.18	0.163
ENSMUSG00000015599	2.959	1.413	1.365
ENSMUSG00000015714	52.8103	159.0037	155.603
ENSMUSG00000015837	410.896	91.1553	89.778
ENSMUSG00000015970	0.6873	0	0
ENSMUSG00000016206	11.1733	4.3963	4.4933
ENSMUSG00000016427	21.251	9.1377	7.0423
ENSMUSG00000016477	5.5227	14.7947	14.672
ENSMUSG00000016496	12.6077	30.9407	26.7737
ENSMUSG00000016526	8.792	3.47	2.9137
ENSMUSG00000016528	137.6013	61.403	62.5403
ENSMUSG00000016534	71.5443	32.761	29.5547
ENSMUSG00000016552	0.0793	0.3623	0.3197
ENSMUSG00000017195	0.274	0	0.016
ENSMUSG00000017561	30.343	68.2663	62.8597
ENSMUSG00000017718	5.0967	2.232	2.3627
ENSMUSG00000017724	0	0.6877	0.7413
ENSMUSG00000017737	0.0843	3.7403	2.393
ENSMUSG00000017776	16.8003	40.909	41.5523
ENSMUSG00000017897	0	1.125	0.7187
ENSMUSG00000017950	0.766	0.0787	0.0127
ENSMUSG00000018166	8.932	39.0163	40.1797
ENSMUSG00000018168	1.6167	0.046	0
ENSMUSG00000018199	2.5583	10.0197	10.0183
ENSMUSG00000018293	560.1133	1531.515	1497.0437
ENSMUSG00000018377	33.4107	83.2127	85.714
ENSMUSG00000018474	81.9467	28.4393	32.66
ENSMUSG00000018500	0.0227	2.7003	2.5677
ENSMUSG00000018654	2.6977	1.138	0.651
ENSMUSG00000018796	58.7147	10.2847	9.9263
ENSMUSG00000018841	22.404	9.4433	8.7847
ENSMUSG00000018909	4.955	22.0273	22.7047

ENSMUSG00000019102	0.9563	2.387	3.089
ENSMUSG00000019301	4.136	0.4143	0.505
ENSMUSG00000019528	81.353	36.416	30.647
ENSMUSG00000019842	5.3683	2.2553	1.7933
ENSMUSG00000019850	5.2937	1.369	1.398
ENSMUSG00000019916	16.7923	40.6607	34.803
ENSMUSG00000019947	3.517	0.2813	0.0767
ENSMUSG00000019960	7.791	81.0083	85.21
ENSMUSG00000019977	58.7247	26.7	26.7407
ENSMUSG00000019982	316.9017	16.552	9.737
ENSMUSG00000020009	219.3943	8.2393	7.1177
ENSMUSG00000020029	18.036	245.3767	270.97
ENSMUSG00000020048	831.2237	395.63	405.8777
ENSMUSG00000020057	0.7077	0.1853	0.12
ENSMUSG00000020077	928.756	184.8317	176.837
ENSMUSG00000020097	29.132	13.397	13.161
ENSMUSG00000020101	0.097	0.7307	0.6613
ENSMUSG00000020134	8.26	29.193	25.992
ENSMUSG00000020178	8.3707	23.379	28.0023
ENSMUSG00000020189	45.3757	21.376	16.6797
ENSMUSG00000020227	63.1273	22.934	21.054
ENSMUSG00000020256	1.165	0.2123	0.19
ENSMUSG00000020279	3.7003	1.4207	1.144
ENSMUSG00000020323	6.7607	0.0493	0.0867
ENSMUSG00000020383	0.3843	0	0
ENSMUSG00000020395	0.4157	0.072	0.0263
ENSMUSG00000020407	0.3287	4.744	5.1177
ENSMUSG00000020534	16.293	42.898	44.3047
ENSMUSG00000020576	19.4307	6.1297	5.8103
ENSMUSG00000020604	0.348	1.2513	1.473
ENSMUSG00000020620	0.0607	0	0.004
ENSMUSG00000020629	17.1613	6.1257	6.6927

ENSMUSG00000020644	1.8717	3.8393	4.0443
ENSMUSG00000020653	5.3753	1.257	1.347
ENSMUSG00000020659	21.3557	8.1817	9.1077
ENSMUSG00000020669	5.114	1.225	0.9793
ENSMUSG00000020689	1.6757	161.8493	189.4047
ENSMUSG00000020715	22.0573	9.7963	9.7163
ENSMUSG00000020733	82.21	39.1347	35.8733
ENSMUSG00000020743	17.0937	7.415	6.466
ENSMUSG00000020787	2.5147	38.5323	40.56
ENSMUSG00000020788	49.502	163.4477	177.6187
ENSMUSG00000020838	0.0927	7.337	11.865
ENSMUSG00000020877	6.9623	2.668	2.9657
ENSMUSG00000020901	1.5867	3.6943	4.0943
ENSMUSG00000020919	33.9677	15.526	13.913
ENSMUSG00000020964	58.5547	18.408	18.66
ENSMUSG00000020990	0.0593	1.2977	0.95
ENSMUSG00000021024	191.839	94.4277	94.2283
ENSMUSG00000021027	43.7933	13.261	13.5073
ENSMUSG00000021036	27.0133	12.706	12.0763
ENSMUSG00000021065	206.7893	34.653	39.1413
ENSMUSG00000021071	0.011	0.2963	0.199
ENSMUSG00000021108	1.6043	0.0053	0
ENSMUSG00000021109	138.423	31.8643	33.7673
ENSMUSG00000021120	14.11	6.3553	6.6503
ENSMUSG00000021123	4.1297	1.5793	1.8237
ENSMUSG00000021127	18.0027	5.812	6.523
ENSMUSG00000021182	22.4287	11.1443	9.5557
ENSMUSG00000021190	2.6137	60.536	76.849
ENSMUSG00000021226	13.6203	2.842	2.9933
ENSMUSG00000021236	23.289	7.3397	5.894
ENSMUSG00000021238	10.2987	5.0143	4.3883
ENSMUSG00000021240	7.5887	2.347	2.4727

ENSMUSG00000021250	1.2977	6.8983	18.9157
ENSMUSG00000021257	7.871	1.7303	1.8957
ENSMUSG00000021266	75.3003	33.9863	37.4067
ENSMUSG00000021322	0.2453	0.8893	0.8453
ENSMUSG00000021366	11.5583	5.748	5.3483
ENSMUSG00000021420	14.988	6.8467	7.3093
ENSMUSG00000021514	14.8693	6.585	6.368
ENSMUSG00000021549	11.4143	24.9863	38.3917
ENSMUSG00000021573	0.081	0.0037	0.004
ENSMUSG00000021608	9.6697	28.7937	28.2523
ENSMUSG00000021661	15.4267	6.2773	6.321
ENSMUSG00000021665	79.7153	21.634	21.022
ENSMUSG00000021733	5.7873	11.9983	14.4383
ENSMUSG00000021738	10.0507	4.81	4.9723
ENSMUSG00000021876	0.393	2.812	2.5963
ENSMUSG00000021884	13.495	5.5433	5.6763
ENSMUSG00000021900	13.88	6	5.807
ENSMUSG00000021948	21.44	61.3477	66.9017
ENSMUSG00000021951	8.3013	22.3417	24.669
ENSMUSG00000021958	4.262	8.685	9.0743
ENSMUSG00000021959	4.0643	1.1927	1.348
ENSMUSG00000021996	67.242	135.7127	146.8787
ENSMUSG00000022020	2.6827	8.787	7.6127
ENSMUSG00000022022	2.6273	7.2187	7.1217
ENSMUSG00000022037	34.267	182.9437	196.48
ENSMUSG00000022051	25.343	65.1237	60.2943
ENSMUSG00000022053	0	2.951	1.25
ENSMUSG00000022094	4.1727	15.7663	17.9243
ENSMUSG00000022157	776.0777	0.7297	0.505
ENSMUSG00000022215	0	0.7283	0.557
ENSMUSG00000022216	39.8627	80.6203	83.2457
ENSMUSG00000022219	0.0177	3.299	2.7113

ENSMUSG00000022221	0.6767	0.0613	0.0213
ENSMUSG00000022225	898.3517	4.9623	3.9283
ENSMUSG00000022244	3.2307	1.2293	1.4547
ENSMUSG00000022253	57.6127	18.7593	17.7243
ENSMUSG00000022255	63.2417	197.7653	213.221
ENSMUSG00000022307	27.2153	69.3137	62.9763
ENSMUSG00000022309	64.978	172.9983	162.883
ENSMUSG00000022359	12.0917	4.9313	4.9093
ENSMUSG00000022394	35.6437	14.6657	15.403
ENSMUSG00000022442	16.218	8.0317	7.1803
ENSMUSG00000022453	0.1973	1.5187	1.5007
ENSMUSG00000022475	57.8793	6.0153	4.0913
ENSMUSG00000022623	0.0457	16.8287	12.5787
ENSMUSG00000022637	44.2437	13.3183	13.327
ENSMUSG00000022742	37.8133	15.352	15.412
ENSMUSG00000022769	105.8837	30.431	29.5657
ENSMUSG00000022780	0.3333	0.0627	0.0717
ENSMUSG00000022791	15.899	54.938	55.217
ENSMUSG00000022836	0.046	5.7677	5.3207
ENSMUSG00000022861	9.756	4.868	4.2427
ENSMUSG00000022885	0.6033	0.01	0.0173
ENSMUSG00000022895	11.9823	25.053	27.8863
ENSMUSG00000022951	15.2467	6.362	5.637
ENSMUSG00000022952	104.8183	18.0923	17.3257
ENSMUSG00000023031	3.055	6.988	10.555
ENSMUSG00000023045	14.6557	3.63	3.6727
ENSMUSG00000023087	422.473	1017.296	936.059
ENSMUSG00000023333	0	0.5593	0.4527
ENSMUSG00000023411	1.9593	0.1187	0.044
ENSMUSG00000023805	3.059	6.869	7.5987
ENSMUSG00000023908	20.9037	9.0847	10.1287
ENSMUSG00000023952	51.8927	19.1643	19.7937

ENSMUSG00000023972	0.285	0	0.0273
ENSMUSG00000024006	47.5953	22.8743	20.1287
ENSMUSG00000024034	0	0.488	0.5213
ENSMUSG00000024042	0.932	15.7523	13.263
ENSMUSG00000024063	70.5203	16.6447	15.9287
ENSMUSG00000024070	21.2287	0.2957	0.585
ENSMUSG00000024114	3.7273	0.9927	1.036
ENSMUSG00000024127	27.3797	13.0627	12.524
ENSMUSG00000024171	3.7203	0.043	0
ENSMUSG00000024206	9.7317	2.6567	2.5933
ENSMUSG00000024220	16.226	5.25	5.4113
ENSMUSG00000024238	9.3647	20.9793	22.339
ENSMUSG00000024268	1.8233	0.905	0.883
ENSMUSG00000024308	40.569	15.6627	16.4937
ENSMUSG00000024371	0.6417	1.8317	1.922
ENSMUSG00000024381	3.8353	15.2283	14.4983
ENSMUSG00000024399	3.7267	30.8867	28.8527
ENSMUSG00000024401	0.3157	6.932	11.6
ENSMUSG00000024402	0.029	0.8073	0.7517
ENSMUSG00000024427	0.0653	1.1093	1.0833
ENSMUSG00000024457	12.0013	5.6583	5.2107
ENSMUSG00000024579	21.483	10.4833	10.101
ENSMUSG00000024589	15.3217	38.4807	47.527
ENSMUSG00000024594	50.7647	12.854	11.9347
ENSMUSG00000024614	32.8997	14.168	16.4343
ENSMUSG00000024642	40.1313	18.7737	18.4877
ENSMUSG00000024644	36.077	7.836	10.3537
ENSMUSG00000024646	40.9877	14.95	18.3003
ENSMUSG00000024659	1000.327	5.8763	4.6423
ENSMUSG00000024691	94.274	200.9873	200.477
ENSMUSG00000024747	0.1693	0	0
ENSMUSG00000024781	29.8383	14.7697	12.642

ENSMUSG00000024807	81.8287	19.2377	22.4277
ENSMUSG00000024827	0.3453	0.0163	0.0627
ENSMUSG00000024885	0.084	1.6303	1.168
ENSMUSG00000024887	1.6633	7.5907	7.796
ENSMUSG00000024899	17.6593	0.078	0.0533
ENSMUSG00000024900	41.0053	13.3977	14.5287
ENSMUSG00000024905	0.893	0.307	0.2917
ENSMUSG00000024912	0.648	2.0753	2.5303
ENSMUSG00000024921	57.7407	26.5063	27.5957
ENSMUSG00000024935	0.102	0.005	0
ENSMUSG00000024968	8.3603	23.8093	23.0147
ENSMUSG00000024975	41.651	14.3417	15.4583
ENSMUSG00000025010	1.7543	3.9167	4.737
ENSMUSG00000025068	223.21	106.528	102.988
ENSMUSG00000025197	8.3097	2.1763	1.8163
ENSMUSG00000025260	44.8747	95.6017	94.223
ENSMUSG00000025283	71.308	29.036	26.6687
ENSMUSG00000025351	382.136	126.691	123.2727
ENSMUSG00000025355	1.513	0.4863	0.2023
ENSMUSG00000025407	0.7477	0.1817	0.137
ENSMUSG00000025408	52.203	3.813	4.7703
ENSMUSG00000025421	12.239	25.4723	27.372
ENSMUSG00000025425	0.1957	0.0093	0.0107
ENSMUSG00000025432	22.4483	2.8803	1.7003
ENSMUSG00000025477	5.818	18.547	19.8
ENSMUSG00000025494	3.7597	11.1753	11.7337
ENSMUSG00000025571	23.6843	9.4473	10.3037
ENSMUSG00000025574	32.585	71.8663	68.5277
ENSMUSG00000025579	89.2927	24.061	25.3663
ENSMUSG00000025701	0.2457	1.143	1.2303
ENSMUSG00000025746	14.7677	0.718	0.057
ENSMUSG00000025809	78.7293	160.0777	164.2873

ENSMUSG00000025888	5.233	2.542	2.3727
ENSMUSG00000025921	3.4903	10.5547	10.0163
ENSMUSG00000025934	0.369	0.0143	0.0147
ENSMUSG00000025939	38.59	12.9217	11.3767
ENSMUSG00000025981	14.8343	7.3847	7.3623
ENSMUSG00000025986	5.198	17.0047	17.5483
ENSMUSG00000025997	101.5637	4.937	2.5163
ENSMUSG00000026003	90.5353	27.592	28.7897
ENSMUSG00000026017	5.608	1.8343	1.4147
ENSMUSG00000026024	18.2977	5.5377	5.947
ENSMUSG00000026094	24.447	79.1677	65.3323
ENSMUSG00000026107	26.0467	9.8223	7.2483
ENSMUSG00000026180	2.3517	11.2257	11.326
ENSMUSG00000026204	0.1333	0	0
ENSMUSG00000026209	86.7073	35.8903	38.5093
ENSMUSG00000026223	50.4797	21.6547	20.574
ENSMUSG00000026307	32.6447	14.94	15.3993
ENSMUSG00000026360	11.128	4.4533	4.2687
ENSMUSG00000026411	37.788	11.7693	12.718
ENSMUSG00000026435	0.7533	0.0233	0.0393
ENSMUSG00000026447	1.666	4.0463	4.041
ENSMUSG00000026456	61.987	21.5003	20.1837
ENSMUSG00000026483	51.9413	8.0993	7.5837
ENSMUSG00000026484	72.4363	25.67	24.5503
ENSMUSG00000026509	31.6757	78.765	86.3357
ENSMUSG00000026544	4.459	0.7893	0.7313
ENSMUSG00000026564	0.3323	0.024	0.0997
ENSMUSG00000026572	0	0.636	0.5833
ENSMUSG00000026576	0.2943	0.03	0.0663
ENSMUSG00000026579	0.48	19.3847	23.8363
ENSMUSG00000026582	0.042	93.1897	116.2293
ENSMUSG00000026604	1.833	0.5433	0.585

ENSMUSG00000026638	0.34	0.0697	0.0187
ENSMUSG00000026672	18.3273	8.86	8.3193
ENSMUSG00000026715	0.01	0.536	0.396
ENSMUSG00000026728	183.41	54.7057	30.264
ENSMUSG00000026749	4.1057	26.7807	26.9887
ENSMUSG00000026764	0.2683	0.0347	0.0207
ENSMUSG00000026784	3.908	13.0367	12.5747
ENSMUSG00000026805	0	0.1073	0.0983
ENSMUSG00000026812	5.4183	12.38	11.647
ENSMUSG00000026815	30.5383	63.052	61.702
ENSMUSG00000026829	0	0.725	0.3057
ENSMUSG00000026836	0.6493	0	0.0083
ENSMUSG00000026837	12.2463	0.6443	0.341
ENSMUSG00000026843	18.5133	38.0927	39.7363
ENSMUSG00000026854	9.3453	22.5167	22.218
ENSMUSG00000026864	1782.343	396.569	426.741
ENSMUSG00000026879	10.7477	28.1197	22.0263
ENSMUSG00000026923	2.14	0.7187	0.8557
ENSMUSG00000026944	76.1403	12.0133	12.7253
ENSMUSG00000026970	21.827	49.0693	55.694
ENSMUSG00000026988	3.609	11.0027	10.708
ENSMUSG00000027072	3.5403	0.2463	0.021
ENSMUSG00000027073	302.7497	14.837	3.5933
ENSMUSG00000027078	2.5887	12.1403	13.9413
ENSMUSG00000027111	1.4773	5.7907	8.811
ENSMUSG00000027195	25.7163	86.564	85.696
ENSMUSG00000027201	22.49	51.5907	53.6543
ENSMUSG00000027204	0.0083	1.5537	2.3927
ENSMUSG00000027210	2.2757	5.061	5.2787
ENSMUSG00000027233	0.716	0	0.0327
ENSMUSG00000027239	0.058	2.042	1.7137
ENSMUSG00000027296	0.1127	0.541	0.5257

ENSMUSG00000027314	0.0983	0.359	0.565
ENSMUSG00000027315	0.0983	1.7437	1.3947
ENSMUSG00000027351	5.2887	65.6417	74.4257
ENSMUSG00000027360	114.2503	3.275	0.518
ENSMUSG00000027366	37.893	17.8903	17.232
ENSMUSG00000027368	1.28	4.6237	8.2767
ENSMUSG00000027381	14.563	7.12	6.6637
ENSMUSG00000027387	1.795	3.7057	4.196
ENSMUSG00000027438	1.7513	0.869	0.6197
ENSMUSG00000027489	0.5543	1.209	1.342
ENSMUSG00000027636	1.603	80.796	74.9267
ENSMUSG00000027639	14.391	43.4433	43.175
ENSMUSG00000027646	0.0423	0.25	0.4017
ENSMUSG00000027737	3.8607	0.9707	0.5587
ENSMUSG00000027763	143.0217	330.372	353.0567
ENSMUSG00000027765	5.311	177.172	187.55
ENSMUSG00000027803	0	0.0777	0.6377
ENSMUSG00000027834	22.2733	0.6003	0.5083
ENSMUSG00000027843	10.441	2.065	1.5997
ENSMUSG00000027931	3.125	1.5537	1.4393
ENSMUSG00000027950	0.814	1.7243	1.6827
ENSMUSG00000027962	0.2313	0.022	0.024
ENSMUSG00000027963	4.7807	10.4397	9.7273
ENSMUSG00000027981	9.5847	20.0503	20.5927
ENSMUSG00000028063	10.8597	36.1603	40.6143
ENSMUSG00000028086	4.5663	11.251	12.9033
ENSMUSG00000028088	0.2007	0.5677	0.5313
ENSMUSG00000028100	1.543	0.2943	0.437
ENSMUSG00000028121	0.724	0	0.0707
ENSMUSG00000028124	107.4813	42.7153	52.5407
ENSMUSG00000028175	16.9637	35.3387	34.2003
ENSMUSG00000028211	14.5267	6.1883	4.2483

ENSMUSG00000028212	6.216	14.4687	15.1747
ENSMUSG00000028223	55.8233	19.1237	18.701
ENSMUSG00000028266	196.547	19.1833	20.7963
ENSMUSG00000028300	0.566	2.486	2.6437
ENSMUSG00000028410	72.537	161.109	149.9027
ENSMUSG00000028420	7.873	1.7713	1.6383
ENSMUSG00000028476	3.875	0.6457	0.6163
ENSMUSG00000028521	17.7597	4.6797	4.59
ENSMUSG00000028528	67.208	18.0803	17.9603
ENSMUSG00000028542	15.0883	3.541	3.6803
ENSMUSG00000028552	108.8133	52.29	48.8713
ENSMUSG00000028558	0.332	0.0353	0
ENSMUSG00000028576	6.397	3.0373	2.912
ENSMUSG00000028602	0.031	0.3173	0.275
ENSMUSG00000028613	6.8257	15.8617	17.708
ENSMUSG00000028641	16.842	7.4433	8.0203
ENSMUSG00000028669	62.0283	16.6623	20.7377
ENSMUSG00000028680	16.98	4.1597	6.3073
ENSMUSG00000028687	7.2283	3.5477	3.523
ENSMUSG00000028756	13.7927	4.2387	4.394
ENSMUSG00000028757	165.6353	65.9243	64.691
ENSMUSG00000028771	26.558	95.8247	103.3987
ENSMUSG00000028779	39.6757	17.4893	16.2683
ENSMUSG00000028793	7.849	16.4553	23.216
ENSMUSG00000028799	28.4613	11.078	12.32
ENSMUSG00000028807	1.0287	0.009	0
ENSMUSG00000028838	5.1363	0.285	0.2377
ENSMUSG00000028862	1.3883	12.63	11.1723
ENSMUSG00000028864	0.1403	0	0.009
ENSMUSG00000028885	1.4197	4.579	4.8387
ENSMUSG00000028893	42.355	20.6477	20.8327
ENSMUSG00000028977	1.7067	0.2207	0.3167

ENSMUSG00000029004	32.959	13.4513	13.8153
ENSMUSG00000029060	16.7493	4.7643	4.5013
ENSMUSG00000029096	0.0427	1.1303	1.3697
ENSMUSG00000029162	16.3163	41.2393	42.7513
ENSMUSG00000029165	12.429	5.086	5.661
ENSMUSG00000029171	20.7947	46.6987	47.311
ENSMUSG00000029201	61.8877	17.189	15.6637
ENSMUSG00000029217	32.067	11.9317	10.3717
ENSMUSG00000029254	2.8183	0.7597	0.9613
ENSMUSG00000029263	4.394	12.577	12.6617
ENSMUSG00000029265	37.265	121.974	147.1697
ENSMUSG00000029311	48.645	23.1167	19.581
ENSMUSG00000029312	1.8287	0.871	0.6147
ENSMUSG00000029313	60.5597	15.7423	15.1497
ENSMUSG00000029314	1.0703	0.2373	0.218
ENSMUSG00000029322	496.4317	45.6117	18.2777
ENSMUSG00000029344	8.8887	119.114	136.548
ENSMUSG00000029359	1.1707	0.0973	0
ENSMUSG00000029373	0.1523	4.6043	2.8107
ENSMUSG00000029403	2.6017	1.111	1.1203
ENSMUSG00000029456	6.0767	1.2483	1.4277
ENSMUSG00000029471	9.6717	25.0767	27.386
ENSMUSG00000029478	18.3823	48.7547	52.0793
ENSMUSG00000029484	71.441	30.5647	18.519
ENSMUSG00000029512	11.292	3.496	3.954
ENSMUSG00000029516	29.718	13.6033	12.0727
ENSMUSG00000029553	0	1.4807	1.8153
ENSMUSG00000029561	0.2763	6.7597	9.1003
ENSMUSG00000029580	919.7383	2976.486	2659.2857
ENSMUSG00000029586	0	0.6143	0.2117
ENSMUSG00000029718	3.4023	0.349	0.0247
ENSMUSG00000029725	14.881	6.4193	7.094

ENSMUSG00000029752	275.9253	109.4563	102.002
ENSMUSG00000029762	10.1653	22.5943	24.0577
ENSMUSG00000029811	0.9747	0	0
ENSMUSG00000029925	2.1263	0.0613	0.7993
ENSMUSG00000030032	0.1633	0.9647	0.8387
ENSMUSG00000030041	0.723	5.3717	3.463
ENSMUSG00000030054	0.3763	25.6633	30.3247
ENSMUSG00000030060	24.4843	9.279	9.3487
ENSMUSG00000030103	16.0807	4.7833	4.087
ENSMUSG00000030104	73.333	21.8157	23.8193
ENSMUSG00000030107	0.1283	10.939	9.418
ENSMUSG00000030124	5.917	1.14	1.126
ENSMUSG00000030142	42.7243	3.6597	2.5647
ENSMUSG00000030162	48.613	0.989	0.142
ENSMUSG00000030203	8.735	2.3987	3.209
ENSMUSG00000030228	124.9217	3.152	1.3843
ENSMUSG00000030281	4.13	13.8793	12.6367
ENSMUSG00000030303	15.878	1.0963	0.994
ENSMUSG00000030309	2.921	6.0877	6.7397
ENSMUSG00000030341	6.1517	15.7197	15.3517
ENSMUSG00000030350	0.0147	3.6353	3.433
ENSMUSG00000030365	37.1007	8.6603	6.9037
ENSMUSG00000030413	12.244	2.1637	1.2127
ENSMUSG00000030433	3.1597	1.037	1.1147
ENSMUSG00000030468	0.8123	0	0.022
ENSMUSG00000030470	1.628	0.1443	0.063
ENSMUSG00000030546	1.1407	0.1747	0.3803
ENSMUSG00000030557	21.4667	59.267	64.3203
ENSMUSG00000030559	43.2567	11.828	10.4713
ENSMUSG00000030657	11.286	1.1997	0.7073
ENSMUSG00000030659	0.2043	0.9877	1.535
ENSMUSG00000030671	26.759	10.4333	11.2133

ENSMUSG00000030674	0.9527	0.2053	0.036
ENSMUSG00000030717	2.8177	0.036	0
ENSMUSG00000030742	0.0997	80.1557	92.311
ENSMUSG00000030748	2.8413	8.2857	7.4937
ENSMUSG00000030782	0.1387	0.4527	0.607
ENSMUSG00000030785	17.5927	0.062	0.029
ENSMUSG00000030787	0.0143	1.7277	1.2607
ENSMUSG00000030788	11.1947	49.133	40.0647
ENSMUSG00000030851	5.3443	0.364	0.4217
ENSMUSG00000030879	23.255	51.467	47.0703
ENSMUSG00000030921	5.7987	16.6997	15.735
ENSMUSG00000030946	9.052	19.2383	18.3907
ENSMUSG00000031007	103.6227	38.629	32.9943
ENSMUSG00000031012	23.8623	3.7853	3.2983
ENSMUSG00000031026	0.0253	2.5077	2.074
ENSMUSG00000031066	35.0447	12.8993	13.219
ENSMUSG00000031078	0.0487	0.4077	0.3227
ENSMUSG00000031103	16.1377	6.214	6.5817
ENSMUSG00000031162	61.649	13.9223	18.5567
ENSMUSG00000031309	68.9007	19.725	17.973
ENSMUSG00000031328	77.6887	273.8553	252.181
ENSMUSG00000031445	0.4137	1.0483	1.1913
ENSMUSG00000031467	8.4887	19.2933	19.807
ENSMUSG00000031503	0.494	0.032	0.0637
ENSMUSG00000031530	1.0623	79.002	89.3817
ENSMUSG00000031555	6.9237	21.7497	20.5417
ENSMUSG00000031562	4.347	11.567	13.8263
ENSMUSG00000031570	37.298	16.5183	17.137
ENSMUSG00000031586	41.4917	11.6197	10.402
ENSMUSG00000031616	0.131	0	0.006
ENSMUSG00000031628	341.411	96.117	86.8143
ENSMUSG00000031639	0.005	0.3857	0.333

ENSMUSG00000031698	0.3667	9.891	14.8807
ENSMUSG00000031712	10.7493	0.2837	0.2577
ENSMUSG00000031714	3.9167	0.0783	0.0667
ENSMUSG00000031749	2.864	12.4283	11.663
ENSMUSG00000031750	0	0.7983	1.6917
ENSMUSG00000031751	85.8787	23.6587	29.205
ENSMUSG00000031760	3.4497	0.262	0.2217
ENSMUSG00000031767	14.9733	5.1047	5.58
ENSMUSG00000031770	182.884	47.315	53.6153
ENSMUSG00000031778	0.291	1.149	1.8207
ENSMUSG00000031785	80.3167	237.7823	286.737
ENSMUSG00000031860	3.906	0.1327	0.2013
ENSMUSG00000031877	183.3753	34.1143	38.7277
ENSMUSG00000031901	9.0597	3.5647	3.914
ENSMUSG00000031925	16.271	3.8397	4.5257
ENSMUSG00000031967	41.4627	17.9003	19.1483
ENSMUSG00000031970	4.334	1.8713	1.7403
ENSMUSG00000031974	27.8533	11.8867	10.5107
ENSMUSG00000031995	2.2523	0.8397	0.4827
ENSMUSG00000031997	15.018	1.042	0.566
ENSMUSG00000032009	3.341	0.645	0.719
ENSMUSG00000032020	7.0747	54.4357	61.2923
ENSMUSG00000032035	20.3077	0.0413	0.0077
ENSMUSG00000032038	18.8327	48.5623	52.6457
ENSMUSG00000032047	27.4307	66.8147	65.396
ENSMUSG00000032051	31.557	11.047	10.7427
ENSMUSG00000032101	1.447	3.3947	3.5893
ENSMUSG00000032109	3.164	6.3737	6.8517
ENSMUSG00000032177	5.2703	15.5213	19.1813
ENSMUSG00000032265	52.607	1.2513	1.0957
ENSMUSG00000032297	2.4107	0.981	1.179
ENSMUSG00000032298	6.5923	2.3973	2.8257

ENSMUSG00000032323	281.6317	24.3077	28.458
ENSMUSG00000032348	21.6767	1.704	1.3503
ENSMUSG00000032380	1.8247	0.331	0.336
ENSMUSG00000032402	1.881	15.8133	17.3957
ENSMUSG00000032418	15.114	3.869	3.215
ENSMUSG00000032420	0.233	0.0207	0
ENSMUSG00000032425	16.6637	6.386	6.088
ENSMUSG00000032456	2.5823	5.3803	5.184
ENSMUSG00000032462	16.8257	40.058	42.4517
ENSMUSG00000032468	26.1563	10.986	11.1733
ENSMUSG00000032492	1.2487	0.4867	0.5517
ENSMUSG00000032508	14.1377	34.826	34.427
ENSMUSG00000032554	90.5433	21.365	17.405
ENSMUSG00000032584	1.7297	0.108	0.082
ENSMUSG00000032596	1.7203	14.2773	15.5663
ENSMUSG00000032607	1.2853	0.3613	0.2743
ENSMUSG00000032643	6.48	41.2023	43.3973
ENSMUSG00000032661	0.289	4.4937	4.956
ENSMUSG00000032690	0	2.8547	2.9367
ENSMUSG00000032698	12.127	120.2003	123.4863
ENSMUSG00000032715	28.382	5.1677	5.8053
ENSMUSG00000032741	11.3933	28.467	31.4933
ENSMUSG00000032850	0.1673	0.0087	0.0277
ENSMUSG00000032855	6.4717	3.2163	3.1977
ENSMUSG00000032902	14.4307	37.0663	37.6113
ENSMUSG00000032905	52.355	9.3737	8.5947
ENSMUSG00000032913	4.3	12.5417	13.4227
ENSMUSG00000032965	20.895	6.089	4.999
ENSMUSG00000032966	56.8353	178.5223	183.2133
ENSMUSG00000033022	2.2503	0.5067	0.4847
ENSMUSG00000033107	53.243	19.2147	16.4827
ENSMUSG00000033191	6.222	65.344	66.9787

ENSMUSG00000033233	14.4757	2.2973	1.4947
ENSMUSG00000033276	0.6237	0.2207	0.1703
ENSMUSG00000033318	3.272	0.9717	0.784
ENSMUSG00000033326	50.0983	18.538	18.4187
ENSMUSG00000033327	0.9577	0.3123	0.336
ENSMUSG00000033545	31.6557	8.411	7.931
ENSMUSG00000033557	24.1333	7.9817	8.64
ENSMUSG00000033581	4.047	1.603	1.0407
ENSMUSG00000033623	2.789	6.218	6.1923
ENSMUSG00000033730	0.005	2.1127	2.8363
ENSMUSG00000033857	6.0007	2.121	2.1633
ENSMUSG00000033862	24.9957	10.4227	9.9323
ENSMUSG00000033955	1.5097	0.5997	0.657
ENSMUSG00000033967	1.0007	2.0067	2.2253
ENSMUSG00000033985	5.0947	2.1603	1.8657
ENSMUSG00000034041	10.5753	111.3547	127.4307
ENSMUSG00000034101	16.1887	34.6397	34.8873
ENSMUSG00000034165	39.4473	276.468	296.3067
ENSMUSG00000034175	13.3883	6.003	6.295
ENSMUSG00000034177	3.2523	0.7957	0.4017
ENSMUSG00000034187	30.9507	93.8963	95.3167
ENSMUSG00000034235	1.278	2.558	2.6393
ENSMUSG00000034353	22.1217	2.9033	0.9387
ENSMUSG00000034371	17.3937	6.8353	8.1473
ENSMUSG00000034430	8.1623	3.2517	4.081
ENSMUSG00000034570	5.7397	2.3337	2.169
ENSMUSG00000034579	1.1027	0.3457	0.2093
ENSMUSG00000034613	7.6073	2.792	2.864
ENSMUSG00000034614	5.628	1.601	1.8273
ENSMUSG00000034663	11.8483	71.436	84.9893
ENSMUSG00000034738	1.818	7.966	7.529
ENSMUSG00000034744	28.1813	13.85	12.1757

ENSMUSG00000034758	1.4287	6.3103	5.7527
ENSMUSG00000034765	3.629	8.6683	10.892
ENSMUSG00000034771	0.8777	1.9003	2.129
ENSMUSG00000034793	36.7033	10.817	11.5743
ENSMUSG00000034818	0.2937	0.0817	0.0363
ENSMUSG00000035049	13.13	27.3643	27.713
ENSMUSG00000035064	57.1873	6.0207	4.301
ENSMUSG00000035158	3.5807	10.925	10.3467
ENSMUSG00000035183	4.1627	72.584	94.2233
ENSMUSG00000035311	27.498	11.5223	11.393
ENSMUSG00000035329	23.697	7.239	7.6717
ENSMUSG00000035547	0.1833	3.5397	2.3627
ENSMUSG00000035678	5.7717	12.4657	14.105
ENSMUSG00000035713	0.961	0.447	0.4313
ENSMUSG00000035783	5.1763	46.049	23.3573
ENSMUSG00000035845	20.398	7.622	7.5167
ENSMUSG00000035868	2.427	0.968	0.8103
ENSMUSG00000035967	85.8973	30.941	35.118
ENSMUSG00000036052	6.645	1.0397	0.6997
ENSMUSG00000036067	2.713	0.4727	0.0723
ENSMUSG00000036091	2.149	0.9387	0.5423
ENSMUSG00000036109	14.0003	6.679	6.1697
ENSMUSG00000036158	4.7253	0.571	0.5923
ENSMUSG00000036181	31.5533	8.851	11.923
ENSMUSG00000036257	73.4853	16.48	17.9753
ENSMUSG00000036295	12.521	0.126	0.0253
ENSMUSG00000036390	89.9913	5.9387	5.8567
ENSMUSG00000036461	48.4607	155.364	149.0897
ENSMUSG00000036478	12.1507	3.6827	3.9017
ENSMUSG00000036537	4.5797	1.2543	1.6433
ENSMUSG00000036585	0.0327	0.2583	0.284
ENSMUSG00000036587	23.2503	2.2147	0.853

ENSMUSG00000036599	26.6653	8.5023	9.6287
ENSMUSG00000036622	7.4803	25.8677	28.863
ENSMUSG00000036743	6.799	17.467	16.7057
ENSMUSG00000036820	13.613	6.2447	4.7663
ENSMUSG00000036931	0.8817	3.8053	3.8563
ENSMUSG00000036986	11.0243	29.535	25.035
ENSMUSG00000037003	0.2907	1.855	2.7577
ENSMUSG00000037103	26.8077	11.771	13.1113
ENSMUSG00000037108	14.6877	4.7183	4.7097
ENSMUSG00000037169	1.082	6.7787	9.8437
ENSMUSG00000037410	15.4373	7.668	6.443
ENSMUSG00000037447	14.1317	4.167	5.2423
ENSMUSG00000037463	0.9477	0.2683	0.1957
ENSMUSG00000037470	62.823	27.7317	29.8307
ENSMUSG00000037572	12.8103	27.196	27.0213
ENSMUSG00000037580	5.2493	91.6687	91.305
ENSMUSG00000037628	5.0063	15.177	13.998
ENSMUSG00000037706	3.1513	15.0717	12.5293
ENSMUSG00000037722	9.867	25.4397	24.8053
ENSMUSG00000037725	15.9263	33.0607	33.412
ENSMUSG00000037754	0.485	3.4043	4.066
ENSMUSG00000037820	1.2717	72.4303	64.2993
ENSMUSG00000037824	7.076	33.1237	34.3603
ENSMUSG00000037868	0.1547	1.9367	5.32
ENSMUSG00000037922	9.8097	27.7117	25.01
ENSMUSG00000037940	31.5173	2.853	3.662
ENSMUSG00000038025	11.7763	5.4807	5.7767
ENSMUSG00000038028	2.39	5.509	5.1893
ENSMUSG00000038037	0.132	0.9873	1.0243
ENSMUSG00000038065	2.8223	1.3533	1.1343
ENSMUSG00000038068	1.86	0.0253	0.0267
ENSMUSG00000038172	3.7817	19.556	22.9267

ENSMUSG00000038205	0.9863	4.337	4.4913
ENSMUSG00000038235	6.8187	14.044	13.8117
ENSMUSG00000038290	23.924	9.822	8.824
ENSMUSG00000038295	0.5477	1.253	1.335
ENSMUSG00000038312	47.1843	20.5913	22.6933
ENSMUSG00000038349	0.153	0	0
ENSMUSG00000038417	3.1707	1.3527	1.1107
ENSMUSG00000038563	8.664	29.486	30.2907
ENSMUSG00000038615	60.13	23.9473	27.7137
ENSMUSG00000038712	15.221	50.419	63.4673
ENSMUSG00000038732	29.1497	11.7717	10.297
ENSMUSG00000038763	5.5857	2.5447	2.3697
ENSMUSG00000038770	0.4623	1.217	1.0503
ENSMUSG00000038797	4.2827	2.0703	1.7443
ENSMUSG00000038843	19.4737	44.6283	41.6363
ENSMUSG00000038910	26.478	9.234	9.106
ENSMUSG00000038936	21.3257	10.298	9.6603
ENSMUSG00000038954	15.9477	5.7407	7.5983
ENSMUSG00000038963	2.192	117.465	125.013
ENSMUSG00000039005	1.5747	8.1857	9.025
ENSMUSG00000039063	0.7197	2.6303	3.306
ENSMUSG00000039081	4.6147	1.5557	1.8583
ENSMUSG00000039109	6.2873	268.7407	284.1973
ENSMUSG00000039153	6.1787	1.7523	1.5843
ENSMUSG00000039158	6.5673	2.6627	2.8697
ENSMUSG00000039191	24.6333	73.97	86.537
ENSMUSG00000039199	2.1493	0.974	0.9423
ENSMUSG00000039242	74.354	19.3223	17.2963
ENSMUSG00000039308	57.503	10.5927	11.3873
ENSMUSG00000039354	26.1153	12.4717	11.7147
ENSMUSG00000039384	1.7263	14.4103	17.1433
ENSMUSG00000039431	0.7547	4.791	4.225

ENSMUSG00000039474	3.378	0.7083	1.1003
ENSMUSG00000039616	5.6487	2.4263	2.2147
ENSMUSG00000039621	6.0947	16.6773	17.9067
ENSMUSG00000039623	11.6647	4.642	4.9517
ENSMUSG00000039652	2.214	0.826	0.766
ENSMUSG00000039699	0.527	2.7487	1.8617
ENSMUSG00000039735	1.12	0.0293	0.0233
ENSMUSG00000039740	29.7133	13.0453	11.981
ENSMUSG00000039783	2.524	0.0423	0.0593
ENSMUSG00000039959	1.813	5.7293	4.8167
ENSMUSG00000039982	8.693	1.95	1.4423
ENSMUSG00000040013	1.372	0.2543	0.1513
ENSMUSG00000040061	16.1317	70.2227	76.6917
ENSMUSG00000040187	1.6237	0.0983	0.0767
ENSMUSG00000040209	8.7373	4.0727	3.5513
ENSMUSG00000040253	0.4517	2.1023	2.3393
ENSMUSG00000040272	2.869	6.9083	5.9663
ENSMUSG00000040274	92.7323	32.2247	31.59
ENSMUSG00000040283	5.0633	32.4823	29.038
ENSMUSG00000040296	7.713	16.543	16.097
ENSMUSG00000040297	27.0623	10.612	10.6263
ENSMUSG00000040314	293.3983	1.316	0.4713
ENSMUSG00000040350	0.341	1.1637	1.909
ENSMUSG00000040359	23.2367	9.8417	10.555
ENSMUSG00000040433	13.7527	40.591	40.8323
ENSMUSG00000040447	16.9373	146.0247	146.7897
ENSMUSG00000040479	16.5787	101.473	106.713
ENSMUSG00000040502	6.676	2.3913	2.6647
ENSMUSG00000040562	0.439	46.7017	41.7867
ENSMUSG00000040613	3.668	1.2587	1.2633
ENSMUSG00000040627	0.39	0	0
ENSMUSG00000040732	9.9653	0.3333	0.1333

ENSMUSG00000040913	13.396	4.983	4.9293
ENSMUSG00000040998	1.3153	0.2577	0.2103
ENSMUSG00000041058	68.6653	11.2703	10.6443
ENSMUSG00000041096	20.9957	7.6813	7.488
ENSMUSG00000041135	7.958	3.5187	3.842
ENSMUSG00000041168	108.687	46.914	47.549
ENSMUSG00000041187	9.222	24.686	25.1243
ENSMUSG00000041220	7.8437	29.2953	29.644
ENSMUSG00000041235	0.544	0.041	0.1217
ENSMUSG00000041372	0.4977	0.1937	0.1867
ENSMUSG00000041396	4.901	13.8153	15.207
ENSMUSG00000041607	9.7917	22.646	24.4823
ENSMUSG00000041608	1.4897	0.1333	0.0397
ENSMUSG00000041679	2.4483	0.8023	0.687
ENSMUSG00000041836	13.299	41.829	40.5737
ENSMUSG00000041890	40.033	19.3263	17.2923
ENSMUSG00000041895	6.965	16.3323	18.9947
ENSMUSG00000041911	3.7837	9.285	9.3957
ENSMUSG00000041959	94.6797	257.407	265.144
ENSMUSG00000042042	182.3963	34.9153	34.4043
ENSMUSG00000042082	36.479	1.3897	0.7573
ENSMUSG00000042148	27.2903	11.5057	13.146
ENSMUSG00000042182	0.2243	0	0
ENSMUSG00000042207	30.1107	14.848	12.7347
ENSMUSG00000042228	17.6253	87.8833	82.0443
ENSMUSG00000042249	4.76	2.2437	2.1677
ENSMUSG00000042275	25.3947	8.5463	11.254
ENSMUSG00000042320	1.5607	0.4517	0.5013
ENSMUSG00000042333	9.5313	1.0497	0.8857
ENSMUSG00000042345	0.612	22.8157	25.0263
ENSMUSG00000042349	8.681	3.6467	3.1647
ENSMUSG00000042350	40.6637	15.9147	14.728

ENSMUSG00000042354	28.8687	58.1097	62.819
ENSMUSG00000042532	0.1893	0	0.015
ENSMUSG00000042594	9.0497	60.543	73.9727
ENSMUSG00000042622	0.7697	2.8827	3.518
ENSMUSG00000042647	3.85	1.5357	1.749
ENSMUSG00000042684	17.1583	8.464	8.0233
ENSMUSG00000042688	13.7547	31.2743	35.5217
ENSMUSG00000042745	6.226	48.925	49.2907
ENSMUSG00000042751	0.2733	0.6477	1.07
ENSMUSG00000042807	0	0.0773	0.051
ENSMUSG00000042842	0.7327	4.5547	4.408
ENSMUSG00000043019	66.9143	8.2737	7.6877
ENSMUSG00000043257	7.8917	2.8483	2.8387
ENSMUSG00000043510	16.3977	6.2467	7.1377
ENSMUSG00000043639	0.3113	0	0.0157
ENSMUSG00000043733	17.8933	36.695	40.504
ENSMUSG00000043881	0.1397	0.5783	0.511
ENSMUSG00000043969	0.1523	0	0
ENSMUSG00000044199	4.3547	2.08	1.6687
ENSMUSG00000044258	0.4233	33.9847	51.9463
ENSMUSG00000044303	0	0.319	0.634
ENSMUSG00000044350	2.7213	5.801	6.602
ENSMUSG00000044456	38.972	15.939	16.7613
ENSMUSG00000044734	108.7277	5.1547	0.6517
ENSMUSG00000044786	1.7093	12.4283	17.702
ENSMUSG00000044952	1.2223	0.465	0.4323
ENSMUSG00000044968	2.4747	0.4723	0.5473
ENSMUSG00000045038	4.4137	9.53	11.1457
ENSMUSG00000045092	1.0523	7.6763	5.704
ENSMUSG00000045102	0.7973	0.227	0.16
ENSMUSG00000045140	3.273	6.7757	6.556
ENSMUSG00000045216	8.5783	23.906	29.0503

ENSMUSG00000045282	6.9237	2.7307	2.9153
ENSMUSG00000045409	15.9553	5.9673	5.638
ENSMUSG00000045414	27.838	7.8713	7.2257
ENSMUSG00000045466	6.9093	3.131	3.441
ENSMUSG00000045502	1.0937	0.193	0
ENSMUSG00000045671	6.1527	26.3593	29.807
ENSMUSG00000045827	0.4803	3.4063	3.0623
ENSMUSG00000046179	80.2127	24.4643	26.219
ENSMUSG00000046223	2.3713	16.859	18.9863
ENSMUSG00000046324	25.2023	11.9477	12.0577
ENSMUSG00000046668	11.883	25.8287	26.954
ENSMUSG00000046711	69.6923	160.6903	174.29
ENSMUSG00000046806	3.287	16.8967	15.3393
ENSMUSG00000046879	9.3377	26.2497	26.7307
ENSMUSG00000046962	19.4737	7.0603	6.563
ENSMUSG00000047180	5.868	13.375	13.6527
ENSMUSG00000047250	7.1977	168.6627	168.4303
ENSMUSG00000047343	0	0.235	0.3637
ENSMUSG00000047407	46.5107	11.0937	12.0283
ENSMUSG00000047492	0.883	0.068	0.0833
ENSMUSG00000047496	0.057	0	0.0027
ENSMUSG00000047648	12.677	5.587	5.785
ENSMUSG00000047746	1.1267	0.4427	0.5373
ENSMUSG00000047822	11.508	1.0387	1.74
ENSMUSG00000047921	8.4543	22.6323	24.4763
ENSMUSG00000047953	0.2133	0.8133	1.2753
ENSMUSG00000048148	0.02	1.035	1.0193
ENSMUSG00000048376	25.21	214.9043	249.221
ENSMUSG00000048410	6.1093	2.438	2.3057
ENSMUSG00000048478	1.493	0.355	0.6807
ENSMUSG00000048612	0.3093	2.4963	2.8193
ENSMUSG00000048652	4.643	0.4837	0.198

ENSMUSG00000048897	5.5443	15.175	14.1503
ENSMUSG00000048992	0.234	0	0
ENSMUSG00000049090	38.606	7.2633	11.2903
ENSMUSG00000049184	6.1677	2.166	2.181
ENSMUSG00000049225	15.8863	61.596	55.037
ENSMUSG00000049577	22.7263	220.257	270.084
ENSMUSG00000049709	0.7017	2.6713	2.156
ENSMUSG00000049775	583.109	1824.687	1754.8043
ENSMUSG00000049791	5.7707	2.2287	1.552
ENSMUSG00000049858	10.6517	5.1773	4.7337
ENSMUSG00000050014	1.5237	6.3503	5.5917
ENSMUSG00000050199	8.13	1.9727	2.091
ENSMUSG00000050377	0.1573	0	0
ENSMUSG00000050675	0.4153	9.3507	10.6503
ENSMUSG00000050965	29.0703	79.6293	90.5843
ENSMUSG00000051224	5.4147	1.9663	1.5277
ENSMUSG00000051457	69.2527	28.6077	26.6053
ENSMUSG00000051495	57.8213	12.405	12.9157
ENSMUSG00000051590	0.1833	1.301	1.5613
ENSMUSG00000051615	4.3363	33.4683	38.7007
ENSMUSG00000051650	108.7097	44.27	49.8263
ENSMUSG00000051890	1.988	0.7217	0.5407
ENSMUSG00000052040	28.6893	10.1423	9.1663
ENSMUSG00000052087	4.8663	12.6217	11.8937
ENSMUSG00000052234	724.6347	11.773	1.7873
ENSMUSG00000052293	24.9883	8.6943	6.1947
ENSMUSG00000052296	138.865	63.9013	62.7887
ENSMUSG00000052353	0.0197	0.1423	0.0903
ENSMUSG00000052435	74.201	9.0293	4.7073
ENSMUSG00000052751	7.3507	3.1697	3.495
ENSMUSG00000052837	9.8693	23.259	45.845
ENSMUSG00000053040	2.757	1.1333	0.9303

ENSMUSG00000053113	1.7033	6.3517	8.8407
ENSMUSG00000053192	3.2813	7.334	7.8573
ENSMUSG00000053226	1.708	0.4917	0.5093
ENSMUSG00000053338	2.439	0.0447	0.089
ENSMUSG00000053475	1.6703	0	0
ENSMUSG00000053477	9.6447	20.6083	22.1413
ENSMUSG00000053560	14.055	79.7703	125.2843
ENSMUSG00000053604	39.454	10.563	11.0207
ENSMUSG00000053693	1.4263	0.3927	0.346
ENSMUSG00000053914	1.2707	0.093	0.0303
ENSMUSG00000054021	29.861	86.3943	77.8333
ENSMUSG00000054115	10.435	23.6853	21.7087
ENSMUSG00000054136	9.2913	1.292	0.722
ENSMUSG00000054191	31.1497	2.096	1.893
ENSMUSG00000054200	1.522	0.2243	0.214
ENSMUSG00000054385	0.7387	5.408	5.4137
ENSMUSG00000054469	41.8197	10.9903	11.7723
ENSMUSG00000054568	0.0237	0.2733	0.2657
ENSMUSG00000054619	115.955	8.015	7.2547
ENSMUSG00000054675	0.418	29.3883	56.5377
ENSMUSG00000054814	4.1087	8.8143	8.8573
ENSMUSG00000054892	1.4537	0.267	0.233
ENSMUSG00000054931	0.3413	0.9407	1.3373
ENSMUSG00000055172	14.2497	2.2427	2.3163
ENSMUSG00000055202	0.192	0.714	0.8797
ENSMUSG00000055210	1.7263	0.732	0.7423
ENSMUSG00000055447	336.1433	80.5837	73.1483
ENSMUSG00000055485	1.4777	13.463	14.926
ENSMUSG00000055835	12.0263	3.7213	3.5827
ENSMUSG00000056130	6.4747	2.1387	1.8607
ENSMUSG00000056215	0.7617	0.084	0.116
ENSMUSG00000056220	21.4993	181.7147	175.578

ENSMUSG00000056234	279.0897	65.2103	50.5037
ENSMUSG00000056399	586.8093	0.988	0.4387
ENSMUSG00000056515	17.6293	63.1153	71.167
ENSMUSG00000056643	2.7583	0.0113	0.011
ENSMUSG00000056656	0	0.15	0.1043
ENSMUSG00000056749	16.3493	7.6947	7.906
ENSMUSG00000057069	20.9973	6.812	6.2973
ENSMUSG00000057074	0	0.106	0.157
ENSMUSG00000057596	0	5.4483	5.1383
ENSMUSG00000057729	0.6733	4.5693	2.228
ENSMUSG00000058099	6.073	16.3653	13.656
ENSMUSG00000058186	1.26	3.5893	2.7507
ENSMUSG00000058216	2.1793	0	0.029
ENSMUSG00000058756	19.73	8.2963	8.872
ENSMUSG00000059430	23.752	110.724	89.516
ENSMUSG00000059456	9.8637	42.8943	51.537
ENSMUSG00000059708	1.686	0.541	0.2593
ENSMUSG00000059895	46.4373	240.7433	261.726
ENSMUSG00000059923	189.5623	85.416	89.1407
ENSMUSG00000059970	18.1747	6.104	6.0503
ENSMUSG00000060002	10.604	1.5747	1.4713
ENSMUSG00000060063	29.1813	78.7987	86.3553
ENSMUSG00000060147	9.9443	4.9423	4.2977
ENSMUSG00000060216	36.29	159.3263	157.0657
ENSMUSG00000060224	4.4807	11.128	12.096
ENSMUSG00000060376	27.4133	11.5557	10.987
ENSMUSG00000060470	7.4083	14.9617	19.327
ENSMUSG00000060579	0.7277	2.5797	2.361
ENSMUSG00000060600	13.2017	64.3007	53.5517
ENSMUSG00000061046	4.8543	1.8843	2.297
ENSMUSG00000061143	2.0853	8.3347	9.8603
ENSMUSG00000061186	8.1437	17.791	17.1503

ENSMUSG00000061665	14.0713	29.4363	29.8593
ENSMUSG00000061751	0.148	5.1693	6.267
ENSMUSG00000061815	0	5.1803	6.2833
ENSMUSG00000062232	21.516	3.1893	3.437
ENSMUSG00000062593	9.445	53.3593	81.871
ENSMUSG00000062713	1.3313	0.0713	0.0403
ENSMUSG00000062826	0.221	0.0077	0
ENSMUSG00000062861	2.7297	18.356	19.814
ENSMUSG00000062901	17.6267	7.9457	7.3353
ENSMUSG00000062937	15.8527	582.3593	577.6717
ENSMUSG00000063129	2.308	0.082	0.251
ENSMUSG00000063160	3.7703	1.3403	0.7807
ENSMUSG00000063275	6.433	13.506	13.1267
ENSMUSG00000063382	3.4737	8.4923	9.6083
ENSMUSG00000063535	2.3997	1.1	1.0793
ENSMUSG00000063683	7.7987	0.2093	0.0783
ENSMUSG00000063851	0.4877	1.3877	2.094
ENSMUSG00000063873	1.4637	0.3423	0.728
ENSMUSG00000064120	12.324	6.1237	5.2917
ENSMUSG00000064368	914.4783	436.1683	428.339
ENSMUSG00000066007	0.5777	1.1837	1.2437
ENSMUSG00000066026	11.704	82.5533	93.5117
ENSMUSG00000066440	10.2213	4.1167	3.978
ENSMUSG00000066442	4.7003	0.5683	0.9047
ENSMUSG00000066687	25.5997	8.829	8.8663
ENSMUSG00000066735	34.5157	13.9583	15.337
ENSMUSG00000067399	1.4433	0.0103	0.0577
ENSMUSG00000067851	121.1403	30.3047	27.524
ENSMUSG00000068129	74.9343	31.7613	29.0027
ENSMUSG00000068744	9.395	22.2527	20.5273
ENSMUSG00000069830	7.3583	0.0167	0.0053
ENSMUSG00000069833	0.0423	1.423	1.8133

ENSMUSG00000070323	0.495	0.0633	0.0097
ENSMUSG00000070348	0.111	0.9433	0.8117
ENSMUSG00000070732	3.3383	1.3207	1.4267
ENSMUSG00000070780	1.5913	0.009	0.0063
ENSMUSG00000071076	72.292	14.966	17.596
ENSMUSG00000071113	0.2023	0.6397	0.6713
ENSMUSG00000071470	3.1303	7.8413	6.7833
ENSMUSG00000071552	2.317	0.7693	0.9197
ENSMUSG00000071637	16.21	3.5043	3.533
ENSMUSG00000071648	6.805	1.3623	1.0573
ENSMUSG00000071713	438.0577	17.0993	6.8077
ENSMUSG00000071714	389.6727	15.9123	9.9363
ENSMUSG00000072419	0.2423	0.0097	0.011
ENSMUSG00000072889	25.7043	9.111	10.2697
ENSMUSG00000072915	1.407	0.6253	0.5457
ENSMUSG00000073802	0.0143	1.05	1.2467
ENSMUSG00000073987	38.153	12.882	11.3853
ENSMUSG00000074028	0.6403	0.0513	0.02
ENSMUSG00000074151	1.0983	4.918	5.955
ENSMUSG00000074220	5.555	2.134	1.8417
ENSMUSG00000074227	57.7257	14.0613	14.5753
ENSMUSG00000074305	6.839	40.8877	43.9823
ENSMUSG00000074344	0	1.3103	1.193
ENSMUSG00000074527	18.9203	5.9583	6.161
ENSMUSG00000074604	6.8113	80.348	76.5417
ENSMUSG00000074794	8.1897	2.4927	3.3113
ENSMUSG00000074874	0.1907	16.756	22.8703
ENSMUSG00000074923	1.055	3.4593	4.111
ENSMUSG00000075054	4.207	11.936	10.83
ENSMUSG00000075122	0.0723	0.717	0.595
ENSMUSG00000075254	5.2183	1.7183	1.8193
ENSMUSG00000075706	58.9553	195.2347	206.3187

ENSMUSG00000076435	11.243	2.996	2.6237
ENSMUSG00000078249	167.697	374.4287	405.5803
ENSMUSG00000078498	0.262	0.0067	0.007
ENSMUSG00000078653	1.5353	0.471	0.2533
ENSMUSG00000078851	2.5433	0.61	0.528
ENSMUSG00000078853	0.5487	1.9837	1.8617
ENSMUSG00000078865	9.19	4.2867	4.228
ENSMUSG00000078866	22.9207	7.0233	6.6777
ENSMUSG00000078872	27.482	0.8553	0.9997
ENSMUSG00000079033	1.7327	0.0957	0.0603
ENSMUSG00000079110	0.3103	6.8277	7.9707
ENSMUSG00000079162	0.179	0	0
ENSMUSG00000079184	5.4803	19.321	19.5663
ENSMUSG00000079197	20.8397	44.4483	43.2113
ENSMUSG00000079334	14.3987	6.3557	5.334
ENSMUSG00000079427	8.6593	2.6373	2.862
ENSMUSG00000079442	3.264	6.7717	7.6857
ENSMUSG00000079451	0.5847	0	0
ENSMUSG00000079563	0.3523	9.252	7.8567
ENSMUSG00000085793	11.3563	5.571	4.9703
ENSMUSG00000089665	0.16	1.6797	1.586
ENSMUSG00000089694	0	0.1977	0.1473
ENSMUSG00000089876	7.383	2.9553	3.0557
ENSMUSG00000089951	0.5663	0.034	0.035
ENSMUSG00000090353	4.0503	1.58	1.3417
ENSMUSG00000090958	0.0053	0.3	0.2607
ENSMUSG00000091455	0.8497	0.0107	0
ENSMUSG00000091780	4.3837	19.3357	19.821
ENSMUSG00000093769	0.949	0.204	0.353
ENSMUSG00000094081	0.9413	0.3493	0.3323
ENSMUSG00000095545	53.859	21.8583	23.1947
ENSMUSG00000095687	63.0003	28.9183	29.6813

ENSMUSG00000096370	2.69	0.6023	0.5753
ENSMUSG00000096472	5.559	30.3647	28.8287
ENSMUSG00000096740	4.095	8.215	8.7107
ENSMUSG00000097084	0.1747	0	0
ENSMUSG00000099583	0.79	0.196	0.111
ENSMUSG00000099974	3.055	0.0477	0
ENSMUSG00000110206	4.1377	25.7773	27.543
ENSMUSG00000110277	6.6867	2.5987	2.313
ENSMUSG00000112023	4.1593	14.175	23.4983
ENSMUSG00000114635	0	1.7363	1.5207
ENSMUSG00000116564	11.0133	23.0713	23.8167
ENSMUSG00000118669	1.1767	0.3167	0.442
